# Nickel catalyzed multicomponent stereodivergent synthesis of olefins enabled by electrochemistry, photocatalysis and photo-electrochemistry

**DOI:** 10.1038/s41467-022-30985-2

**Published:** 2022-06-10

**Authors:** Chen Zhu, Huifeng Yue, Magnus Rueping

**Affiliations:** 1 KAUST Catalysis Center (KCC), King Abdullah University of Science and Technology (KAUST), Thuwal, 23955-6900 Saudi Arabia; 2 RWTH Aachen University, Forckenbeckstrasse 55, 52074 Aachen, Germany

**Keywords:** Synthetic chemistry methodology, Photocatalysis, Electrochemistry

## Abstract

Trisubstituted alkenes are important organic synthons and have broad applications in the synthesis of many pharmaceuticals and materials. The stereoselective synthesis of such compounds has long been a research focus for organic researchers. Herein, we report a three-component, reductive cascade, cross-coupling reaction for the arylalkylation of alkynes. A wide range of trisubstituted alkenes are obtained in good to high yields with excellent chemo- and stereoselectivity by switching between electrochemistry and photocatalysis. The *E* isomer of the product is obtained exclusively when the reaction is conducted with electricity and nickel, while the *Z* isomer is generated with high stereoselectivity when photo- and nickel dual catalysts are used. Moreover, photo-assisted electrochemically enabled nickel catalyzed protocol is demonstrated to selectively deliver *Z*-trisubstituted alkenes without the addition of photocatalysts.

## Abstract (web summary)

The construction of trisubstituted alkenes with high stereoselectivity is challenging. Here, the authors realize the stereodivergent synthesis of such compounds via switching between electrochemistry, photochemistry and photoelectrochemistry.

## Introduction

Trisubstituted alkenes are important organic synthons and have broad applications in the synthesis of many drugs, materials, and fine chemicals^1,2^. Applying multicomponent reactions (MCRs) to perform one-pot alkyne difunctionalizations^3,4^, which involves the installation of two chemical bonds across triple bonds in one synthetic operation, is one of the most straightforward and powerful strategies for preparing valuable trisubstituted alkenes. To date, the construction of trisubstituted alkenes with high stereoselectivity continues to be a challenging topic for organic chemists^5^. Although great efforts have been made^6^, the established methodologies are confined to one stereoisomer generation^7–10^ or require starting materials with fixed configurations^11^, and few cases have successfully produced the corresponding alkenes in both *Z* and *E* stereoselectivities^12^.

**Fig. 1:**
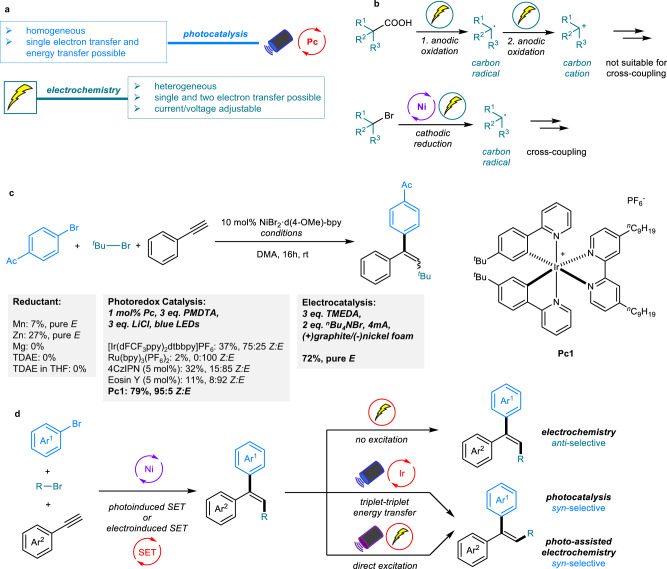
Nickel-catalyzed arylalkylation of alkynes. **a** The advantages of photocatalysis and electrochemistry. **b** Limitation of the electrochemical oxidation pathway and potential of the electrochemical reduction pathway in the cross-coupling field. **c** Initial results of nickel-catalyzed reductive cascade cross-couplings via the traditional reductant, electrochemical, and photocatalyzed pathways. **d** Stereocontrol of alkenes by switching between electrochemistry and photocatalysis. TDAE, tetrakis(dimethylamino)-ethylene; DMA, dimethylacetamide; PMDTA, N,N,N′,N′′,N′′-pentamethyldiethylentriamin; TMEDA, tetramethylethylenediamine; bpy, 2,2′-bipyridine.

In the last five years, photocatalysis and electrochemistry have emerged as two powerful platforms for the construction of a wide range of organic molecules^13–16^. In particular, dual catalysis that combines transition metals with photocatalysts^17–29^ or electricity^30–42^ provides opportunities for the development of more general and efficient catalytic methodologies^43^. In contrast to traditional cross-coupling processes in which the oxidative addition or reductive elimination steps often require high temperatures or the assistance of elaborated ligands^44^, these dual catalytic strategies can usually be applied under mild reaction conditions. Considering the reaction mechanisms, photocatalysts or electricity can act as both oxidant and reductant, enabling single-electron transfers (SET) to reactants or transition metal intermediates. In addition to their fundamentally common ground, photocatalysis and electrochemistry also have respective catalytic advantages. Homogeneous photocatalysis possesses one more catalytic mode involving an energy transfer (ET) pathway since the excited photocatalyst can undergo Dexter energy transfer with the substrate or metal complex, thus expanding the types of organic transformations^45,46^. For heterogeneous electrochemistry, the current or voltage can be adjusted to the reaction requirements, allowing unprecedented organic transformations that cannot occur with chemical oxidants or reductants (Zn/Mn). Thus, the process is an appealing synthetic alternative considering that the availability of photosensitizers for targeted adjustment and appropriate redox potentials with cross-coupling catalysts is not always given (Fig. 1a). Recently, elegant electrochemical oxidative cross-coupling reactions have been reported^47,48^. However, electrochemical reductive cross-couplings remain less developed^49^.

In recent years, our group has contributed to the field of combined metal/photoredox dual catalysis and has developed cascade reactions that proceed with high chemo-, regio-, and stereoselectivity under mild conditions. In these cases, the arylsulfonylation cross-coupling products of trisubstituted alkenes can be obtained with either *anti*- or *syn-*selectivity by choosing an appropriate photocatalyst with suitable triplet energy^12^. However, when we extended this photoredox and nickel dual-catalyzed redox neutral cascade protocol to the more interesting alkylarylations of alkynes, only *syn* addition trisubstituted alkenes bearing three different carbon-linked groups could be obtained^9^. The reason for this limitation is that a photocatalyst with high triplet energy must be used for the generation of alkyl radicals, thus leading to the inevitable *E* to *Z* isomerization of the generated products via an energy transfer process. To address this issue and given the underlying principle in both photoredox and electrochemical processes is electron transfer, we supposed that electrochemical nickel catalysis would be a good choice to address this problem. However, under electrochemical conditions, the generation of alkyl radicals via an oxidation pathway is typically difficult due to anodic overoxidation that produces alkyl cations^50^. Therefore, we turned our attention to the reductive pathway. In this case, the radical can be generated from alkyl halides via a reduction pathway and participate in the nickel catalytic cycle without the late-stage energy transfer process that occurs in photo/Ni dual catalysis, thus providing the possibility for the development of electrochemical nickel catalyzed reductive cascade cross-couplings to form *anti*-addition products (Fig. 1b).

Our initial results showed that traditional reductants such as Mn^51^, Zn, Mg, and TDAE^52^ were inefficient in this three-component cross-electrophile coupling, affording low or no yield of the desired product. Photocatalysts including [Ir(dFCF_3_ppy)_2_dtbbpy]PF_6_, Ru(bpy)_3_(PF_6_)_2_, 4CzIPN and Eosin Y all showed low catalytic reactivity or generated products with poor stereoselectivity (Fig. 1c).

In this work, photocatalyst **Pc1** bearing a suitable triplet energy could selectively generate the *syn*-selective isomer as the main product. Significantly, when we conducted the reaction under electrochemical conditions, the *anti-*selective isomer was obtained as the main product in good yield with excellent stereoselectivity since the ET process was avoided. Thus, stereocontrol was realized to give both isomers of the product via elaborate switching between electrochemistry and photocatalysis. Moreover, *syn*-selective isomer could also be obtained via photo-assisted electrochemistry in the absence of photocatalyst (Fig. 1d).

## Results

### Substrate scope

**Fig. 2:**
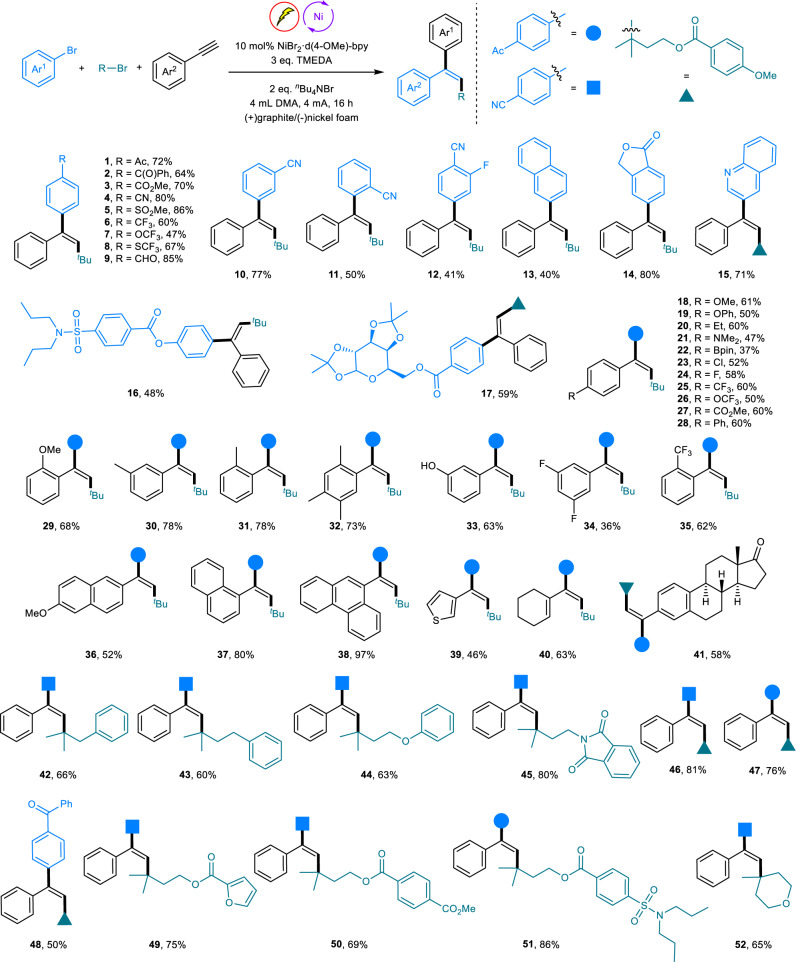
Scope of the nickel catalyzed electrochemical arylalkylation of alkynes. Reactions were performed with 0.2 mmol of aryl bromides, 0.4 mmol of alkynes, and 0.6 mmol of alkyl bromide. Yields are of the isolated products.

The scope of the nickel catalyzed electrochemical reductive cascade cross-coupling reaction was explored next (Fig. 2). A series of aryl bromides underwent this process smoothly, giving the corresponding three-component product in moderate to high yields. The transformation proceeded with not only exclusive stereoselectivity but also high chemoselectivity, which was well illustrated by the tolerance of different types of functional groups, such as ketones, esters, cyanos and sulfones as well as trifluoromethyl, trifluoromethoxy and trifluoromethylthio (**1**–**8**). Steric hindrance has some effect on the efficiency of the transformation, as *ortho* cyano-substituted aryl bromide gave a lower yield than that of the para-surrogate (**10** and **11**). Notably, aryl bromide bearing a reactive aldehyde group was also a suitable substrate for this reaction and gave the arylalkylation product in an 85% yield (**9**). Di-substituted and naphthyl bromides reacted well, albeit with moderate efficiency (**12** and **13**). Additionally, bicyclic and heteroaryl bromides participated in the reaction in high yields (**14** and **15**). Significantly, aryl bromides derived from probenecid and galactopyranose could also be applied, demonstrating that this cross-electrophile coupling is practical and can be broadly applied (**16** and **17**). Regarding alkyne coupling partners, a wide range of electronically and sterically diverse terminal alkynes could undergo this electrochemical process, delivering the corresponding *anti*-addition product in moderate to excellent yields. Ethynylbenzenes containing electron-donating and electron-withdrawing functional groups such as ethers, alkyl, amine, fluoro, trifluoromethyl, trifluoromethoxy, and methyl ester were all amenable to this procedure (**18**–**28**). Remarkably, a number of sensitive functional groups, including borate ester, chloride, and unprotected free hydroxy groups, remained untouched during the reaction, providing the possibility for late functionalization (**22**, **23**, and **33**). Unlike aryl bromides, the steric properties of the alkynes had almost no effect on the catalytic activity (**29**–**35**). Alkynes bearing π-extended systems reacted well (**36**–**38**). Moreover, 3-ethynylthiophene, cyclohexenyl alkyne, and estrone-derived alkyne could also be easily converted to the products (**39**–**41**). In addition, a variety of linear tertiary alkyl bromides bearing phenyl, ether, phthalimidyl, and ester groups coupled with the alkyne and aryl halides effectively (**42**–**50**). Importantly, alkyl bromides containing probenecid motifs could also give the product in high yield (**51**). Additionally, the cyclic tertiary bromide bearing a tetrahydropyran moiety proved to be an effective substrate, affording the desired product in a good yield (**52**).

**Fig. 3:**
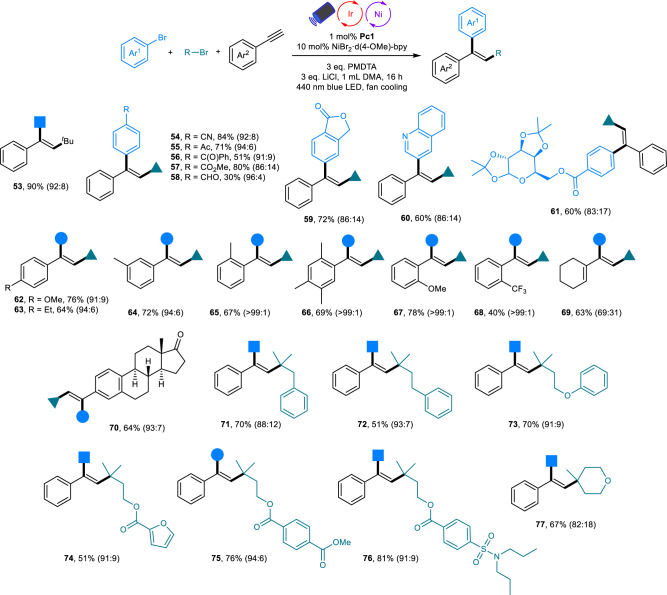
Scope of the photoredox/nickel dual catalyzed arylalkylation of alkynes. Reactions were performed with 0.4 mmol of aryl bromides, 0.2 mmol of alkynes, and 0.6 mmol of alkyl bromide. Yields are of isolated products. The values in parentheses represent the ratio of the two isomers (**P**_***syn***_:**P**_***anti***_), which was determined by ^1^H NMR analysis.

In contrast, trisubstituted alkenes were selectively obtained in a *syn* addition when the arylalkylation reactions were performed under photoredox/nickel dual catalysis, which is due to the involvement of both SET and ET processes (Fig. 3). In this regard, the SET process contributes to the formation of product, while the ET process or direct excitation leads to the *E/Z* isomerization. The depletion of *E* isomer and accumulation of *Z* isomer may be attributed to the difference of conjugation level and triplet state energy of the resulting alkene isomers (Supplementary Fig. 22)^12,53^. Notably, the addition of LiCl as additive improved the yield (Supplementary Table 6, entries 1 and 5). Regarding the scope of this protocol, many electron-poor aryl halides, electron-rich heterocyclic halides and natural product-derived surrogates all reacted in good to high yields with good to excellent steroselectivities (**53**–**61**). Alkynes containing electron-donating and electron-withdrawing functional groups as well as estrone-derived alkynes participated with excellent stereocontrol (**62**–**70**). Vinyl alkyne also smoothly underwent this reductive cascade reaction, albeit with moderate stereoselectivity (**69**). Notably, *ortho*-substituents on the alkyne aromatic ring led to the formation of pure *syn* addition products (**65**–**68**). Linear alkyl bromides bearing diverse motifs and cyclic alkyl bromides were all suitable substrates for the efficient generation of *syn* addition products (**71**–**77**).

**Fig. 4:**
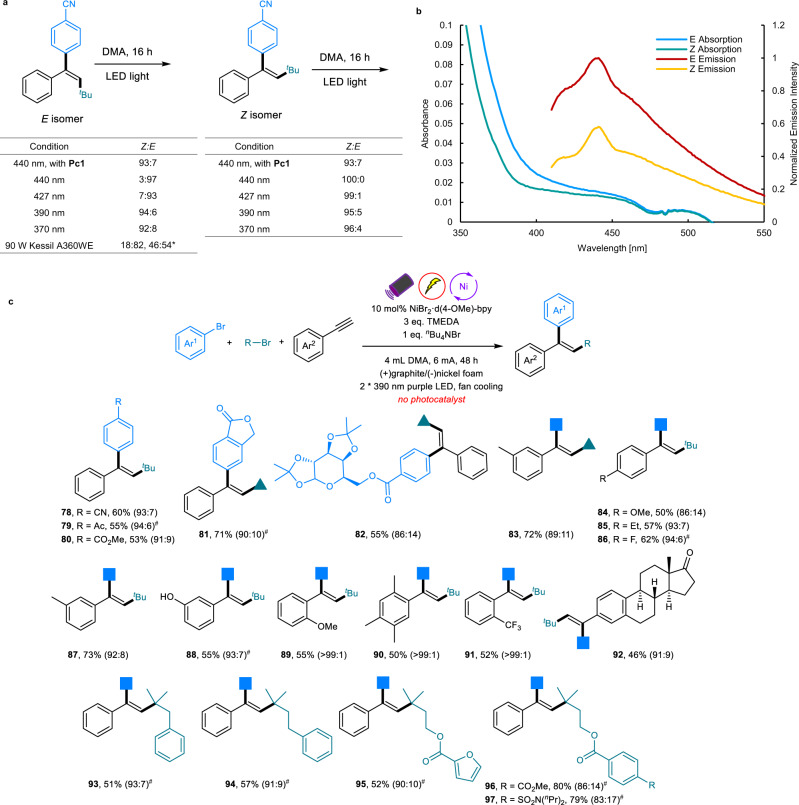
Mechanistic study and scope of the photo-assisted electrochemically nickel catalyzed arylalkylation of alkynes. **a** Isomerization control reactions. **b** UV/Vis absorption spectroscopy and fluorescence emission spectroscopy of *E* and *Z* isomers. **c** Reactions were performed with 0.2 mmol of aryl bromides, 0.4 mmol of alkynes, and 0.6 mmol of alkyl bromide. Yields are of the isolated products. The values in parentheses represent the ratio of the two isomers (**P**_***syn***_:**P**_***anti***_), which was determined by ^1^H NMR analysis. *Reaction performed in DMSO. ^#^Reaction conditions: aryl bromides (0.2 mmol, 1 equiv.), alkynes (0.4 mmol), alkyl bromides (0.6 mmol), NiBr_2_·d(4-OMe)-bpy (10 mol%), TMEDA (3 equiv.), ^*n*^Bu_4_NBr (2 equiv.) in 4 mL DMA and electrolysis for 16 h at 4 mA using a graphite anode and nickel foam cathode. The reaction mixture was then directly irradiated with 2*390 nm purple LEDs for 24 h.

During the mechanistic investigations, we found that using 440 nm blue LEDs together with **Pc1** could lead to efficient isomerization of the *E* isomer to the *Z* isomer, while in the absence of a photocatalyst, 440 nm irradiation resulted in no obvious isomerization. Further evaluation of the light sources revealed that 390 nm purple LEDs also led to efficient isomerization of the *E* isomer to the *Z* isomer without a photocatalyst, while the *Z* isomer in contrast did not isomerize to the *E* isomer (Fig. 4a). UV/Vis absorption spectroscopy of the *E* and *Z* isomers in DMA at the same concentration showed that the *E* isomer has a higher absorption intensity than that of the *Z* isomer at 390 nm. Additionally, fluorescence emission spectroscopy indicated that the *E* isomer has a much higher emission intensity than that of the *Z* isomer when excited by a 390 nm light source (Fig. 4b). Both of these observations rationalized the results illustrated in Fig. 4a. Considering these observations, we wondered whether we could achieve the arylalkylation of alkynes in a *syn* addition manner via the combination of 390 nm LED irradiation with an electrochemically nickel catalytic cycle and without the necessity of a photocatalyst. Gratifyingly, after minor optimizations, this photo-assisted electrochemical protocol worked efficiently for a series of aryl halides, alkynes and alkyl bromides, affording *syn* addition products with good to excellent stereoselectivities. Additionally, the reaction could be conducted in a sequential way in which the reaction mixture was directly irradiated by purple LED after the nickel-electrocatalytic process was complete (Fig. 4c). Likewise, *ortho*-substituents on the aromatic ring of alkyne could also deliver pure *syn* addition products (**89**–**91**). Of particular note is that alkynes bearing hydroxyl groups that failed in photo/nickel dual catalysis could give the corresponding *syn-addition* product with high stereoselectivity under these conditions (**88**), showcasing the advantage of these three catalytic pathways and their mutual complementation.

### Synthetic application

**Fig. 5:**
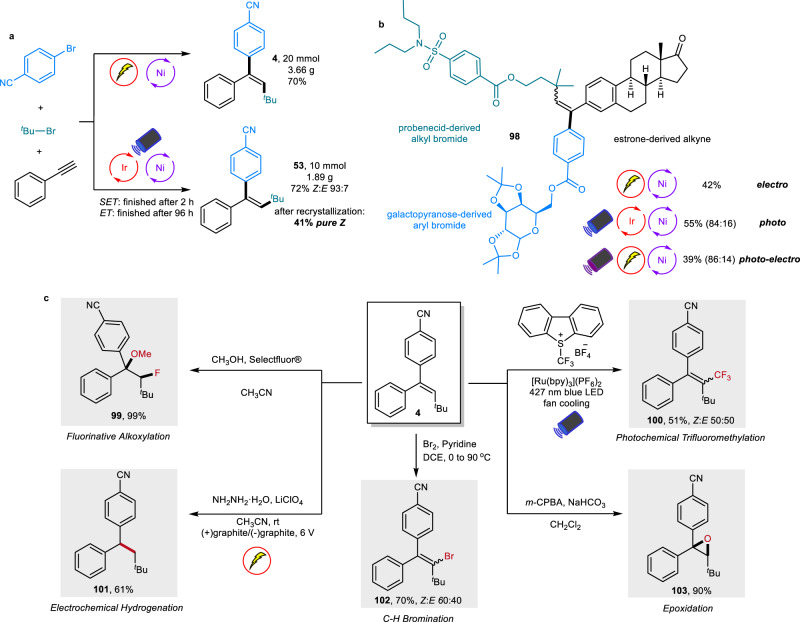
Applications. **a** Scale up reactions for both the *E* and *Z* isomers. **b** Complex molecule synthesis via three different approaches. **c** Further functionalization of the generated trisubstituted alkene.

Additionally, scale-up reactions were conducted for both the nickel-electrochemistry and nickel-photocatalysis, and no loss of yield and stereoselectivity occurred (Fig. 5a). Notably, although the *Z* isomer (**53**) was generated in a 93:7 ratio after nickel-photocatalysis, a pure *Z* product could be obtained after one cycle of simple recrystallization. Furthermore, via these three methods, both *P*_*anti*_ and *P*_*syn*_ products of a more complex molecule (**98**) were synthesized in one simple step from three natural product-derived starting materials bearing probenecid, estrone, and galactopyranose motifs with good stereoselectivity (Fig. 5b). To further assess the synthetic potential of the newly developed methodologies in the construction of molecular complexity, a series of late-stage functionalizations of the generated trisubstituted alkene have also been performed despite its large steric hindrance. As illustrated in Fig. 5c, fluorinative alkoxylation, electrochemical hydrogenation, photochemical trifluoromethylation, epoxidation, and vinyl C-H bromination were all successfully realized, generating the corresponding product in good to excellent yields (**99**–**103**). These successful applications strongly indicate the high practicability of these mild reductive cascade cross-couplings.

### Mechanistic studies

To further investigate the mechanism, a series of mechanistic experiments were performed. First, radical trapping experiments were conducted with two equivalents of TEMPO (2,2,6,6-tetramethyl-1-piperidinyloxy) or 1,1-diphenylethylene. They both suppressed the reaction completely, and the product with tertiary butyl group addition to 1,1-diphenylethylene was observed by GC–MS, suggesting the involvement of an alkyl radical in the transformation (Supplementary Information section 10). Stern-Volmer quenching experiments of all three starting materials and amine reductant show that only PMDTA quenches the photocatalyst **Pc1**. Additionally, cyclic voltammetry (CV) measurements show that both PMDTA (*E*_h/2_ = 0.63 V *vs*. SCE) and TMEDA (*E*_h/2_ = 0.62 V *vs*. SCE) could be oxidized by **Pc1** or the anode (Supplementary Fig. 5–16). All of these results suggest that alkyl radicals may be generated from the reduction in alkyl bromide by nickel intermediates. Therefore, a Ni^0^-Ni^II^- Ni^I^- Ni^II^- Ni^III^- Ni^I^ mechanism involving SET events and possible late-stage *Z/E* isomerization via energy transfer or direct excitation of the product (for photo-involved reactions) is proposed (Supplementary Fig. 22).

## Discussion

In conclusion, we developed a three-component, reductive cascade cross-coupling reaction for the synthesis of *E*- and *Z*-trisubstituted alkenes via the one-pot arylalkylation of alkynes. We also report the stereoselective alkyne functionalization using electrochemistry. The stereoselectivity is well-controlled by simply switching between electrochemical and photocatalyzed approaches or by use of a photo-assisted electrochemical reaction. The results also demonstrate that electrochemistry and photocatalysis can be mutually complementary and provide an efficient strategy for the stereodivergent synthesis of valuable organic compounds.

## Methods

### General procedure for the electrochemical reactions

A dry 5-mL vial equipped with a Teflon-coated magnetic stir bar (10 mm*3 mm) was charged with aryl halide (0.2 mmol, 1 equiv., if solid), alkyl halide (0.6 mmol, 3 equiv., if solid), alkyne (0.4 mmol, 2 equiv., if solid), NiBr_2_·d(4-OMe)-bpy (8.7 mg, 0.02 mmol, 10 mol%), and ^*n*^Bu_4_NBr (129 mg, 0.4 mmol, 2 equiv.) in glovebox. Anhydrous and degassed DMA (4.0 mL), aryl halide (0.2 mmol, 1 equiv., if liquid), alkyl halide (0.6 mmol, 3 equiv., if liquid), alkyne (0.4 mmol, 2 equiv., if liquid), and TMEDA (90 μL, 0.6 mmol, 3 equiv.) were added via syringe. Then, it was capped with a Teflon lid equipped with graphite electrode (20 × 7 × 2 mm) as the anode and nickel foam electrode (20 × 10 × 1 mm) as the cathode. The reaction mixture was stirred and electrolyzed at a constant current of 4 mA for 16 h. After the reaction is completed, the mixture was transferred to a 100 mL round bottom flask via syringe, electrodes were washed with ethyl acetate. The solution was diluted with H_2_O (30 mL), and extract by ethyl acetate (3 × 20 mL), and the combined organic layers were concentrated with a rotary evaporator. The product was purified by flash column chromatography on silica gel using hexane/EtOAc as eluent.

### General procedure for the photocatalyzed reactions

A dry reaction tube (10 mL) equipped with a Teflon-coated magnetic stir bar (6 mm*10 mm) was charged with aryl halide (0.4 mmol, 2 equiv., if solid), alkyl halide (0.6 mmol, 3 equiv., if solid), alkyne (0.2 mmol, 1 equiv., if solid), NiBr_2_·d(4-OMe)-bpy (8.7 mg, 0.02 mmol, 10 mol%), **Pc1** (2.3 mg, 0.002 mmol, 1 mol%), and LiCl (26 mg, 0.6 mmol, 3 equiv.) in glovebox. Anhydrous and degassed DMA (1.0 mL), aryl halide (0.4 mmol, 2 equiv., if liquid), alkyl halide (0.6 mmol, 3 equiv., if liquid), alkyne (0.2 mmol, 1 equiv., if liquid), and PMDTA (125 μL, 0.6 mmol, 3 equiv.) were added via syringe. The reaction mixture was stirred for 16 h under irradiation with a 45 W Kessil PR160-440 nm blue LED lamp with 50 W fan cooling. After the reaction is completed, the mixture was transferred to a 100 mL round bottom flask via syringe. The solution was diluted with H_2_O (30 mL), and extract by ethyl acetate (3 × 20 mL), and the combined organic layers were concentrated with a rotary evaporator. The product was purified by flash column chromatography on silica gel using hexane/EtOAc as eluent.

### General procedure for the photo-assisted electrochemical reactions

A dry 5-mL vial equipped with a Teflon-coated magnetic stir bar (10 mm*3 mm) was charged with aryl halide (0.2 mmol, 1 equiv., if solid), alkyl halide (0.6 mmol, 3 equiv., if solid), alkyne (0.4 mmol, 2 equiv., if solid), NiBr_2_·d(4-OMe)-bpy (8.7 mg, 0.02 mmol, 10 mol%), and ^*n*^Bu_4_NBr (65 mg, 0.2 mmol, 1 equiv.) in glovebox. Anhydrous and degassed DMA (4.0 mL), aryl halide (0.2 mmol, 1 equiv., if liquid), alkyl halide (0.6 mmol, 3 equiv., if liquid), alkyne (0.4 mmol, 2 equiv., if liquid), and TMEDA (90 μL, 0.6 mmol, 3 equiv.) were added via syringe. Then, it was capped with a Teflon lid equipped with graphite electrode (20 × 7 × 2 mm) as the anode and nickel foam electrode (20 × 10 × 1 mm) as the cathode. The reaction mixture was stirred, electrolyzed at a constant current of 6 mA under irradiation with two 52 W Kessil PR160-390 nm purple LED lamp with 50 W fan cooling for 48 h. After the reaction is completed, the mixture was transferred to a 100 mL round bottom flask via syringe, electrodes were washed with ethyl acetate. The solution was diluted with H_2_O (30 mL), and extract by ethyl acetate (3 × 20 mL), and the combined organic layers were concentrated with a rotary evaporator. The product was purified by flash column chromatography on silica gel using hexane/EtOAc as eluent.

## Supplementary information

Supplementary Information

## Data Availability

The authors declare that all data generated in this study are available within the article and the Supplementary Information.
