# Supplementary material for: Nickel catalyzed multicomponent stereodivergent synthesis of olefins enabled by electrochemistry, photocatalysis and photo-electrochemistry
Source: Nat Commun. 2022 Jun 10;13:3240. doi: 10.1038/s41467-022-30985-2 (PMC9187637; doi:10.1038/s41467-022-30985-2)
Supplement: Supplementary file 1 — Supplementary Information [file 41467_2022_30985_MOESM1_ESM.pdf]

Supplementary Information

Nickel Catalyzed Multicomponent Stereodivergent Synthesis of Olefins enabled by Electrochemistry, Photocatalysis and Photo-electrochemistry

Chen Zhu, Huifeng Yue, & Magnus Rueping**

KAUST Catalysis Center (KCC), King Abdullah University of Science and Technology (KAUST)
E-mail: huifeng.yue@kaust.edu.sa; magnus.rueping@kaust.edu.sa

Supplementary Methods

1. **General Information**
2. **Reaction Optimization Details**
3. **General Procedure for the Catalytic Reactions**
4. **Gram-scale Reaction**
5. ***E/Z* Isomerization Control Reaction**
6. **UV/Vis Absorption and Fluorescence spectroscopy of *E* and *Z* isomers**
7. **Steady-State Stern-Volmer Quenching Experiments**
8. **Cyclic Voltammetry Measurements**
9. **Reaction Progress Monitoring**
10. **Radical Trap Reactions**
11. **Proposed Mechanism**
12. **Spectroscopic Data of the Products**
13. **Copies of NMR Spectra**

Supplementary References

Supplementary Methods

1. General Information

Unless otherwise noted, all commercially available compounds were used as provided without further purification. Solvents for chromatography were HPLC grade. Anhydrous and degassed DMA (99.8%) used in reactions was purchased from Sigma-Aldrich in Sure/Seal™ bottle. Analytical thin-layer chromatography (TLC) was performed on Merck silica gel aluminium plates with F-254 indicator, visualized by irradiation with UV light. Column chromatography was performed on silica gel (particle size 0.043–0.063 mm) by using Interchim PuriFlash®430 automatic purification system. ^{1}H-NMR and ^{13}C-NMR were recorded on Bruker DRX-500 and AMX-400 instruments in $CDCl_3$ and are reported relative to the solvent residual peaks. Data are reported in the following order: chemical shift (δ) in ppm; multiplicities are indicated s (singlet), bs (broad singlet), d (doublet), t (triplet), m (multiplet); coupling constants (J) are in Hertz (Hz). Mass spectra (EI-MS, 70 eV) were conducted on a Agilent 7890 gas chromatograph equipped with 5975C EI-MSD Triple-Axis Detector using DB5MS and HP5MS columns. HRMS analysis was performed using a Thermo LTQ Velos Orbitrap mass spectrometer (Thermo Scientific, Pittsburgh, PA, USA) equipped with an ESI source. UV/Vis spectra were recorded using Agilent Cary 60 UV-Vis. Luminescence intensities were recorded using a fluoromax-4 spectrophotometer from Horiba Scientific. Cyclic voltammetry (CV) were performed on BioLogic Potentiostat SP-50. For the electrochemical reactions at constant current modes, Matsusada R4K36-0.1-L(230V) was used as power supply. For the photoredox catalyzed reactions, Kessil PR160-440nm blue LED lamp was used as light source. For the photo-assisted electrochemical reactions, Matsusada R4K36-0.1-L(230V) was used as power supply, Kessil PR160-390nm purple LED lamp was used as light source.

2. Reaction Optimization Details

2.1 Electrochemical reaction optimization

Supplementary Table 1: Initial Optimization

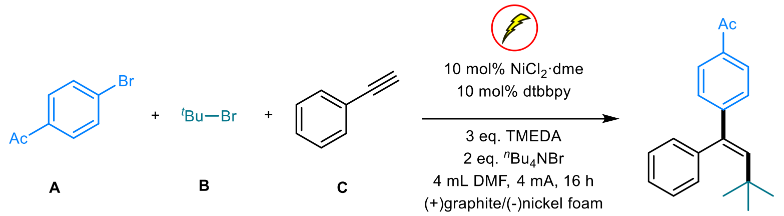

Entry	A	B	C	Variables	GC-Yield (%)	*Z:E*
1	1	2	3	(+)Stainless steel/(-)nickel foam, no TMEDA	0	--
2	1	2	3	--	23	pure E
3	1	2	3	Et_3N as donor	11	pure E
4	2	3	1	--	17	pure E
5	1	2	3	DMA as solvent	29	pure E
6	1	2	3	DMSO as solvent	7	pure E
7	1	2	3	CH_3CN as solvent	2	pure E
8	1	2	3	**D1** as donor, DMA as solvent	0	--
9	1	2	3	PMDTA as donor, DMA as solvent	40	pure E
10	1	2	3	**D2** as donor, DMA as solvent	29	pure E
11	1	2	3	quinuclidine as donor, DMA as solvent	7	pure E
12	2	1	3	DMA as solvent	17	pure E
13	1	3	2	DMA as solvent	52	pure E
14	1	3	2	PMDTA as donor, DMA as solvent	37	pure E
15	1	3	2	**D3** as donor, DMA as solvent	3	pure E
16	1	3	2	DABCO as donor, DMA as solvent	37	pure E
17	1	3	2	BTMG as donor, DMA as solvent	0	--

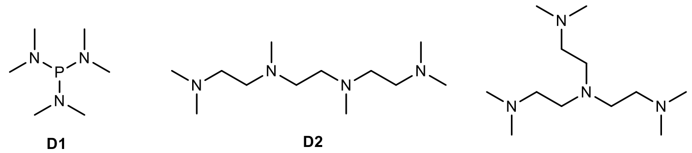

D1 **D2** **D3**

Supplementary Table 2: Catalysts and Ligands screening:

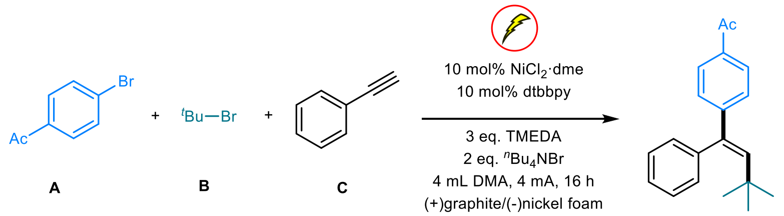

Entry	A	B	C	Variables	GC-Yield (%)	*Z:E*
1	1	3	2	**L2** as ligand	28	pure E
2	1	3	2	**L3** as ligand	16	pure E
3	1	3	2	**L4** as ligand	3	pure E
4	1	3	2	**L5** as ligand	64	pure E
5	1	3	2	**L6** as ligand	4	pure E
6	1	3	2	**L7** as ligand	28	pure E
7	1	3	2	**L8** as ligand	3	pure E
8	1	3	2	**L9** as ligand	17	pure E
9	1	3	2	**L10** as ligand	63	pure E
10	1	3	2	**L11** as ligand	17	pure E
11	1	3	2	**L12** as ligand	37	pure E
12	1	3	2	**L13** as ligand	22	pure E
13	1	3	2	**L14** as ligand	63	pure E
14	1	3	2	**L15** as ligand	35	pure E
15	1	3	2	**L16** as ligand	36	pure E
16	1	3	2	**L17** as ligand	0	--
17	1	3	2	**L18** as ligand	8	pure E
18	1	3	2	$NiBr_2$·dme as catalyst	58	pure E
19	1	3	2	$NiBr_2$·dtbbpy as catalyst	71	pure E
20	1	3	2	$NiBr_2$·bpy as catalyst	51	pure E
21	1	3	2	$NiBr_2$·d(4-OMe)-bpy as catalyst	76	pure E
22	1	3	2	$NiCl_2·6H_2O$ as catalyst	54	pure E
23	1	3	2	$NiBr_2·3H_2O$ as catalyst	69	pure E

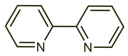
L2
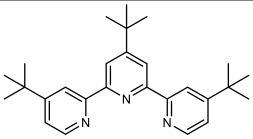
L3
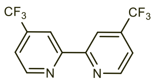

L4
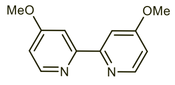

L5
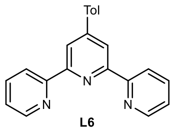

L6
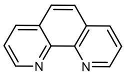
L7
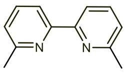
L8
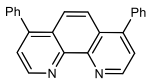

L9
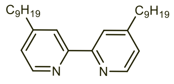

L10
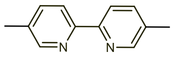
L11
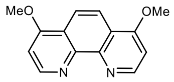

L12
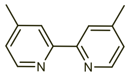
L13
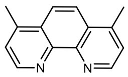
L14
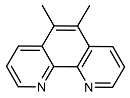
L15
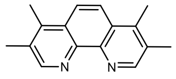
L16
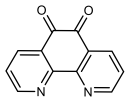
L17
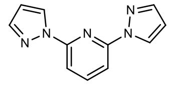
L18

Supplementary Table 3: Electrolytes screening

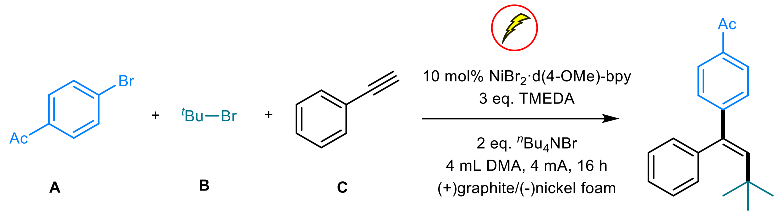

Entry	A	B	C	Variables	GC-Yield (%)	*Z:E*
1	1	3	2	NEt_4Br as electrolyte	68	pure E
2	1	3	2	NEt_4BF_4 as electrolyte	65	pure E
3	1	3	2	$^nBu_4NPF_6$ as electrolyte	68	pure E
4	1	3	2	TBAI as electrolyte	14	pure E
5	1	3	2	KBr as electrolyte	69	pure E
6	1	3	2	4 eq. $^nBu_4NBr$ as electrolyte	64	pure E

Supplementary Table 4: Others screening

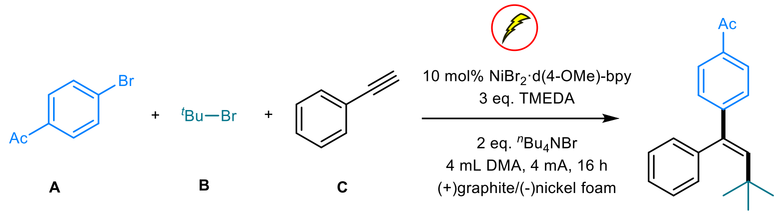

Entry	A	B	C	Variables	GC-Yield (%)	*Z:E*
1	1	3	2	2 eq. TMEDA	53	pure E
2	1	3	2	2 mA current	45	pure E
3	1	3	2	6 mA current	61	pure E
4	1	3	2	8 mA current	47	pure E
5	1	3	2	5 eq. TMEDA	70	pure E
6	1	3	2	Cu-foam as cathode	55	pure E
7	1	3	2	Pt as cathode	37	pure E
8	1	3	2	Pt as anode	69	pure E

Supplementary Table 5: Control Reactions

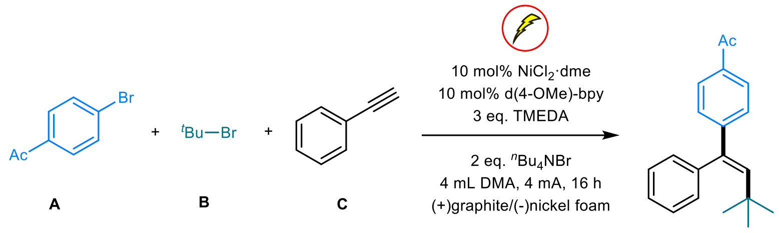

Entry	A	B	C	Variables	GC-Yield (%)	*Z:E*
1	1	3	2	no nickel	0	--
2	1	3	2	no ligand	0	--
3	1	3	2	no TMEDA	0	--
4	1	3	2	no ^{n}Bu$_4$NBr	62	pure E
5	1	3	2	no electricity	0	--

2.2 Photoredox-catalyzed reaction optimization

Supplementary Table 6: Initial Optimization

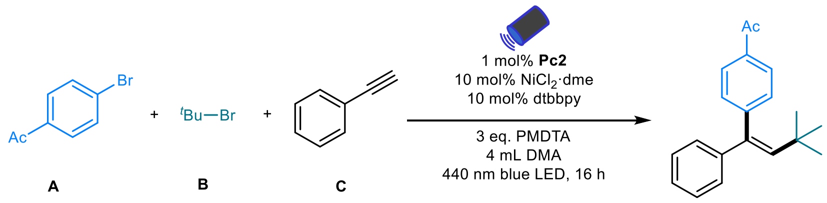

Entry	A	B	C	Variables	GC-Yield (%)	*Z:E*
1	1	3	2	--	25	94:6
2	1	3	2	**Pc3** as photocatalyst	50	84:16
3	2	3	1	--	57	87:13
4	1	3	2	3 eq. ^{n}Bu$_4$NBr as additive	49	94:6
5	1	3	2	3 eq. LiCl as additive	57	94:6
6	2	3	1	3 eq. LiCl as additive	65	94:6
7	2	3	1	**Pc3** as photocatalyst, 3 eq. LiCl as additive	69	90:10
8	2	3	1	**Pc4** as photocatalyst, 3 eq. LiCl as additive	62	77:23
9	2	3	1	TMEDA as donor, 3 eq. LiCl as additive	33	84:16
10	3	1	2	3 eq. LiCl as additive	61	82:18

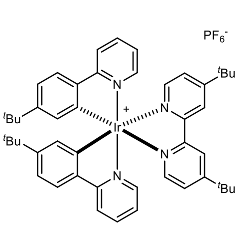

Pc2

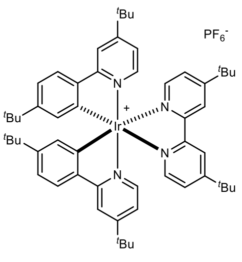

Pc3

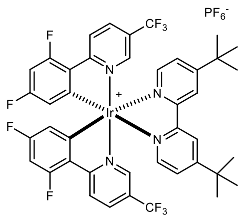

Pc4

Supplementary Table 7: Photocatalysts Screening

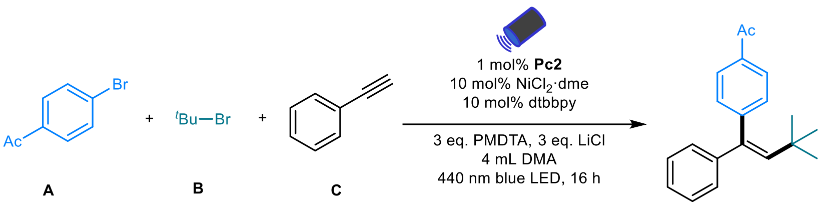

Entry	A	B	C	Variables	GC-Yield (%)	*Z:E*
1	2	3	1	5 mol% 4CzIPN	30	20:80
2	2	3	1	**Pc5** as photocatalyst	70	92:8
3	2	3	1	**Pc6** as photocatalyst	54	90:10
4	2	3	1	**Pc7** as photocatalyst	75	86:14
5	2	3	1	**Pc8** as photocatalyst	84	85:15
6	2	3	1	**Pc9** as photocatalyst	12	33:67
7	2	3	1	**Pc10** as photocatalyst	9	36:64
8	2	3	1	**Pc11** as photocatalyst	8	85:15
9	2	3	1	**Pc12** as photocatalyst	66	92:8
10	2	3	1	**Pc13** as photocatalyst	0	--
11	2	3	1	**Pc14** as photocatalyst	35	87:13
12	2	3	1	**Pc15** as photocatalyst	43	91:9
13	2	3	1	**Pc16** as photocatalyst	0	--
14	2	3	1	**Pc17** as photocatalyst	0	--
15	2	3	1	**Ru(bpy)3** as photocatalyst	1	pure E
16	2	3	1	**Pc18** as photocatalyst	2	pure E
17	2	3	1	**Eosin Y** as photocatalyst	3	pure E

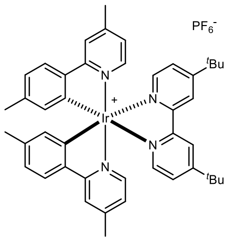

Pc5

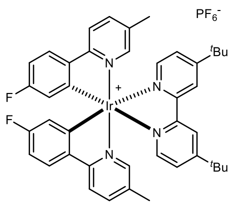

Pc6

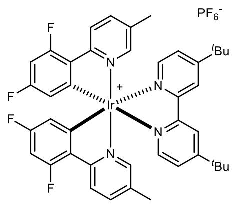

Pc7

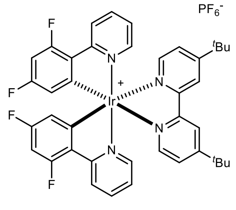

Pc8

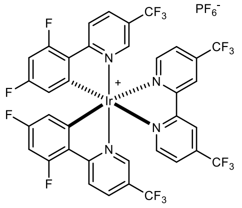

Pc9

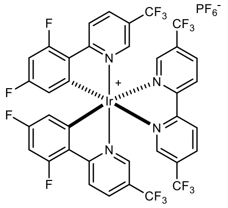

Pc10

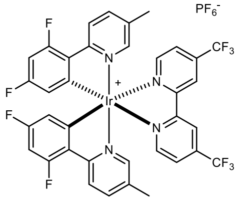

Pc11

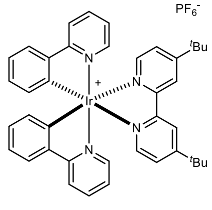

Pc12

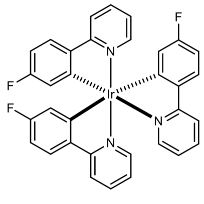

Pc13

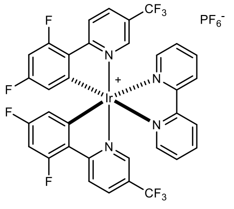

Pc14

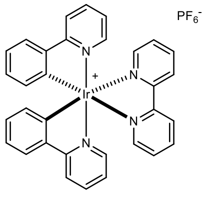

Pc15

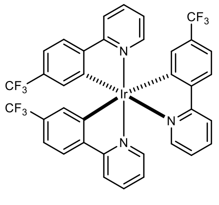

Pc16

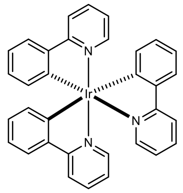

Pc17

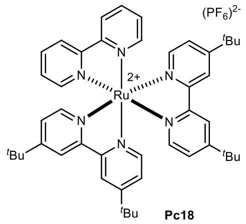

Pc18

Supplementary Table 8: Ligand Screening

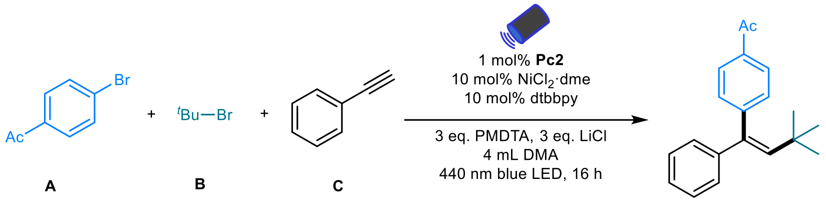

Entry	A	B	C	Variables	GC-Yield (%)	*Z:E*
1	2	3	1	**L2** as ligand	63	93:7
2	2	3	1	**L5** as ligand	73	94:6
3	2	3	1	**L10** as ligand	65	93:7
4	2	3	1	**L12** as ligand	15	94:6
5	2	3	1	**L13** as ligand	35	94:6

6	2	3	1	**L14** as ligand	45	93:7

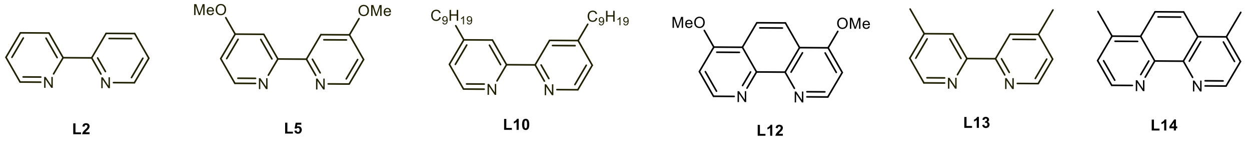

Supplementary Table 9: Others Screening

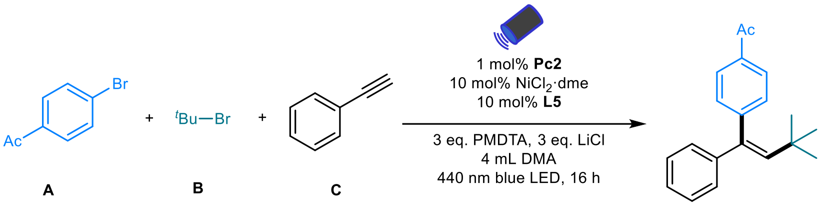

Entry	A	B	C	Variables	GC-Yield (%)	*Z:E*
1	2	3	1	$NiBr_2$·dtbbpy as catalyst	71	93:7
2	2	3	1	$NiBr_2$·bpy as catalyst	69	93:7
3	2	3	1	$NiBr_2$·d(4-OMe)-bpy as catalyst	75	94:6
4	2	3	1	$NiBr_2$·d(4-OMe)-bpy as catalyst, 3 eq. NEt_4Br as additive	66	88:12
5	2	3	1	$NiBr_2$·d(4-OMe)-bpy as catalyst, 3 eq. NaI as additive	30	80:20
6	2	3	1	$NiBr_2$·d(4-OMe)-bpy as catalyst, 3 eq. TBAI as additive	69	93:7
7	2	3	1	$NiBr_2$·d(4-OMe)-bpy as catalyst, 3 eq. LiBr as additive	69	93:7
8	2	3	1	**Pc1** as photocatalyst, $NiBr_2$·d(4-OMe)-bpy as catalyst	79	95:5

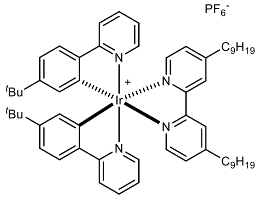

Pc1

Supplementary Table 10: Control Reactions

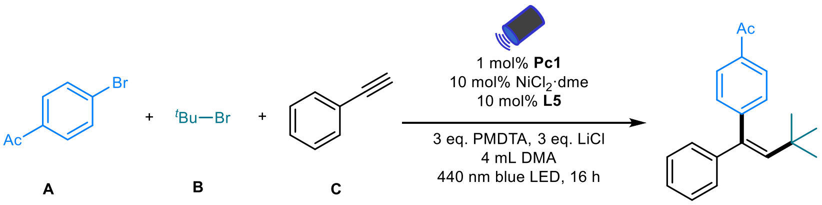

Entry	A	B	C	Variables	GC-Yield (%)	*Z:E*
1	1	3	2	no photocatalyst	0	--
2	1	3	2	no nickel	0	--
3	1	3	2	no ligand	3	88:12
4	1	3	2	no PMDTA	0	--
5	1	3	2	no LiCl	70	92:8
6	1	3	2	no light	0	--

2.2 Photo-assisted electrochemical reaction optimization

Supplementary Table 11: Reaction Optimization

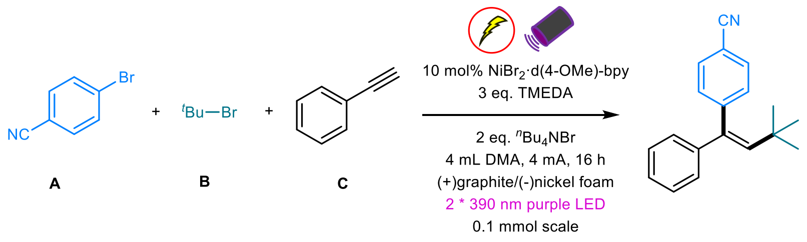

Entry	A	B	C	Variables	GC-Yield (%)	*Z:E*
1	1	3	2	--	58	92:8
2	1	3	2	5 mol% $NiBr_2$·d(4-OMe)-bpy	26	94:6
3	1	3	2	20 mol% $NiBr_2$·d(4-OMe)-bpy	68	84:16
4	1	3	2	3 mL solvent	63	91:9
5	1	3	2	2 mA	29	81:19
6	1	3	2	6 mA	69	94:6
7	1	3	2	4 eq. $^nBu_4NBr$	53	93:7
8	1	3	2	8 mA	48	94:6
9	1	3	2	1 eq. $^nBu_4NBr$	31	94:6
10	1	3	2	0.2 mmol scale, 6 mA, 24 h	60	93:7
11	1	3	2	0.2 mmol scale, 6 mA, 1 eq. $^nBu_4NBr$, 24 h	68	93:7

3. General Procedure for the Catalytic Reactions

3.1 General Procedure A (electrochemical reaction)

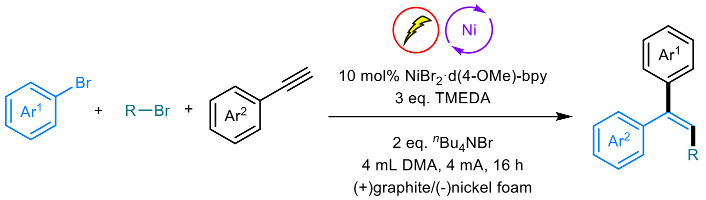

A dry 5-mL vial equipped with a Teflon-coated magnetic stir bar (10mm*3mm) was charged with aryl halide (0.2 mmol, 1 equiv., if solid), alkyl halide (0.6 mmol, 3 equiv., if solid), alkyne (0.4 mmol, 2 equiv., if solid), $NiBr_2$·d(4-OMe)-bpy (8.7 mg, 0.02 mmol, 10 mol%), and $^{n}Bu_4NBr$ (129 mg, 0.4 mmol, 2 equiv.) in glovebox. Anhydrous and degassed DMA (4.0 mL), aryl halide (0.2 mmol, 1 equiv., if liquid), alkyl halide (0.6 mmol, 3 equiv., if liquid), alkyne (0.4 mmol, 2 equiv., if liquid), and TMEDA (90 µL, 0.6 mmol, 3 equiv.) were added via syringe. Then, it was capped with a Teflon lid equipped with graphite electrode (20×7×2 mm) as the anode and nickel foam electrode (20×10×1 mm) as the cathode. The reaction mixture was stirred and electrolyzed at a constant current of 4 mA for 16 h. After the reaction is completed, the mixture was transferred to a 100 mL round bottom flask via syringe, electrodes were washed with ethyl acetate. The solution was diluted with H_2O (30 mL), and extract by ethyl acetate (3×20 mL), and the combined organic layers were concentrated with a rotary evaporator. The product was purified by flash column chromatography on silica gel using hexane/EtOAc as eluent.

3.2 General Procedure B (photocatalysis)

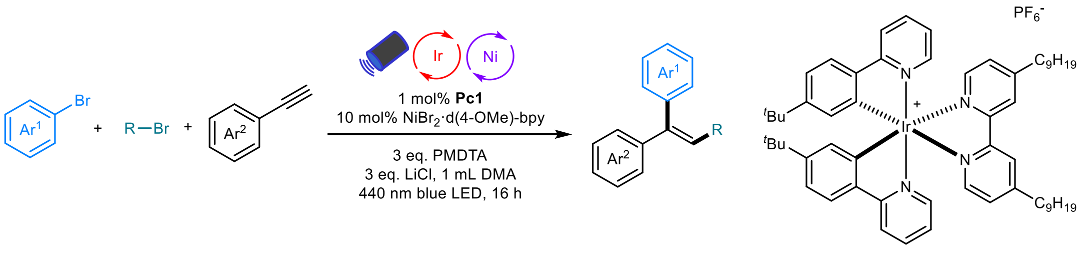

Pc1

A dry reaction tube (10 mL) equipped with a Teflon-coated magnetic stir bar (6mm*10mm) was charged with aryl halide (0.4 mmol, 2 equiv., if solid), alkyl halide (0.6 mmol, 3 equiv., if solid), alkyne (0.2 mmol, 1 equiv., if solid), $NiBr_2$·d(4-OMe)-bpy (8.7 mg, 0.02 mmol, 10 mol%), **Pc1** (2.3 mg, 0.002 mmol, 1 mol%), and LiCl (26 mg, 0.6 mmol, 3 equiv.) in glovebox. Anhydrous and degassed DMA (1.0 mL), aryl halide (0.4 mmol, 2 equiv., if liquid), alkyl halide (0.6 mmol, 3 equiv., if liquid), alkyne (0.2 mmol, 1 equiv., if liquid), and PMDTA (125 µL, 0.6 mmol, 3 equiv.) were added via syringe. The reaction mixture was stirred for 16 h under irradiation with a 45 W Kessil PR160-440 nm blue LED lamp with 50 W fan cooling. After the reaction is completed, the mixture was transferred to a 100 mL round bottom flask via syringe. The solution was diluted with H_2O (30 mL), and extract by ethyl acetate (3×20 mL), and the combined organic

layers were concentrated with a rotary evaporator. The product was purified by flash column chromatography on silica gel using hexane/EtOAc as eluent.

3.3 General Procedure C (photo-assisted electrochemical reaction)

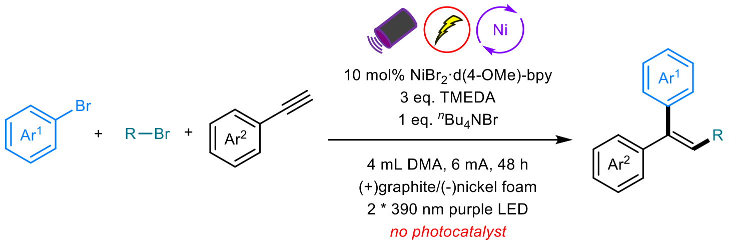

A dry 5-mL vial equipped with a Teflon-coated magnetic stir bar (10mm*3mm) was charged with aryl halide (0.2 mmol, 1 equiv., if solid), alkyl halide (0.6 mmol, 3 equiv., if solid), alkyne (0.4 mmol, 2 equiv., if solid), $NiBr_2$·d(4-OMe)-bpy (8.7 mg, 0.02 mmol, 10 mol%), and $^{n}Bu_4NBr$ (65 mg, 0.2 mmol, 1 equiv.) in glovebox. Anhydrous and degassed DMA (4.0 mL), aryl halide (0.2 mmol, 1 equiv., if liquid), alkyl halide (0.6 mmol, 3 equiv., if liquid), alkyne (0.4 mmol, 2 equiv., if liquid), and TMEDA (90 µL, 0.6 mmol, 3 equiv.) were added via syringe. Then, it was capped with a Teflon lid equipped with graphite electrode (20×7×2 mm) as the anode and nickel foam electrode (20×10×1 mm) as the cathode. The reaction mixture was stirred, electrolyzed at a constant current of 6 mA under irradiation with two 52 W Kessil PR160-390 nm purple LED lamp with 50 W fan cooling for 48 h. After the reaction is completed, the mixture was transferred to a 100 mL round bottom flask via syringe, electrodes were washed with ethyl acetate. The solution was diluted with H_2O (30 mL), and extract by ethyl acetate (3×20 mL), and the combined organic layers were concentrated with a rotary evaporator. The product was purified by flash column chromatography on silica gel using hexane/EtOAc as eluent.

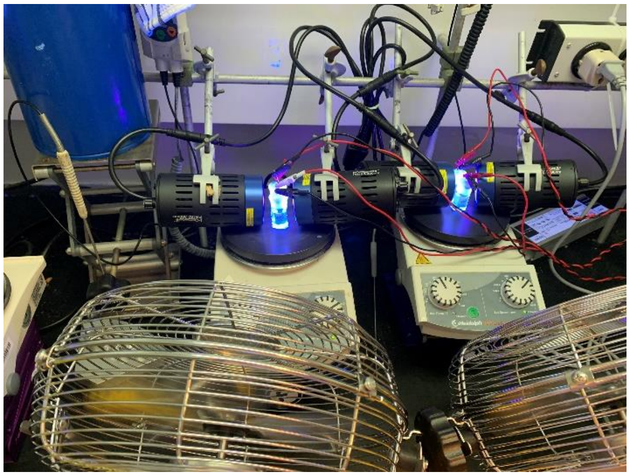

Supplementary Figure 1: Photo-assisted electrochemical reaction setup

3.4 General Procedure D (photo-assisted electrochemical reaction)

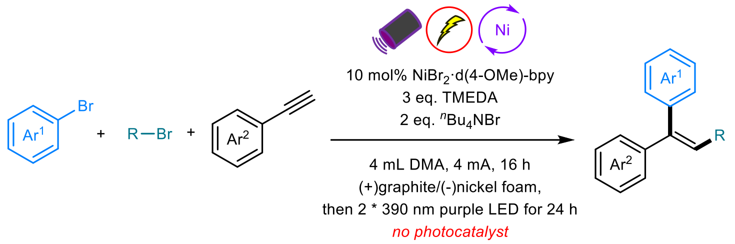

A dry 5-mL vial equipped with a Teflon-coated magnetic stir bar (10mm*3mm) was charged with aryl halide (0.2 mmol, 1 equiv., if solid), alkyl halide (0.6 mmol, 3 equiv., if solid), alkyne (0.4 mmol, 2 equiv., if solid), $NiBr_2$·d(4-OMe)-bpy (8.7 mg, 0.02 mmol, 10 mol%), and $^nBu_4NBr$ (129 mg, 0.4 mmol, 1 equiv.) in glovebox. Anhydrous and degassed DMA (4.0 mL), aryl halide (0.2 mmol, 1 equiv., if liquid), alkyl halide (0.6 mmol, 3 equiv., if liquid), alkyne (0.4 mmol, 2 equiv., if liquid), and TMEDA (90 µL, 0.6 mmol, 3 equiv.) were added via syringe. Then, it was capped with a Teflon lid equipped with graphite electrode (20×7×2 mm) as the anode and nickel foam electrode (20×10×1 mm) as the cathode. The reaction mixture was stirred and electrolyzed at a constant current of 4 mA. After 16 h, the power supply was switched off and the vial was directly irradiated with two 52 W Kessil PR160-390 nm purple LED lamp with 50 W fan cooling for 24 h. After the reaction is completed, the mixture was transferred to a 100 mL round bottom flask via syringe, electrodes were washed with ethyl acetate. The solution was diluted with H_2O (30 mL), and extract by ethyl acetate (3×20 mL), and the combined organic layers were concentrated with a rotary evaporator. The product was purified by flash column chromatography on silica gel using hexane/EtOAc as eluent.

4. Gram-scale Reaction

4.1 Electrochemical Reaction

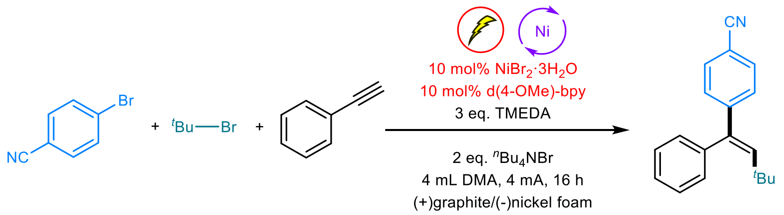

A dry 250-mL beaker equipped with a Teflon-coated magnetic stir bar was charged with 4-bromobenzonitrile (20 mmol, 1 equiv., 3.64 g), $NiBr_2·3H_2O$ (545 mg, 2 mmol, 10 mol%), d(4-OMe)-bpy (432 mg, 2 mmol, 10 mol%), and $^nBu_4NBr$ (13 g, 40 mmol, 2 equiv.) in glovebox. Anhydrous and degassed DMA (150 mL), 2-bromo-2-methylpropane (60 mmol, 3 equiv., 6.7 mL), ethynylbenzene (40 mmol, 2

equiv., 4.4 mL), and TMEDA (8.9 mL, 60 mmol, 3 equiv.) were added via syringe. Then, it was capped with a Teflon lid equipped with graphite electrode (70×60×2 mm) as the anode and nickel foam electrode (55×40×2 mm) as the cathode. The reaction mixture was stirred and electrolyzed at a constant current of 100 mA for 16 h. After the reaction is completed, the mixture was transferred to a 1 L round bottom flask, electrodes were washed with ethyl acetate. The solution was diluted with H_2O (200 mL), and extract by ethyl acetate (3×200 mL), and the combined organic layers were concentrated with a rotary evaporator. The title compound was isolated in 70% (3.66 g) yield after flash chromatography on silica gel.

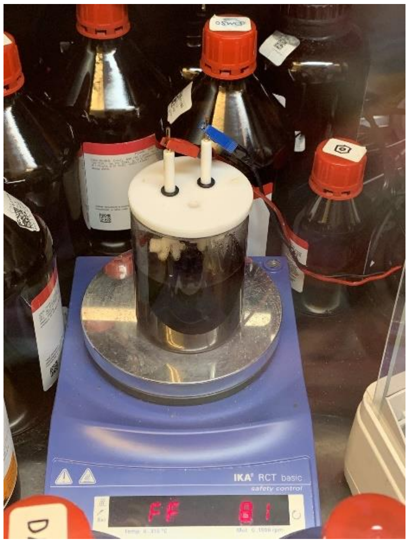

Supplementary Figure 2: Electrochemical gram-scale reaction setup

4.2 Photocatalysis

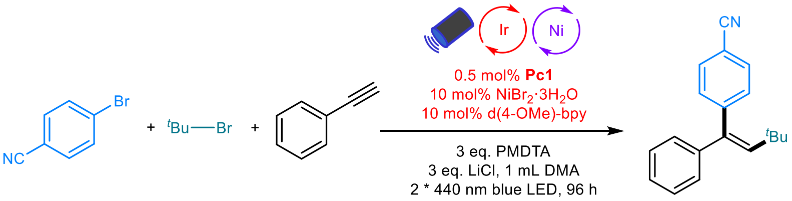

A dry flask (100 mL) equipped with a Teflon-coated magnetic stir bar was charged with 4-bromobenzonitrile (20 mmol, 2 equiv., 3.64 g), $NiBr_2 \cdot 3H_2O$ (273 mg, 1 mmol, 10 mol%), d(4-OMe)-bpy (216 mg, 1 mmol, 10 mol%), **Pc1** (58.3 mg, 0.05 mmol%, 0.5 mol%), and LiCl (1.27 g, 30 mmol, 3 equiv.) in glovebox. Anhydrous and degassed DMA (50 mL), 2-bromo-2-methylpropane (30 mmol, 3 equiv., 3.37 mL), ethynylbenzene (10 mmol, 1 equiv., 1.10 mL), and PMDTA (6.26 mL, 30 mmol, 3 equiv.) were added via syringe. The reaction mixture was stirred for 96 h under irradiation with two 45 W Kessil PR160-440 nm blue LED lamp with 50 W fan cooling. After the reaction is completed, the mixture was transferred to

a 1 L round bottom flask via syringe. The solution was diluted with H_2O (200 mL), and extract by ethyl acetate (3×200 mL), and the combined organic layers were concentrated with a rotary evaporator. The title compound was isolated in 72% (1.89 g) yield with 93:7 *Z:E* ratio after flash chromatography on silica gel. The product can be further purified by recrystallization from DCM and hexane to give the pure *Z* isomer (41%, 1.08 g). The scale-up reaction monitoring was shown below. The cross-coupling reaction (SET process) was finished within 2h, while the *Z*:*E* isomersization (ET process) was finished in 96 h.

Table S12: Photocatalyzed gram-scale reaction monitoring

Time	GC-Yield (%)	*Z:E*
2 h	88	17:83
12 h	88	52:48
36 h	88	81:19
48 h	88	85:15
72 h	88	90:10
96 h	88	93:7

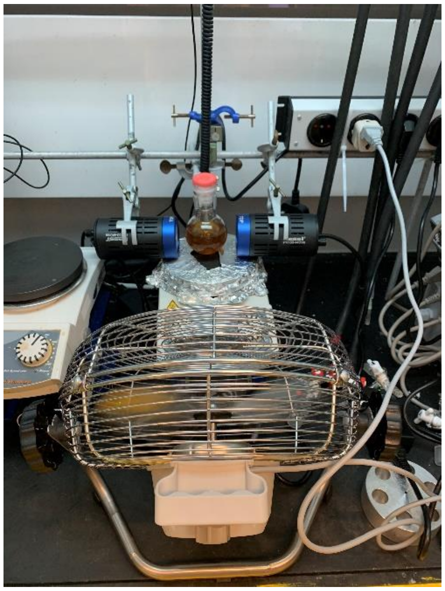

Supplementary Figure 3: Photocatalyzed gram-scale reaction setup

5. *E/Z* Isomerization Control Reaction

A dry reaction tube (10 mL) equipped with a Teflon-coated magnetic stir bar (6mm*10mm) was charged with pure (*E*)-4-(3,3-dimethyl-1-phenylbut-1-en-1-yl)benzonitrile or (*Z*)-4-(3,3-dimethyl-1-phenylbut-1-en-1-yl)benzonitrile (10 mg) in glovebox. Then, degassed DMA (1.0 mL) was added via syringe. The reaction mixture was stirred for 16 h under irradiation with Kessil lamp with 50 W fan cooling. Upon completion, the solution was passed through a pad of silica gel and washed with DCM and ethyl acetate. The *Z/E* ratio was determined via GC-analysis.

Table S13: Isomerization control reactions of *E* isomer

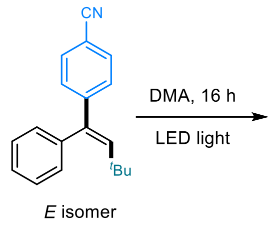

Conditions	*Z:E*
440 nm, with **Pc1**	94:6
440 nm	3:97
427 nm	7:93
390 nm	94:6
370 nm	92:8
90 W Kessil A360WE in DMA	18:82
90 W Kessil A360WE in DMSO	46:54

Table S14: Isomerization control reactions of *Z* isomer

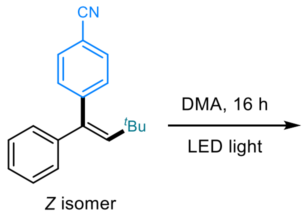

Conditions	*Z:E*
440 nm, with **Pc1**	94:6
440 nm	100:0
427 nm	99:1
390 nm	95:5
370 nm	96:4

6. UV/Vis Absorption and Fluorescence spectroscopy of *E* and *Z* isomers

The *E* and *Z* isomers solution were prepared in 2 mL quartz cuvettes (20 mM concentration, in DMA), equipped with PTFE stoppers, and sealed with parafilm inside glovebox. Pure DMA solvent was used as reference in the UV/Vis absorption spectroscopy. The excitation wavelength for fluorescence spectroscopy was 390 nm. The emission maximum for both *E* and *Z* isomers were 440 nm.

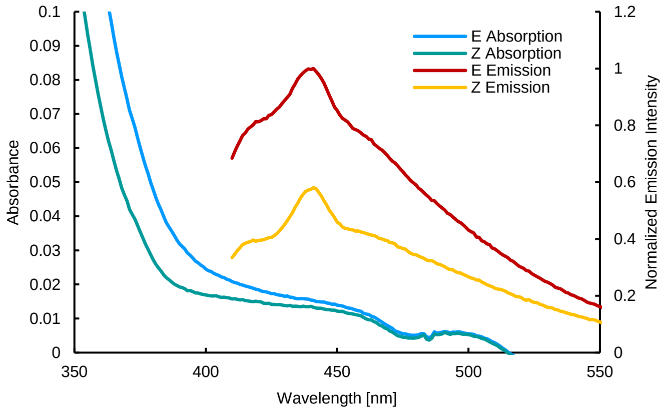

Supplementary Figure 4: UV/Vis absorption and fluorescence spectroscopy of *E* and *Z* isomers

7. Steady-State Stern-Volmer Quenching Experiments

Steady-state Stern-Volmer quenching experiments were carried out using a 0.01 mM solution of photocatalyst **Pc1** and variable concentrations (4, 8, 12, 16, 20, 30, 40 mM) of PMDTA, 4-bromobenzonitrile, 2-bromo-2-methylpropane, or ethynylbenzene in anhydrous DMA. The samples were prepared in 2 mL quartz cuvettes, equipped with PTFE stoppers, and sealed with parafilm inside argon filled glove-box. The intensity of the emission peak at 575 nm (λ_{ex} = 390 nm) for **Pc1** expressed as the ratio I_0/I, where I_0 is the emission intensity of **Pc1** at 575 nm in the absence of a quencher and I is the observed intensity, as a function of the quencher concentration was measured. Fluorescence emission spectra and Steady-state Stern-Volmer plots for each component are given in Figure below.

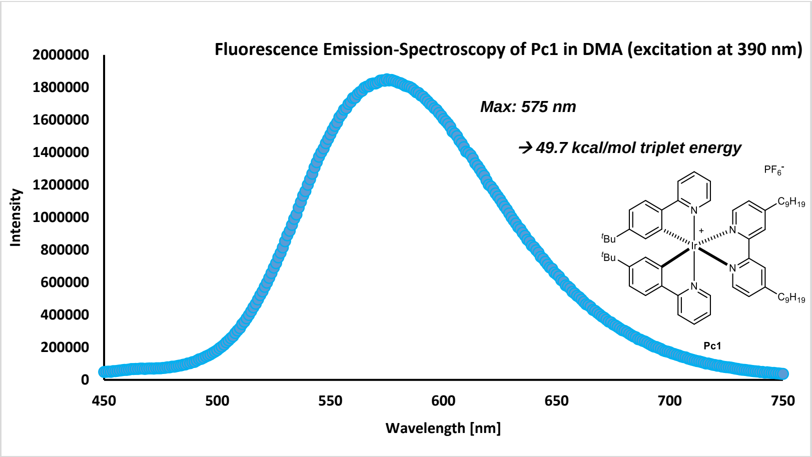

Supplementary Figure 5: Fluorescence spectroscopy of **Pc1** (0.01 mM) in DMA

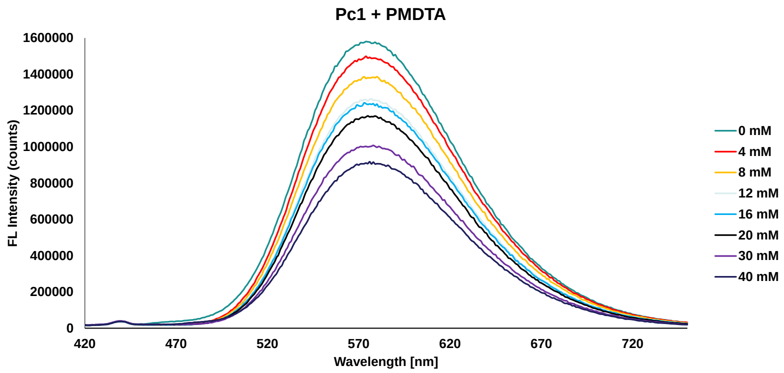

Supplementary Figure 6: Emission spectra of **Pc1** (0.01 mM) at different concentrations of PMDTA.

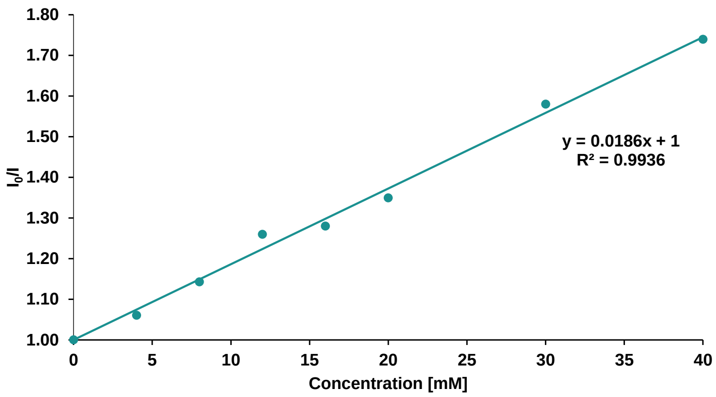

Supplementary Figure 7: Steady-state Stern-Volmer plot of **Pc1** (0.01 mM) at different concentrations of PMDTA.

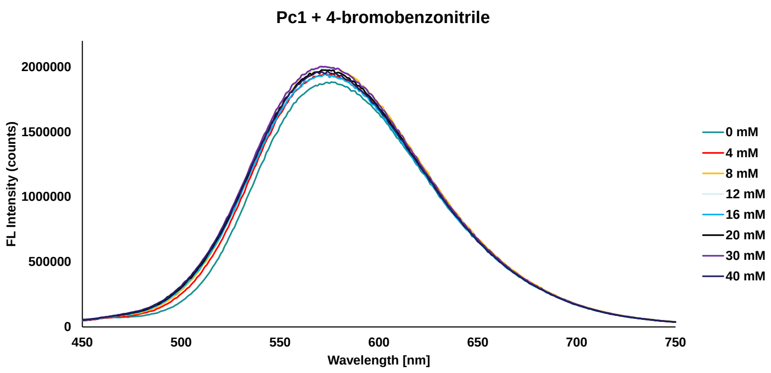

Supplementary Figure 8: Emission spectra of **Pc1** (0.01 mM) at different concentrations of 4-bromobenzonitrile.

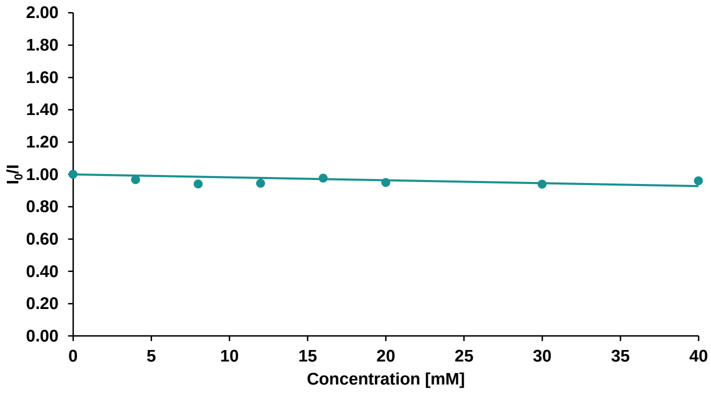

Supplementary Figure 9: Steady-state Stern-Volmer plot of **Pc1** (0.01 mM) at different concentrations of 4-bromobenzonitrile.

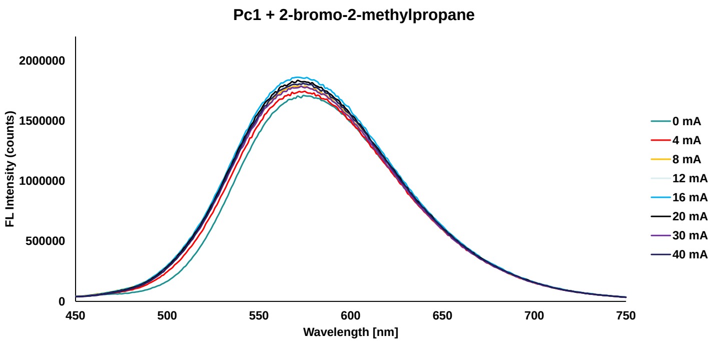

Supplementary Figure 10: Emission spectra of **Pc1** (0.01 mM) at different concentrations of 2-bromo-2-methylpropane.

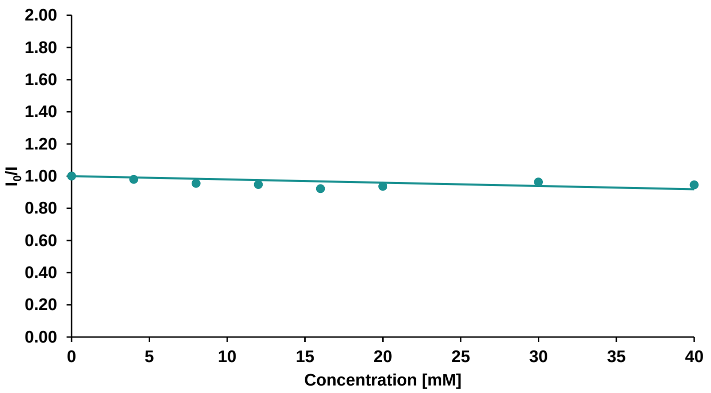

Supplementary Figure 11: Steady-state Stern-Volmer plot of **Pc1** (0.01 mM) at different concentrations of 2-bromo-2-methylpropane.

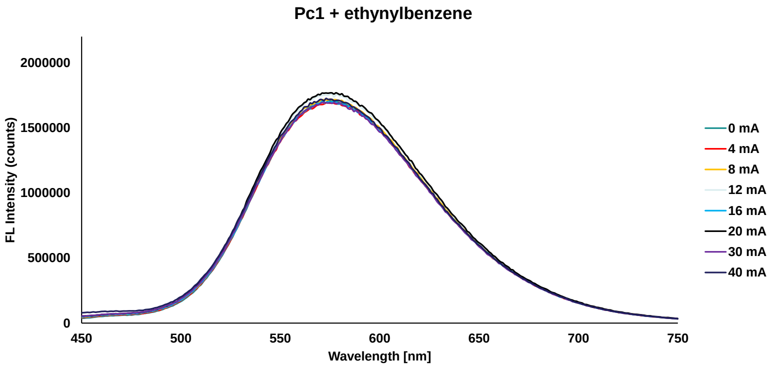

Supplementary Figure 12: Emission spectra of **Pc1** (0.01 mM) at different concentrations of ethynylbenzene

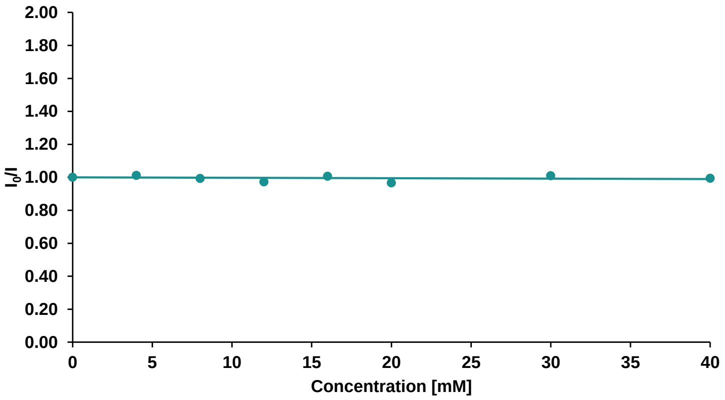

Supplementary Figure 13: Steady-state Stern-Volmer plot of **Pc1** (0.01 mM) at different concentrations

of ethynylbenzene.

8. Cyclic Voltammetry Measurements

All measurements were performed under anhydrous conditions in argon-filled glovebox. All supporting electrolytes were dried under dynamic vacuum (less than 0.1 mbar) over 24 h at 100 °C and stored inside the glovebox. The cell for the analysis was equipped with a glass vial (working volume is 10 mL) and Teflon cap, equipped with O-ring for tight sealing. Glassy carbon was used as working electrodes (circle, d = 3 mm), platinum wire as a counter electrode, and Ag/$AgNO_3$ (0.01 M in 0.1 M Bu_4NPF_6 in acetonitrile) as a reference. All measurements were conducted in 0.1 M solutions of Bu_4NPF_6 in CH_3CN. The scan rate was 50 mV/s. The potentials were given relative to the Fc/Fc^+ redox couple with ferrocene as internal standard. For conversion to SCE as reference, it is known that SCE is 400 mV more negative than Fc/Fc^+ in MeCN with NBu_4PF_6 as supporting electrolyte[1].

The concentration of photocatalyst **PC1** was 0.5 mM. The scan direction was positive. Excited state potentials are estimated using the Rehm-Weller equations as given[2]:

$$E_{Ox}^{o*} = E_{Ox}^{o'} - E^{0-0}$$

$$E_{Red}^{o*} = E_{Red}^{o'} + E^{0-0}$$

Where E^{o*} represents the excited state potential, $E^{o'}$ represents the ground state potential, and $E^{0\text{-}0}$ refers to the energy gap between the zeroeth level vibrational levels of the ground and excited state. E_{ox} refers to the Ir(III)/Ir(IV) couples and E_{red} to the Ir(II)/(III) couples. $E^{0\text{-}0}$ is approximated as the difference between the onset of oxidation and the onset of reduction[3].

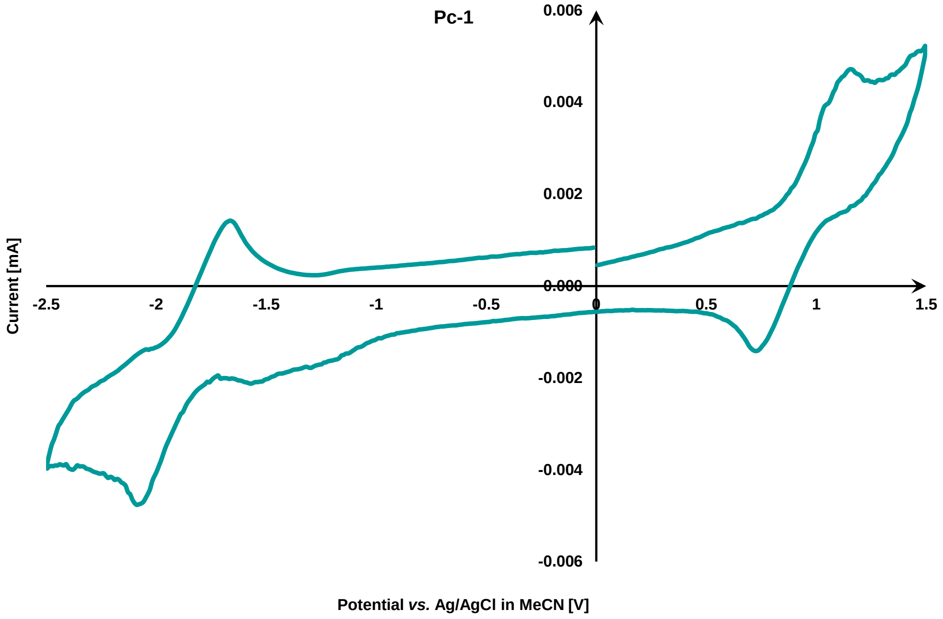

Supplementary Figure 14: Cyclic voltammetry scan of **Pc1** in MeCN.

The reversible peaks at 1.16 V and 0.72 V correspond to the Ir(III)/Ir(IV) couples. The reversible peaks at -2.09 V and -1.66 V correspond to the Ir(II)/(III) couples.

$E_{1/2}$(Fc/Fc$^+$) = +0.092 V

$E^{0\text{-}0}$ = 2.55 V

Ir(II)/Ir(III)

$E_{1/2}$ = -1.97 V v. Fc/Fc$^+$

$E_{1/2}$ = -1.57 V v. SCE

Ir(II)/Ir*(III)

$E_{1/2}$ = 0.58 V v. Fc/Fc$^+$

$E_{1/2}$ = 0.98 V v. SCE

Ir(III)/Ir(IV)

$E_{1/2}$ = 0.85 V v. Fc/Fc$^+$

$E_{1/2}$ = 1.25 V v. SCE

Ir* (III)/Ir(IV)

$E_{1/2}$ = -1.70 V v. Fc/Fc$^+$

$E_{1/2}$ = -1.30 V v. SCE

Cyclic voltammetry measurement of *N,N,N′,N′*-Tetramethylethylenediamine (TMEDA):

The concentration of TMEDA was 10 mM. The scan direction was positive.

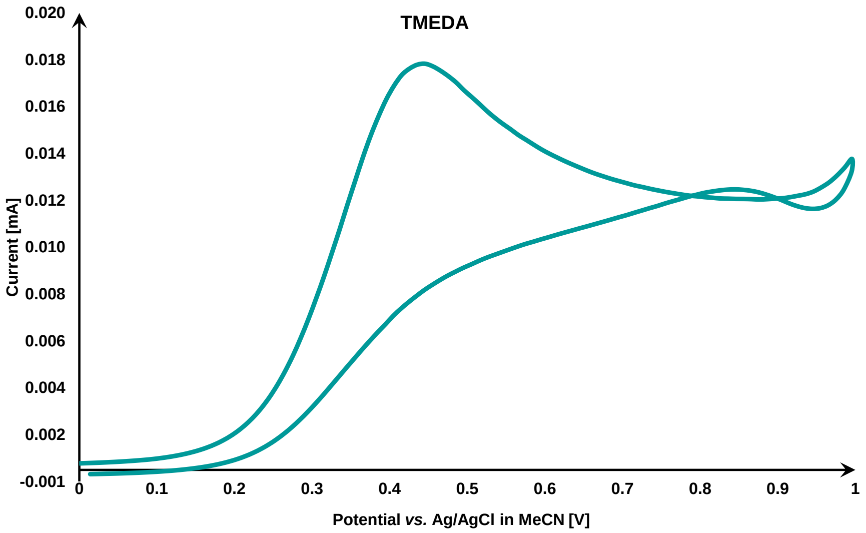

Supplementary Figure 15: Cyclic voltammetry scan of TMEDA in MeCN.

The irreversible peak at $E_{h/2}$ = 0.32 V correspond to the oxidation of TMEDA.

$E_{1/2}(Fc/Fc^{+})$ = +0.089 V

$E_{h/2}$ = 0.22 V v. Fc/Fc^{+}

$E_{h/2}$ = 0.62 V v. SCE

Cyclic voltammetry measurement of N,N,N′,N′′,N′′-Pentamethyldiethylenetriamine (PMDTA):

The concentration of PMDTA was 10 mM. The scan direction was positive.

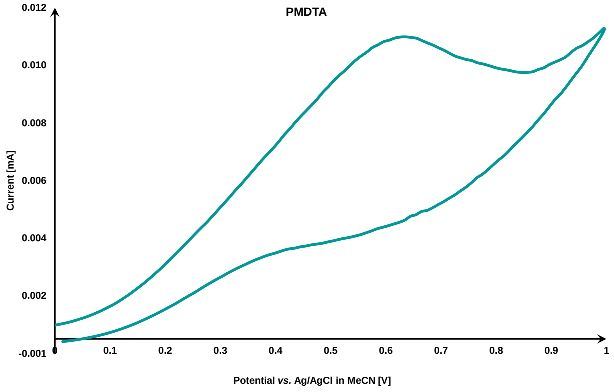

Supplementary Figure 16: Cyclic voltammetry scan of PMDTA in MeCN.

The irreversible peak at $E_{h/2}$ = 0.33 V correspond to the oxidation of TMEDA.

$E_{1/2}(Fc/Fc^+) = +0.089$ V

$E_{h/2} = 0.23$ V v. Fc/Fc^+

$E_{h/2} = 0.63$ V v. SCE

9. Reaction Progress Monitoring

9.1 Reaction Progress Monitoring of electrochemical reaction

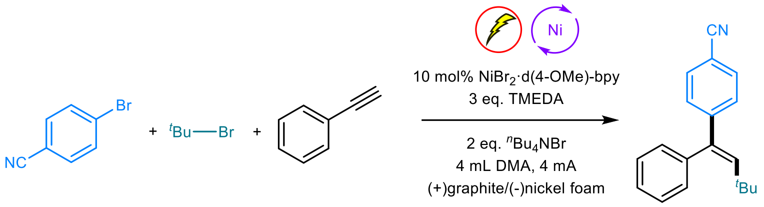

According to the general procedure A, the model reaction (4-bromobenzonitrile, ethynylbenzene, and 2-bromo-2-methylpropane as starting materials) with dodecane as internal standard was conducted on a 0.2 mmol scale. The samples (several drops for each) were collected respectively when the reaction was run for 5,10, 20, 30, 45, 60, 120, 180, 240, 300, 360, 420, 1440 min via syringe and were then passed through a pad of silica gel and washed with ethyl acetate. The yields were determined via GC-FID analysis. The results were shown in Table S15 and Figure S17. The current efficiency at 420 min was 30%.

Table S15: Reaction Progress Monitoring of electrochemical reaction

Time	GC-Yield (%)	*Z:E*
0 min	0	pure *E*
5 min	1	pure *E*
10 min	2	pure *E*
20 min	5	pure *E*
30 min	7	pure *E*
45 min	12	pure *E*
60 min	16	pure *E*
120 min	31	pure *E*
180 min	47	pure *E*
240 min	57	pure *E*
300 min	68	pure *E*
360 min	76	pure *E*
420 min	80	pure *E*
1440 min	85	pure *E*

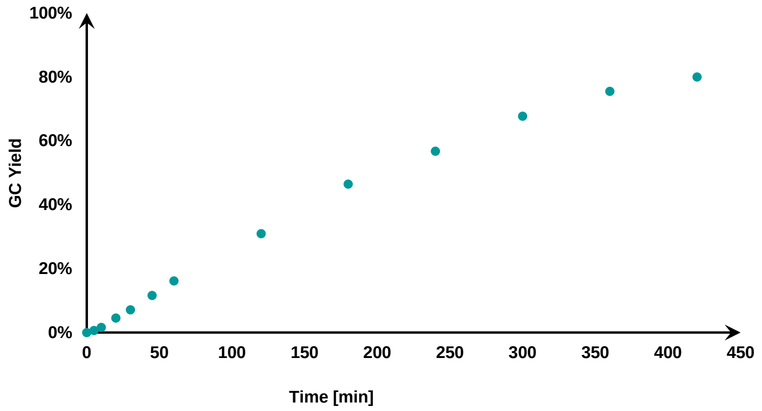

Supplementary Figure 17: Reaction Progress Monitoring of electrochemical reaction.

9.2 Reaction Progress Monitoring of photocatalysis

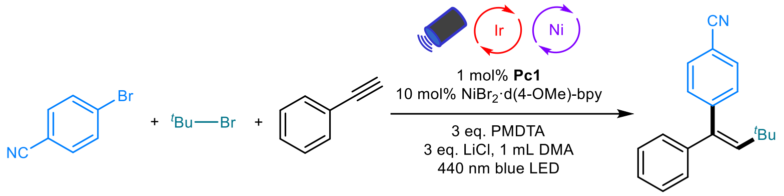

According to the general procedure B, the model reaction (4-bromobenzonitrile, ethynylbenzene, and 2-bromo-2-methylpropane as starting materials) with dodecane as internal standard was conducted on a 0.2 mmol scale. The samples (several drops for each) were collected respectively when the reaction was run for 5,10, 20, 30, 45, 60, 120, 180, 240, 300, 360, 420, 480, 540, 1380 min via syringe and were then passed through a pad of silica gel and washed with ethyl acetate. The yields were determined via GC-FID analysis. The results were shown in Table S16 and Figure S18. The results showed that the SET process (cross-coupling) was finished within 60 min, while the ET process (*Z/E* isomerization) took much longer time compared to SET process.

Table S16: Reaction Progress Monitoring of photocatalysis

Time	GC-Yield (%)	*Z:E*
0 min	0	0:100
5 min	10	0:100
10 min	19	0:100
20 min	44	2:98
30 min	61	5:95
45 min	80	9:91
60 min	84	16:84
120 min	85	35:65
180 min	87	53:47

240 min	87	61:39
300 min	87	69:31
360 min	87	76:24
420 min	87	79:21
480 min	87	82:18
540 min	87	85:15
1380 min	87	94:6

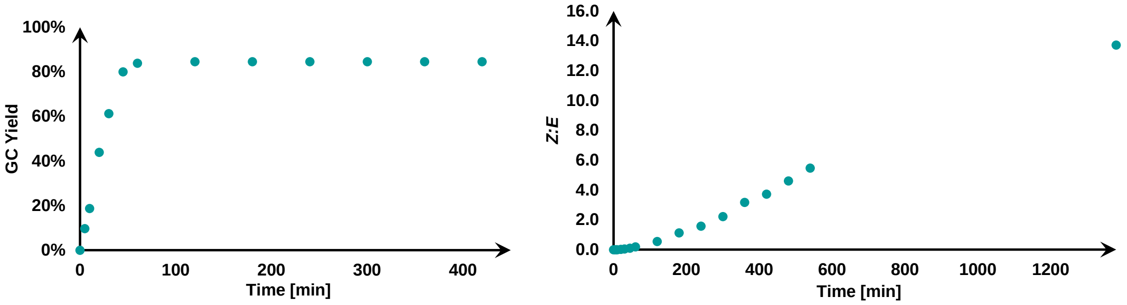

Supplementary Figure 18: Reaction Progress Monitoring of photocatalysis.

9.3 Reaction Progress Monitoring of photo-assisted electrochemical reaction

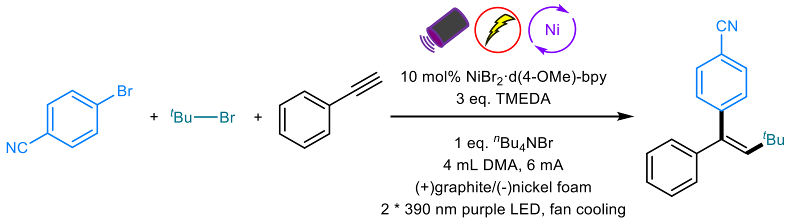

According to the general procedure C, the model reaction (4-bromobenzonitrile, ethynylbenzene, and 2-bromo-2-methylpropane as starting materials) with dodecane as internal standard was conducted on a 0.2 mmol scale. The samples (several drops for each) were collected respectively when the reaction was run for 5,10, 20, 30, 45, 60, 120, 180, 240, 300, 360, 420, 480, 1440, 2880 min via syringe and were then passed through a pad of silica gel and washed with ethyl acetate. The yields were determined via GC-FID analysis. The results were shown in Table S17 and Figure S19. The results showed that the *Z/E* isomerization caused by direct excitation was slower than triplet-triplet energy transfer *Z/E* isomerization.

Table S17: Reaction Progress Monitoring of photo-assisted electrochemical reaction

Time	GC-Yield (%)	*Z:E*
0 min	0	0:100
5 min	2	0:100

10 min	4	0:100
20 min	7	5:95
30 min	10	5:95
45 min	14	5:95
60 min	19	5:95
120 min	40	5:95
180 min	54	5:95
240 min	64	5:95
300 min	71	7:93
360 min	76	12:88
420 min	76	18:82
480 min	76	25:75
1440 min	76	93:7
2880 min	77	93:7

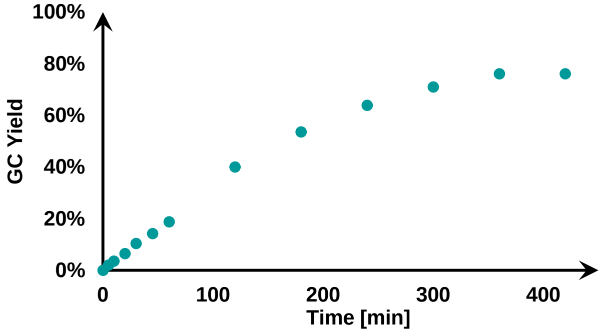

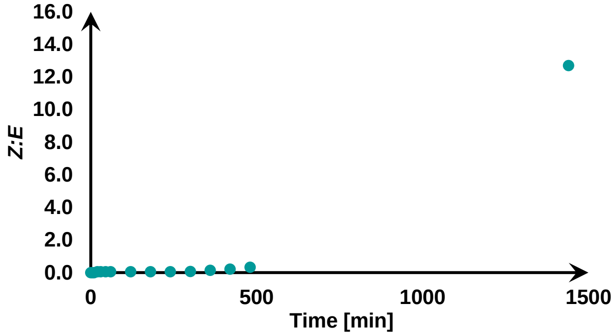

Supplementary Figure 19: Reaction Progress Monitoring of photo-assisted electrochemical reaction.

9.4 On-Off Monitoring of electrochemical reaction:

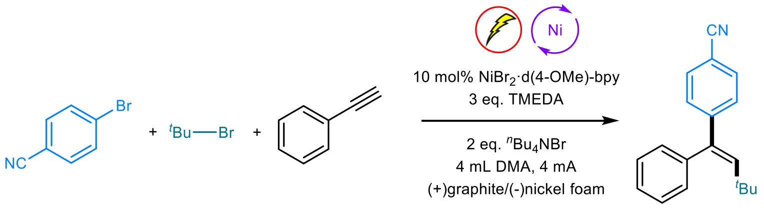

According to the general procedure A, the model reaction (4-bromobenzonitrile, ethynylbenzene, and 2-bromo-2-methylpropane as starting materials) with dodecane as internal standard was conducted on a 0.2 mmol scale. The power supply was switched on/off after a period. The samples (several drops for each) were collected respectively when the reaction was run for 10, 20, 30, 40, 50, 60, 90, 120, 180, 240, 300, 780, 1260 min via syringe and were then passed through a pad of silica gel and washed with ethyl acetate. The yields were determined via GC-FID analysis. The results were shown in Table S18 and Figure S20.

Table S18: On-Off Monitoring of electrochemical reaction

Time	GC-Yield (%)	*Z:E*	On/Off
0 min	0	pure *Z*	Off
10 min	2	pure *Z*	On
20 min	2	pure *Z*	Off
30 min	5	pure *Z*	On
40 min	5	pure *Z*	Off
50 min	8	pure *Z*	On
60 min	8	pure *Z*	Off
90 min	16	pure *Z*	On
120 min	16	pure *Z*	Off
180 min	32	pure *Z*	On
240 min	32	pure *Z*	Off
300 min	45	pure *Z*	On
780 min	45	pure *Z*	Off
1260 min	57	pure *Z*	On

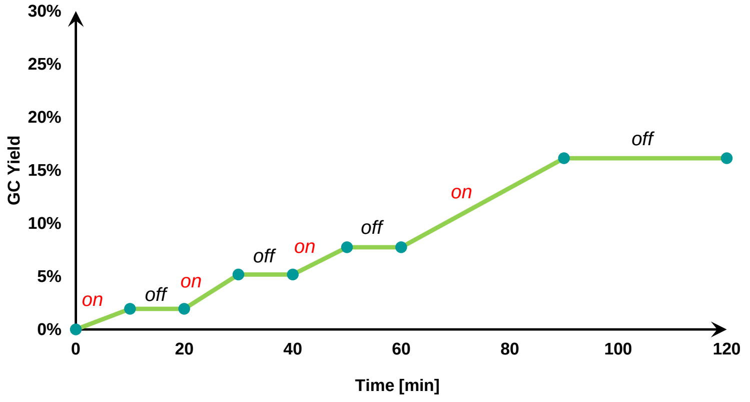

Supplementary Figure 20: On-Off Monitoring of electrochemical reaction.

9.5 On-Off Monitoring of photocatalysis

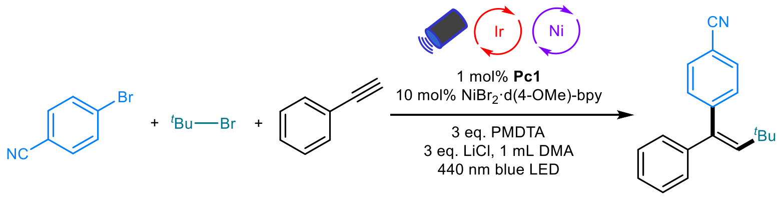

According to the general procedure B, the model reaction (4-bromobenzonitrile, ethynylbenzene, and 2-bromo-2-methylpropane as starting materials) with dodecane as internal standard was conducted on a 0.2 mmol scale. The

Kessil PR160-440 nm lamp was turned on/off after a period. The samples (several drops for each) were collected respectively when the reaction was run for 5, 10, 20, 30, 40, 50, 60, 70, 80, 90, 100, 110, 120, min via syringe and were then passed through a pad of silica gel and washed with ethyl acetate. The yields were determined via GC-FID analysis. The results were shown in Table S19 and Figure S21.

Table S19: On-Off Monitoring of photocatalysis

Time	GC-Yield (%)	*Z:E*	On/Off
0 min	0	0:100	Off
5 min	8	0:100	On
10 min	8	0:100	Off
20 min	14	0:100	On
30 min	14	0:100	Off
40 min	43	2:98	On
50 min	44	2:98	Off
60 min	62	5:95	On
70 min	62	5:95	Off
80 min	72	8:92	On
90 min	72	8:92	Off
100 min	76	12:88	On
110 min	76	12:88	Off
120 min	76	18:82	On

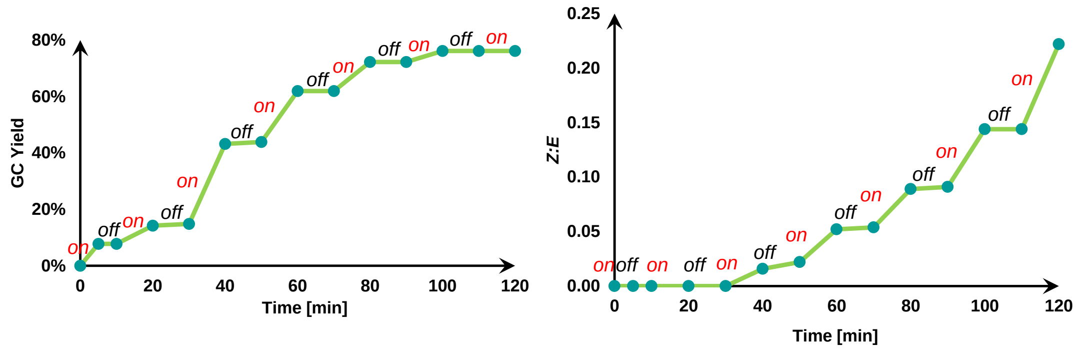

Supplementary Figure 21: On-Off Monitoring of photocatalysis

10.Radical Trap Reactions

10.1 Reaction with TEMPO

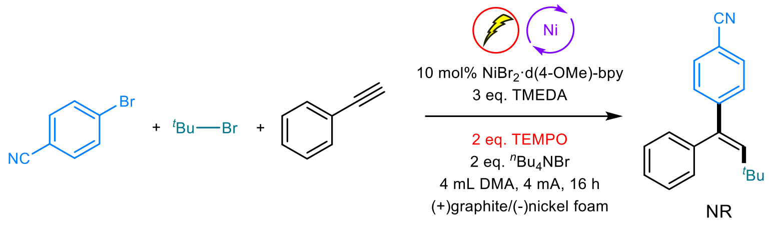

According to the general procedure A, the model reaction (4-bromobenzonitrile, ethynylbenzene, and 2-bromo-2-methylpropane as starting materials) with 2 equiv. TEMPO was conducted on 0.2 mmol scale. No product was formed after the reaction, suggesting that alkyl radical may be involved in the transformation.

10.2 Reaction with 1,1-diphenylethylene

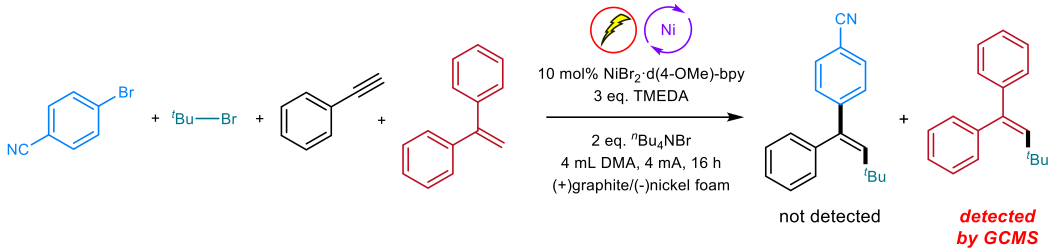

According to the general procedure A, the model reaction (4-bromobenzonitrile, ethynylbenzene, and 2-bromo-2-methylpropane as starting materials) with 2 equiv. 1,1-diphenylethylene was conducted on 0.2 mmol scale. No product was formed after the reaction. The ^{t}Bu-radical was captured by the 1,1-diphenylethylene.

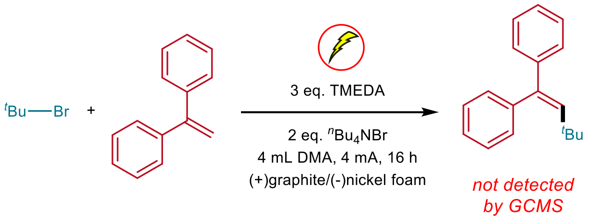

The reaction without nickel catalyst was also conducted. No formation of the (3,3-dimethylbut-1-ene-1,1-diyl)dibenzene was observed. These experimental results show that the ^{t}Bu-radical was generated by the reduction of nickel catalyst, not by the reduction of the cathode.

11.Proposed Mechanism

Based on the mechanistic studies, the reaction mechanism was proposed in Figure S22.

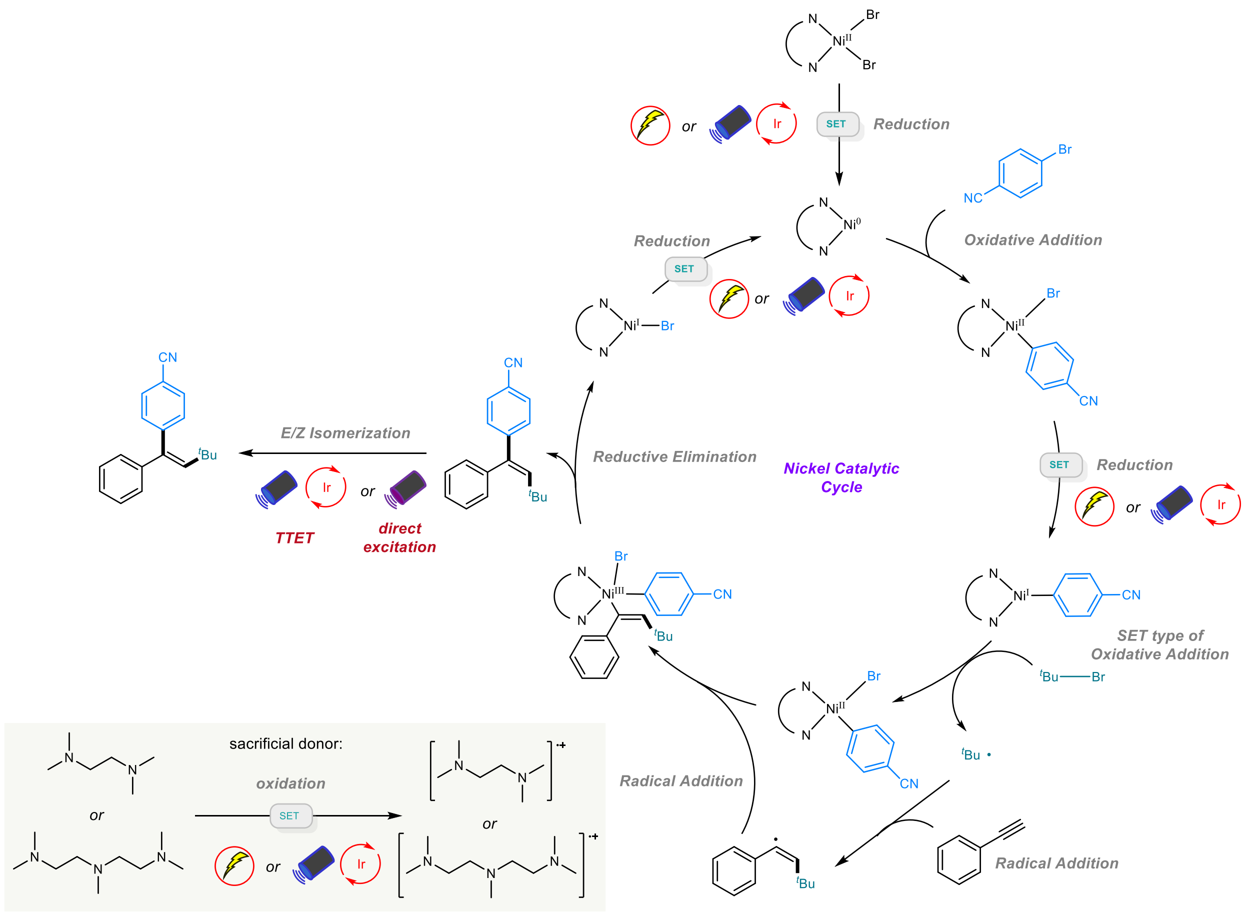

Supplementary Figure 22: Proposed mechanism

12.Spectroscopic Data of the Products

(*E*)-1-(4-(3,3-dimethyl-1-phenylbut-1-en-1-yl)phenyl)ethan-1-one (1)

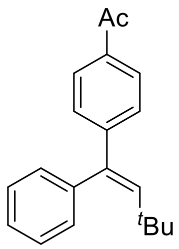

According to the general procedure A, the title compound was isolated as viscous oil after flash chromatography on silica gel (R_f = 0.50, eluent: 100:1 hexane: EtOAc) in 72% yield (40.1 mg). ^{1}H NMR (500 MHz, $CDCl_3$) δ 7.86 – 7.83 (m, 2H), 7.40 – 7.33 (m, 3H), 7.29 (d, *J* = 7.9 Hz, 2H), 7.21 (d, *J* = 7.7 Hz, 2H), 6.23 (s, 1H), 2.58 (s, 3H),

1.01 (s, 9H). ^{13}C NMR (126 MHz, $CDCl_3$) δ 197.7, 148.7, 142.4, 139.9, 138.4, 135.2, 130.3, 128.2, 128.0, 127.1, 126.9, 34.2, 31.2, 26.6. HRMS (ESI) for $C_{20}H_{22}O$: calculated for $[M+Na]^+$ 301.15629, found 301.15542.

(*E*)-(4-(3,3-dimethyl-1-phenylbut-1-en-1-yl)phenyl)(phenyl)methanone (2)

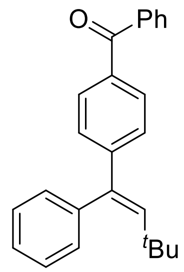

According to the general procedure A, the title compound was isolated as viscous oil after flash chromatography on silica gel (R_f = 0.55, eluent: 100:1 hexane: EtOAc) in 64% yield (43.5 mg). ^1H NMR (400 MHz, $CDCl_3$) δ 7.80 (d, *J* = 7.2 Hz, 2H), 7.72 (d, *J* = 8.3 Hz, 2H), 7.59 (t, *J* = 7.3 Hz, 1H), 7.48 (t, *J* = 7.6 Hz, 2H), 7.43 – 7.35 (m, 3H), 7.32 (d, *J* = 8.3 Hz, 2H), 7.26 – 7.22 (m, 2H), 6.27 (s, 1H), 1.02 (s, 9H). ^{13}C NMR (101 MHz, $CDCl_3$) δ 196.3, 148.2, 142.3, 140.0, 138.5, 137.9, 135.5, 132.2, 130.3, 130.1, 130.0, 128.2, 128.0, 127.1, 126.6, 34.3, 31.2. HRMS (ESI) for $C_{25}H_{24}O$: calculated for $[M+Na]^+$ 363.17194, found 363.17150.

Methyl (*E*)-4-(3,3-dimethyl-1-phenylbut-1-en-1-yl)benzoate (3)

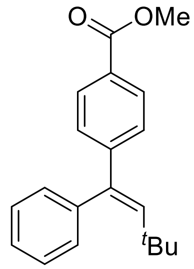

According to the general procedure A, the title compound was isolated as viscous oil after flash chromatography on silica gel (R_f = 0.50, eluent: 100:1 hexane: EtOAc) in 70% yield (41.3 mg). ^1H NMR (400 MHz, $CDCl_3$) δ 7.92 (d, *J* = 8.4 Hz, 2H), 7.41 – 7.34 (m, 3H), 7.27 (d, *J* = 8.4 Hz, 2H), 7.23 – 7.19 (m, 2H), 6.22 (s, 1H), 3.91 (s, 3H), 1.00 (s, 9H). ^{13}C NMR (101 MHz, $CDCl_3$) δ 167.1, 148.6, 142.2, 140.0, 138.5, 130.3, 129.4, 128.1, 128.0, 127.1, 126.8, 52.0, 34.2, 31.2. HRMS (ESI) for $C_{20}H_{22}O$: calculated for $[M+Na]^+$ 317.15120, found 317.15091.

(*E*)-4-(3,3-dimethyl-1-phenylbut-1-en-1-yl)benzonitrile (4)

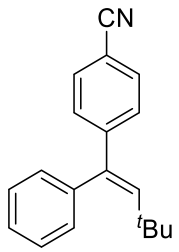

According to the general procedure A, the title compound was isolated as white solid after flash chromatography on silica gel (R_f = 0.62, eluent: hexane) in 80% yield (42.0 mg). ^{1}H NMR (400 MHz, $CDCl_3$) δ 7.53 (d, *J* = 8.5 Hz, 2H), 7.42 – 7.35 (m, 3H), 7.29 (d, *J* = 8.5 Hz, 2H), 7.18 (dd, *J* = 7.6, 1.6 Hz, 2H), 6.22 (s, 1H), 1.00 (s, 9H). ^{13}C NMR (101 MHz, $CDCl_3$) δ 148.5, 143.2, 139.3, 137.9, 131.9, 130.3, 128.1, 127.4, 119.2, 109.9, 34.3, 31.1. HRMS (ESI) for $C_{19}H_{19}N$: calculated for $[M+Na]^+$ 284.14097, found 284.14035.

(*E*)-1-(3,3-dimethyl-1-phenylbut-1-en-1-yl)-4-(methylsulfonyl)benzene (5)

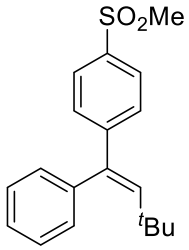

According to the general procedure A, the title compound was isolated as viscous oil after flash chromatography on silica gel (R_f = 0.35, eluent: 4:1 hexane: EtOAc) in 86% yield (54.0 mg). ^{1}H NMR (400 MHz, $CDCl_3$) δ 7.80 (d, *J* = 8.4 Hz, 2H), 7.41 – 7.34 (m, 5H), 7.21 – 7.17 (m, 2H), 6.23 (s, 1H), 3.03 (s, 3H), 1.00 (s, 9H). ^{13}C NMR (101 MHz, $CDCl_3$) δ 149.5, 143.5, 139.5, 138.2, 137.8, 130.2, 128.1, 127.6, 127.4, 127.2, 44.6, 34.4, 31.1. HRMS (ESI) for $C_{19}H_{22}O_2S$: calculated for $[M+Na]^+$ 337.12327, found 337.12422.

(*E*)-1-(3,3-dimethyl-1-phenylbut-1-en-1-yl)-4-(trifluoromethyl)benzene (6)

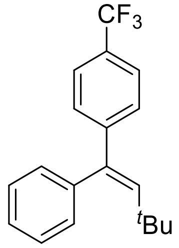

According to the general procedure A, the title compound was isolated as viscous oil after flash chromatography on silica gel (R_f = 0.75, eluent: hexane) in 60% yield (36.5 mg). ^{1}H NMR (500 MHz, $CDCl_3$) δ 7.51 (d, *J* = 8.3 Hz, 2H), 7.41 – 7.35 (m, 3H), 7.32 (d, *J* = 8.2 Hz, 2H), 7.22 (dd, *J* = 8.0, 1.5 Hz, 2H), 6.19 (s, 1H), 1.01 (s, 9H). ^{13}C

NMR (126 MHz, $CDCl_3$) δ 147.6, 142.2, 139.9, 138.2, 130.3, 128.5 (d, *J* = 32.3 Hz), 128.0, 127.2, 127.1, 124.9 (q, *J* = 3.6 Hz), 124.3 (q, *J* = 271.8 Hz), 34.2, 31.2. HRMS (APPI FT-ICR MS) for $C_{19}H_{19}F_3$: calculated for $[M]^+$ 304.14334, found 304.14339.

(*E*)-1-(3,3-dimethyl-1-phenylbut-1-en-1-yl)-4-(trifluoromethoxy)benzene (7)

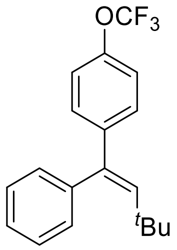

According to the general procedure A, the title compound was isolated as viscous oil after flash chromatography on silica gel (R_f = 0.70, eluent: hexane) in 47% yield (30.1 mg). ^{1}H NMR (500 MHz, $CDCl_3$) δ 7.40 – 7.32 (m, 3H), 7.24 – 7.19 (m, 4H), 7.10 (d, *J* = 8.1 Hz, 2H), 6.10 (s, 1H), 0.99 (s, 9H). ^{13}C NMR (126 MHz, $CDCl_3$) δ 147.9, 142.8, 140.9, 140.2, 137.9, 130.3, 128.1, 127.9, 127.0, 120.5 (d, *J* = 256.8 Hz), 120.4, 34.1, 31.2. ^{19}F NMR (377 MHz, $CDCl_3$) δ -57.87. HRMS (APPI FT-ICR MS) for $C_{19}H_{19}F_3O$: calculated for $[M]^+$ 320.13825, found 320.13830.

(*E*)-(4-(3,3-dimethyl-1-phenylbut-1-en-1-yl)phenyl)(trifluoromethyl)sulfane (8)

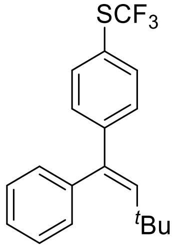

According to the general procedure A, the title compound was isolated as viscous oil after flash chromatography on silica gel (R_f = 0.70, eluent: hexane) in 67% yield (45.1 mg). ^{1}H NMR (400 MHz, $CDCl_3$) δ 7.53 (d, *J* = 8.3 Hz, 2H), 7.42 – 7.34 (m, 3H), 7.26 (d, *J* = 8.4 Hz, 2H), 7.23 – 7.19 (m, 2H), 6.20 (s, 1H), 1.00 (s, 9H). ^{13}C NMR (101 MHz, $CDCl_3$) δ 146.6, 142.1, 139.9, 138.0, 136.0, 131.1, 130.3, 128.0, 127.8, 127.2, 122.0, 34.2, 31.2. ^{19}F NMR (377 MHz, $CDCl_3$) δ -42.90. HRMS (ESI) for $C_{19}H_{19}F_3S$: calculated for $[M+H]^+$ 337.12323, found 337.12138.

(*E*)-4-(3,3-dimethyl-1-phenylbut-1-en-1-yl)benzaldehyde (9)

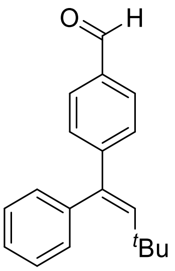

According to the general procedure A, the title compound was isolated as viscous oil after flash chromatography on silica gel (R_f = 0.50, eluent: 50:1 hexane: EtOAc) in 85% yield (44.9 mg). ^{1}H NMR (500 MHz, $CDCl_3$) δ 9.97 (s, 1H), 7.78 – 7.75 (m, 2H), 7.37 (dd, J = 11.7, 7.5 Hz, 5H), 7.21 (d, J = 7.8 Hz, 2H), 6.26 (s, 1H), 1.01 (s, 9H). ^{13}C NMR (126 MHz, $CDCl_3$) δ 191.9, 150.1, 143.1, 139.7, 138.4, 134.6, 130.3, 129.6, 128.0, 127.4, 127.2, 34.3, 31.1. HRMS (ESI) for $C_{19}H_{20}O$: calculated for $[M+Na]^+$ 287.14064, found 287.14065.

(*E*)-3-(3,3-dimethyl-1-phenylbut-1-en-1-yl)benzonitrile (10)

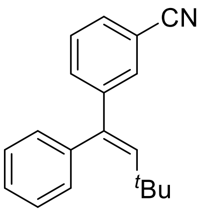

According to the general procedure A, the title compound was isolated as viscous oil after flash chromatography on silica gel (R_f = 0.50, eluent: 100:1 hexane: EtOAc) in 77% yield (40.2 mg). ^{1}H NMR (400 MHz, $CDCl_3$) δ 7.49 – 7.32 (m, 7H), 7.19 (dd, J = 7.5, 1.5 Hz, 2H), 6.14 (s, 1H), 1.00 (s, 9H). ^{13}C NMR (101 MHz, $CDCl_3$) δ 145.2, 142.3, 139.4, 137.4, 131.1, 130.5, 130.2, 130.0, 128.8, 128.2, 127.4, 119.1, 112.1, 34.2, 31.1. HRMS (ESI) for $C_{19}H_{19}N$: calculated for $[M+Na]^+$ 284.14097, found 284.14176.

(*E*)-2-(3,3-dimethyl-1-phenylbut-1-en-1-yl)benzonitrile (11)

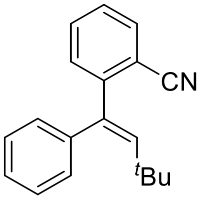

According to the general procedure A, the title compound was isolated as viscous oil after flash chromatography on silica gel (R_f = 0.50, eluent: 100:1 hexane: EtOAc) in 50% yield (26.1 mg). ^{1}H NMR (400 MHz, $CDCl_3$) δ 7.67 (d, J = 7.0 Hz, 1H), 7.42 (t, J = 7.1 Hz, 1H), 7.38 – 7.28 (m, 6H), 7.19 (d, J = 7.9 Hz, 1H), 6.02 (s, 1H), 1.06 (s, 9H).

^{13}C NMR (101 MHz, $CDCl_3$) δ 148.7, 145.7, 139.9, 136.0, 133.4, 132.0, 130.0, 129.8, 128.0, 127.3, 126.8, 119.0, 111.3, 34.6, 30.8. HRMS (ESI) for $C_{19}H_{19}N$: calculated for $[M+Na]^+$ 284.14097, found 284.14121.

(*E*)-4-(3,3-dimethyl-1-phenylbut-1-en-1-yl)-2-fluorobenzonitrile (12)

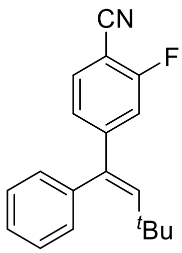

According to the general procedure A, the title compound was isolated as light yellow solid after flash chromatography on silica gel (R_f = 0.55, eluent: 100:1 hexane: EtOAc) in 41% yield (23.1 mg). ^1H NMR (400 MHz, $CDCl_3$) δ 7.50 – 7.45 (m, 1H), 7.43 – 7.36 (m, 3H), 7.17 (dd, J = 7.3, 1.8 Hz, 2H), 7.09 (dd, J = 8.2, 1.5 Hz, 1H), 7.00 (dd, J = 10.9, 1.5 Hz, 1H), 6.25 (s, 1H), 0.99 (s, 9H). ^{13}C NMR (101 MHz, $CDCl_3$) δ 163.1 (d, J = 257.6 Hz), 151.5, 151.4, 144.3, 138.7, 137.1, 132.8, 130.2, 128.3, 127.7, 122.9, 114.5, 114.3, 34.4, 31.0. ^{19}F NMR (377 MHz, $CDCl_3$) δ -107.17. HRMS (ESI) for $C_{19}H_{19}FN$: calculated for $[M+Na]^+$ 302.13155, found 302.13200.

(*E*)-2-(3,3-dimethyl-1-phenylbut-1-en-1-yl)naphthalene (13)

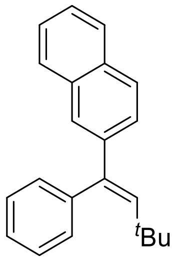

According to the general procedure A, the title compound was isolated as white solid after flash chromatography on silica gel (R_f = 0.60, eluent: hexane) in 40% yield (23.1 mg). ^1H NMR (400 MHz, $CDCl_3$) δ 7.82 – 7.78 (m, 1H), 7.77 – 7.72 (m, 2H), 7.54 – 7.48 (m, 2H), 7.46 – 7.36 (m, 5H), 7.31 – 7.28 (m, 2H), 6.28 (s, 1H), 1.05 (s, 9H). ^{13}C NMR (101 MHz, $CDCl_3$) δ 141.5, 140.7, 139.1, 133.3, 132.3, 130.5, 128.2, 127.8, 127.4, 126.9, 126.0, 125.9, 125.6, 125.2, 34.1, 31.4. HRMS (ESI) for $C_{22}H_{22}$: calculated for $[M+Na]^+$ 309.16137, found 309.16185.

(*E*)-5-(3,3-dimethyl-1-phenylbut-1-en-1-yl)isobenzofuran-1(3*H*)-one (14)

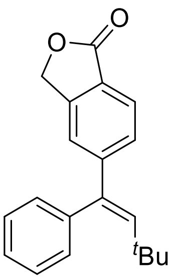

According to the general procedure A, the title compound was isolated as white solid after flash chromatography on silica gel (R_f = 0.52, eluent: 25:1 hexane: EtOAc) in 80% yield (46.8 mg). ^{1}H NMR (500 MHz, $CDCl_3$) δ 7.79 (d, *J* = 8.1 Hz, 1H), 7.45 – 7.35 (m, 4H), 7.21 (dd, *J* = 7.1, 5.5 Hz, 3H), 6.24 (s, 1H), 5.24 (s, 2H), 1.00 (s, 9H). ^{13}C NMR (126 MHz, $CDCl_3$) δ 171.1, 150.4, 146.8, 143.6, 139.7, 138.3, 130.3, 128.1, 128.0, 127.4, 125.2, 123.8, 120.3, 69.6, 34.4, 31.1. HRMS (ESI) for $C_{20}H_{20}O_2$: calculated for $[M+Na]^+$ 315.13555, found 315.13554.

(*E*)-3,3-dimethyl-5-phenyl-5-(quinolin-3-yl)pent-4-en-1-yl 4-methoxybenzoate (15)

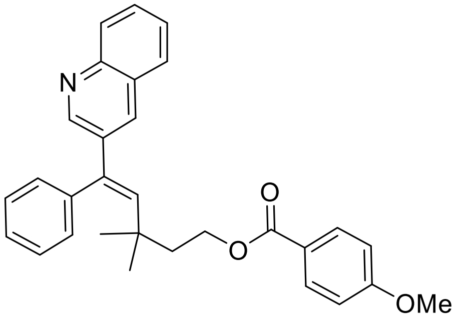

According to the general procedure A, the title compound was isolated as viscous oil after flash chromatography on silica gel (R_f = 0.40, eluent: 10:1 hexane: EtOAc) in 71% yield (64.0 mg). ^{1}H NMR (400 MHz, $CDCl_3$) δ 8.95 – 8.89 (m, 1H), 8.06 (d, *J* = 8.8 Hz, 1H), 7.91 (d, *J* = 8.9 Hz, 2H), 7.70 – 7.62 (m, 3H), 7.48 (t, *J* = 7.5 Hz, 1H), 7.43 – 7.35 (m, 3H), 7.28 (d, *J* = 6.4 Hz, 2H), 6.73 (d, *J* = 8.9 Hz, 2H), 6.31 (s, 1H), 4.44 (t, *J* = 6.9 Hz, 2H), 3.73 (s, 3H), 1.89 (t, *J* = 6.9 Hz, 2H), 1.07 (s, 6H). ^{13}C NMR (101 MHz, $CDCl_3$) δ 166.4, 163.2, 149.6, 146.9, 140.3, 139.3, 137.5, 136.4, 133.0, 131.5, 130.1, 129.0, 128.3, 128.0, 127.6, 127.4, 126.7, 126.1, 122.6, 113.5, 62.2, 55.3, 42.7, 36.7, 29.3. HRMS (ESI) for $C_{30}H_{29}NO_3$: calculated for $[M+H]^+$ 452.22202, found 452.22206.

(*E*)-4-(3,3-dimethyl-1-phenylbut-1-en-1-yl)phenyl 4-(*N,N*-dipropylsulfamoyl)benzoate (16)

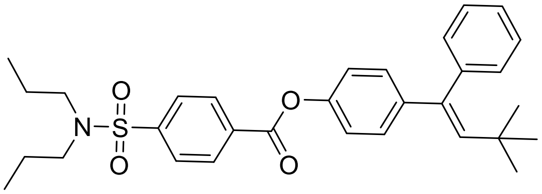

According to the general procedure A, the title compound was isolated as viscous oil after flash chromatography on silica gel (R_f = 0.35, eluent: 30:1 hexane: EtOAc) in 48% yield (50.2 mg). ^{1}H NMR (400 MHz, $CDCl_3$) δ 8.32 (d, *J* = 8.4 Hz, 2H), 7.96 (d, *J* = 8.4 Hz, 2H), 7.41 – 7.31 (m, 3H), 7.28 – 7.20 (m, 4H), 7.10 (d, *J* = 8.6 Hz, 2H), 6.12 (s, 1H), 3.17 – 3.12 (m, 4H), 1.58 (dq, *J* = 14.9, 7.6 Hz, 4H), 0.99 (s, 9H), 0.90 (t, *J* = 7.4 Hz, 6H). ^{13}C NMR (101 MHz, $CDCl_3$) δ 163.9, 149.3, 144.8, 142.2, 140.6, 140.4, 138.2, 132.9, 130.8, 130.3, 128.0, 127.9, 127.2, 126.9, 120.9, 49.9, 34.1, 31.3, 21.9, 11.2. HRMS (ESI) for $C_{31}H_{37}NO_4S$: calculated for $[M+Na]^+$ 542.23355, found 542.23417.

(*E*)-3,3-dimethyl-5-phenyl-5-(4-((((5*R*,5a*S*,8a*S*,8b*R*)-2,2,7,7-tetramethyltetrahydro-5*H*-bis([1,3]dioxolo)[4,5-b:4',5'-d]pyran-5-yl)methoxy)carbonyl)phenyl)pent-4-en-1-yl 4-methoxybenzoate (17)

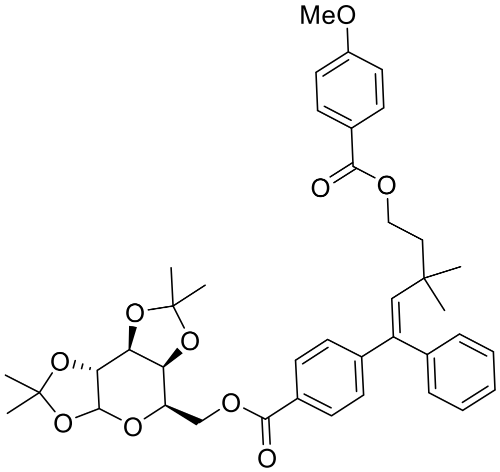

According to the general procedure A, the title compound was isolated as viscous oil after flash chromatography on silica gel (R_f = 0.42, eluent: 10:1 hexane: EtOAc) in 59% yield (81.2 mg). ^{1}H NMR (400 MHz, $CDCl_3$) δ 7.92 (t, *J* = 8.7 Hz, 4H), 7.36 (q, *J* = 7.3, 6.7 Hz, 3H), 7.24 (d, *J* = 8.4 Hz, 2H), 7.22 – 7.19 (m, 2H), 6.83 (d, *J* = 8.8 Hz, 2H), 6.22 (s, 1H), 5.59 – 5.56 (m, 1H), 4.66 (dd, *J* = 7.9, 2.3 Hz, 1H), 4.51 (dd, *J* = 11.5, 5.0 Hz, 1H), 4.46 – 4.31 (m, 5H), 4.18 (t, *J* = 5.6 Hz, 1H), 3.84 (s, 3H), 1.84 (t, *J* = 7.0 Hz, 2H), 1.51 (d, *J* = 11.6 Hz, 6H), 1.36 (d, *J* = 9.7 Hz, 6H), 1.02 (s, 6H). ^{13}C NMR (101 MHz, $CDCl_3$) δ 166.4, 166.3, 163.3, 148.3, 140.0, 139.7, 139.6, 131.5, 130.1, 129.5, 128.13, 128.08, 127.2, 126.8, 122.7, 113.5, 109.7, 108.8, 96.3, 71.1, 70.7, 70.5, 66.1, 63.7, 62.3, 55.4, 42.7, 36.5, 29.3, 26.1, 26.0, 25.0, 24.5. HRMS (ESI) for $C_{40}H_{46}O_{10}$: calculated for $[M+Na]^+$ 709.29832, found 709.29834.

(*Z*)-1-(4-(1-(4-methoxyphenyl)-3,3-dimethylbut-1-en-1-yl)phenyl)ethan-1-one (18)

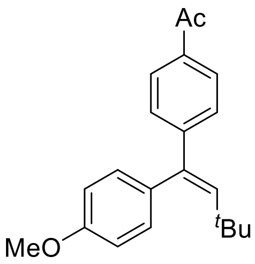

According to the general procedure A, the title compound was isolated as viscous oil after flash chromatography on silica gel (R_f = 0.55, eluent: 50:1 hexane: EtOAc) in 61% yield (37.6 mg). ^{1}H NMR (500 MHz, $CDCl_3$) δ 7.84 (d, J = 8.6 Hz, 2H), 7.28 (d, J = 8.6 Hz, 2H), 7.10 (d, J = 8.7 Hz, 2H), 6.92 (d, J = 8.7 Hz, 2H), 6.21 (s, 1H), 3.86 (s, 3H), 2.57 (s, 3H), 1.01 (s, 9H). ^{13}C NMR (126 MHz, $CDCl_3$) δ 197.7, 158.7, 149.1, 142.6, 138.1, 135.2, 132.1, 131.3, 128.2, 126.9, 113.4, 55.2, 34.2, 31.2, 26.6. HRMS (ESI) for $C_{21}H_{24}O_2$: calculated for $[M+Na]^+$ 331.16685, found 331.1676.

(*Z*)-1-(4-(3,3-dimethyl-1-(4-phenoxyphenyl)but-1-en-1-yl)phenyl)ethan-1-one (19)

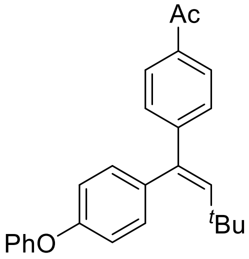

According to the general procedure A, the title compound was isolated as viscous oil after flash chromatography on silica gel (R_f = 0.40, eluent: 10:1 hexane: EtOAc) in 50% yield (37.2 mg). ^{1}H NMR (500 MHz, $CDCl_3$) δ 7.89 – 7.85 (m, 2H), 7.39 (t, J = 7.9 Hz, 2H), 7.31 (d, J = 8.4 Hz, 2H), 7.16 (d, J = 8.4 Hz, 3H), 7.09 (d, J = 8.3 Hz, 2H), 7.03 (d, J = 8.4 Hz, 2H), 6.23 (s, 1H), 2.59 (s, 3H), 1.03 (s, 9H). ^{13}C NMR (126 MHz, $CDCl_3$) δ 197.7, 157.1, 156.5, 148.8, 142.8, 137.8, 135.3, 134.8, 131.6, 129.8, 128.2, 126.9, 123.4, 119.0, 118.3, 34.3, 31.2, 26.6. HRMS (ESI) for $C_{26}H_{26}O_2$: calculated for $[M+Na]^+$ 393.18250, found 393.18164.

(*E*)-1-(4-(1-(4-ethylphenyl)-3,3-dimethylbut-1-en-1-yl)phenyl)ethan-1-one (20)

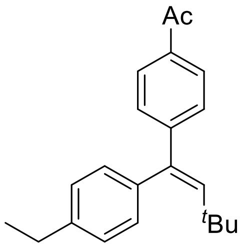

According to the general procedure A, the title compound was isolated as viscous oil after flash chromatography on silica gel (R_f = 0.70, eluent: 50:1 hexane: EtOAc) in 60% yield (36.5 mg). ^{1}H NMR (500 MHz, $CDCl_3$) δ 7.84 (d, *J* = 8.2 Hz, 2H), 7.29 (d, *J* = 8.1 Hz, 2H), 7.21 (d, *J* = 7.7 Hz, 2H), 7.10 (d, *J* = 7.6 Hz, 2H), 6.21 (s, 1H), 2.74 – 2.69 (m, 2H), 2.57 (s, 3H), 1.30 (t, *J* = 7.5 Hz, 3H), 1.00 (s, 9H). ^{13}C NMR (126 MHz, $CDCl_3$) δ 197.7, 149.0, 143.1, 142.3, 138.5, 137.1, 135.1, 130.2, 128.2, 127.4, 126.9, 34.2, 31.2, 28.6, 26.6, 15.5. HRMS (ESI) for $C_{22}H_{26}O$: calculated for $[M+Na]^+$ 329.18759, found 329.18786.

(*Z*)-1-(4-(1-(4-(dimethylamino)phenyl)-3,3-dimethylbut-1-en-1-yl)phenyl)ethan-1-one (21)

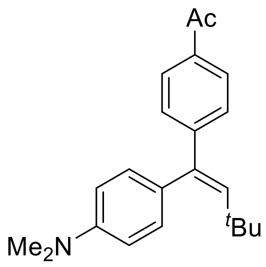

According to the general procedure A, the title compound was isolated as viscous oil after flash chromatography on silica gel (R_f = 0.55, eluent: 20:1 hexane: EtOAc) in 47% yield (30.2mg). ^{1}H NMR (500 MHz, $CDCl_3$) δ 7.83 (d, *J* = 8.3 Hz, 2H), 7.31 (d, *J* = 8.4 Hz, 2H), 7.03 (d, *J* = 8.5 Hz, 2H), 6.74 (d, *J* = 8.5 Hz, 2H), 6.18 (s, 1H), 3.00 (s, 6H), 2.57 (s, 3H), 1.03 (s, 9H). ^{13}C NMR (126 MHz, $CDCl_3$) δ 197.8, 149.8, 149.5, 142.3, 138.7, 135.0, 131.0, 128.1, 127.7, 127.0, 111.8, 40.5, 34.2, 31.3, 26.6. HRMS (ESI) for $C_{22}H_{27}NO$: calculated for $[M+Na]^+$ 344.19849, found 344.19850.

(*E*)-1-(4-(3,3-dimethyl-1-(4-(4,4,5,5-tetramethyl-1,3,2-dioxaborolan-2-yl)phenyl)but-1-en-1-yl)phenyl)ethan-1-one (22)

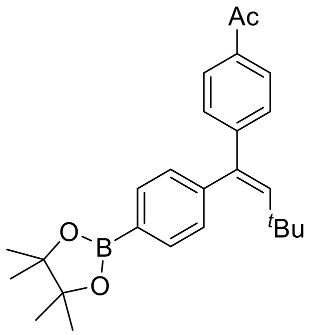

According to the general procedure A, the title compound was isolated as viscous oil after flash chromatography on silica gel (R_f = 0.45, eluent: 20:1 hexane: EtOAc) in 37% yield (30.0 mg). ^{1}H NMR (500 MHz, $CDCl_3$) δ 7.82 (d, *J* = 7.9 Hz, 4H), 7.26 (d, *J* = 8.2 Hz, 2H), 7.22 (d, *J* = 7.4 Hz, 2H), 6.22 (s, 1H), 2.57 (s, 3H), 1.39 (s, 12H), 0.99 (s,

9H). ^{13}C NMR (126 MHz, $CDCl_3$) δ 197.7, 148.5, 143.1, 142.4, 138.4, 135.2, 134.4, 131.3, 129.7, 128.2, 126.9, 83.9, 34.2, 31.2, 26.6, 24.9. HRMS (ESI) for $C_{26}H_{33}BO_3$: calculated for $[M+Na]^+$ 427.24195, found 427.24160.

(*Z*)-1-(4-(1-(4-chlorophenyl)-3,3-dimethylbut-1-en-1-yl)phenyl)ethan-1-one (23)

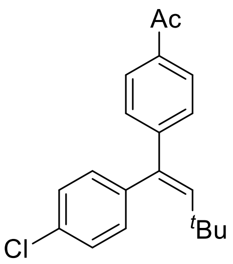

According to the general procedure A, the title compound was isolated as white solid after flash chromatography on silica gel (R_f = 0.50, eluent: 50:1 hexane: EtOAc) in 52% yield (32.5 mg). ^{1}H NMR (500 MHz, $CDCl_3$) δ 7.85 (d, *J* = 8.2 Hz, 2H), 7.36 (d, *J* = 8.1 Hz, 2H), 7.25 (d, *J* = 8.2 Hz, 2H), 7.14 (d, *J* = 8.1 Hz, 2H), 6.23 (s, 1H), 2.58 (s, 3H), 1.00 (s, 9H). ^{13}C NMR (126 MHz, $CDCl_3$) δ 197.6, 148.2, 143.0, 138.4, 137.2, 135.4, 133.2, 131.6, 128.3, 126.9, 34.3, 31.2, 26.6. HRMS (ESI) for $C_{20}H_{21}ClO$: calculated for $[M+Na]^+$ 335.11731, found 335.11680.

(*Z*)-1-(4-(1-(4-fluorophenyl)-3,3-dimethylbut-1-en-1-yl)phenyl)ethan-1-one (24)

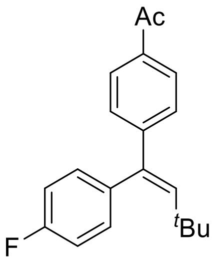

According to the general procedure A, the title compound was isolated as viscous oil after flash chromatography on silica gel (R_f = 0.50, eluent: 50:1 hexane: EtOAc) in 58% yield (34.3 mg). ^{1}H NMR (500 MHz, $CDCl_3$) δ 7.85 (d, *J* = 8.4 Hz, 2H), 7.26 (d, *J* = 8.4 Hz, 2H), 7.16 (dd, *J* = 8.3, 5.7 Hz, 2H), 7.08 (t, *J* = 8.6 Hz, 2H), 6.24 (s, 1H), 2.58 (s, 3H), 1.00 (s, 9H). ^{13}C NMR (126 MHz, $CDCl_3$) δ 197.7, 162.1 (d, *J* = 246.2 Hz), 148.5, 143.0, 137.3, 135.8, 135.3, 131.8 (d, *J* = 7.9 Hz), 128.2, 126.9, 115.0 (d, *J* = 21.3 Hz), 34.3, 31.2, 26.6. ^{19}F NMR (471 MHz, $CDCl_3$) δ -115.16. HRMS (ESI) for $C_{20}H_{21}FO$: calculated for $[M+Na]^+$ 319.14686, found 319.14696.

(*Z*)-1-(4-(3,3-dimethyl-1-(4-(trifluoromethyl)phenyl)but-1-en-1-yl)phenyl)ethan-1-one (25)

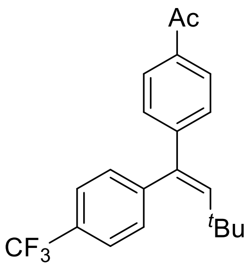

According to the general procedure A, the title compound was isolated as white solid after flash chromatography on silica gel (R_f = 0.50, eluent: 50:1 hexane: EtOAc) in 60% yield (41.5 mg). ^{1}H NMR (400 MHz, $CDCl_3$) δ 7.86 (d, *J* = 8.4 Hz, 2H), 7.65 (d, *J* = 8.1 Hz, 2H), 7.35 (d, *J* = 8.0 Hz, 2H), 7.24 (d, *J* = 8.4 Hz, 2H), 6.28 (s, 1H), 2.58 (s, 3H), 1.00 (s, 9H). ^{13}C NMR (101 MHz, $CDCl_3$) δ 197.6, 147.8, 144.0, 143.2, 137.1, 135.5, 130.7, 129.5 (d, *J* = 32.5 Hz), 128.3, 126.9, 125.0 (q, *J* = 3.4 Hz), 124.2 (d, *J* = 271.9 Hz), 34.3, 31.2, 26.6. ^{19}F NMR (377 MHz, $CDCl_3$) δ -62.40. HRMS (ESI) for $C_{21}H_{21}F_3O$: calculated for $[M+Na]^+$ 369.14367, found 369.14361.

(*Z*)-1-(4-(3,3-dimethyl-1-(4-(trifluoromethoxy)phenyl)but-1-en-1-yl)phenyl)ethan-1-one (26)

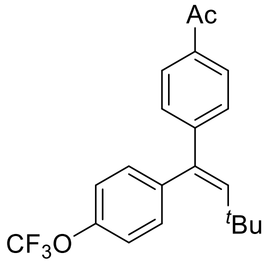

According to the general procedure A, the title compound was isolated as viscous oil after flash chromatography on silica gel (R_f = 0.45, eluent: 50:1 hexane: EtOAc) in 50% yield (36.2 mg). ^{1}H NMR (400 MHz, $CDCl_3$) δ 7.86 (d, *J* = 8.3 Hz, 2H), 7.28 – 7.22 (m, 6H), 6.25 (s, 1H), 2.58 (s, 3H), 1.00 (s, 9H). ^{13}C NMR (101 MHz, $CDCl_3$) δ 197.6, 148.5, 148.1, 143.2, 138.6, 137.0, 135.4, 131.7, 128.3, 126.9, 120.5 (d, *J* = 257.3 Hz), 120.4, 34.3, 31.2, 26.6. ^{19}F NMR (377 MHz, $CDCl_3$) δ -57.83. HRMS (ESI) for $C_{21}H_{21}F_3O_2$: calculated for $[M+Na]^+$ 385.13859, found 385.13852.

Methyl (*Z*)-4-(1-(4-acetylphenyl)-3,3-dimethylbut-1-en-1-yl)benzoate (27)

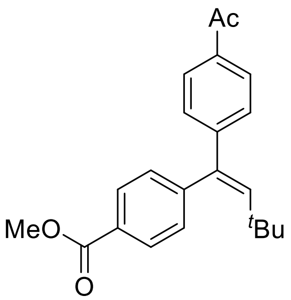

According to the general procedure A, the title compound was isolated as viscous oil after flash chromatography on silica gel (R_f = 0.38, eluent: 50:1 hexane: EtOAc) in 60% yield (37.6 mg). ^{1}H NMR (500 MHz, $CDCl_3$) δ 8.06 (d, *J* = 8.2 Hz, 2H), 7.84 (d, *J* = 8.4 Hz, 2H), 7.30 (d, *J* = 8.2 Hz, 2H), 7.24 (d, *J* = 8.4 Hz, 2H), 6.25 (s, 1H), 3.96 (s, 3H), 2.57 (s, 3H), 0.99 (s, 9H). ^{13}C NMR (126 MHz, $CDCl_3$) δ 197.6, 166.9, 147.9, 145.2, 142.9, 137.4, 135.4, 130.4, 129.3, 129.1, 128.3, 126.9, 52.2, 34.3, 31.2, 26.6. HRMS (ESI) for $C_{22}H_{24}O_3$: calculated for $[M+Na]^+$ 359.16177, found 359.1618.

(*E*)-1-(4-(1-([1,1'-biphenyl]-4-yl)-3,3-dimethylbut-1-en-1-yl)phenyl)ethan-1-one (28)

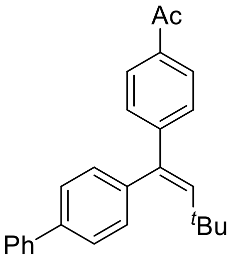

According to the general procedure A, the title compound was isolated as viscous oil after flash chromatography on silica gel (R_f = 0.42, eluent: 50:1 hexane: EtOAc) in 60% yield (42.5 mg). ^{1}H NMR (500 MHz, $CDCl_3$) δ 7.87 (d, *J* = 8.3 Hz, 2H), 7.68 (d, *J* = 8.2 Hz, 2H), 7.64 (d, *J* = 8.0 Hz, 2H), 7.49 (t, *J* = 7.6 Hz, 2H), 7.39 (t, *J* = 7.4 Hz, 1H), 7.34 (d, *J* = 8.3 Hz, 2H), 7.28 (d, *J* = 7.9 Hz, 2H), 6.26 (s, 1H), 2.59 (s, 3H), 1.05 (s, 9H). ^{13}C NMR (126 MHz, $CDCl_3$) δ 197.7, 148.7, 142.7, 140.7, 139.9, 139.0, 138.1, 135.3, 130.7, 128.8, 128.3, 127.4, 127.02, 127.00, 126.6, 34.3, 31.3, 26.6. HRMS (ESI) for $C_{26}H_{26}O$: calculated for $[M+Na]^+$ 377.18759, found 377.18776.

(*Z*)-1-(4-(1-(2-methoxyphenyl)-3,3-dimethylbut-1-en-1-yl)phenyl)ethan-1-one (29)

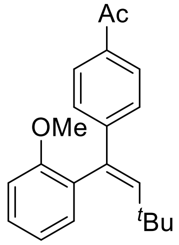

According to the general procedure A, the title compound was isolated as light yellow solid after flash chromatography on silica gel (R_f = 0.45, eluent: 30:1 hexane: EtOAc) in 68% yield (41.9 mg). ^{1}H NMR (500 MHz, $CDCl_3$) δ 7.84 (d, *J* = 8.5 Hz, 2H), 7.35 (td, *J* = 8.3, 1.6 Hz, 1H), 7.32 (d, *J* = 8.5 Hz, 2H), 7.13 (dd, *J* = 7.3, 1.5 Hz, 1H), 6.99 (t, *J* = 7.4 Hz, 1H), 6.93 (d, *J* = 8.2 Hz, 1H), 6.27 (s, 1H), 3.74 (s, 3H), 2.57 (s, 3H), 0.99 (s, 9H). ^{13}C NMR (126 MHz, $CDCl_3$) δ 197.8, 157.2, 148.3, 142.7, 135.1, 134.6, 131.8, 128.9, 128.8, 128.2, 126.4, 120.1, 110.7, 55.3, 34.2, 30.5, 26.6. HRMS (ESI) for $C_{21}H_{24}O_2$: calculated for $[M+Na]^+$ 331.16685, found 331.16781.

(*Z*)-1-(4-(3,3-dimethyl-1-(m-tolyl)but-1-en-1-yl)phenyl)ethan-1-one (30)

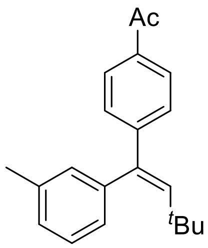

According to the general procedure A, the title compound was isolated as viscous oil after flash chromatography on silica gel (R_f = 0.45, eluent: 50:1 hexane: EtOAc) in 78% yield (45.6 mg). ^{1}H NMR (500 MHz, $CDCl_3$) δ 7.85 (d, *J* = 8.5 Hz, 2H), 7.29 (d, *J* = 8.6 Hz, 2H), 7.26 (d, *J* = 7.9 Hz, 1H), 7.15 (d, *J* = 7.5 Hz, 1H), 7.01 (d, *J* = 6.8 Hz, 2H), 6.20 (s, 1H), 2.58 (s, 3H), 2.38 (s, 3H), 1.01 (s, 9H). ^{13}C NMR (126 MHz, $CDCl_3$) δ 197.7, 148.9, 142.2, 139.8, 138.5, 137.5, 135.2, 130.9, 128.2, 127.80, 127.79, 127.4, 126.9, 34.2, 31.2, 26.6, 21.5. HRMS (ESI) for $C_{21}H_{24}O$: calculated for $[M+Na]^+$ 315.17194, found 315.17275.

(*Z*)-1-(4-(3,3-dimethyl-1-(o-tolyl)but-1-en-1-yl)phenyl)ethan-1-one (31)

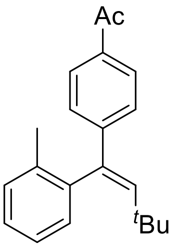

According to the general procedure A, the title compound was isolated as viscous oil after flash chromatography on silica gel (R_f = 0.45, eluent: 50:1 hexane: EtOAc) in 78% yield (45.6 mg). ^{1}H NMR (500 MHz, $CDCl_3$) δ 7.85 (d, *J* = 8.4 Hz, 2H), 7.30 – 7.24 (m, 4H), 7.21 (d, *J* = 7.4 Hz, 2H), 6.27 (s, 1H), 2.58 (s, 3H), 2.08 (s, 3H), 0.98 (s, 9H). ^{13}C NMR (126 MHz, $CDCl_3$) δ 197.7, 147.7, 142.1, 139.1, 137.2, 136.5, 135.3, 130.9, 130.1, 128.3, 127.6, 126.5, 125.2, 34.3, 30.6, 26.6, 19.9. HRMS (ESI) for $C_{21}H_{24}O$: calculated for $[M+Na]^+$ 315.17194, found 315.17291.

(*Z*)-1-(4-(3,3-dimethyl-1-(2,4,5-trimethylphenyl)but-1-en-1-yl)phenyl)ethan-1-one (32)

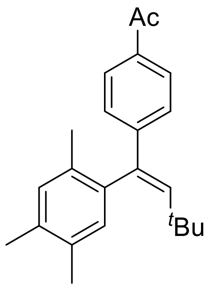

According to the general procedure A, the title compound was isolated as viscous oil after flash chromatography on silica gel (R_f = 0.45, eluent: 50:1 hexane: EtOAc) in 73% yield (46.7 mg). ^{1}H NMR (500 MHz, $CDCl_3$) δ 7.85 – 7.82 (m, 2H), 7.29 (d, *J* = 8.5 Hz, 2H), 6.96 (d, *J* = 11.8 Hz, 2H), 6.23 (s, 1H), 2.57 (s, 3H), 2.29 (s, 6H), 2.00 (s, 3H), 0.99 (s, 9H). ^{13}C NMR (126 MHz, $CDCl_3$) δ 197.7, 148.1, 141.9, 137.4, 136.4, 135.5, 135.1, 133.5, 133.0, 132.0, 131.4, 128.3, 126.5, 34.3, 30.6, 26.6, 19.5, 19.4, 19.3. HRMS (ESI) for $C_{23}H_{28}O$: calculated for $[M+Na]^+$ 343.20324, found 343.20231.

(*Z*)-1-(4-(1-(3-hydroxyphenyl)-3,3-dimethylbut-1-en-1-yl)phenyl)ethan-1-one (33)

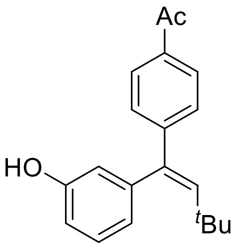

According to the general procedure A, the title compound was isolated as light yellow solid after flash chromatography on silica gel (R_f = 0.30, eluent: 4:1 hexane: EtOAc) in 63% yield (37.1 mg). ^{1}H NMR (500 MHz, $CDCl_3$) δ 7.83 (d, *J* = 8.3 Hz, 2H), 7.29 (d, *J* = 8.0 Hz, 2H), 7.24 (t, *J* = 7.8 Hz, 1H), 6.86 (d, *J* = 5.9 Hz, 1H), 6.76 (d, *J* = 7.5 Hz, 1H), 6.72 (s, 1H), 6.18 (s, 1H), 6.00 (s, 1H), 2.58 (s, 3H), 1.02 (s, 9H). ^{13}C NMR (126 MHz, $CDCl_3$) δ 198.6, 155.6, 148.8, 142.4, 141.4, 138.0, 135.0, 129.2, 128.3, 126.9, 122.8, 117.3, 114.2, 34.3, 31.1, 26.6. HRMS (ESI) for $C_{20}H_{22}O_2$: calculated for $[M+Na]^+$ 317.1512, found 317.15139.

(*Z*)-1-(4-(1-(3,5-difluorophenyl)-3,3-dimethylbut-1-en-1-yl)phenyl)ethan-1-one (34)

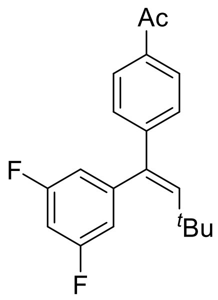

According to the general procedure A, the title compound was isolated as viscous oil after flash chromatography on silica gel (R_f = 0.50, eluent: 50:1 hexane: EtOAc) in 36% yield (23.3 mg). ^{1}H NMR (400 MHz, $CDCl_3$) δ 7.87 (d, J = 8.4 Hz, 2H), 7.25 (d, J = 8.4 Hz, 2H), 6.85 – 6.80 (m, 1H), 6.78 – 6.73 (m, 2H), 6.22 (s, 1H), 2.59 (s, 3H), 1.03 (s, 9H). ^{13}C NMR (101 MHz, $CDCl_3$) δ 197.6, 162.8 (d, J = 249.6 Hz), 162.6 (d, J = 249.6 Hz) 147.3, 143.3, 136.2, 135.6, 128.4, 126.8, 113.4 (d, J = 24.8 Hz), 102.8 (t, J = 25.2 Hz), 34.4, 31.0, 26.6. ^{19}F NMR (377 MHz, $CDCl_3$) δ -110.01. HRMS (ESI) for $C_{20}H_{20}F_2O$: calculated for $[M+Na]^+$ 337.13744, found 337.13744.

(*Z*)-1-(4-(3,3-dimethyl-1-(2-(trifluoromethyl)phenyl)but-1-en-1-yl)phenyl)ethan-1-one (35)

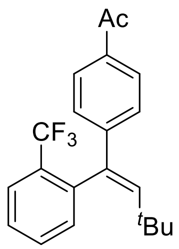

According to the general procedure A, the title compound was isolated as viscous oil after flash chromatography on silica gel (R_f = 0.50, eluent: 50:1 hexane: EtOAc) in 62% yield (43.1 mg). ^{1}H NMR (500 MHz, $CDCl_3$) δ 7.85 (d, J = 8.6 Hz, 2H), 7.71 (d, J = 7.9 Hz, 1H), 7.61 (t, J = 7.5 Hz, 1H), 7.51 (t, J = 7.7 Hz, 1H), 7.40 (d, J = 7.6 Hz, 1H), 7.23 (d, J = 8.6 Hz, 2H), 6.22 (s, 1H), 2.58 (s, 3H), 0.96 (s, 9H). ^{13}C NMR (126 MHz, $CDCl_3$) δ 197.7, 147.8, 143.4, 137.7, 135.3, 134.2, 133.3, 131.0, 129.3 (q, J = 30.0 Hz), 128.2, 127.9, 126.8 (q, J = 4.9 Hz), 126.3, 124.0 (q, J = 274.4 Hz), 34.8, 30.4, 26.6. ^{19}F NMR (471 MHz, $CDCl_3$) δ -59.09. HRMS (ESI) for $C_{21}H_{21}F_3O$: calculated for $[M+Na]^+$ 369.14367, found 369.14395.

(*Z*)-1-(4-(1-(6-methoxynaphthalen-2-yl)-3,3-dimethylbut-1-en-1-yl)phenyl)ethan-1-one (36)

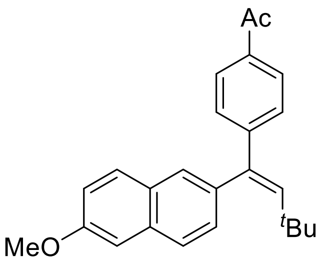

According to the general procedure A, the title compound was isolated as viscous oil after flash chromatography on silica gel (R_f = 0.38, eluent: 20:1 hexane: EtOAc) in 52% yield (37.2 mg). ^{1}H NMR (500 MHz, $CDCl_3$) δ 7.84 (d, *J* = 8.4 Hz, 2H), 7.76 (d, *J* = 8.3 Hz, 2H), 7.63 (s, 1H), 7.32 (d, *J* = 8.4 Hz, 2H), 7.26 (d, *J* = 8.3 Hz, 1H), 7.21 (d, *J* = 7.8 Hz, 2H), 6.31 (s, 1H), 3.97 (s, 3H), 2.57 (s, 3H), 1.02 (s, 9H). ^{13}C NMR (126 MHz, $CDCl_3$) δ 197.7, 157.8, 148.9, 142.8, 138.4, 135.24, 135.16, 133.6, 129.5, 129.1, 128.8, 128.5, 128.2, 127.1, 126.5, 119.1, 105.7, 55.4, 34.3, 31.3, 26.6. HRMS (ESI) for $C_{25}H_{26}O_2$: calculated for $[M+Na]^+$ 381.1825, found 381.18251.

(*Z*)-1-(4-(3,3-dimethyl-1-(naphthalen-1-yl)but-1-en-1-yl)phenyl)ethan-1-one (37)

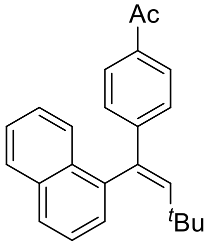

According to the general procedure A, the title compound was isolated as viscous oil after flash chromatography on silica gel (R_f = 0.42, eluent: 50:1 hexane: EtOAc) in 80% yield (52.5 mg). ^{1}H NMR (500 MHz, $CDCl_3$) δ 7.88 (d, *J* = 8.2 Hz, 2H), 7.82 (d, *J* = 8.4 Hz, 2H), 7.79 (d, *J* = 8.4 Hz, 1H), 7.54 (t, *J* = 7.6 Hz, 1H), 7.47 (t, *J* = 7.4 Hz, 1H), 7.41 (d, *J* = 7.0 Hz, 2H), 7.35 (d, *J* = 8.4 Hz, 2H), 6.53 (s, 1H), 2.54 (s, 3H), 0.93 (s, 9H). ^{13}C NMR (126 MHz, $CDCl_3$) δ 197.7, 148.2, 144.0, 137.2, 136.0, 135.3, 133.7, 132.7, 128.4, 128.3, 128.1, 127.9, 126.5, 126.1, 125.8, 125.1, 34.6, 30.6, 26.6. HRMS (ESI) for $C_{24}H_{24}O$: calculated for $[M+Na]^+$ 351.17194, found 351.17195.

(*Z*)-1-(4-(3,3-dimethyl-1-(phenanthren-9-yl)but-1-en-1-yl)phenyl)ethan-1-one (38)

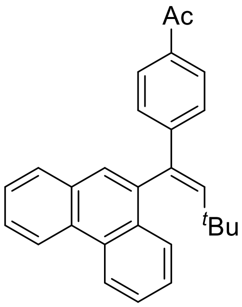

According to the general procedure A, the title compound was isolated as viscous oil after flash chromatography on silica gel (R_f = 0.40, eluent: 50:1 hexane: EtOAc) in 97% yield (73.3 mg). ^{1}H NMR (500 MHz, $CDCl_3$) δ 8.78 – 8.75 (m, 2H), 7.95 (d, J = 7.7 Hz, 1H), 7.87 (d, J = 8.2 Hz, 1H), 7.82 (d, J = 8.4 Hz, 2H), 7.75 – 7.71 (m, 2H), 7.70 – 7.63 (m, 2H), 7.52 (t, J = 7.5 Hz, 1H), 7.44 (d, J = 8.4 Hz, 2H), 6.60 (s, 1H), 2.54 (s, 3H), 1.00 (s, 9H). ^{13}C NMR (126 MHz, $CDCl_3$) δ 197.7, 147.9, 144.3, 136.2, 135.7, 135.5, 131.8, 131.3, 130.5, 130.2, 128.8, 128.7, 128.5, 127.3, 126.9, 126.8, 126.6, 126.5, 122.9, 122.7, 34.7, 30.7, 26.6.

(*Z*)-1-(4-(3,3-dimethyl-1-(thiophen-3-yl)but-1-en-1-yl)phenyl)ethan-1-one (39)

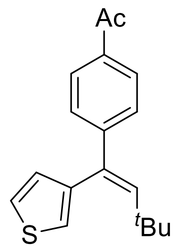

According to the general procedure A, the title compound was isolated as viscous oil after flash chromatography on silica gel (R_f = 0.45, eluent: 50:1 hexane: EtOAc) in 46% yield (26.2 mg). ^{1}H NMR (500 MHz, $CDCl_3$) δ 7.85 (d, J = 7.6 Hz, 2H), 7.37 – 7.33 (m, 1H), 7.29 (d, J = 8.2 Hz, 2H), 7.10 (d, J = 2.8 Hz, 1H), 6.90 (d, J = 4.9 Hz, 1H), 6.28 (s, 1H), 2.58 (s, 3H), 1.03 (s, 9H). ^{13}C NMR (126 MHz, $CDCl_3$) δ 197.7, 148.2, 144.0, 139.4, 135.3, 133.4, 129.8, 128.3, 126.6, 125.1, 123.9, 34.1, 30.9, 26.6. HRMS (ESI) for $C_{18}H_{20}OS$: calculated for $[M+Na]^+$ 307.11271, found 307.11256.

(*E*)-1-(4-(1-(cyclohex-1-en-1-yl)-3,3-dimethylbut-1-en-1-yl)phenyl)ethan-1-one (40)

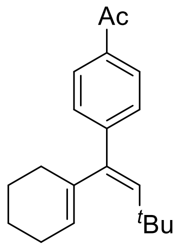

According to the general procedure A, the title compound was isolated as viscous oil after flash chromatography on silica gel (R_f = 0.60, eluent: 50:1 hexane: EtOAc) in 63% yield (35.5 mg). ^{1}H NMR (500 MHz, $CDCl_3$) δ 7.89 (d, J = 8.3 Hz, 2H), 7.47 – 7.44 (m, 2H), 5.82 (s, 1H), 5.71 (s, 1H), 2.60 (s, 3H), 2.23 (s, 2H), 1.90 (s, 2H), 1.68 – 1.64 (m, 4H), 1.20 (s, 9H). ^{13}C NMR (126 MHz, $CDCl_3$) δ 197.8, 147.6, 141.3, 140.8, 136.0, 135.4, 128.3, 127.8, 126.7, 34.2, 31.1, 29.1, 26.6, 25.4, 22.8, 22.0. HRMS (ESI) for $C_{20}H_{26}O$: calculated for $[M+Na]^+$ 305.18759, found 305.18756.

(*Z*)-5-(4-acetylphenyl)-3,3-dimethyl-5-((8*R*,9*S*,13*S*,14*S*)-13-methyl-17-oxo-7,8,9,11,12,13,14,15,16,17-decahydro-6*H*-cyclopenta[a]phenanthren-3-yl)pent-4-en-1-yl 4-methoxybenzoate (41)

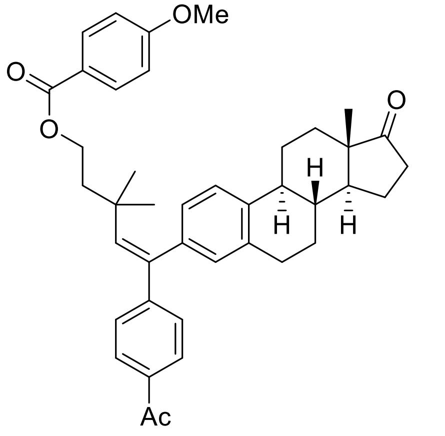

According to the general procedure A, the title compound was isolated as viscous oil after flash chromatography on silica gel (R_f = 0.32, eluent: 5:1 hexane: EtOAc) in 58% yield (71.7 mg). ^{1}H NMR (400 MHz, $CDCl_3$) δ 7.93 (d, *J* = 8.9 Hz, 2H), 7.81 (d, *J* = 8.5 Hz, 2H), 7.26 (dd, *J* = 13.6, 8.1 Hz, 3H), 6.97 (d, *J* = 8.0 Hz, 1H), 6.89 (s, 1H), 6.84 (d, *J* = 8.9 Hz, 2H), 6.16 (s, 1H), 4.38 (t, *J* = 7.1 Hz, 2H), 3.84 (s, 3H), 2.91 – 2.84 (m, 2H), 2.57 (s, 3H), 2.55 – 2.49 (m, 1H), 2.46 – 2.40 (m, 1H), 2.32 (t, *J* = 8.7 Hz, 1H), 2.20 – 1.97 (m, 4H), 1.82 (t, *J* = 7.1 Hz, 2H), 1.67 – 1.45 (m, 6H), 1.05 (s, 6H), 0.95 (s, 3H). ^{13}C NMR (101 MHz, $CDCl_3$) δ 220.9, 197.7, 166.4, 163.3, 148.7, 140.0, 139.7, 138.6, 136.9, 136.1, 135.2, 131.5, 130.3, 128.2, 127.5, 127.0, 124.8, 122.8, 113.5, 62.4, 55.4, 50.6, 48.0, 44.3, 42.3, 38.1, 36.5, 35.9, 31.6, 29.5, 29.3, 26.6, 26.5, 25.6, 21.6, 13.9. HRMS (ESI) for $C_{41}H_{46}O_5$: calculated for $[M+Na]^+$ 641.32375, found 641.32368.

(*E*)-4-(3,3-dimethyl-1,4-diphenylbut-1-en-1-yl)benzonitrile (42)

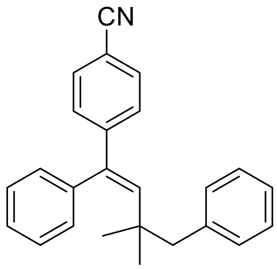

According to the general procedure A, the title compound was isolated as viscous oil after flash chromatography on silica gel (R_f = 0.65, eluent: 50:1 hexane: EtOAc) in 66% yield (44.7 mg). ^{1}H NMR (400 MHz, $CDCl_3$) δ 7.54 (d, *J* = 8.3 Hz, 2H), 7.35 – 7.25 (m, 8H), 7.15 (d, *J* = 7.5 Hz, 2H), 6.88 – 6.81 (m, 2H), 6.17 (s, 1H), 2.71 (s, 2H), 0.93 (s, 6H). ^{13}C NMR (101 MHz, $CDCl_3$) δ 148.4, 141.4, 139.1, 138.7, 138.4, 131.9, 130.6, 130.0, 127.9, 127.8, 127.3,

127.2, 126.2, 119.2, 110.0, 50.5, 39.1, 28.6. HRMS (ESI) for $C_{25}H_{23}N$: calculated for $[M+Na]^+$ 360.17227, found 360.17211.

(*E*)-4-(3,3-dimethyl-1,5-diphenylpent-1-en-1-yl)benzonitrile (43)

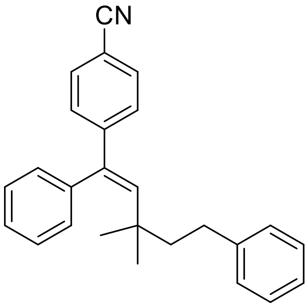

According to the general procedure A, the title compound was isolated as viscous oil after flash chromatography on silica gel (R_f = 0.65, eluent: 50:1 hexane: EtOAc) in 60% yield (33.4 mg). ^1H NMR (400 MHz, $CDCl_3$) δ 7.54 (d, *J* = 8.4 Hz, 2H), 7.42 – 7.36 (m, 3H), 7.28 (t, *J* = 6.7 Hz, 4H), 7.23 – 7.15 (m, 5H), 6.18 (s, 1H), 2.69 – 2.61 (m, 2H), 1.71 – 1.65 (m, 2H), 1.00 (s, 6H). ^{13}C NMR (101 MHz, $CDCl_3$) δ 148.5, 142.8, 141.9, 139.3, 139.0, 131.9, 130.2, 128.4, 128.3, 128.2, 127.5, 127.4, 125.7, 119.1, 110.0, 46.7, 37.6, 31.6, 28.8. HRMS (ESI) for $C_{20}H_{22}O$: calculated for $[M+Na]^+$ 301.15629, found 301.15542. HRMS (ESI) for $C_{26}H_{25}N$: calculated for $[M+Na]^+$ 374.18792, found 374.18831.

(*E*)-4-(3,3-dimethyl-5-phenoxy-1-phenylpent-1-en-1-yl)benzonitrile (44)

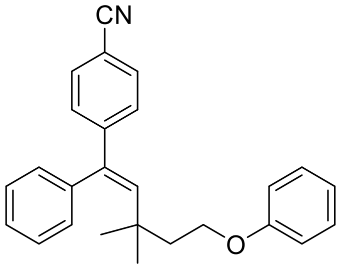

According to the general procedure A, the title compound was isolated as viscous oil after flash chromatography on silica gel (R_f = 0.55, eluent: 50:1 hexane: EtOAc) in 63% yield (46.2 mg). ^1H NMR (400 MHz, $CDCl_3$) δ 7.54 (d, *J* = 8.4 Hz, 2H), 7.41 – 7.37 (m, 3H), 7.32 (t, *J* = 7.6 Hz, 4H), 7.18 (dd, *J* = 6.8, 2.3 Hz, 2H), 6.98 (t, *J* = 7.3 Hz, 1H), 6.90 (d, *J* = 8.2 Hz, 2H), 6.26 (s, 1H), 4.07 (t, *J* = 6.9 Hz, 2H), 1.90 (t, *J* = 6.9 Hz, 2H), 1.03 (s, 6H). ^{13}C NMR (101 MHz, $CDCl_3$) δ 158.9, 148.3, 141.4, 139.1, 138.9, 131.9, 130.1, 129.5, 128.3, 127.5, 127.4, 120.7, 119.1, 114.5, 110.1, 65.1, 43.3, 36.6, 29.2. HRMS (ESI) for $C_{26}H_{25}NO$: calculated for $[M+Na]^+$ 390.18284, found 390.18285.

(*E*)-4-(5-(1,3-dioxoisoindolin-2-yl)-3,3-dimethyl-1-phenylpent-1-en-1-yl)benzonitrile (45)

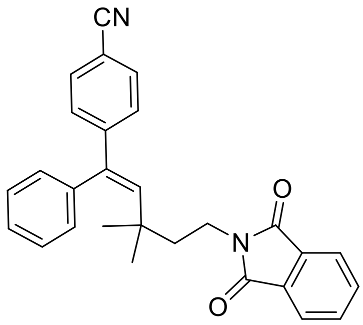

According to the general procedure A, the title compound was isolated as white solid after flash chromatography on silica gel (R_f = 0.40, eluent: 10:1 hexane: EtOAc) in 80% yield (67.5 mg). ^{1}H NMR (400 MHz, $CDCl_3$) δ 7.81 (dd, *J* = 5.4, 3.1 Hz, 2H), 7.69 (dd, *J* = 5.5, 3.0 Hz, 2H), 7.48 (d, *J* = 8.5 Hz, 2H), 7.42 – 7.34 (m, 3H), 7.28 (d, *J* = 8.5 Hz, 2H), 7.23 – 7.19 (m, 2H), 6.21 (s, 1H), 3.79 – 3.74 (m, 2H), 1.81 – 1.75 (m, 2H), 0.97 (s, 6H). ^{13}C NMR (101 MHz, $CDCl_3$) δ 168.3, 148.0, 140.6, 139.8, 139.0, 133.9, 132.1, 131.9, 130.1, 128.2, 127.5, 127.3, 123.1, 119.1, 110.0, 43.2, 36.8, 34.9, 28.5. HRMS (ESI) for $C_{28}H_{24}N_2O_2$: calculated for $[M+Na]^+$ 443.17300, found 443.17273.

(*E*)-5-(4-cyanophenyl)-3,3-dimethyl-5-phenylpent-4-en-1-yl 4-methoxybenzoate (46)

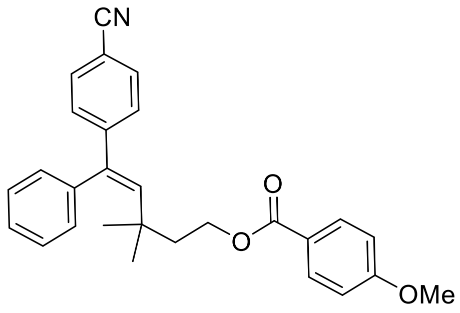

According to the general procedure A, the title compound was isolated as viscous oil after flash chromatography on silica gel (R_f = 0.40, eluent: 30:1 hexane: EtOAc) in 81% yield (68.9 mg). ^{1}H NMR (400 MHz, $CDCl_3$) δ 7.94 – 7.90 (m, 2H), 7.49 (d, *J* = 8.4 Hz, 2H), 7.40 – 7.33 (m, 3H), 7.26 (d, *J* = 8.4 Hz, 2H), 7.21 – 7.17 (m, 2H), 6.84 (d, *J* = 8.8 Hz, 2H), 6.22 (s, 1H), 4.39 (t, *J* = 6.9 Hz, 2H), 3.87 (s, 3H), 1.85 (t, *J* = 6.9 Hz, 2H), 1.01 (s, 6H). ^{13}C NMR (101 MHz, $CDCl_3$) δ 166.4, 163.3, 148.1, 141.1, 139.0, 131.9, 131.5, 130.1, 128.3, 127.5, 127.4, 122.6, 119.1, 113.5, 110.0, 62.2, 55.4, 42.7, 36.6, 29.2. HRMS (ESI) for $C_{28}H_{27}NO_3$: calculated for $[M+Na]^+$ 448.18831, found 448.18831.

(*E*)-5-(4-acetylphenyl)-3,3-dimethyl-5-phenylpent-4-en-1-yl 4-methoxybenzoate (47)

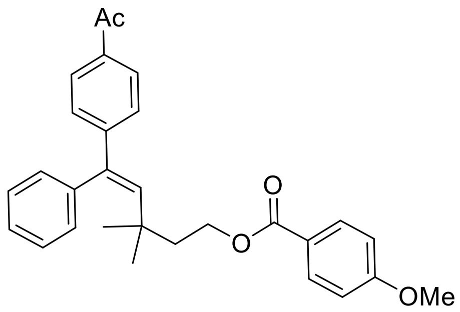

According to the general procedure A, the title compound was isolated as viscous oil after flash chromatography on silica gel (R_f = 0.40, eluent: 10:1 hexane: EtOAc) in 76% yield (67.1 mg). ^{1}H NMR (400 MHz, $CDCl_3$) δ 7.94 (d, *J* = 8.8 Hz, 2H), 7.81 (d, *J* = 8.4 Hz, 2H), 7.35 (t, *J* = 7.4 Hz, 3H), 7.26 (d, *J* = 8.4 Hz, 2H), 7.21 (d, *J* = 7.5 Hz, 2H), 6.83 (d, *J* = 8.8 Hz, 2H), 6.24 (s, 1H), 4.40 (t, *J* = 7.0 Hz, 2H), 3.84 (s, 3H), 2.57 (s, 3H), 1.85 (t, *J* = 7.0 Hz, 2H), 1.03 (s, 6H). ^{13}C NMR (101 MHz, $CDCl_3$) δ 197.7, 166.4, 163.3, 148.4, 140.2, 139.6, 139.5, 135.3, 131.5, 130.1, 128.2, 128.1, 127.3, 126.9, 122.7, 113.5, 62.3, 55.4, 42.7, 36.5, 29.3, 26.6. HRMS (ESI) for $C_{29}H_{30}O_4$: calculated for $[M+Na]^+$ 465.20363, found 465.20368.

(*E*)-5-(4-benzoylphenyl)-3,3-dimethyl-5-phenylpent-4-en-1-yl 4-methoxybenzoate (48)

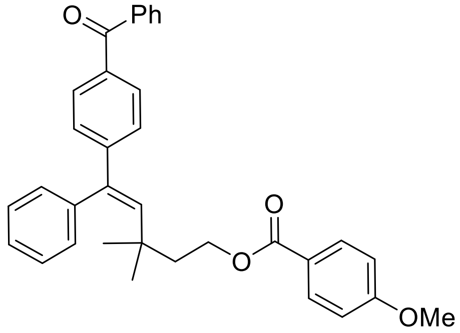

According to the general procedure A, the title compound was isolated as viscous oil after flash chromatography on silica gel (R_f = 0.35, eluent: 10:1 hexane: EtOAc) in 50% yield (50.4 mg). ^{1}H NMR (400 MHz, $CDCl_3$) δ 7.95 (d, *J* = 8.8 Hz, 2H), 7.79 (d, *J* = 7.3 Hz, 2H), 7.69 (d, *J* = 8.3 Hz, 2H), 7.59 (t, *J* = 7.4 Hz, 1H), 7.48 (t, *J* = 7.6 Hz, 2H), 7.41 – 7.33 (m, 3H), 7.30 (d, *J* = 8.4 Hz, 2H), 7.24 (d, *J* = 6.5 Hz, 2H), 6.85 (d, *J* = 8.8 Hz, 2H), 6.27 (s, 1H), 4.42 (t, *J* = 7.0 Hz, 2H), 3.84 (s, 3H), 1.86 (t, *J* = 7.0 Hz, 2H), 1.04 (s, 6H). ^{13}C NMR (101 MHz, $CDCl_3$) δ 196.2, 166.5, 163.3, 147.8, 140.2, 139.7, 139.6, 137.8, 135.6, 132.3, 131.6, 130.13, 130.09, 130.0, 128.2, 128.1, 127.3, 126.7, 122.7, 113.5, 62.3, 55.4, 42.7, 36.5, 29.3. HRMS (ESI) for $C_{34}H_{32}O_4$: calculated for $[M+Na]^+$ 527.21928, found 527.2193.

(*E*)-5-(4-cyanophenyl)-3,3-dimethyl-5-phenylpent-4-en-1-yl furan-2-carboxylate (49)

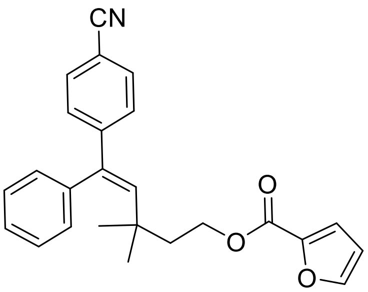

According to the general procedure A, the title compound was isolated as viscous oil after flash chromatography on silica gel (R_f = 0.50, eluent: 10:1 hexane: EtOAc) in 75% yield (57.8 mg). ^{1}H NMR (400 MHz, $CDCl_3$) δ 7.54 – 7.48 (m, 3H), 7.41 – 7.34 (m, 3H), 7.29 – 7.25 (m, 2H), 7.19 (dd, *J* = 7.5, 1.7 Hz, 2H), 7.10 (d, *J* = 3.5 Hz, 1H), 6.48 (s, 1H), 6.20 (s, 1H), 4.40 (t, *J* = 7.1 Hz, 2H), 1.83 (t, *J* = 7.1 Hz, 2H), 1.00 (s, 6H). ^{13}C NMR (101 MHz, $CDCl_3$) δ 158.7, 148.1, 146.3, 144.7, 140.8, 139.3, 139.0, 131.9, 130.0, 128.3, 127.5, 127.4, 119.1, 117.9, 111.8, 110.1, 62.5, 42.6, 36.5, 29.1. HRMS (ESI) for $C_{25}H_{23}NO_3$: calculated for $[M+Na]^+$ 408.15701, found 408.15717.

(*E*)-5-(4-acetylphenyl)-3,3-dimethyl-5-phenylpent-4-en-1-yl methyl terephthalate (50)

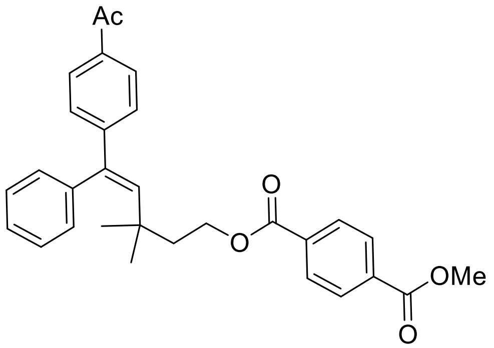

According to the general procedure A, the title compound was isolated as viscous oil after flash chromatography on silica gel (R_f = 0.38, eluent: 10:1 hexane: EtOAc) in 69% yield (64.5 mg). ^{1}H NMR (400 MHz, $CDCl_3$) δ 8.01 (d, *J* = 2.1 Hz, 4H), 7.80 (d, *J* = 8.3 Hz, 2H), 7.36 (q, *J* = 6.8, 6.4 Hz, 3H), 7.24 (d, *J* = 8.3 Hz, 2H), 7.21 (d, *J* = 7.7 Hz, 2H), 6.22 (s, 1H), 4.45 (t, *J* = 7.0 Hz, 2H), 3.95 (s, 3H), 2.57 (s, 3H), 1.87 (t, *J* = 7.0 Hz, 2H), 1.03 (s, 6H). ^{13}C NMR (101 MHz, $CDCl_3$) δ 197.6, 166.2, 165.8, 148.2, 140.0, 139.7, 139.5, 135.3, 134.0, 133.8, 130.0, 129.5, 128.2, 128.1, 127.3, 126.9, 63.0, 52.5, 42.5, 36.5, 29.3, 26.6. HRMS (ESI) for $C_{30}H_{30}O_5$: calculated for $[M+Na]^+$ 493.19854, found 493.19843.

(*E*)-5-(4-cyanophenyl)-3,3-dimethyl-5-phenylpent-4-en-1-yl 4-(*N,N*-dipropylsulfamoyl)benzoate (51)

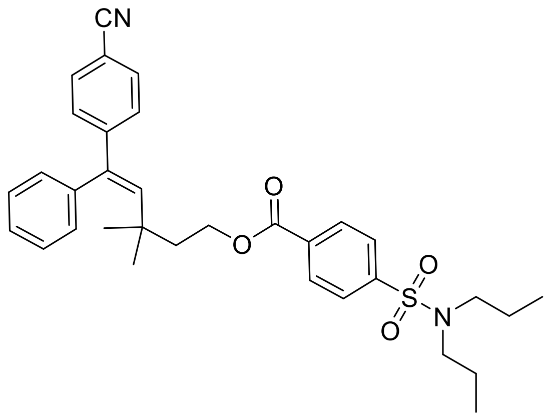

According to the general procedure A, the title compound was isolated as viscous oil after flash chromatography on silica gel (R_f = 0.30, eluent: 10:1 hexane: EtOAc) in 86% yield (96.5 mg). ^{1}H NMR (400 MHz, $CDCl_3$) δ 8.10 (d, *J* = 8.4 Hz, 2H), 7.84 (d, *J* = 8.4 Hz, 2H), 7.52 (d, *J* = 8.4 Hz, 2H), 7.40 – 7.34 (m, 3H), 7.28 (d, *J* = 8.4 Hz, 2H), 7.18 (dd, *J* = 7.3, 1.9 Hz, 2H), 6.19 (s, 1H), 4.43 (t, *J* = 7.3 Hz, 2H), 3.14 – 3.09 (m, 4H), 1.85 (t, *J* = 7.3 Hz, 2H), 1.60 – 1.53 (m, 4H), 1.03 (s, 6H), 0.88 (t, *J* = 7.4 Hz, 6H). ^{13}C NMR (101 MHz, $CDCl_3$) δ 165.3, 148.0, 144.3, 140.6, 139.5, 138.9, 133.5, 131.9, 130.1, 130.0, 128.3, 127.6, 127.4, 127.0, 119.0, 110.3, 63.2, 49.9, 42.2, 36.5, 29.2, 21.9, 11.2. HRMS (ESI) for $C_{33}H_{38}N_2O_4S$: calculated for $[M+Na]^+$ 581.24445, found 581.2453.

(*E*)-4-(2-(4-methyltetrahydro-2*H*-pyran-4-yl)-1-phenylvinyl)benzonitrile (52)

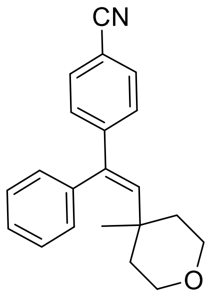

According to the general procedure A, the title compound was isolated as viscous oil after flash chromatography on silica gel (R_f = 0.70, eluent: 10:1 hexane: EtOAc) in 65% yield (39.3 mg). ^{1}H NMR (500 MHz, $CDCl_3$) δ 7.55 (d, *J* = 8.5 Hz, 2H), 7.42 – 7.34 (m, 3H), 7.30 (d, *J* = 8.5 Hz, 2H), 7.17 (dd, *J* = 7.8, 1.5 Hz, 2H), 6.13 (s, 1H), 3.66 – 3.56 (m, 4H), 1.53 (d, *J* = 13.8 Hz, 2H), 1.38 – 1.32 (m, 2H), 1.14 (s, 3H). ^{13}C NMR (126 MHz, $CDCl_3$) δ 148.2, 140.6, 140.3, 139.2, 132.0, 129.8, 128.4, 127.7, 127.4, 119.0, 110.4, 64.7, 39.0, 35.4, 28.3. HRMS (ESI) for $C_{21}H_{21}NO$: calculated for $[M+Na]^+$ 326.15153, found 326.15196.

(*Z*)-4-(3,3-dimethyl-1-phenylbut-1-en-1-yl)benzonitrile (53, 78)

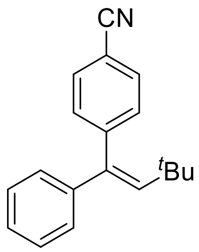

According to the general procedure B or the general procedure C, the title compound was isolated as white solid after flash chromatography on silica gel (R_f = 0.70, eluent: 50:1 hexane: EtOAc) in 90% yield (47.0 mg, P_{syn} : P_{anti} = 92:8) or 60% yield (31.3 mg, P_{syn} : P_{anti} = 93:7). ^{1}H NMR (500 MHz, $CDCl_3$) δ 7.67 (d, J = 8.1 Hz, 2H), 7.36 (d, J = 8.1 Hz, 2H), 7.30 – 7.23 (m, 3H), 7.14 (d, J = 7.1 Hz, 2H), 6.16 (s, 1H), 0.99 (s, 9H). ^{13}C NMR (126 MHz, $CDCl_3$) δ 146.2, 142.9, 141.4, 137.4, 131.7, 131.2, 128.3, 127.1, 126.9, 118.9, 110.9, 34.1, 31.3. HRMS (ESI) for $C_{19}H_{19}N$: calculated for $[M+Na]^+$ 284.14097, found 284.14165.

(*Z*)-5-(4-cyanophenyl)-3,3-dimethyl-5-phenylpent-4-en-1-yl 4-methoxybenzoate (54)

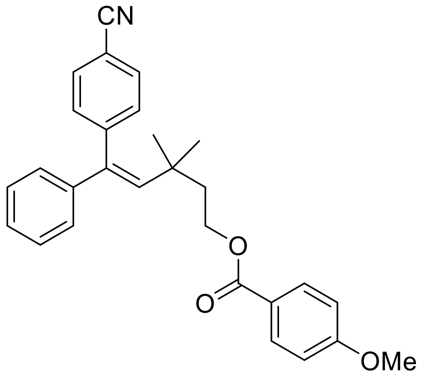

According to the general procedure B, the title compound was isolated as viscous oil after flash chromatography on silica gel (R_f = 0.45, eluent: 20:1 hexane: EtOAc) in 84% yield (71.4 mg, P_{syn} : P_{anti} = 92:8). ^{1}H NMR (400 MHz, $CDCl_3$) δ 7.93 (d, J = 8.8 Hz, 2H), 7.65 (d, J = 8.0 Hz, 2H), 7.36 (d, J = 8.0 Hz, 2H), 7.27 – 7.22 (m, 3H), 7.14 – 7.10 (m, 2H), 6.86 (d, J = 8.8 Hz, 2H), 6.17 (s, 1H), 4.39 (t, J = 7.0 Hz, 2H), 3.86 (s, 3H), 1.84 (t, J = 7.0 Hz, 2H), 1.01 (s, 6H). ^{13}C NMR (101 MHz, $CDCl_3$) δ 166.4, 163.3, 145.9, 142.6, 139.2, 138.7, 131.8, 131.5, 131.0, 128.3, 127.3, 126.9, 122.6, 118.8, 113.6, 111.0, 62.2, 55.4, 42.8, 36.4, 29.5. HRMS (ESI) for $C_{28}H_{27}NO_3$: calculated for $[M+Na]^+$ 448.18831, found 448.18875.

(*Z*)-5-(4-acetylphenyl)-3,3-dimethyl-5-phenylpent-4-en-1-yl 4-methoxybenzoate (55)

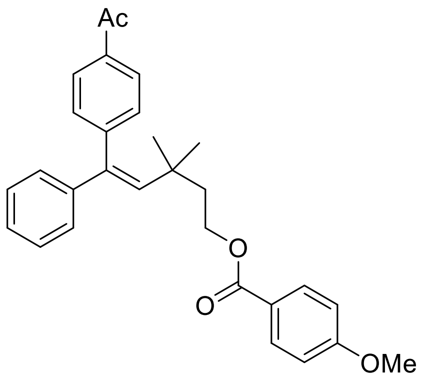

According to the general procedure B, the title compound was isolated as viscous oil after flash chromatography on silica gel (R_f = 0.48, eluent: 10:1 hexane: EtOAc) in 71% yield (63.1 mg, P_{syn} : P_{anti} = 94:6). ^{1}H NMR (400 MHz, $CDCl_3$) δ 7.95 (dd, *J* = 8.4, 4.8 Hz, 4H), 7.35 (d, *J* = 8.1 Hz, 2H), 7.28 – 7.21 (m, 3H), 7.18 – 7.13 (m, 2H), 6.85 (d, *J* = 8.8 Hz, 2H), 6.15 (s, 1H), 4.40 (t, *J* = 7.0 Hz, 2H), 3.86 (s, 3H), 2.62 (s, 3H), 1.84 (t, *J* = 7.0 Hz, 2H), 1.02 (s, 6H). ^{13}C NMR (101 MHz, $CDCl_3$) δ 197.8, 166.4, 163.3, 146.0, 143.0, 139.4, 138.6, 135.8, 131.6, 130.5, 128.2, 128.0, 127.1, 126.9, 122.7, 113.6, 62.3, 55.4, 42.7, 36.4, 29.5, 26.6. HRMS (ESI) for $C_{29}H_{30}O_4$: calculated for $[M+Na]^+$ 465.20363, found 465.20363.

(*Z*)-5-(4-benzoylphenyl)-3,3-dimethyl-5-phenylpent-4-en-1-yl 4-methoxybenzoate (56)

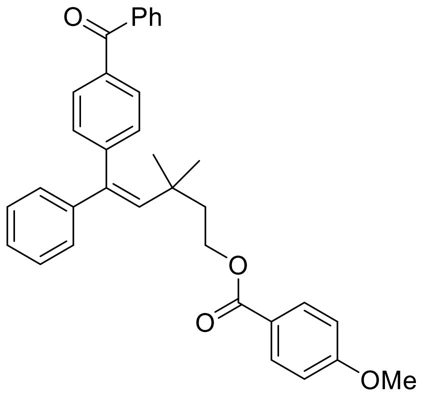

According to the general procedure B, the title compound was isolated as viscous oil after flash chromatography on silica gel (R_f = 0.38, eluent: 10:1 hexane: EtOAc) in 51% yield (51.4 mg, P_{syn} : P_{anti} = 91:9). ^{1}H NMR (400 MHz, $CDCl_3$) δ 7.94 (d, *J* = 8.8 Hz, 2H), 7.83 (dd, *J* = 7.5, 4.7 Hz, 4H), 7.62 (t, *J* = 7.4 Hz, 1H), 7.51 (t, *J* = 7.6 Hz, 2H), 7.37 (d, *J* = 8.0 Hz, 2H), 7.28 – 7.18 (m, 5H), 6.84 (d, *J* = 8.8 Hz, 2H), 6.17 (s, 1H), 4.41 (t, *J* = 7.1 Hz, 2H), 3.85 (s, 3H), 1.86 (t, *J* = 7.1 Hz, 2H), 1.05 (s, 6H). ^{13}C NMR (101 MHz, $CDCl_3$) δ 196.5, 166.4, 163.3, 145.4, 143.1, 139.4, 138.7, 137.6, 136.1, 132.4, 131.5, 130.2, 130.0, 129.8, 128.3, 128.2, 127.1, 127.0, 122.7, 113.6, 62.3, 55.4, 42.8, 36.4, 29.5. HRMS (ESI) for $C_{34}H_{32}O_4$: calculated for $[M+Na]^+$ 527.21928, found 527.21917.

(*Z*)-5-(4-(methoxycarbonyl)phenyl)-3,3-dimethyl-5-phenylpent-4-en-1-yl 4-methoxybenzoate (57)

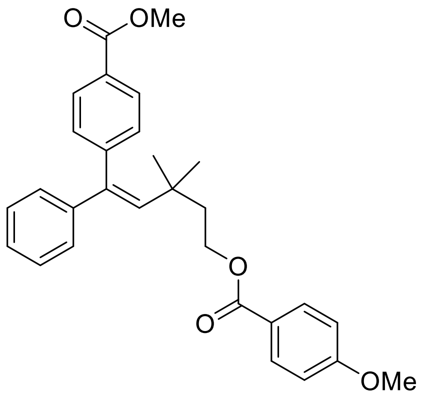

According to the general procedure B, the title compound was isolated as viscous oil after flash chromatography on silica gel (R_f = 0.40, eluent: 20:1 hexane: EtOAc) in 80% yield (73.2 mg, P_{syn} : P_{anti} = 86:14). ^{1}H NMR (500 MHz, $CDCl_3$) δ 8.04 (d, *J* = 8.3 Hz, 2H), 7.94 (d, *J* = 8.9 Hz, 2H), 7.33 (d, *J* = 8.3 Hz, 2H), 7.26 – 7.21 (m, 3H), 7.18 – 7.16 (m, 2H), 6.85 (d, *J* = 9.0 Hz, 2H), 6.15 (s, 1H), 4.40 (t, *J* = 7.1 Hz, 2H), 3.95 (s, 3H), 3.86 (s, 3H), 1.84 (t, *J* = 7.0 Hz, 2H), 1.01 (s, 6H). ^{13}C NMR (126 MHz, $CDCl_3$) δ 167.0, 166.4, 163.3, 145.7, 143.1, 139.4, 138.5, 131.6, 130.3, 129.2, 128.9, 128.2, 127.0, 126.9, 122.7, 113.6, 62.3, 55.4, 52.1, 42.8, 36.3, 29.4. HRMS (ESI) for $C_{29}H_{30}O_5$: calculated for $[M+Na]^+$ 481.19854, found 481.19844.

(*Z*)-5-(4-formylphenyl)-3,3-dimethyl-5-phenylpent-4-en-1-yl 4-methoxybenzoate (58)

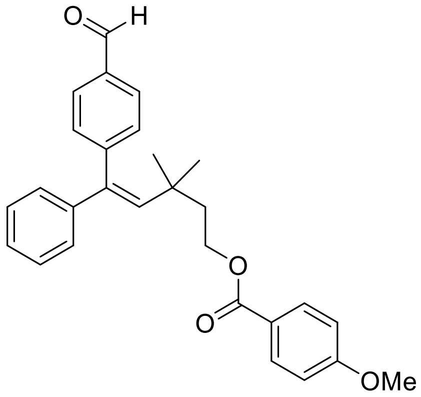

According to the general procedure B, the title compound was isolated as viscous oil after flash chromatography on silica gel (R_f = 0.38, eluent: 20:1 hexane: EtOAc) in 30% yield (25.8 mg, P_{syn} : P_{anti} = 96:4). ^{1}H NMR (500 MHz, $CDCl_3$) δ 10.04 (s, 1H), 7.94 (d, *J* = 8.9 Hz, 2H), 7.88 (d, *J* = 8.1 Hz, 2H), 7.42 (d, *J* = 8.1 Hz, 2H), 7.27 – 7.22 (m, 3H), 7.16 (dd, *J* = 7.9, 1.6 Hz, 2H), 6.85 (d, *J* = 8.9 Hz, 2H), 6.16 (s, 1H), 4.40 (t, *J* = 7.1 Hz, 2H), 3.86 (s, 3H), 1.84 (t, *J* = 7.1 Hz, 2H), 1.02 (s, 6H). ^{13}C NMR (126 MHz, $CDCl_3$) δ 191.9, 166.4, 163.3, 147.5, 142.9, 139.2, 138.8, 135.2, 131.5, 130.9, 129.4, 128.2, 127.2, 126.9, 122.7, 113.6, 62.2, 55.4, 42.8, 36.4, 29.4. HRMS (ESI) for $C_{28}H_{28}O_4$: calculated for $[M+Na]^+$ 451.18798, found 451.18799.

(Z)-3,3-dimethyl-5-(1-oxo-1,3-dihydroisobenzofuran-5-yl)-5-phenylpent-4-en-1-yl 4-methoxybenzo ate (59)

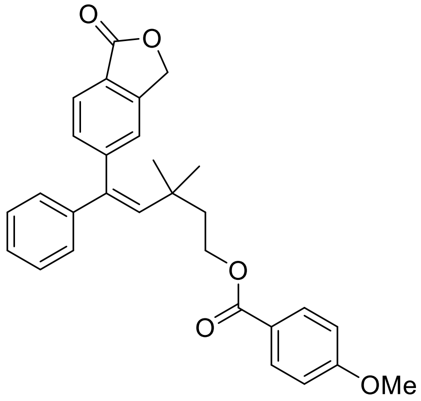

According to the general procedure B, the title compound was isolated as viscous oil after flash chromatography on silica gel (R_f = 0.55, eluent: 7:1 hexane: EtOAc) in 72% yield (65.7 mg, P_{syn} : P_{anti} = 86:14). ^{1}H NMR (400 MHz, $CDCl_3$) δ 7.92 (d, J = 8.7 Hz, 3H), 7.44 (d, J = 7.8 Hz, 1H), 7.35 (s, 1H), 7.27 – 7.20 (m, 3H), 7.16 – 7.11 (m, 2H), 6.85 (d, J = 8.8 Hz, 2H), 6.17 (s, 1H), 5.27 (s, 2H), 4.40 (t, J = 7.0 Hz, 2H), 3.86 (s, 3H), 1.84 (t, J = 7.0 Hz, 2H), 1.02 (s, 6H). ^{13}C NMR (101 MHz, $CDCl_3$) δ 170.9, 166.4, 163.4, 147.3, 146.5, 142.8, 139.1, 139.0, 131.5, 131.4, 128.3, 127.3, 126.9, 125.2, 124.6, 123.6, 122.5, 113.6, 69.5, 62.2, 55.4, 42.6, 36.5, 29.6. HRMS (ESI) for $C_{29}H_{28}O_5$: calculated for $[M+Na]^+$ 479.18289, found 479.18266.

(Z)-3,3-dimethyl-5-phenyl-5-(quinolin-3-yl)pent-4-en-1-yl 4-methoxybenzoate (60)

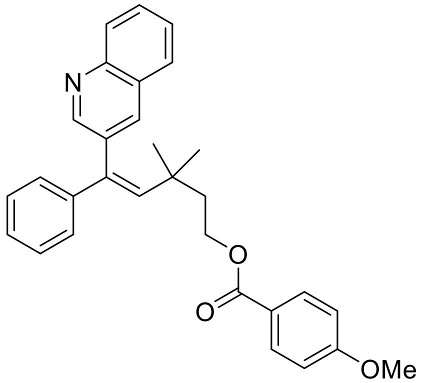

According to the general procedure B, the title compound was isolated as viscous oil after flash chromatography on silica gel (R_f = 0.40, eluent: 10:1 hexane: EtOAc) in 60% yield (54.2 mg, P_{syn} : P_{anti} = 86:14). ^{1}H NMR (500 MHz, $CDCl_3$) δ 8.83 – 8.79 (m, 1H), 8.16 (d, J = 8.4 Hz, 1H), 8.03 (d, J = 1.9 Hz, 1H), 7.91 (d, J = 8.9 Hz, 2H), 7.80 (d, J = 7.9 Hz, 1H), 7.74 (t, J = 8.4 Hz, 1H), 7.56 (t, J = 7.5 Hz, 1H), 7.25 (t, J = 7.3 Hz, 2H), 7.22 – 7.19 (m, 2H), 6.85 – 6.80 (m, 2H), 6.35 (s, 1H), 4.44 (t, J = 7.0 Hz, 2H), 3.85 (s, 3H), 1.87 (t, J = 7.0 Hz, 2H), 1.03 (s, 6H). ^{13}C NMR (126 MHz, $CDCl_3$) δ 166.4, 163.3, 152.0, 147.1, 143.1, 140.6, 136.8, 136.3, 133.7, 131.6, 129.5, 129.3, 128.3, 127.9,

127.5, 127.2, 127.1, 126.9, 122.7, 113.6, 62.2, 55.4, 43.0, 36.5, 29.8. HRMS (ESI) for $C_{30}H_{29}NO_3$: calculated for $[M+Na]^+$ 474.20396, found 474.20401.

(*Z*)-3,3-dimethyl-5-phenyl-5-(4-((((5R,5aS,8aS,8bR)-2,2,7,7-tetramethyltetrahydro-5H-bis([1,3]dioxolo)[4,5-b:4',5'-d]pyran-5-yl)methoxy)carbonyl)phenyl)pent-4-en-1-yl 4-methoxybenzoate (61)

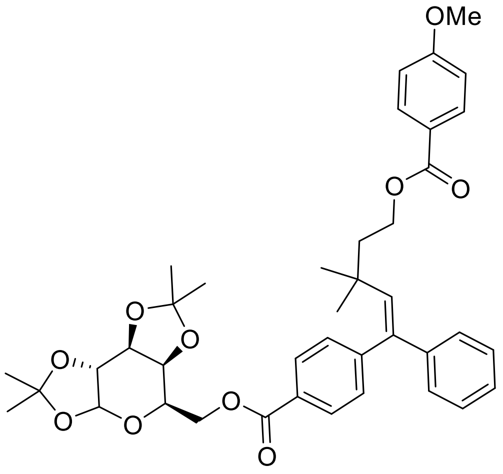

According to the general procedure B, the title compound was isolated as viscous oil after flash chromatography on silica gel (R_f = 0.60, eluent: 10:1 hexane: EtOAc) in 60% yield (82.0 mg, P_{syn} : P_{anti} = 83:17). ^{1}H NMR (400 MHz, $CDCl_3$) δ 8.05 (d, *J* = 8.2 Hz, 2H), 7.94 (d, *J* = 8.9 Hz, 2H), 7.32 (d, *J* = 8.2 Hz, 2H), 7.23 (t, *J* = 7.2 Hz, 3H), 7.16 (dd, *J* = 7.8, 1.7 Hz, 2H), 6.85 (d, *J* = 8.9 Hz, 2H), 6.15 (s, 1H), 5.60 (d, *J* = 5.0 Hz, 1H), 4.69 (dd, *J* = 7.9, 2.4 Hz, 1H), 4.56 – 4.47 (m, 2H), 4.42 – 4.37 (m, 4H), 4.23 (t, *J* = 5.4 Hz, 1H), 3.86 (s, 3H), 1.84 (t, *J* = 7.0 Hz, 2H), 1.53 (d, *J* = 18.6 Hz, 6H), 1.38 (d, *J* = 10.3 Hz, 6H), 1.01 (s, 6H). ^{13}C NMR (101 MHz, $CDCl_3$) δ 166.4, 166.3, 163.3, 145.9, 143.0, 139.4, 138.5, 131.6, 130.3, 129.4, 128.7, 128.2, 127.0, 126.9, 122.7, 113.5, 109.7, 108.8, 96.4, 71.2, 70.7, 70.5, 66.1, 63.9, 62.3, 55.4, 42.9, 36.3, 29.4, 26.1, 26.0, 25.0, 24.5. HRMS (ESI) for $C_{40}H_{46}O_{10}$: calculated for $[M+Na]^+$ 709.29832, found 709.29829.

(*E*)-5-(4-acetylphenyl)-5-(4-methoxyphenyl)-3,3-dimethylpent-4-en-1-yl 4-methoxybenzoate (62)

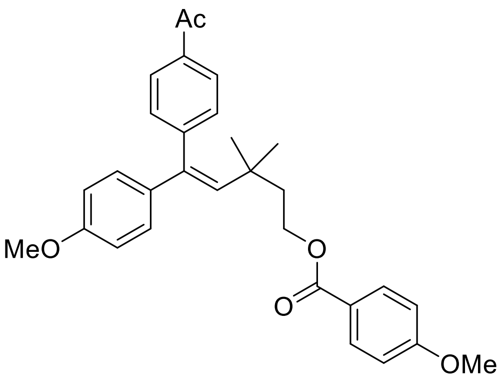

According to the general procedure B, the title compound was isolated as viscous oil after flash chromatography on silica gel (R_f = 0.55, eluent: 10:1 hexane: EtOAc) in 76% yield (72.1 mg, P_{syn} : P_{anti} = 91:9). ^{1}H NMR (400 MHz, $CDCl_3$) δ 7.96 – 7.92 (m, 4H), 7.33 (d, *J* = 8.2 Hz, 2H), 7.07 (d, *J* = 8.8 Hz, 2H), 6.86 (d, *J* = 8.9 Hz, 2H), 6.78 (d, *J* = 8.8 Hz, 2H), 6.05 (s, 1H), 4.39 (t, *J* = 7.1 Hz, 2H), 3.86 (s, 3H), 3.78 (s, 3H), 2.61 (s, 3H), 1.82 (t, *J* = 7.1 Hz, 2H), 1.00 (s, 6H). ^{13}C NMR (101 MHz, $CDCl_3$) δ 197.8, 166.4, 163.3, 158.8, 146.3, 138.8, 136.9, 135.7, 131.6, 130.4, 128.0, 122.7, 113.6, 113.5, 62.3, 55.4, 55.3, 42.7, 36.2, 29.6, 26.6. HRMS (ESI) for $C_{30}H_{32}O_5$: calculated for $[M+Na]^+$ 495.21419, found 495.21419.

(*Z*)-5-(4-acetylphenyl)-5-(4-ethylphenyl)-3,3-dimethylpent-4-en-1-yl 4-methoxybenzoate (63)

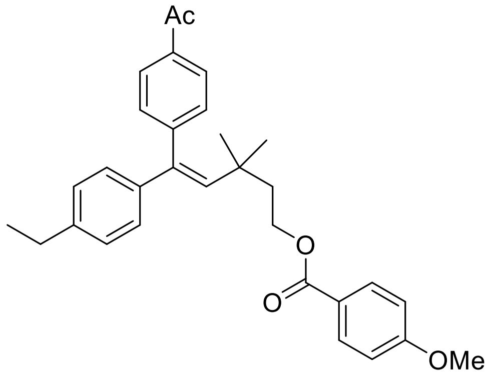

According to the general procedure B, the title compound was isolated as viscous oil after flash chromatography on silica gel (R_f = 0.62, eluent: 10:1 hexane: EtOAc) in 64% yield (60.2 mg, P_{syn} : P_{anti} = 94:6). ^{1}H NMR (400 MHz, $CDCl_3$) δ 7.97 – 7.93 (m, 4H), 7.34 (d, *J* = 8.2 Hz, 2H), 7.08 (s, 4H), 6.86 (d, *J* = 8.9 Hz, 2H), 6.12 (s, 1H), 4.39 (t, *J* = 7.1 Hz, 2H), 3.86 (s, 3H), 2.62 (s, 3H), 1.82 (t, *J* = 7.1 Hz, 2H), 1.28 – 1.15 (m, 5H), 1.01 (s, 6H). ^{13}C NMR (101 MHz, $CDCl_3$) δ 197.9, 166.4, 163.3, 146.2, 143.3, 140.4, 139.2, 137.7, 135.7, 131.6, 130.4, 128.0, 127.7, 126.8, 122.7, 113.6, 62.4, 55.4, 42.7, 36.3, 29.5, 28.4, 26.6, 15.6. HRMS (ESI) for $C_{31}H_{34}O_4$: calculated for $[M+Na]^+$ 493.23493, found 493.23527.

(*E*)-5-(4-acetylphenyl)-3,3-dimethyl-5-(m-tolyl)pent-4-en-1-yl 4-methoxybenzoate (64)

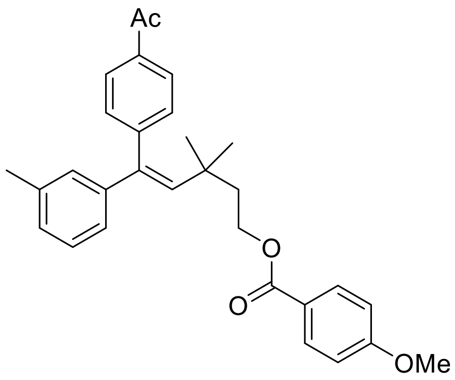

According to the general procedure B, the title compound was isolated as viscous oil after flash chromatography on silica gel (R_f = 0.60, eluent: 10:1 hexane: EtOAc) in 72% yield (65.4 mg, P_{syn} : P_{anti} = 94:6). ^1H NMR (400 MHz, $CDCl_3$) δ 7.97 – 7.93 (m, 4H), 7.34 (d, *J* = 8.1 Hz, 2H), 7.14 (t, *J* = 7.6 Hz, 1H), 7.04 (d, *J* = 7.4 Hz, 1H), 6.99 – 6.94 (m, 2H), 6.86 (d, *J* = 8.8 Hz, 2H), 6.12 (s, 1H), 4.40 (t, *J* = 7.0 Hz, 2H), 3.86 (s, 3H), 2.62 (s, 3H), 2.29 (s, 3H), 1.83 (t, *J* = 7.6 Hz, 2H), 1.02 (s, 6H). ^{13}C NMR (101 MHz, $CDCl_3$) δ 197.9, 166.4, 163.3, 146.1, 143.1, 139.5, 138.4, 137.7, 135.8, 131.6, 130.4, 128.1, 128.0, 127.9, 127.5, 124.2, 122.7, 113.6, 62.3, 55.4, 42.6, 36.3, 29.5, 26.6, 21.5. HRMS (ESI) for $C_{30}H_{32}O_4$: calculated for $[M+Na]^+$ 479.21928, found 479.22020.

(*E*)-5-(4-acetylphenyl)-3,3-dimethyl-5-(o-tolyl)pent-4-en-1-yl 4-methoxybenzoate (65)

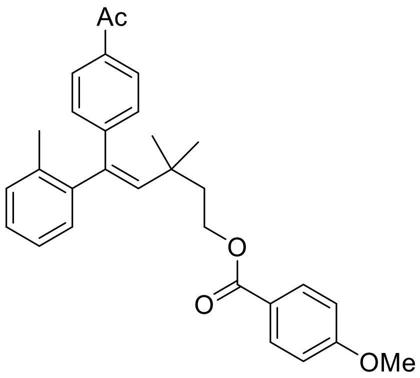

According to the general procedure B, the title compound was isolated as viscous oil after flash chromatography on silica gel (R_f = 0.60, eluent: 10:1 hexane: EtOAc) in 67% yield (61.1 mg, P_{syn} : P_{anti} > 99:1). ^1H NMR (400 MHz, $CDCl_3$) δ 7.95 (d, *J* = 8.9 Hz, 2H), 7.87 (d, *J* = 8.2 Hz, 2H), 7.39 (d, *J* = 8.2 Hz, 2H), 7.18 – 7.11 (m, 4H), 6.90 (d, *J* = 8.9 Hz, 2H), 5.71 (s, 1H), 4.41 (t, *J* = 7.2 Hz, 2H), 3.87 (s, 3H), 2.56 (s, 3H), 2.33 (s, 3H), 1.86 (t, *J* = 7.2 Hz, 2H), 1.09 (s, 6H). ^{13}C NMR (101 MHz, $CDCl_3$) δ 197.7, 166.4, 163.3, 146.4, 144.2, 141.1, 139.5, 135.5, 135.2, 131.6, 130.6, 129.8, 129.7, 127.8, 127.1, 125.7, 122.7, 113.6, 62.3, 55.4, 42.3, 36.4, 29.6, 26.5, 20.7. HRMS (ESI) for $C_{30}H_{32}O_4$: calculated for $[M+Na]^+$ 479.21928, found 479.21915.

(*E*)-5-(4-acetylphenyl)-3,3-dimethyl-5-(2,4,5-trimethylphenyl)pent-4-en-1-yl 4-methoxybenzoate (66)

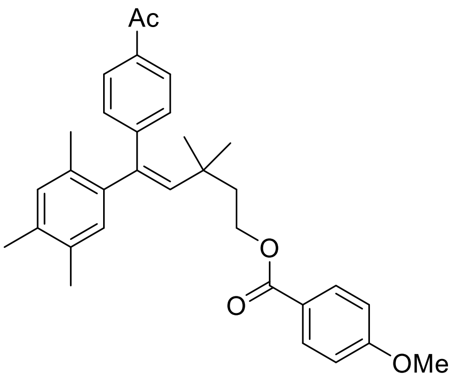

According to the general procedure B, the title compound was isolated as viscous oil after flash chromatography on silica gel (R_f = 0.55, eluent: 10:1 hexane: EtOAc) in 69% yield (67.1 mg, P_{syn} : P_{anti} > 99:1). ^{1}H NMR (400 MHz, $CDCl_3$) δ 7.96 (d, J = 8.8 Hz, 2H), 7.88 (d, J = 8.2 Hz, 2H), 7.39 (d, J = 8.2 Hz, 2H), 6.95 – 6.89 (m, 4H), 5.68 (s, 1H), 4.41 (t, J = 7.2 Hz, 2H), 3.88 (s, 3H), 2.56 (s, 3H), 2.28 (s, 3H), 2.21 (s, 3H), 2.19 (s, 3H), 1.85 (t, J = 7.2 Hz, 2H), 1.09 (s, 6H). ^{13}C NMR (101 MHz, $CDCl_3$) δ 197.8, 166.4, 163.3, 146.8, 141.8, 140.8, 139.3, 135.4, 135.3, 133.6, 132.2, 131.9, 131.6, 130.8, 129.7, 127.8, 122.8, 113.6, 62.4, 55.4, 42.2, 36.4, 29.7, 26.5, 20.1, 19.3, 19.2. HRMS (ESI) for $C_{32}H_{36}O_4$: calculated for $[M+Na]^+$ 507.25058, found 507.25087.

(*E*)-5-(4-acetylphenyl)-5-(2-methoxyphenyl)-3,3-dimethylpent-4-en-1-yl 4-methoxybenzoate (67)

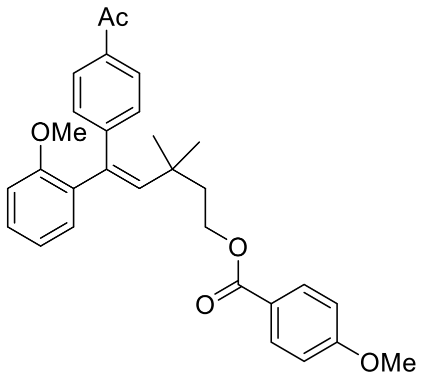

According to the general procedure B, the title compound was isolated as viscous oil after flash chromatography on silica gel (R_f = 0.48, eluent: 10:1 hexane: EtOAc) in 78% yield (73.5 mg, P_{syn} : P_{anti} > 99:1). ^{1}H NMR (400 MHz, $CDCl_3$) δ 7.96 (d, J = 8.9 Hz, 2H), 7.85 (d, J = 8.3 Hz, 2H), 7.41 (d, J = 8.3 Hz, 2H), 7.24 – 7.17 (m, 2H), 6.90 (dd, J = 8.2, 6.2 Hz, 3H), 6.84 – 6.78 (m, 1H), 5.80 (s, 1H), 4.44 (t, J = 7.3 Hz, 2H), 3.87 (s, 3H), 3.67 (s, 3H), 2.56 (s, 3H), 1.81 (t, J = 7.3 Hz, 2H), 1.04 (s, 6H). ^{13}C NMR (101 MHz, $CDCl_3$) δ 197.9, 166.4, 163.3, 156.7, 146.9, 140.7, 137.6, 135.2, 134.0, 131.5, 130.4, 130.0, 128.5, 127.4, 122.9, 120.5, 113.6, 111.3, 62.5, 55.4, 55.3, 42.6, 36.4, 29.6, 26.6. HRMS (ESI) for $C_{30}H_{32}O_5$: calculated for $[M+Na]^+$ 495.21419, found 495.21400.

(*E*)-5-(4-acetylphenyl)-3,3-dimethyl-5-(2-(trifluoromethyl)phenyl)pent-4-en-1-yl 4-methoxybenzoate (68)

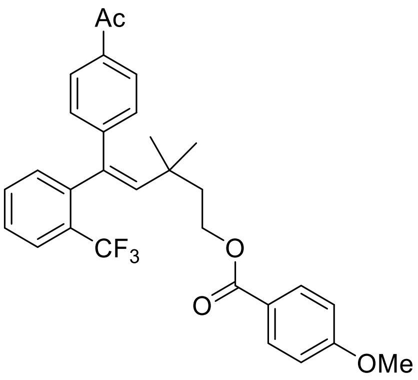

According to the general procedure B, the title compound was isolated as viscous oil after flash chromatography on silica gel (R_f = 0.50, eluent: 10:1 hexane: EtOAc) in 40% yield (40.5 mg, P_{syn} : P_{anti} > 99:1). ^{1}H NMR (400 MHz, $CDCl_3$) δ 7.94 (d, J = 8.8 Hz, 2H), 7.89 (d, J = 8.2 Hz, 2H), 7.68 (d, J = 7.6 Hz, 1H), 7.46 (d, J = 8.2 Hz, 2H), 7.39 (t, J = 7.2 Hz, 1H), 7.33 (t, J = 7.6 Hz, 1H), 7.24 (d, J = 7.6 Hz, 1H), 6.89 (d, J = 8.8 Hz, 2H), 5.78 (s, 1H), 4.39 (t, J = 7.1 Hz, 2H), 3.87 (s, 3H), 2.56 (s, 3H), 1.84 (t, J = 7.2 Hz, 2H), 1.05 (s, 6H). ^{13}C NMR (101 MHz, $CDCl_3$) δ 197.7, 166.4, 163.3, 145.6, 143.0, 142.5, 136.2, 135.8, 131.5, 131.4 (d, J = 25.1 Hz), 131.2, 129.8, 127.9, 127.6, 126.9, 126.6 (q, J = 5.4 Hz), 124.4 (q, J = 273.9 Hz), 122.8, 113.6, 62.1, 55.4, 42.1, 36.6, 29.2, 26.6. ^{19}F NMR (377 MHz, $CDCl_3$) δ -55.65. HRMS (ESI) for $C_{30}H_{29}F_3O_4$: calculated for $[M+Na]^+$ 533.19101, found 533.19111.

(*Z*)-5-(4-acetylphenyl)-5-(cyclohex-1-en-1-yl)-3,3-dimethylpent-4-en-1-yl 4-methoxybenzoate (69)

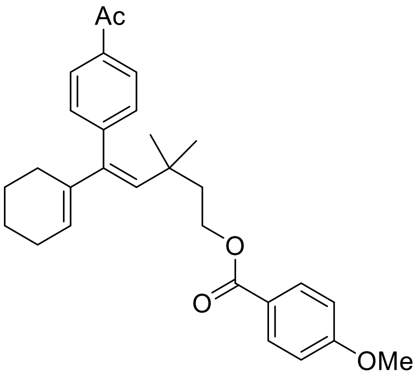

According to the general procedure B, the title compound was isolated as viscous oil after flash chromatography on silica gel (R_f = 0.52, eluent: 10:1 hexane: EtOAc) in 63% yield (56.4 mg, P_{syn} : P_{anti} = 69:31). ^{1}H NMR (400 MHz, $CDCl_3$) δ 7.97 (d, J = 8.8 Hz, 2H), 7.89 (d, J = 8.2 Hz, 2H), 7.23 (d, J = 8.2 Hz, 2H), 6.92 (d, J = 8.9 Hz, 2H), 5.67 (s, 1H), 5.04 (t, J = 4.0 Hz, 1H), 4.32 (t, J = 7.2 Hz, 2H), 3.88 (s, 3H), 2.60 (s, 3H), 2.23 (t, J = 5.3 Hz, 2H), 2.02 – 1.97 (m, 2H), 1.71 – 1.65 (m, 4H), 1.57 – 1.51 (m, 2H), 0.89 (s, 6H). ^{13}C NMR (101 MHz, $CDCl_3$) δ 198.0, 166.5, 163.3, 146.3, 141.0, 138.8, 135.4, 133.4, 131.6, 130.7, 128.2, 127.5, 122.9, 113.6, 62.5, 55.4, 42.7, 35.7, 29.8, 26.6, 26.3, 26.1, 23.0, 22.1. HRMS (ESI) for $C_{29}H_{34}O_4$: calculated for $[M+Na]^+$ 469.23493, found 469.23496.

(*E*)-5-(4-acetylphenyl)-3,3-dimethyl-5-((8*R*,9*S*,13*S*,14*S*)-13-methyl-17-oxo-7,8,9,11,12,13,14,15,16,17-decahydro-6*H*-cyclopenta[a]phenanthren-3-yl)pent-4-en-1-yl 4-methoxybenzoate (70)

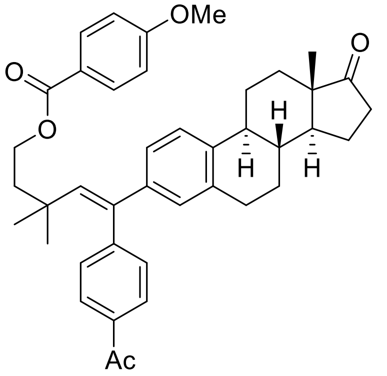

According to the general procedure B, the title compound was isolated as white solid after flash chromatography on silica gel (R_f = 0.35, eluent: 5:1 hexane: EtOAc) in 64% yield (79.1 mg, P_{syn} : P_{anti} = 93:7). ^{1}H NMR (400 MHz, $CDCl_3$) δ 7.94 (d, *J* = 9.0 Hz, 4H), 7.34 (d, *J* = 8.2 Hz, 2H), 7.17 (d, *J* = 8.2 Hz, 1H), 6.95 (d, *J* = 10.0 Hz, 1H), 6.87 (d, *J* = 8.9 Hz, 3H), 6.10 (s, 1H), 4.38 (t, *J* = 7.1 Hz, 2H), 3.86 (s, 3H), 2.86 – 2.80 (m, 2H), 2.62 (s, 3H), 2.52 (dd, *J* = 18.9, 8.5 Hz, 1H), 2.43 – 2.36 (m, 1H), 2.28 (t, *J* = 8.9 Hz, 1H), 2.19 – 1.95 (m, 4H), 1.82 (t, *J* = 7.1 Hz, 2H), 1.65 – 1.41 (m, 6H), 1.00 (s, 6H), 0.92 (s, 3H). ^{13}C NMR (101 MHz, $CDCl_3$) δ 220.9, 197.9, 166.4, 163.3, 146.1, 140.6, 139.1, 138.8, 138.0, 136.3, 135.8, 131.6, 130.4, 128.0, 127.3, 125.2, 124.3, 122.8, 113.6, 62.4, 55.4, 50.5, 48.0, 44.3, 42.6, 38.1, 36.3, 35.9, 31.6, 29.53, 29.48, 26.6, 26.5, 25.7, 21.6, 13.8. HRMS (ESI) for $C_{41}H_{46}O_5$: calculated for $[M+Na]^+$ 641.32375, found 641.32379.

(*Z*)-4-(3,3-dimethyl-1,4-diphenylbut-1-en-1-yl)benzonitrile (71, 93)

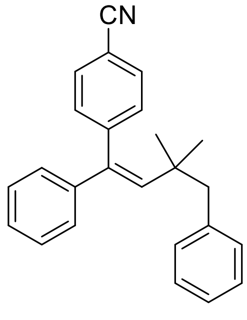

According to the general procedure B or the general procedure D, the title compound was isolated as viscous oil after flash chromatography on silica gel (R_f = 0.65, eluent: 100:1 hexane: EtOAc) in 70% yield (47.2 mg, P_{syn} : P_{anti} = 88:12) or 51% yield (34.5 mg, P_{syn} : P_{anti} = 93:7).^{1}H NMR (400 MHz, $CDCl_3$) δ 7.48 (d, *J* = 8.2 Hz, 2H), 7.33 – 7.30 (m, 3H), 7.28 – 7.22 (m, 3H), 7.14 (dd, *J* = 7.3, 1.8 Hz, 2H), 7.10 (dd, *J* = 8.0, 1.5 Hz, 2H), 6.80 (d, *J* = 8.2 Hz, 2H), 6.09 (s, 1H), 2.68 (s, 2H), 0.90 (s, 6H). ^{13}C NMR (101 MHz, $CDCl_3$) δ 145.9, 142.9, 139.2, 138.8, 138.0,

131.3, 130.8, 130.7, 128.3, 127.9, 127.1, 126.8, 126.2, 119.0, 110.6, 50.5, 39.2, 29.0. HRMS (ESI) for $C_{25}H_{23}N$: calculated for $[M+Na]^+$ 360.17227, found 360.17235.

(*Z*)-4-(3,3-dimethyl-1,5-diphenylpent-1-en-1-yl)benzonitrile (72, 94)

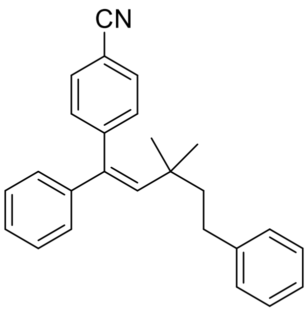

According to the general procedure B or the general procedure D, the title compound was isolated as viscous oil after flash chromatography on silica gel (R_f = 0.65, eluent: 100:1 hexane: EtOAc) in 51% yield (36.0 mg, P_{syn} : P_{anti} = 93:7)) or 57% yield (40.2 mg, P_{syn} : P_{anti} = 91:9). ^{1}H NMR (400 MHz, $CDCl_3$) δ 7.68 (d, *J* = 8.1 Hz, 2H), 7.39 (d, *J* = 8.1 Hz, 2H), 7.35 – 7.28 (m, 5H), 7.23 (d, *J* = 7.3 Hz, 1H), 7.17 (dd, *J* = 9.3, 7.4 Hz, 4H), 6.16 (s, 1H), 2.69 – 2.64 (m, 2H), 1.71 – 1.66 (m, 2H), 0.99 (s, 6H). ^{13}C NMR (101 MHz, $CDCl_3$) δ 146.1, 142.9, 142.7, 140.0, 138.6, 131.7, 131.1, 128.5, 128.34, 128.25, 127.3, 126.9, 125.8, 118.9, 110.9, 47.0, 37.5, 31.6, 29.0. HRMS (ESI) for $C_{26}H_{25}N$: calculated for $[M+Na]^+$ 374.18792, found 374.18786.

(*Z*)-4-(3,3-dimethyl-5-phenoxy-1-phenylpent-1-en-1-yl)benzonitrile (73)

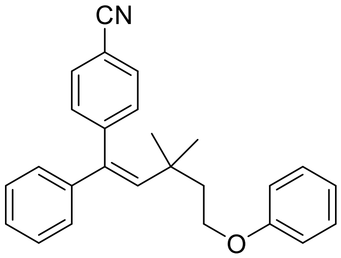

According to the general procedure B, the title compound was isolated as viscous oil after flash chromatography on silica gel (R_f = 0.50, eluent: 50:1 hexane: EtOAc) in 70% yield (51.6 mg, P_{syn} : P_{anti} = 91:9). ^{1}H NMR (400 MHz, $CDCl_3$) δ 7.65 (d, *J* = 8.3 Hz, 2H), 7.34 – 7.27 (m, 7H), 7.16 (dd, *J* = 8.0, 1.6 Hz, 2H), 6.98 (t, *J* = 7.3 Hz, 1H), 6.90 (d, *J* = 7.8 Hz, 2H), 6.18 (s, 1H), 4.08 (t, *J* = 7.0 Hz, 2H), 1.89 (t, *J* = 6.9 Hz, 2H), 1.02 (s, 6H). ^{13}C NMR (101 MHz, $CDCl_3$) δ 158.8, 145.9, 142.7, 139.4, 138.5, 131.8, 131.0, 129.5, 128.3, 127.3, 126.9, 120.7, 118.9, 114.5, 111.0, 65.0, 43.3, 36.5, 29.5. HRMS (ESI) for $C_{26}H_{25}NO$: calculated for $[M+Na]^+$ 390.18284, found 390.18267.

(*Z*)-5-(4-cyanophenyl)-3,3-dimethyl-5-phenylpent-4-en-1-yl furan-2-carboxylate (74, 95)

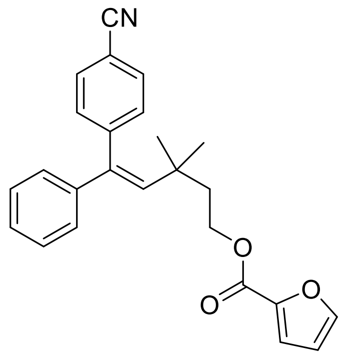

According to the general procedure B or the general procedure D, the title compound was isolated as light-yellow solid after flash chromatography on silica gel (R_f = 0.42, eluent: 10:1 hexane: EtOAc) in 51% yield (39.3 mg, P_{syn} : P_{anti} = 91:9) or 52% yield (40.0 mg, P_{syn} : P_{anti} = 90:10). ^{1}H NMR (400 MHz, $CDCl_3$) δ 7.66 (d, *J* = 8.1 Hz, 2H), 7.55 (s, 1H), 7.36 (d, *J* = 8.1 Hz, 2H), 7.27 – 7.22 (m, 3H), 7.11 (dd, *J* = 7.9, 2.3 Hz, 3H), 6.48 (dd, *J* = 3.4, 1.7 Hz, 1H), 6.13 (s, 1H), 4.40 (t, *J* = 7.2 Hz, 2H), 1.83 (t, *J* = 7.2 Hz, 2H), 0.99 (s, 6H). ^{13}C NMR (101 MHz, $CDCl_3$) δ 158.7, 146.3, 145.8, 144.6, 142.5, 138.9, 138.8, 131.8, 131.0, 128.3, 127.3, 126.8, 118.8, 117.9, 111.9, 111.0, 62.4, 42.7, 36.3, 29.4. HRMS (ESI) for $C_{25}H_{23}NO_3$: calculated for $[M+Na]^+$ 408.15701, found 408.15706.

(*Z*)-5-(4-acetylphenyl)-3,3-dimethyl-5-phenylpent-4-en-1-yl methyl terephthalate (75)

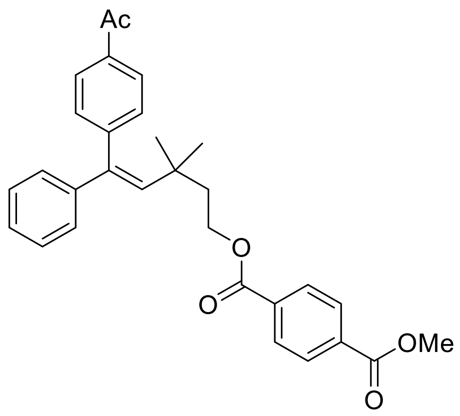

According to the general procedure B, the title compound was isolated as viscous oil after flash chromatography on silica gel (R_f = 0.38, eluent: 10:1 hexane: EtOAc) in 76% yield (71.2 mg, P_{syn} : P_{anti} = 94:6). ^{1}H NMR (400 MHz, $CDCl_3$) δ 8.04 (s, 4H), 7.96 (d, *J* = 8.1 Hz, 2H), 7.34 (d, *J* = 8.1 Hz, 2H), 7.26 – 7.21 (m, 3H), 7.17 – 7.13 (m, 2H), 6.14 (s, 1H), 4.45 (t, *J* = 7.1 Hz, 2H), 3.96 (s, 3H), 2.61 (s, 3H), 1.86 (t, *J* = 7.1 Hz, 2H), 1.02 (s, 6H). ^{13}C NMR (101 MHz, $CDCl_3$) δ 197.7, 166.3, 165.8, 145.9, 142.9, 139.6, 138.3, 135.9, 134.0, 133.8, 130.4, 129.54, 129.49, 128.2, 128.0, 127.2, 126.8, 63.1, 52.5, 42.6, 36.3, 29.5, 26.6. HRMS (ESI) for $C_{30}H_{30}O_5$: calculated for $[M+Na]^+$ 493.19854, found 493.19866.

(*Z*)-5-(4-cyanophenyl)-3,3-dimethyl-5-phenylpent-4-en-1-yl 4-(N,N-dipropylsulfamoyl)benzoate (76, 97)

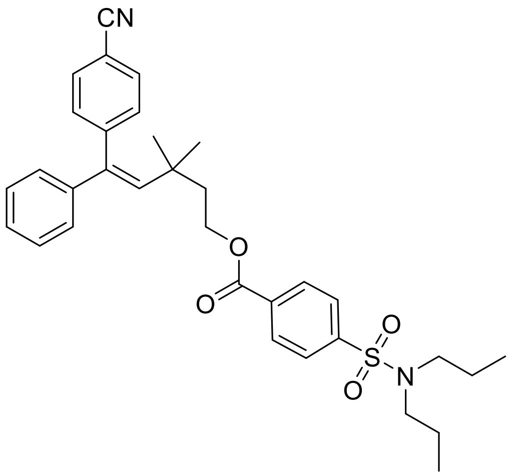

According to the general procedure B or the general procedure D, the title compound was isolated as viscous oil after flash chromatography on silica gel (R_f = 0.32, eluent: 10:1 hexane: EtOAc) in 81% yield (90.1 mg, P_{syn} : P_{anti} = 91:9) or 79% yield (88.3 mg, P_{syn} : P_{anti} = 83:17). ^{1}H NMR (400 MHz, $CDCl_3$) δ 8.08 (d, *J* = 8.5 Hz, 2H), 7.79 (d, *J* = 8.5 Hz, 2H), 7.66 (d, *J* = 8.2 Hz, 2H), 7.36 (d, *J* = 8.2 Hz, 2H), 7.25 (dd, *J* = 5.2, 1.8 Hz, 3H), 7.11 (dd, *J* = 7.3, 2.3 Hz, 2H), 6.16 (s, 1H), 4.45 (t, *J* = 7.1 Hz, 2H), 3.12 – 3.08 (m, 4H), 1.86 (t, *J* = 7.0 Hz, 2H), 1.56 (q, *J* = 7.5 Hz, 4H), 1.01 (s, 6H), 0.88 (t, *J* = 7.4 Hz, 6H). ^{13}C NMR (101 MHz, $CDCl_3$) δ 165.3, 145.8, 144.2, 142.4, 138.9, 138.8, 133.4, 131.8, 131.0, 130.1, 128.3, 127.4, 127.0, 126.8, 118.8, 111.1, 63.1, 50.0, 42.7, 36.4, 29.4, 22.0, 11.2. HRMS (ESI) for $C_{33}H_{38}N_2O_4S$: calculated for $[M+Na]^+$ 581.24445, found 581.24483.

(*Z*)-4-(2-(4-methyltetrahydro-2*H*-pyran-4-yl)-1-phenylvinyl)benzonitrile (77)

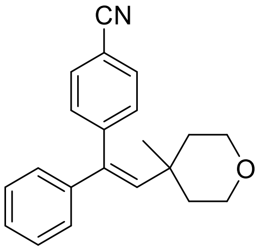

According to the general procedure B, the title compound was isolated as viscous oil after flash chromatography on silica gel (R_f = 0.50, eluent: 50:1 hexane: EtOAc) in 67% yield (40.6 mg, P_{syn} : P_{anti} = 82:18). ^{1}H NMR (500 MHz, $CDCl_3$) δ 7.68 (d, *J* = 8.3 Hz, 2H), 7.34 (d, *J* = 8.2 Hz, 2H), 7.30 – 7.26 (m, 3H), 7.15 (dd, *J* = 8.0, 1.5 Hz, 2H), 6.09 (s, 1H), 3.66 – 3.60 (m, 4H), 1.54 – 1.49 (m, 2H), 1.38 – 1.33 (m, 2H), 1.13 (s, 3H). ^{13}C NMR (126 MHz, $CDCl_3$) δ 146.0, 142.7, 139.8, 138.9, 132.0, 130.8, 128.4, 127.5, 126.9, 118.7, 111.3, 64.5, 39.1, 35.1, 28.3. HRMS (ESI) for $C_{21}H_{21}NO$: calculated for $[M+Na]^+$ 326.15153, found 326.15175.

(*Z*)-1-(4-(3,3-dimethyl-1-phenylbut-1-en-1-yl)phenyl)ethan-1-one (79)

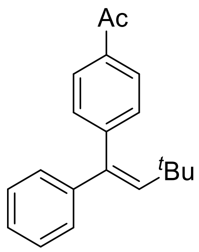

According to the general procedure D, the title compound was isolated as viscous oil after flash chromatography on silica gel (R_f = 0.70, eluent: 50:1 hexane: EtOAc) in 55% yield (30.6 mg, P_{syn} : P_{anti} = 94:6). ^{1}H NMR (400 MHz, $CDCl_3$) δ 7.98 (d, *J* = 8.1 Hz, 2H), 7.34 (d, *J* = 8.1 Hz, 2H), 7.27 – 7.16 (m, 5H), 6.15 (s, 1H), 2.66 (s, 3H), 1.00 (s, 9H). ^{13}C NMR (101 MHz, $CDCl_3$) δ 197.9, 146.4, 143.3, 140.8, 138.1, 135.7, 130.7, 128.2, 127.9, 126.9, 126.9, 34.1, 31.3, 26.7. HRMS (ESI) for $C_{20}H_{22}O$: calculated for $[M+Na]^+$ 301.15629, found 301.15630.

Methyl (*Z*)-4-(3,3-dimethyl-1-phenylbut-1-en-1-yl)benzoate (80)

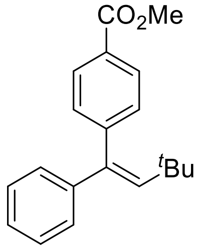

According to the general procedure C, the title compound was isolated as viscous oil after flash chromatography on silica gel (R_f = 0.65, eluent: 50:1 hexane: EtOAc) in 53% yield (31.6 mg, P_{syn} : P_{anti} = 91:9). ^{1}H NMR (400 MHz, $CDCl_3$) δ 8.06 (d, *J* = 8.1 Hz, 2H), 7.32 (d, *J* = 8.1 Hz, 2H), 7.24 (dd, *J* = 13.0, 7.2 Hz, 3H), 7.17 (d, *J* = 7.0 Hz, 2H), 6.14 (s, 1H), 3.96 (s, 3H), 0.99 (s, 9H). ^{13}C NMR (101 MHz, $CDCl_3$) δ 167.1, 146.1, 143.3, 140.8, 138.1, 130.5, 129.1, 128.7, 128.1, 126.9, 52.1, 34.1, 31.3. HRMS (ESI) for $C_{20}H_{22}O_2$: calculated for $[M+Na]^+$ 317.15120, found 317.15192.

(*Z*)-5-(3,3-dimethyl-1-phenylbut-1-en-1-yl)isobenzofuran-1(3*H*)-one (81)

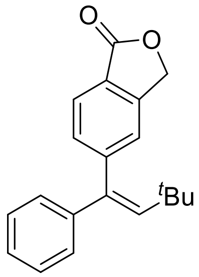

According to the general procedure D, the title compound was isolated as viscous oil after flash chromatography on silica gel (R_f = 0.65, eluent: 50:1 hexane: EtOAc) in 71% yield (41.4 mg, P_{syn} : P_{anti} = 90:10). ^{1}H NMR (400 MHz, $CDCl_3$) δ 7.93 (d, *J* = 7.8 Hz, 1H), 7.43 (d, *J* = 7.8 Hz, 1H), 7.35 (s, 1H), 7.25 (dd, *J* = 11.8, 7.2 Hz, 3H), 7.15 (d, *J* = 6.9 Hz, 2H), 6.17 (s, 1H), 5.35 (s, 2H), 0.99 (s, 9H). ^{13}C NMR (101 MHz, $CDCl_3$) δ 171.0, 147.7, 146.4, 143.1, 141.4, 137.6, 131.6, 128.3, 127.1, 126.9, 125.2, 124.4, 123.8, 69.6, 34.2, 31.4. HRMS (ESI) for $C_{20}H_{20}O_2$: calculated for $[M+Na]^+$ 315.13555, found 315.13619.

((5*R*,5a*S*,8a*S*,8b*R*)-2,2,7,7-tetramethyltetrahydro-5*H*-bis([1,3]dioxolo)[4,5-b:4',5'-d]pyran-5-yl)methyl 4-((*Z*)-3,3-dimethyl-1-phenylbut-1-en-1-yl)benzoate (82)

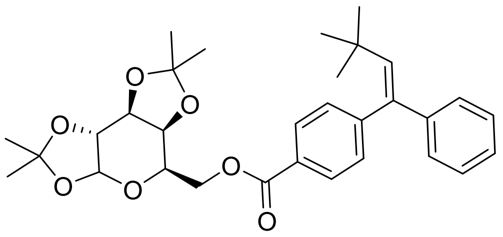

According to the general procedure C, the title compound was isolated as viscous oil after flash chromatography on silica gel (R_f = 0.56, eluent: 10:1 hexane: EtOAc) in 55% yield (58.0 mg, P_{syn} : P_{anti} = 86:14). ^{1}H NMR (400 MHz, $CDCl_3$) δ 8.06 (d, *J* = 8.1 Hz, 2H), 7.31 (d, *J* = 8.1 Hz, 2H), 7.27 – 7.15 (m, 5H), 6.13 (s, 1H), 5.61 (d, *J* = 4.9 Hz, 1H), 4.69 (dd, *J* = 7.9, 2.3 Hz, 1H), 4.57 – 4.45 (m, 2H), 4.40 – 4.36 (m, 2H), 4.23 (t, *J* = 6.1 Hz, 1H), 1.56 (s, 3H), 1.51 (s, 3H), 1.39 (s, 3H), 1.37 (s, 3H), 0.98 (s, 9H). ^{13}C NMR (101 MHz, $CDCl_3$) δ 166.4, 146.2, 143.3, 140.7, 138.1, 130.5, 129.3, 128.6, 128.1, 126.9, 109.7, 108.9, 96.4, 71.2, 70.7, 70.6, 66.2, 63.9, 34.1, 31.3, 26.1, 25.0, 24.5. HRMS (ESI) for $C_{31}H_{38}O_7$: calculated for $[M+Na]^+$ 545.25097, found 545.25102.

(*E*)-5-(4-cyanophenyl)-3,3-dimethyl-5-(m-tolyl)pent-4-en-1-yl 4-methoxybenzoate (83)

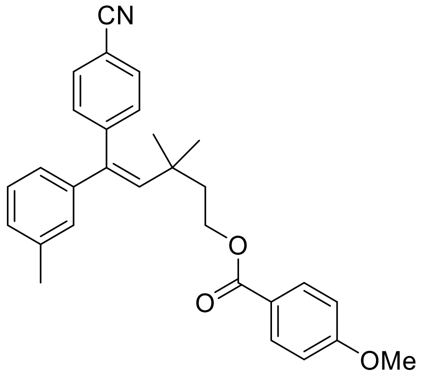

According to the general procedure C, the title compound was isolated as viscous oil after flash chromatography on silica gel (R_f = 0.45, eluent: 10:1 hexane: EtOAc) in 72% yield (63.5 mg, P_{syn} : P_{anti} = 89:11). ^{1}H NMR (400 MHz, $CDCl_3$) δ 7.93 (d, *J* = 8.9 Hz, 2H), 7.64 (d, *J* = 8.0 Hz, 2H), 7.36 (d, *J* = 8.1 Hz, 2H), 7.15 (t, *J* = 7.6 Hz, 1H), 7.05

(d, J = 7.5 Hz, 1H), 6.92 (d, J = 6.3 Hz, 2H), 6.86 (d, J = 8.8 Hz, 2H), 6.13 (s, 1H), 4.39 (t, J = 6.9 Hz, 2H), 3.87 (s, 3H), 2.30 (s, 3H), 1.83 (t, J = 7.0 Hz, 2H), 1.00 (s, 6H). ^{13}C NMR (101 MHz, $CDCl_3$) δ 166.4, 163.3, 146.0, 142.6, 139.0, 138.8, 137.9, 131.8, 131.5, 131.0, 128.2, 128.1, 127.5, 124.2, 122.6, 118.9, 113.6, 110.9, 62.2, 55.4, 42.7, 36.4, 29.5, 21.5. HRMS (ESI) for $C_{29}H_{29}NO_3$: calculated for $[M+Na]^+$ 462.20396, found 462.20398.

(*E*)-4-(1-(4-methoxyphenyl)-3,3-dimethylbut-1-en-1-yl)benzonitrile (84)

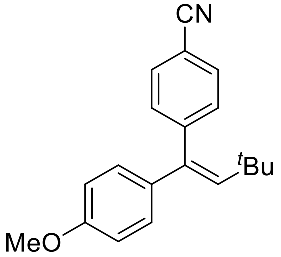

According to the general procedure C, the title compound was isolated as light-yellow solid after flash chromatography on silica gel (R_f = 0.45, eluent: 50:1 hexane: EtOAc) in 50% yield (29.2 mg, P_{syn} : P_{anti} = 86:14). ^{1}H NMR (400 MHz, $CDCl_3$) δ 7.66 (d, J = 8.1 Hz, 2H), 7.34 (d, J = 8.1 Hz, 2H), 7.05 (d, J = 8.7 Hz, 2H), 6.81 (d, J = 8.8 Hz, 2H), 6.06 (s, 1H), 3.80 (s, 3H), 0.97 (s, 9H). ^{13}C NMR (101 MHz, $CDCl_3$) δ 158.8, 146.6, 139.8, 136.8, 135.6, 131.7, 131.1, 128.0, 119.0, 113.6, 110.7, 55.3, 34.0, 31.4. HRMS (ESI) for $C_{20}H_{21}NO$: calculated for $[M+Na]^+$ 314.15153, found 314.15155.

(*Z*)-4-(1-(4-ethylphenyl)-3,3-dimethylbut-1-en-1-yl)benzonitrile (85)

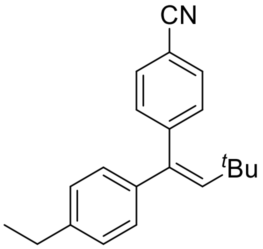

According to the general procedure C, the title compound was isolated as viscous oil after flash chromatography on silica gel (R_f = 0.55, eluent: 50:1 hexane: EtOAc) in 57% yield (33.1 mg, P_{syn} : P_{anti} = 93:7). ^{1}H NMR (400 MHz, $CDCl_3$) δ 7.67 (d, J = 8.0 Hz, 2H), 7.35 (d, J = 8.0 Hz, 2H), 7.12 (d, J = 8.2 Hz, 2H), 7.05 (d, J = 8.2 Hz, 2H), 6.13 (s, 1H), 2.64 (q, J = 7.6, 6.9 Hz, 2H), 1.23 (t, J = 7.6 Hz, 3H), 0.98 (s, 9H). ^{13}C NMR (101 MHz, $CDCl_3$) δ 146.5, 143.3, 140.6, 140.3, 137.2, 131.6, 131.2, 127.8, 126.8, 119.0, 110.7, 34.1, 31.4, 28.4, 15.6. HRMS (ESI) for $C_{21}H_{23}N$: calculated for $[M+Na]^+$ 312.17227, found 312.17279.

(*E*)-4-(1-(4-fluorophenyl)-3,3-dimethylbut-1-en-1-yl)benzonitrile (86)

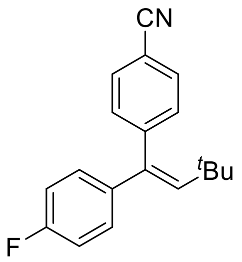

According to the general procedure D, the title compound was isolated as viscous oil after flash chromatography on silica gel (R_f = 0.55, eluent: 50:1 hexane: EtOAc) in 62% yield (34.3 mg, P_{syn} : P_{anti} = 94:6). ^{1}H NMR (400 MHz, $CDCl_3$) δ 7.67 (d, *J* = 8.0 Hz, 2H), 7.33 (d, *J* = 8.0 Hz, 2H), 7.12 – 7.06 (m, 2H), 6.95 (t, *J* = 8.6 Hz, 2H), 6.08 (s, 1H), 0.98 (s, 9H). ^{13}C NMR (101 MHz, $CDCl_3$) δ 162.1 (d, *J* = 246.8 Hz), 146.0, 141.3, 139.0 (d, *J* = 3.2 Hz), 136.4, 131.8, 131.1, 128.5 (d, *J* = 8.0 Hz), 118.9, 115.1 (d, *J* = 21.4 Hz), 111.0, 34.1, 31.3. ^{19}F NMR (377 MHz, $CDCl_3$) δ -115.54. HRMS (ESI) for $C_{19}H_{18}FN$: calculated for $[M+Na]^+$ 302.13155, found 302.13157.

(*E*)-4-(3,3-dimethyl-1-(m-tolyl)but-1-en-1-yl)benzonitrile (87)

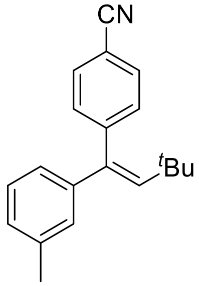

According to the general procedure C, the title compound was isolated as viscous oil after flash chromatography on silica gel (R_f = 0.60, eluent: 50:1 hexane: EtOAc) in 73% yield (40.1 mg, P_{syn} : P_{anti} = 92:8). ^{1}H NMR (400 MHz, $CDCl_3$) δ 7.67 (d, *J* = 8.1 Hz, 2H), 7.35 (d, *J* = 8.1 Hz, 2H), 7.17 (t, *J* = 7.6 Hz, 1H), 7.06 (d, *J* = 7.5 Hz, 1H), 6.96 (s, 1H), 6.92 (d, *J* = 7.8 Hz, 1H), 6.14 (s, 1H), 2.33 (s, 3H), 0.99 (s, 9H). ^{13}C NMR (101 MHz, $CDCl_3$) δ 146.4, 142.9, 141.2, 137.9, 137.5, 131.7, 131.1, 128.2, 127.9, 127.5, 124.1, 119.0, 110.8, 34.1, 31.3, 21.5. HRMS (ESI) for $C_{20}H_{21}N$: calculated for $[M+Na]^+$ 298.15662, found 298.15571.

(*E*)-4-(1-(3-hydroxyphenyl)-3,3-dimethylbut-1-en-1-yl)benzonitrile (88)

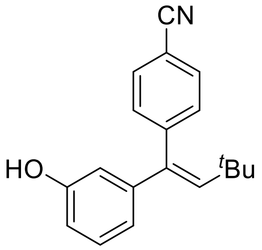

According to the general procedure D, the title compound was isolated as white solid after flash chromatography on silica gel (R_f = 0.42, eluent: 10:1 hexane: EtOAc) in 55% yield (30.5 mg, P_{syn} : P_{anti} = 93:7). ^{1}H NMR (400 MHz, $CDCl_3$) δ 7.66 (d, *J* = 8.0 Hz, 2H), 7.34 (d, *J* = 8.0 Hz, 2H), 7.14 (t, *J* = 7.9 Hz, 1H), 6.72 (d, *J* = 7.9 Hz, 2H), 6.59 (s, 1H), 6.16 (s, 1H), 4.92 (s, 1H), 0.97 (s, 9H). ^{13}C NMR (101 MHz, $CDCl_3$) δ 155.5, 146.1, 144.6, 141.6, 136.9, 131.7, 131.2, 129.4, 119.4, 118.9, 114.0, 114.0, 110.8, 34.1, 31.3. HRMS (ESI) for $C_{19}H_{19}NO$: calculated for $[M+Na]^+$ 300.13588, found 300.13588.

(*E*)-4-(1-(2-methoxyphenyl)-3,3-dimethylbut-1-en-1-yl)benzonitrile (89)

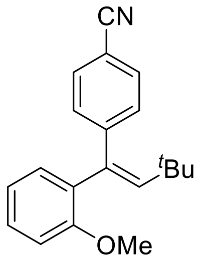

According to the general procedure C, the title compound was isolated as white solid after flash chromatography on silica gel (R_f = 0.45, eluent: 50:1 hexane: EtOAc) in 55% yield (32.1 mg, P_{syn} : P_{anti} > 99:1). ^{1}H NMR (400 MHz, $CDCl_3$) δ 7.56 (d, *J* = 8.1 Hz, 2H), 7.41 (d, *J* = 8.1 Hz, 2H), 7.28 – 7.20 (m, 2H), 6.95 (t, *J* = 7.4 Hz, 1H), 6.80 (d, *J* = 8.1 Hz, 1H), 5.82 (s, 1H), 3.62 (s, 3H), 1.00 (s, 9H). ^{13}C NMR (101 MHz, $CDCl_3$) δ 156.8, 147.0, 143.5, 136.0, 133.9, 130.9, 130.8, 130.6, 128.8, 120.6, 119.3, 111.3, 109.9, 55.3, 34.2, 31.4. HRMS (ESI) for $C_{20}H_{21}NO$: calculated for $[M+Na]^+$ 314.15153, found 314.15069.

(*E*)-4-(3,3-dimethyl-1-(2,3,5-trimethylphenyl)but-1-en-1-yl)benzonitrile (90)

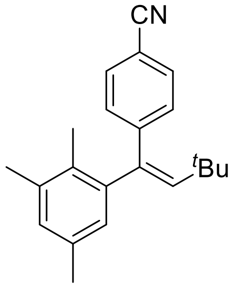

According to the general procedure C, the title compound was isolated as viscous oil after flash chromatography on silica gel (R_f = 0.58, eluent: 50:1 hexane: EtOAc) in 50% yield (30.2 mg, P_{syn} : P_{anti} > 99:1). ^{1}H NMR (400 MHz, $CDCl_3$) δ 7.59 (d, *J* = 8.1 Hz, 2H), 7.38 (d, *J* = 8.1 Hz, 2H), 6.96 (s, 1H), 6.87 (s, 1H), 5.69 (s, 1H), 2.25 (s, 3H), 2.22 (d, *J* = 4.4 Hz, 6H), 1.03 (s, 9H). ^{13}C NMR (101 MHz, $CDCl_3$) δ 147.0, 143.8, 141.5, 136.8, 135.5, 133.7, 132.3, 132.0, 131.4, 130.8, 130.4, 119.1, 110.3, 34.2, 31.4, 20.0, 19.3, 19.2. HRMS (ESI) for $C_{22}H_{25}N$: calculated for $[M+Na]^+$ 326.18792, found 326.18748.

(*E*)-4-(3,3-dimethyl-1-(2-(trifluoromethyl)phenyl)but-1-en-1-yl)benzonitrile (91)

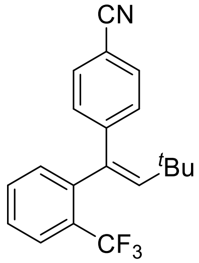

According to the general procedure C, the title compound was isolated as viscous oil after flash chromatography on silica gel (R_f = 0.55, eluent: 50:1 hexane: EtOAc) in 52% yield (34.5 mg, P_{syn} : P_{anti} > 99:1). ^{1}H NMR (400 MHz, $CDCl_3$) δ 7.69 (d, *J* = 7.9 Hz, 1H), 7.61 (d, *J* = 8.1 Hz, 2H), 7.45 (d, *J* = 8.0 Hz, 3H), 7.35 (t, *J* = 7.6 Hz, 1H), 7.22 (d, *J* = 7.7 Hz, 1H), 5.79 (s, 1H), 1.01 (s, 9H). ^{13}C NMR (101 MHz, $CDCl_3$) δ 145.8, 145.3, 142.9, 133.9, 131.6, 131.5, 131.3, 130.6, 128.0 (d, *J* = 29.1 Hz), 127.1, 126.7 (q, *J* = 5.4 Hz), 124.3 (q, *J* = 273.9 Hz), 118.8, 110.9, 34.4, 31.0. ^{19}F NMR (377 MHz, $CDCl_3$) δ -55.79. HRMS (ESI) for $C_{20}H_{18}F_3N$: calculated for $[M+Na]^+$ 352.12835, found 352.12826.

4-((*E*)-3,3-dimethyl-1-((8*R*,9*S*,13*S*,14*S*)-13-methyl-17-oxo-7,8,9,11,12,13,14,15,16,17-decahydro-6*H*-cyclopenta[a]phenanthren-3-yl)but-1-en-1-yl)benzonitrile (92)

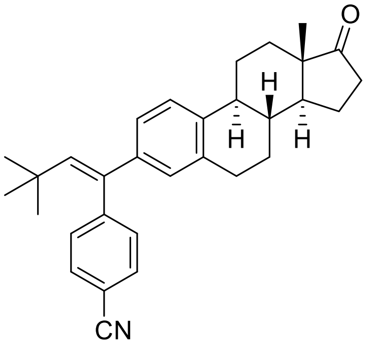

According to the general procedure C, the title compound was isolated as viscous oil after flash chromatography on silica gel (R_f = 0.40, eluent: 10:1 hexane: EtOAc) in 46% yield (40.3 mg, P_{syn} : P_{anti} = 91:9). ^{1}H NMR (400 MHz, $CDCl_3$) δ 7.67 (d, *J* = 8.0 Hz, 2H), 7.34 (d, *J* = 8.0 Hz, 2H), 7.20 (d, *J* = 8.2 Hz, 1H), 6.92 (d, *J* = 8.4 Hz, 1H), 6.85 (s, 1H), 6.11 (s, 1H), 2.87 (dd, *J* = 8.5, 3.5 Hz, 2H), 2.52 (dd, *J* = 18.8, 8.6 Hz, 1H), 2.41 (d, *J* = 9.7 Hz, 1H), 2.29 (t, *J* = 8.7 Hz, 1H), 2.12 – 1.95 (m, 4H), 1.71 – 1.55 (m, 4H), 1.50 – 1.40 (m, 2H), 0.97 (s, 9H), 0.92 (s, 3H). ^{13}C NMR (101 MHz, $CDCl_3$) δ 220.8, 146.3, 140.9, 140.4, 138.9, 137.0, 136.4, 131.7, 131.1, 127.3, 125.3, 124.3, 119.0, 110.8, 50.5, 48.0, 44.3, 38.1, 35.9, 34.1, 31.6, 31.3, 29.5, 26.5, 25.7, 21.6, 13.9. HRMS (ESI) for $C_{31}H_{35}NO$: calculated for $[M+Na]^+$ 460.26109, found 460.26105.

(*Z*)-5-(4-cyanophenyl)-3,3-dimethyl-5-phenylpent-4-en-1-yl methyl terephthalate (**96**)

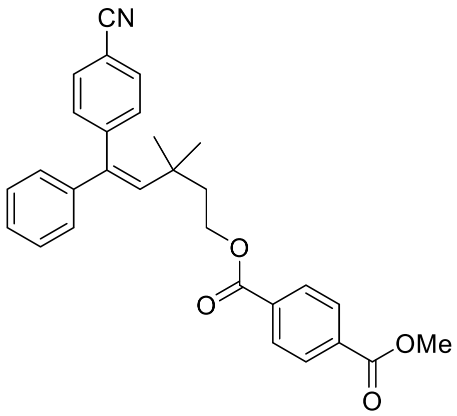

According to the general procedure D, the title compound was isolated as viscous oil after flash chromatography on silica gel (R_f = 0.38, eluent: 10:1 hexane: EtOAc) in 80% yield (72.4 mg, P_{syn} : P_{anti} = 86:14). ^{1}H NMR (400 MHz, $CDCl_3$) δ 8.04 (s, 4H), 7.66 (d, *J* = 8.1 Hz, 2H), 7.36 (d, *J* = 8.1 Hz, 2H), 7.26 – 7.22 (m, 3H), 7.10 (dd, *J* = 7.2, 2.1 Hz, 2H), 6.15 (s, 1H), 4.45 (t, *J* = 7.1 Hz, 2H), 3.96 (s, 3H), 1.87 (t, *J* = 7.1 Hz, 2H), 1.01 (s, 6H). ^{13}C NMR (101 MHz, $CDCl_3$) δ 166.2, 165.8, 145.8, 142.5, 138.9, 138.9, 133.9, 131.8, 131.0, 130.0, 129.6, 129.5, 128.3, 127.4, 126.8, 118.8, 111.1, 62.9, 52.5, 42.7, 36.4, 29.4. HRMS (ESI) for $C_{29}H_{27}NO_4$: calculated for $[M+Na]^+$ 476.18323, found 476.18320.

(*Z*)-3,3-dimethyl-5-((8*R*,9*S*,13*S*,14*S*)-13-methyl-17-oxo-7,8,9,11,12,13,14,15,16,17-decahydro-6*H*-cyclopenta[a]phenanthren-3-yl)-5-(4-((((5*R*,5a*S*,8a*S*,8b*R*)-2,2,7,7-tetramethyltetrahydro-5*H*-bis([1,3]dioxolo)[4,5-b:4',5'-d]pyran-5-yl)methoxy)carbonyl)phenyl)pent-4-en-1-yl 4-(*N*,*N*-dipropylsulfamoyl)benzo ate (P_{anti}-98)

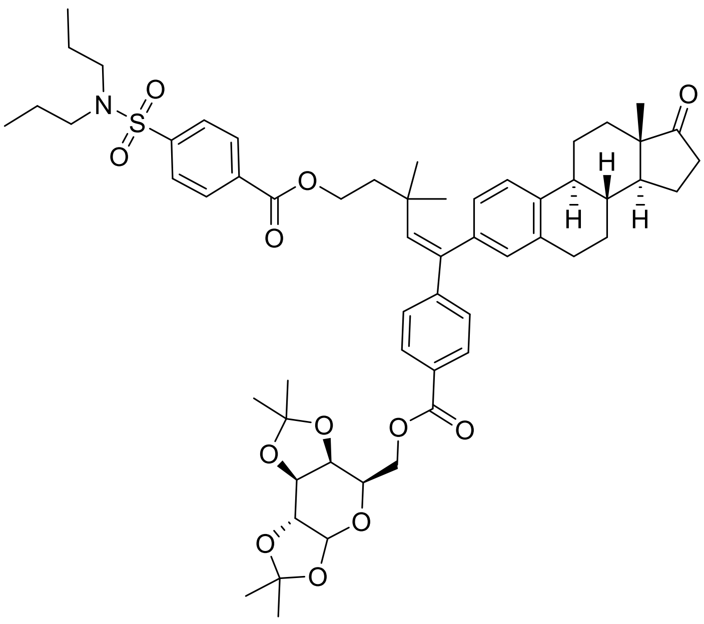

According to the general procedure A, the title compound was isolated as viscous oil after flash chromatography on silica gel (R_f = 0.30, eluent: 5:1 hexane: EtOAc) in 42% yield (83.8 mg). ^{1}H NMR (400 MHz, $CDCl_3$) δ 8.11 (d, *J* = 8.3 Hz, 2H), 7.92 (d, *J* = 8.3 Hz, 2H), 7.82 (d, *J* = 8.3 Hz, 2H), 7.25 (d, *J* = 8.3 Hz, 3H), 6.97 (d, *J* = 7.9 Hz, 1H), 6.88 (s, 1H), 6.13 (s, 1H), 5.56 (d, *J* = 4.9 Hz, 1H), 4.65 (dd, *J* = 7.8, 2.3 Hz, 1H), 4.50 (dd, *J* = 11.5, 4.9 Hz, 1H), 4.46 – 4.38 (m, 3H), 4.36 – 4.30 (m, 2H), 4.17 (t, *J* = 6.0 Hz, 1H), 3.12 – 3.08 (m, 4H), 2.86 (s, 2H), 2.53 (dd, *J* = 18.9, 8.5 Hz, 1H), 2.43 (d, *J* = 11.3 Hz, 1H), 2.32 (t, *J* = 8.8 Hz, 1H), 2.23 – 2.05 (m, 3H), 2.00 (d, *J* = 10.0 Hz, 2H), 1.84 (t, *J* = 7.2 Hz, 2H), 1.61 – 1.50 (m, 12H), 1.48 (s, 3H), 1.35 (d, *J* = 9.0 Hz, 6H), 1.05 (s, 6H), 0.96 (s, 3H), 0.87 (t, *J* = 7.3 Hz, 6H). ^{13}C NMR (101 MHz, $CDCl_3$) δ 220.9, 166.2, 165.3, 148.5, 144.2, 140.2, 139.3, 138.7, 136.9, 136.1, 133.6, 130.3, 130.1, 129.5, 128.2, 127.4, 127.0, 126.8, 124.8, 109.7, 108.8, 96.3, 71.1, 70.7, 70.5, 66.1, 63.7, 63.5, 50.6, 49.9, 48.0, 44.3, 42.1, 38.1, 36.4, 35.9, 31.6, 29.5, 29.3, 26.5, 26.1, 26.0, 25.7, 25.0, 24.5, 21.9, 21.6, 13.9, 11.2. HRMS (ESI) for $C_{57}H_{73}NO_{12}S$: calculated for $[M+Na]^+$ 1018.47457, found 1018.47536.

(*E*)-3,3-dimethyl-5-((8*R*,9*S*,13*S*,14*S*)-13-methyl-17-oxo-7,8,9,11,12,13,14,15,16,17-decahydro-6*H*-cyclopenta[a]phenanthren-3-yl)-5-(4-((((5*R*,5a*S*,8a*S*,8b*R*)-2,2,7,7-tetramethyltetrahydro-5*H*-bis([1,3]dioxolo)[4,5-b:4',5'-d]pyran-5-yl)methoxy)carbonyl)phenyl)pent-4-en-1-yl 4-(*N*,*N*-dipropylsulfamoyl)benzoate (P$_{syn}$-98)

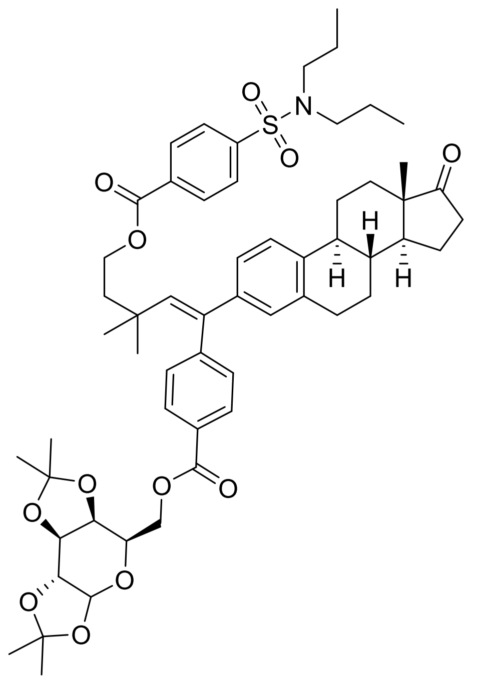

According to the general procedure B or the general procedure D, the title compound was isolated as viscous oil after flash chromatography on silica gel (R_f = 0.30, eluent: 5:1 hexane: EtOAc) in 55% yield (109.5 mg, P_{syn} : P_{anti} = 84:16) or 39% yield (77.6 mg, P_{syn} : P_{anti} = 84:16). ^{1}H NMR (400 MHz, $CDCl_3$) δ 8.08 (d, J = 8.3 Hz, 2H), 8.04 (d, J = 8.0 Hz, 2H), 7.78 (d, J = 8.3 Hz, 2H), 7.28 (dd, J = 17.7, 8.2 Hz, 2H), 7.17 (d, J = 8.3 Hz, 1H), 6.95 (d, J = 8.0 Hz, 1H), 6.86 (s, 1H), 6.13 (s, 0.16H), 6.11 (s, 0.84H), 5.59 (d, J = 4.9 Hz, 1H), 4.68 (dd, J = 7.9, 2.2 Hz, 1H), 4.55 – 4.41 (m, 4H), 4.39 – 4.30 (m, 2H), 4.22 (t, J = 6.0 Hz, 1H), 3.11 – 3.07 (m, 4H), 2.89 – 2.79 (m, 2H), 2.50 (dd, J = 18.7, 8.6 Hz, 1H), 2.39 (d, J = 10.5 Hz, 1H), 2.30 (t, J = 8.2 Hz, 1H), 2.19 – 1.92 (m, 5H), 1.86 – 1.81 (m, 2H), 1.58 – 1.47 (m, 15H), 1.37 (d, J = 9.3 Hz, 6H), 1.02 (d, J = 24.3 Hz, 6H), 0.88 (dd, J = 14.2, 6.9 Hz, 9H). ^{13}C NMR (101 MHz, $CDCl_3$) δ 220.9, 166.3, 165.3, 145.8, 144.1, 140.4, 139.3, 139.0, 137.6, 136.4, 133.5, 130.2, 129.4, 128.8, 127.3, 126.9, 125.2, 124.3, 109.7, 108.8, 96.4, 71.2, 70.7, 70.5, 66.2, 64.1, 63.3, 50.5, 49.9, 48.0, 44.3, 42.7, 38.1, 36.2, 35.9, 31.6, 29.5, 29.4, 26.4, 26.1, 26.0, 25.7, 25.0, 24.5, 22.0, 21.6, 13.9, 11.2. HRMS (ESI) for $C_{57}H_{73}NO_{12}S$: calculated for $[M+Na]^+$ 1018.47457, found 1018.47461.

4-(2-fluoro-1-methoxy-3,3-dimethyl-1-phenylbutyl)benzonitrile (99)

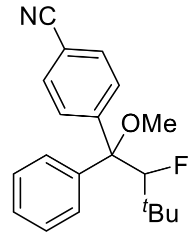

A 10 mL vial equipped with a stirrer bar was charged with alkene **4** (52.3 mg, 0.2 mmol, 1.0 equiv.), Selectfluor® (85 mg, 0.24 mmol, 1.2 equiv.), CH_3OH (81 uL, 2.0 mmol, 10 equiv.) and CH_3CN (2 mL) as solvent. The solution was stirred overnight under the room temperature. Then, the reaction mixture was concentrated under vacuum and the residue was purified by flash column chromatography on silica gel (R_f = 0.70, eluent: 30:1 hexane: EtOAc) to give the title product as viscous oil in 99% yield (61.5 mg).[4] ^{1}H NMR (400 MHz, $CDCl_3$) δ 7.66 – 7.53 (m, 4H), 7.41 – 7.21 (m, 5H), 5.23 (d, *J* = 42.2 Hz, 1H), 3.16 (s, 3H), 0.84 (s, 9H). ^{13}C NMR (101 MHz, $CDCl_3$) δ 148.68, 142.34 (d, *J* = 3.9 Hz), 131.51, 129.22 (d, *J* = 3.6 Hz), 128.09, 127.96, 127.64, 118.95, 110.83, 98.99 (d, *J* = 187.1 Hz), 83.88 (d, *J* = 21.6 Hz), 53.43 (d, *J* = 3.5 Hz), 36.32 (d, *J* = 20.2 Hz), 27.32 (d, *J* = 5.3 Hz). ^{19}F NMR (377 MHz, $CDCl_3$) δ -185.33 (d, *J* = 42.2 Hz). HRMS (ESI) for $C_{20}H_{22}FNO$: calculated for $[M+Na]^+$ 334.15776, found 334.15768.

4-(3,3-dimethyl-1-phenyl-2-(trifluoromethyl)but-1-en-1-yl)benzonitrile (100)

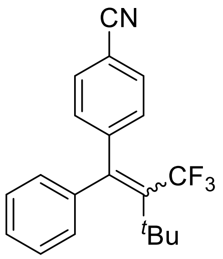

A 10 mL vial equipped with a stirrer bar was charged with alkene **4** (52.3 mg, 0.2 mmol, 1.0 equiv.), Umemoto's reagent (204 mg, 0.6 mmol, 3.0 equiv), $[Ru(bpy)_3](PF_6)_2$ (3.5 mg, 2 mol %), and DMSO (2.0 mL) under N_2. The vial was irradiated with 425 nm blue LEDs for 12 h at room temperature. Then, 20 mL H_2O was added and the resulting mixture was extracted with EtOAc (20 mL x 3). The combined organic phase was washed with H_2O, dried with Na_2SO_4 and filtered. The filtrate was concentrated and the residue was purified by column chromatography on silica gel (R_f = 0.60, eluent: 50:1 hexane: EtOAc) to afford the title product as viscous oil in 51% yield (33.5 mg, Z/E mixture 50:50, trace amount of impurity can not be removed).[5] ^{1}H NMR (400 MHz, $CDCl_3$) δ 7.61 (d, *J* = 8.2 Hz, 1H), 7.56 (d, *J* = 8.2 Hz, 1H), 7.32 (d, *J* = 6.5 Hz, 2H), 7.28 – 7.21 (m, 3H), 7.12 – 7.04 (m, 2H), 1.15 (s, 9H). ^{13}C NMR (101 MHz, $CDCl_3$) δ 148.2, 142.1, 131.9, 129.4, 128.6, 128.2, 128.0, 127.7, 125.0 (q, *J* = 272.5 Hz), 118.5, 111.4, 36.8, 32.2. ^{19}F NMR (377 MHz, $CDCl_3$) δ -49.27, -49.78. HRMS (ESI) for $C_{20}H_{18}F_3N$: calculated for $[M+Na]^+$ 352.12835, found 352.12820.

4-(3,3-dimethyl-1-phenylbutyl)benzonitrile (101)

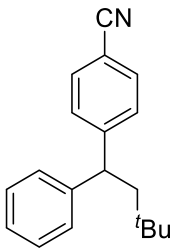

A dry 5-mL vial equipped with a Teflon-coated magnetic stir bar (10 mm*3 mm) was charged with alkene **4** (52.3 mg, 0.2 mmol, 1.0 equiv.) and $LiClO_4$ (11 mg, 0.1 mmol, 0.5 equiv.). Anhydrous CH_3CN (4.0 mL) and hydrazine mono hydrate (40 uL, 0.8 mmol, 4 equiv.) were added via syringe. Then, it was capped with a Teflon lid equipped with graphite electrode (20×7×2 mm) as the anode and cathode. The reaction mixture was stirred and electrolyzed at a constant cell voltage of 6 V under room temperature for 5 h. After the reaction is completed, the reaction mixture was concentrated under vacuum and the residue was purified by flash column chromatography on silica gel (R_f = 0.68, eluent: 30:1 hexane: EtOAc) to give the title product as viscous oil in 61% yield (32.1 mg).[6] ^{1}H NMR (400 MHz, $CDCl_3$) δ 7.57 (d, J = 8.2 Hz, 2H), 7.43 (d, J = 8.3 Hz, 2H), 7.33 – 7.27 (m, 4H), 7.24 – 7.18 (m, 1H), 4.13 (t, J = 6.7 Hz, 1H), 2.13 (qd, J = 14.1, 6.7 Hz, 2H), 0.86 (s, 9H). ^{13}C NMR (101 MHz, $CDCl_3$) δ 152.3, 145.1, 132.4, 128.8, 128.6, 127.7, 126.5, 119.0, 109.7, 49.0, 48.5, 31.6, 30.2. HRMS (ESI) for $C_{19}H_{21}N$: calculated for $[M+Na]^+$ 286.15662, found 286.15603.

4-(2-bromo-3,3-dimethyl-1-phenylbut-1-en-1-yl)benzonitrile (102)

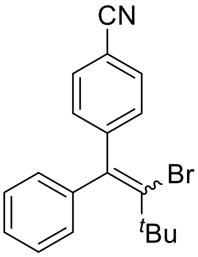

A vial equipped with a stirrer bar was charged with alkene **4** (52.3 mg, 0.2 mmol, 1.0 equiv.) and 1,2-dichloroethane (DCE, 2 mL) as solvent. The reaction mixture was cooled to 0 °C and Br_2 (30.7 uL, 0.6 mmol, 3 equiv.) in 0.5 mL DCE was added dropwise. The resultant mixture was stirred at 0 °C for 0.5 h. Next, pyridine (65 uL, 0.8 mmol, 4 equiv.) was added and the reaction mixture was stirred at 90 °C for 12 h. After being cooled, 20 mL $Na_2S_2O_3$ (aq.) was added and the resulting mixture was extracted with EtOAc (20 mL x 3). The combined organic phase was washed with H_2O, dried with Na_2SO_4 and filtered. The filtrate was concentrated and the residue was purified by column chromatography on silica gel (R_f = 0.60, eluent: 50:1 hexane: EtOAc) to afford the title product as viscous oil in 70% yield (47.7 mg, Z/E mixture 60:40). ^{1}H NMR (400 MHz, $CDCl_3$) δ 7.62 – 7.56 (m, 2H), 7.39 – 7.26 (m,

5H), 7.23 – 7.17 (m, 2H), 1.20 (s, 9H). ^{13}C NMR (101 MHz, $CDCl_3$) δ 151.3, 147.6, 145.9, 141.3, 140.8, 140.7, 140.2, 139.8, 132.3, 131.9, 129.5, 129.0, 128.7, 128.6, 128.3, 128.0, 127.6, 127.1, 118.9, 118.6, 110.9, 110.3, 41.7, 41.7, 32.2, 31.9. HRMS (ESI) for $C_{19}H_{18}BrN$: calculated for $[M+Na]^+$ 362.05148, found 362.05118.

4-(3-(tert-butyl)-2-phenyloxiran-2-yl)benzonitrile (103)

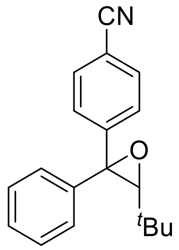

A vial equipped with a stirrer bar was charged with alkene **4** (52.3 mg, 0.2 mmol, 1.0 equiv.), m-CPBA (69.0 mg, 0.4 mmol, 2 equiv.), $NaHCO_3$ (25.2 mg, 0.3 mmol, 1.5 equiv.), and dichloromethane (2 mL) as solvent. The reaction mixture was stirred at room temperature for 12 h. After the reaction is completed, the reaction mixture was directly purified by column chromatography on silica gel (R_f = 0.55, eluent: 50:1 hexane: EtOAc) to afford the title product as viscous oil in 90% yield (50.1 mg). ^{1}H NMR (400 MHz, $CDCl_3$) δ 7.60 (d, J = 8.3 Hz, 2H), 7.51 (d, J = 8.5 Hz, 4H), 7.42 – 7.31 (m, 3H), 3.02 (s, 1H), 0.83 (s, 9H). ^{13}C NMR (101 MHz, $CDCl_3$) δ 147.6, 136.4, 132.2, 128.3, 128.1, 128.0, 127.1, 118.7, 111.2, 75.4, 65.9, 32.5, 26.8. HRMS (ESI) for $C_{19}H_{19}NO$: calculated for $[M+Na]^+$ 300.13588, found 300.13588.

13. Copies of NMR Spectra

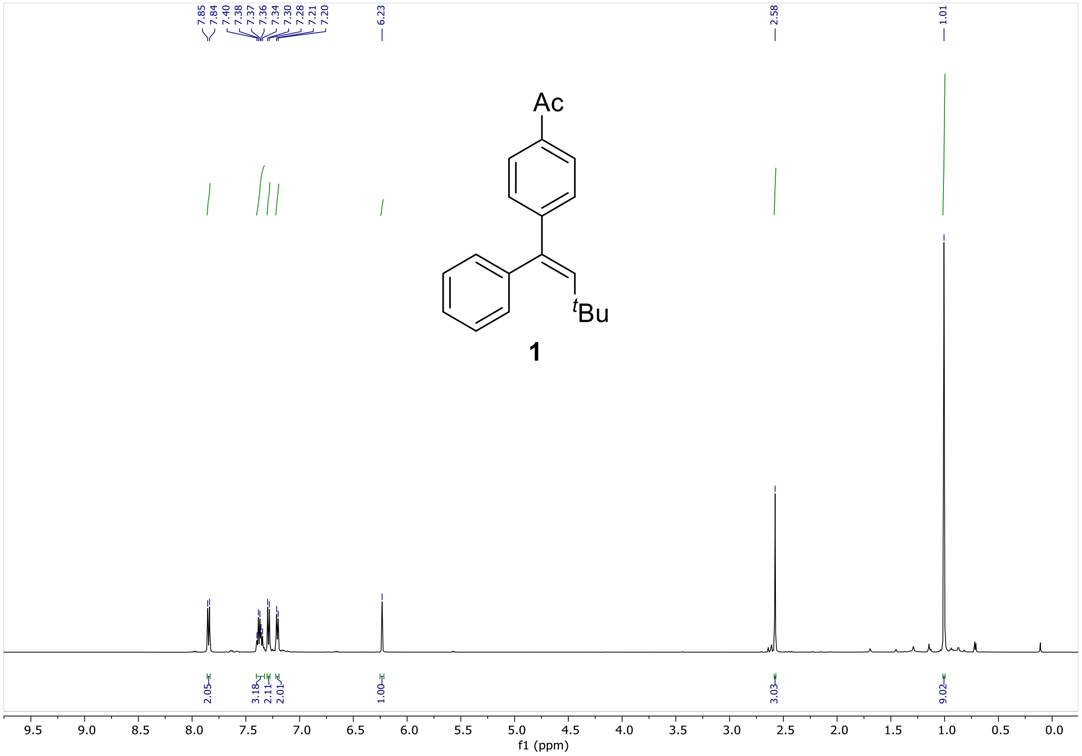

Supplementary Figure 23: ^{1}H NMR spectrum of compound **1**

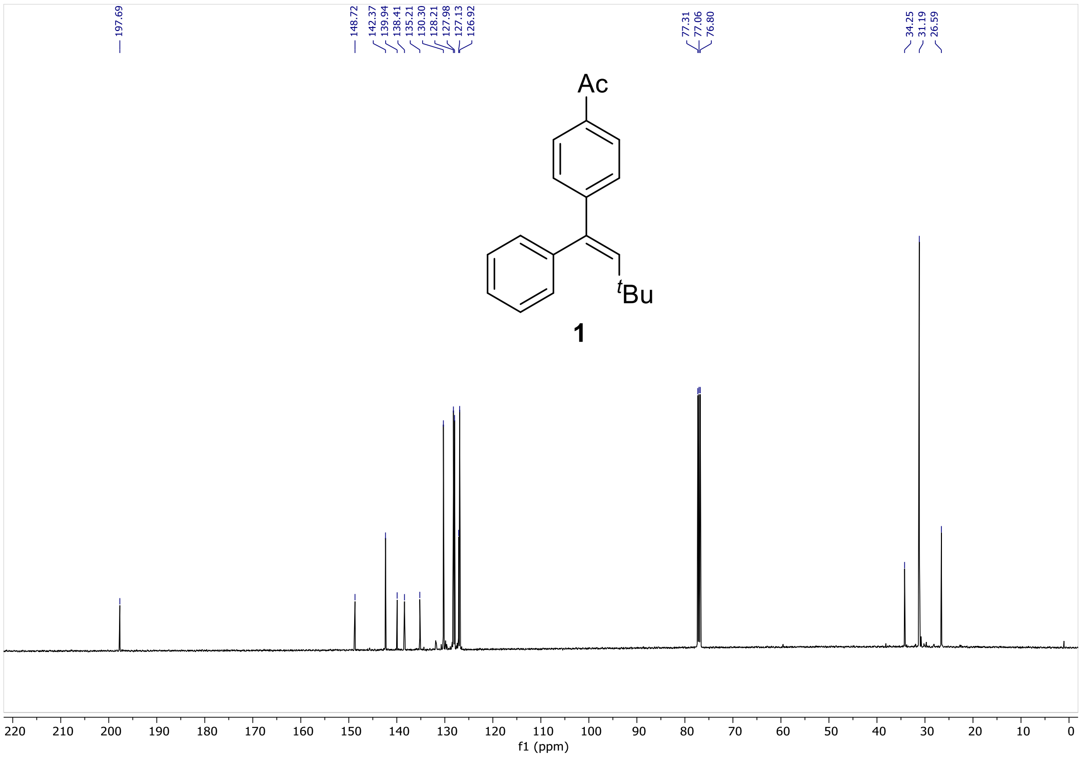

Supplementary Figure 24: ^{13}C NMR spectrum of compound **1**

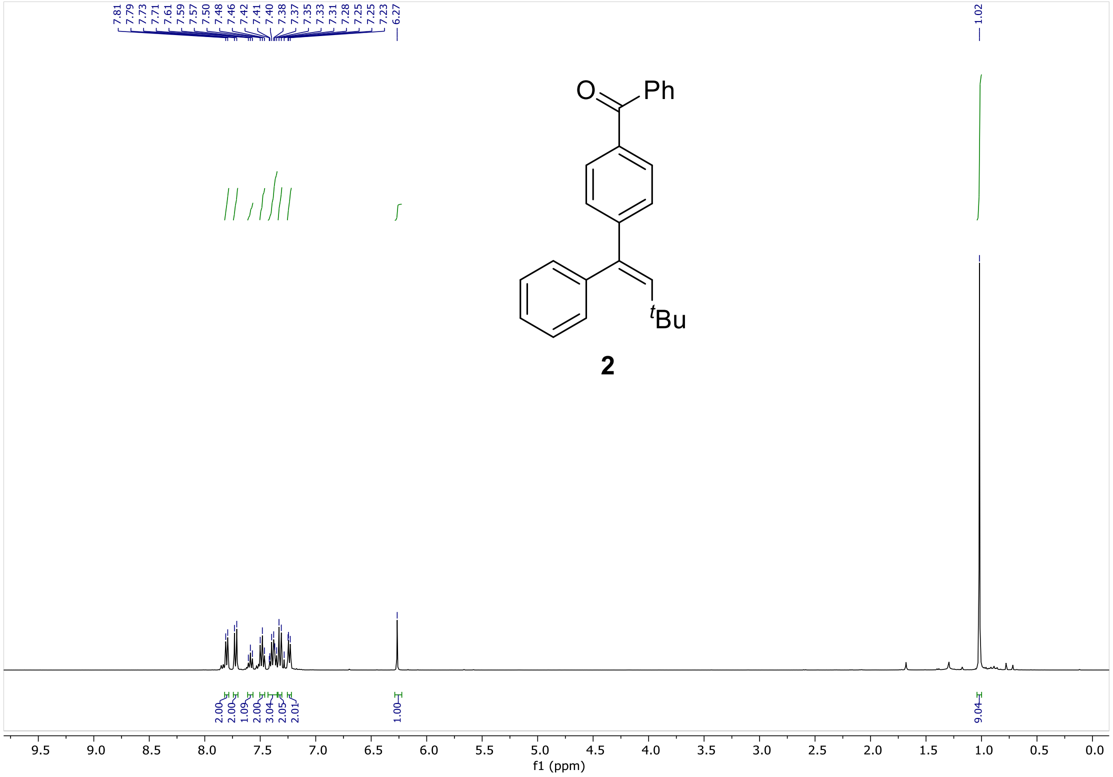

Supplementary Figure 25: ^{1}H NMR spectrum of compound **2**

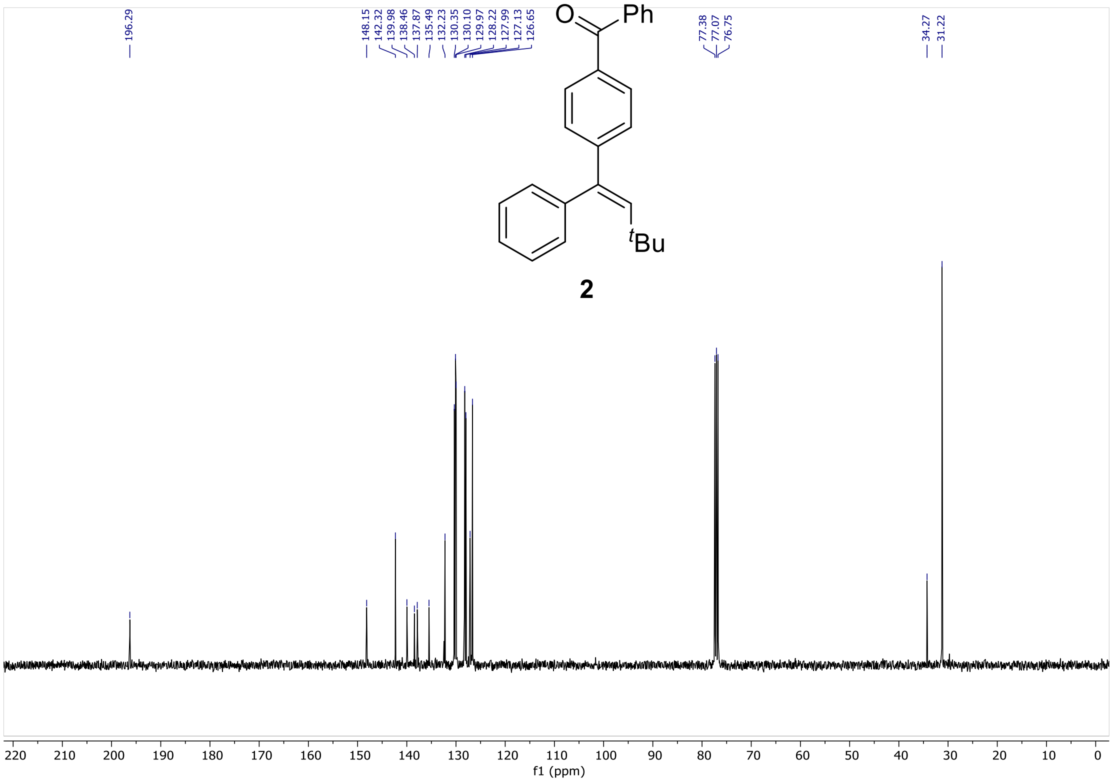

Supplementary Figure 26: ^{13}C NMR spectrum of compound **2**

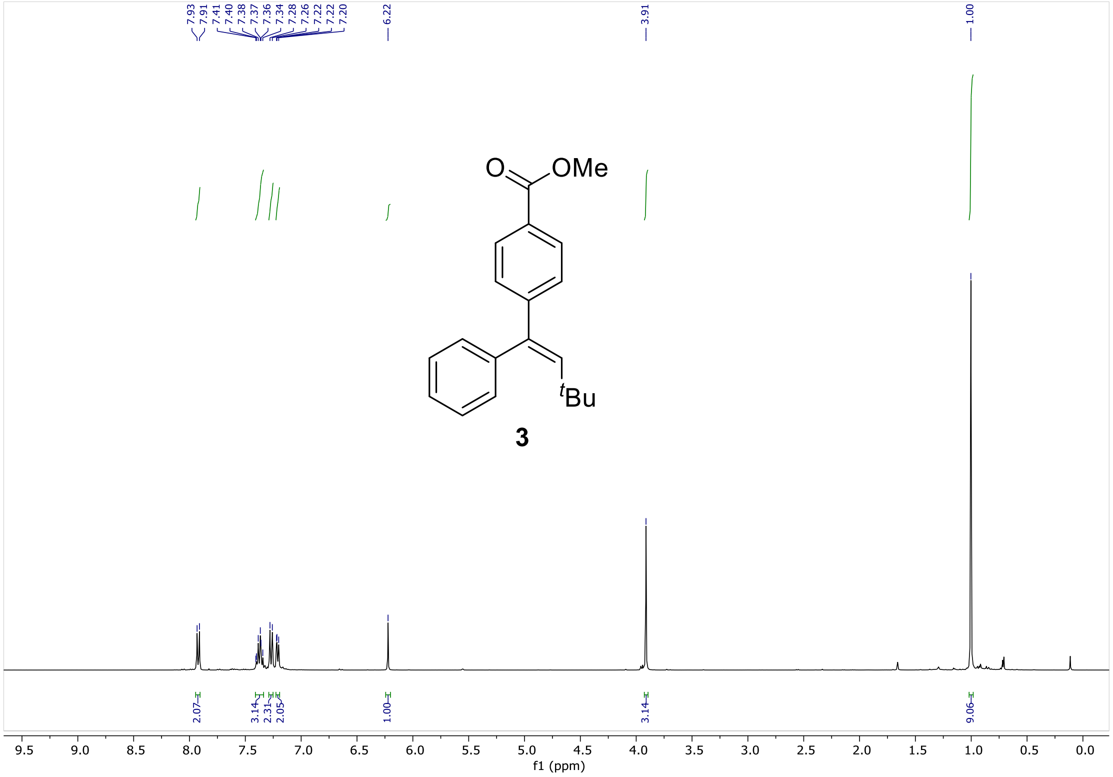

Supplementary Figure 27: ^{1}H NMR spectrum of compound **3**

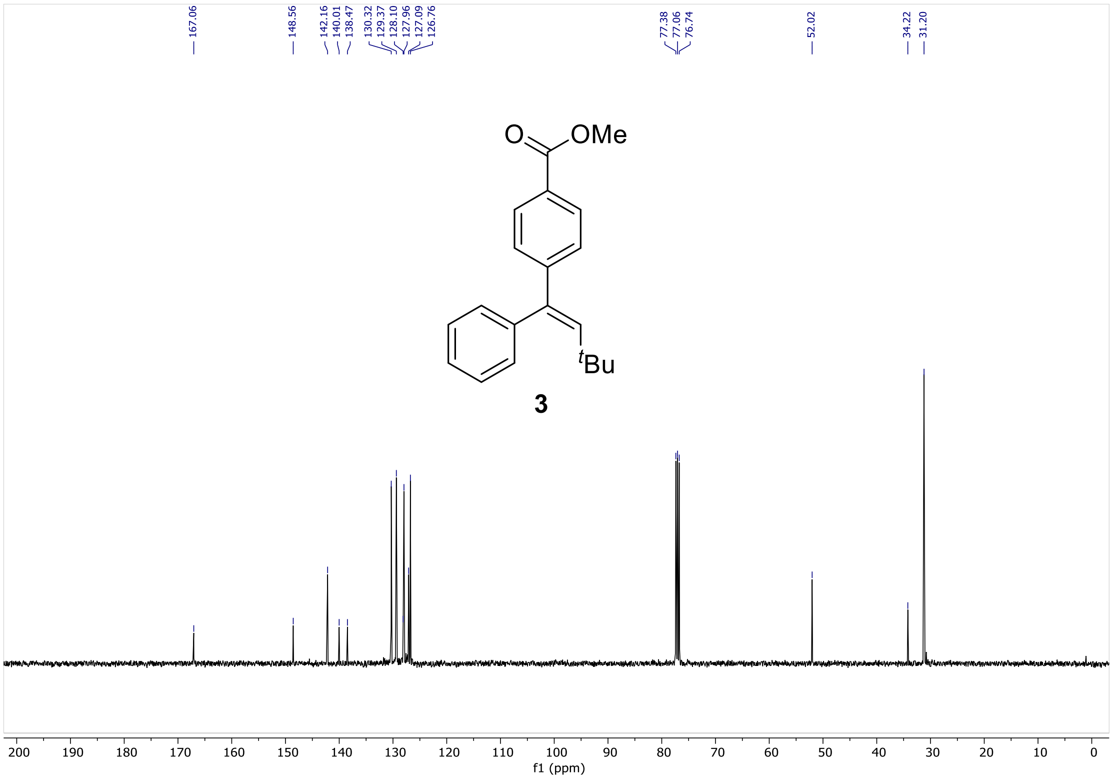

Supplementary Figure 28: ^{13}C NMR spectrum of compound **3**

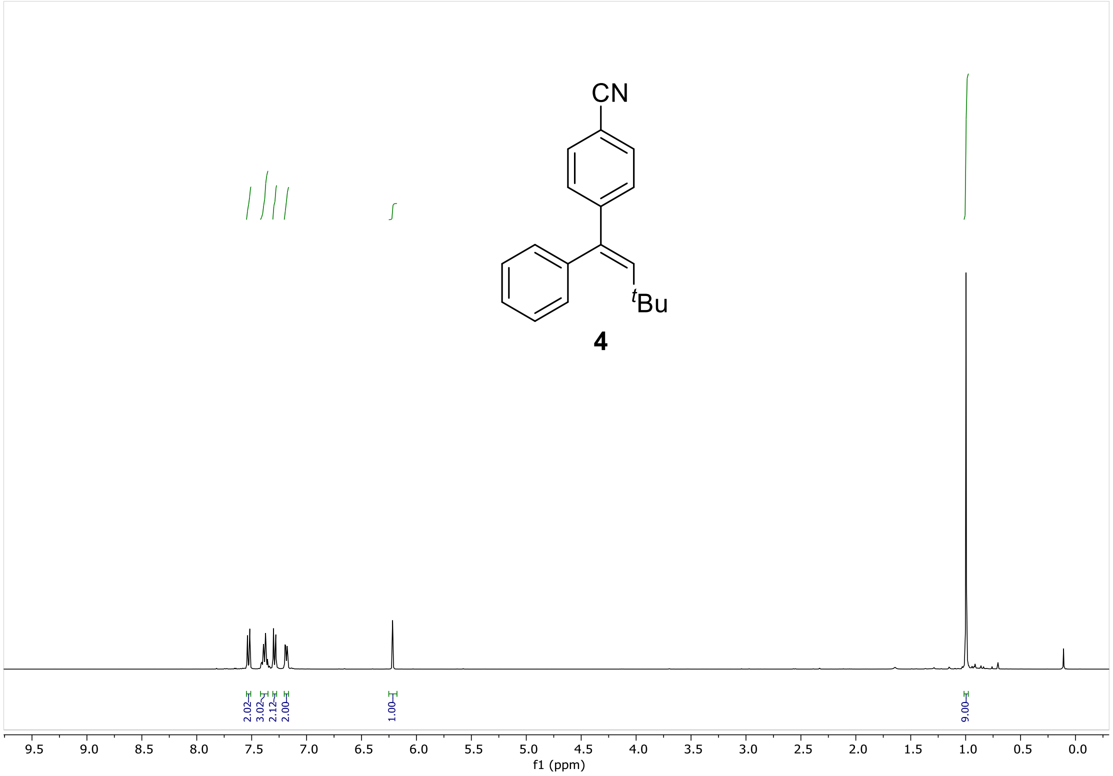

Supplementary Figure 29: ^{1}H NMR spectrum of compound **4**

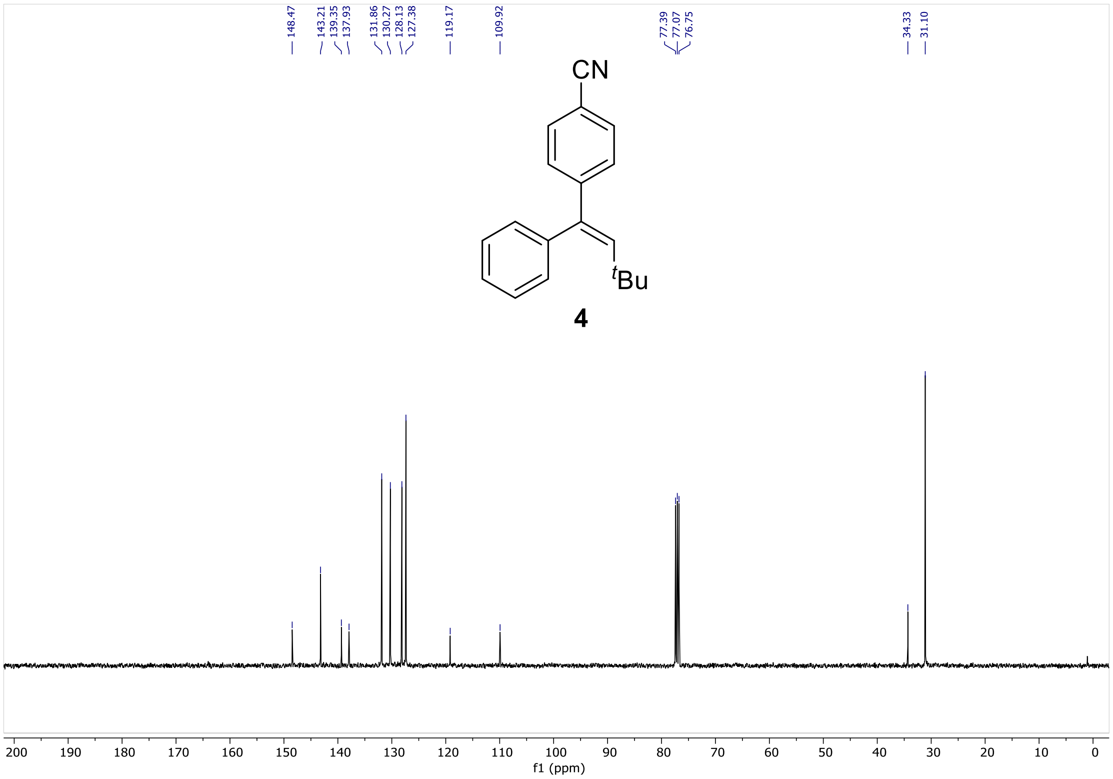

Supplementary Figure 30: ^{13}C NMR spectrum of compound **4**

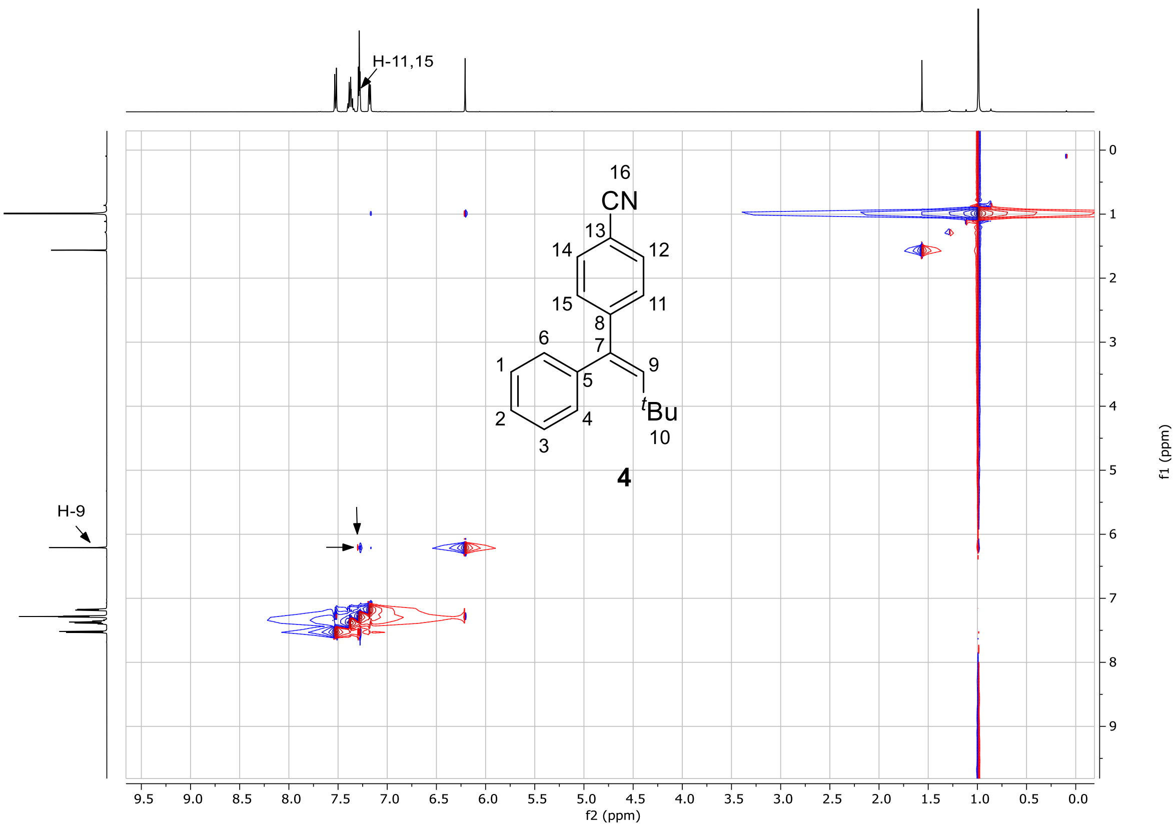

Supplementary Figure 31: NOE spectrum of compound **4**

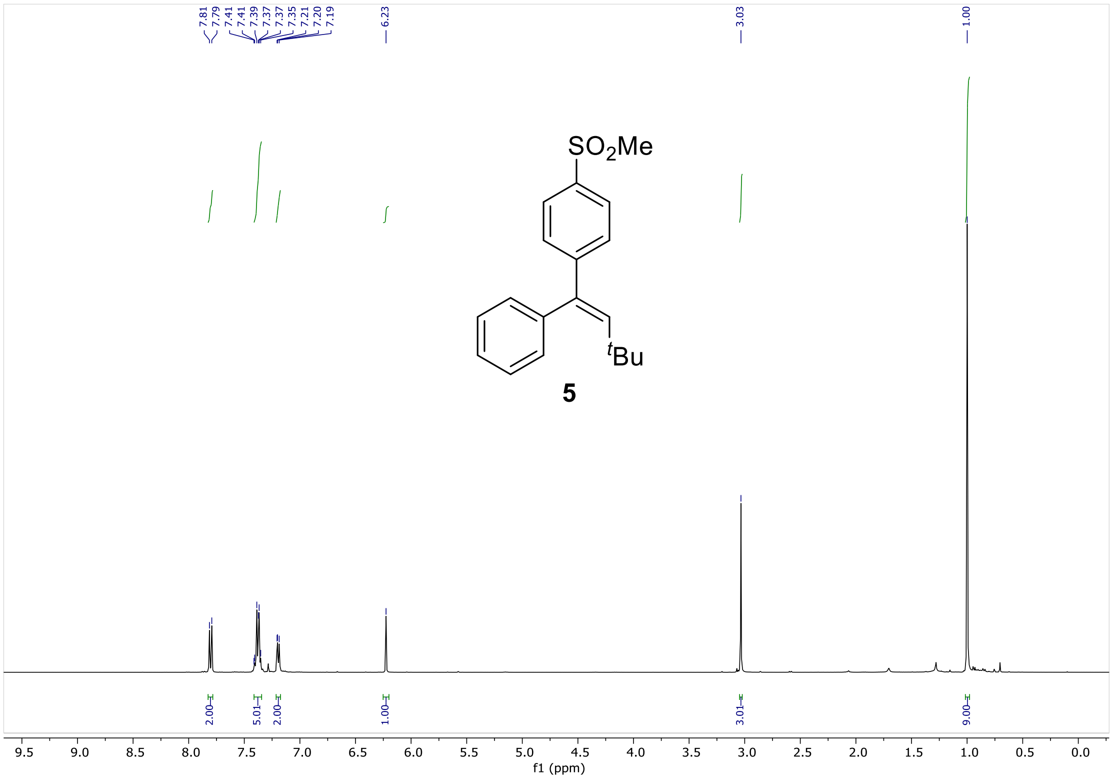

Supplementary Figure 32: ^{1}H NMR spectrum of compound **5**

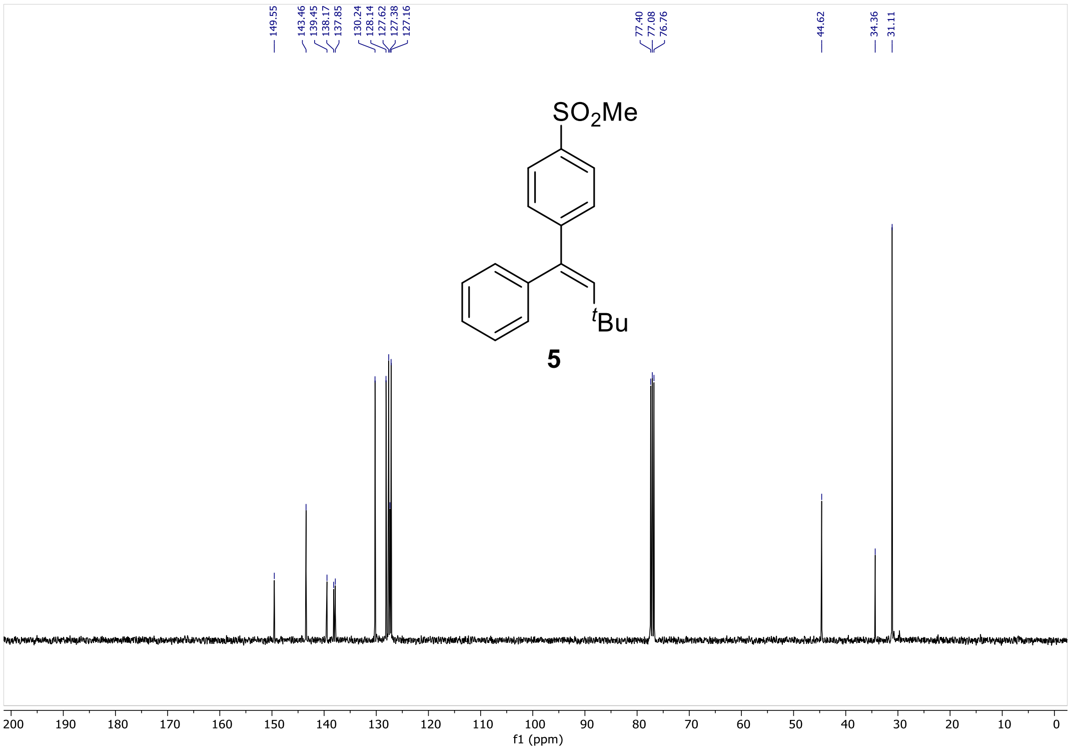

Supplementary Figure 33: ^{13}C NMR spectrum of compound **5**

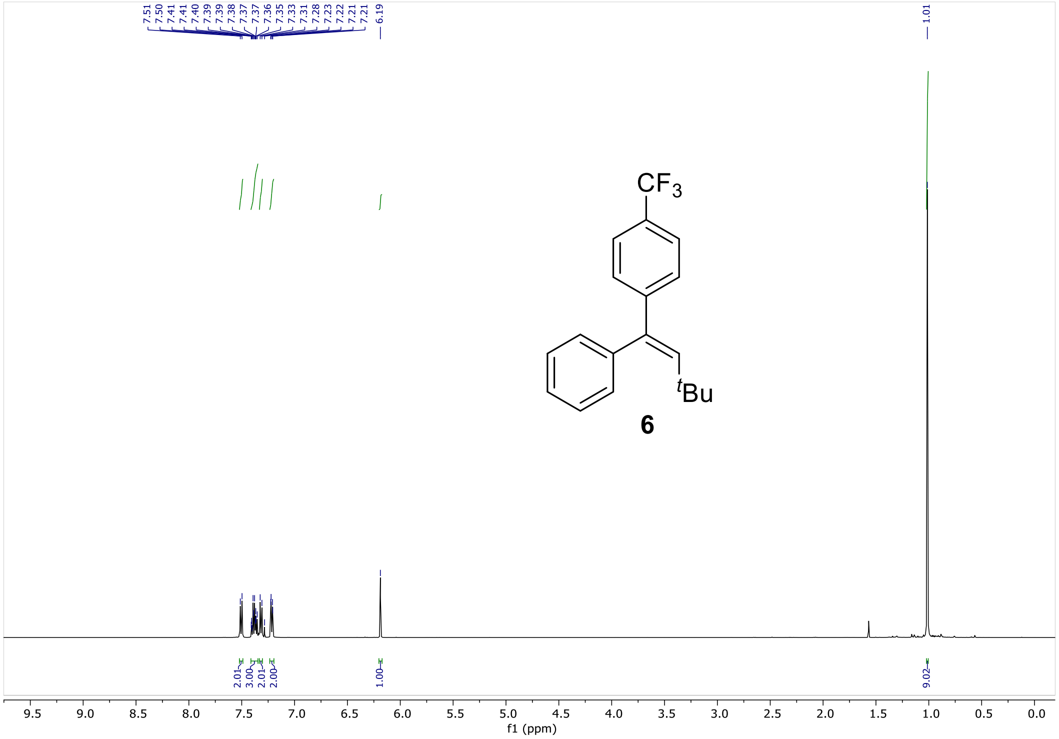

Supplementary Figure 34: ^{1}H NMR spectrum of compound **6**

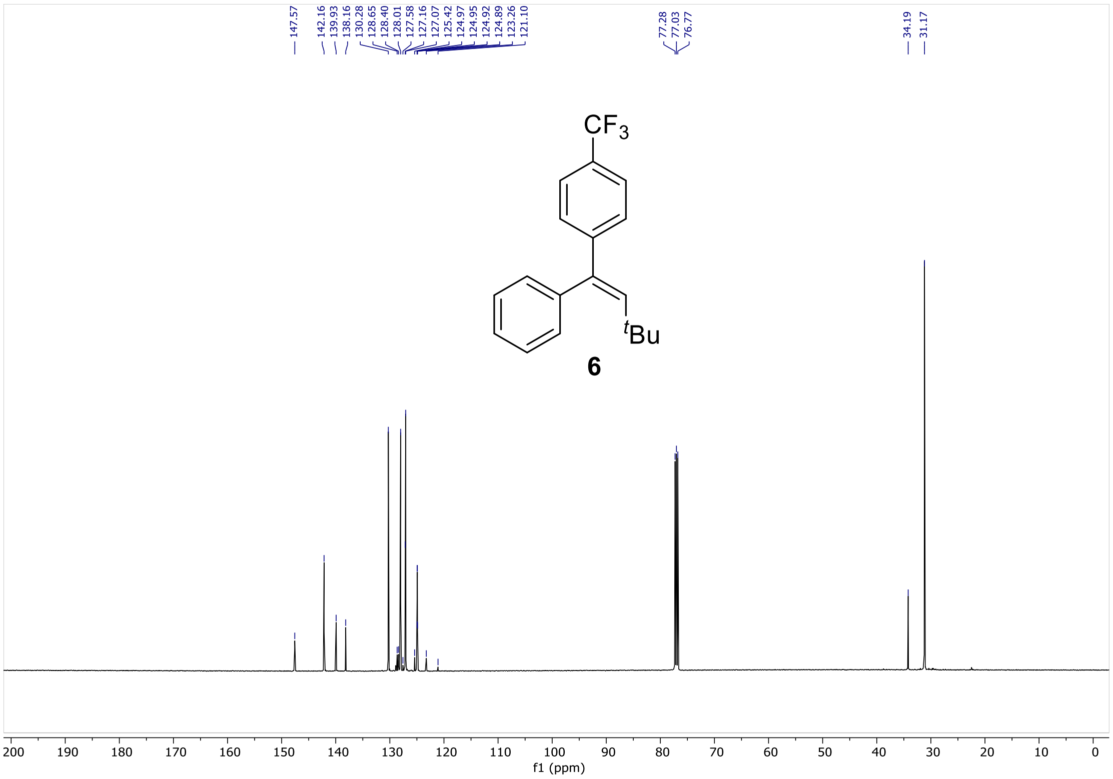

Supplementary Figure 35: ^{13}C NMR spectrum of compound **6**

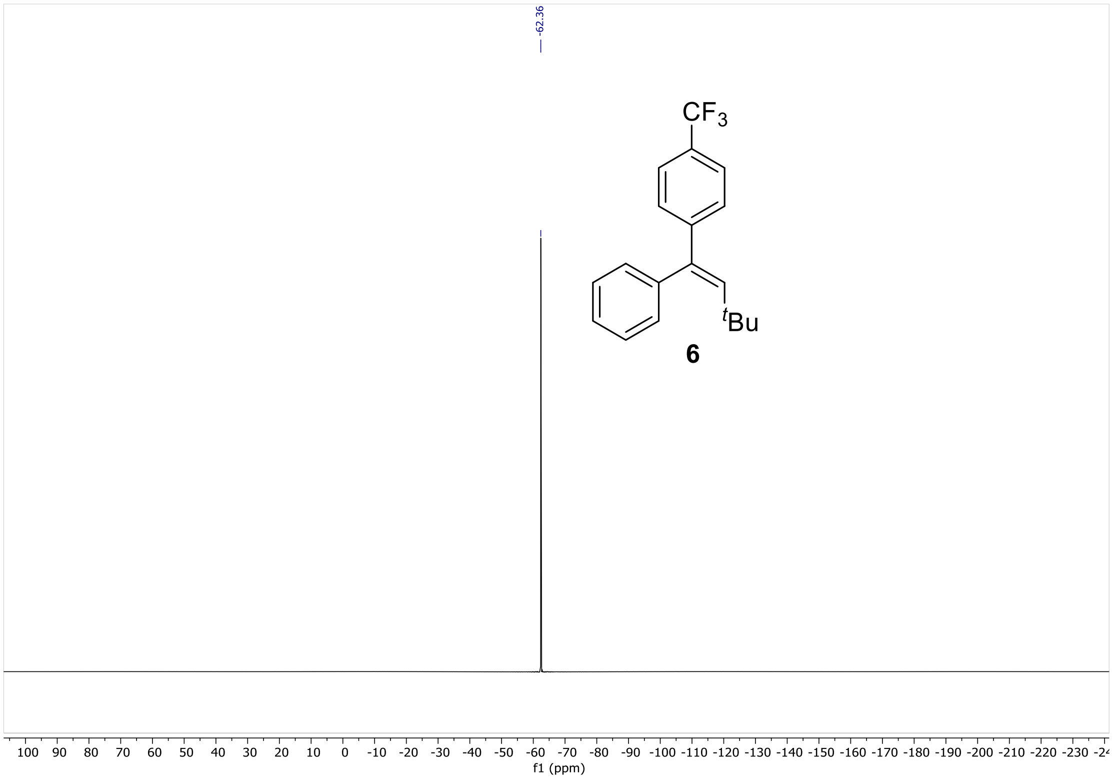

Supplementary Figure 36: ^{19}F NMR spectrum of compound **6**

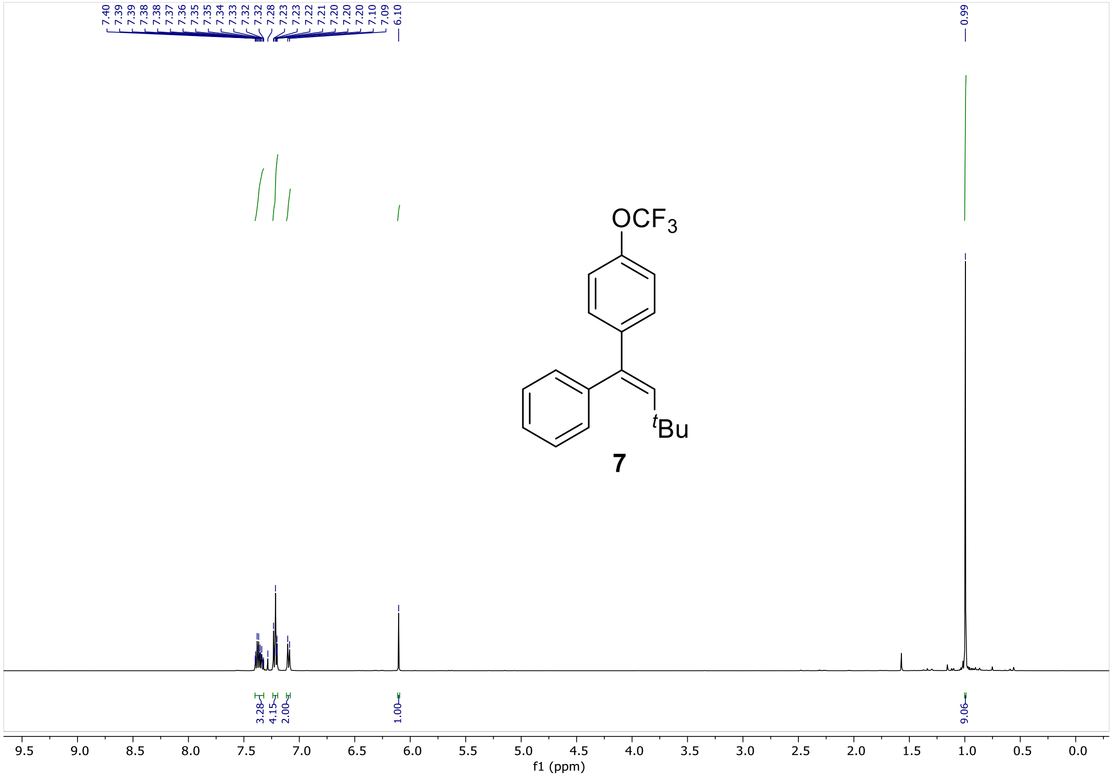

Supplementary Figure 37: ^{1}H NMR spectrum of compound **7**

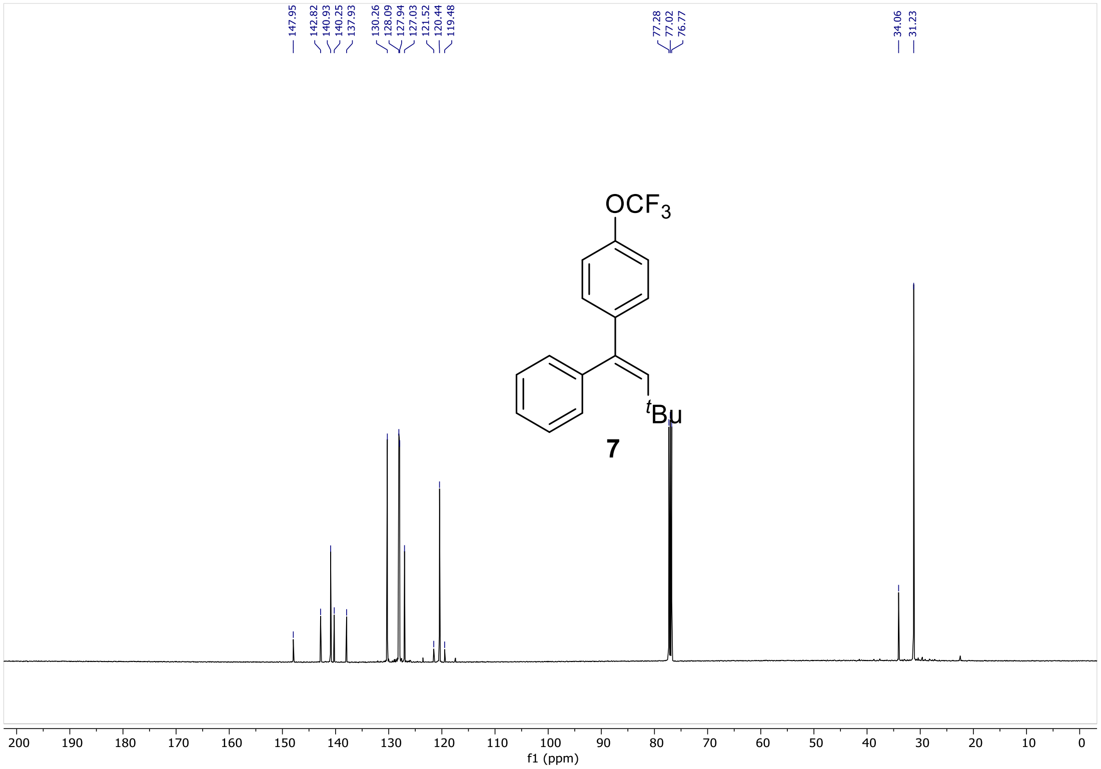

Supplementary Figure 38: ^{13}C NMR spectrum of compound **7**

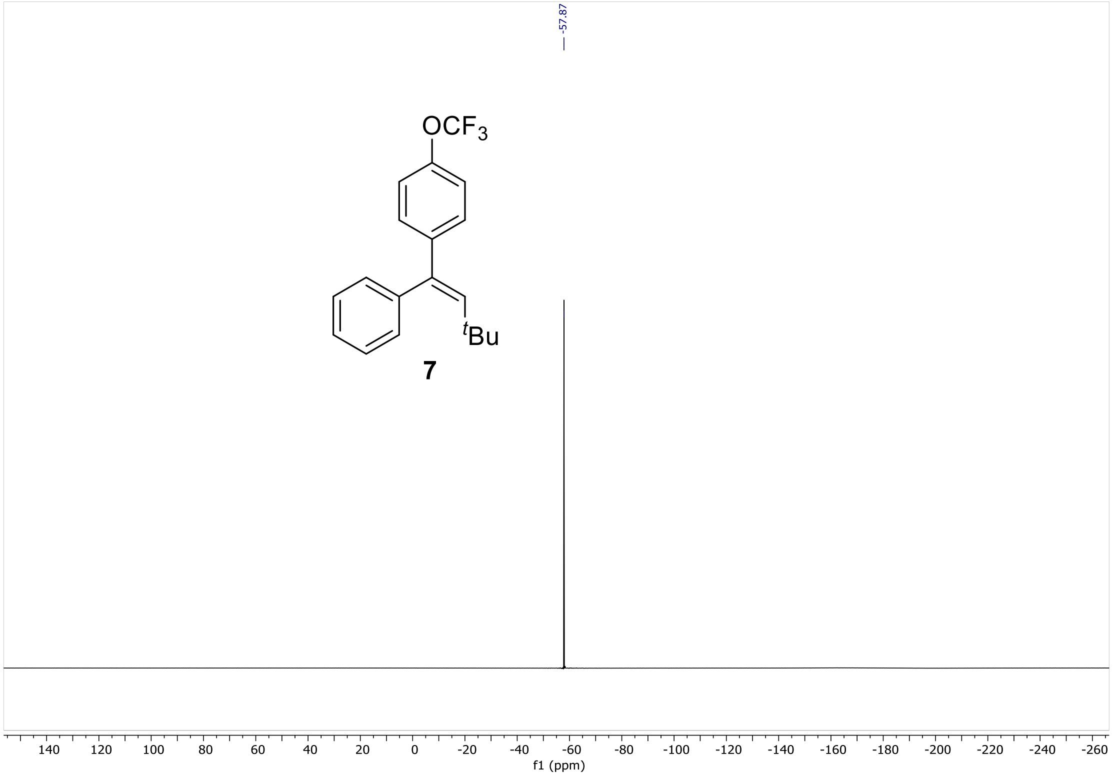

Supplementary Figure 39: ^{19}F NMR spectrum of compound **7**

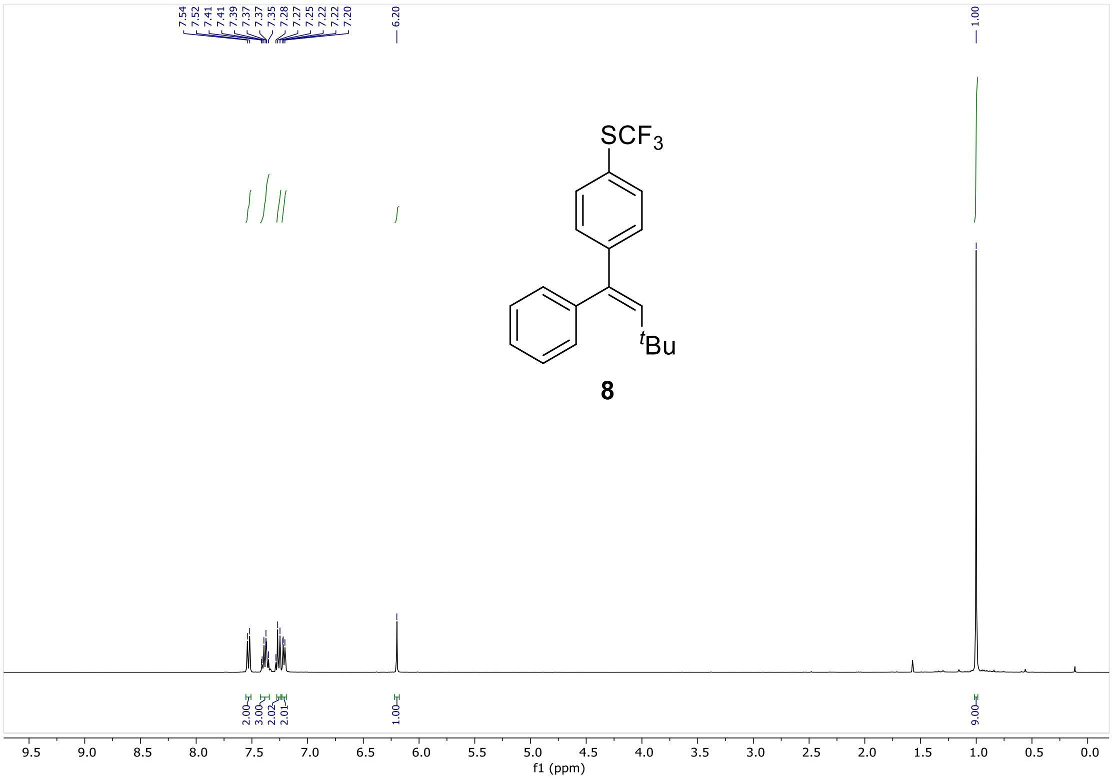

Supplementary Figure 40: ^{1}H NMR spectrum of compound **8**

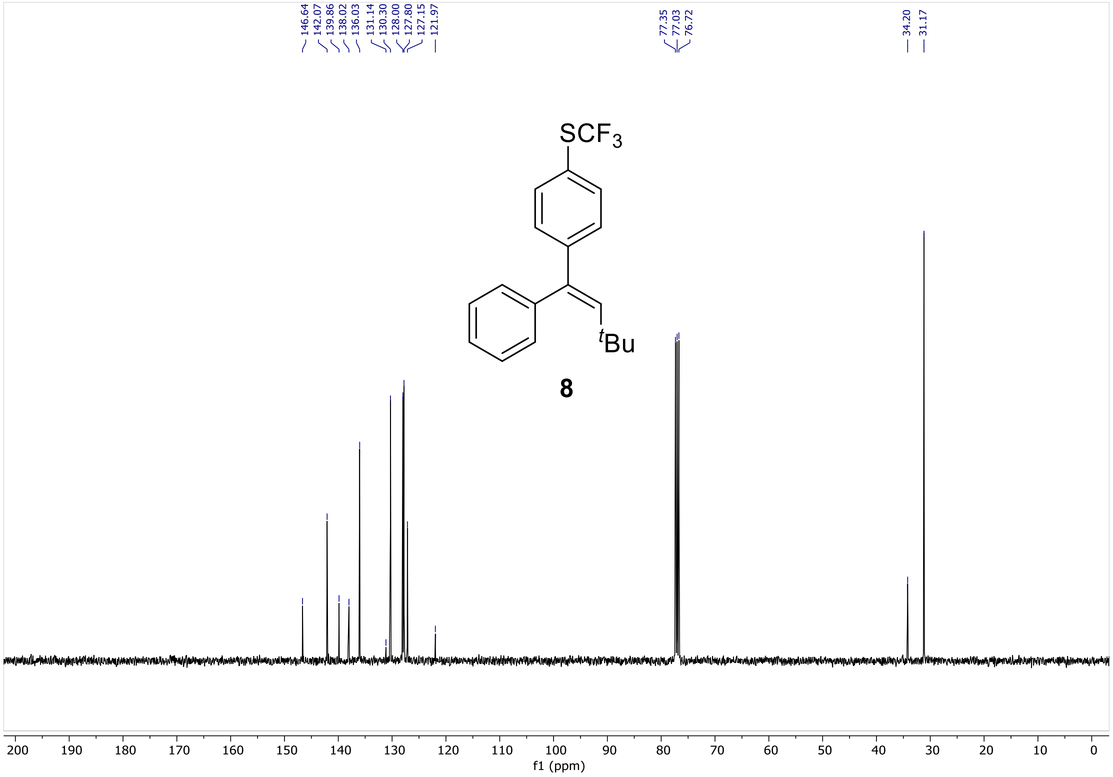

Supplementary Figure 41: ^{13}C NMR spectrum of compound **8**

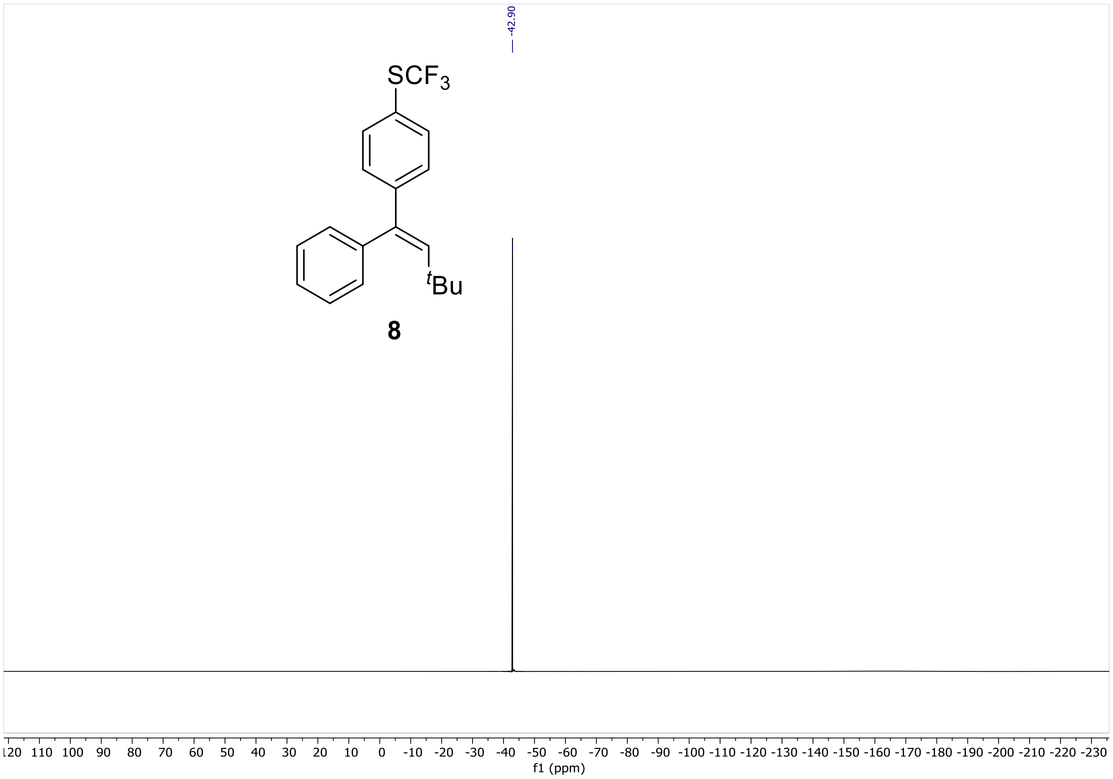

Supplementary Figure 42: ^{19}F NMR spectrum of compound **8**

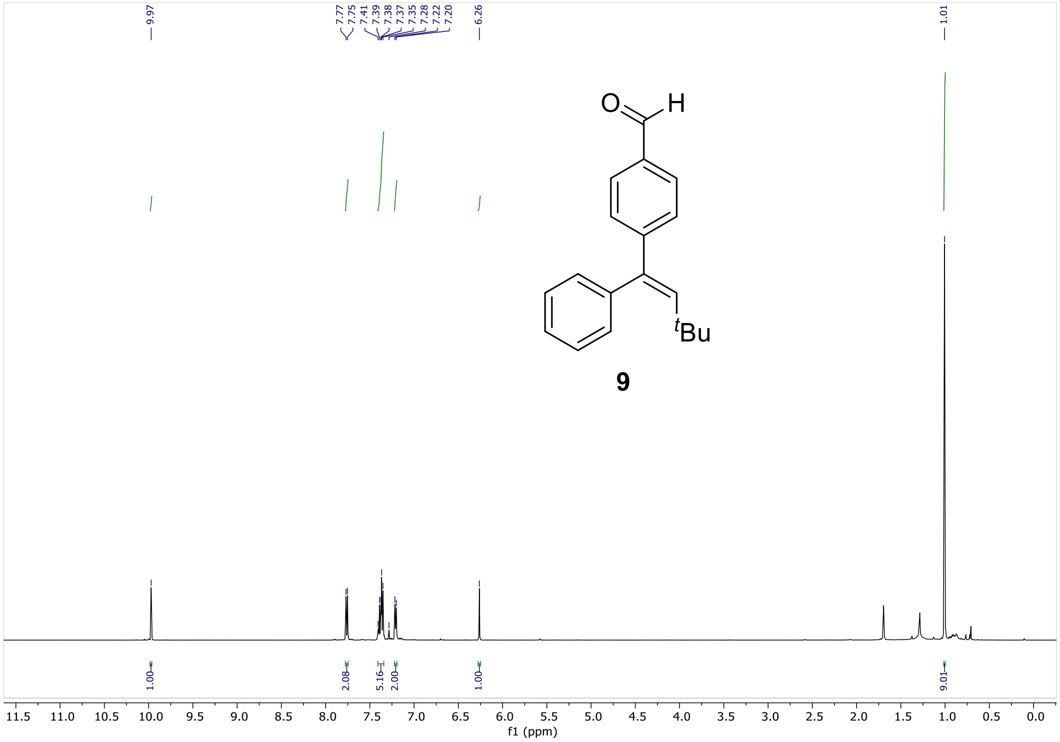

Supplementary Figure 43: ^{1}H NMR spectrum of compound **9**

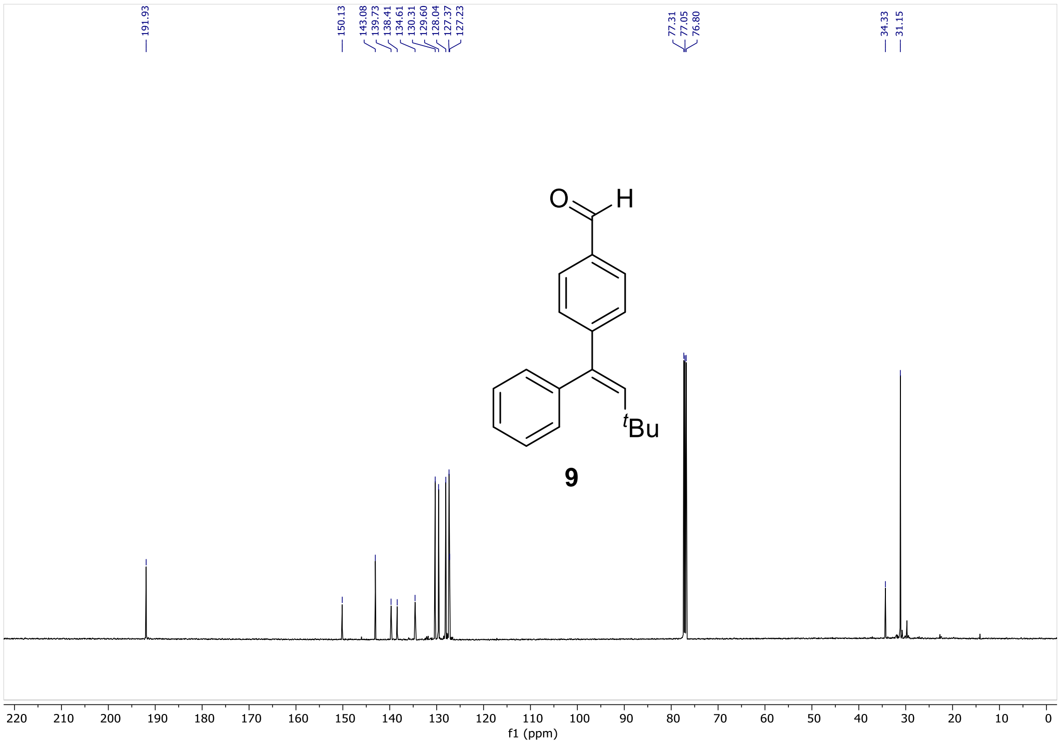

Supplementary Figure 44: ^{13}C NMR spectrum of compound **9**

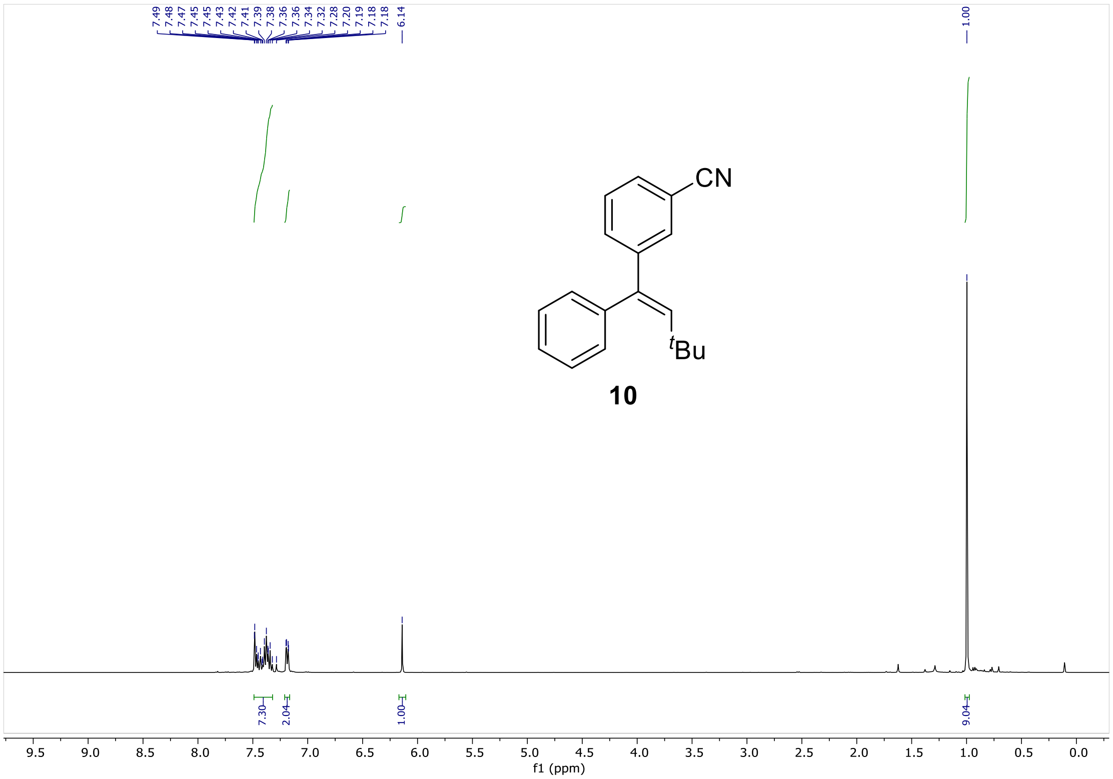

Supplementary Figure 45: ^{1}H NMR spectrum of compound **10**

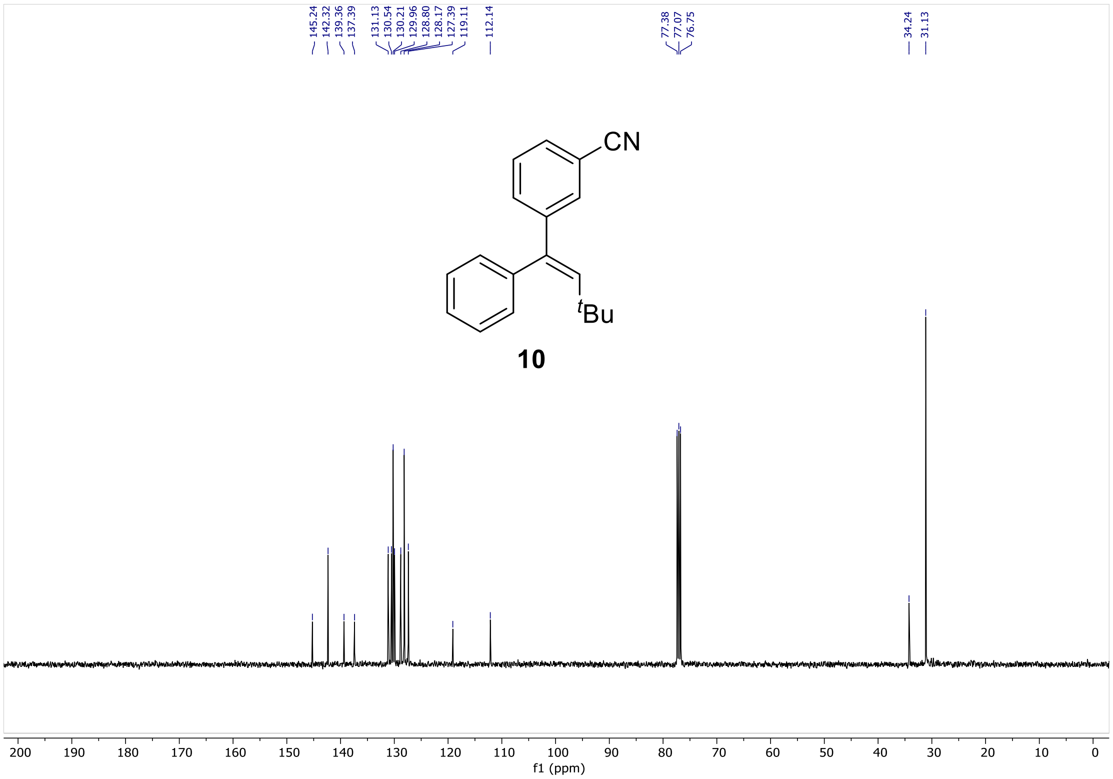

Supplementary Figure 46: ^{13}C NMR spectrum of compound **10**

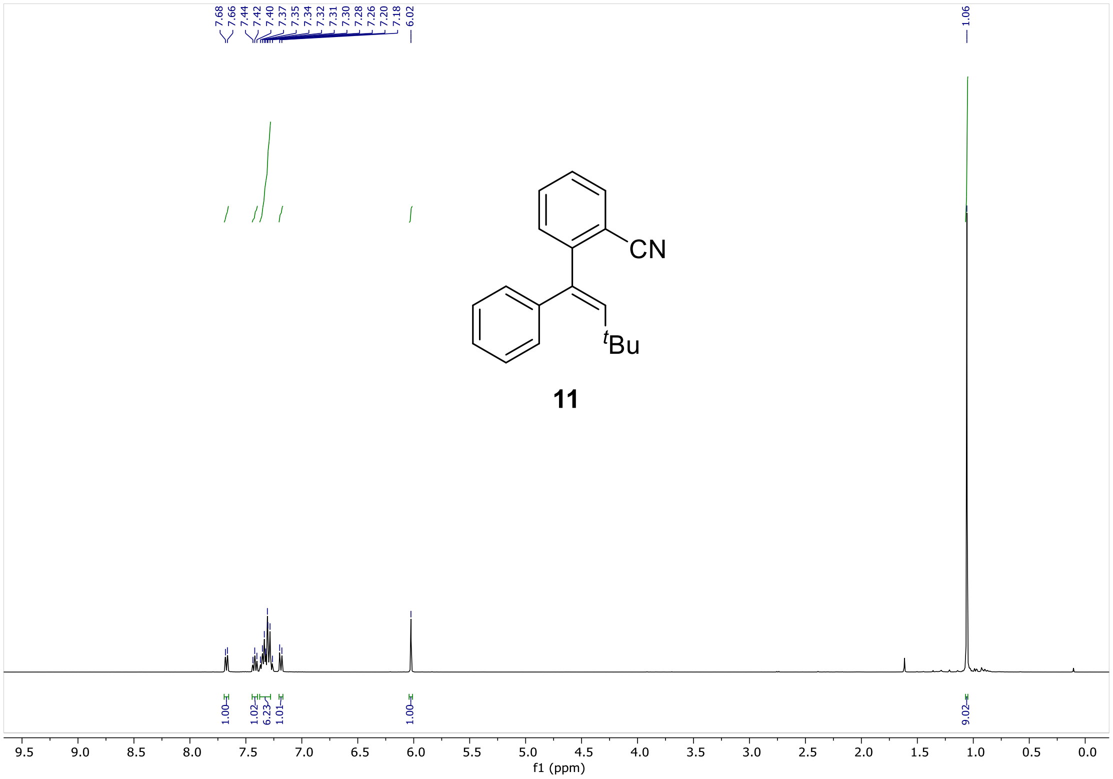

Supplementary Figure 47: ^{1}H NMR spectrum of compound **11**

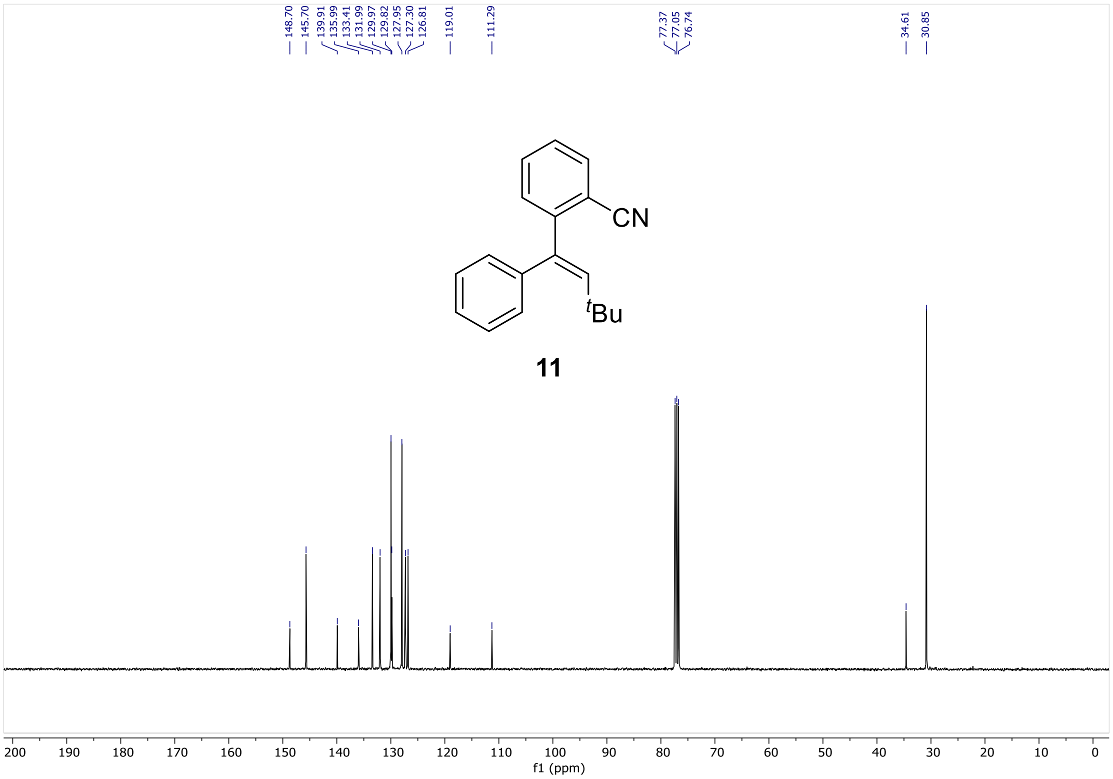

Supplementary Figure 48: ^{13}C NMR spectrum of compound **11**

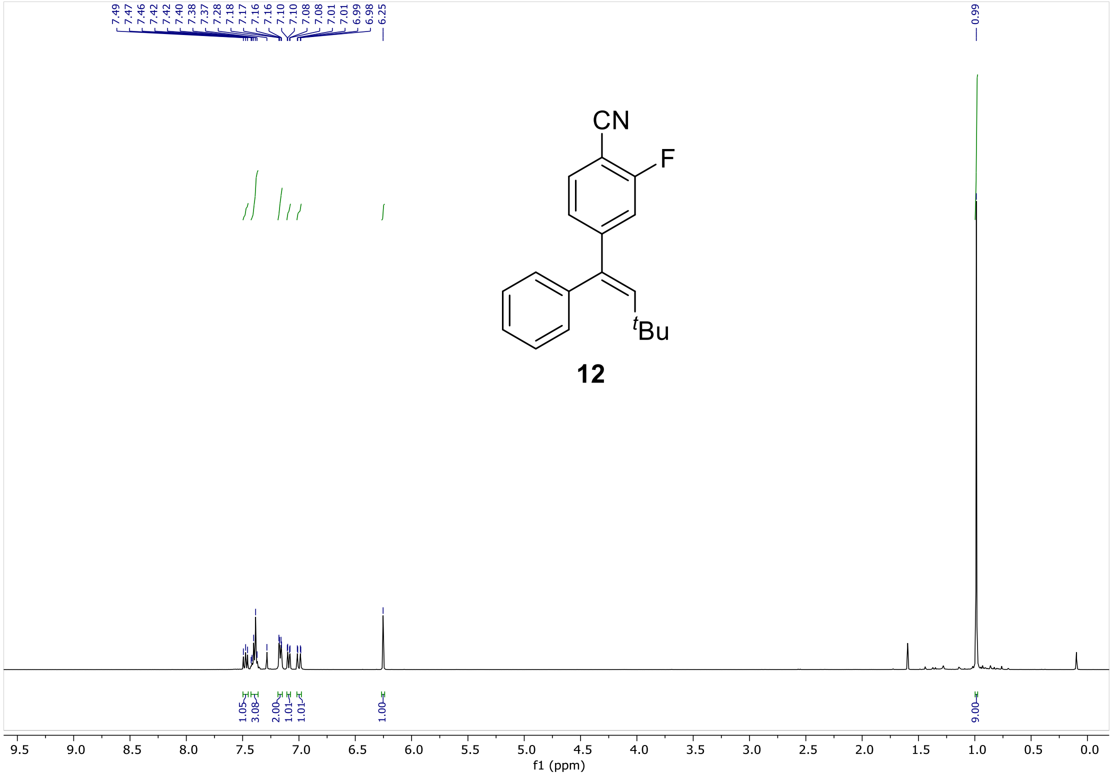

Supplementary Figure 49: ^{1}H NMR spectrum of compound **12**

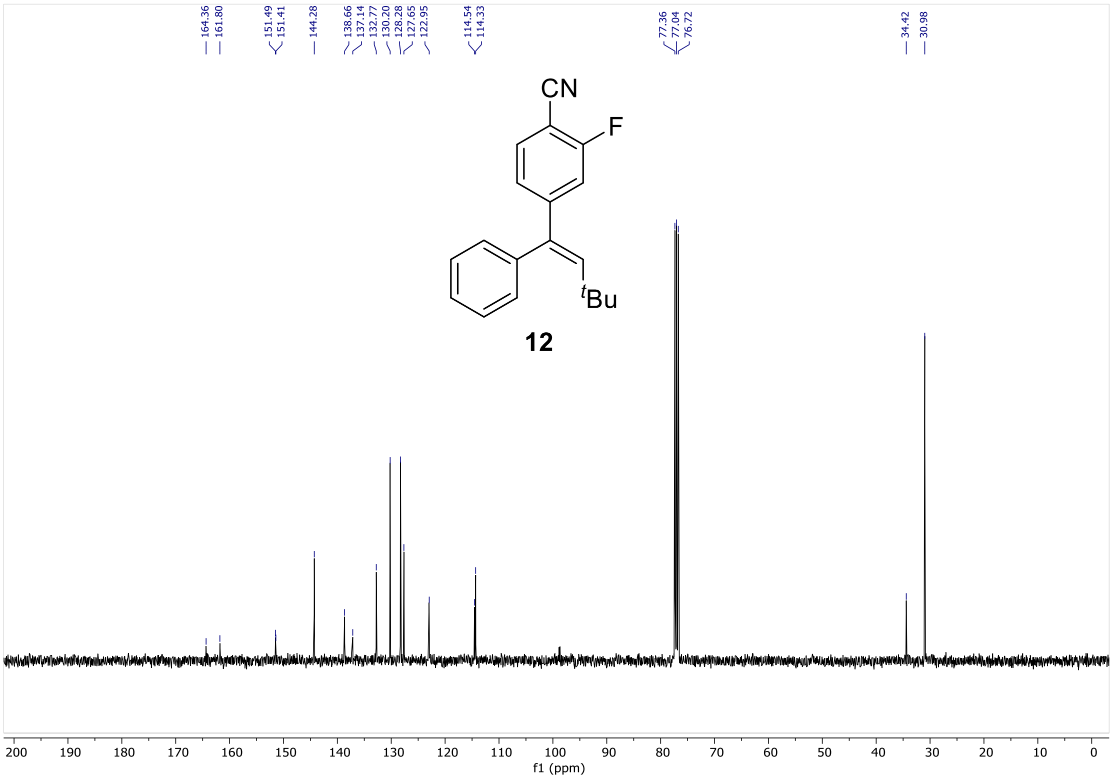

Supplementary Figure 50: ^{13}C NMR spectrum of compound **12**

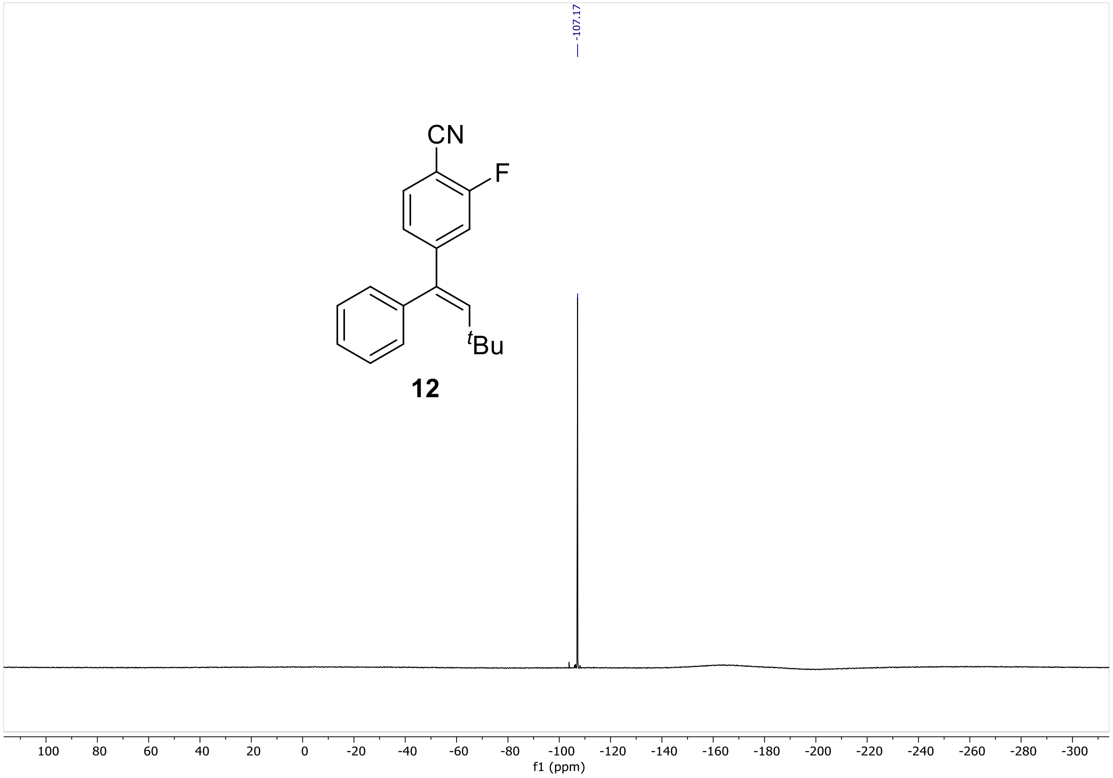

Supplementary Figure 51: ^{19}F NMR spectrum of compound **12**

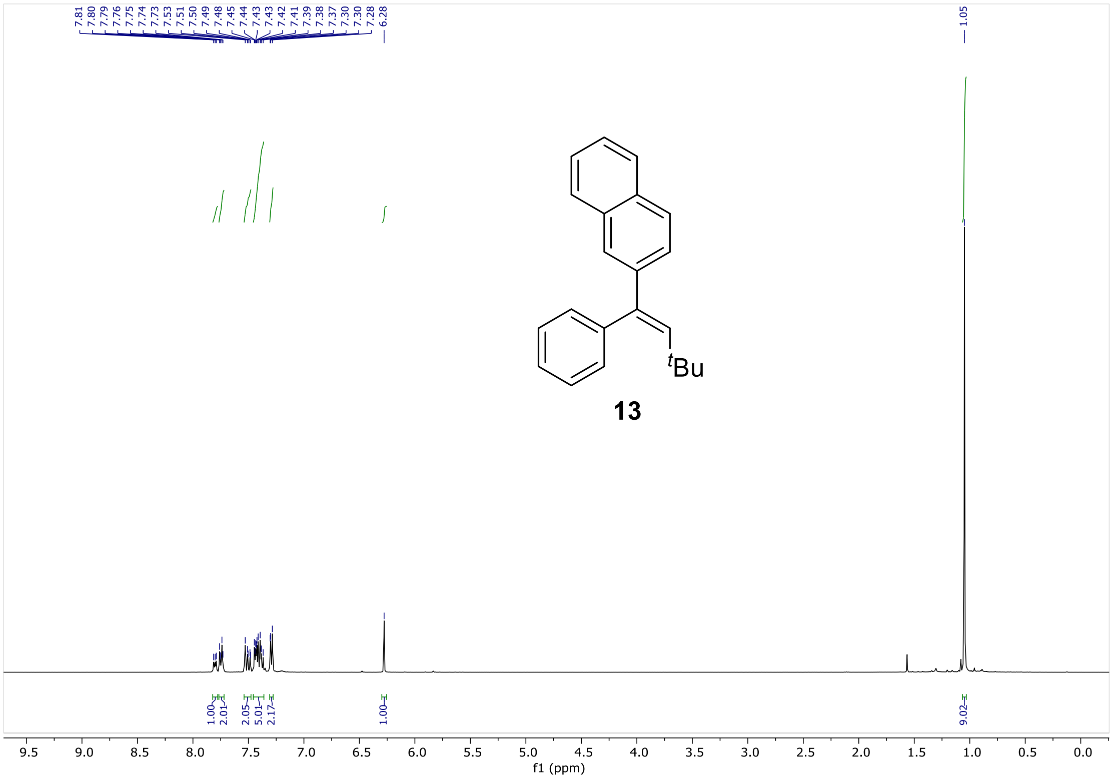

Supplementary Figure 52: ^{1}H NMR spectrum of compound **13**

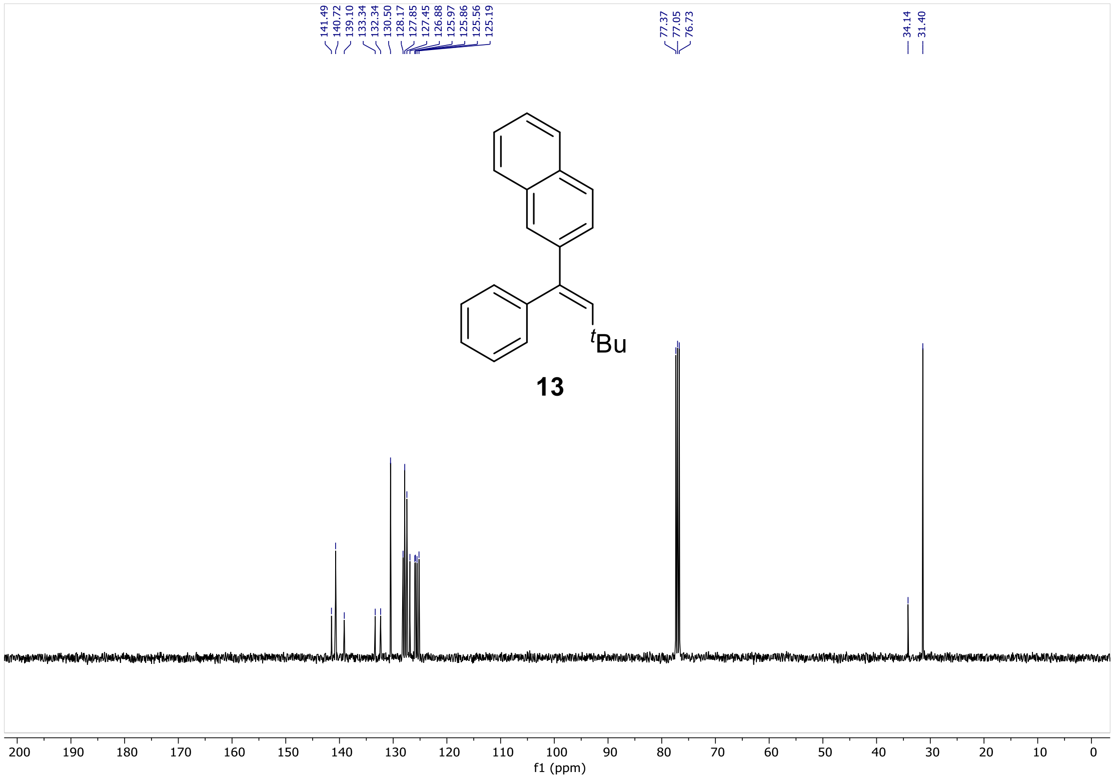

Supplementary Figure 53: ^{13}C NMR spectrum of compound **13**

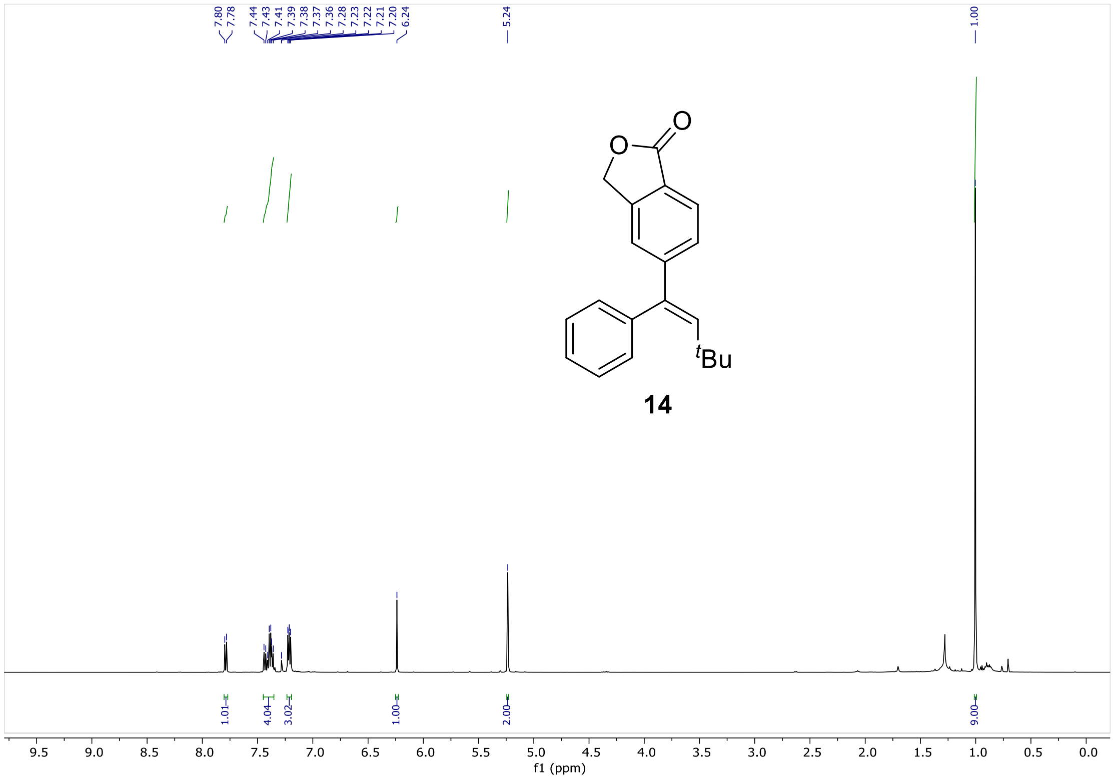

Supplementary Figure 54: ^{1}H NMR spectrum of compound 1**4**

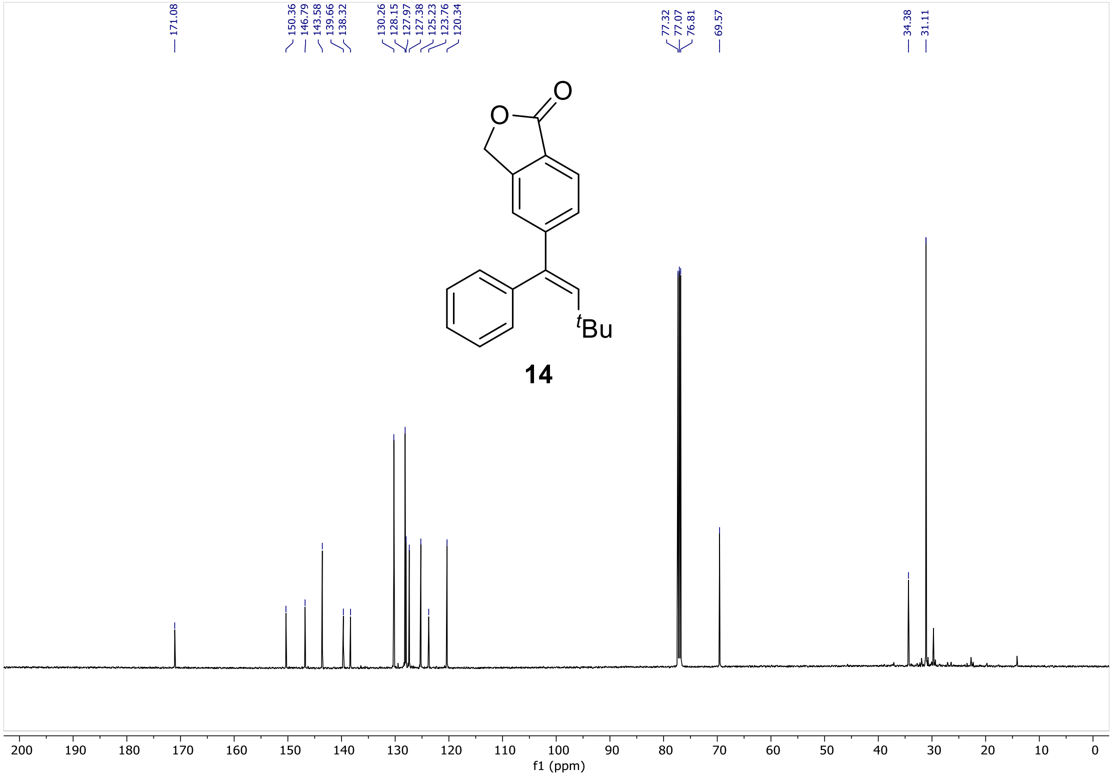

Supplementary Figure 55: ^{13}C NMR spectrum of compound 1**4**

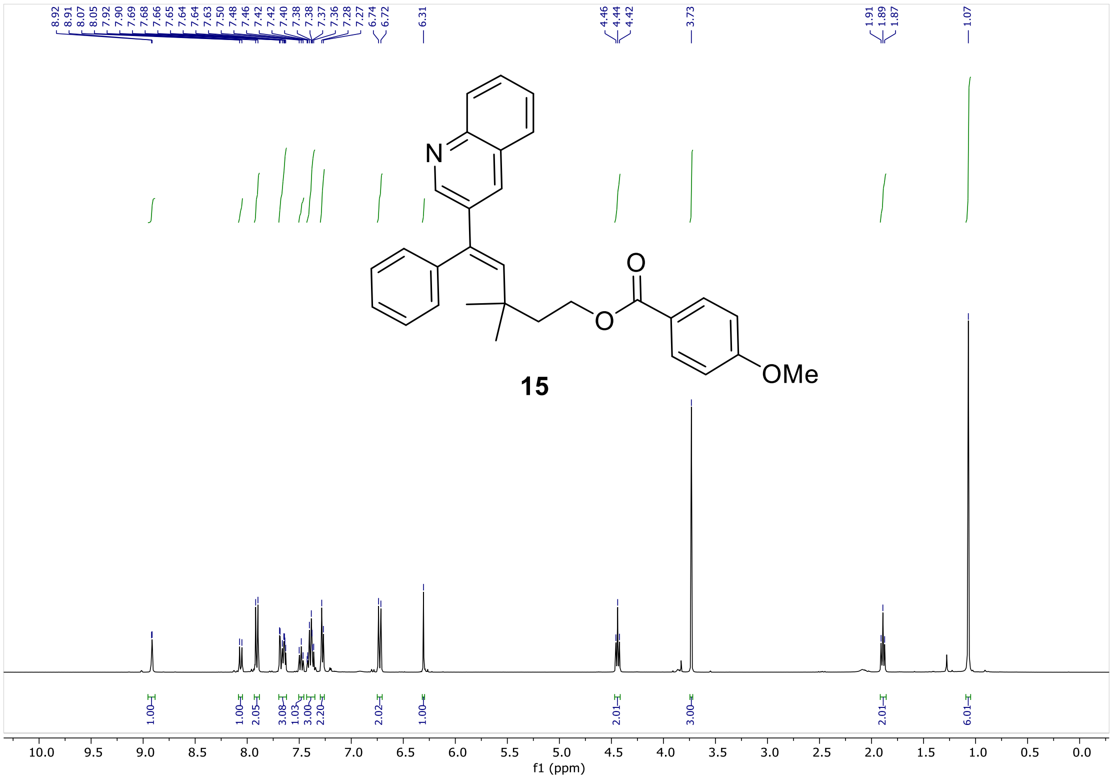

Supplementary Figure 56: ^{1}H NMR spectrum of compound **15**

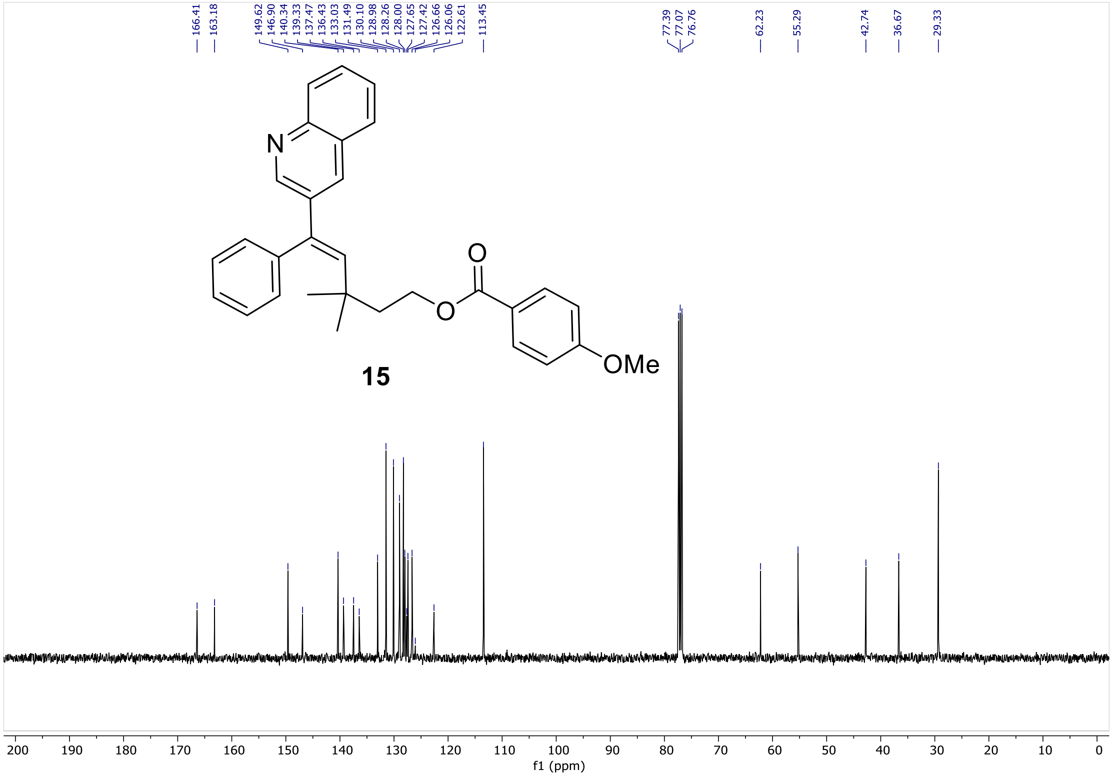

Supplementary Figure 57: ^{13}C NMR spectrum of compound **15**

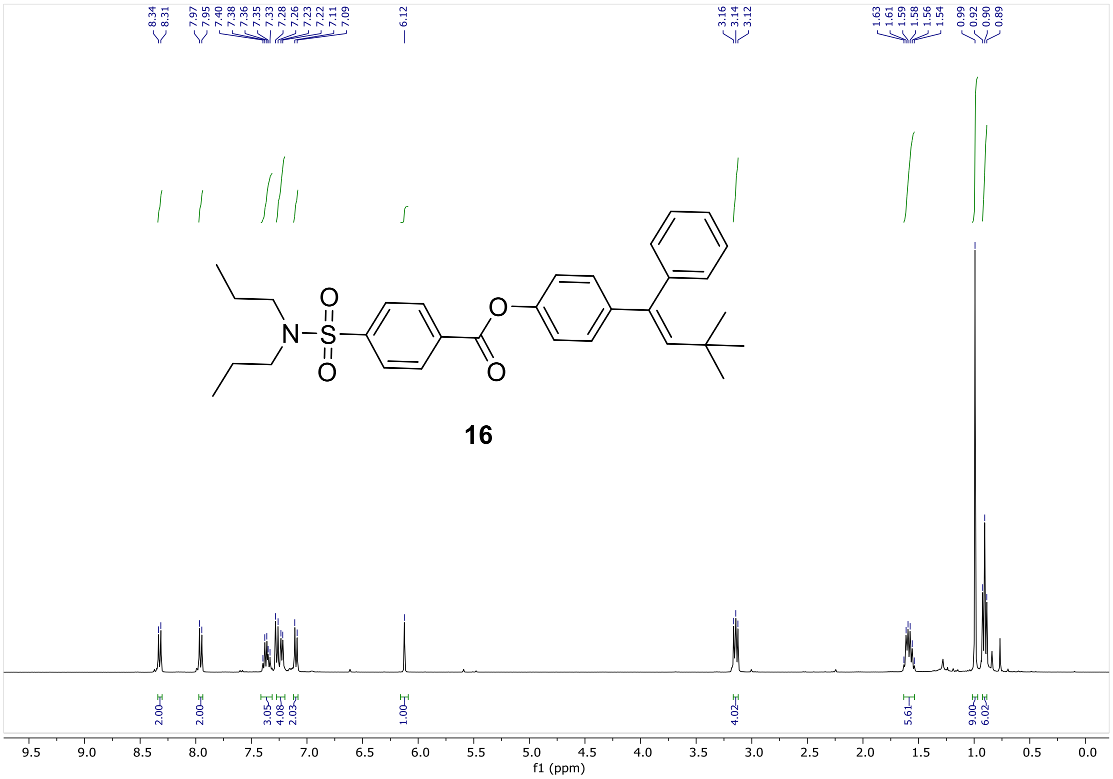

Supplementary Figure 58: ^{1}H NMR spectrum of compound **16**

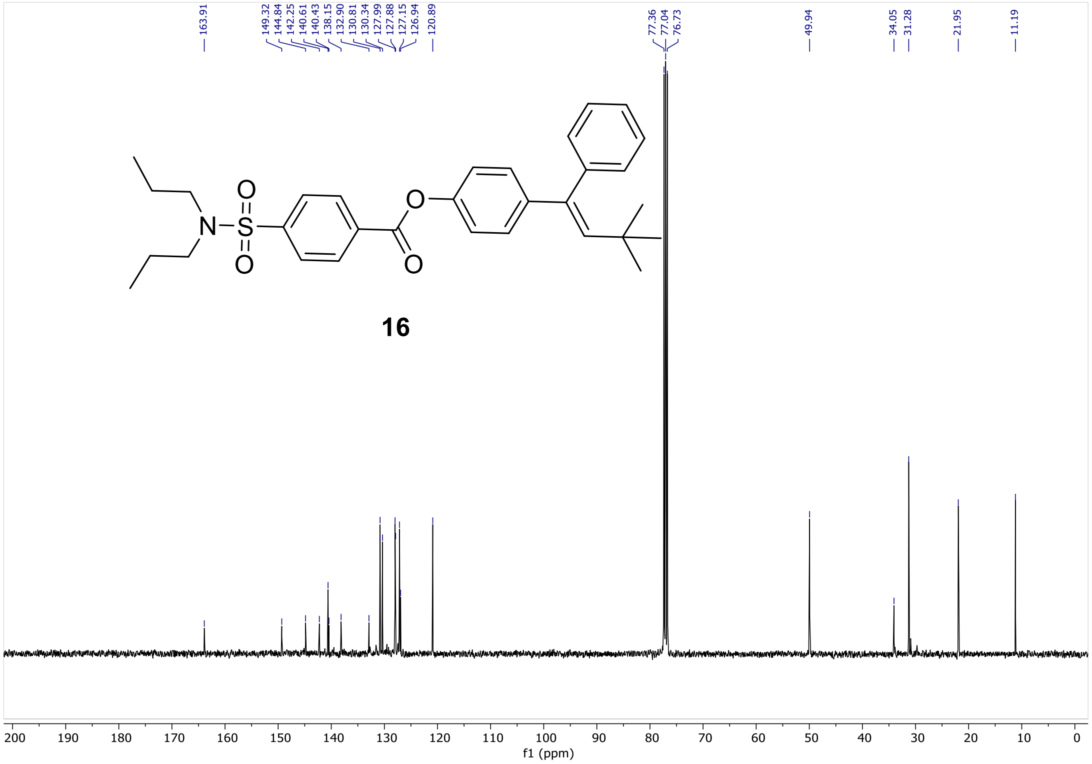

Supplementary Figure 59: ^{13}C NMR spectrum of compound **16**

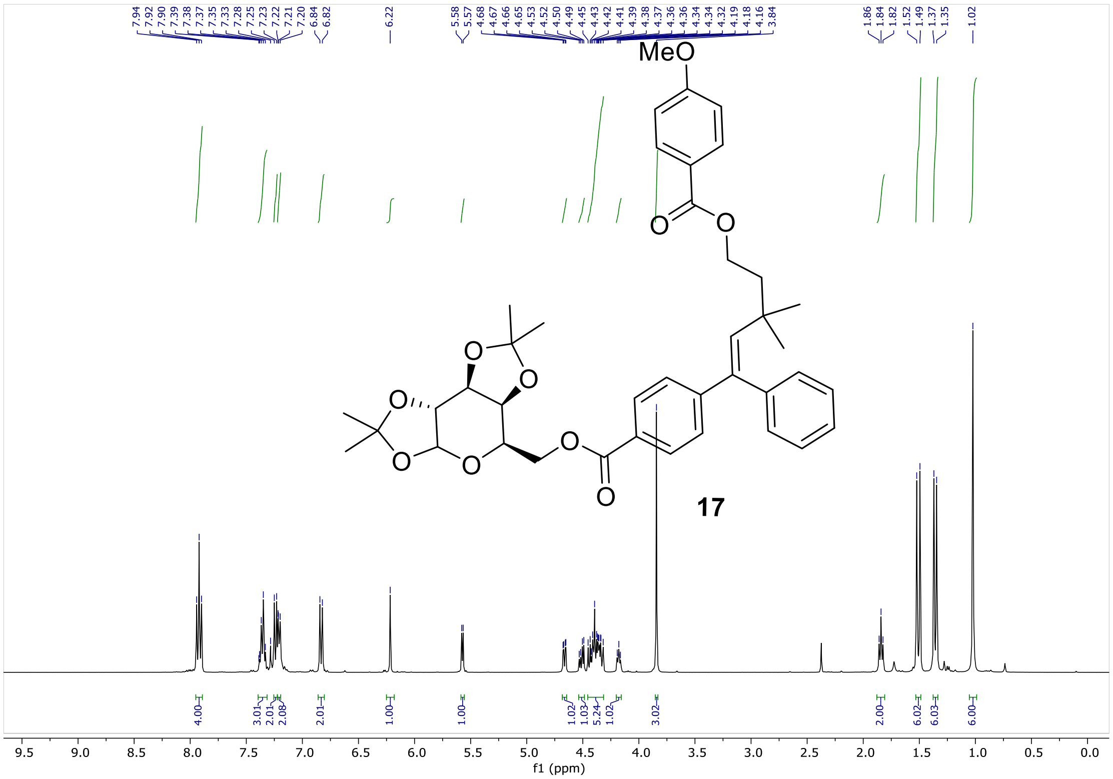

Supplementary Figure 60: ^{1}H NMR spectrum of compound **17**

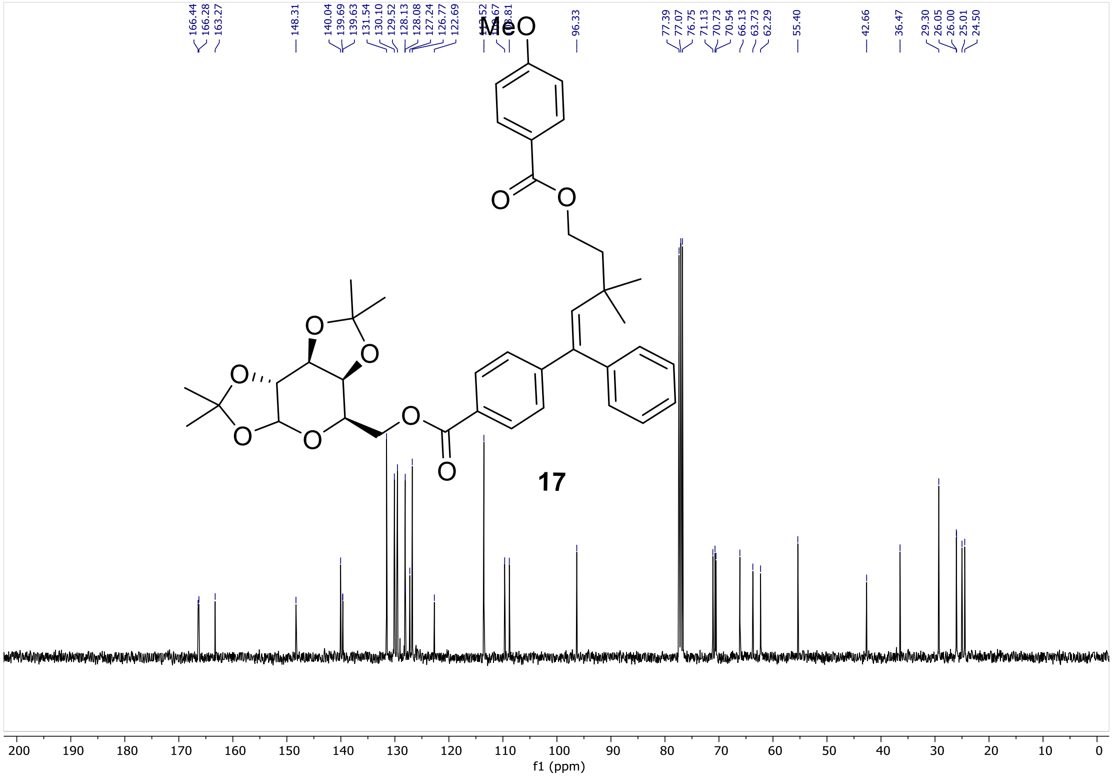

Supplementary Figure 61: ^{13}C NMR spectrum of compound **17**

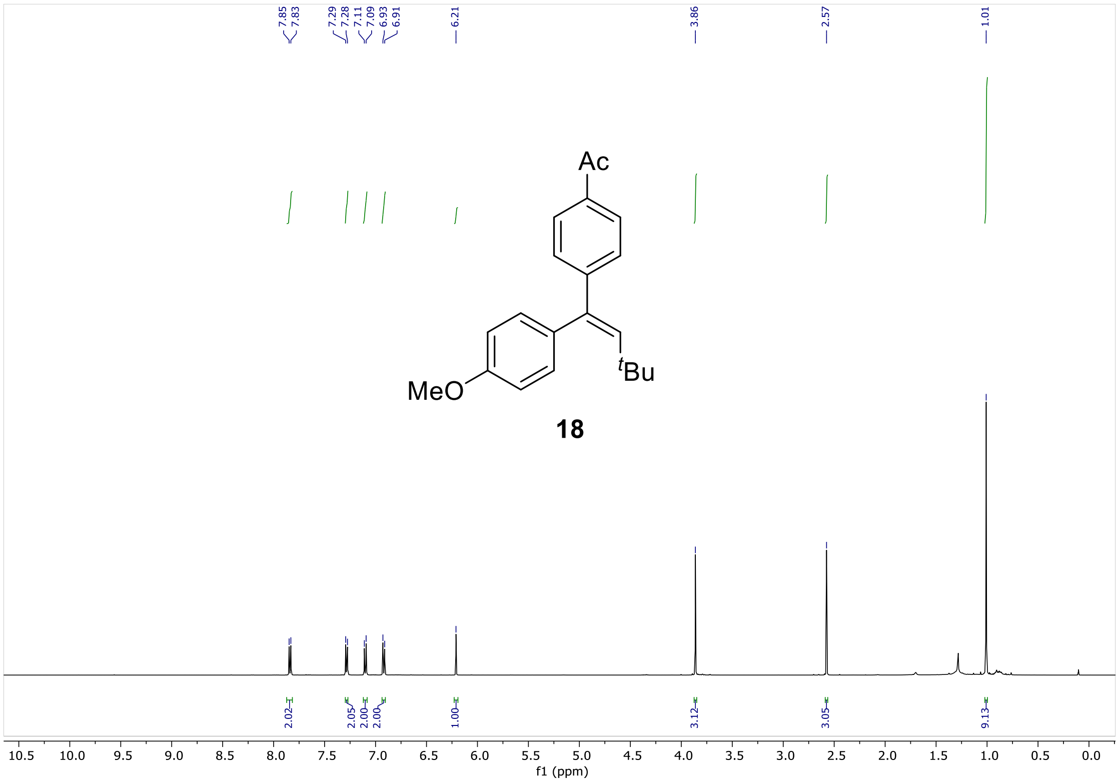

Supplementary Figure 62: ^{1}H NMR spectrum of compound **18**

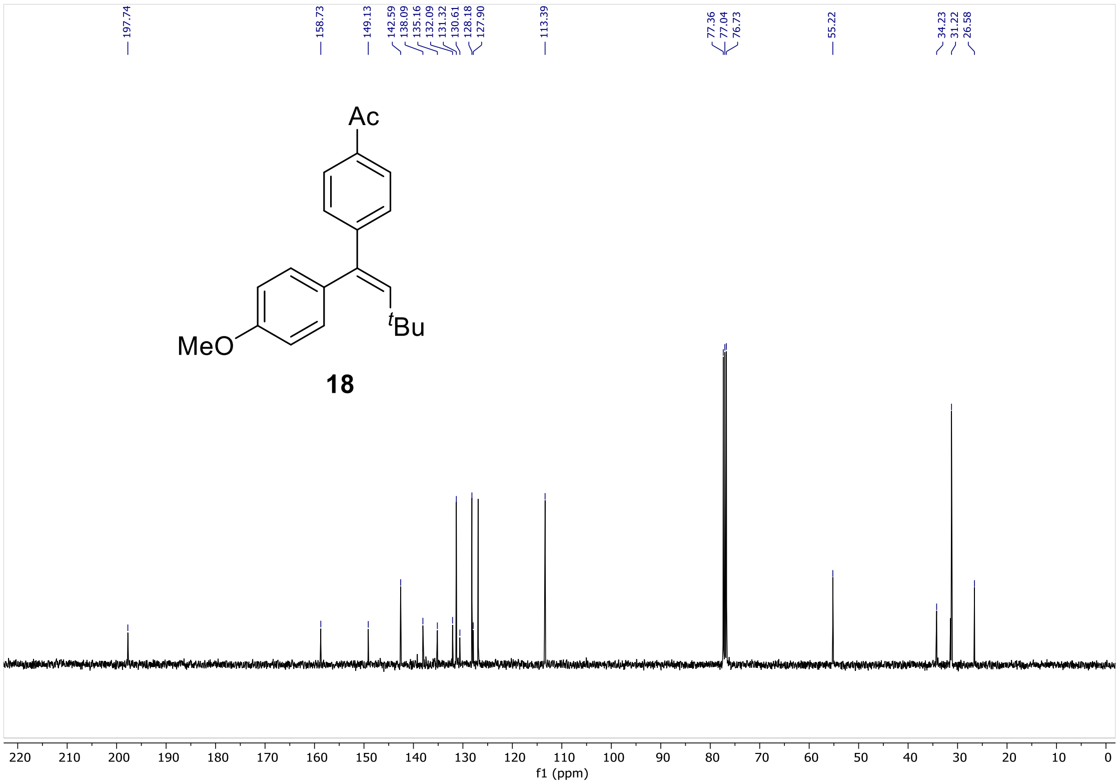

Supplementary Figure 63: ^{13}C NMR spectrum of compound **18**

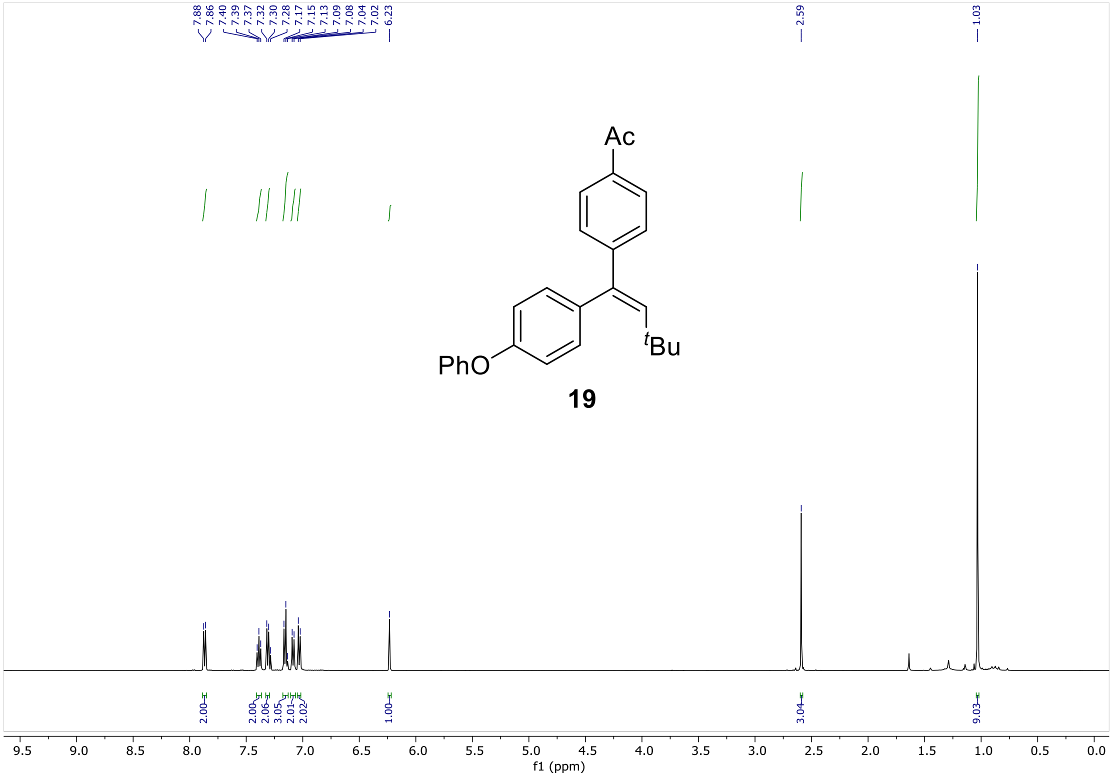

Supplementary Figure 64: ^{1}H NMR spectrum of compound **19**

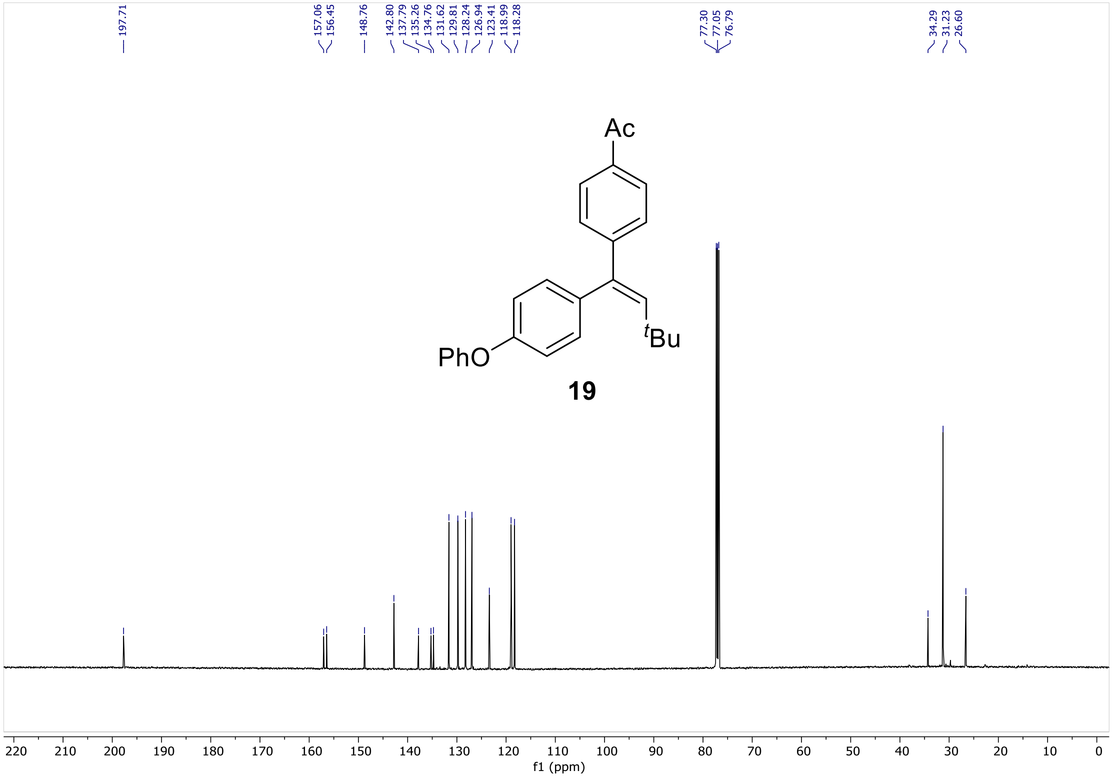

Supplementary Figure 65: ^{13}C NMR spectrum of compound **19**

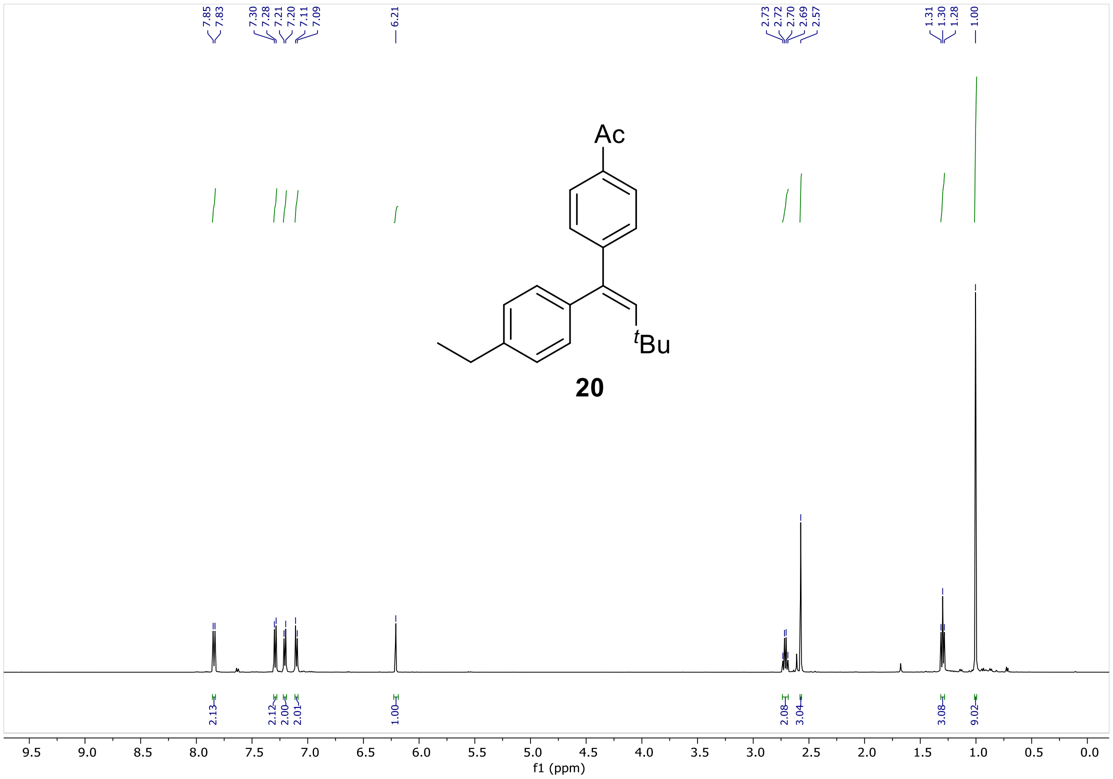

Supplementary Figure 66: ^{1}H NMR spectrum of compound **20**

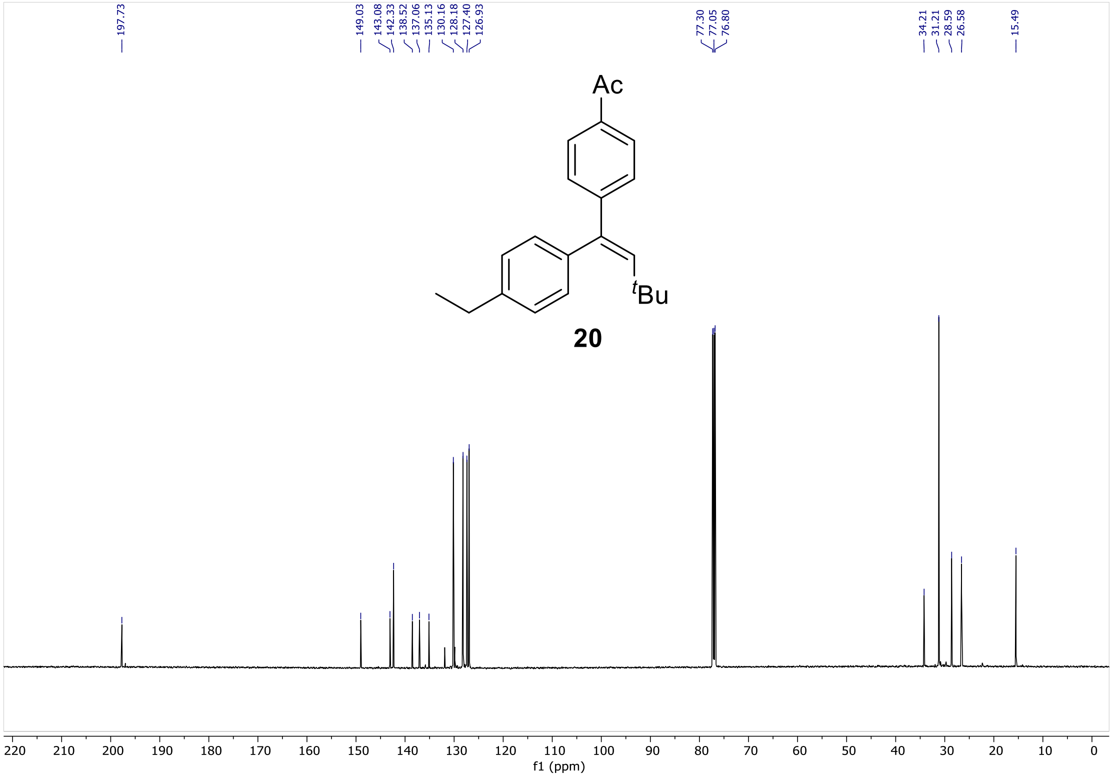

Supplementary Figure 67: ^{13}C NMR spectrum of compound **20**

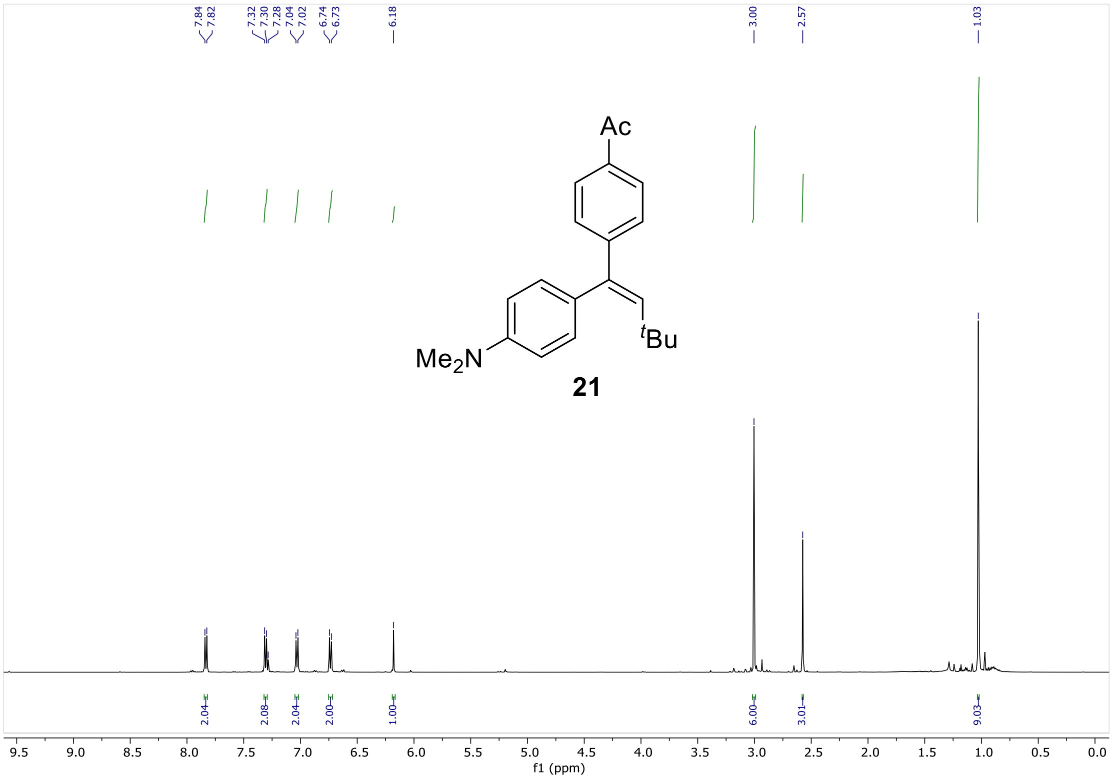

Supplementary Figure 68: ^{1}H NMR spectrum of compound **21**

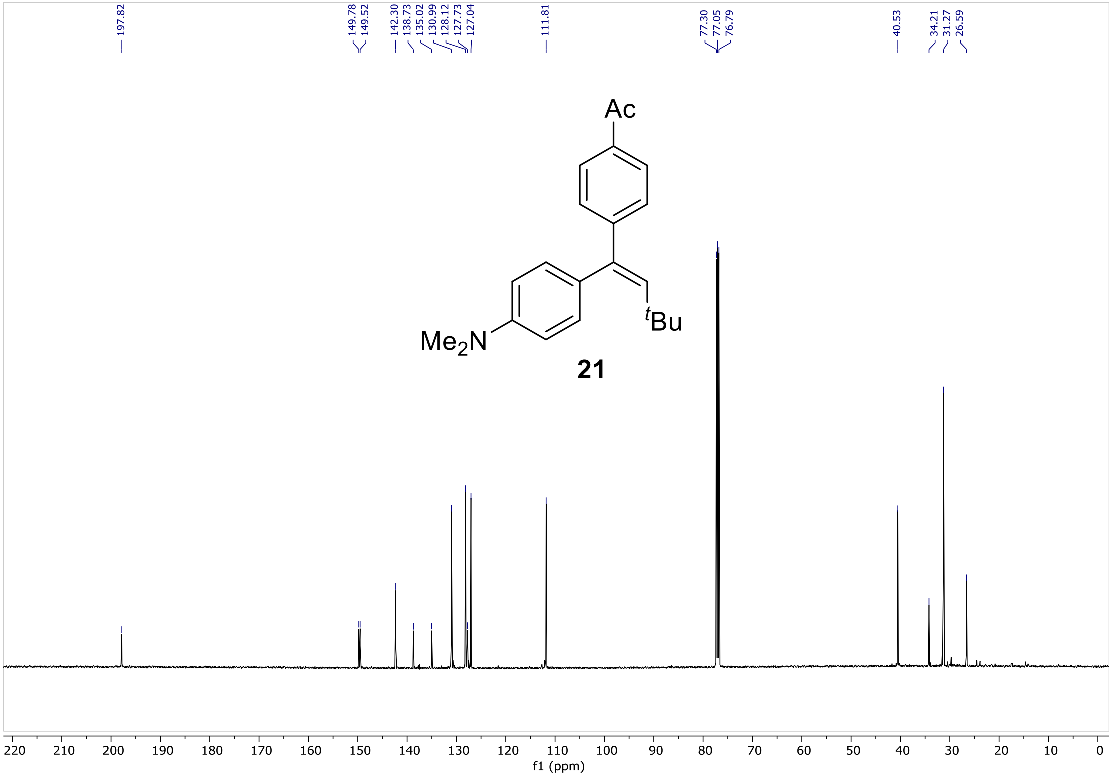

Supplementary Figure 69: ^{13}C NMR spectrum of compound **21**

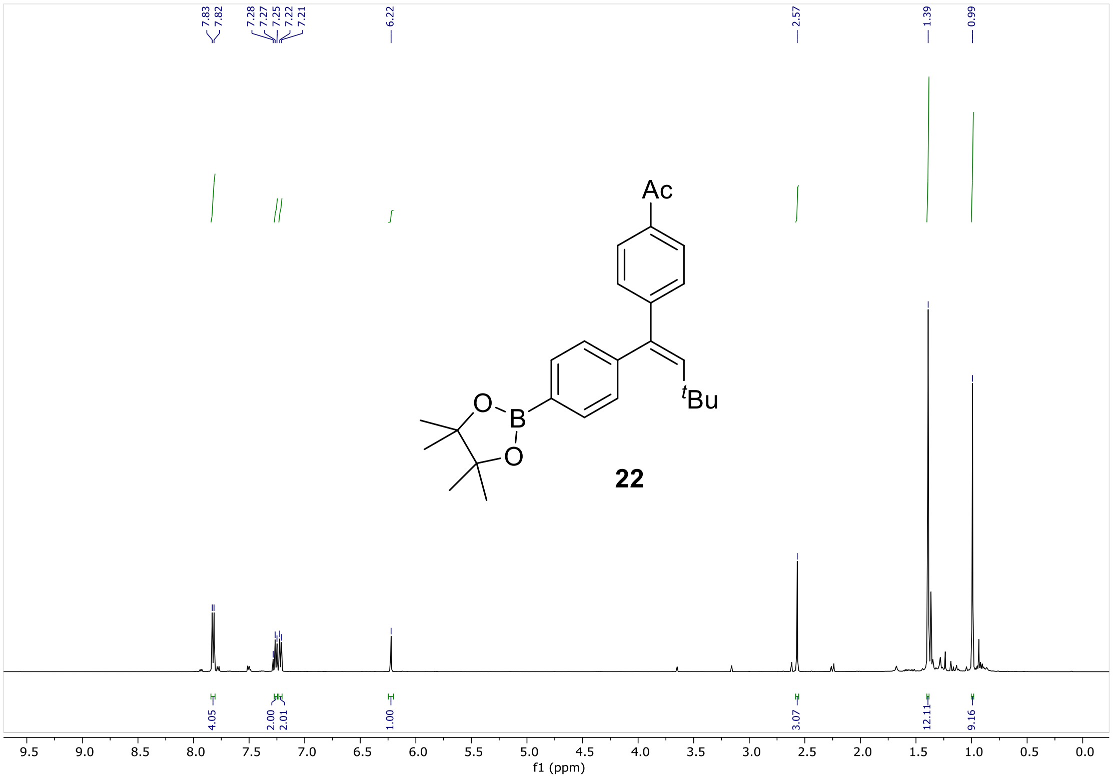

Supplementary Figure 70: ^{1}H NMR spectrum of compound **22**

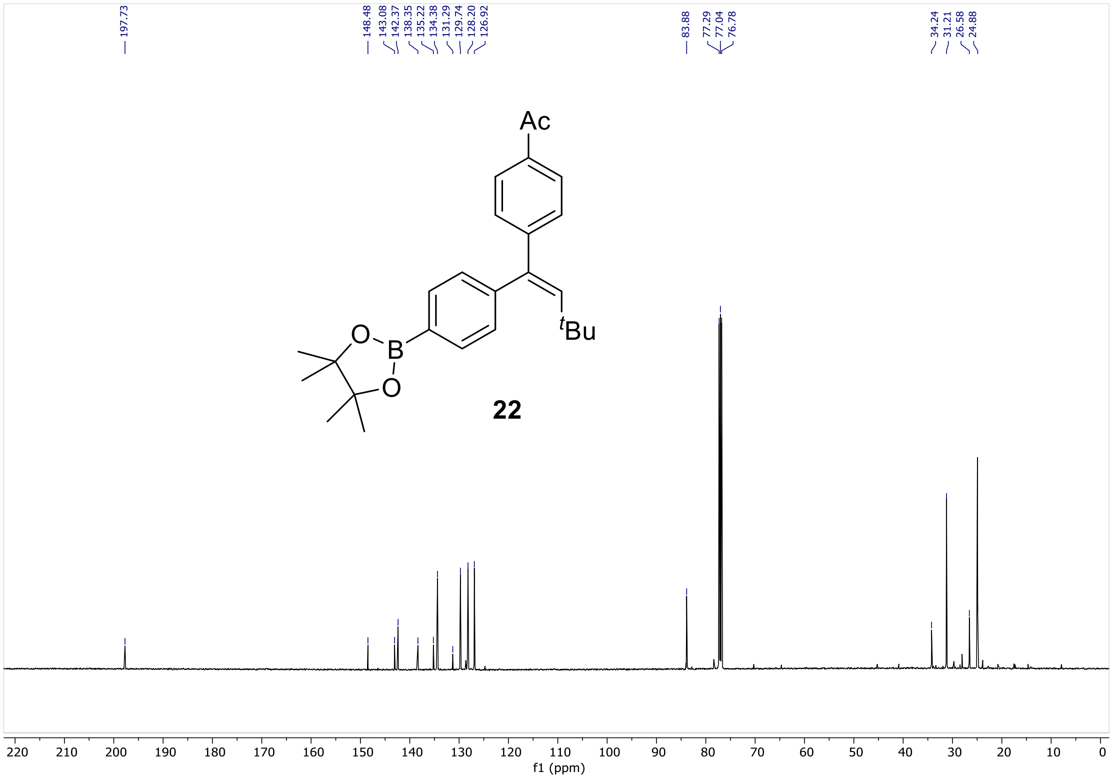

Supplementary Figure 71: ^{13}C NMR spectrum of compound **22**

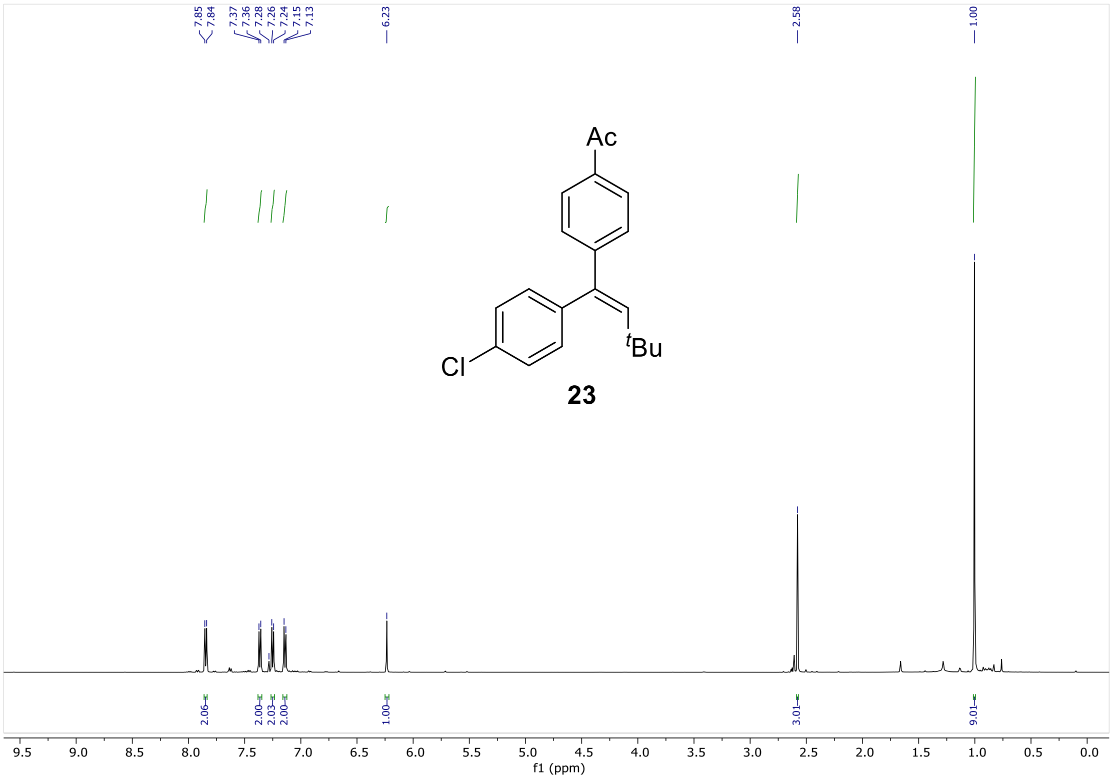

Supplementary Figure 72: ^{1}H NMR spectrum of compound **23**

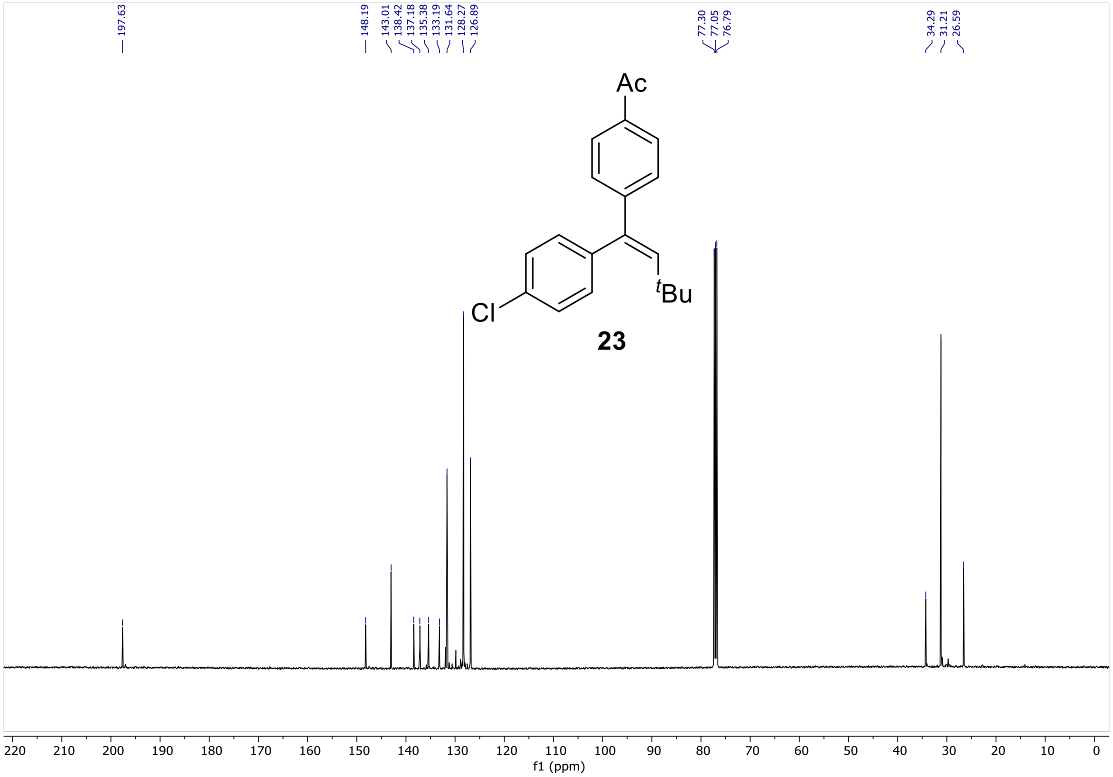

Supplementary Figure 73: ^{13}C NMR spectrum of compound **23**

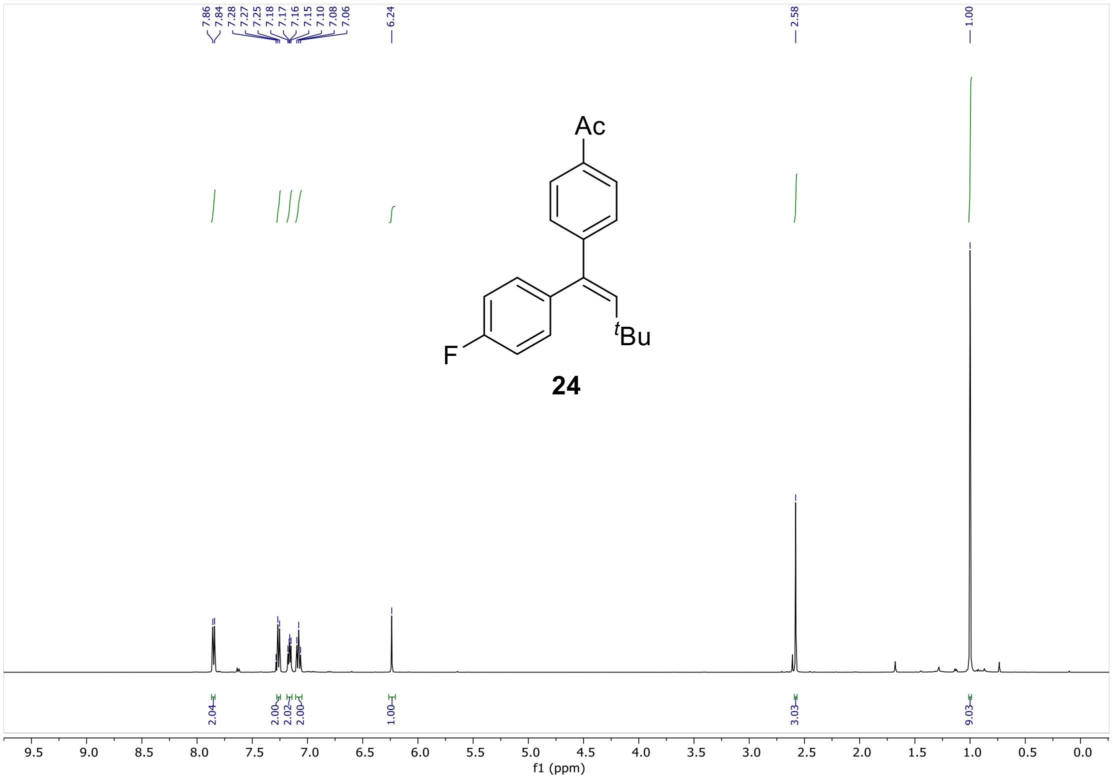

Supplementary Figure 74: ^{1}H NMR spectrum of compound **24**

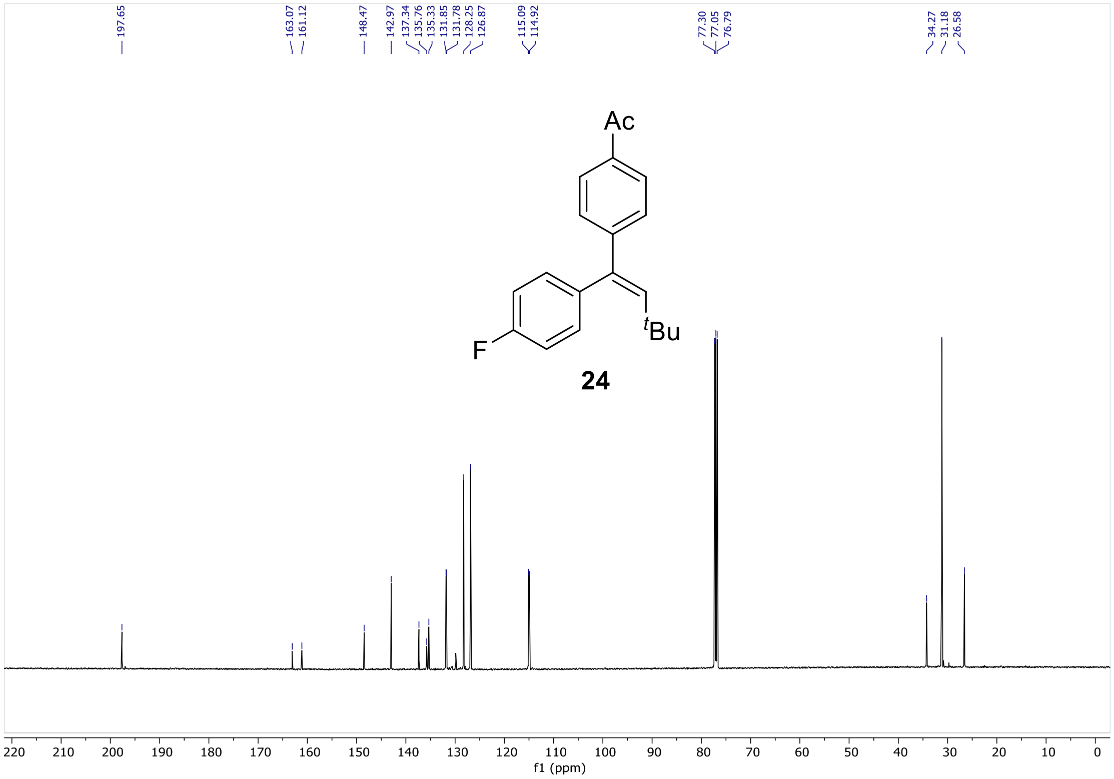

Supplementary Figure 75: ^{13}C NMR spectrum of compound **24**

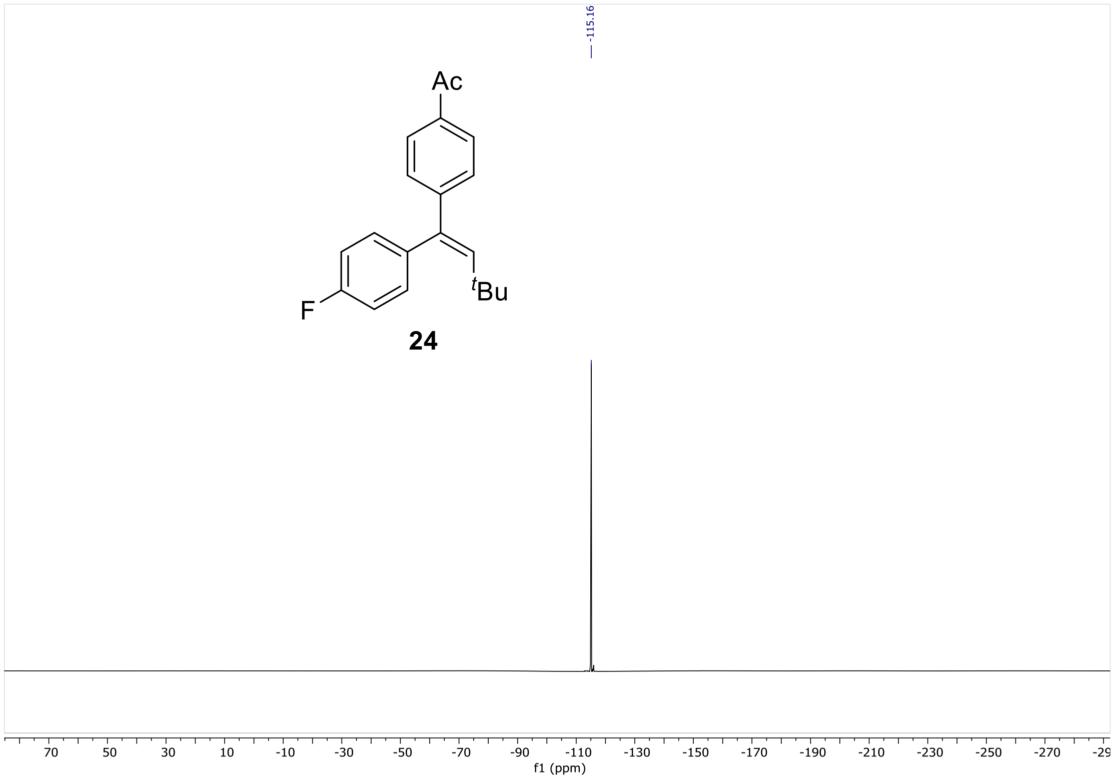

Supplementary Figure 76: ^{19}F NMR spectrum of compound **24**

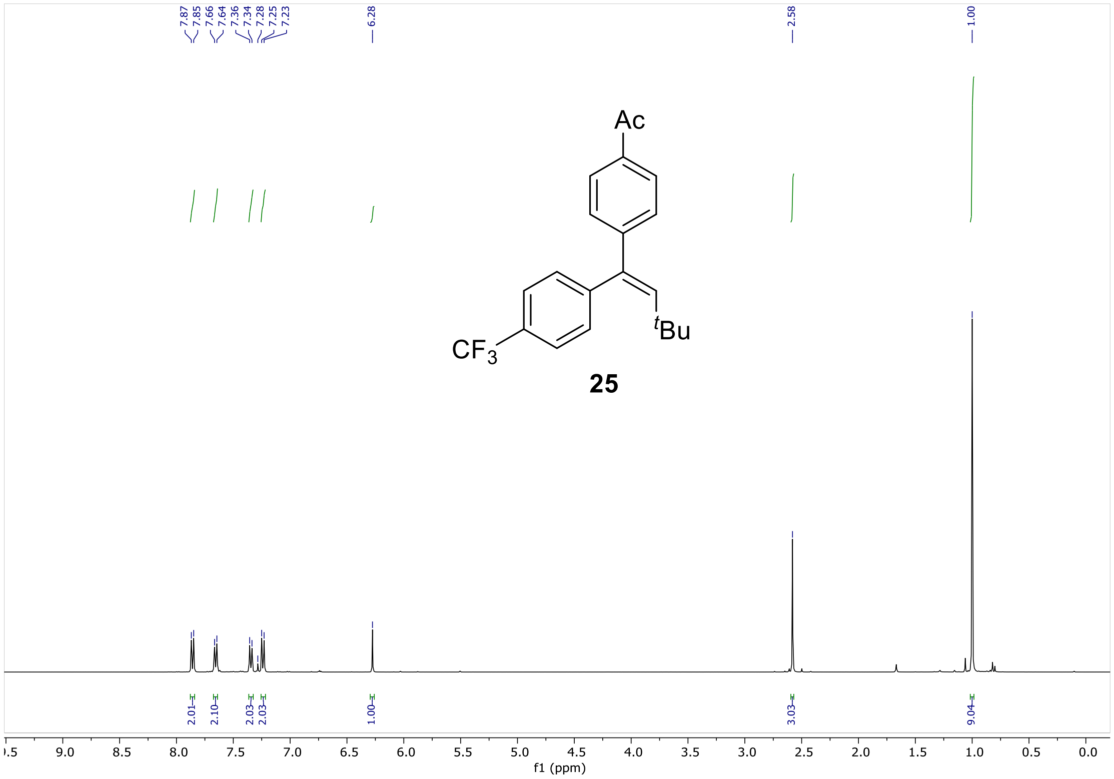

Supplementary Figure 77: ^{1}H NMR spectrum of compound **25**

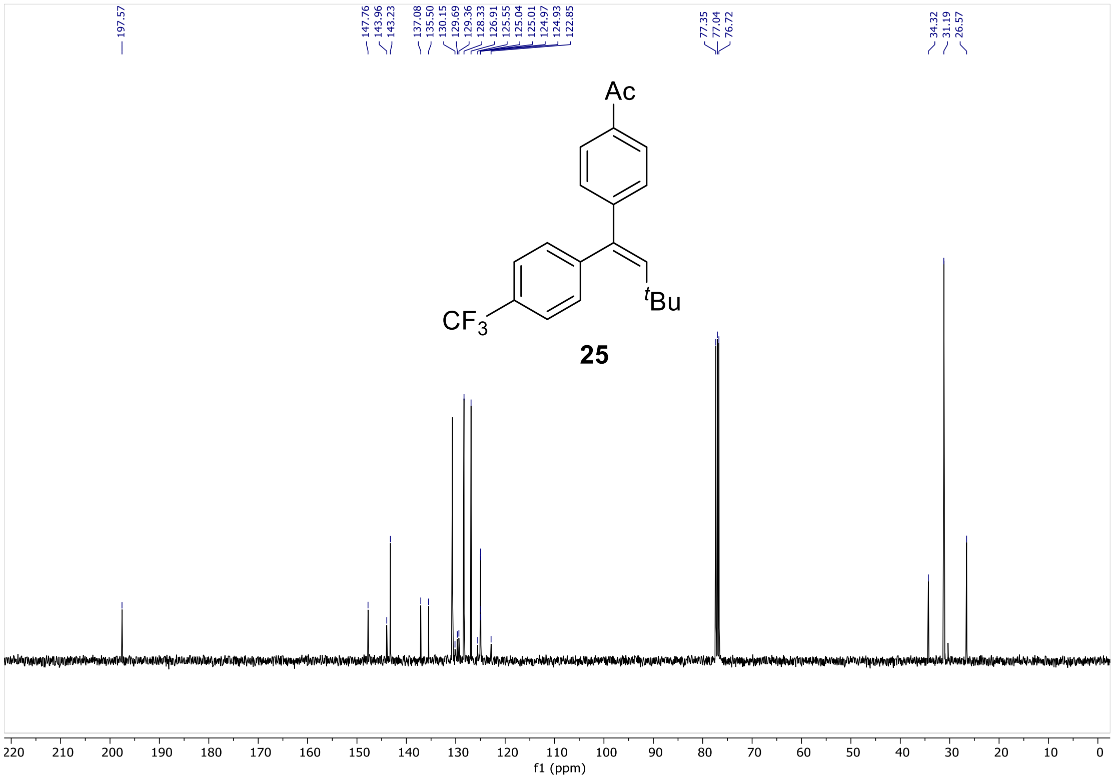

Supplementary Figure 78: ^{13}C NMR spectrum of compound **25**

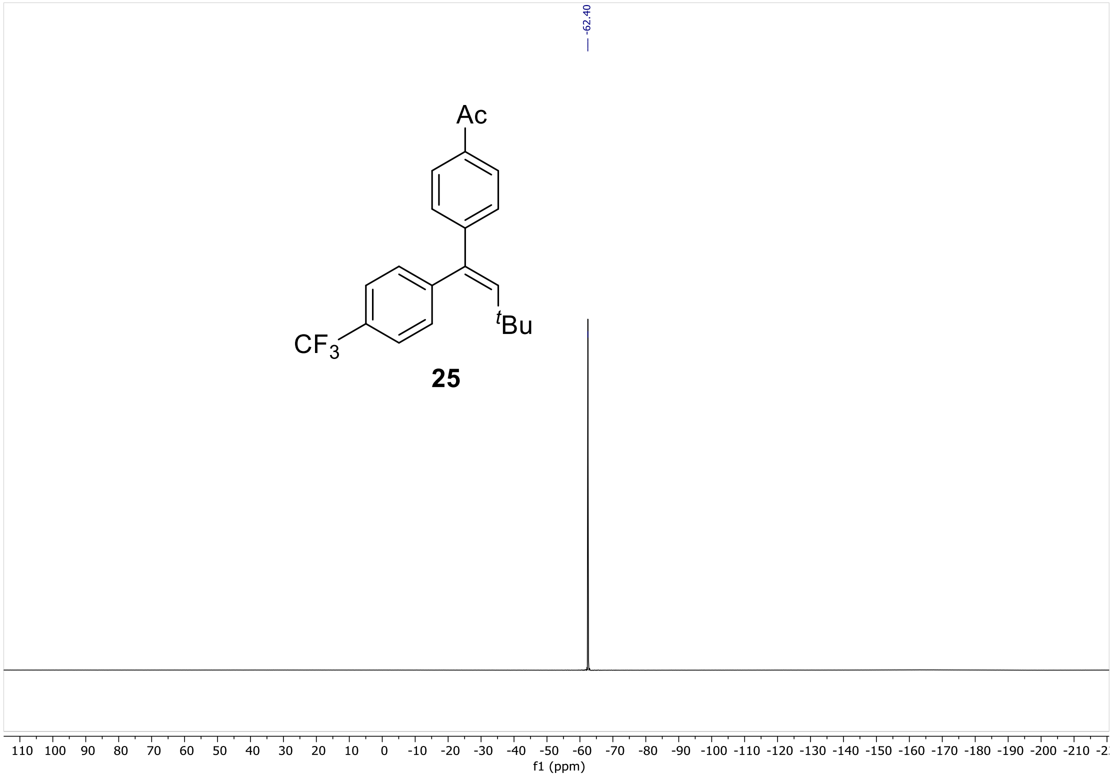

Supplementary Figure 79: ^{19}F NMR spectrum of compound **25**

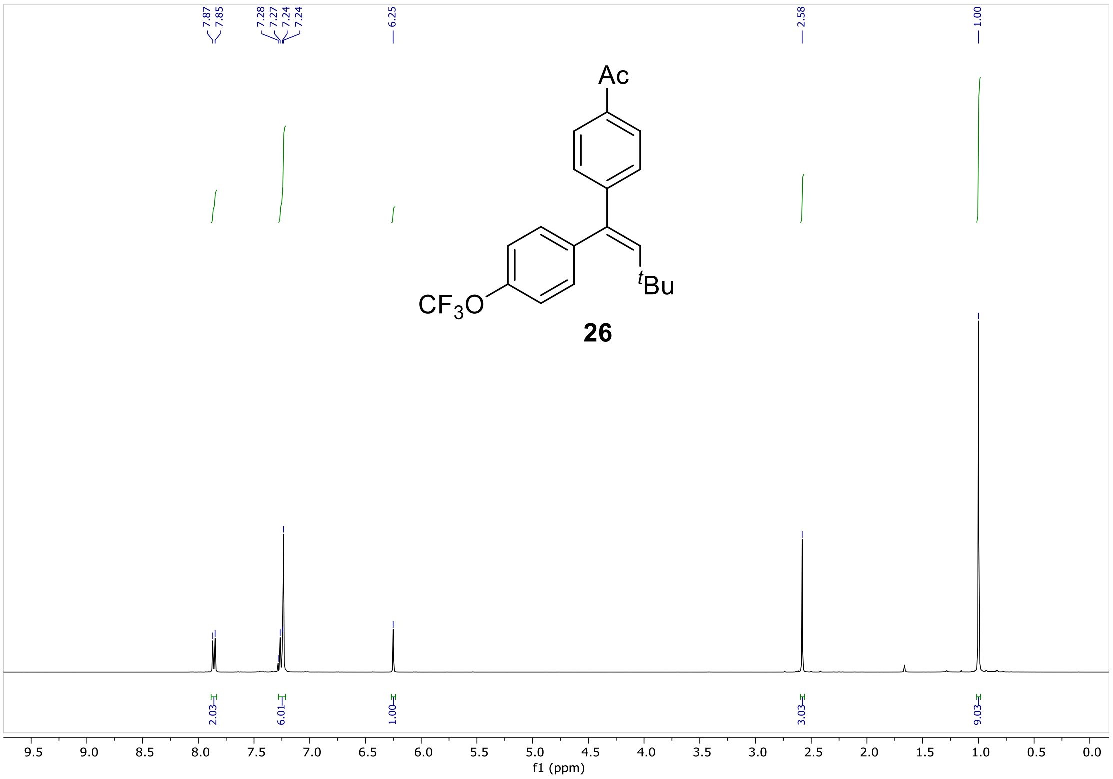

Supplementary Figure 80: ^{1}H NMR spectrum of compound **26**

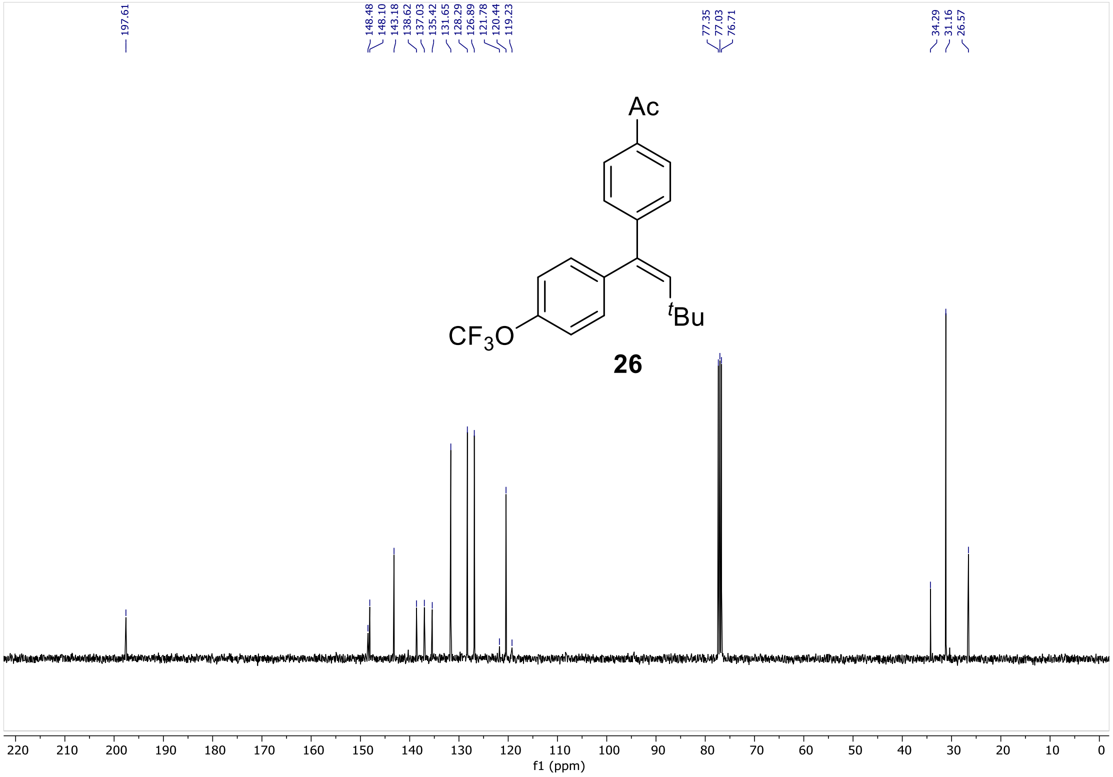

Supplementary Figure 81: ^{13}C NMR spectrum of compound **26**

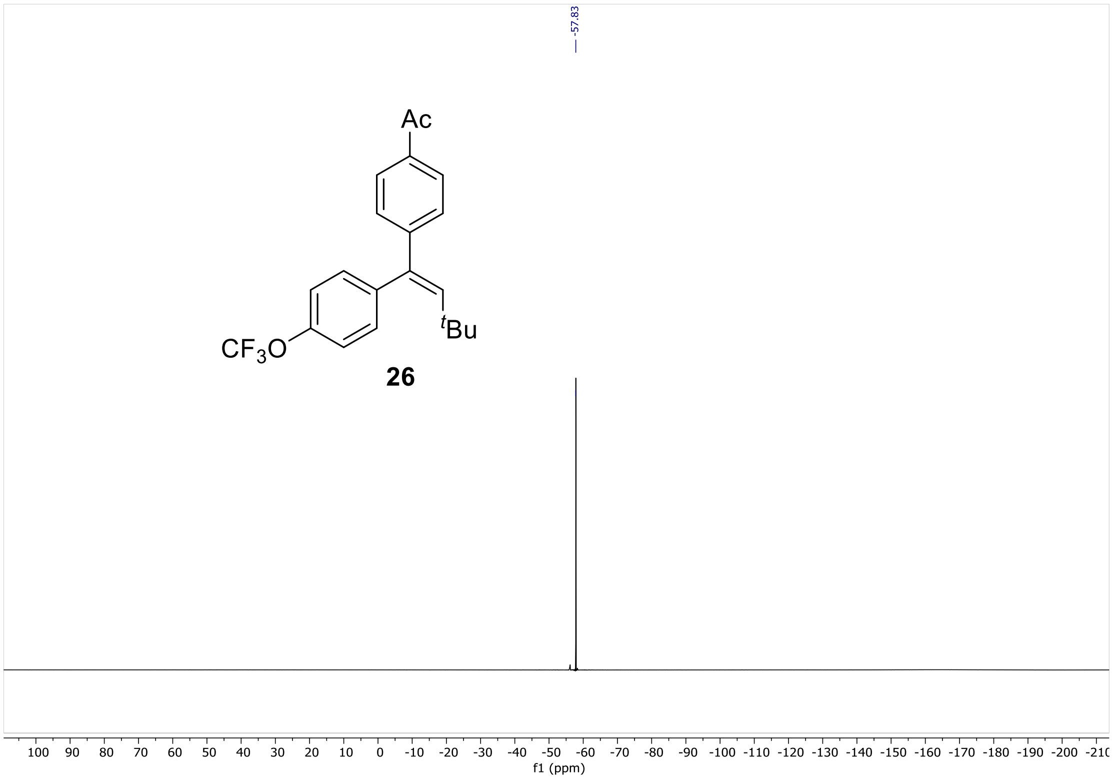

Supplementary Figure 82: ^{19}F NMR spectrum of compound **26**

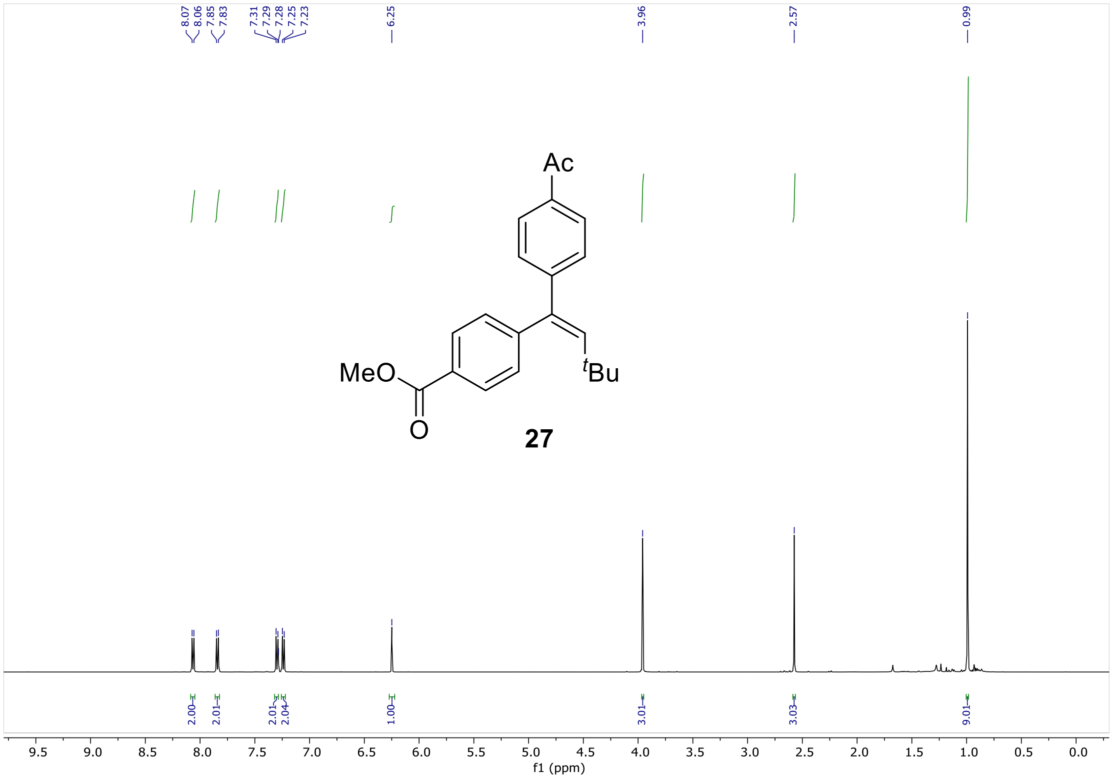

Supplementary Figure 83: ^{1}H NMR spectrum of compound **27**

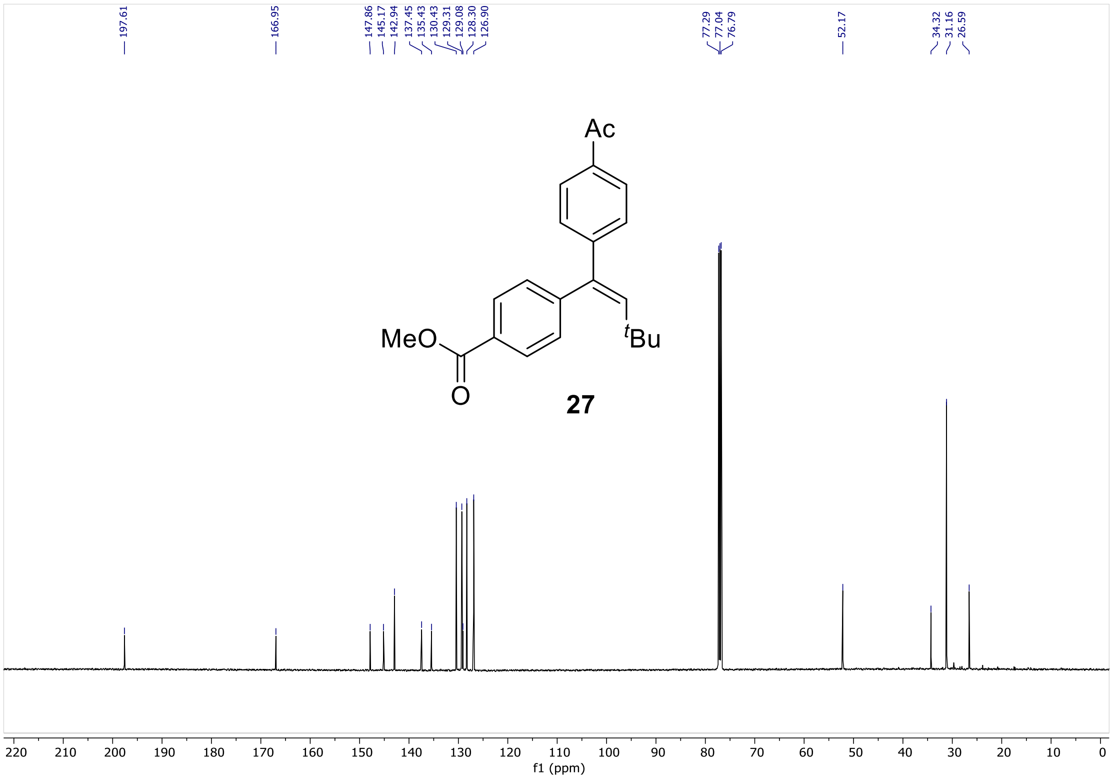

Supplementary Figure 84: ^{13}C NMR spectrum of compound **27**

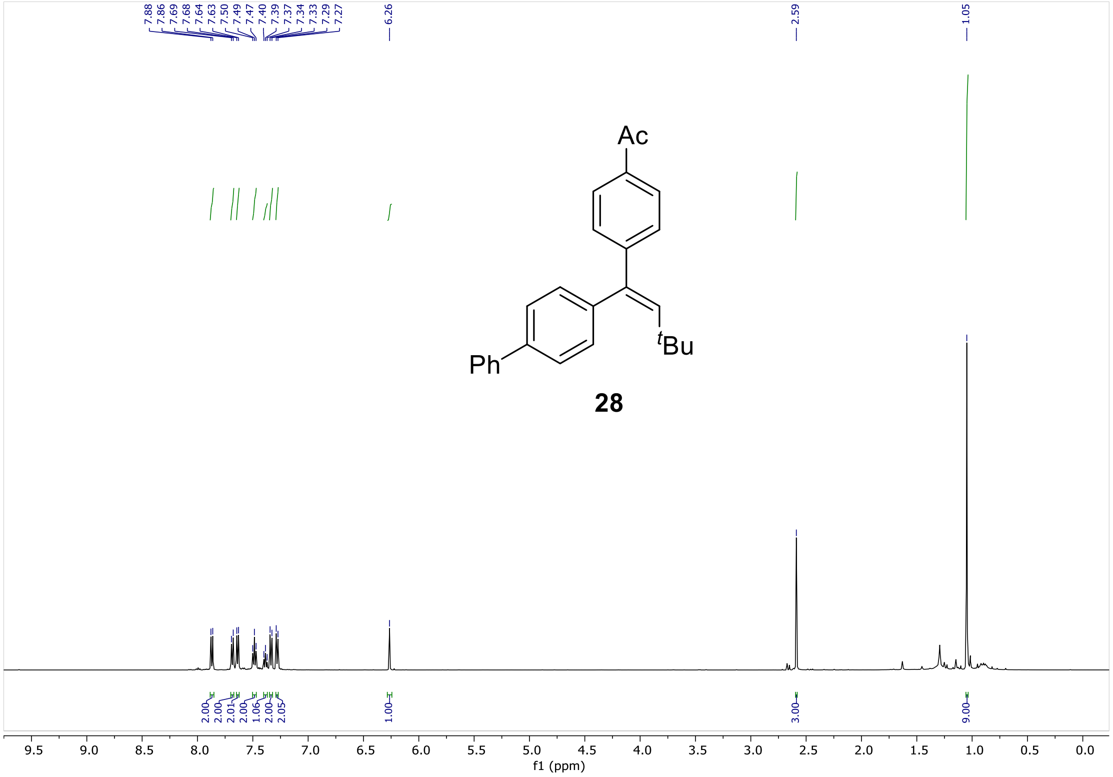

Supplementary Figure 85: ^{1}H NMR spectrum of compound **28**

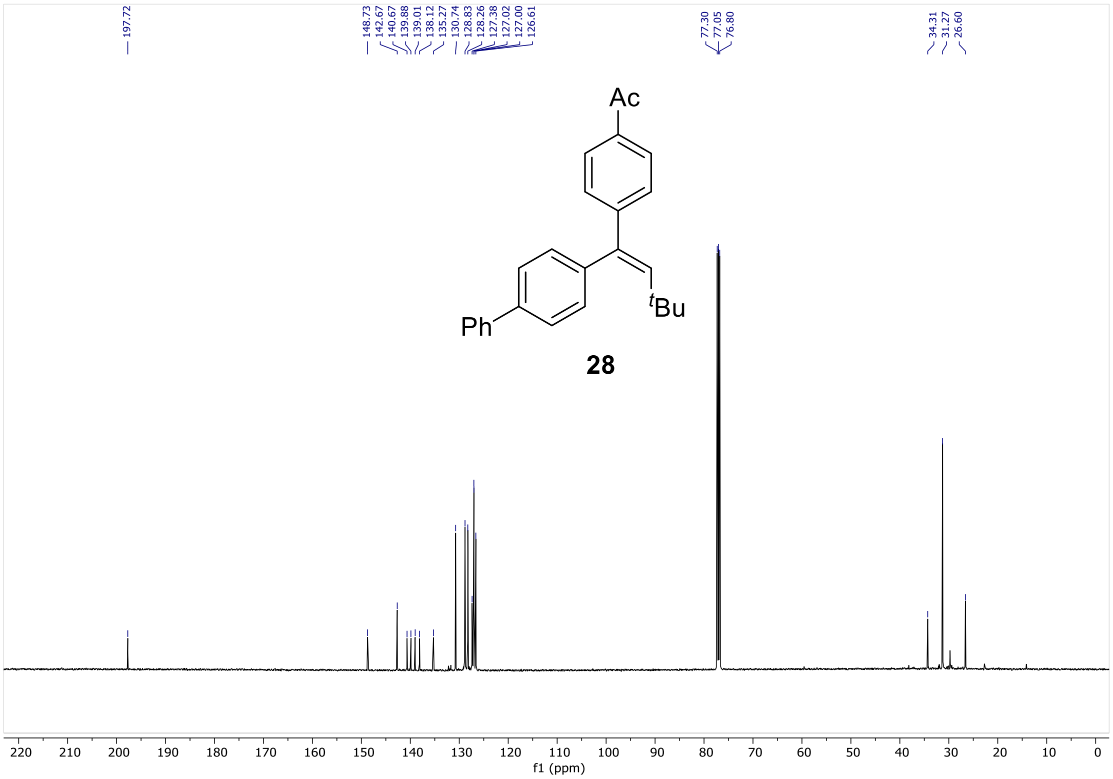

Supplementary Figure 86: ^{13}C NMR spectrum of compound **28**

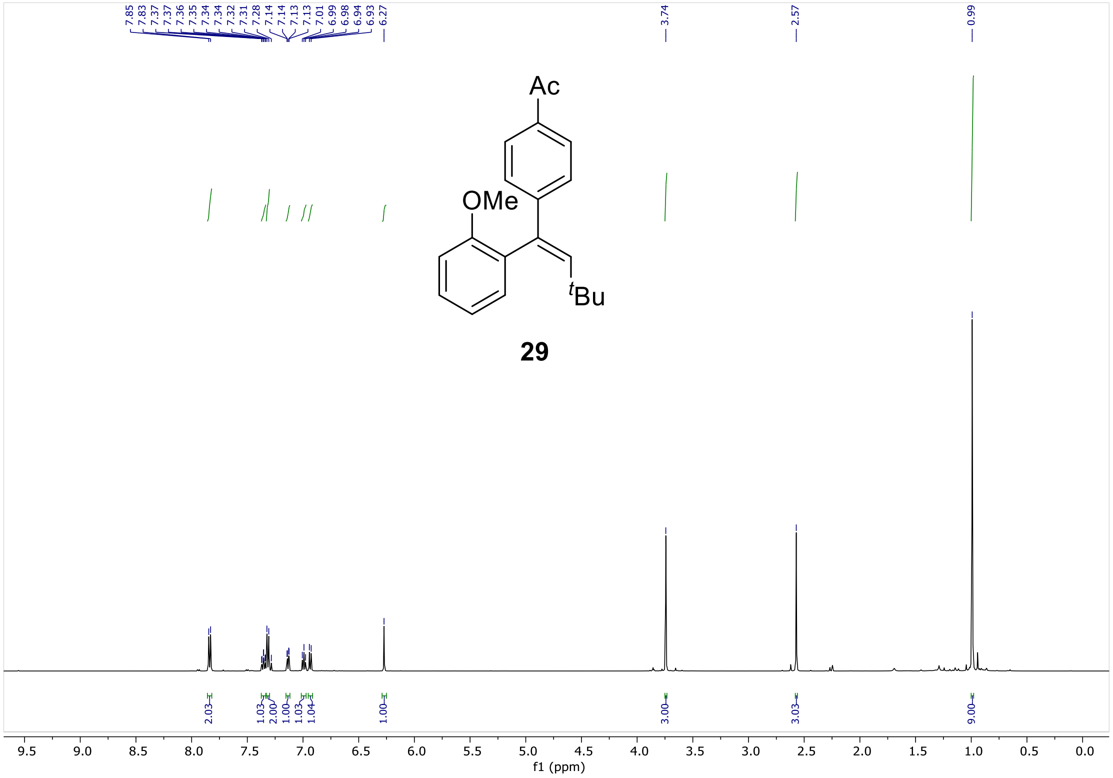

Supplementary Figure 87: ^{1}H NMR spectrum of compound **29**

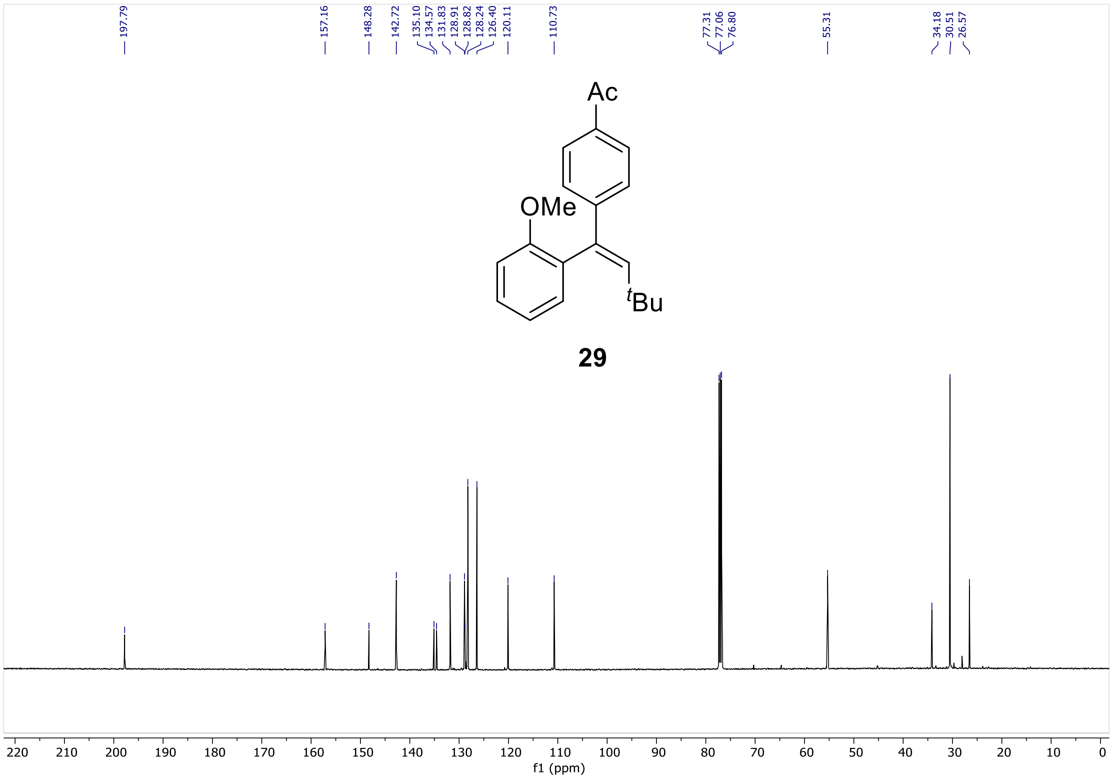

Supplementary Figure 88: ^{13}C NMR spectrum of compound **29**

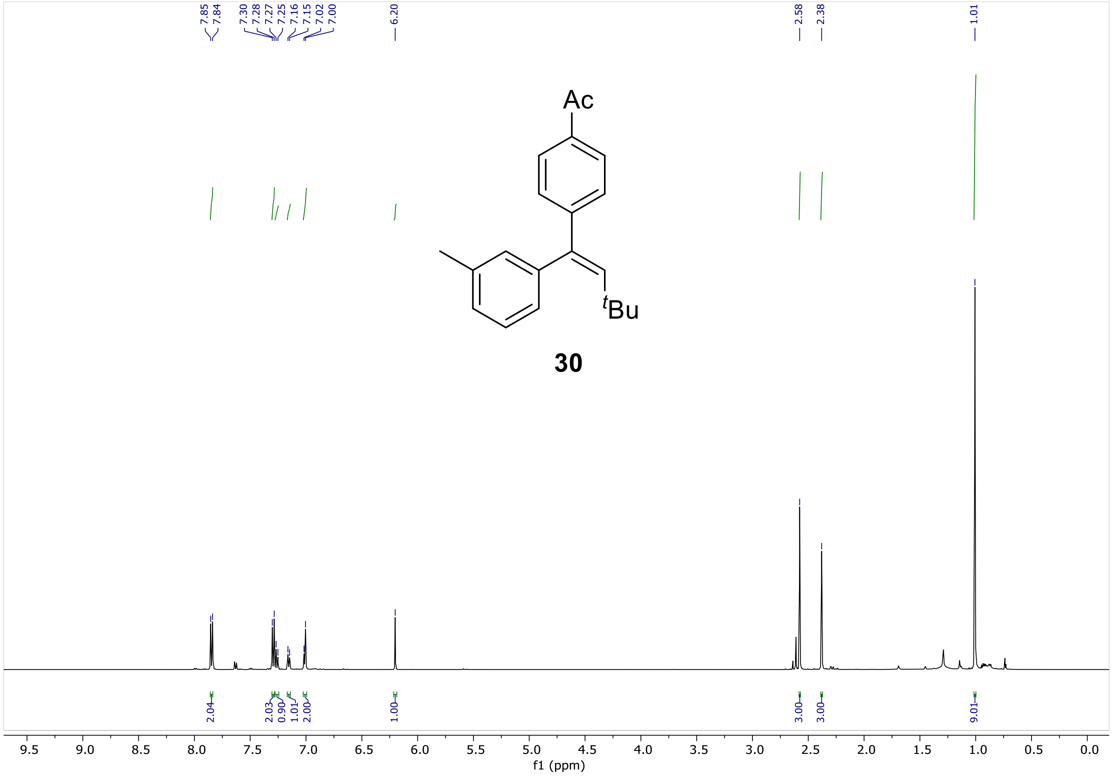

Supplementary Figure 89: ^{1}H NMR spectrum of compound **30**

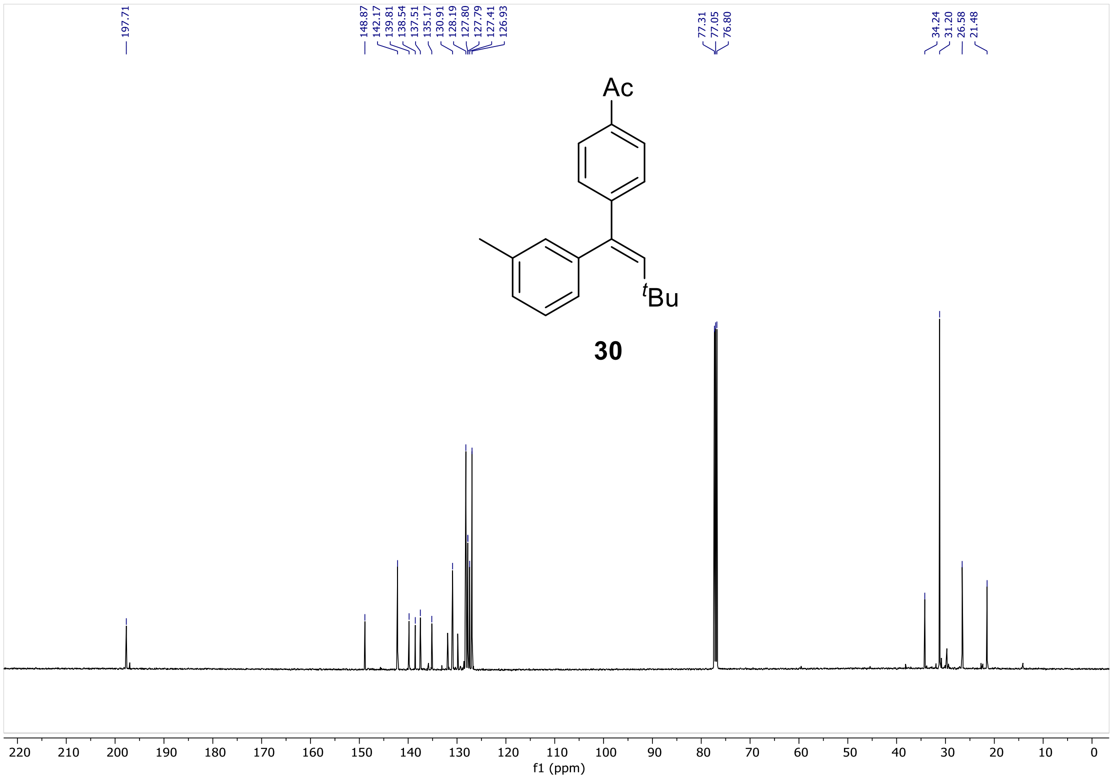

Supplementary Figure 90: ^{13}C NMR spectrum of compound **30**

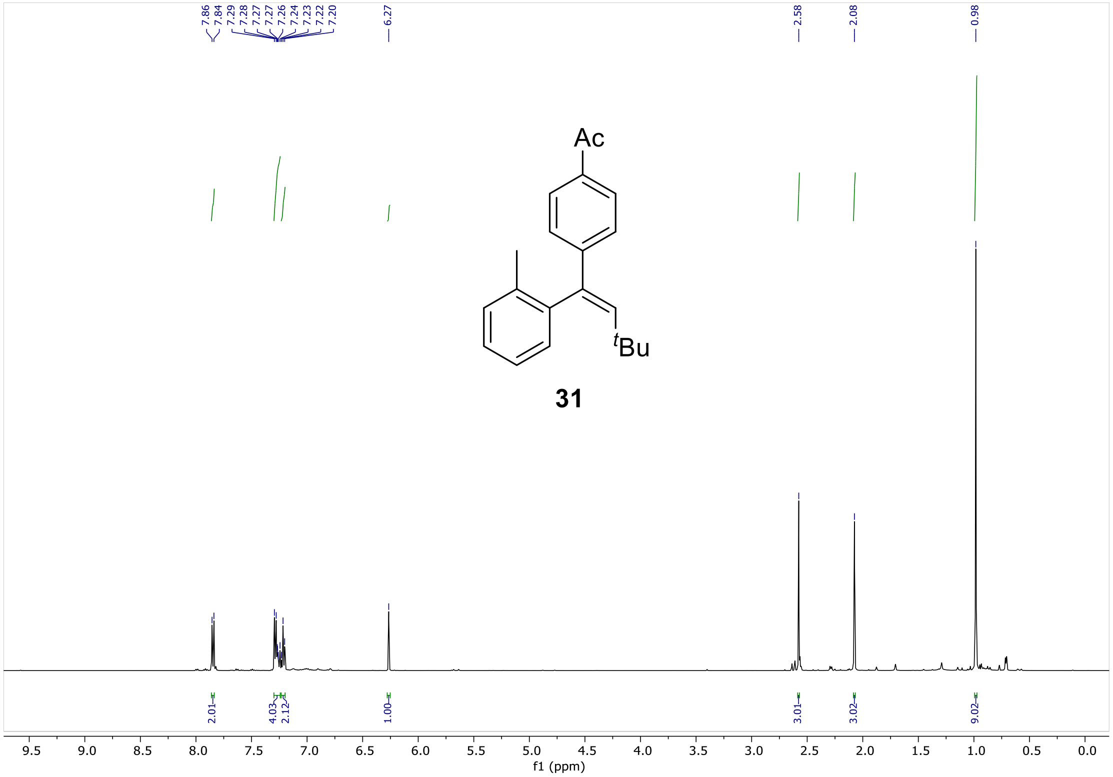

Supplementary Figure 91: ^{1}H NMR spectrum of compound **31**

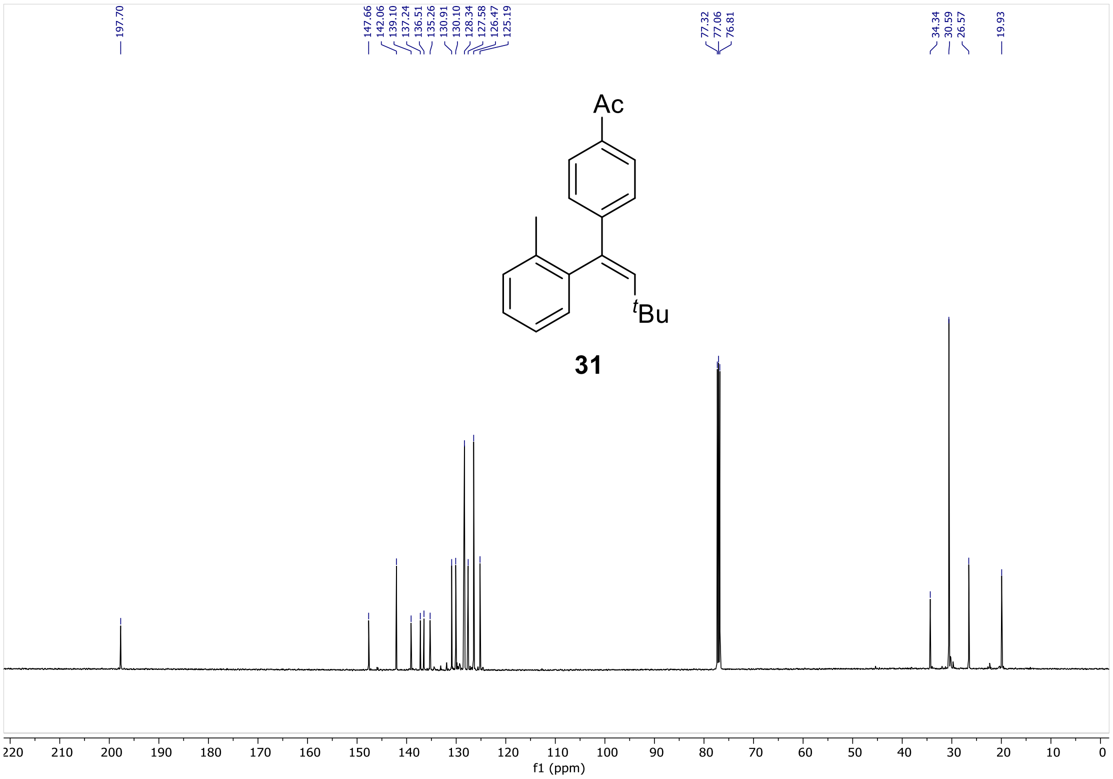

Supplementary Figure 92: ^{13}C NMR spectrum of compound **31**

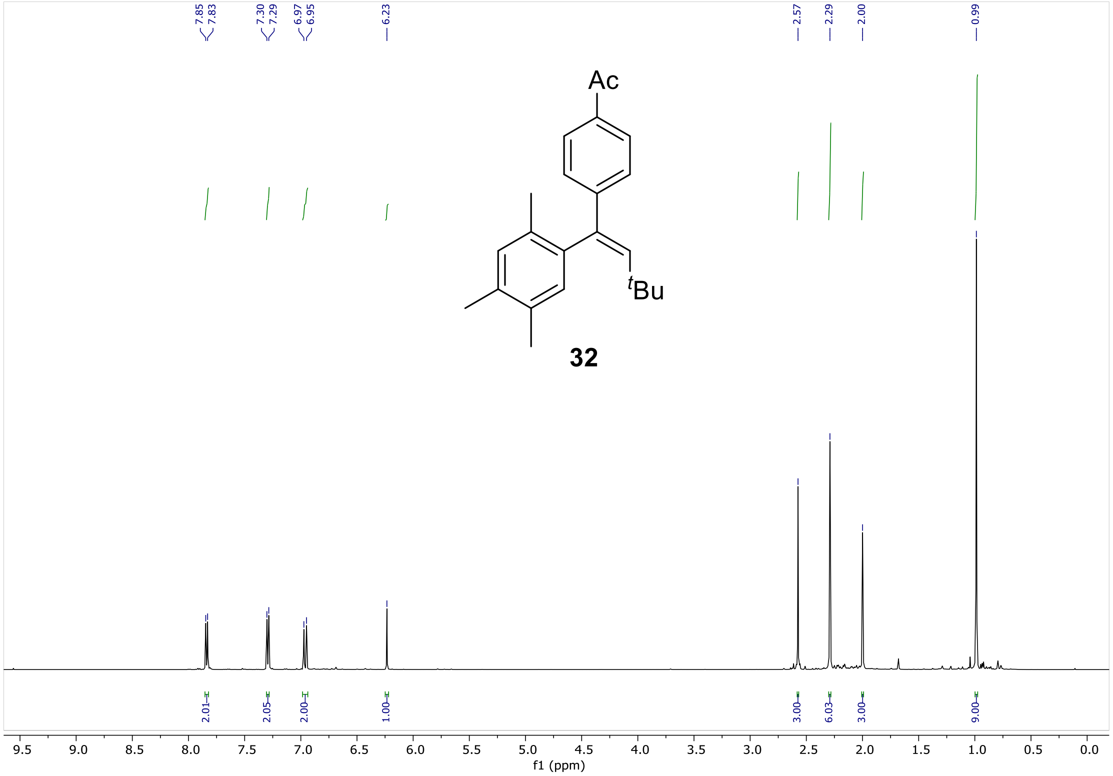

Supplementary Figure 93: ^{1}H NMR spectrum of compound **32**

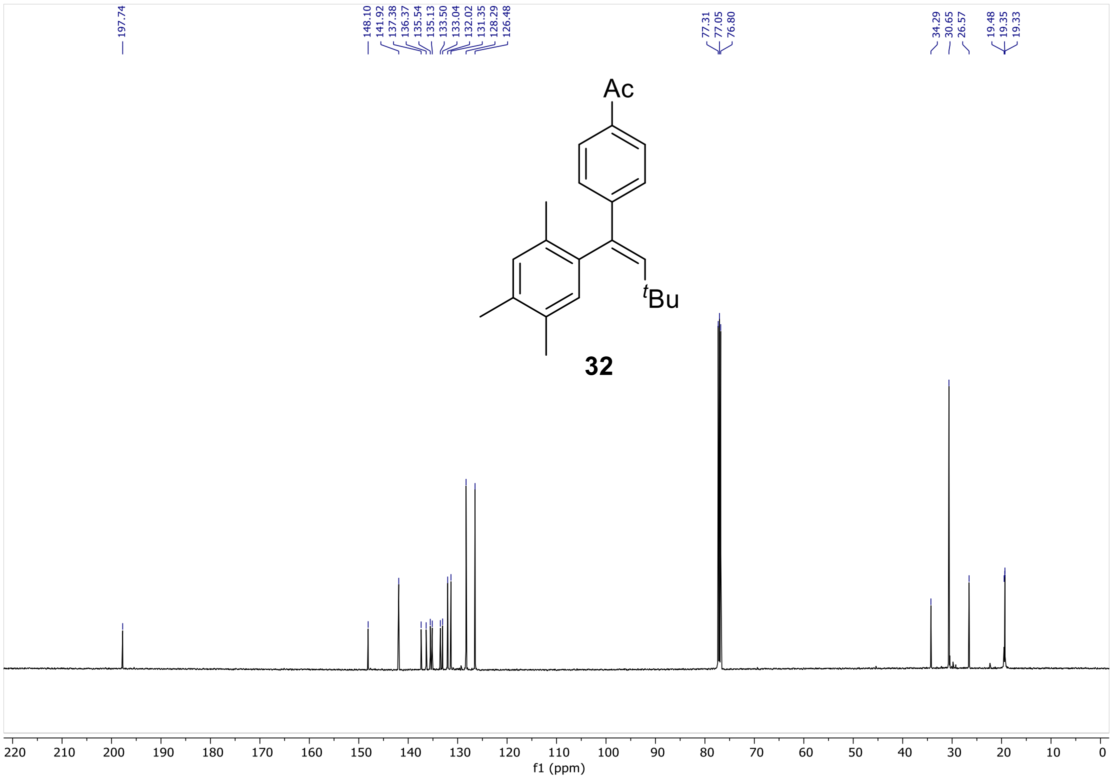

Supplementary Figure 94: ^{13}C NMR spectrum of compound **32**

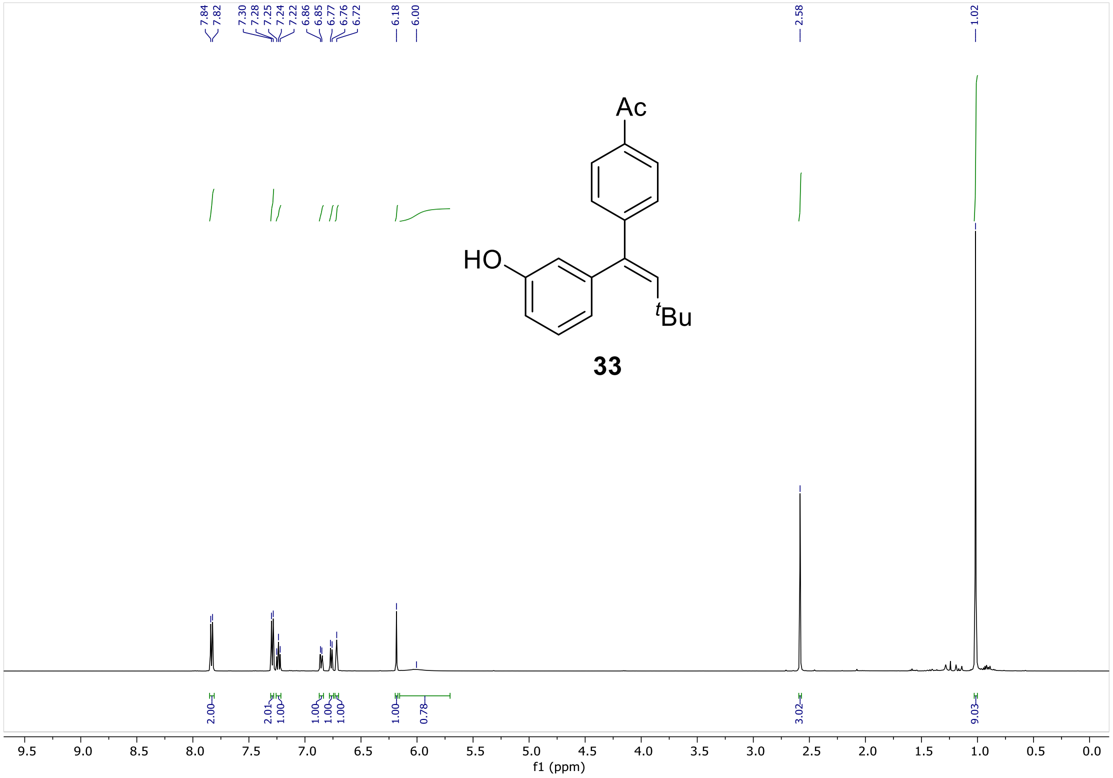

Supplementary Figure 95: ^{1}H NMR spectrum of compound **33**

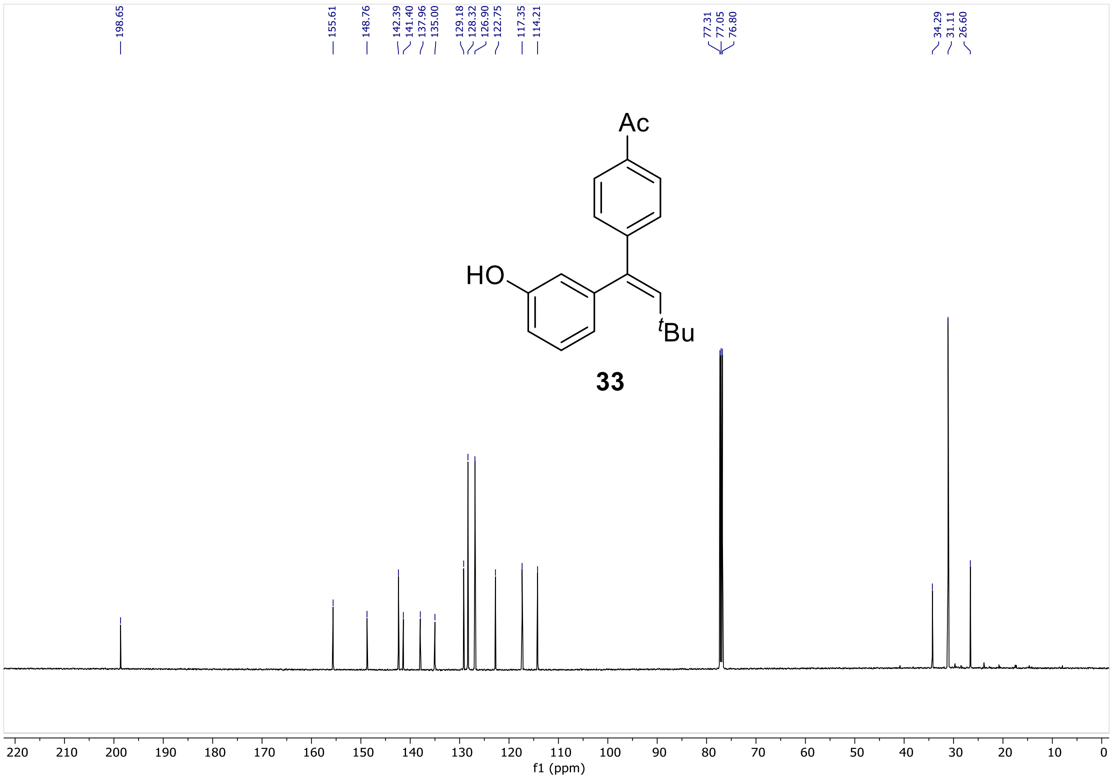

Supplementary Figure 96: ^{13}C NMR spectrum of compound **33**

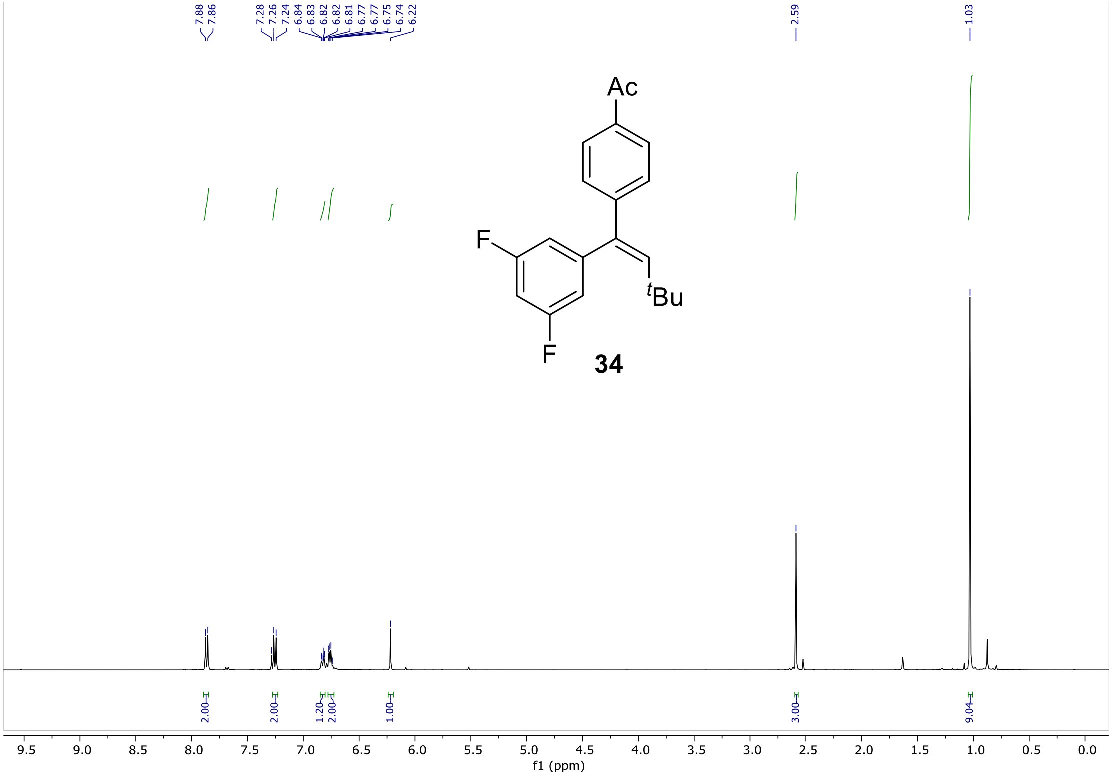

Supplementary Figure 97: ^{1}H NMR spectrum of compound **34**

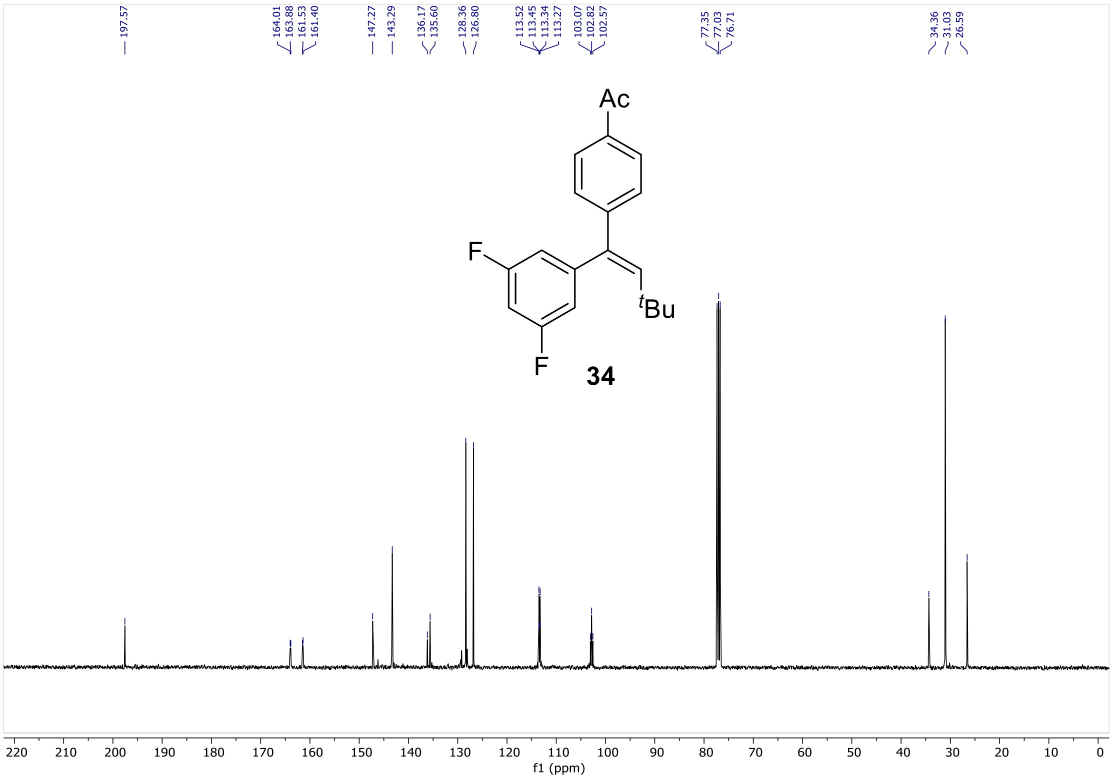

Supplementary Figure 98: ^{13}C NMR spectrum of compound **34**

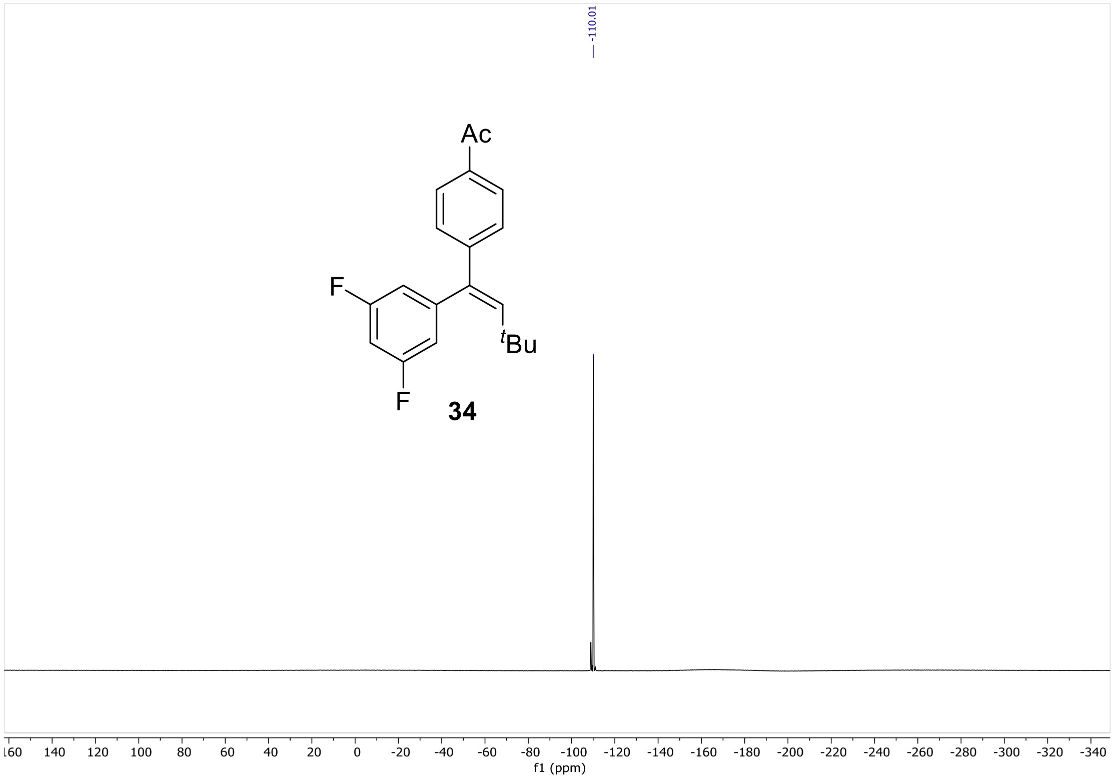

Supplementary Figure 99: ^{19}F NMR spectrum of compound **34**

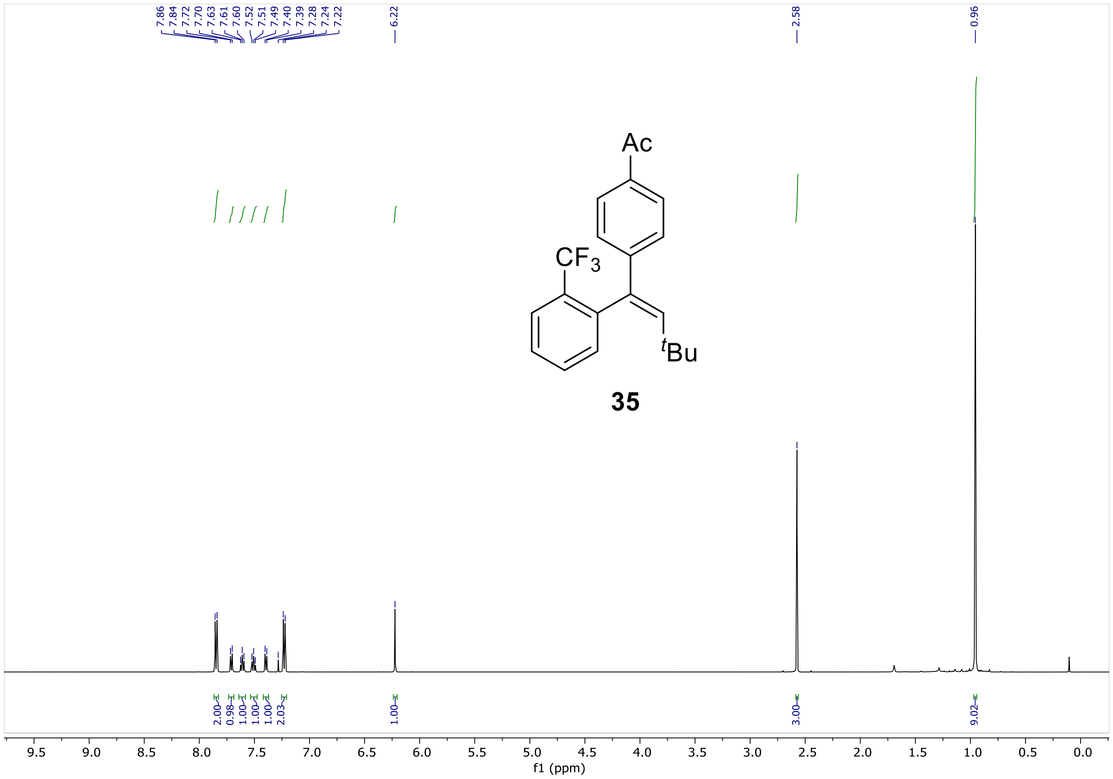

Supplementary Figure 100: ^{1}H NMR spectrum of compound **35**

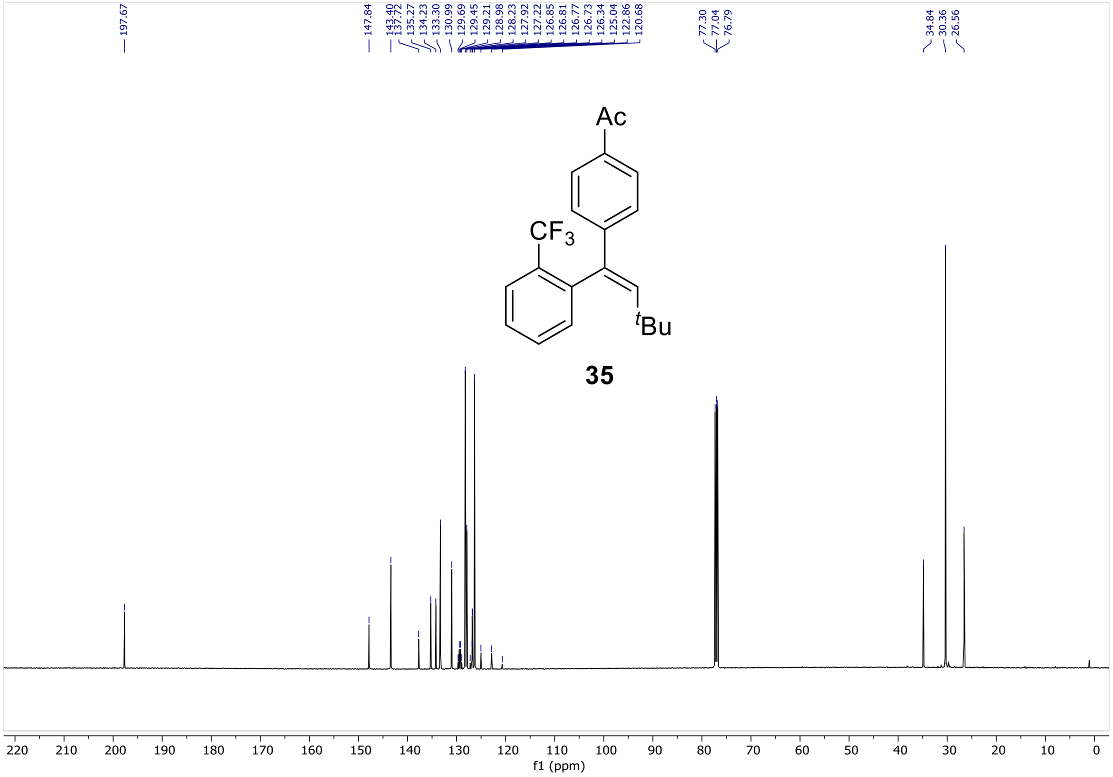

Supplementary Figure 101: ^{13}C NMR spectrum of compound **35**

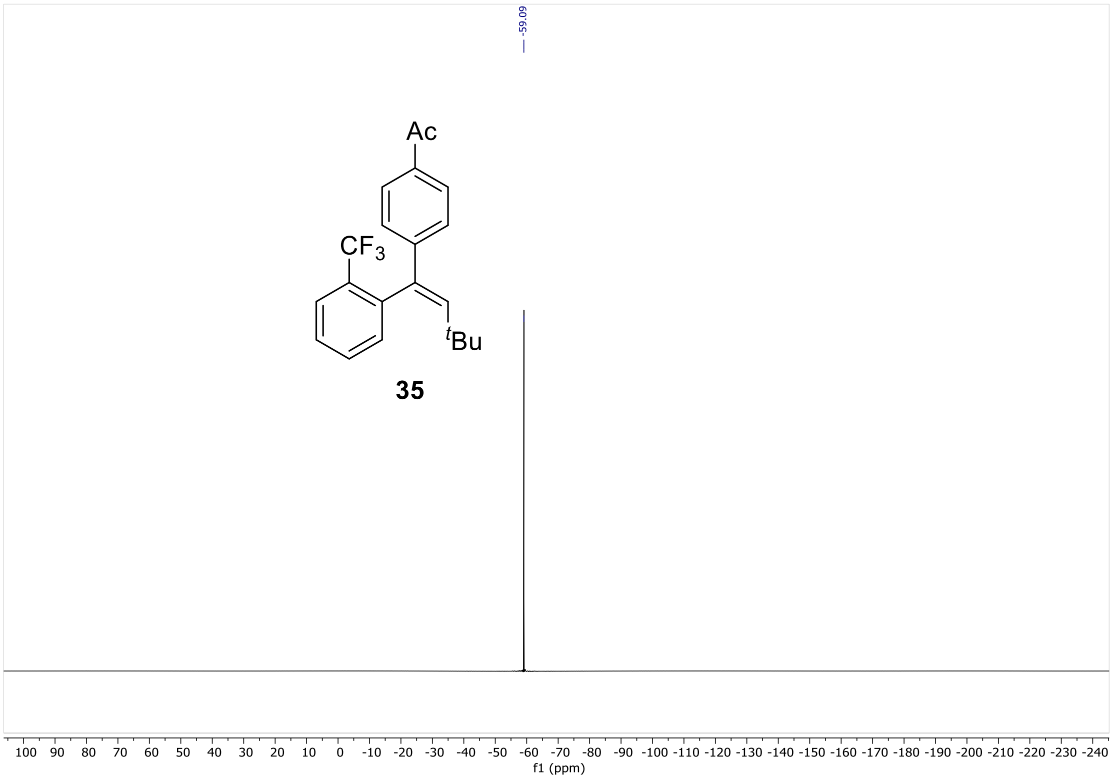

Supplementary Figure 102: ^{19}F NMR spectrum of compound **35**

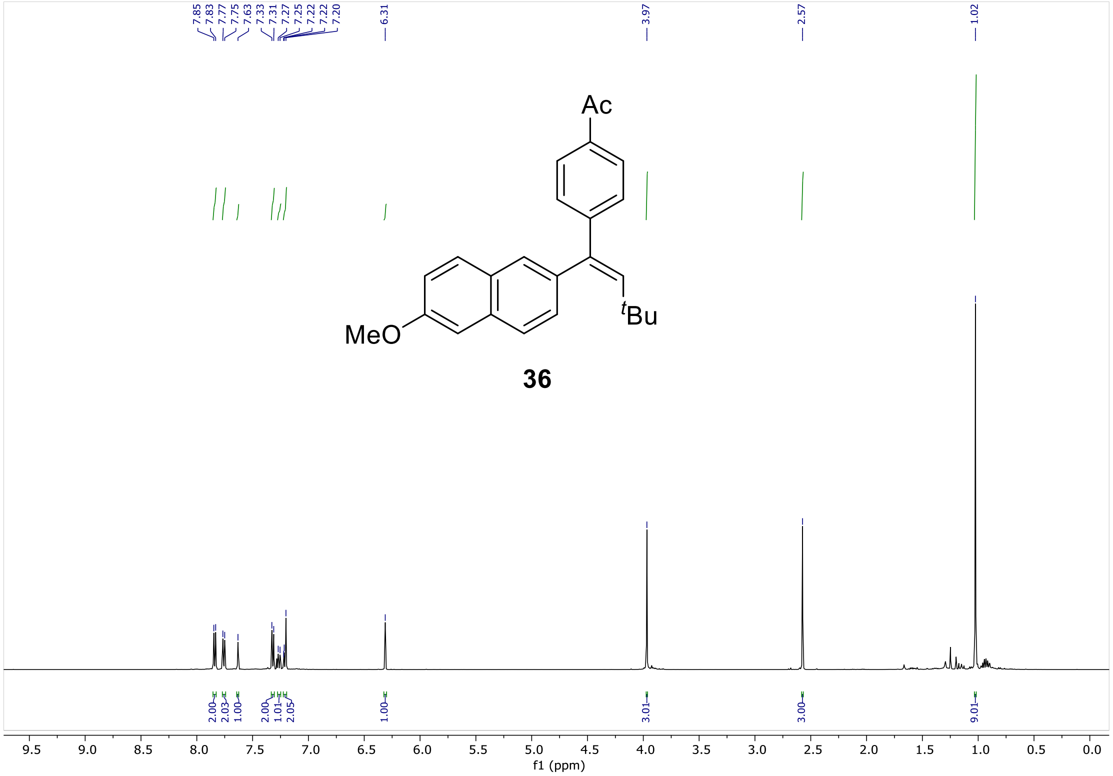

Supplementary Figure 103: ^{1}H NMR spectrum of compound **36**

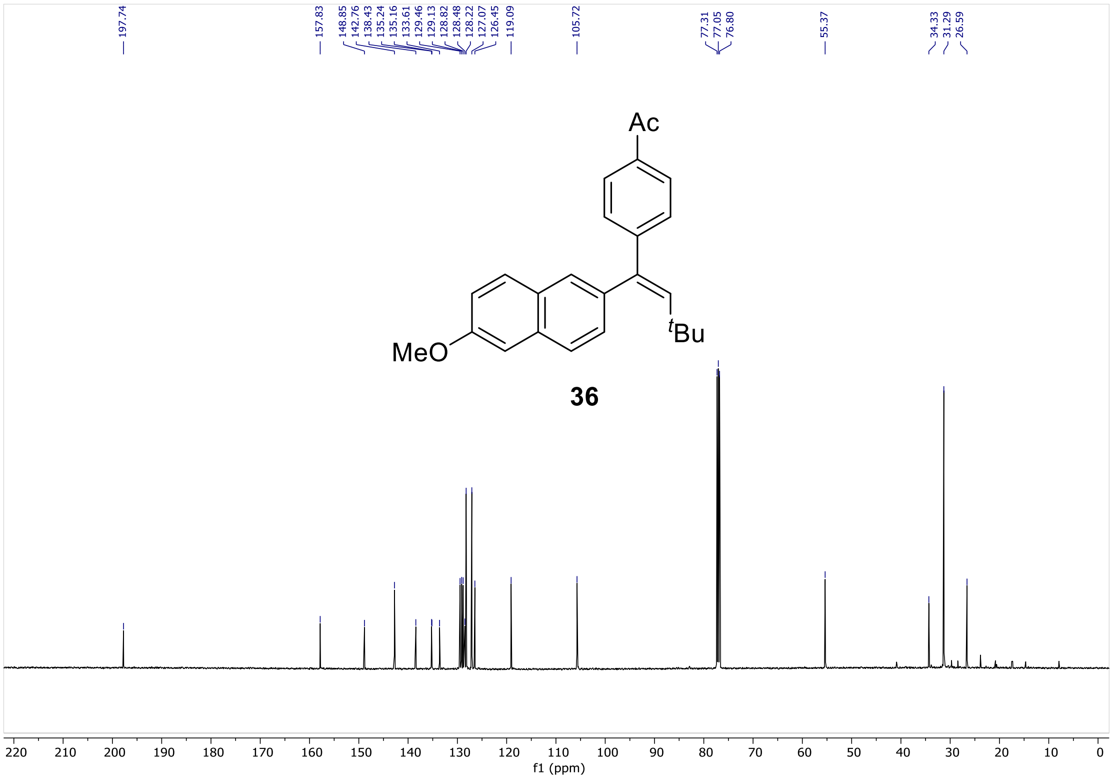

Supplementary Figure 104: ^{13}C NMR spectrum of compound **36**

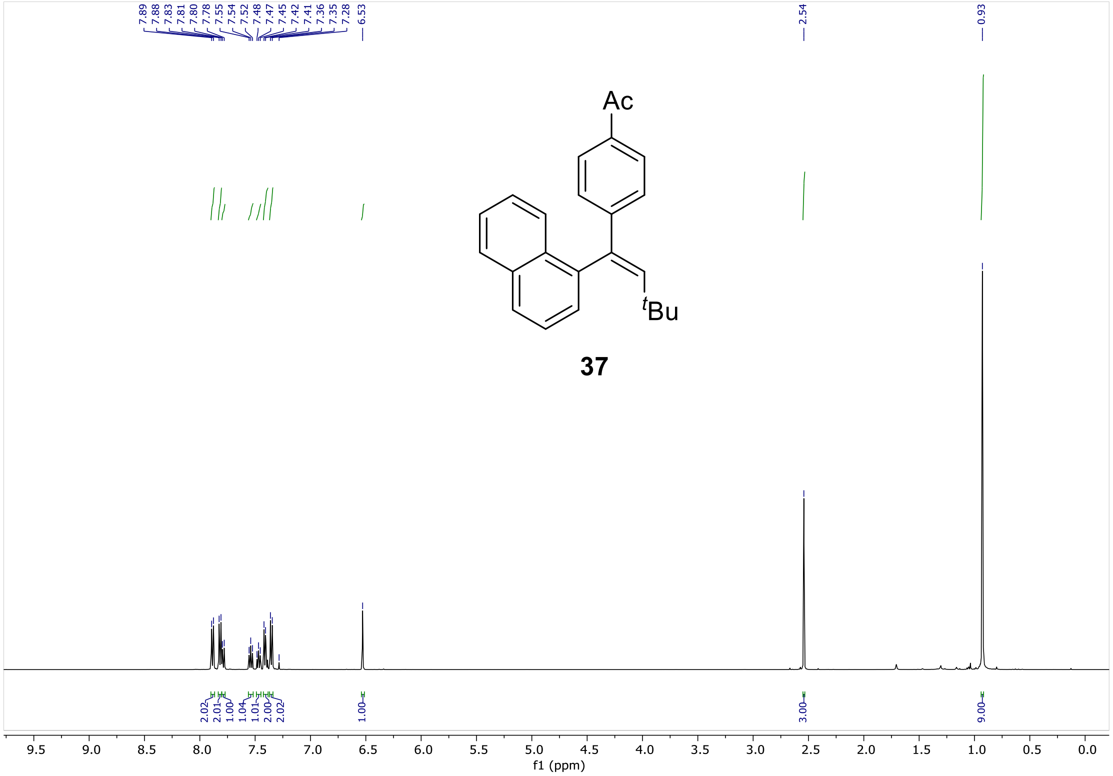

Supplementary Figure 105: ^{1}H NMR spectrum of compound **37**

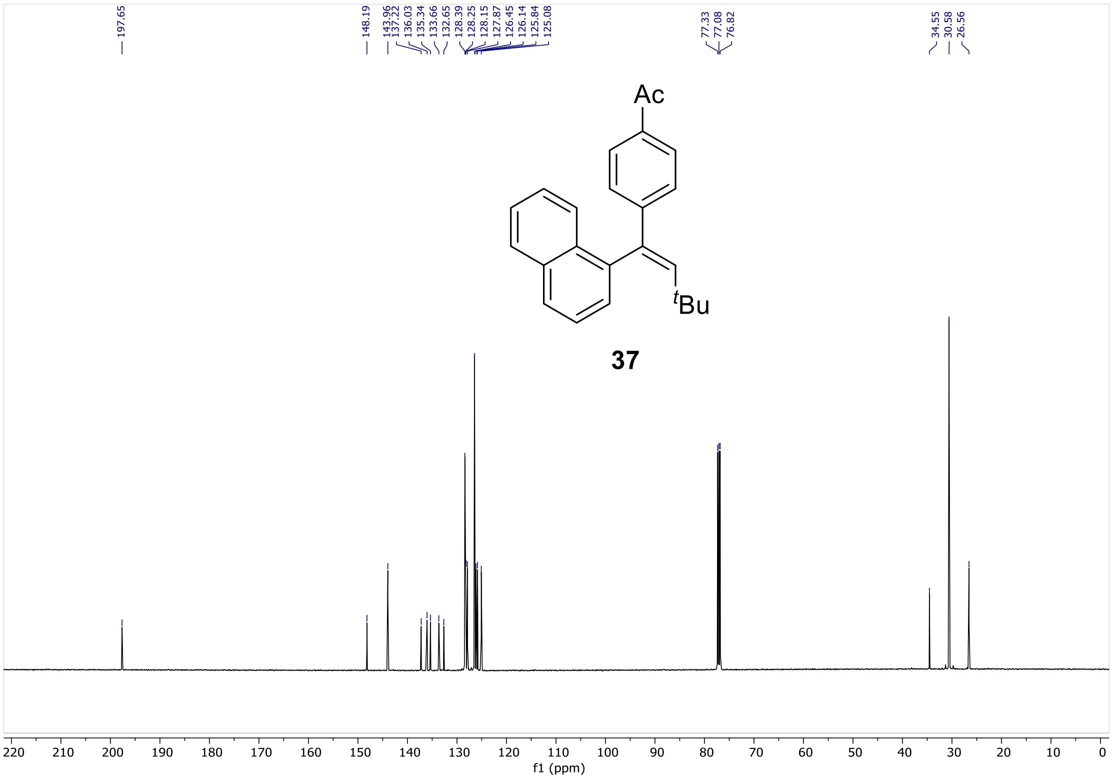

Supplementary Figure 106: ^{13}C NMR spectrum of compound **37**

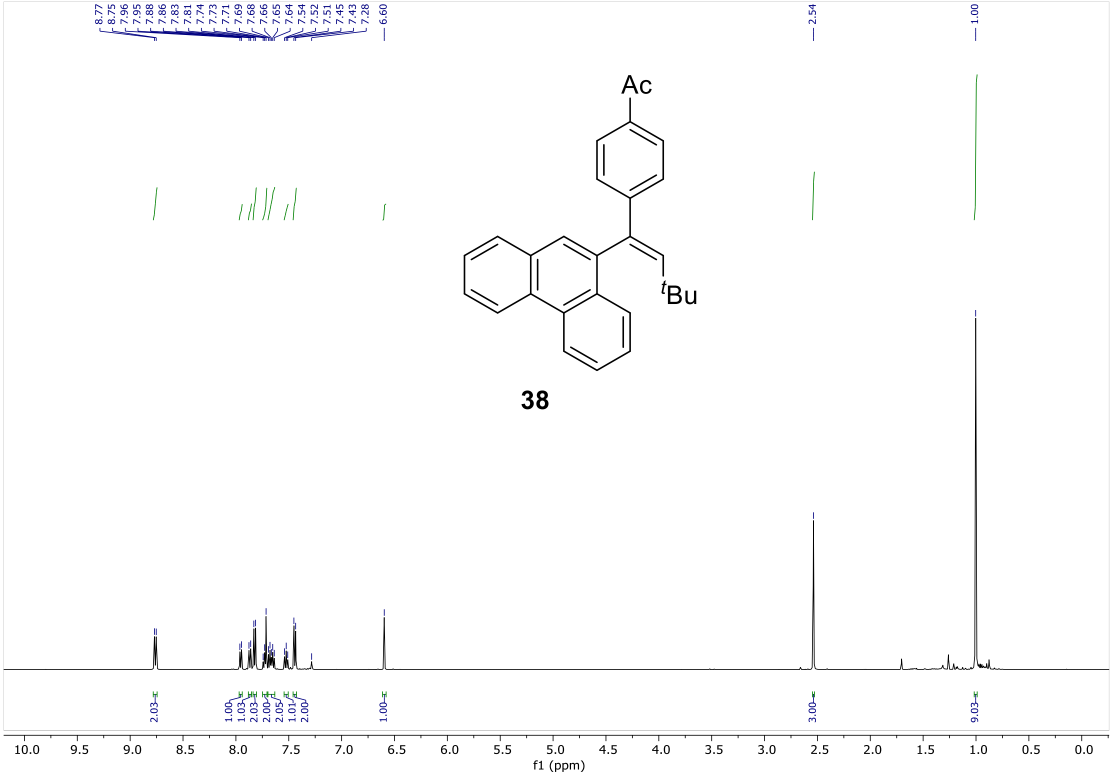

Supplementary Figure 107: ^{1}H NMR spectrum of compound **38**

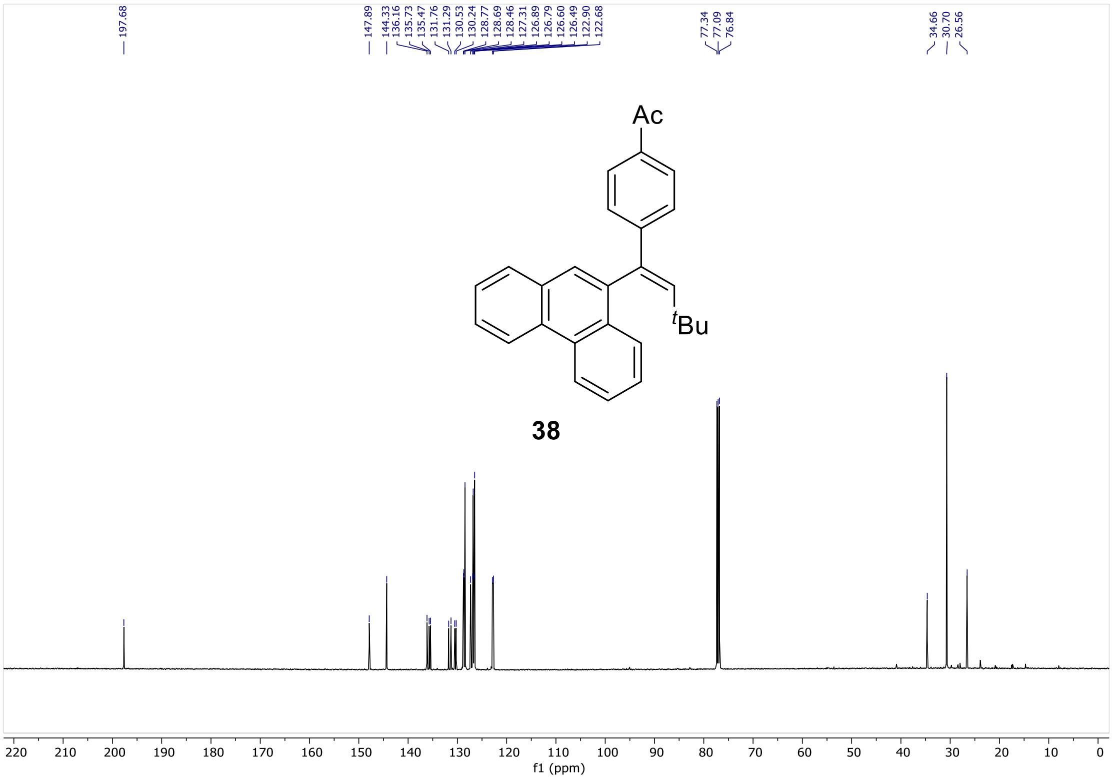

Supplementary Figure 108: ^{13}C NMR spectrum of compound **38**

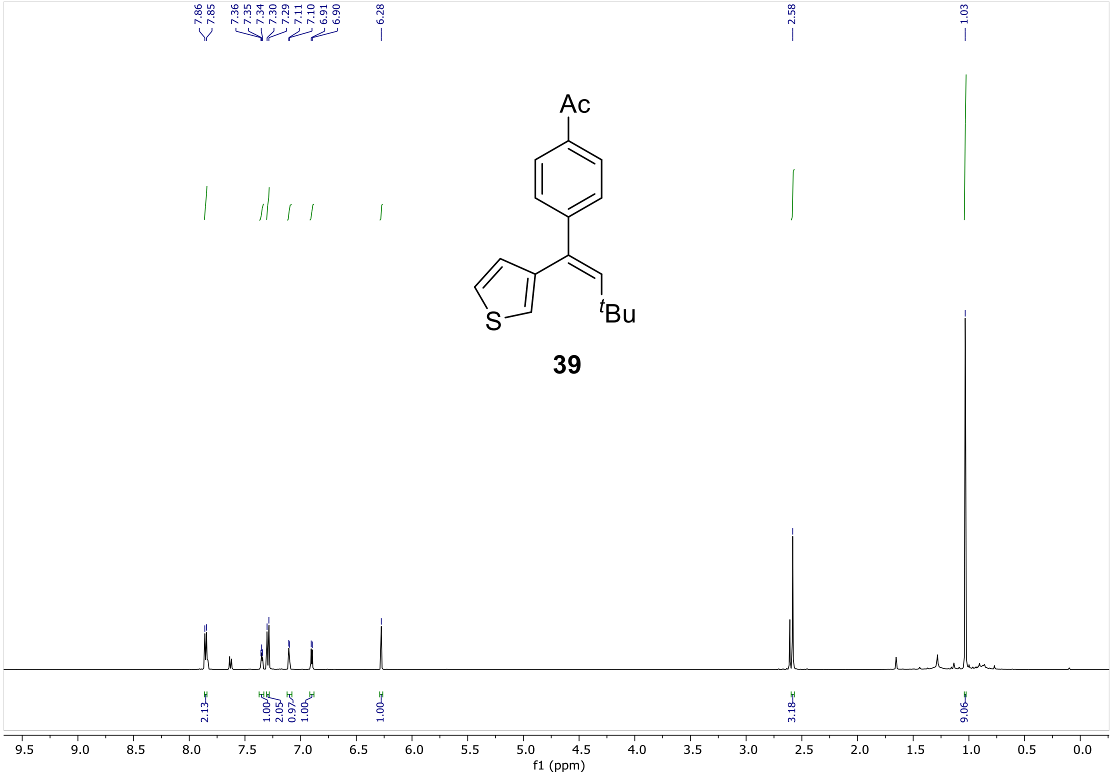

Supplementary Figure 109: ^{1}H NMR spectrum of compound **39**

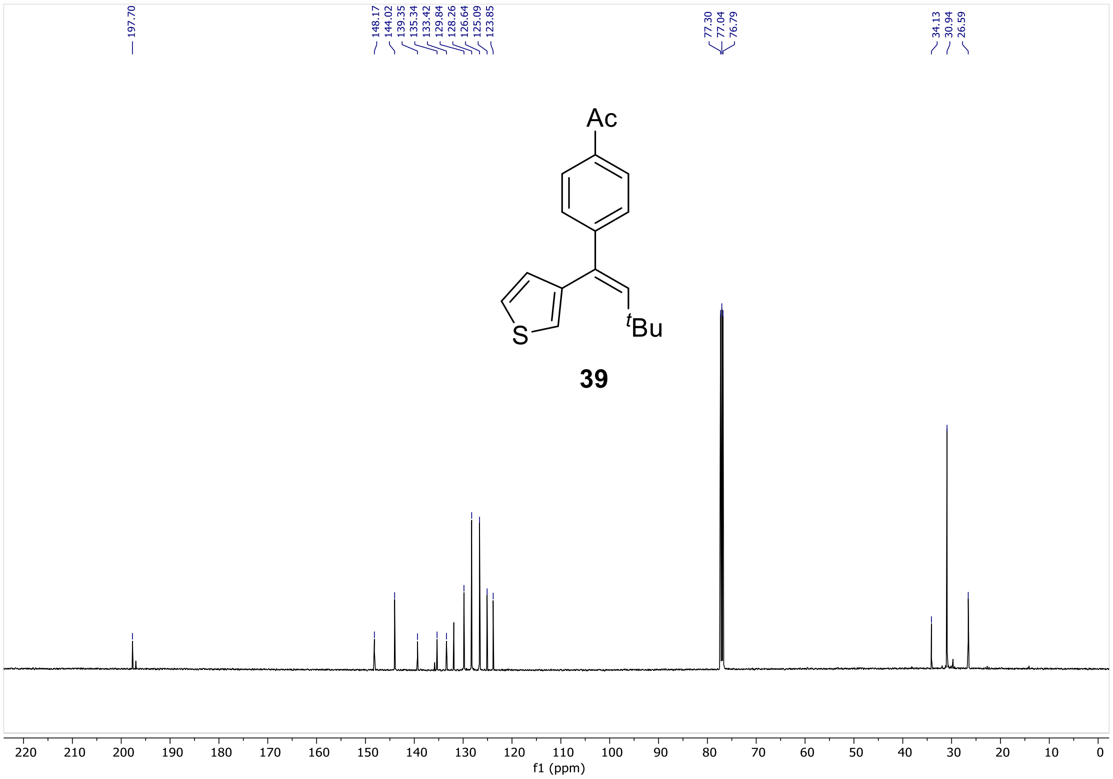

Supplementary Figure 110: ^{13}C NMR spectrum of compound **39**

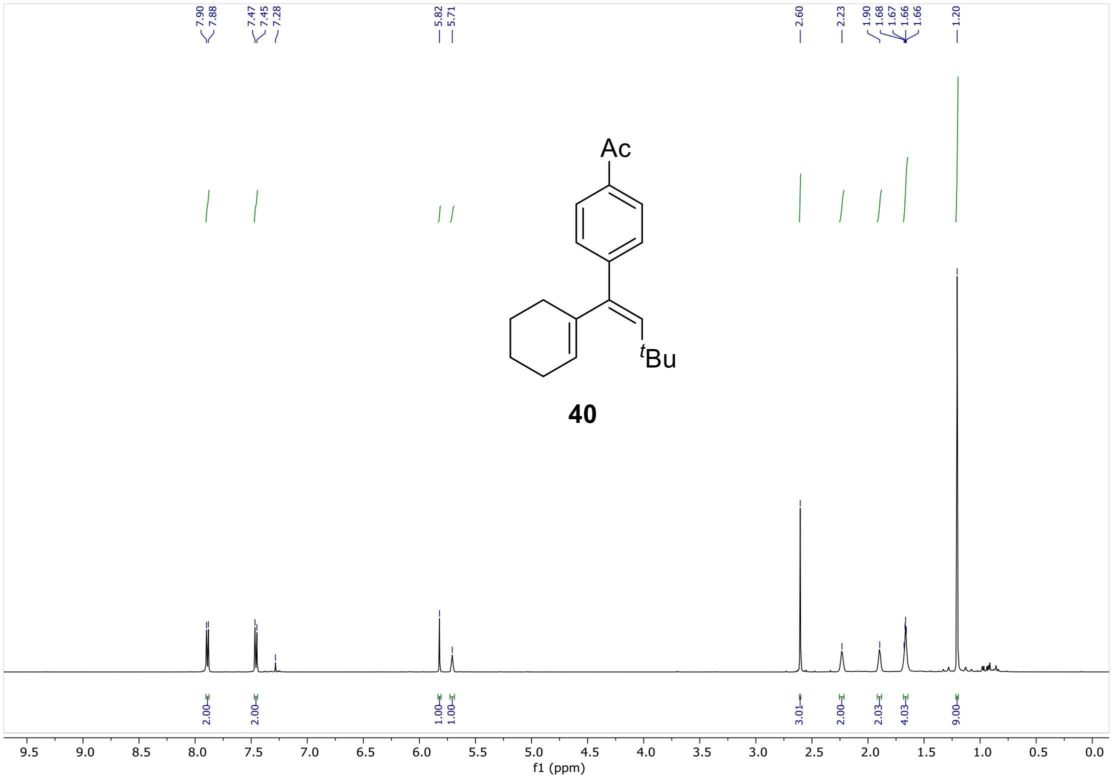

Supplementary Figure 111: ^{1}H NMR spectrum of compound **40**

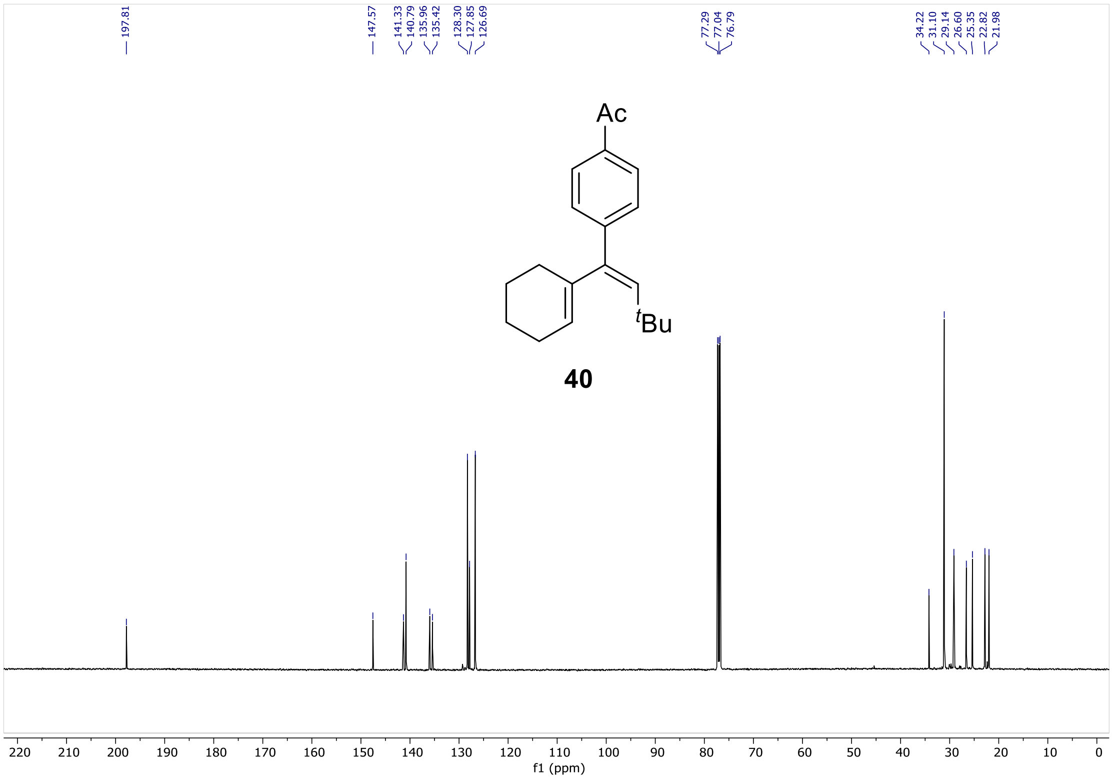

Supplementary Figure 112: ^{13}C NMR spectrum of compound **40**

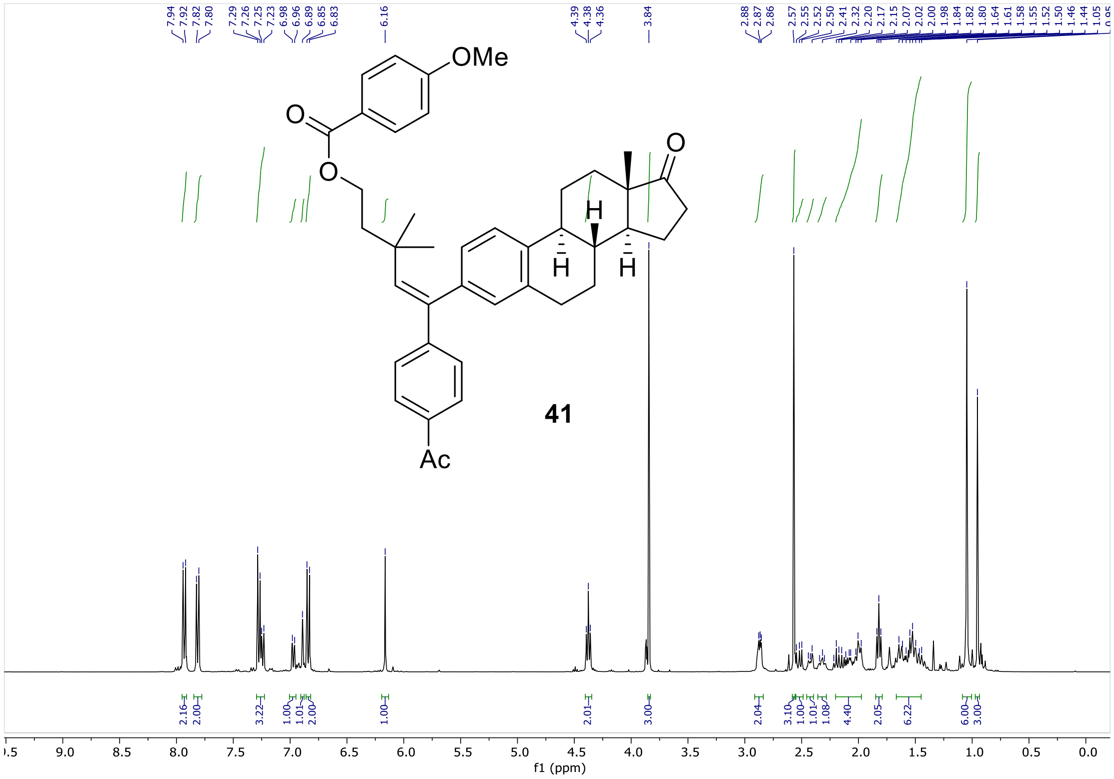

Supplementary Figure 113: ^{1}H NMR spectrum of compound **41**

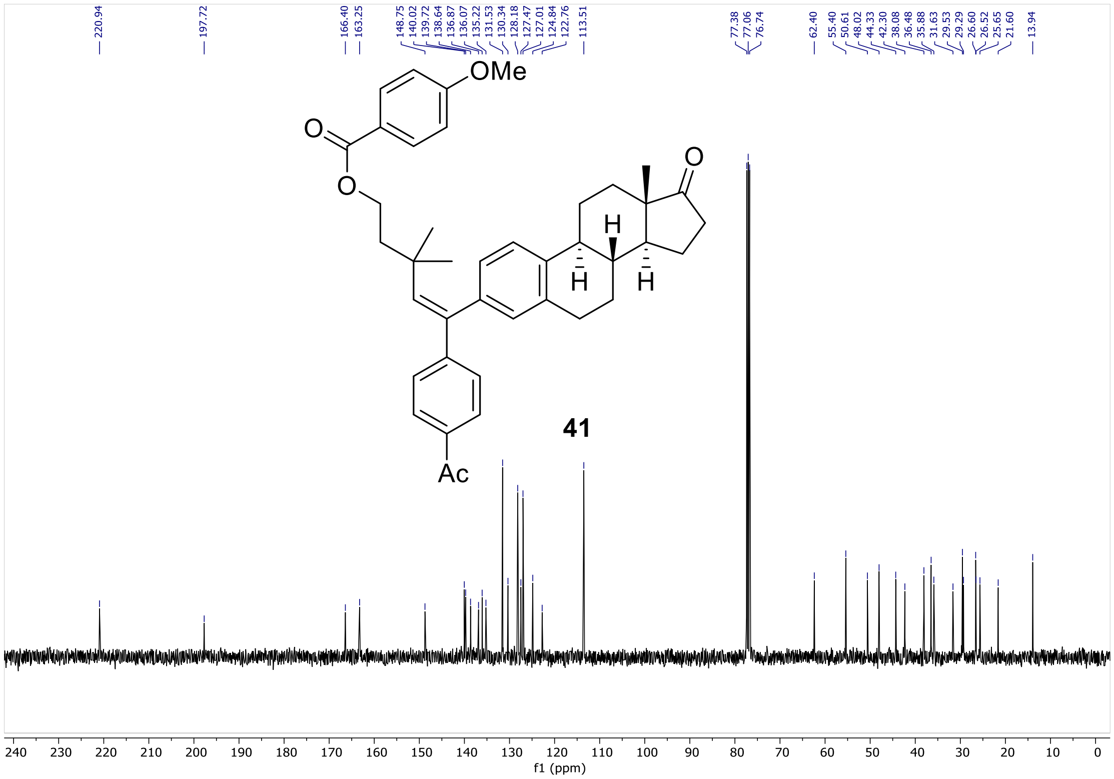

Supplementary Figure 114: ^{13}C NMR spectrum of compound **41**

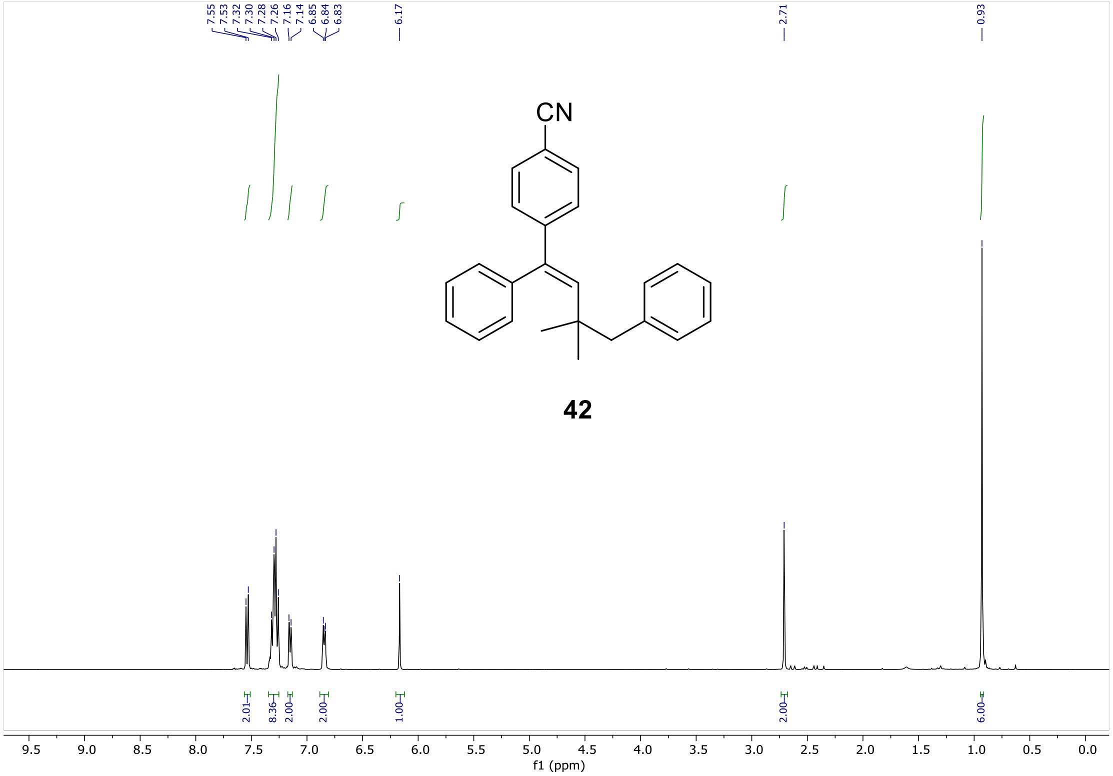

Supplementary Figure 115: ^{1}H NMR spectrum of compound **42**

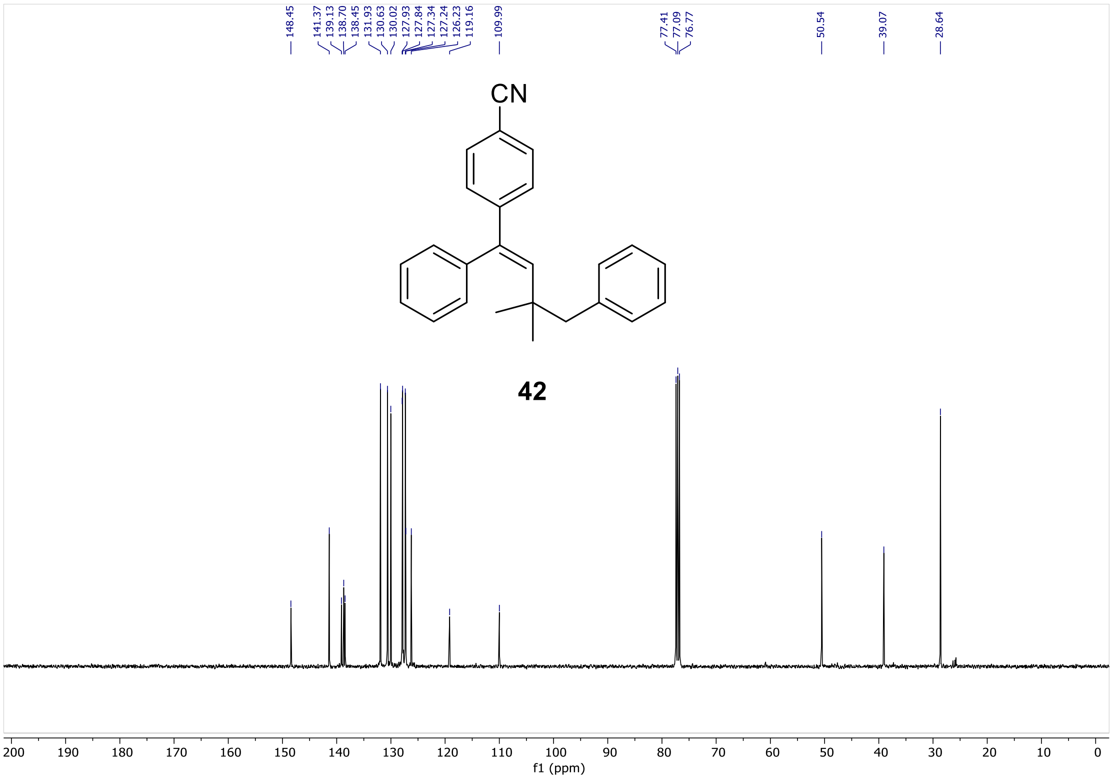

Supplementary Figure 116: ^{13}C NMR spectrum of compound **42**

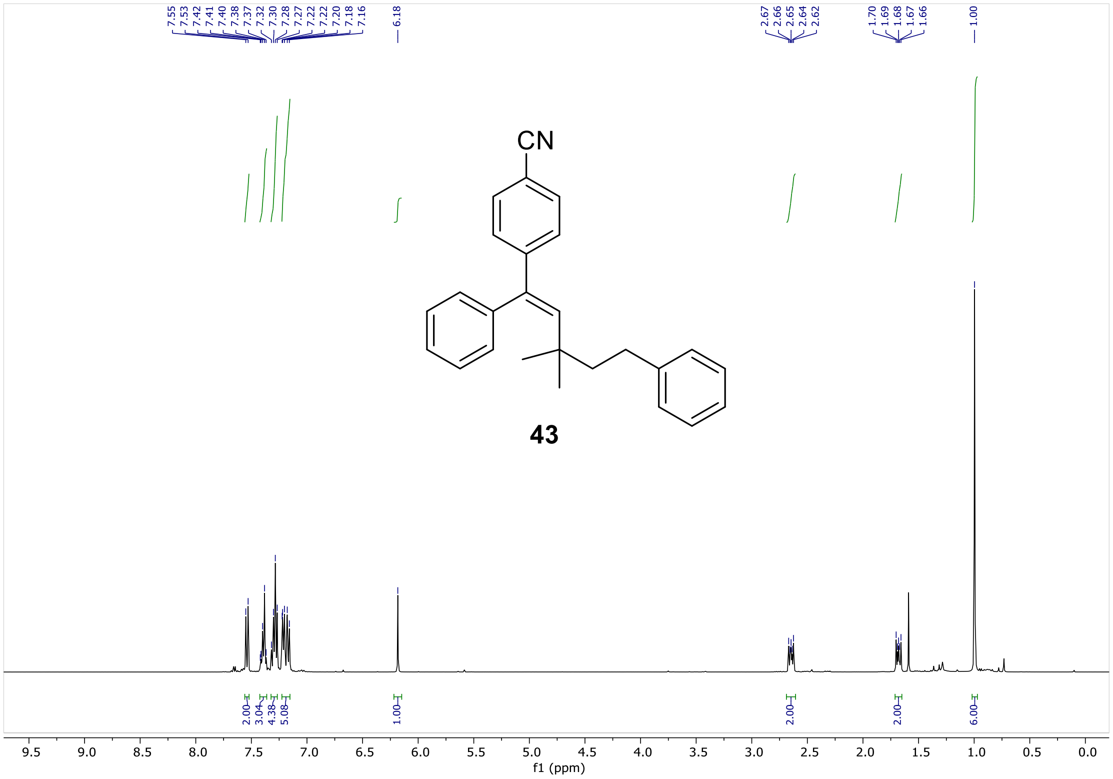

Supplementary Figure 117: ^{1}H NMR spectrum of compound **43**

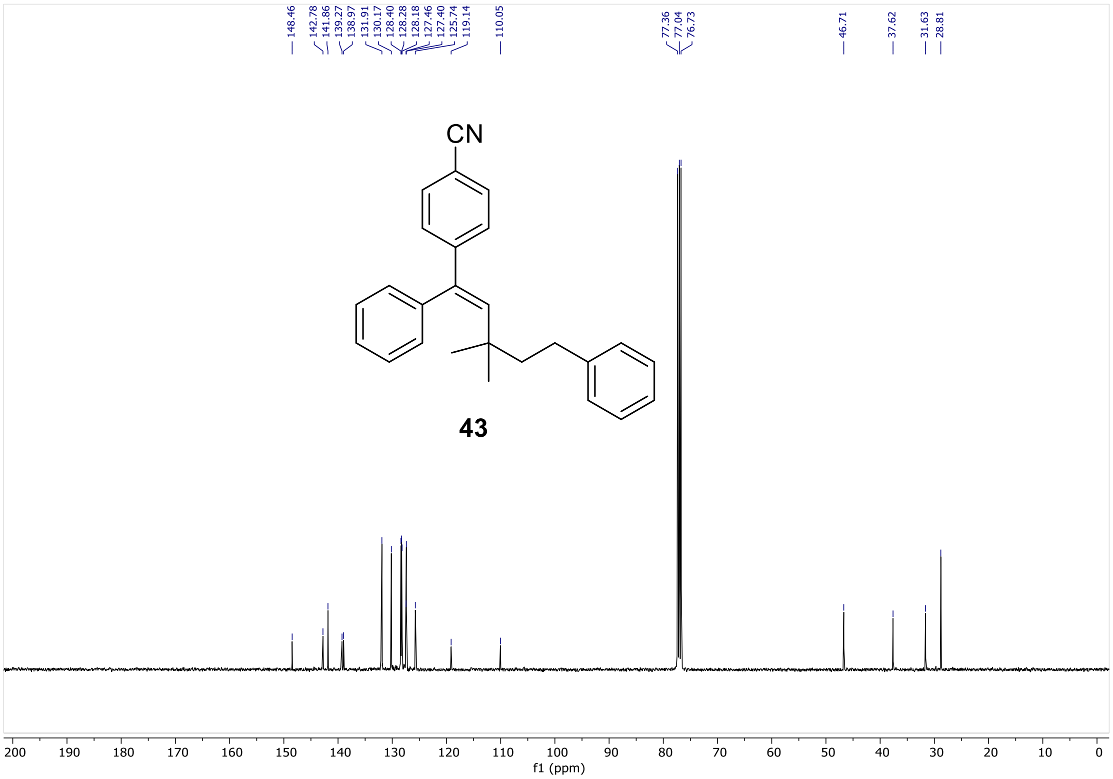

Supplementary Figure 118: ^{13}C NMR spectrum of compound **43**

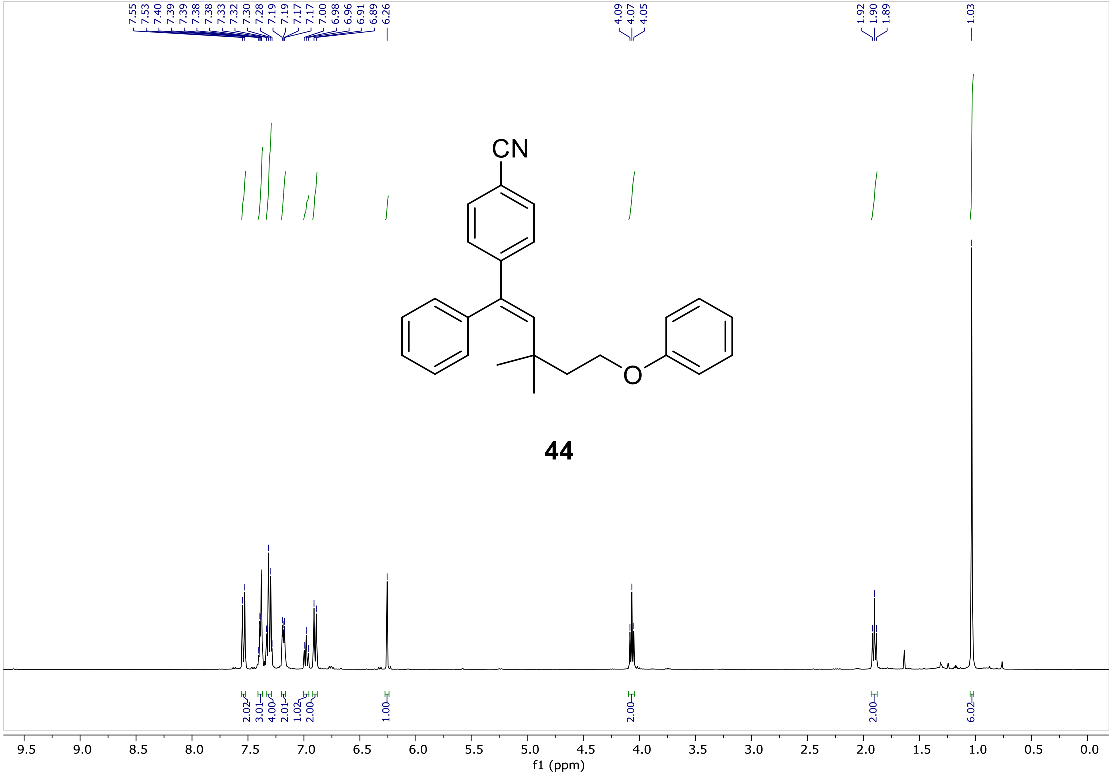

Supplementary Figure 119: ^{1}H NMR spectrum of compound **44**

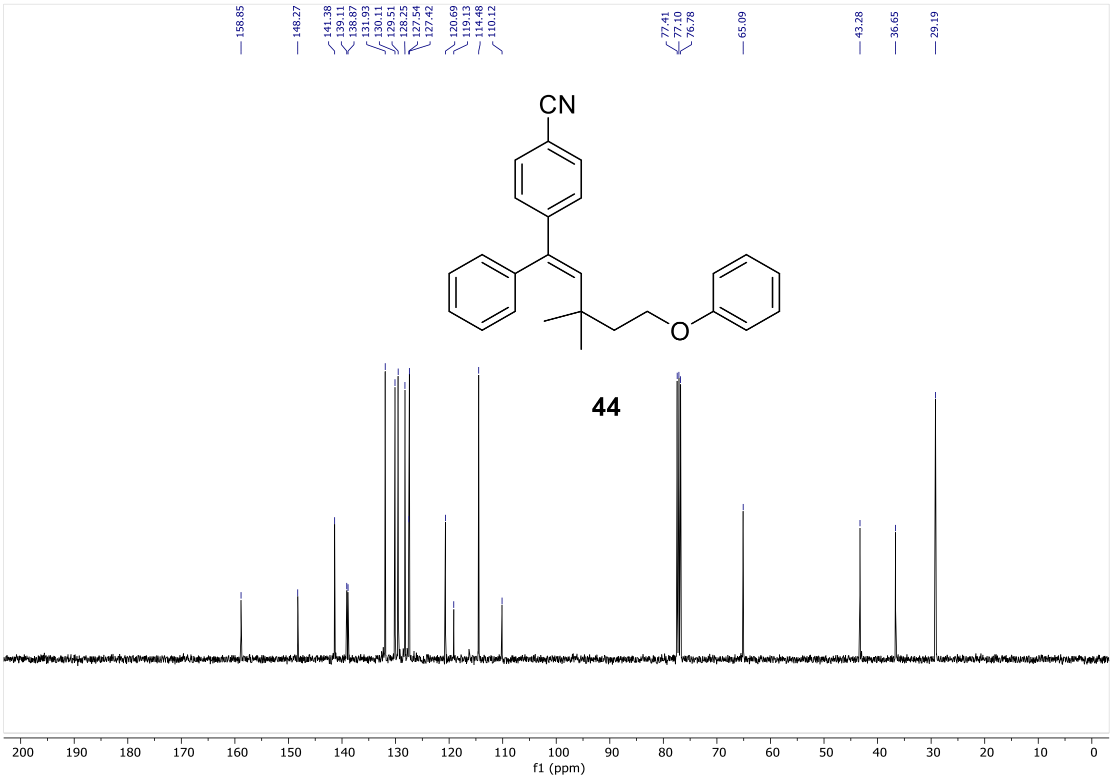

Supplementary Figure 120: ^{13}C NMR spectrum of compound **44**

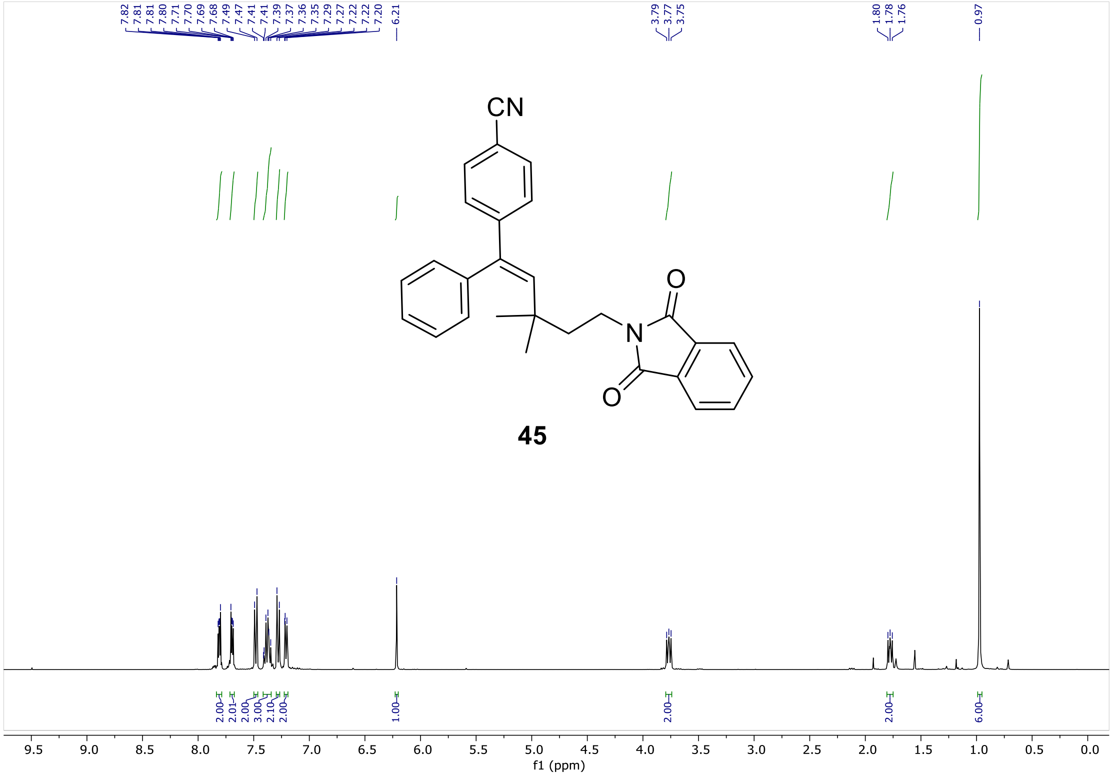

Supplementary Figure 121: ^{1}H NMR spectrum of compound **45**

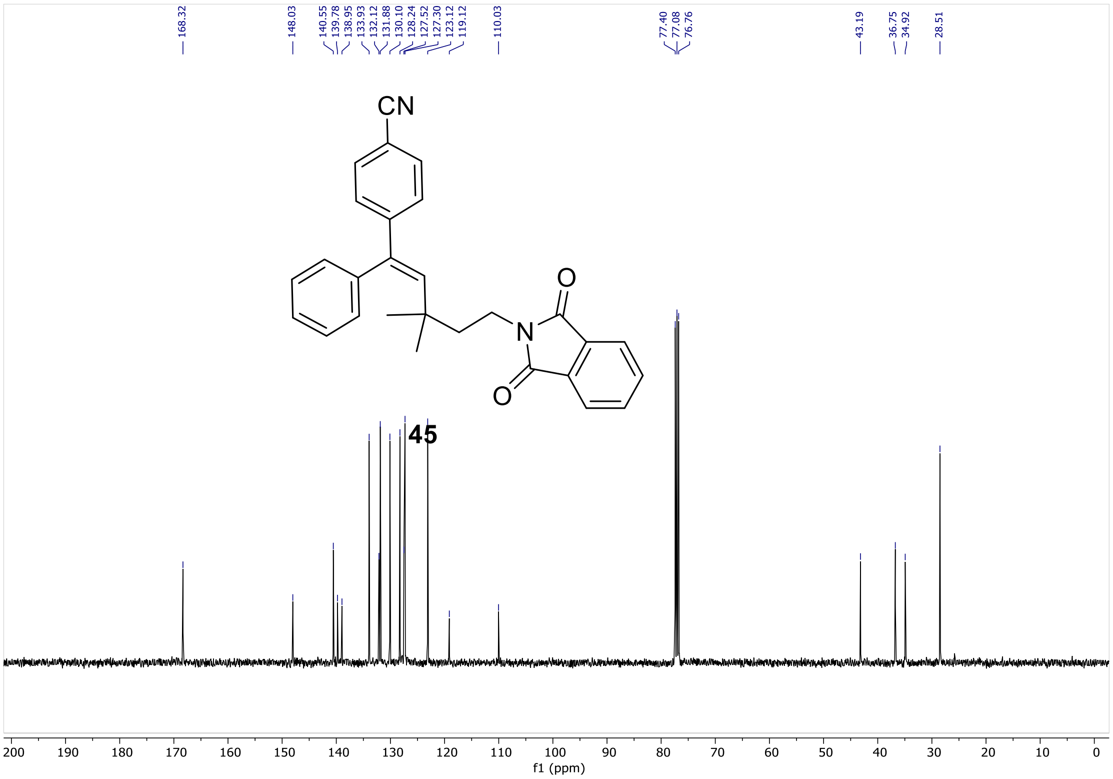

Supplementary Figure 122: ^{13}C NMR spectrum of compound **45**

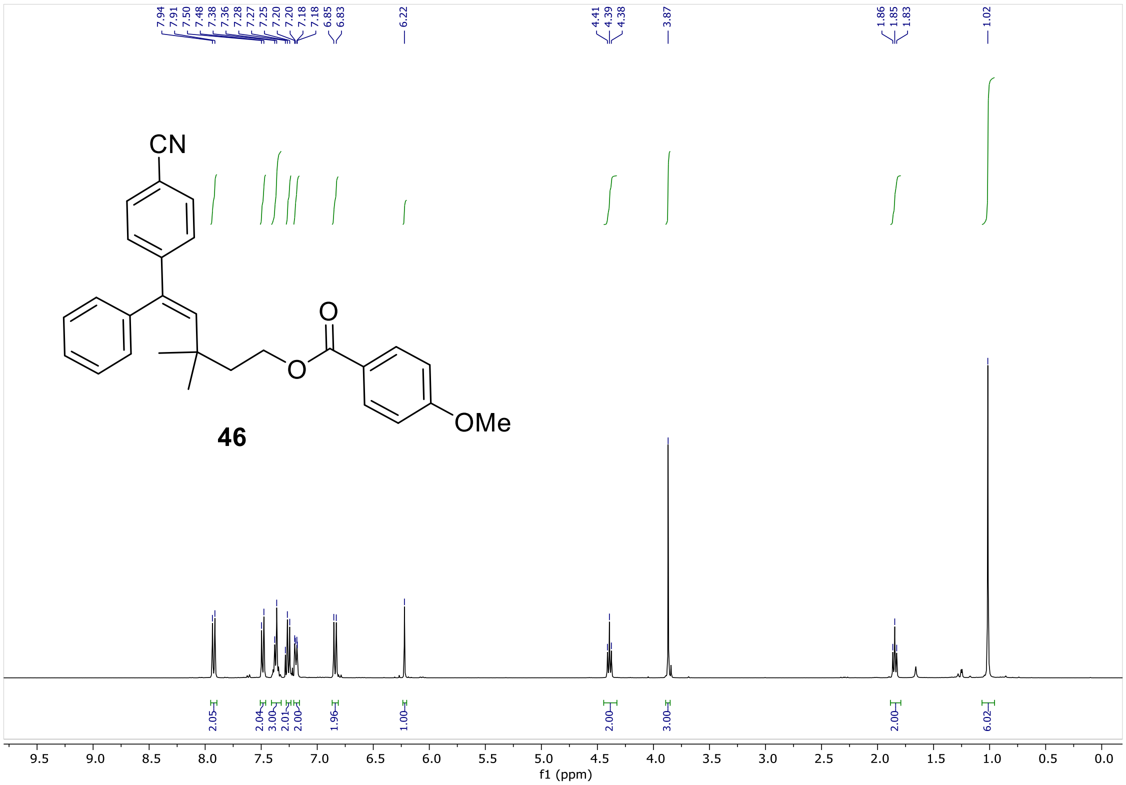

Supplementary Figure 123: ^{1}H NMR spectrum of compound **46**

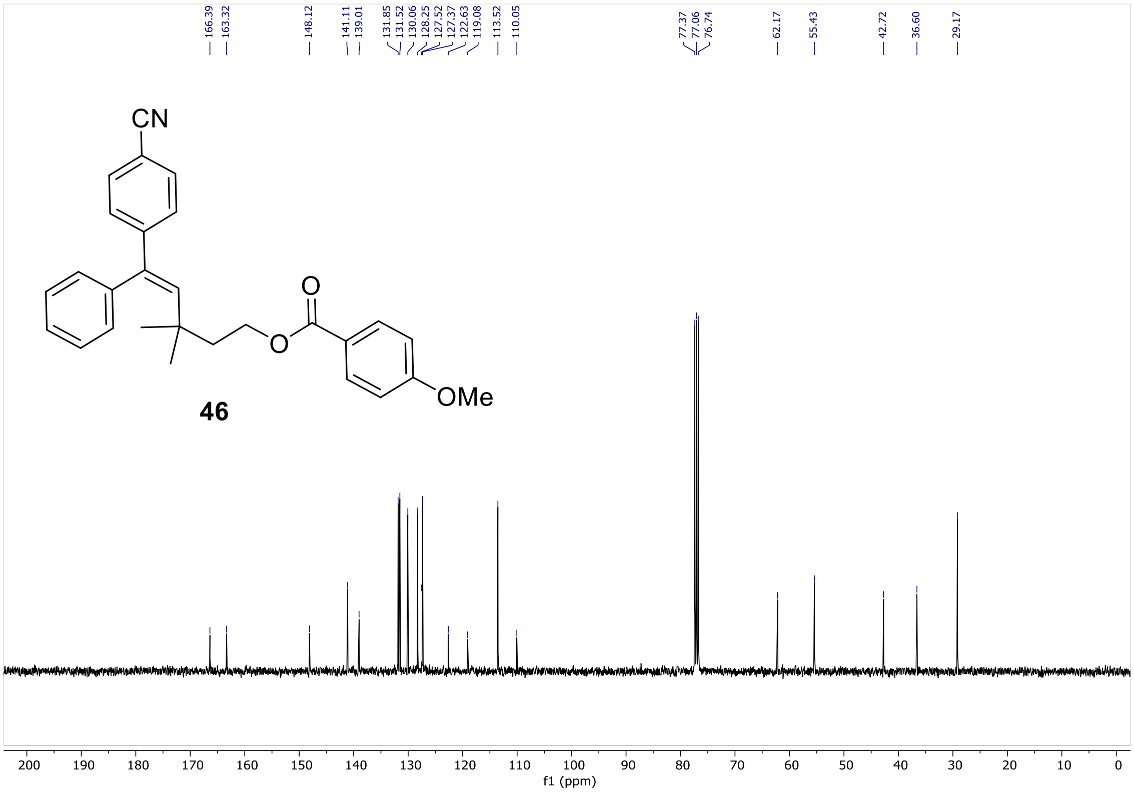

Supplementary Figure 124: ^{13}C NMR spectrum of compound **46**

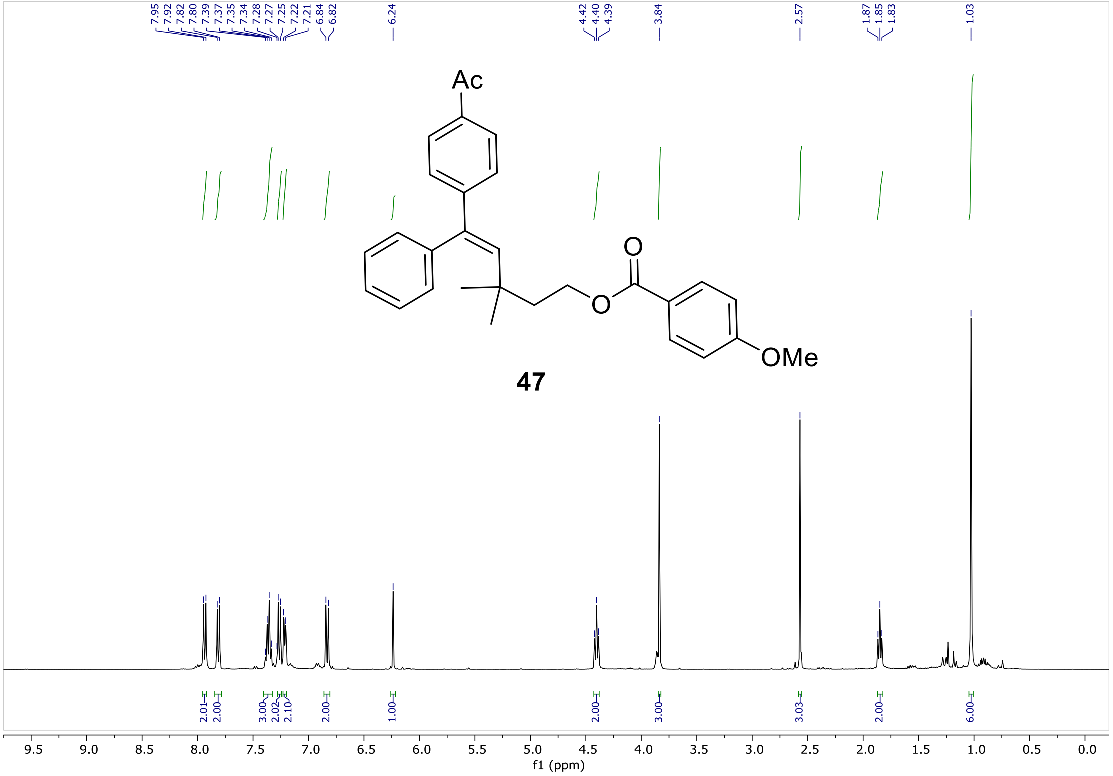

Supplementary Figure 125: ^{1}H NMR spectrum of compound **47**

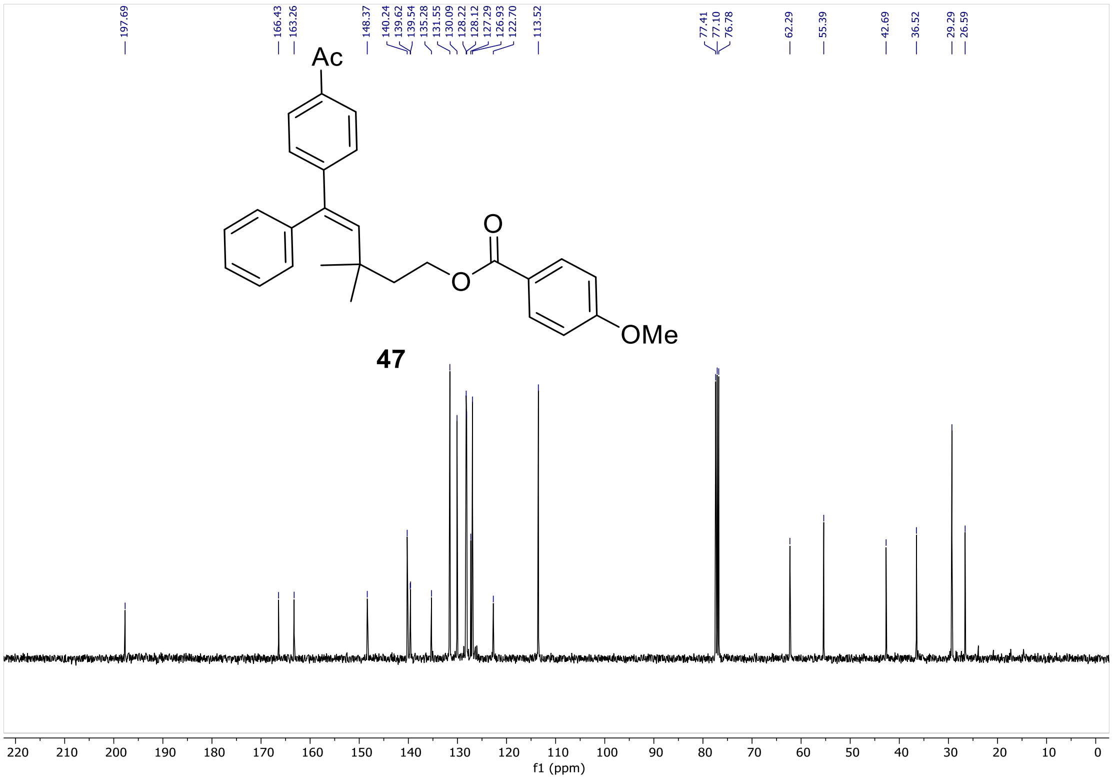

Supplementary Figure 126: ^{13}C NMR spectrum of compound **47**

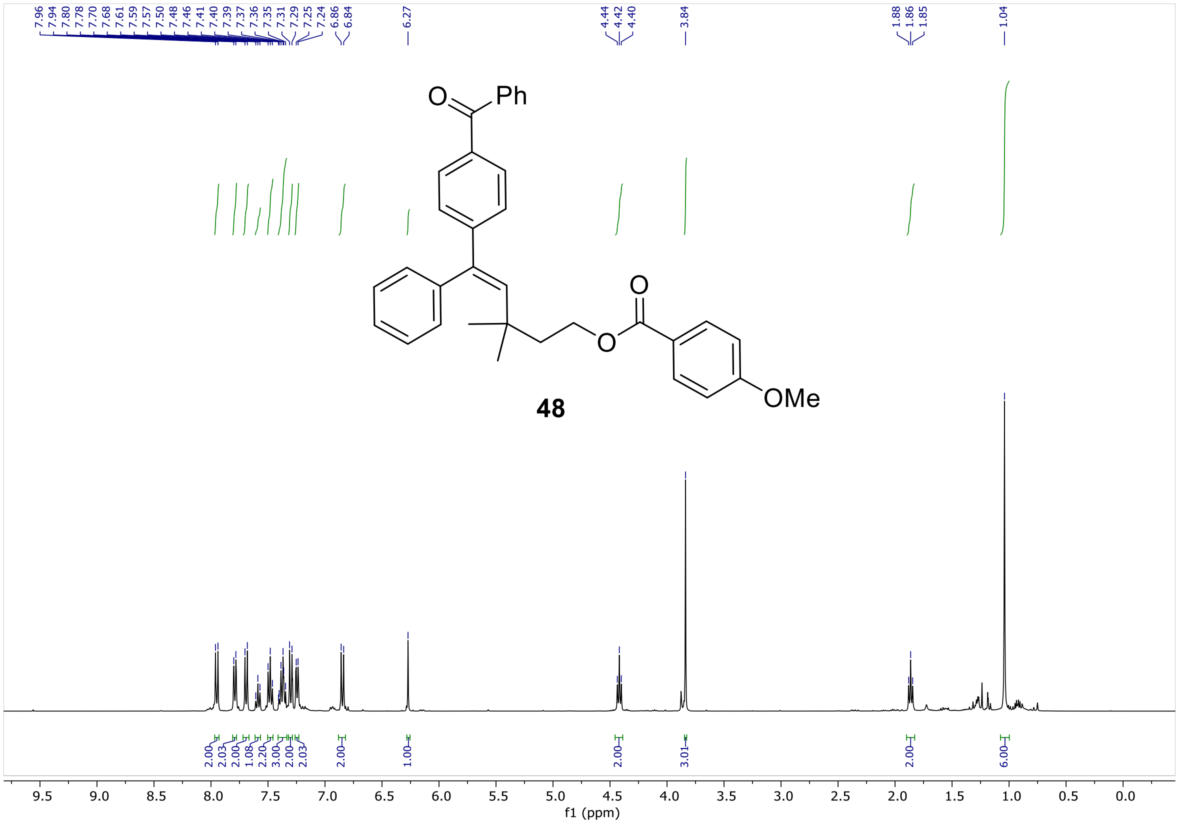

Supplementary Figure 127: ^{1}H NMR spectrum of compound **48**

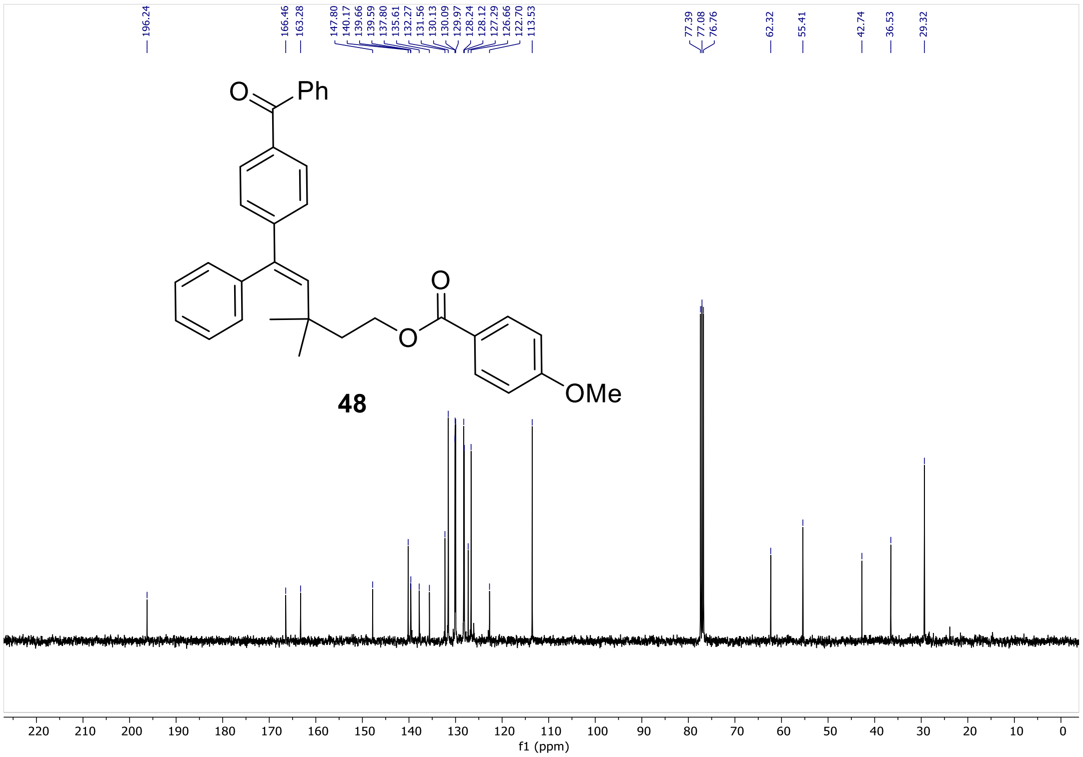

Supplementary Figure 128: ^{13}C NMR spectrum of compound **48**

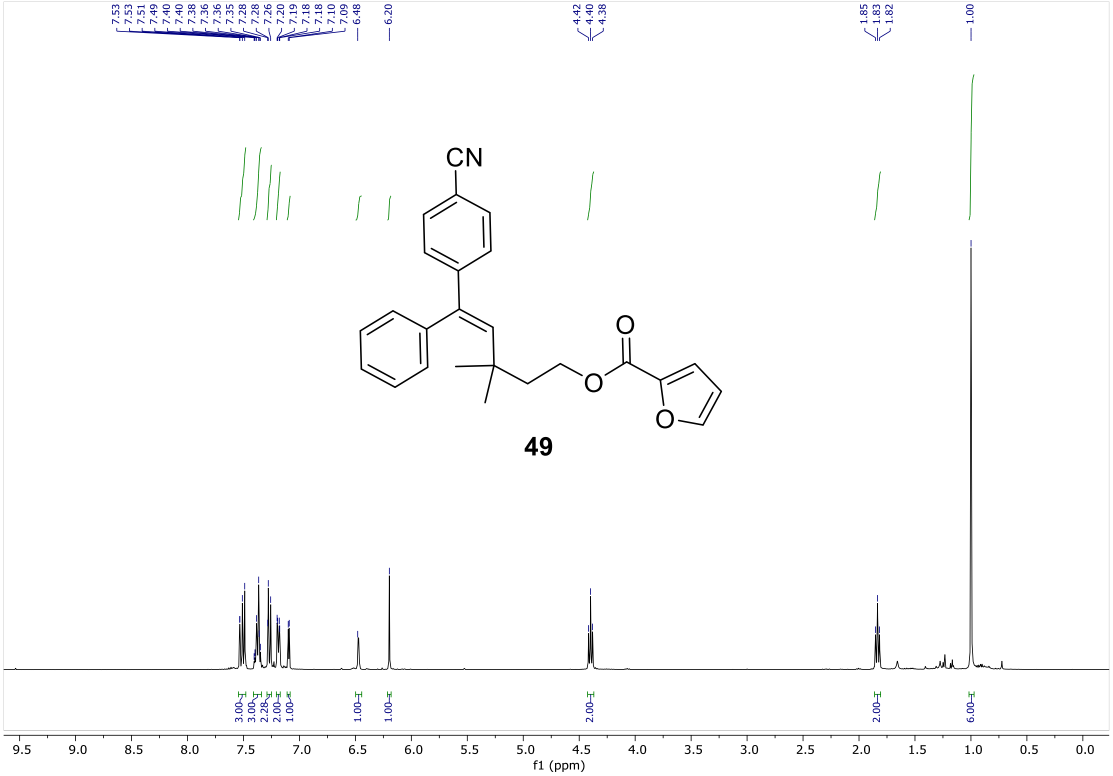

Supplementary Figure 129: ^{1}H NMR spectrum of compound **49**

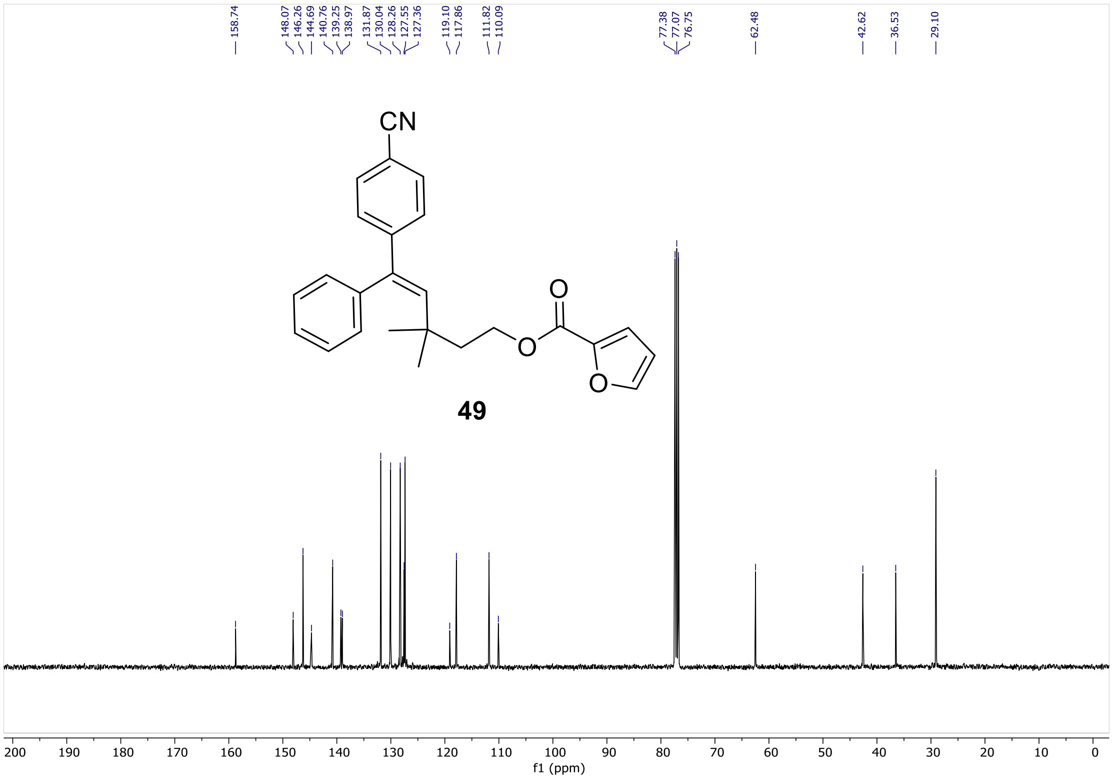

Supplementary Figure 130: ^{13}C NMR spectrum of compound **49**

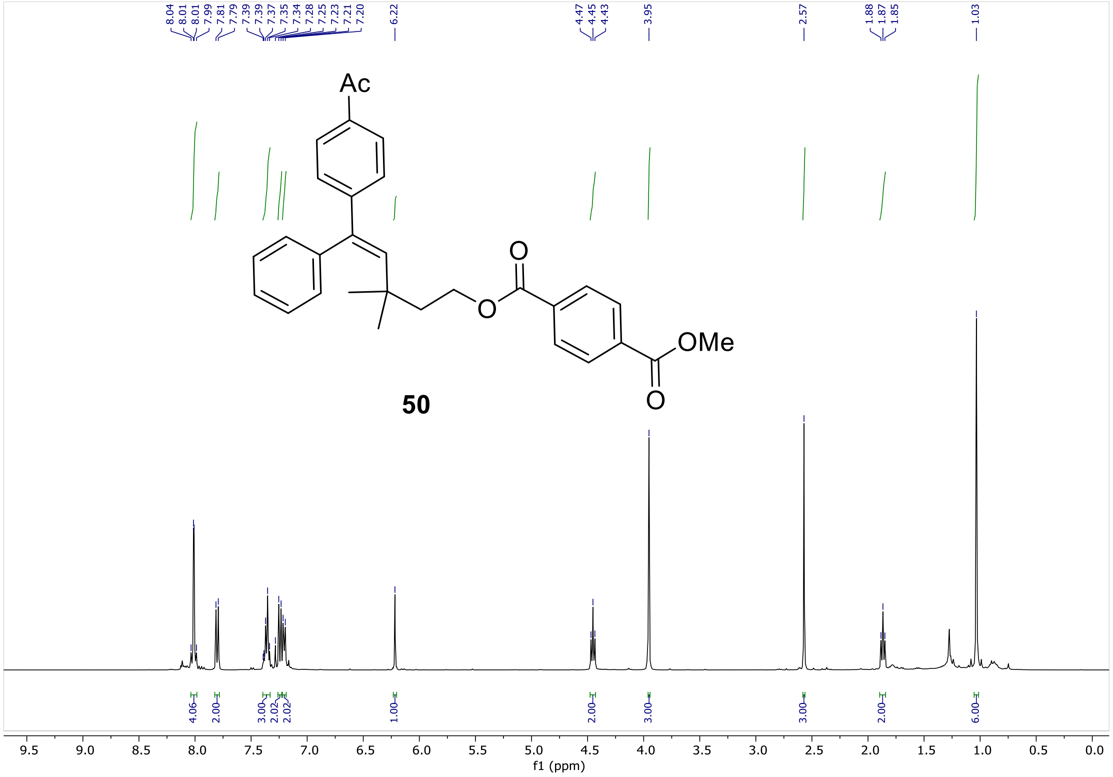

Supplementary Figure 131: ^{1}H NMR spectrum of compound **50**

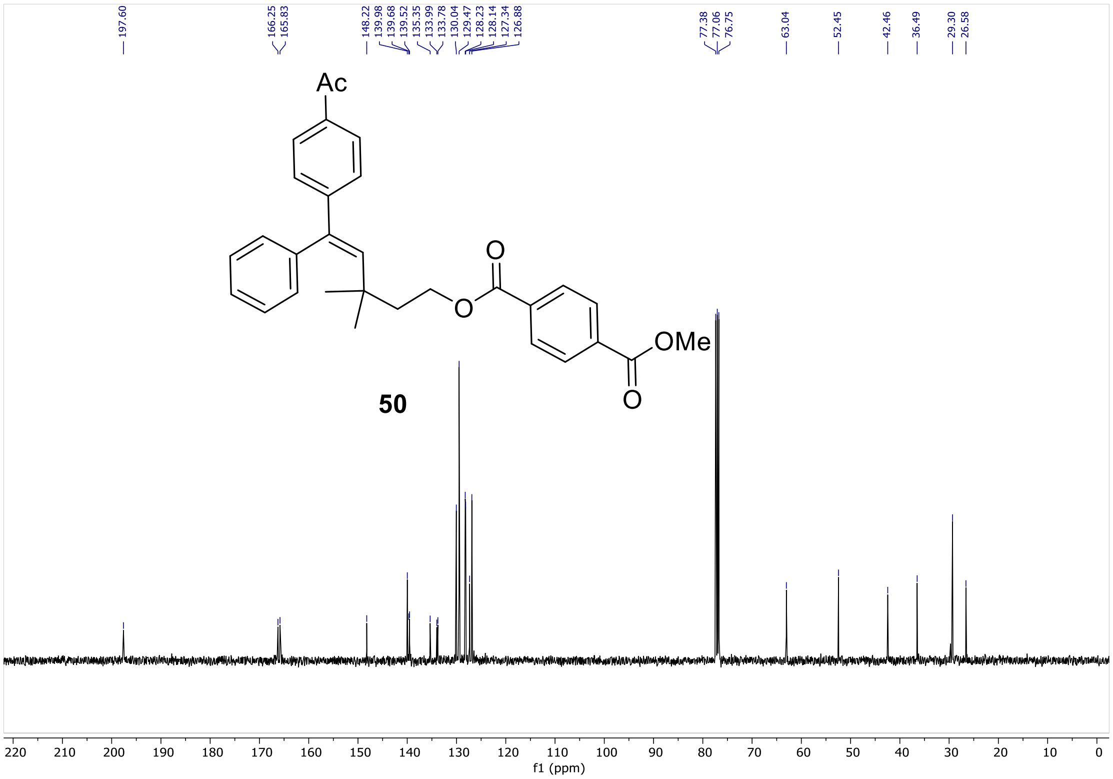

Supplementary Figure 132: ^{13}C NMR spectrum of compound **50**

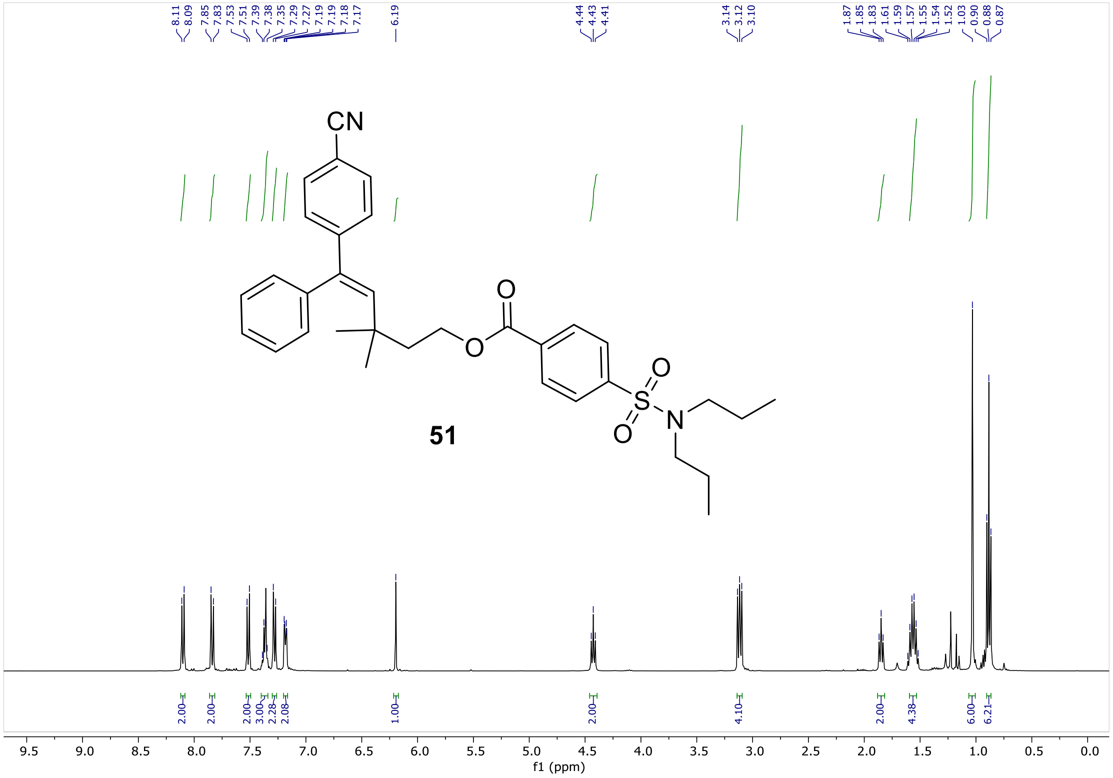

Supplementary Figure 133: ^{1}H NMR spectrum of compound **51**

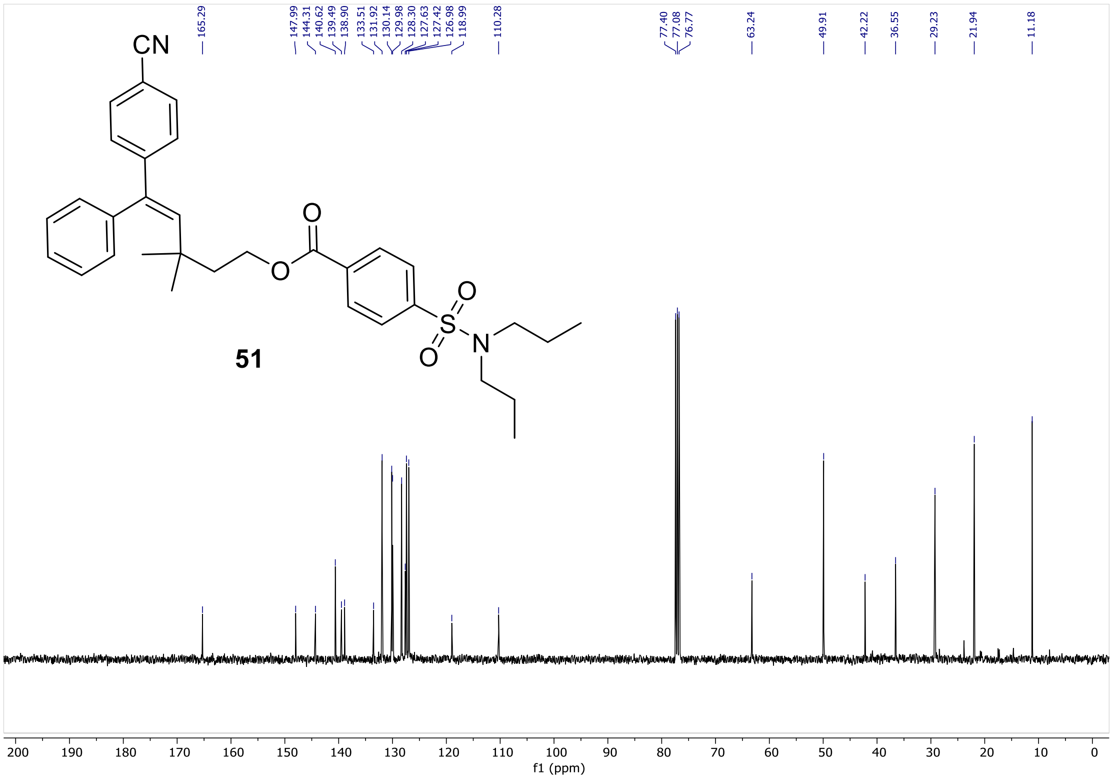

Supplementary Figure 134: ^{13}C NMR spectrum of compound **51**

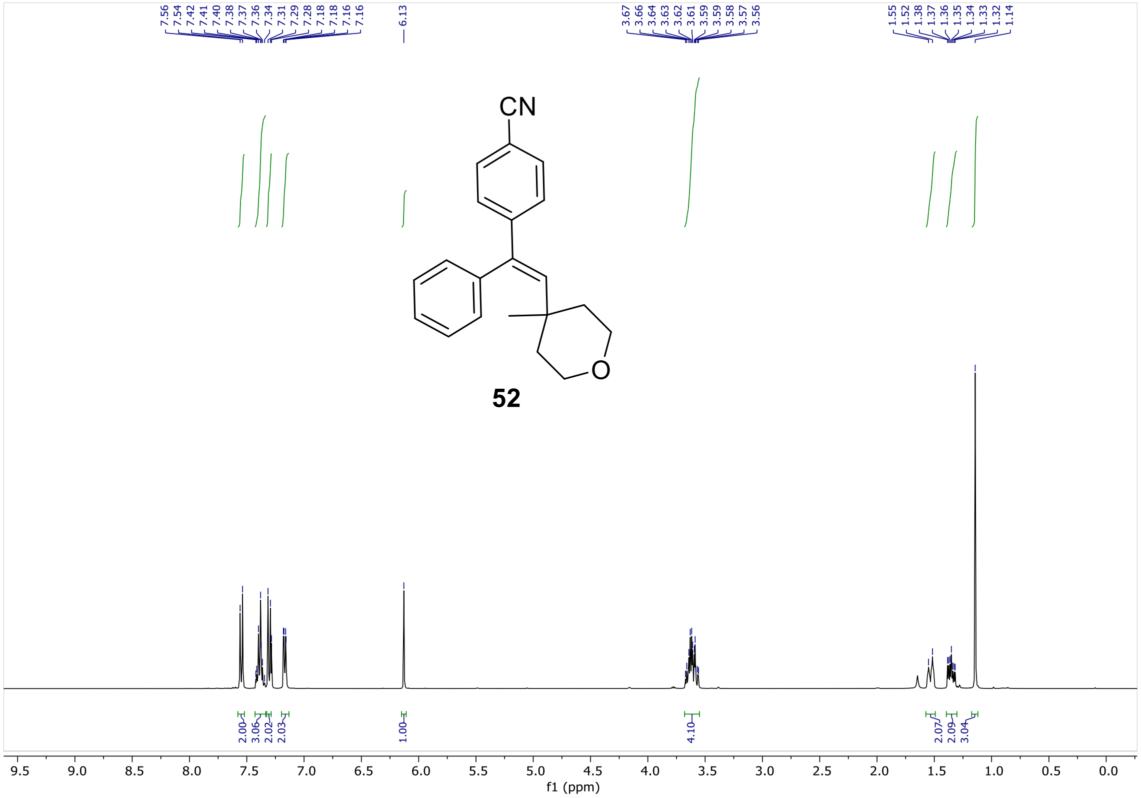

Supplementary Figure 135: ^{1}H NMR spectrum of compound **52**

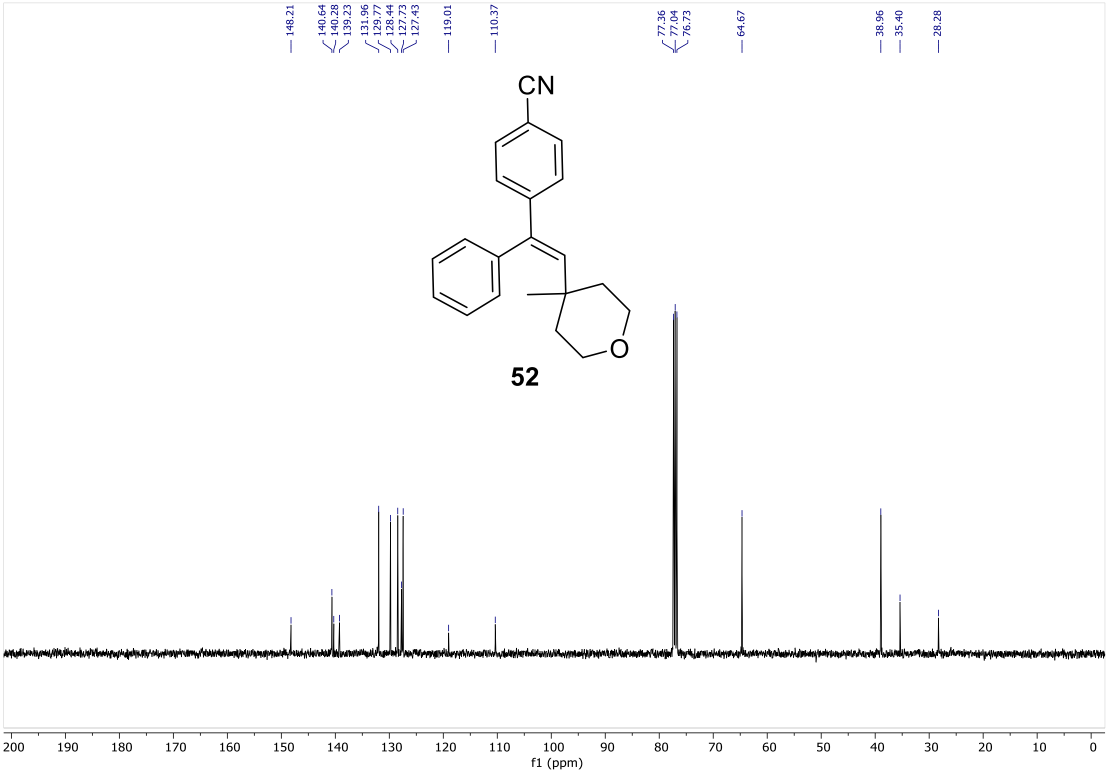

Supplementary Figure 136: ^{13}C NMR spectrum of compound **52**

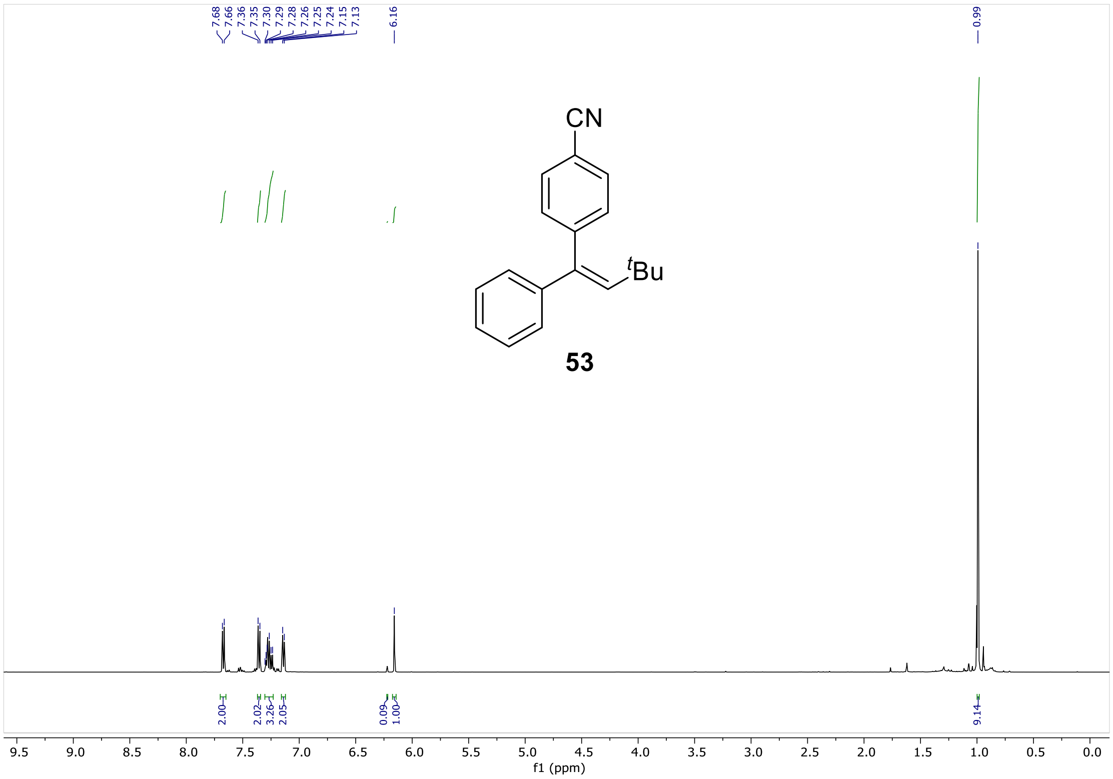

Supplementary Figure 137: ^{1}H NMR spectrum of compound **53**

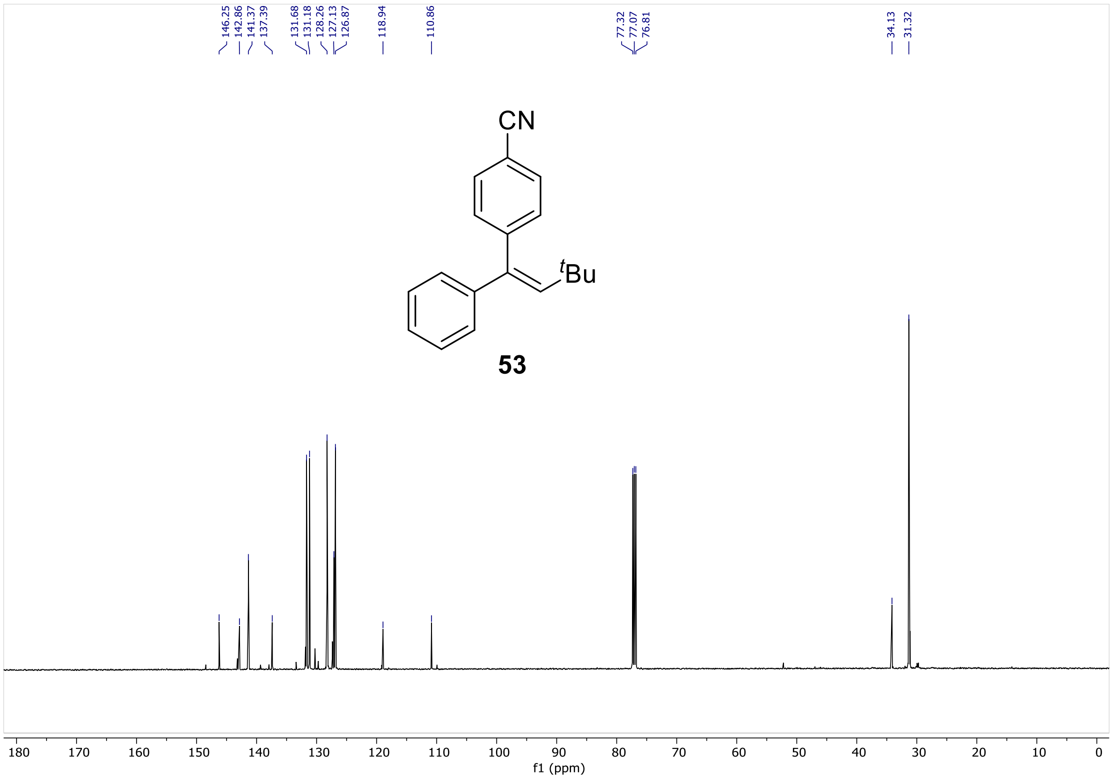

Supplementary Figure 138: ^{13}C NMR spectrum of compound **53**

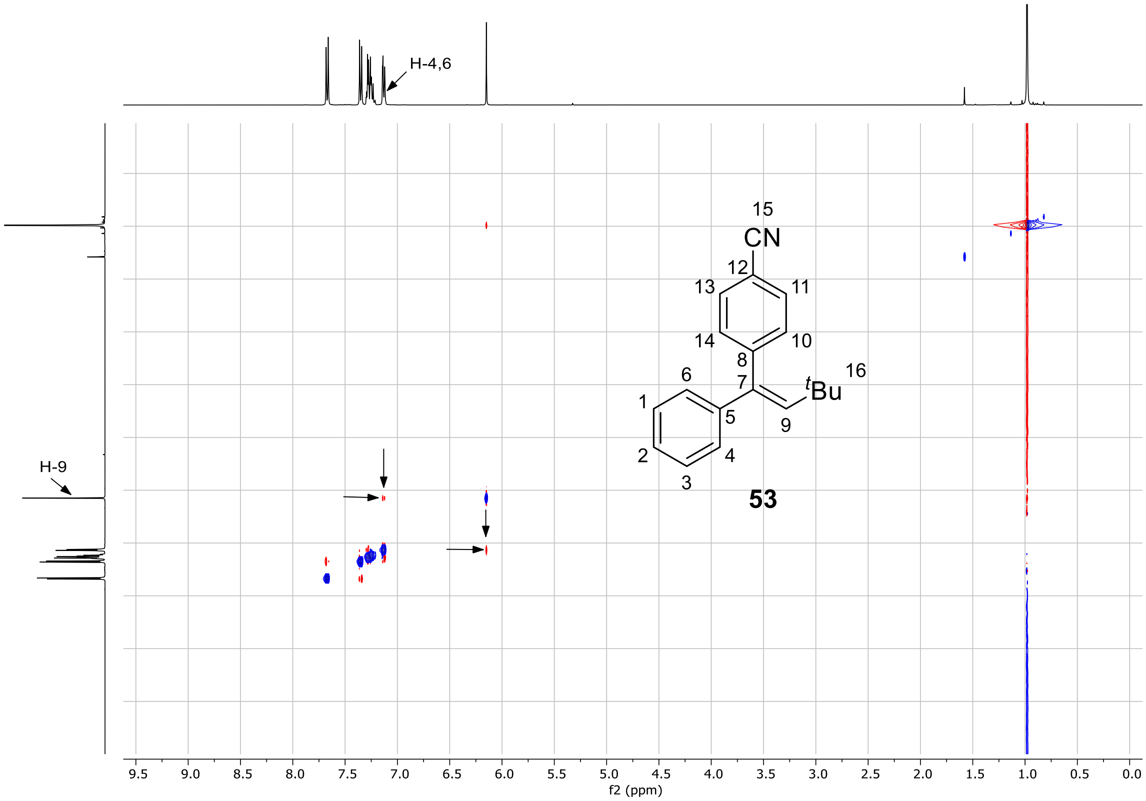

Supplementary Figure 139: NOE spectrum of compound **53**

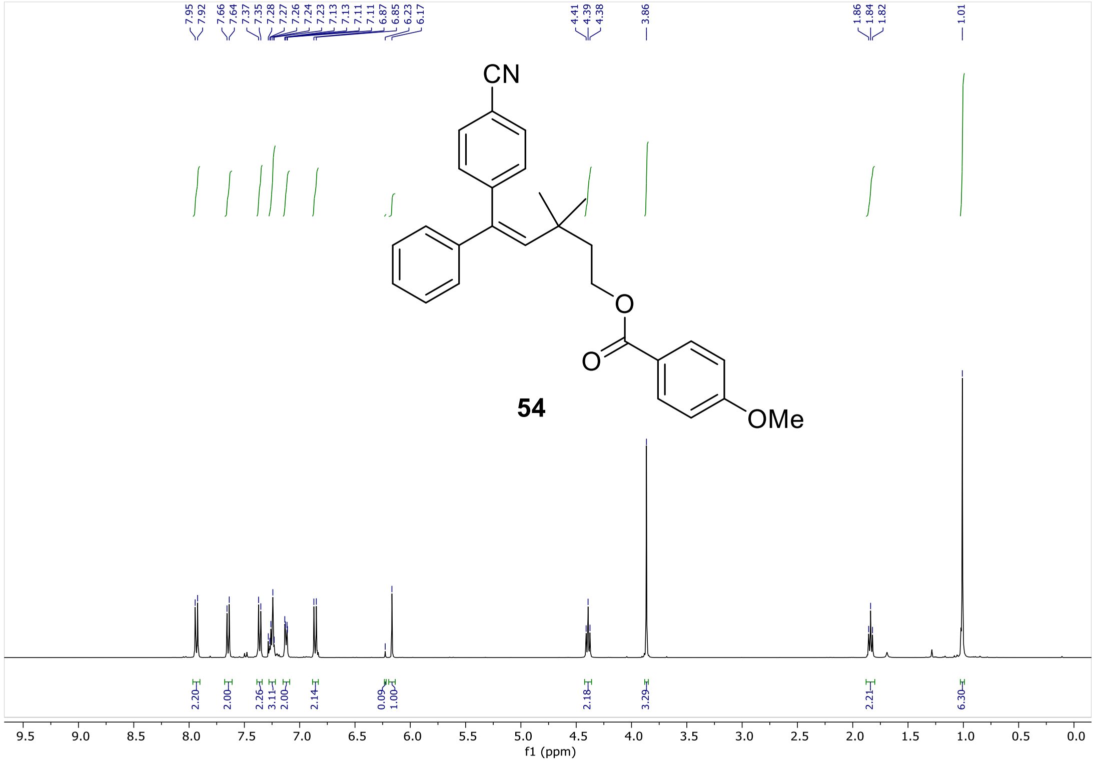

Supplementary Figure 140: ^{1}H NMR spectrum of compound **54**

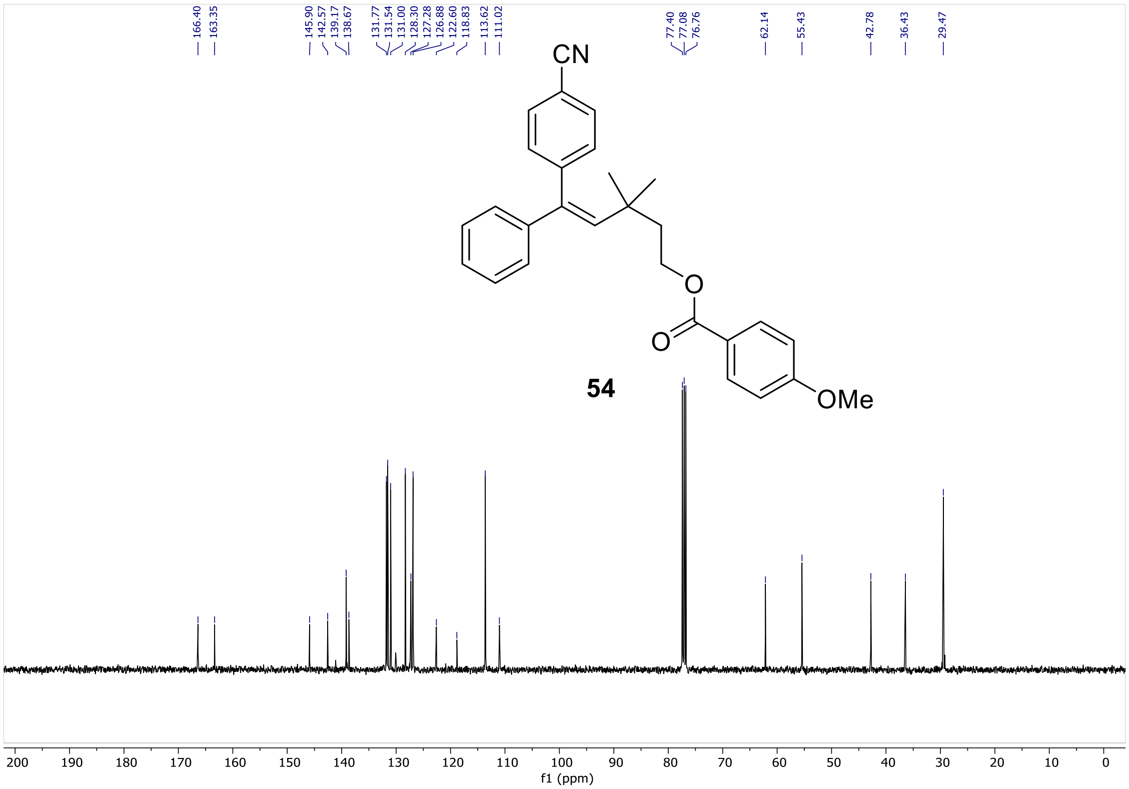

Supplementary Figure 141: ^{13}C NMR spectrum of compound **54**

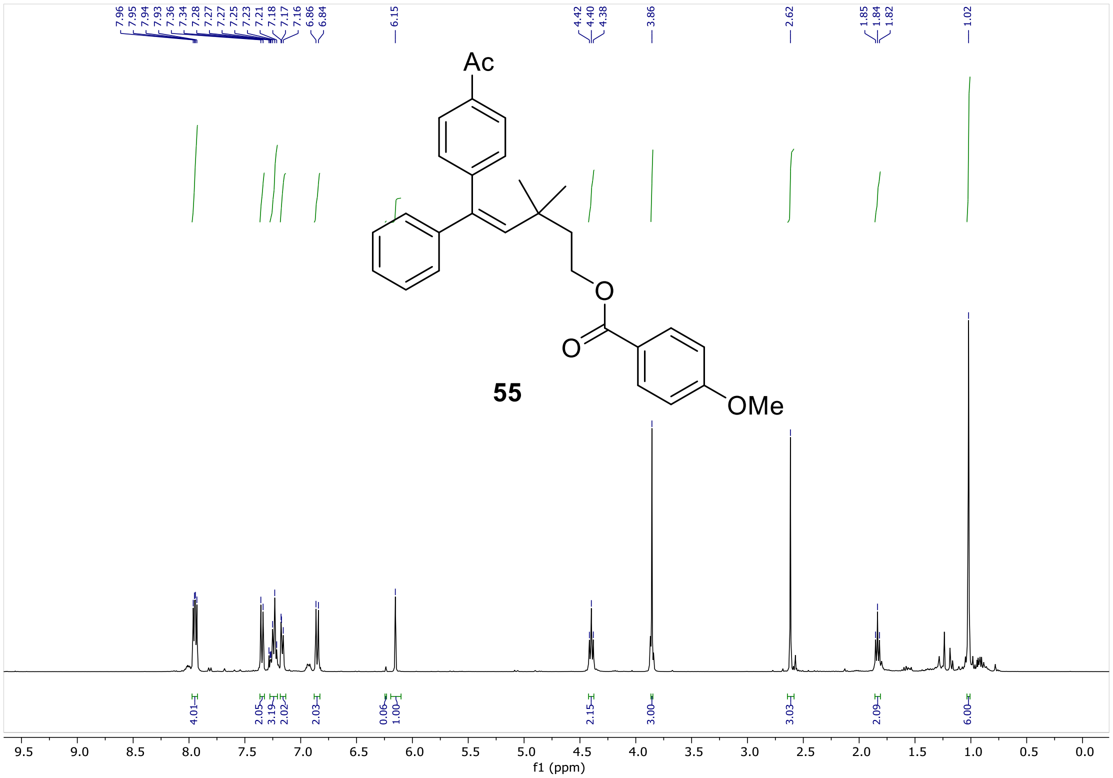

Supplementary Figure 142: ^{1}H NMR spectrum of compound **55**

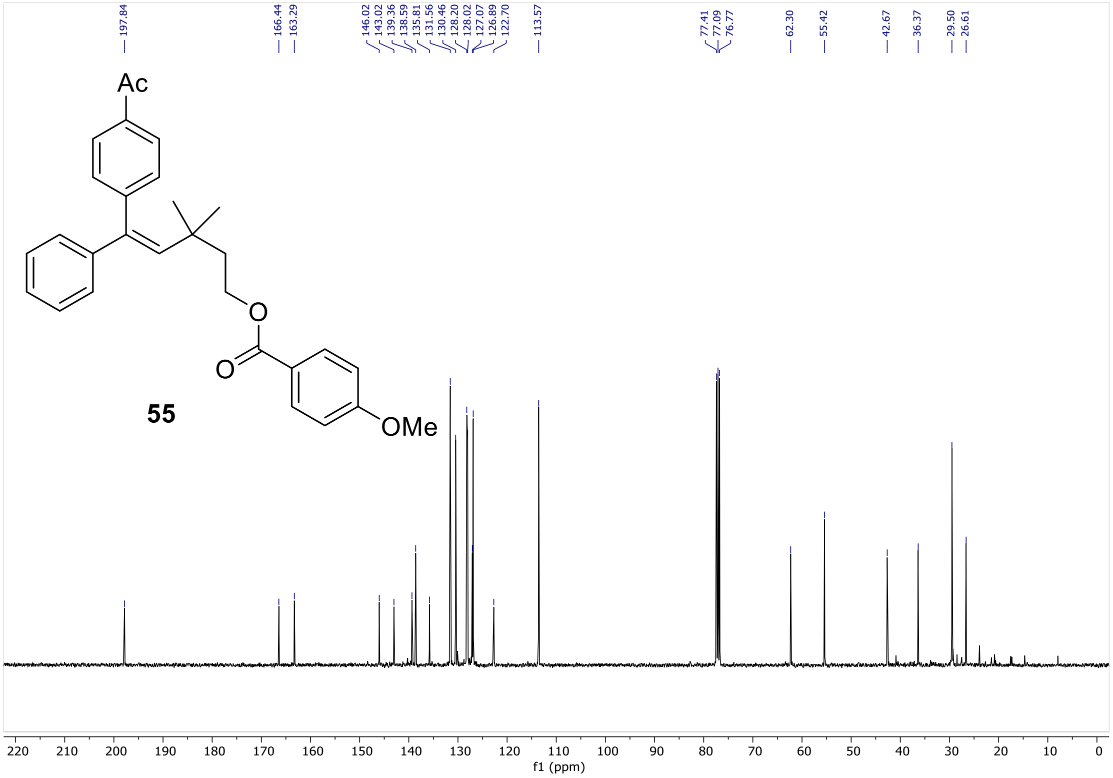

Supplementary Figure 143: ^{13}C NMR spectrum of compound **55**

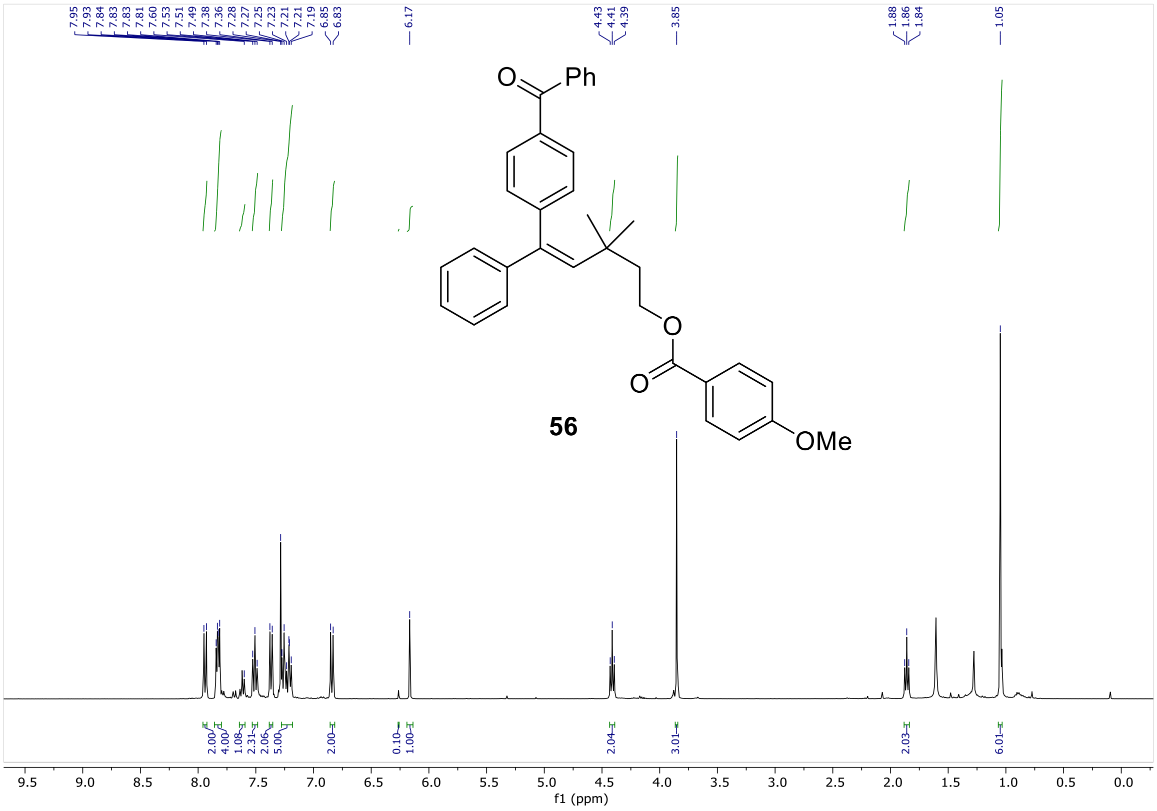

Supplementary Figure 144: ^{1}H NMR spectrum of compound **56**

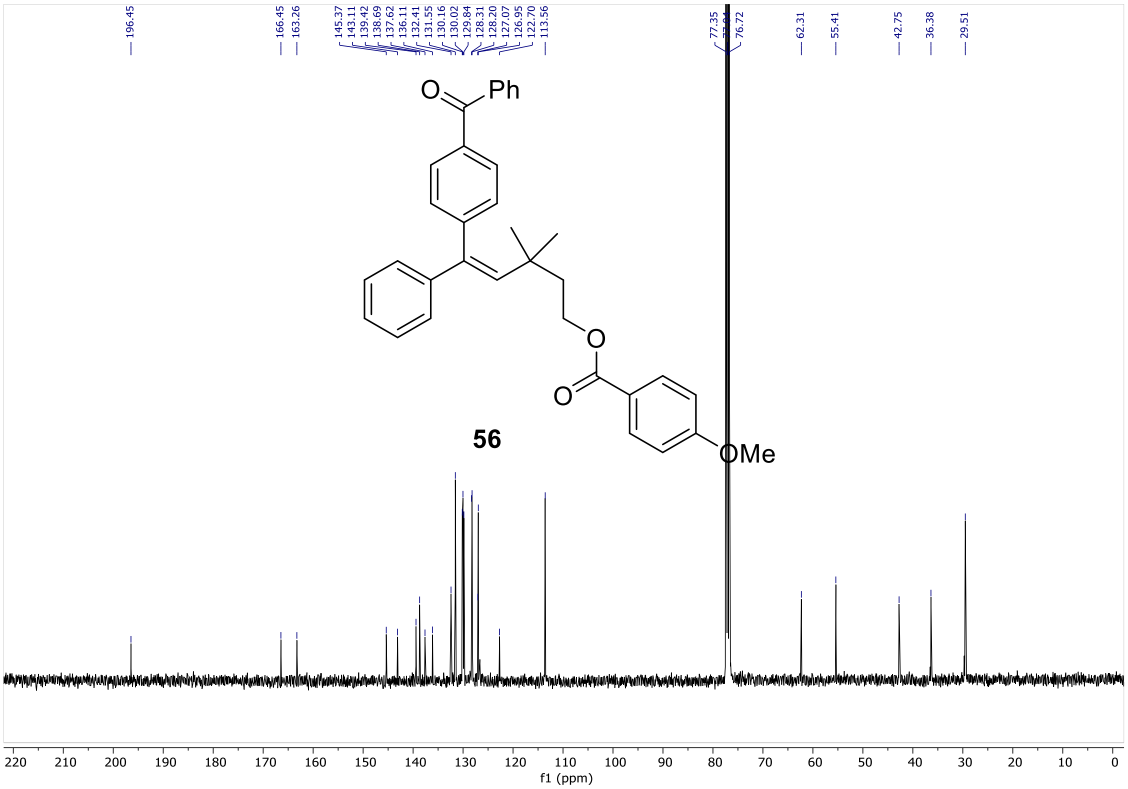

Supplementary Figure 145: ^{13}C NMR spectrum of compound **56**

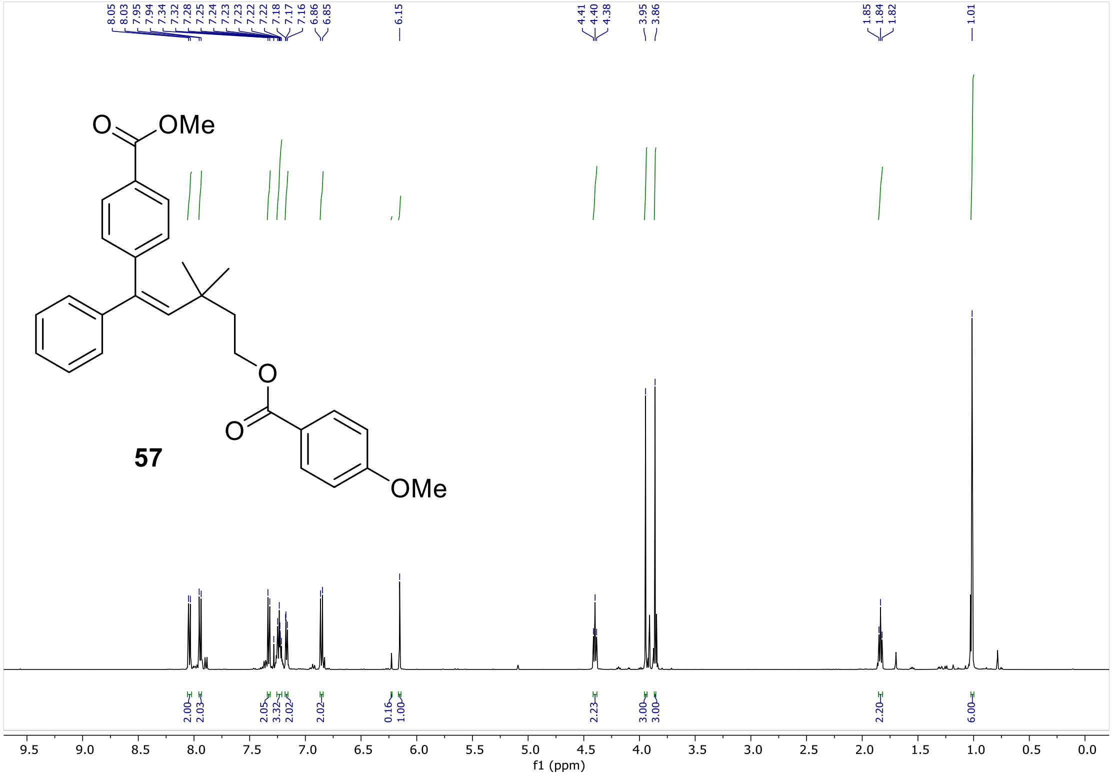

Supplementary Figure 146: ^{1}H NMR spectrum of compound **57**

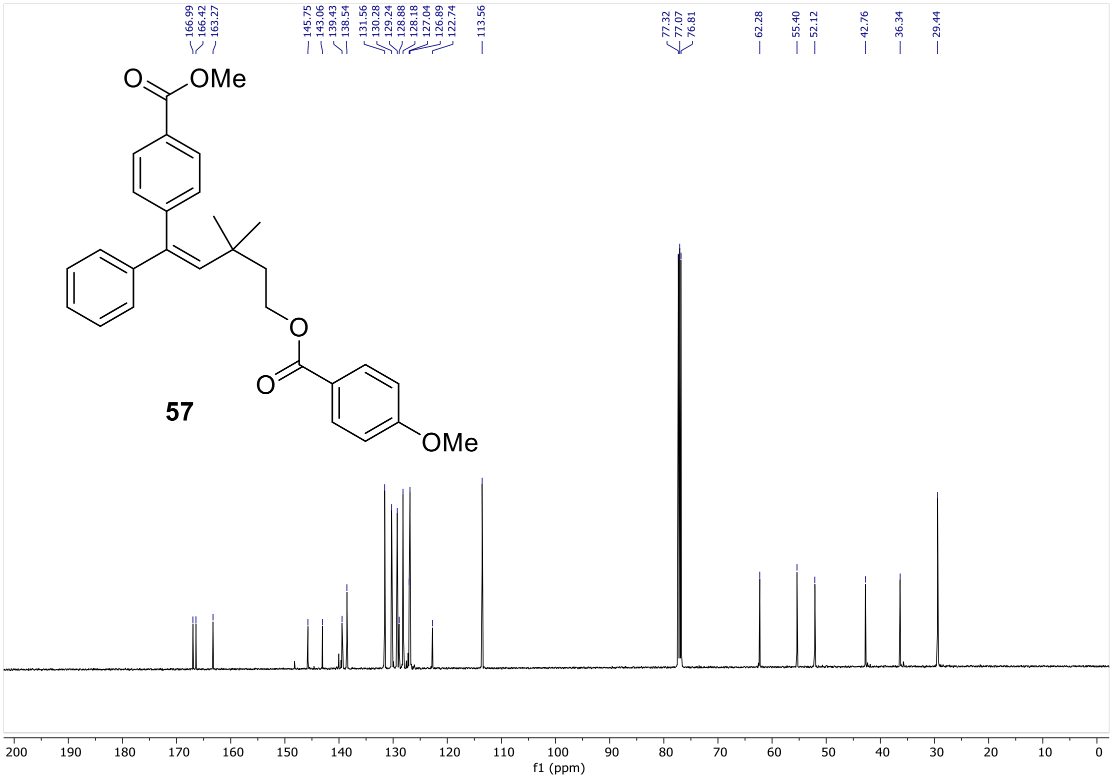

Supplementary Figure 147: ^{13}C NMR spectrum of compound **57**

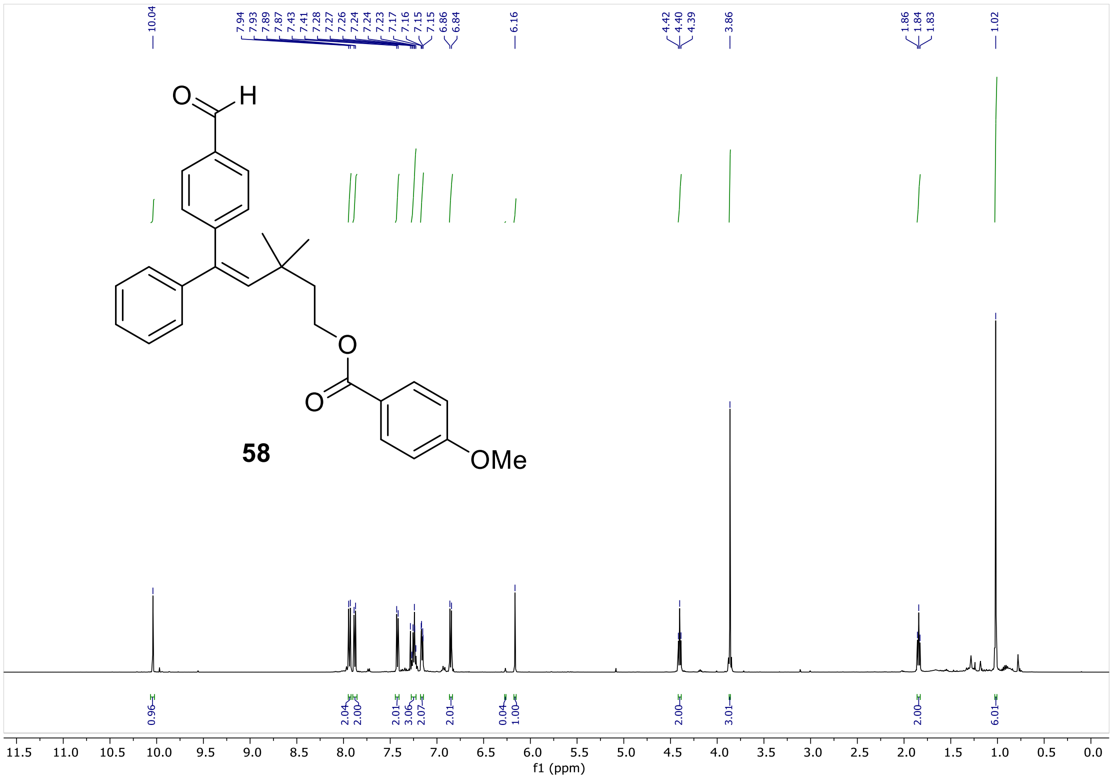

Supplementary Figure 148: ^{1}H NMR spectrum of compound **58**

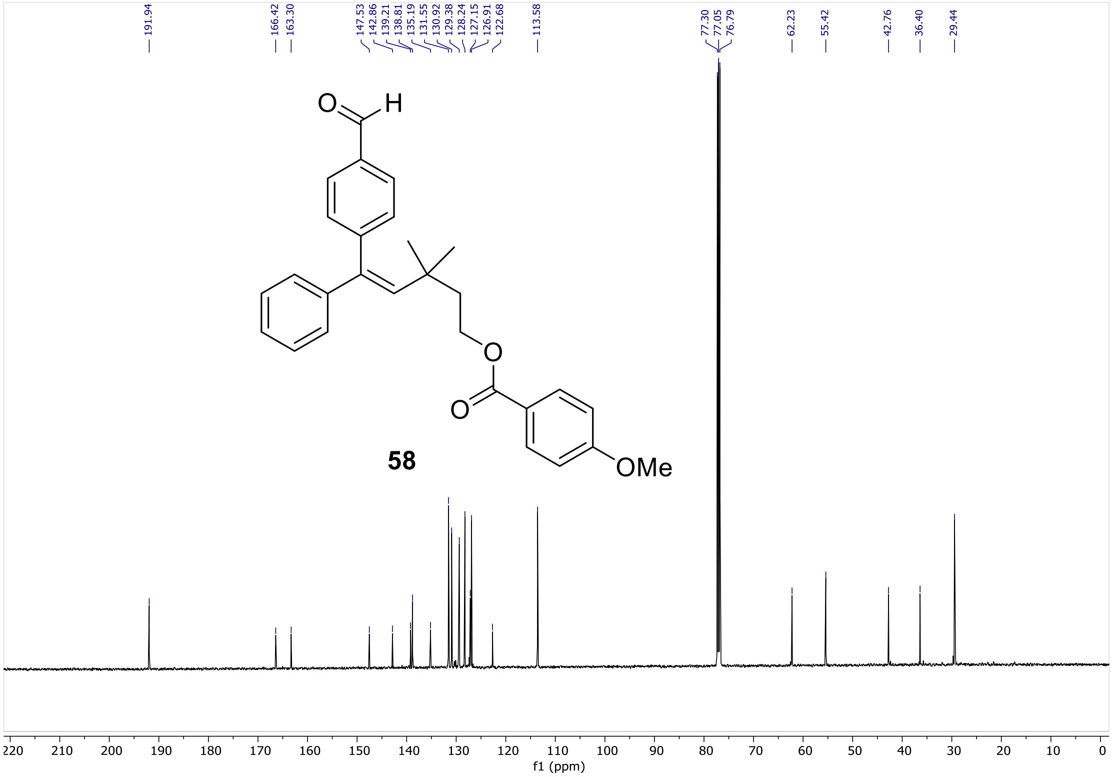

Supplementary Figure 149: ^{13}C NMR spectrum of compound **58**

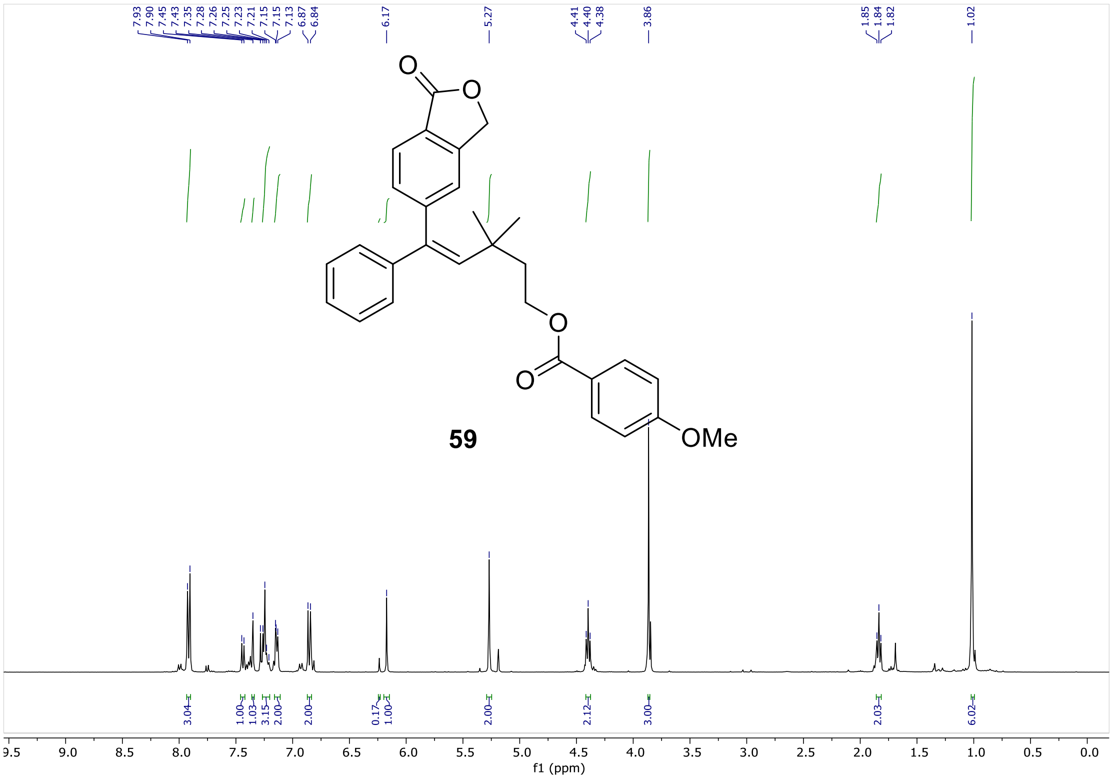

Supplementary Figure 150: ^{1}H NMR spectrum of compound **59**

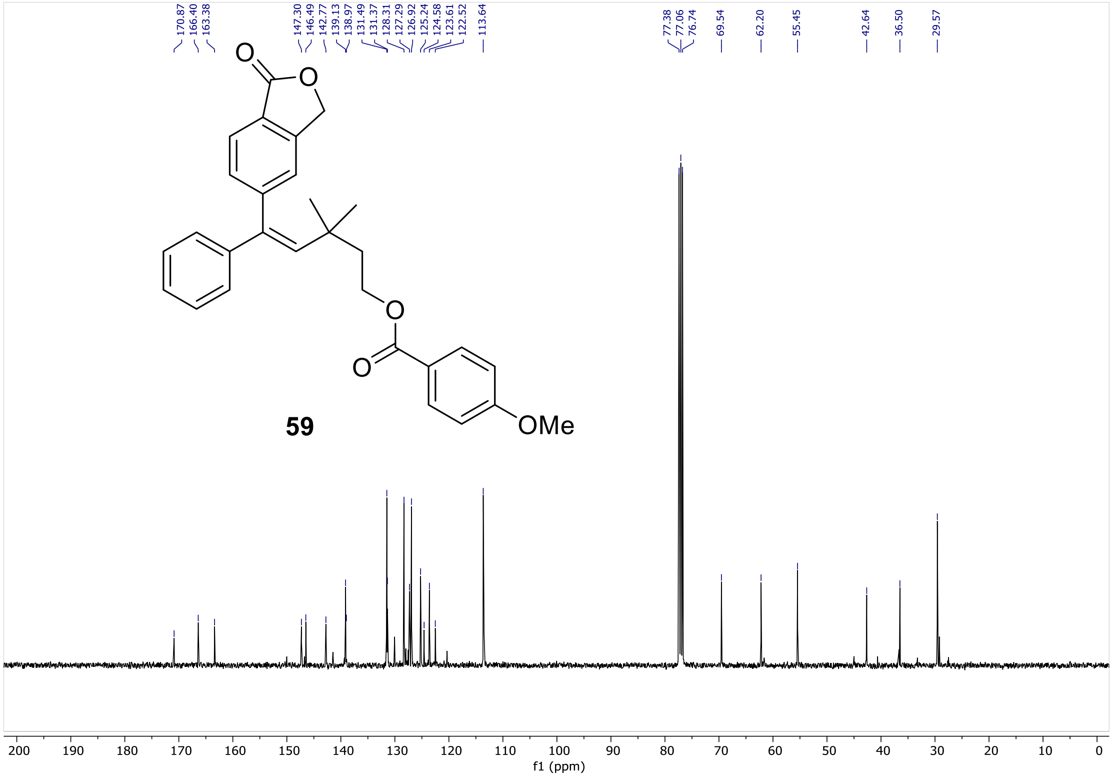

Supplementary Figure 151: ^{13}C NMR spectrum of compound **59**

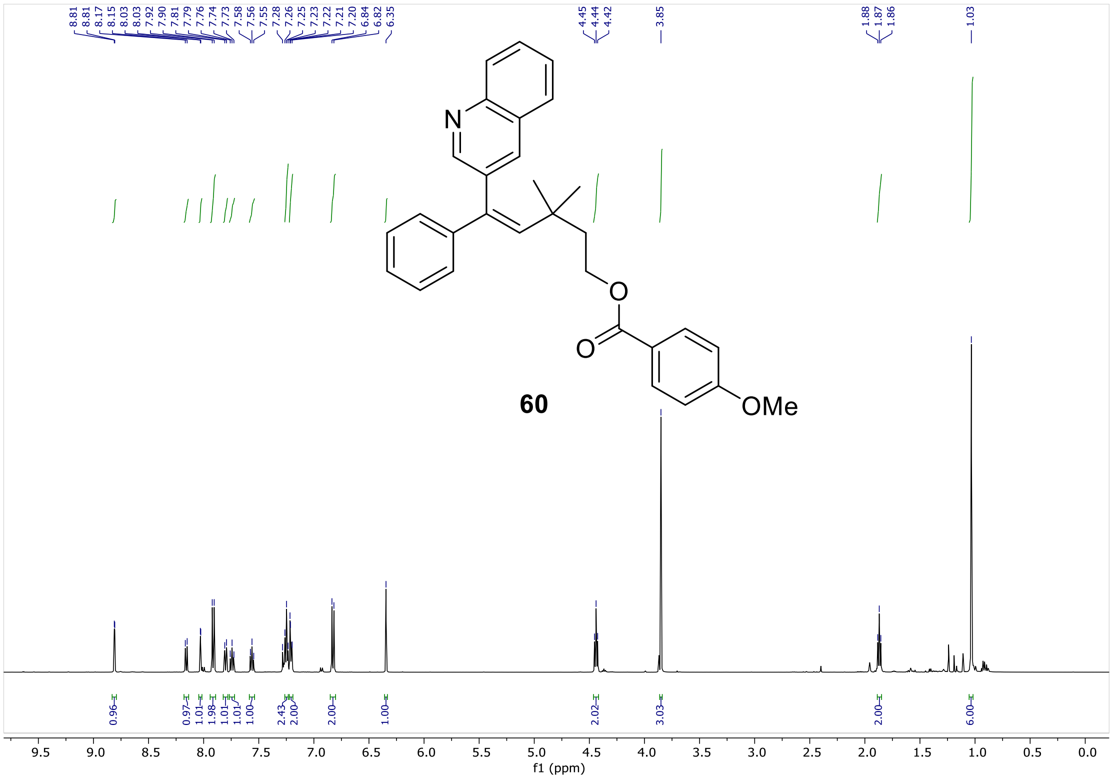

Supplementary Figure 152: ^{1}H NMR spectrum of compound **60**

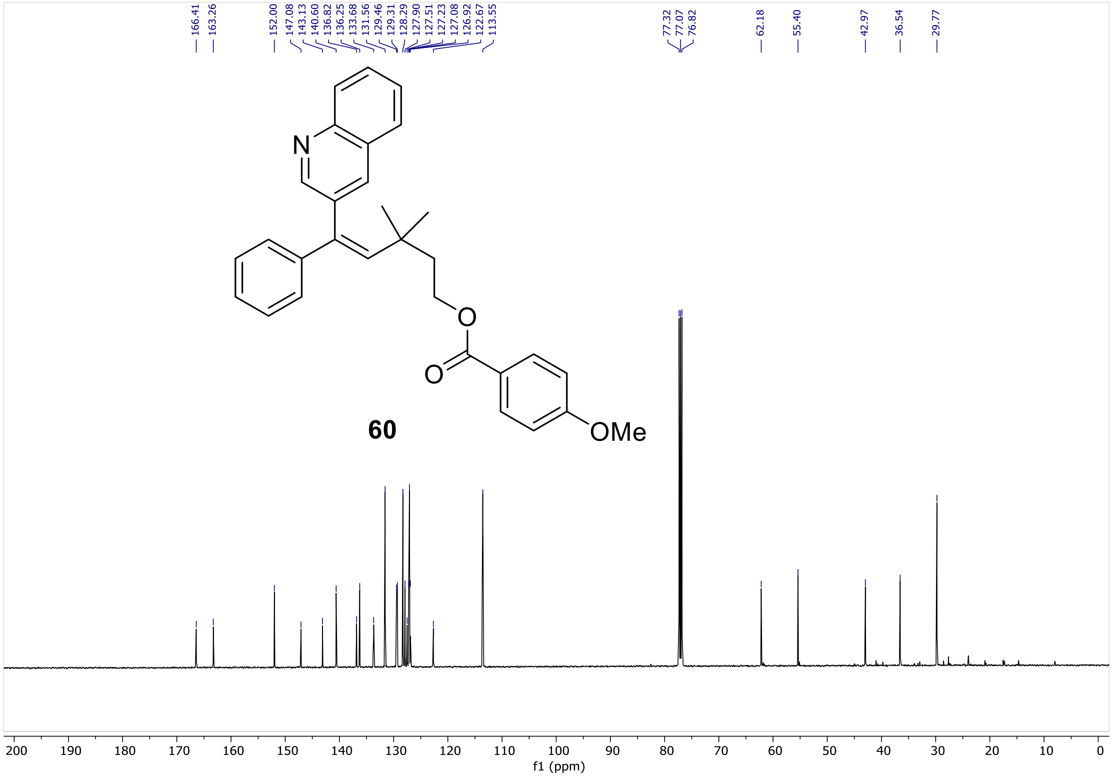

Supplementary Figure 153: ^{13}C NMR spectrum of compound **60**

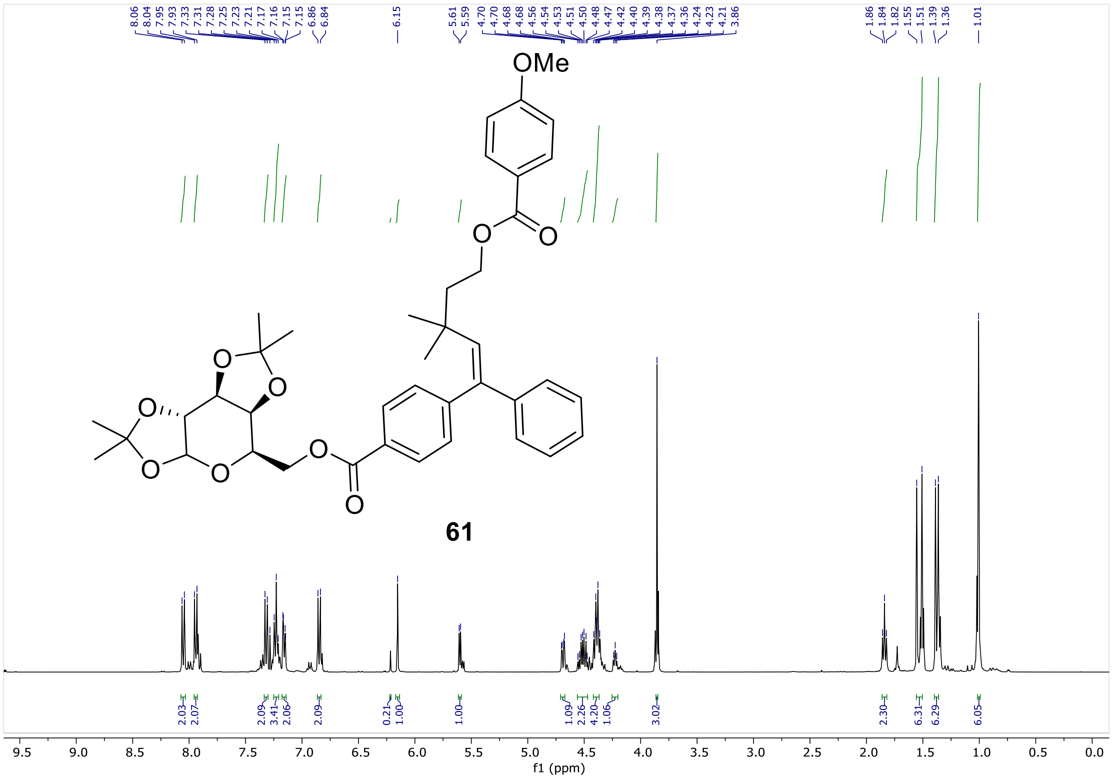

Supplementary Figure 154: ^{1}H NMR spectrum of compound **61**

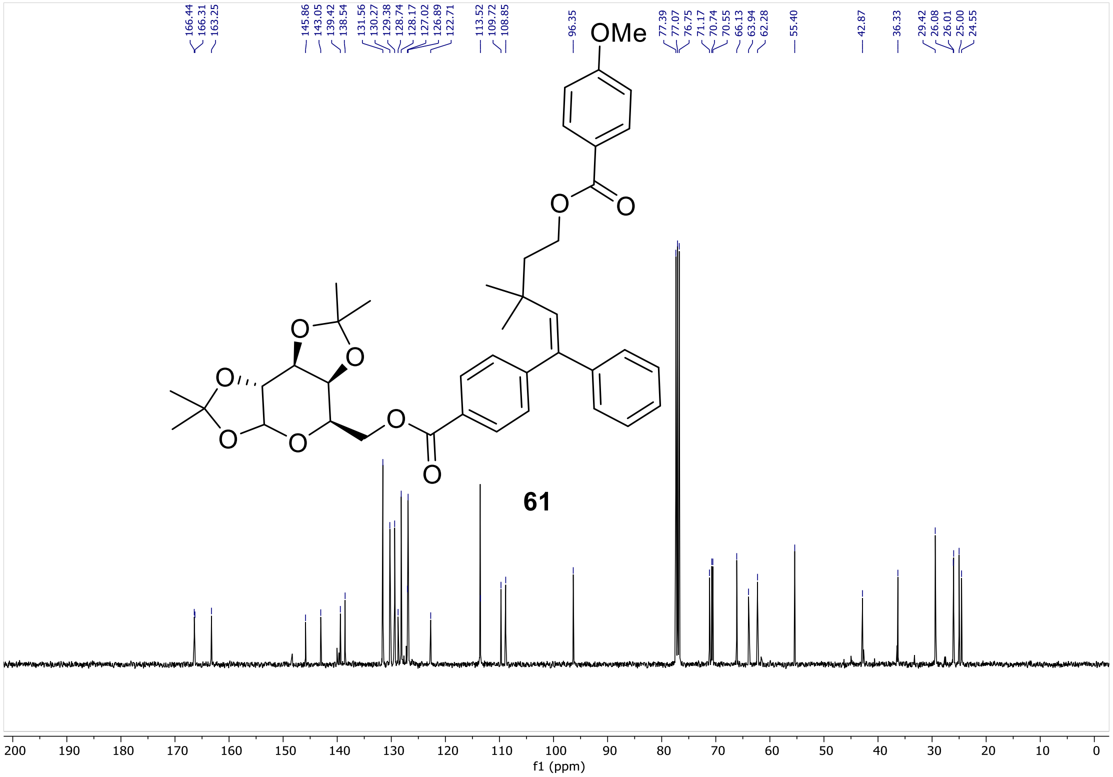

Supplementary Figure 155: ^{13}C NMR spectrum of compound **61**

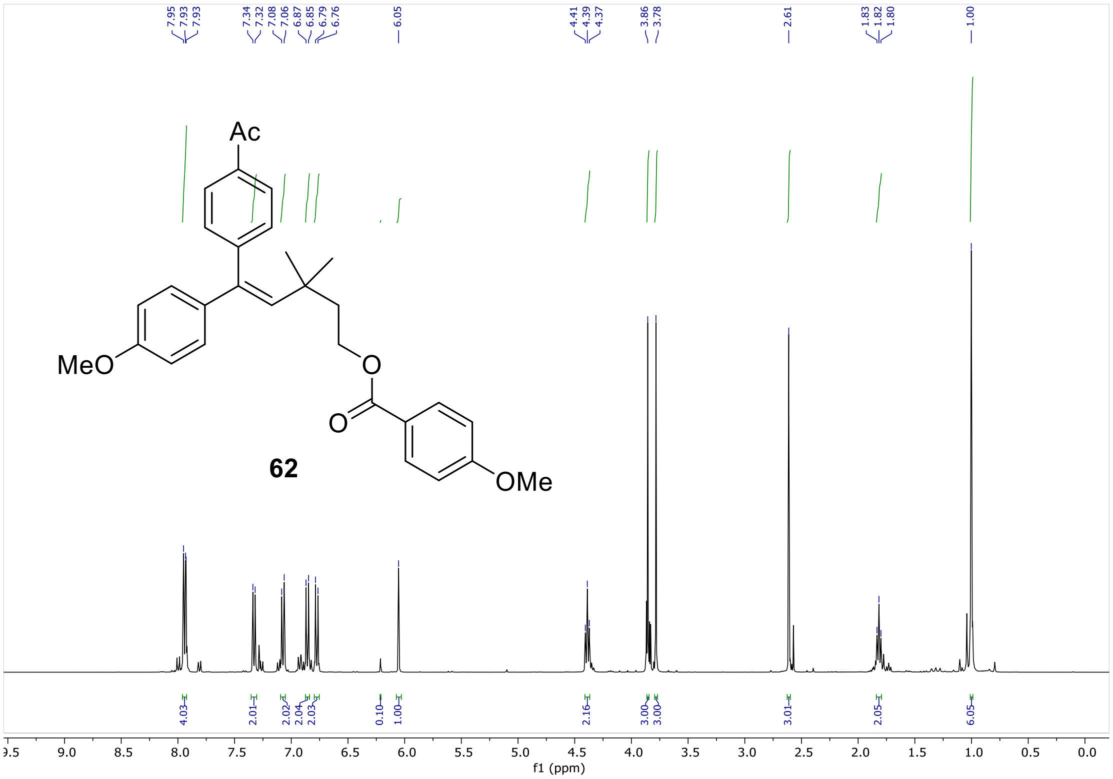

Supplementary Figure 156: ^{1}H NMR spectrum of compound **62**

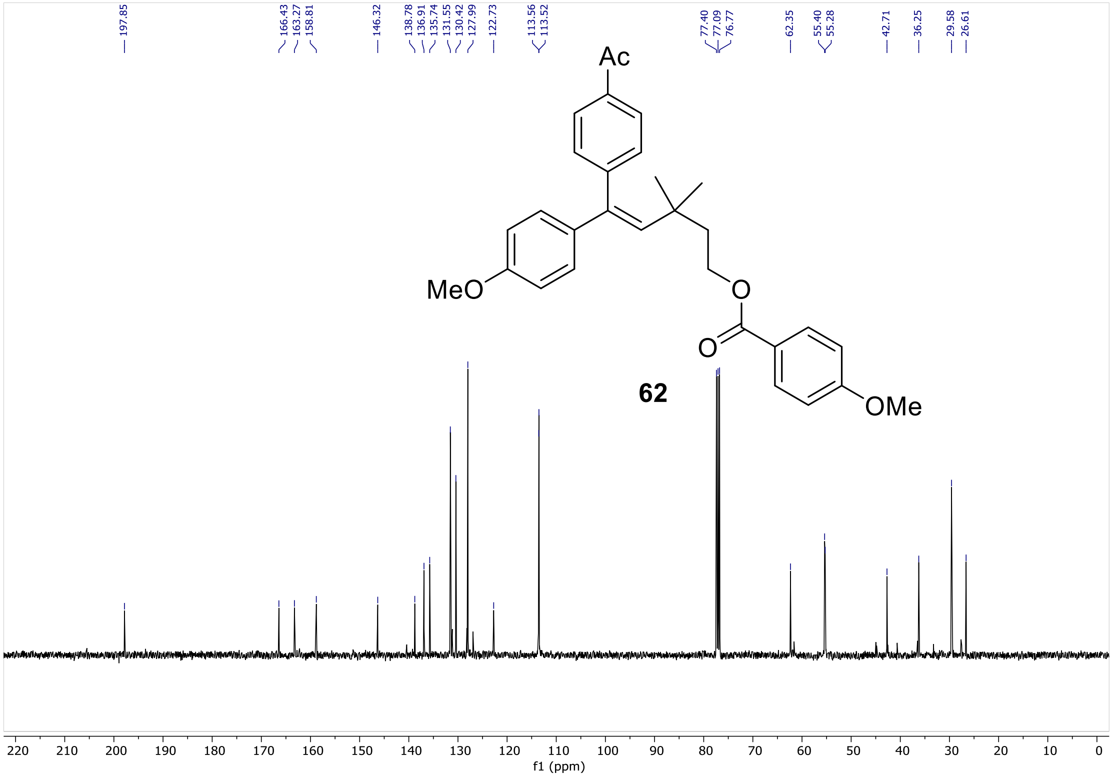

Supplementary Figure 157: ^{13}C NMR spectrum of compound **62**

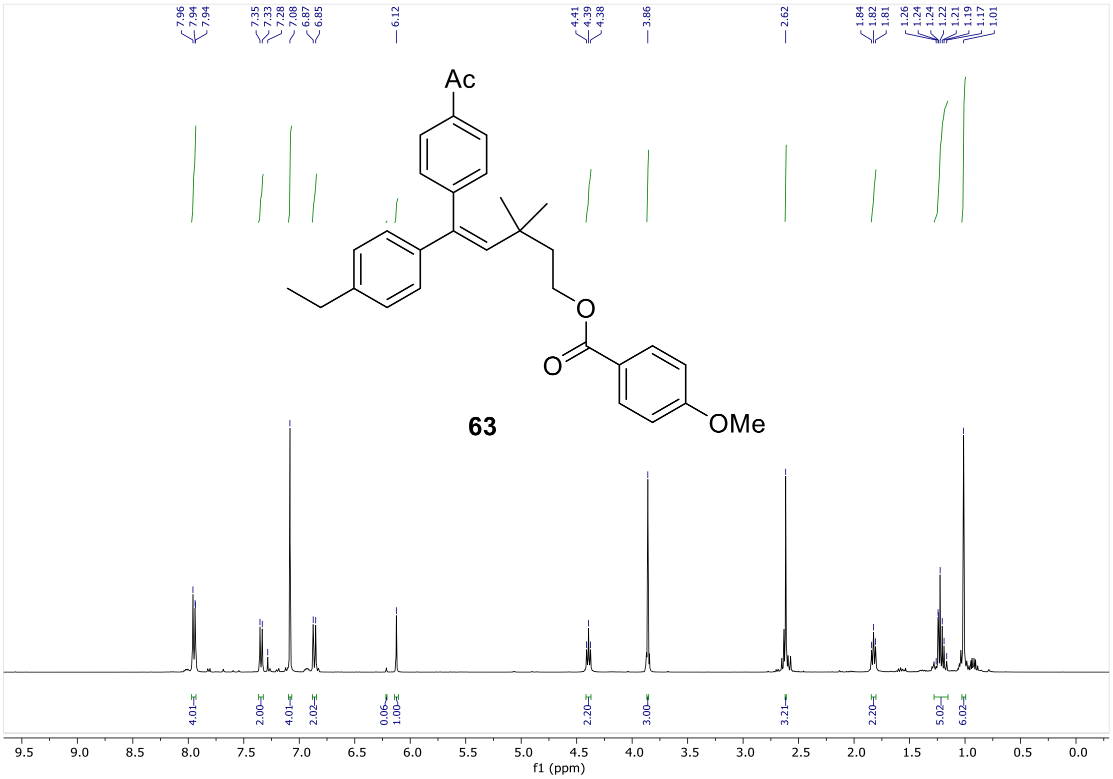

Supplementary Figure 158: ^{1}H NMR spectrum of compound **63**

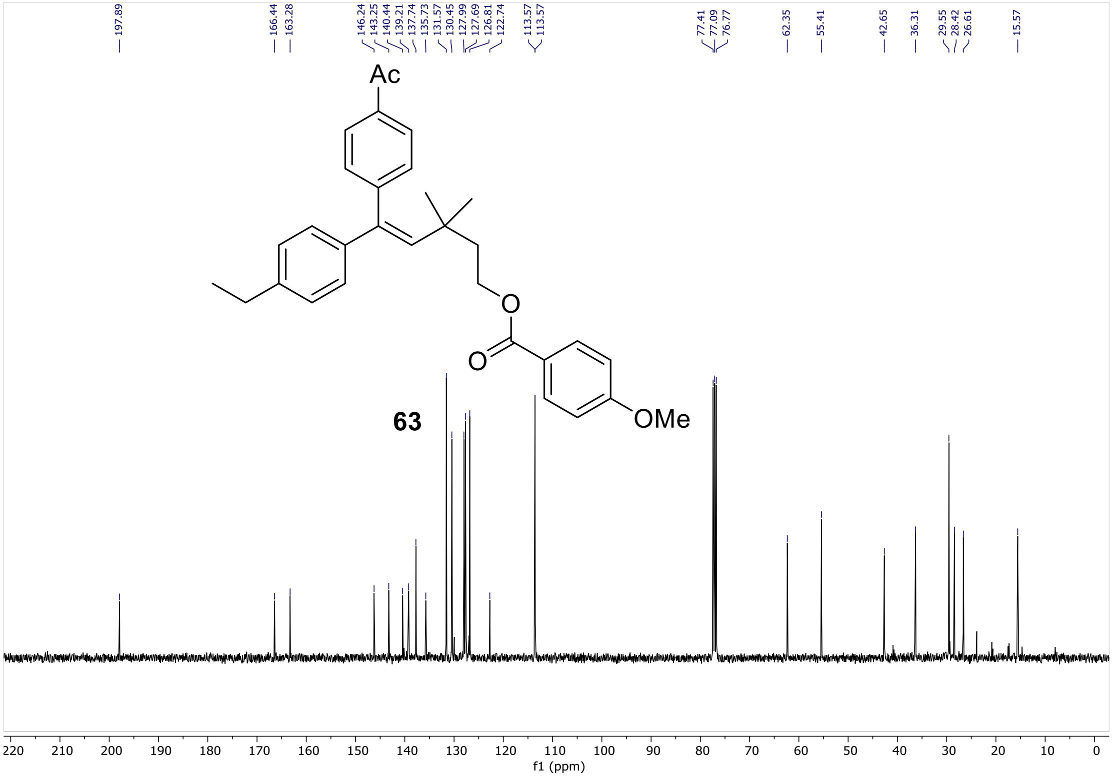

Supplementary Figure 159: ^{13}C NMR spectrum of compound **63**

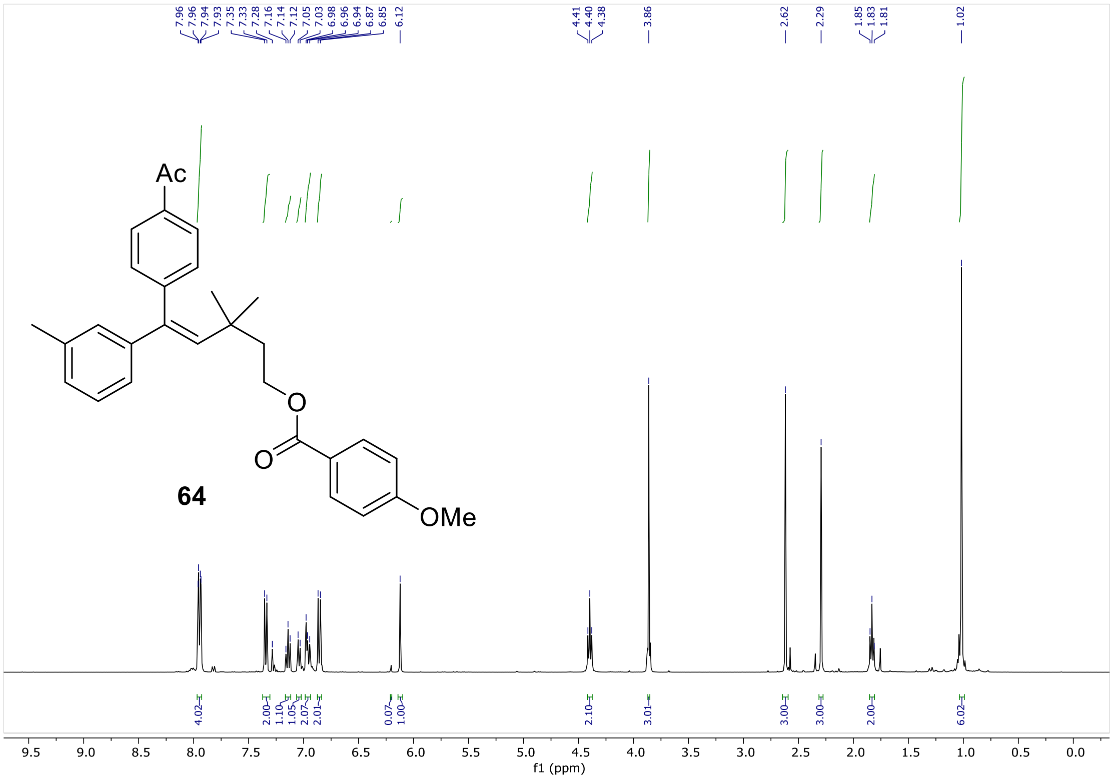

Supplementary Figure 160: ^{1}H NMR spectrum of compound **64**

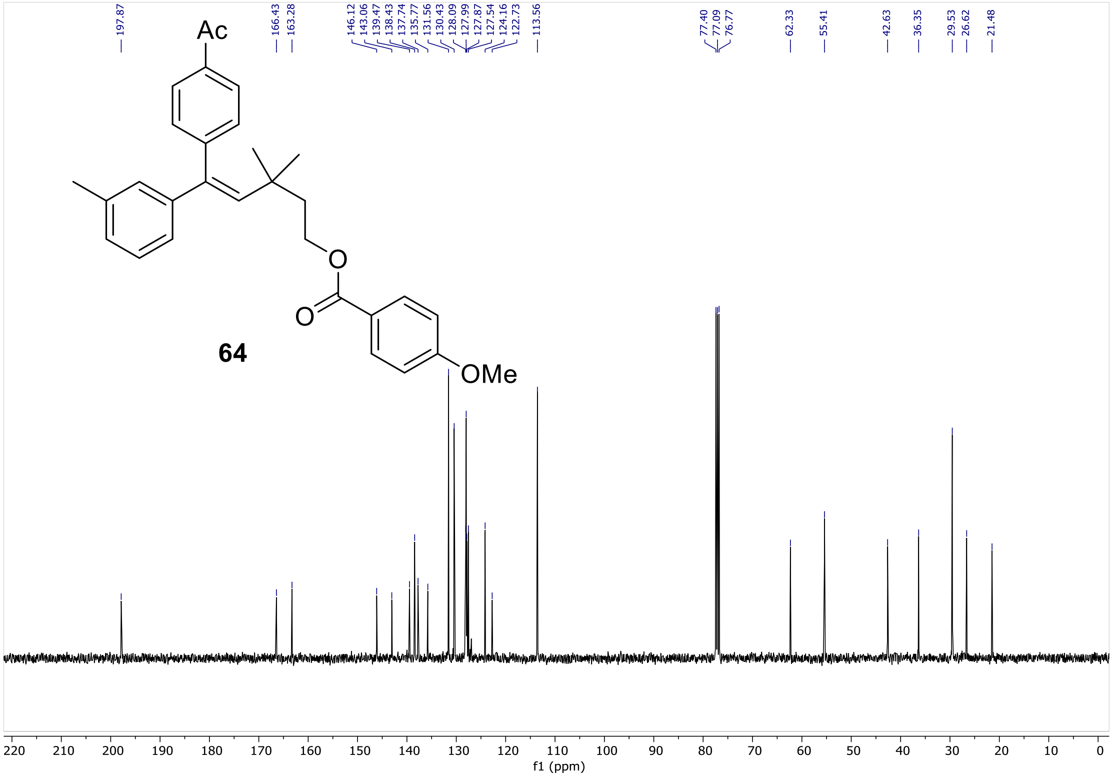

Supplementary Figure 161: ^{13}C NMR spectrum of compound **64**

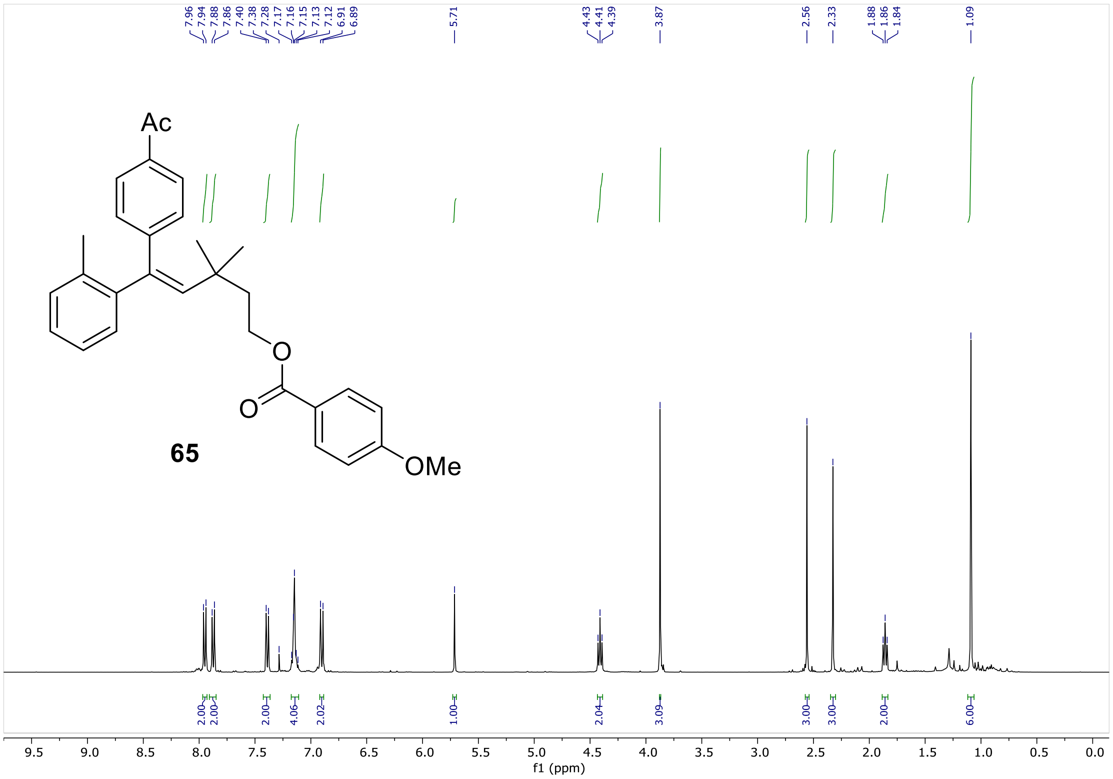

Supplementary Figure 162: ^{1}H NMR spectrum of compound **65**

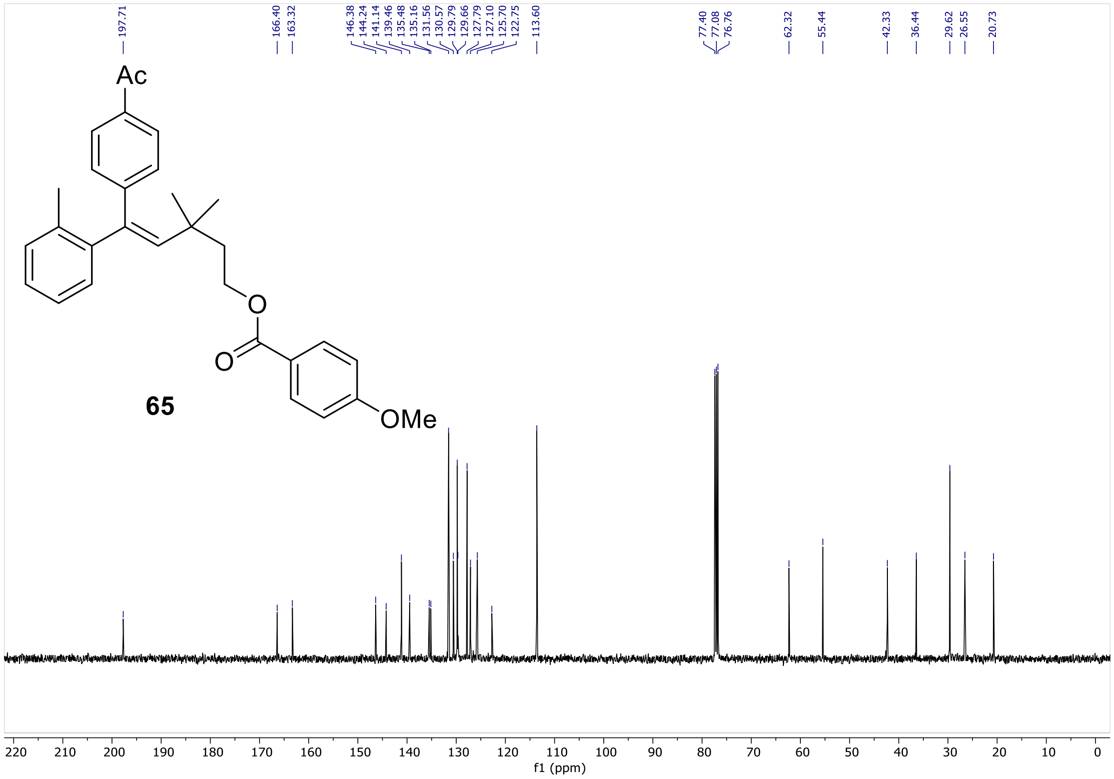

Supplementary Figure 163: ^{13}C NMR spectrum of compound **65**

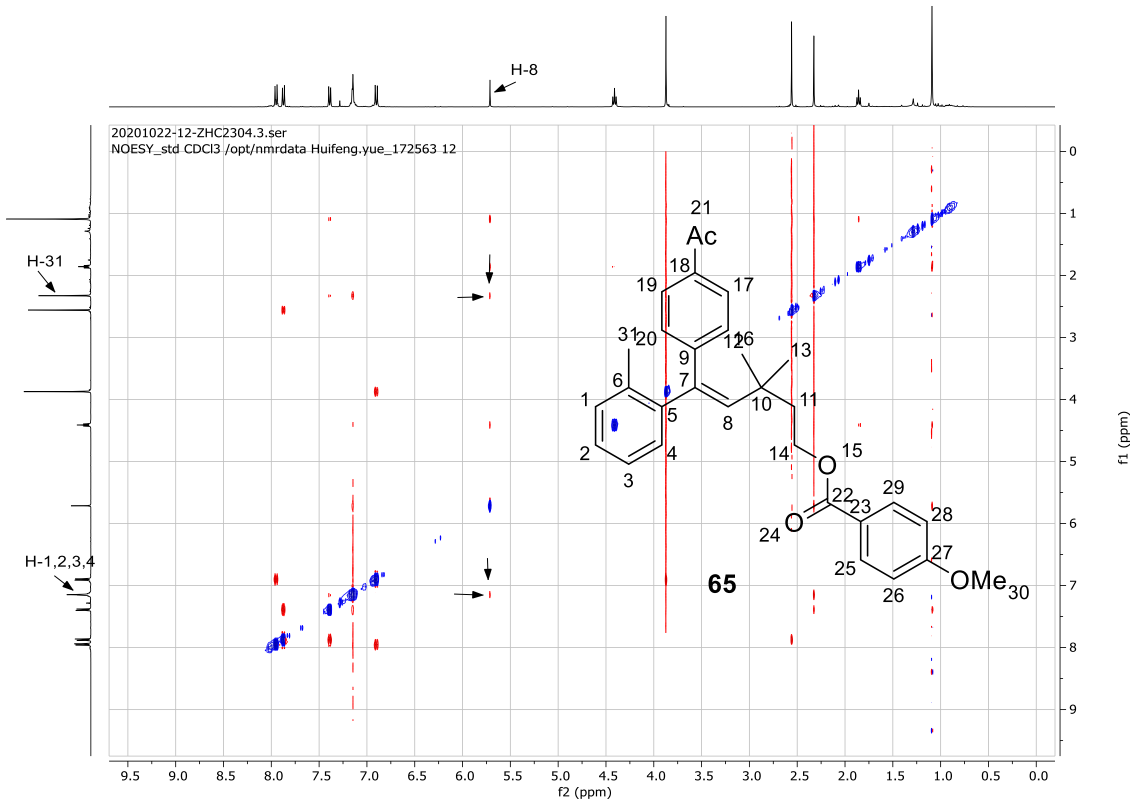

Supplementary Figure 164: NOE spectrum of compound **65**

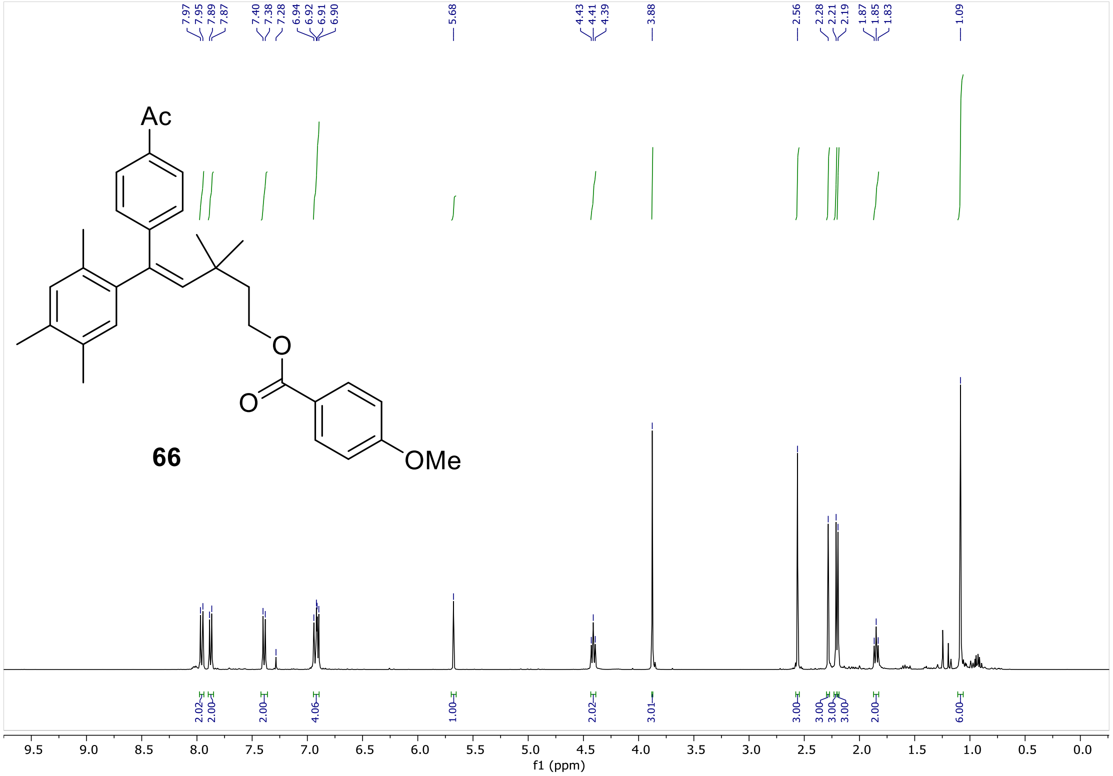

Supplementary Figure 165: ^{1}H NMR spectrum of compound **66**

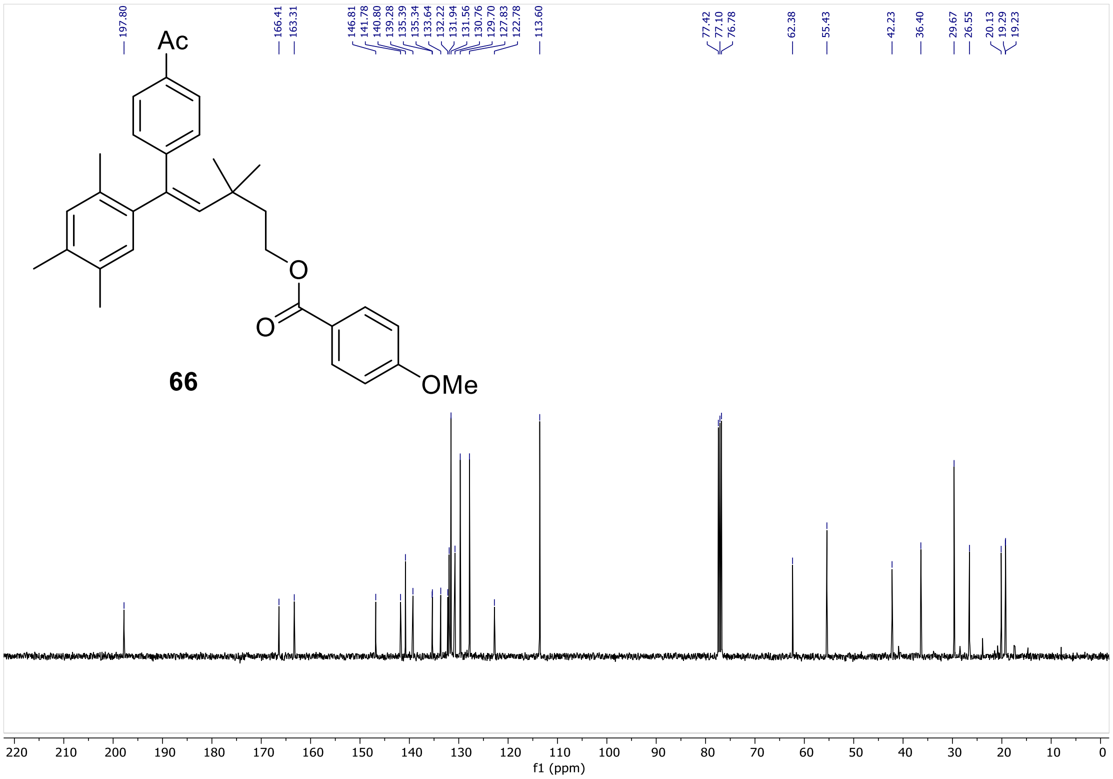

Supplementary Figure 166: ^{13}C NMR spectrum of compound **66**

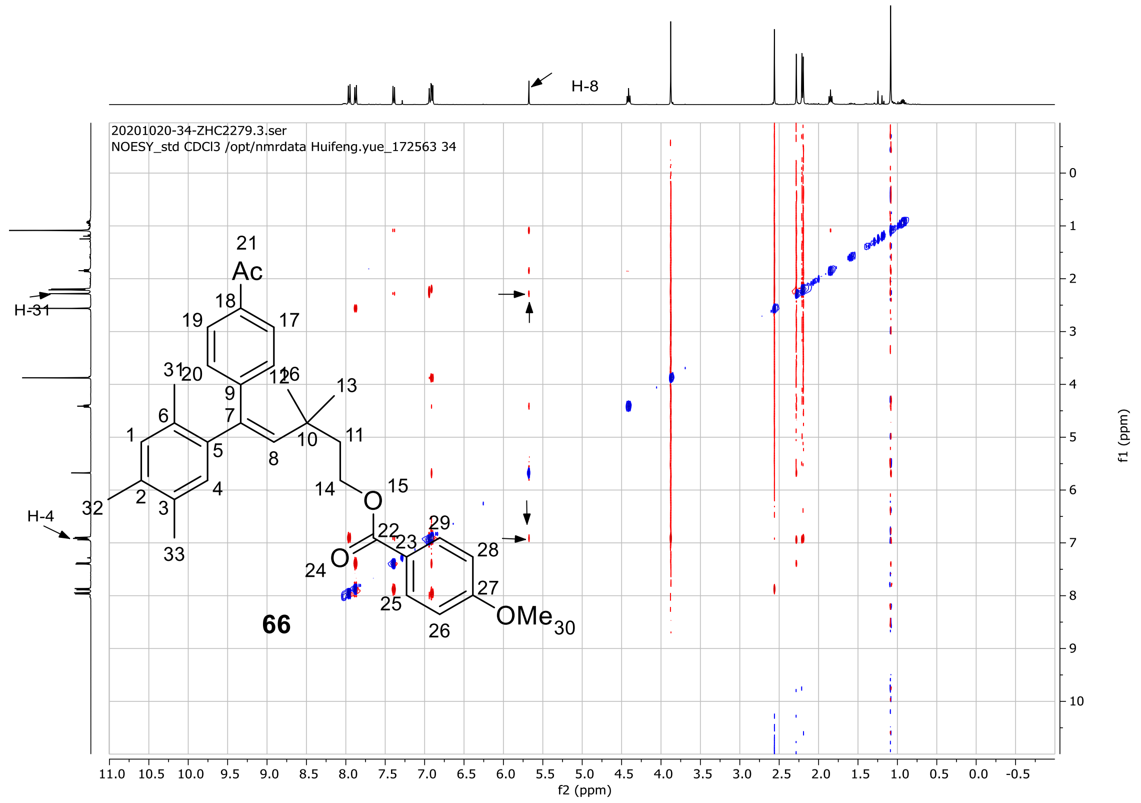

Supplementary Figure 167: NOE spectrum of compound **66**

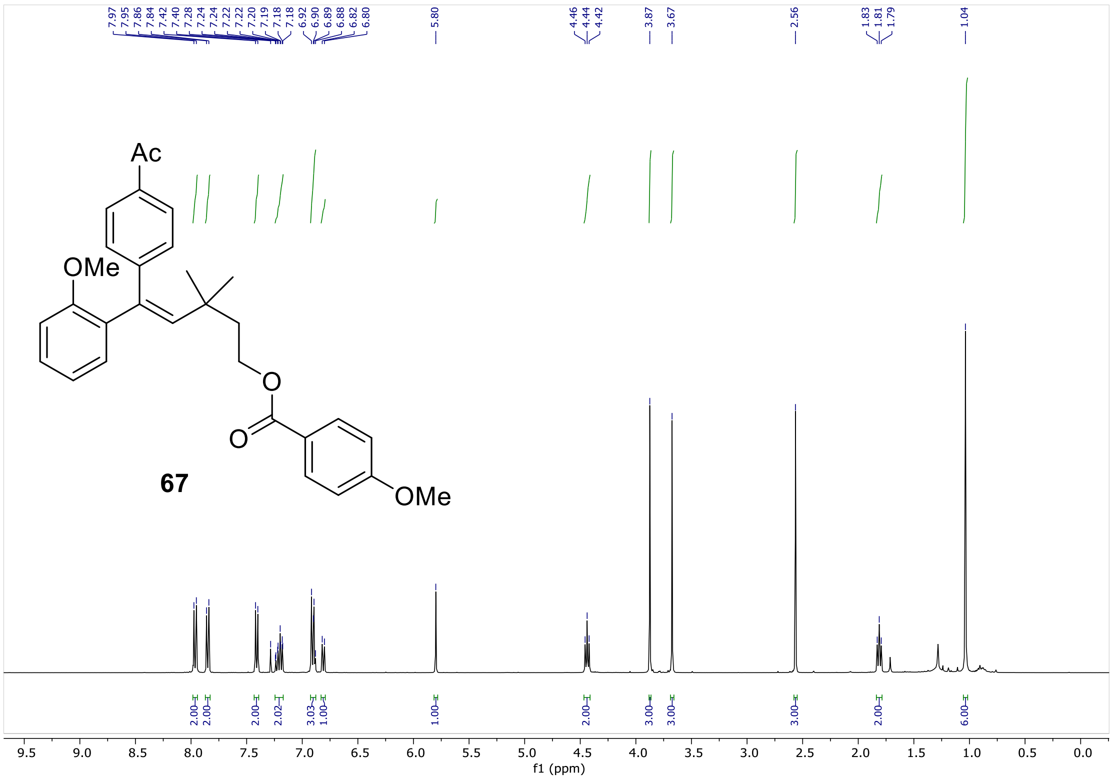

Supplementary Figure 168: ^{1}H NMR spectrum of compound **67**

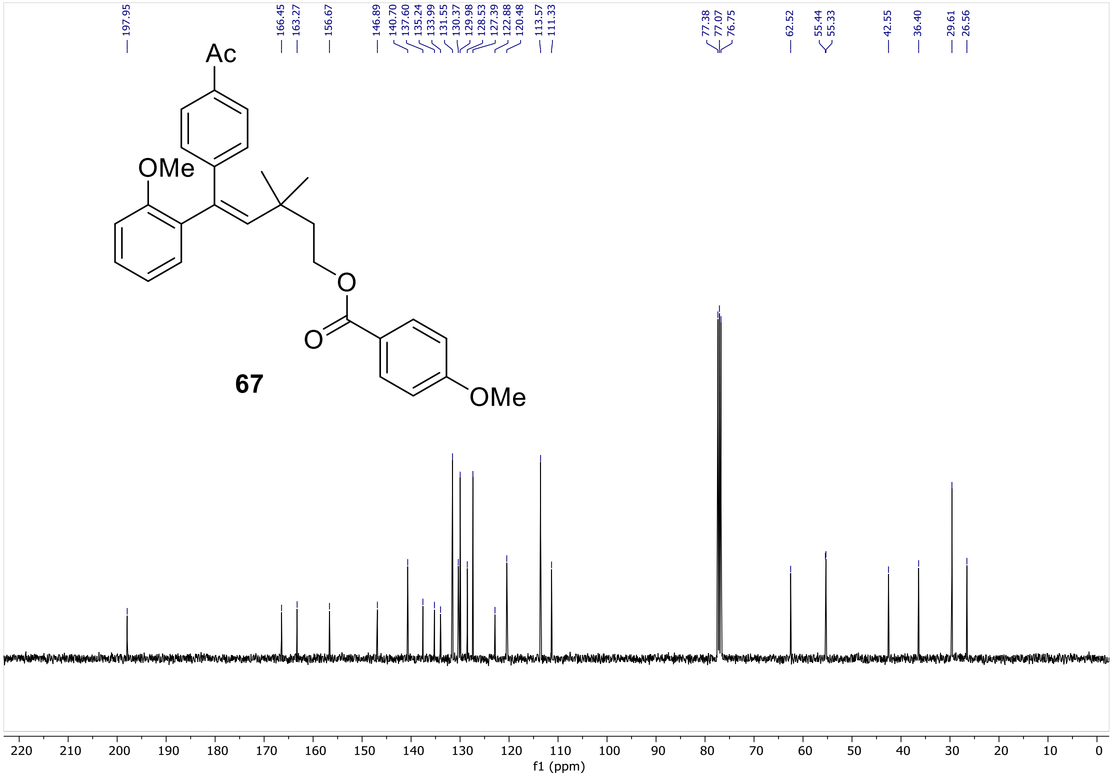

Supplementary Figure 169: ^{13}C NMR spectrum of compound **67**

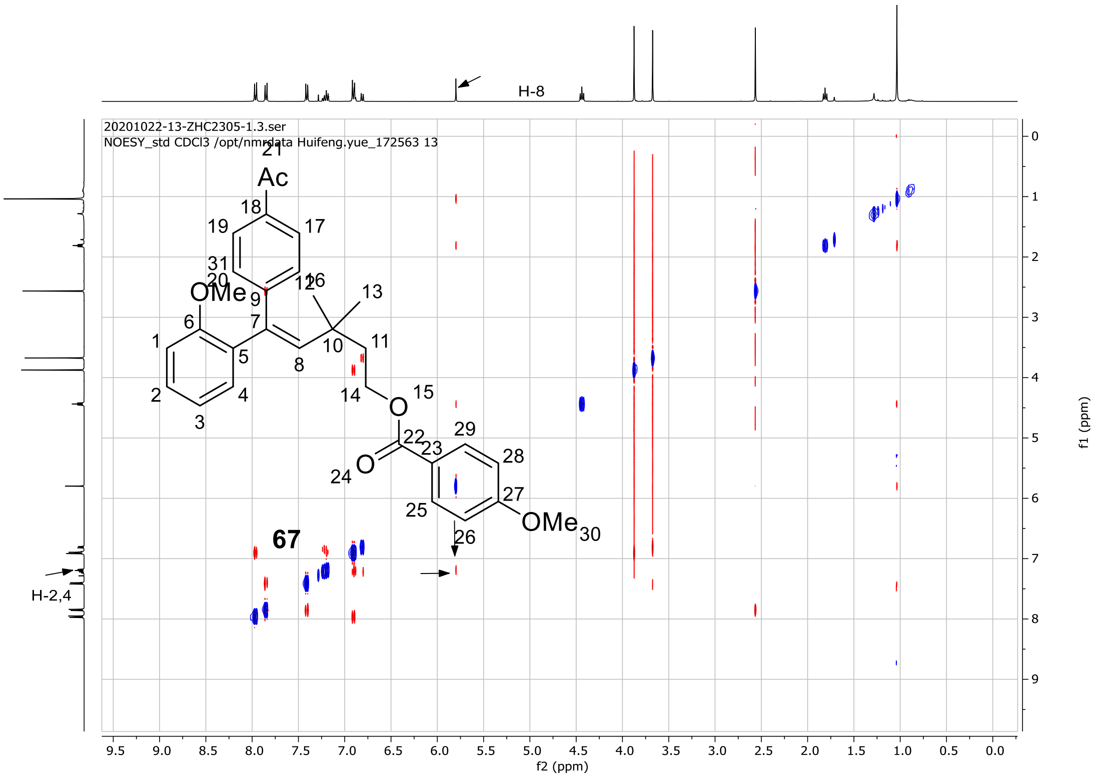

Supplementary Figure 170: NOE spectrum of compound **67**

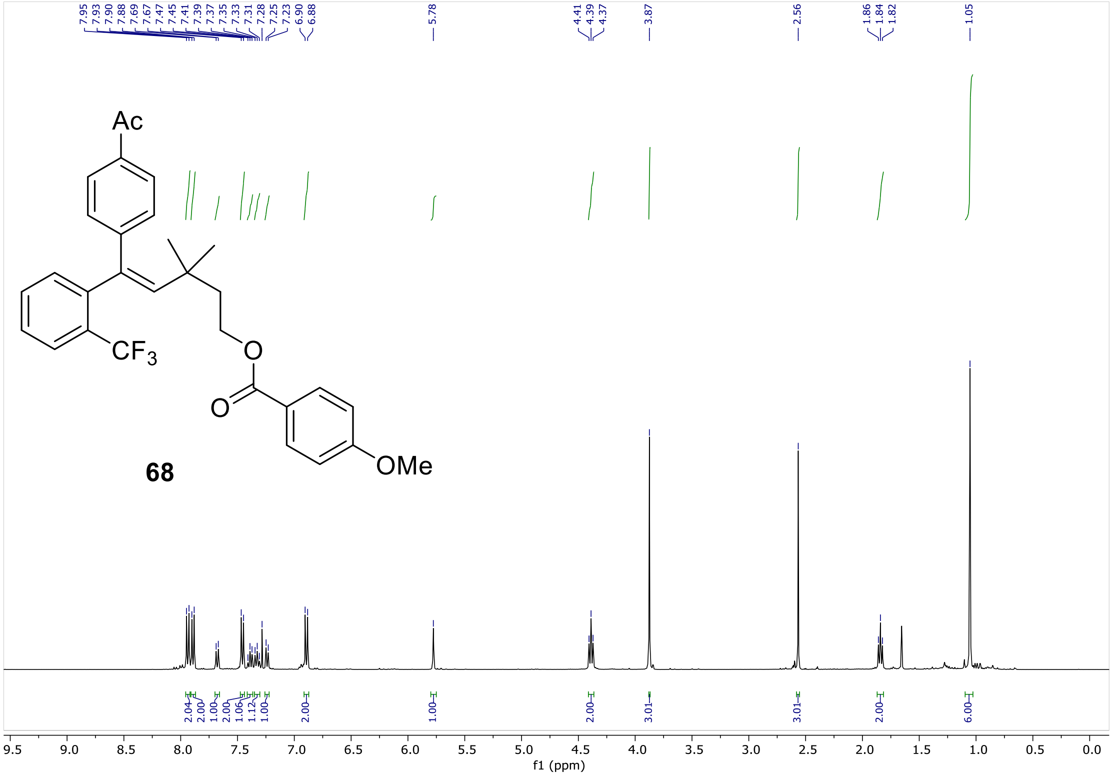

Supplementary Figure 171: ^{1}H NMR spectrum of compound **68**

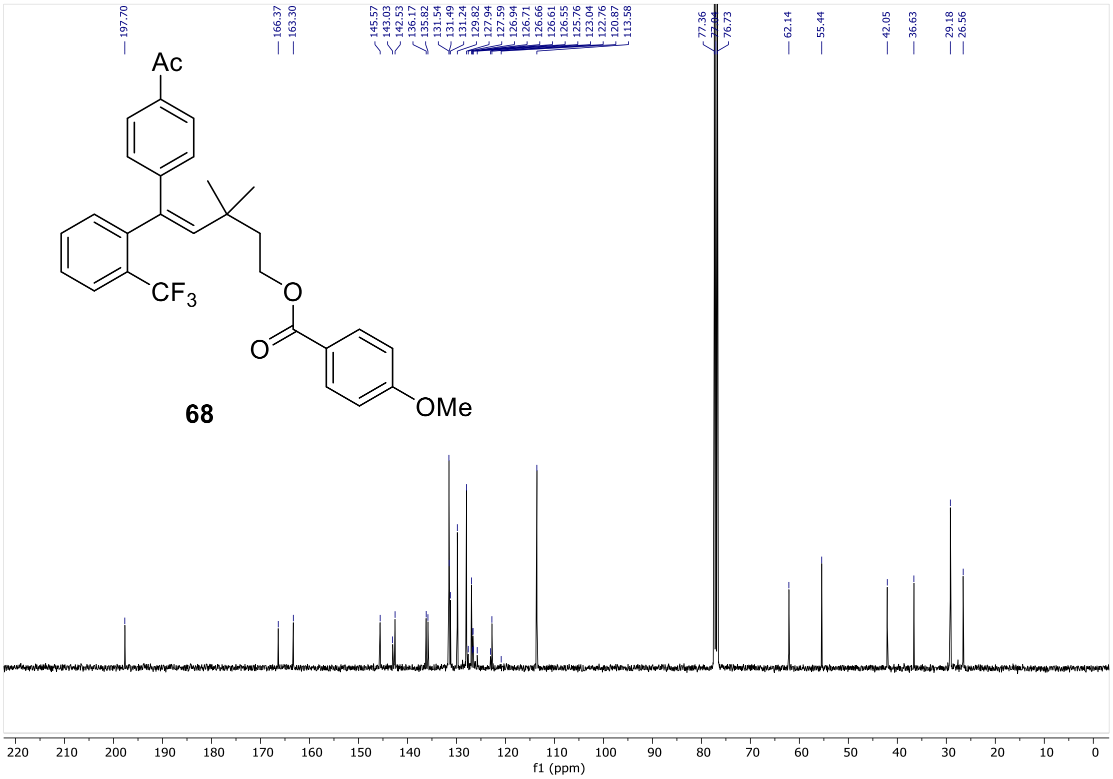

Supplementary Figure 172: ^{13}C NMR spectrum of compound **68**

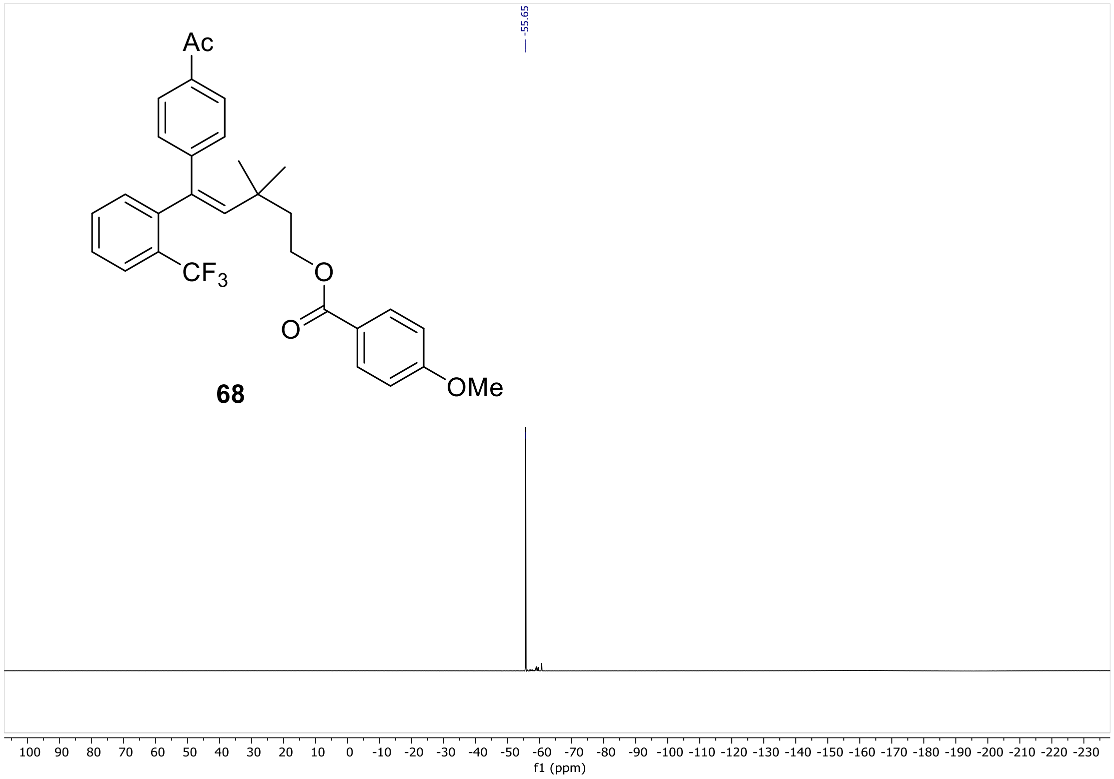

Supplementary Figure 173: ^{19}F NMR spectrum of compound **68**

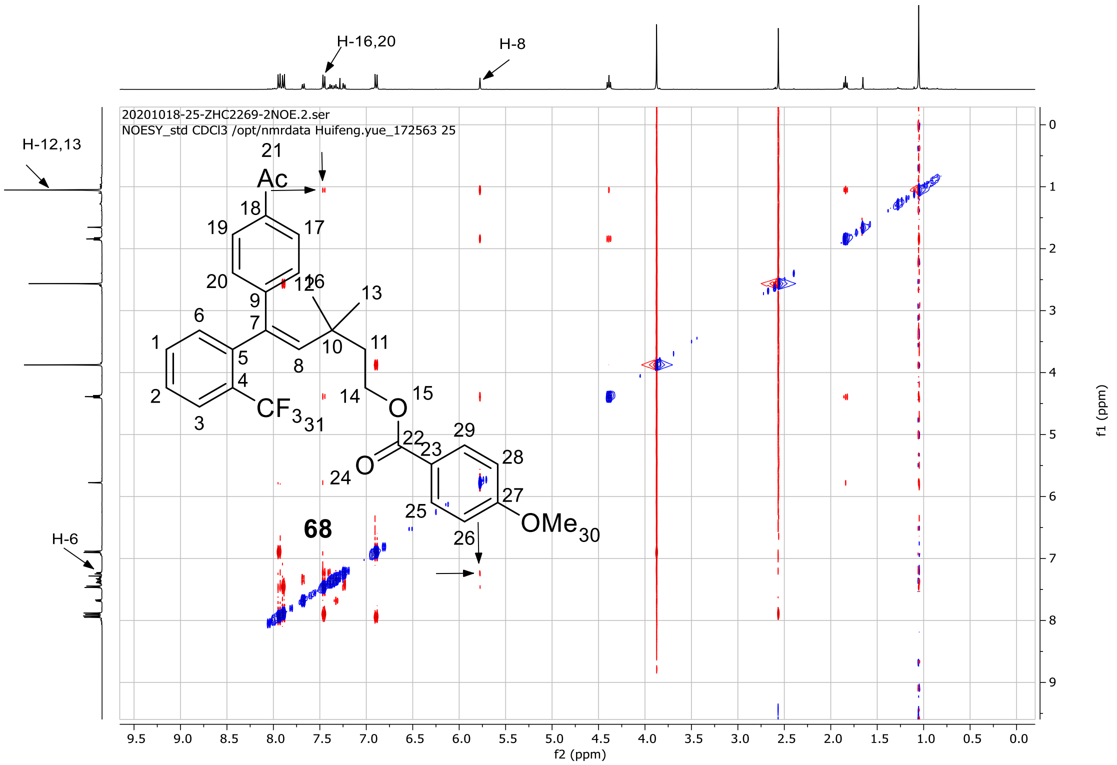

Supplementary Figure 174: NOE spectrum of compound **68**

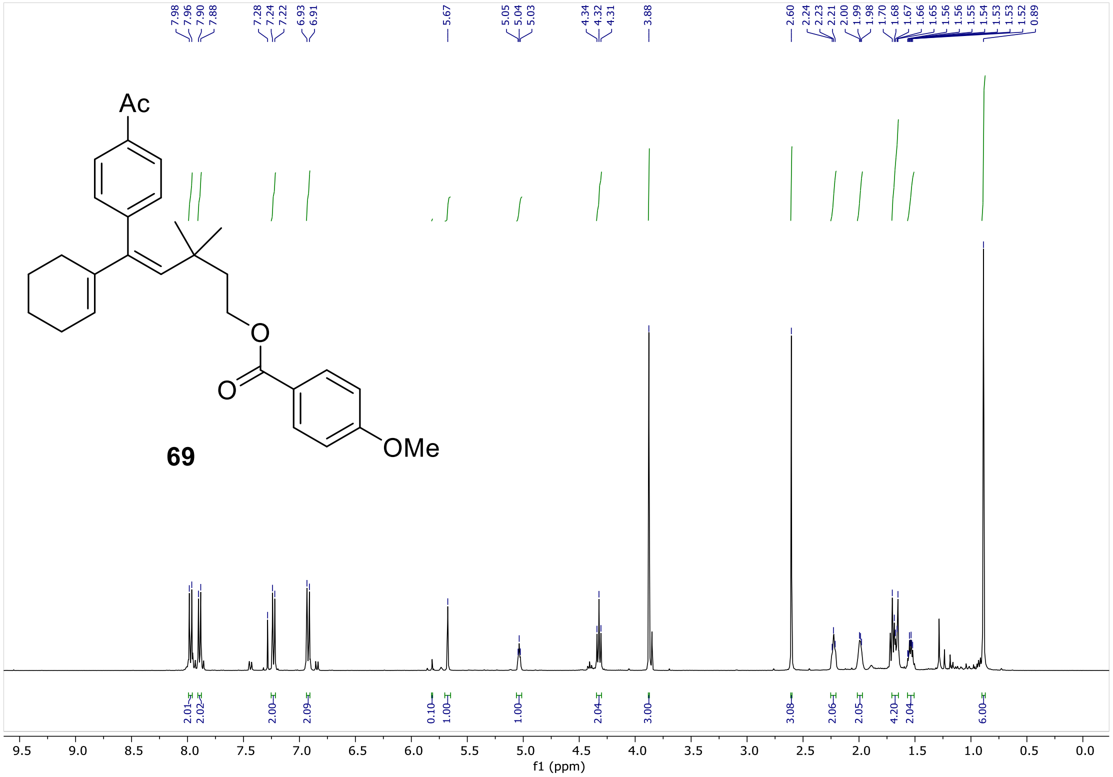

Supplementary Figure 175: ^{1}H NMR spectrum of compound **69**

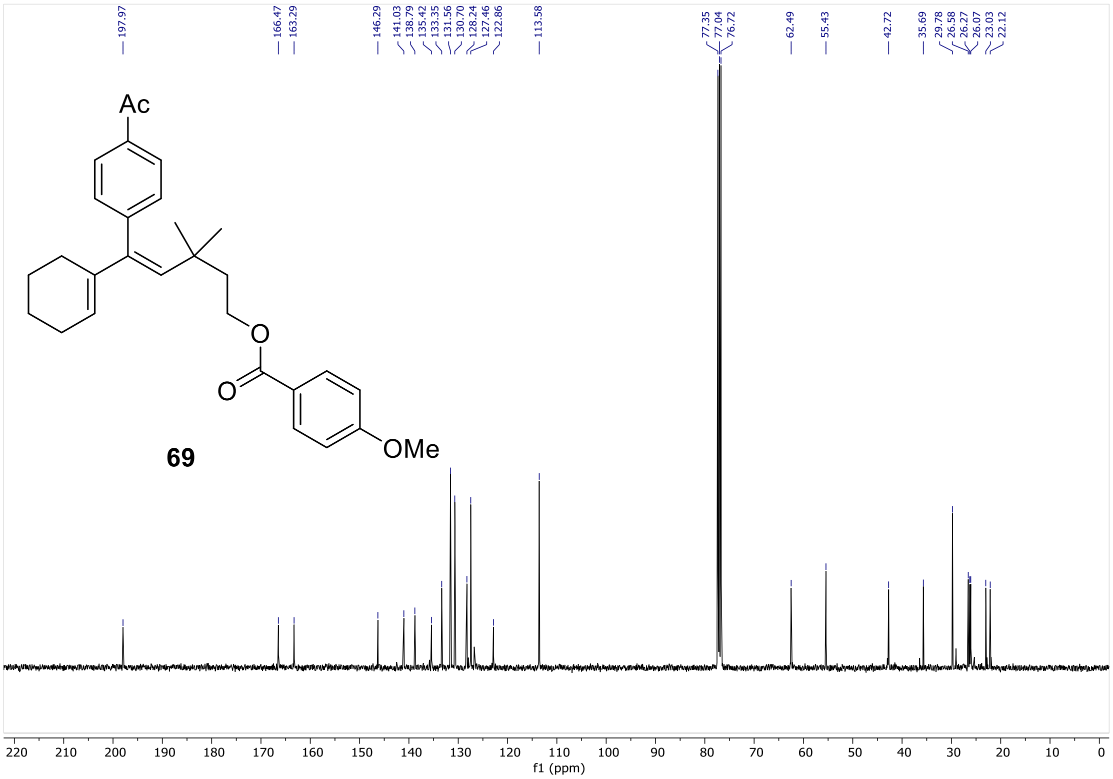

Supplementary Figure 176: ^{13}C NMR spectrum of compound **69**

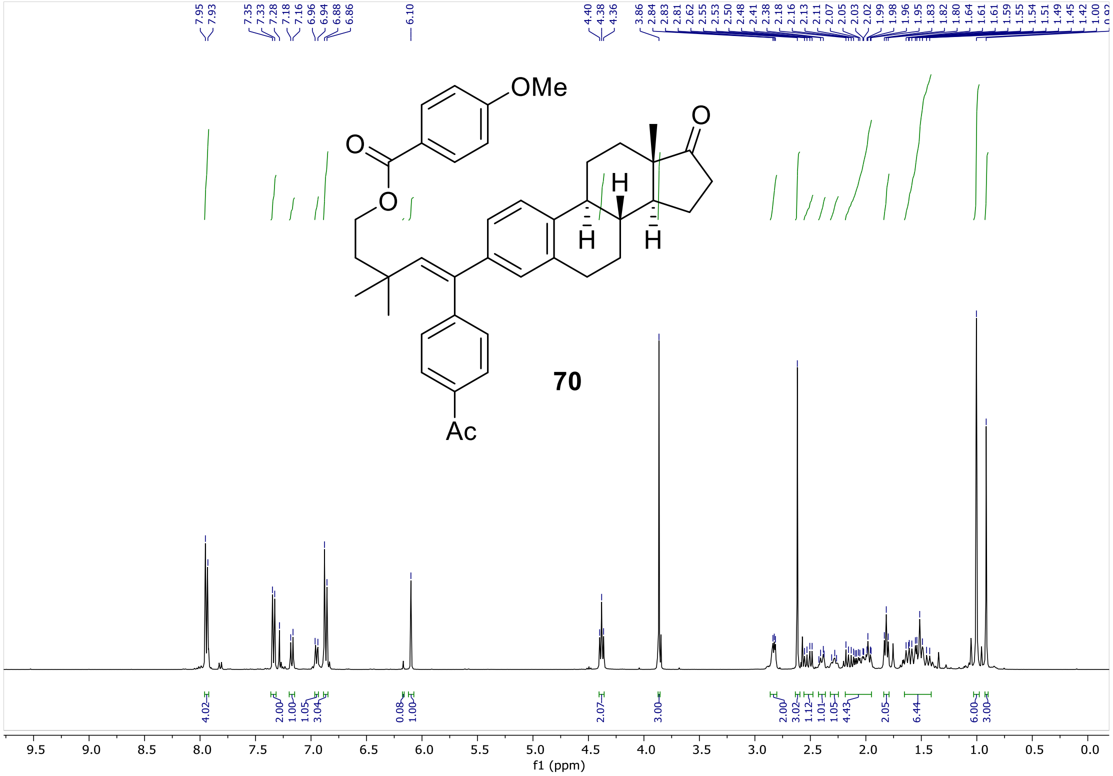

Supplementary Figure 177: ^{1}H NMR spectrum of compound **70**

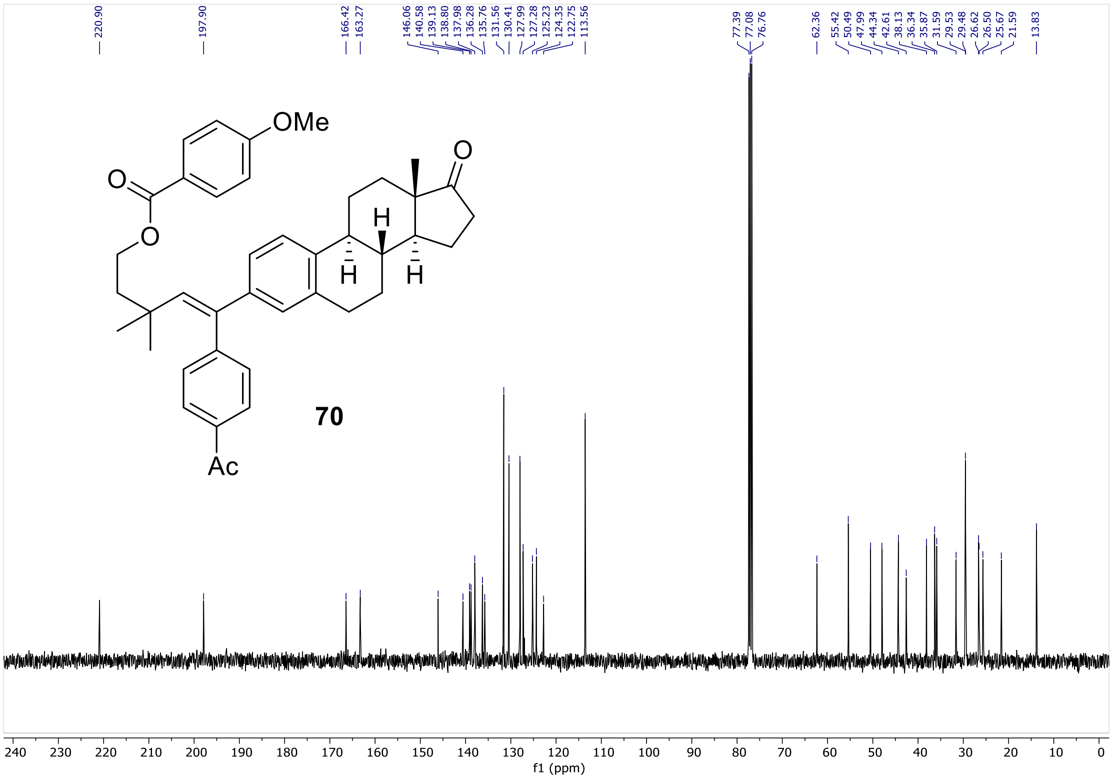

Supplementary Figure 178: ^{13}C NMR spectrum of compound **70**

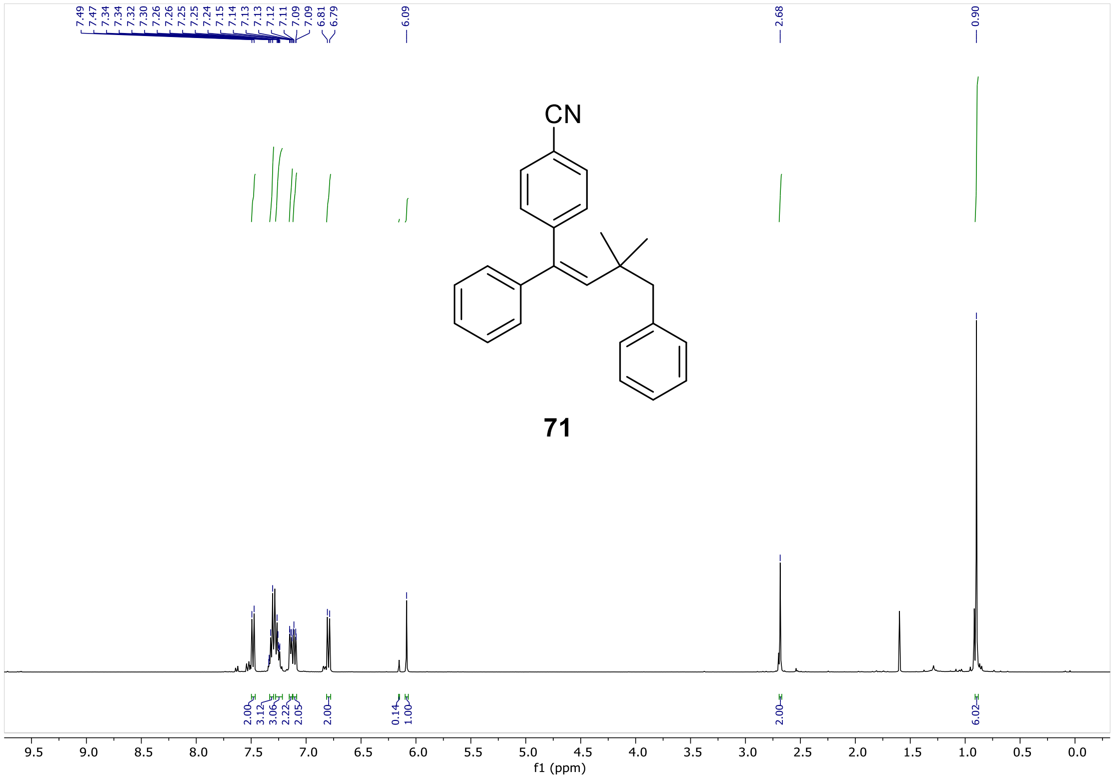

Supplementary Figure 179: ^{1}H NMR spectrum of compound **71**

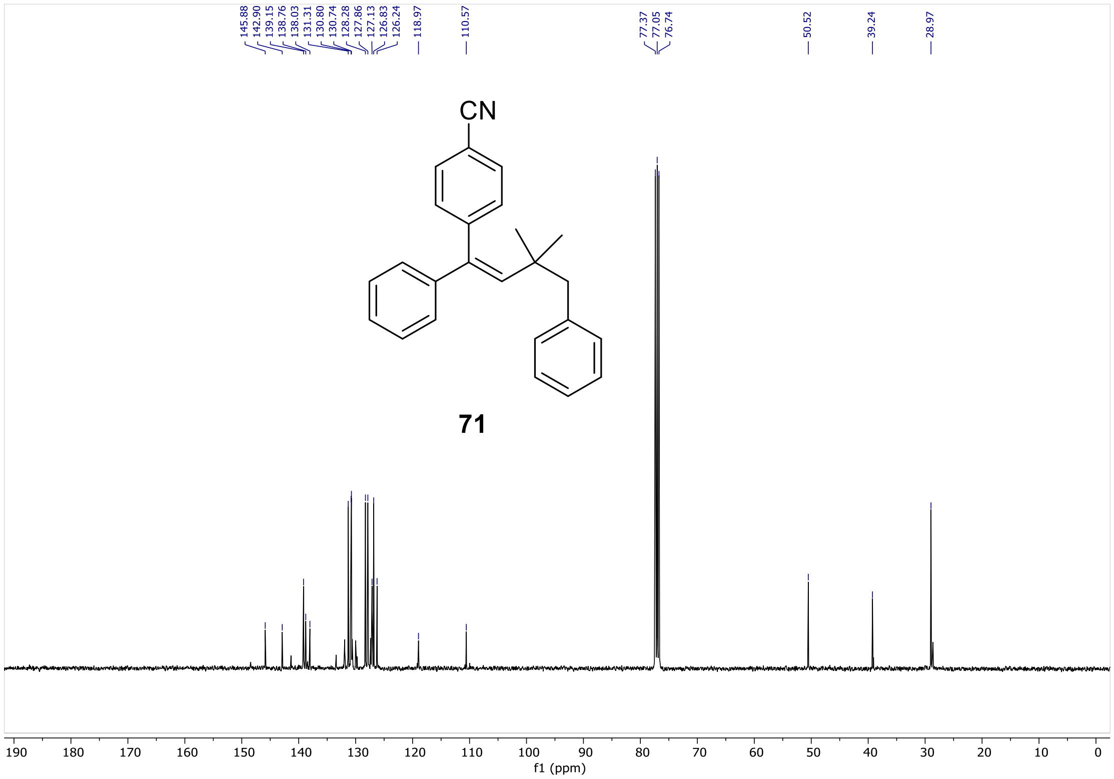

Supplementary Figure 180: ^{13}C NMR spectrum of compound **71**

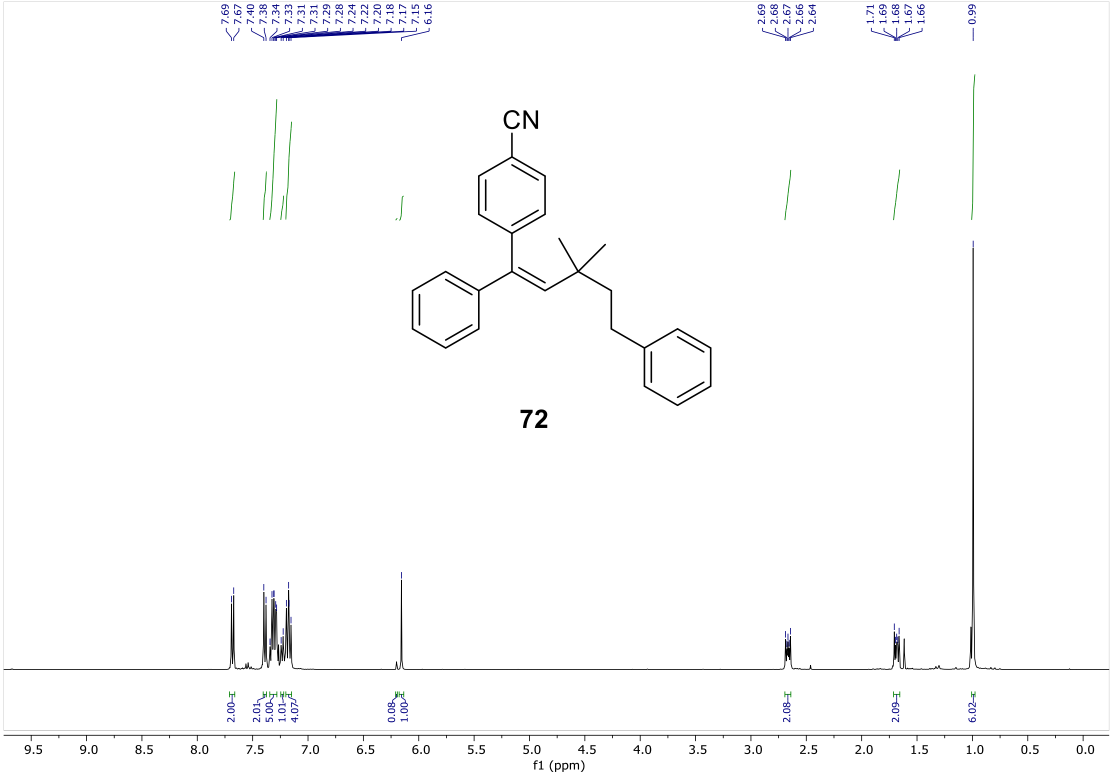

Supplementary Figure 181: ^{1}H NMR spectrum of compound **72**

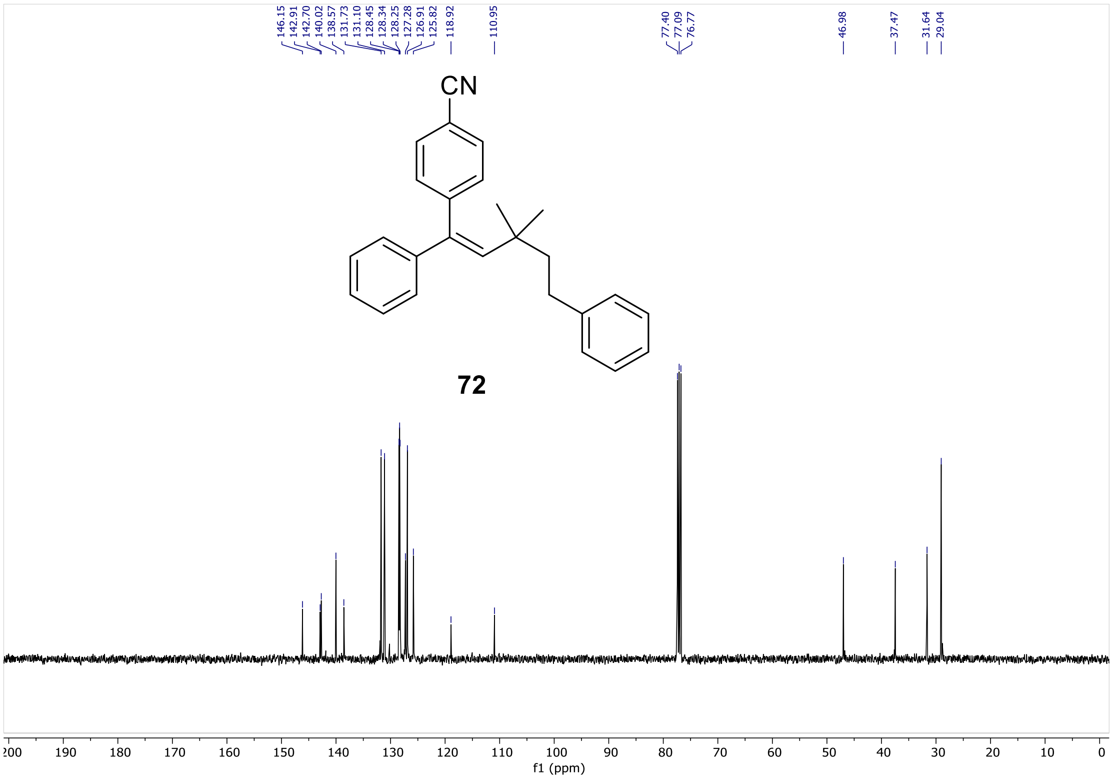

Supplementary Figure 182: ^{13}C NMR spectrum of compound **72**

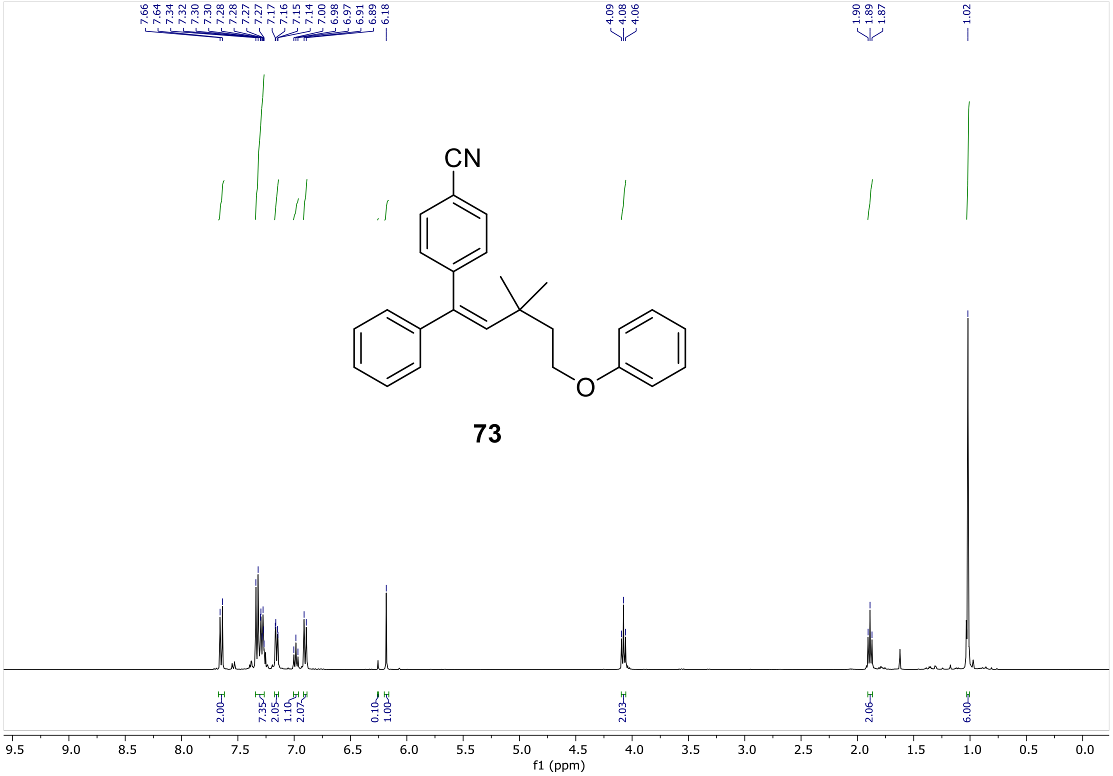

Supplementary Figure 183: ^{1}H NMR spectrum of compound **73**

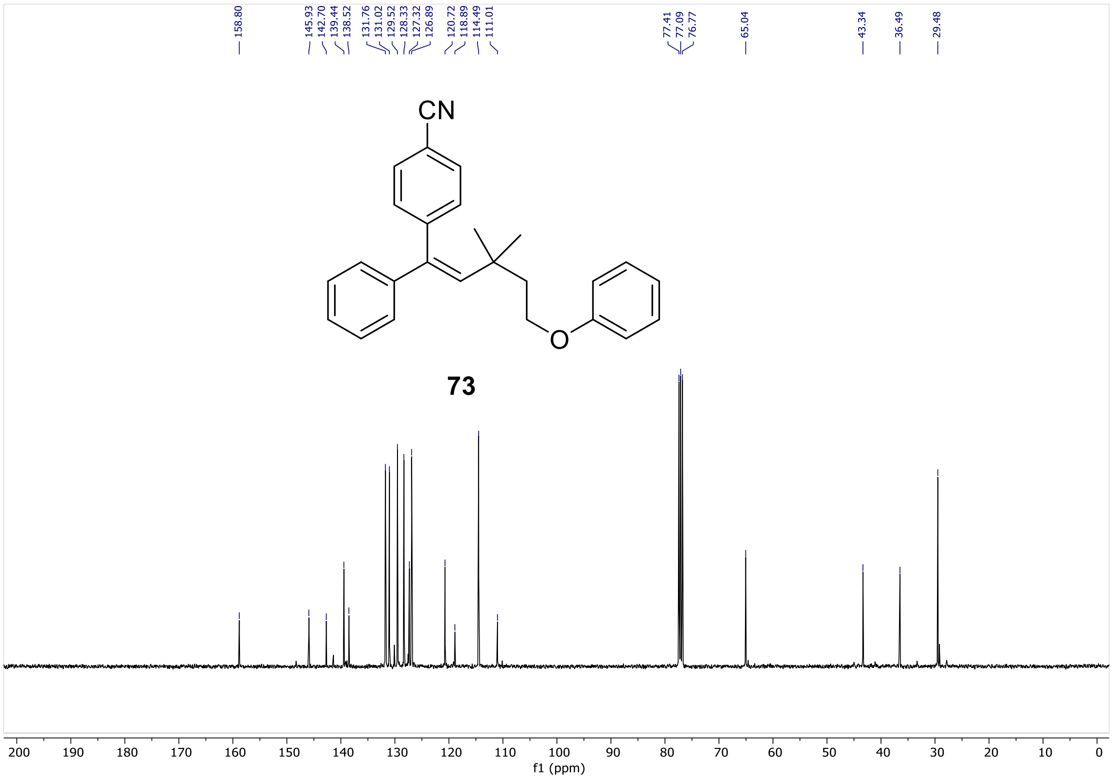

Supplementary Figure 184: ^{13}C NMR spectrum of compound **73**

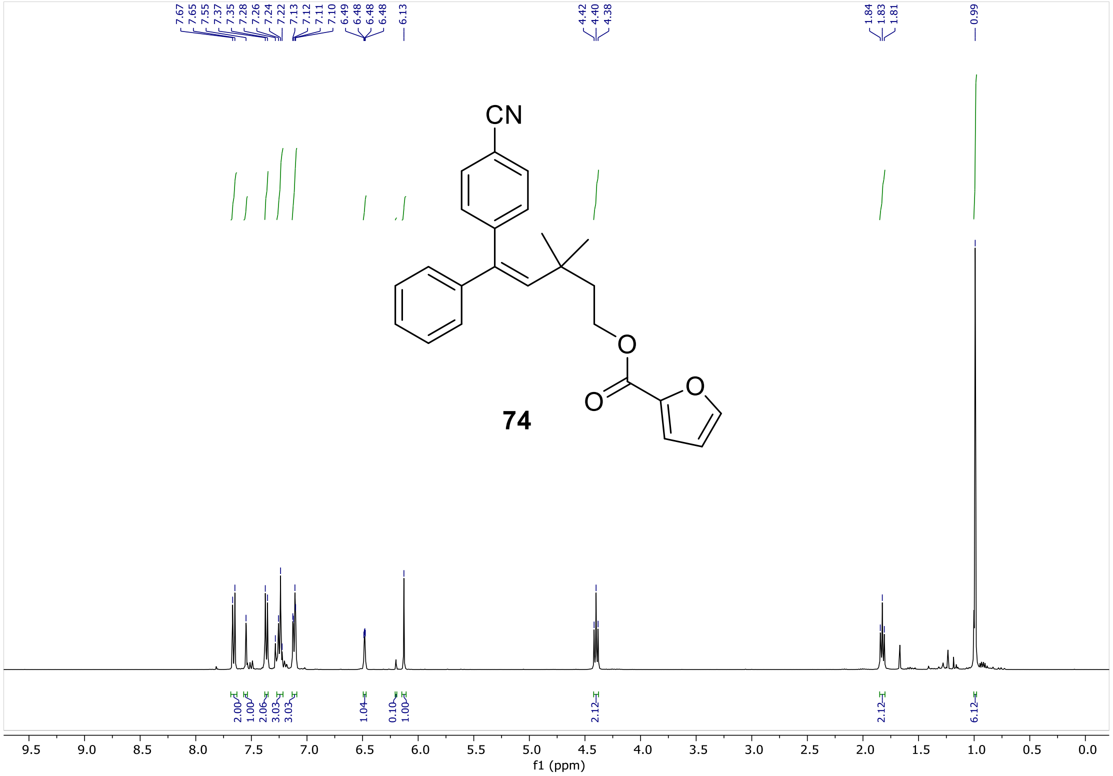

Supplementary Figure 185: ^{1}H NMR spectrum of compound **74**

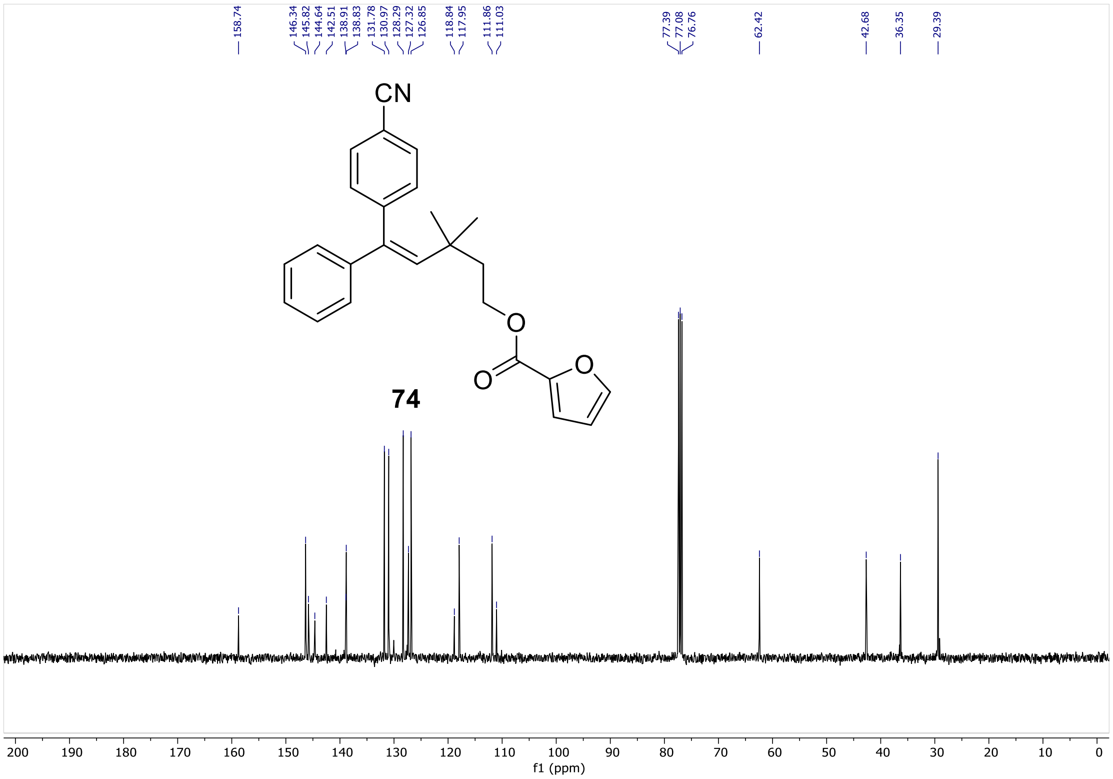

Supplementary Figure 186: ^{13}C NMR spectrum of compound **74**

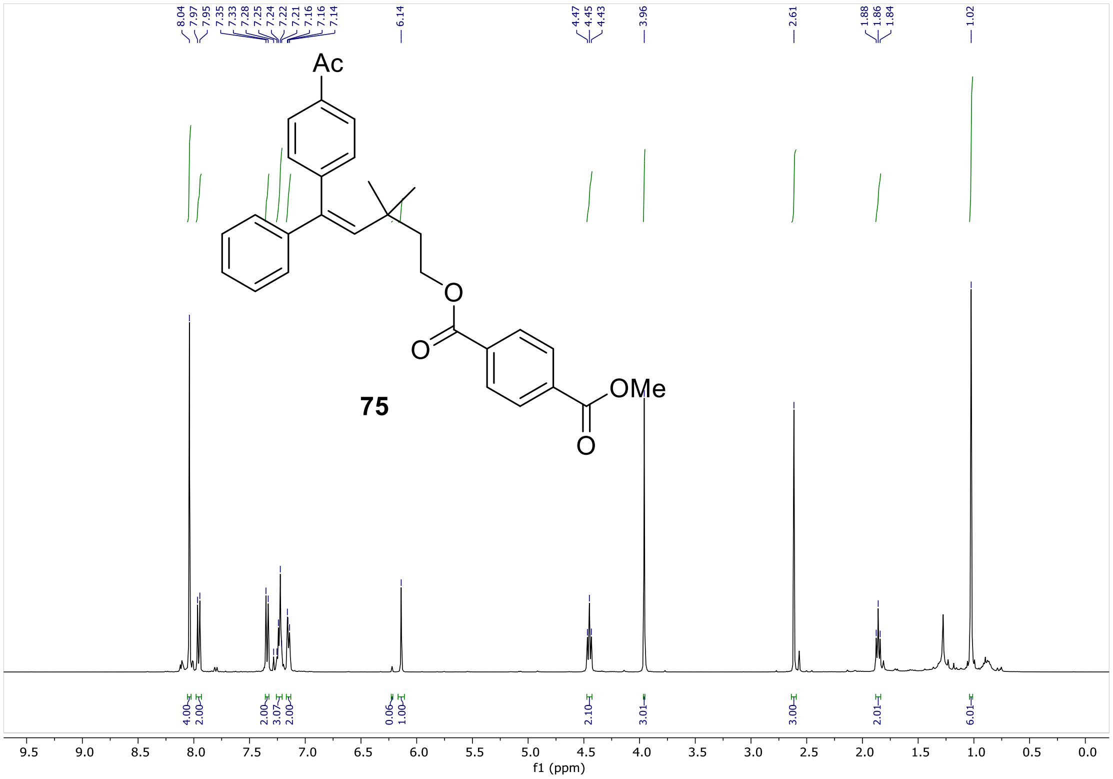

Supplementary Figure 187: ^{1}H NMR spectrum of compound **75**

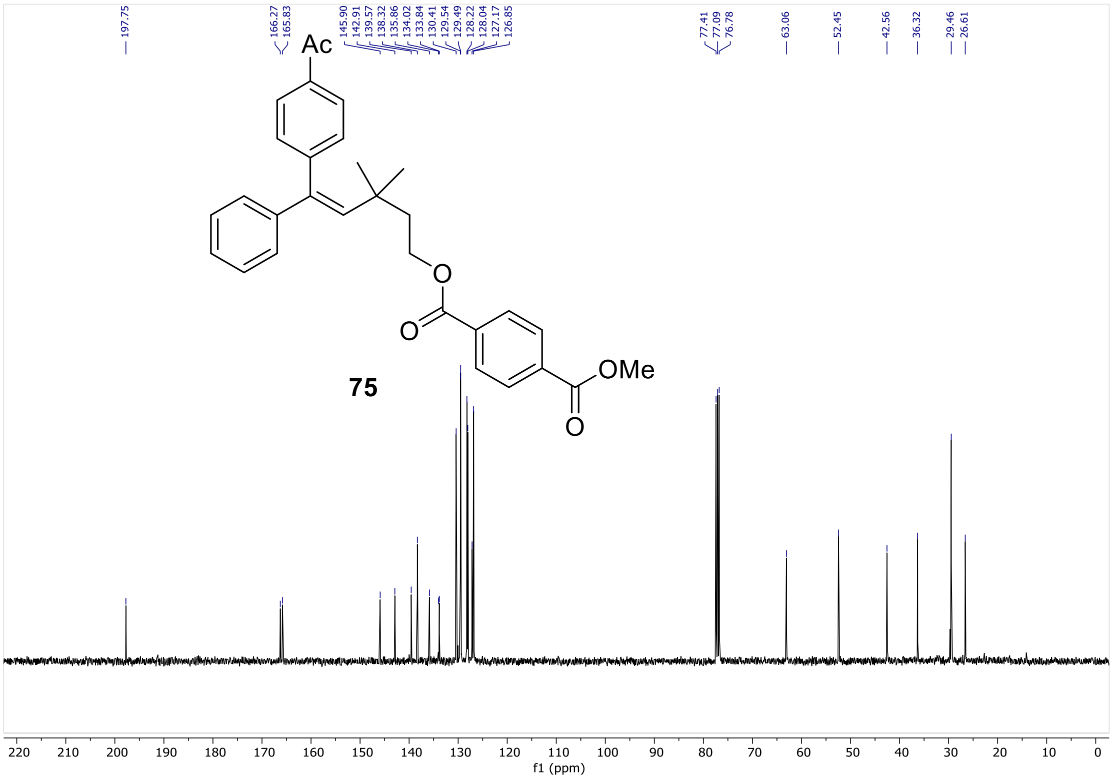

Supplementary Figure 188: ^{13}C NMR spectrum of compound **75**

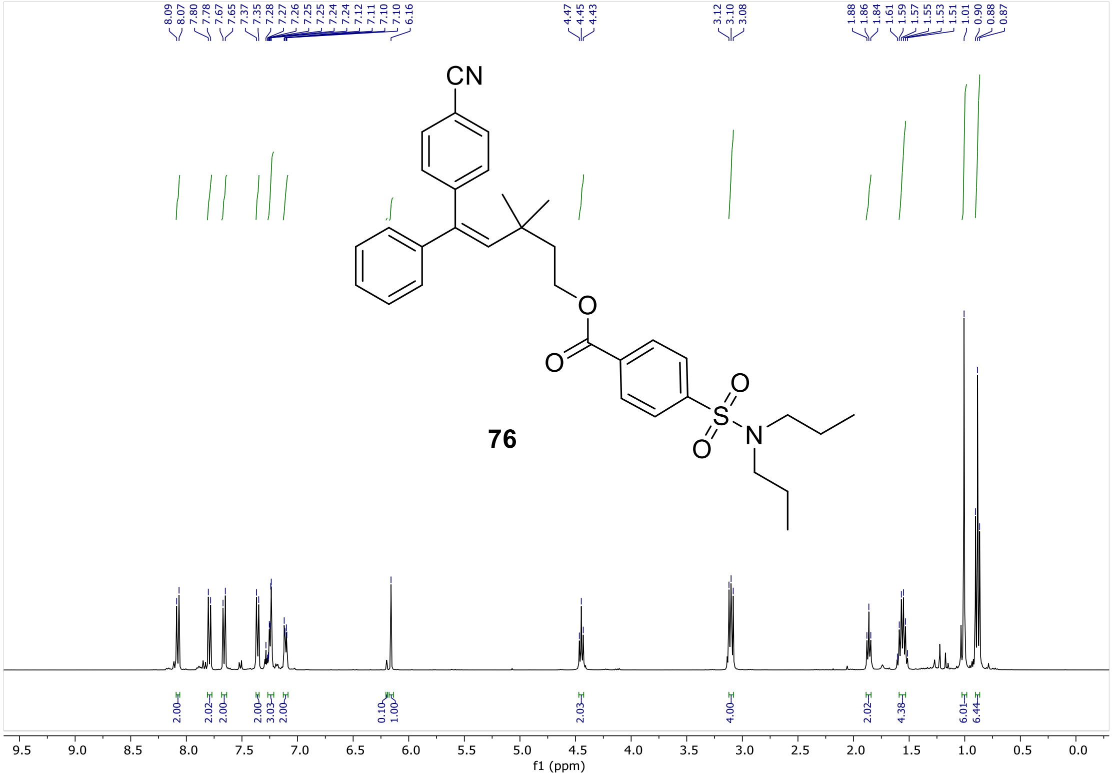

Supplementary Figure 189: ^{1}H NMR spectrum of compound **76**

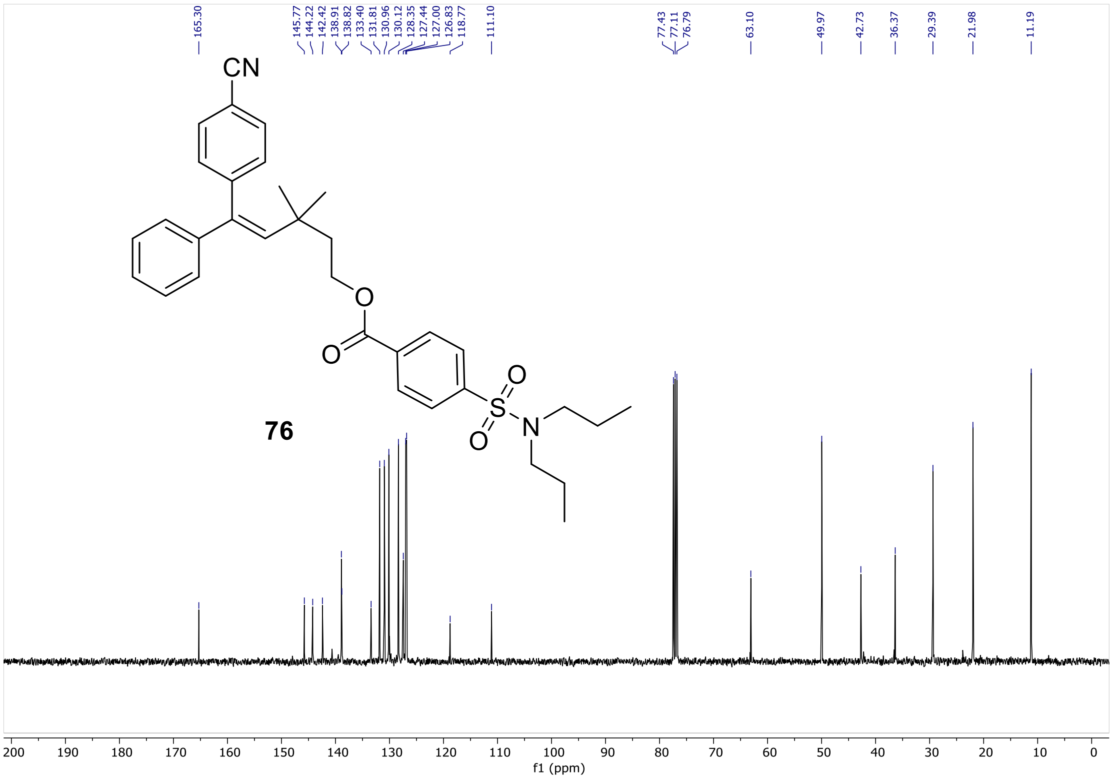

Supplementary Figure 190: ^{13}C NMR spectrum of compound **76**

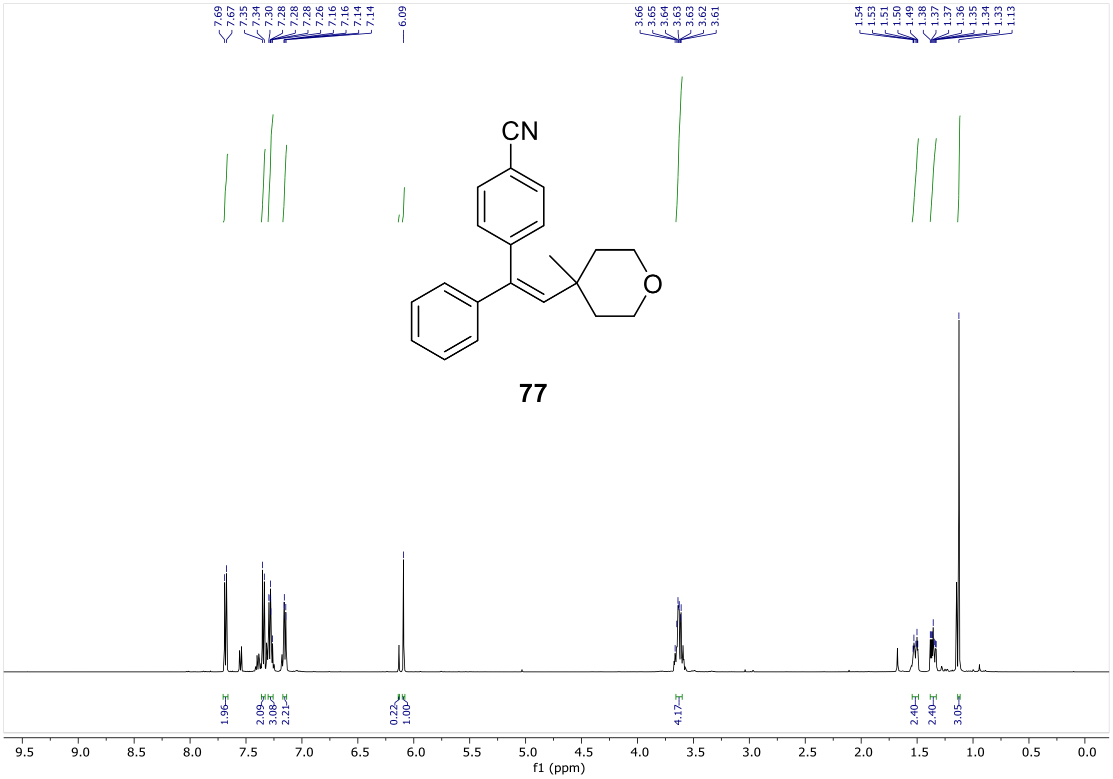

Supplementary Figure 191: ^{1}H NMR spectrum of compound **77**

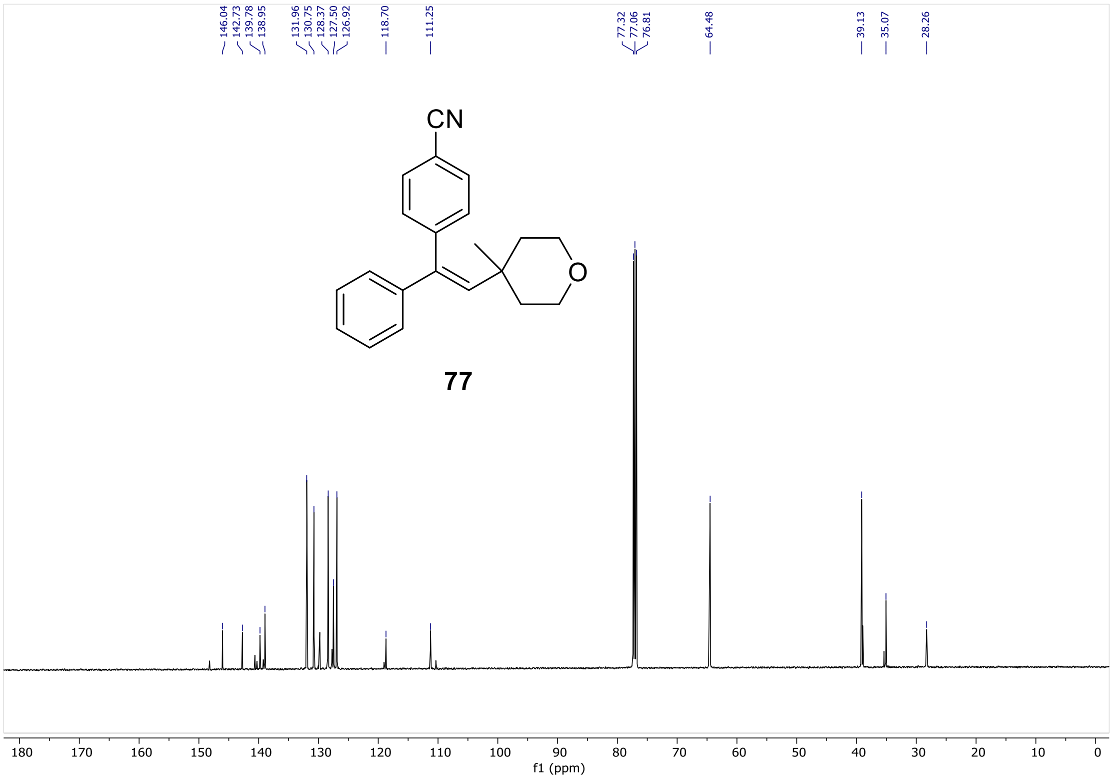

Supplementary Figure 192: ^{13}C NMR spectrum of compound **77**

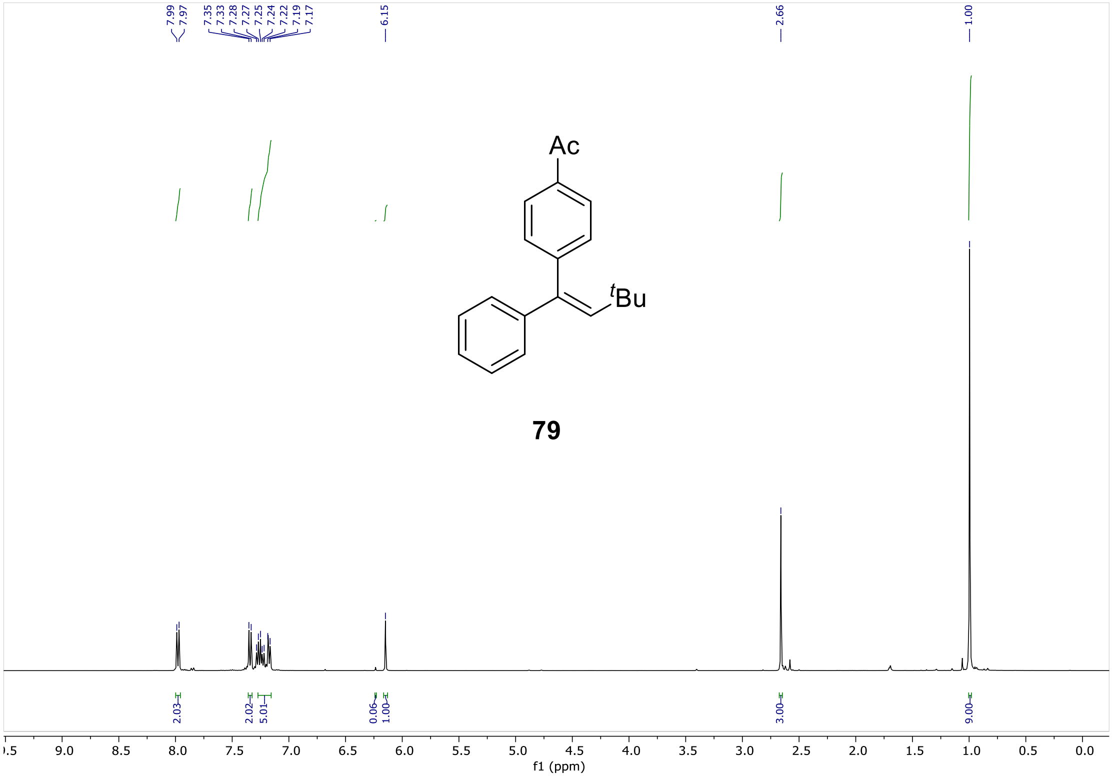

Supplementary Figure 193: ^{1}H NMR spectrum of compound **79**

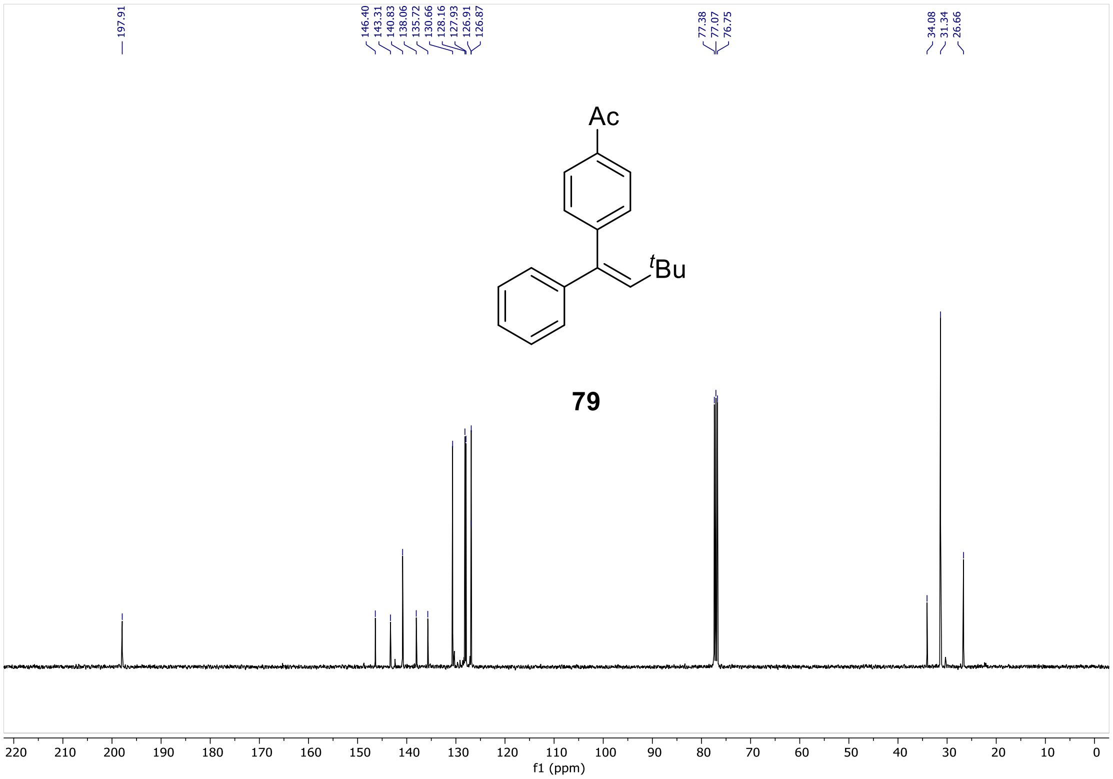

Supplementary Figure 194: ^{13}C NMR spectrum of compound **79**

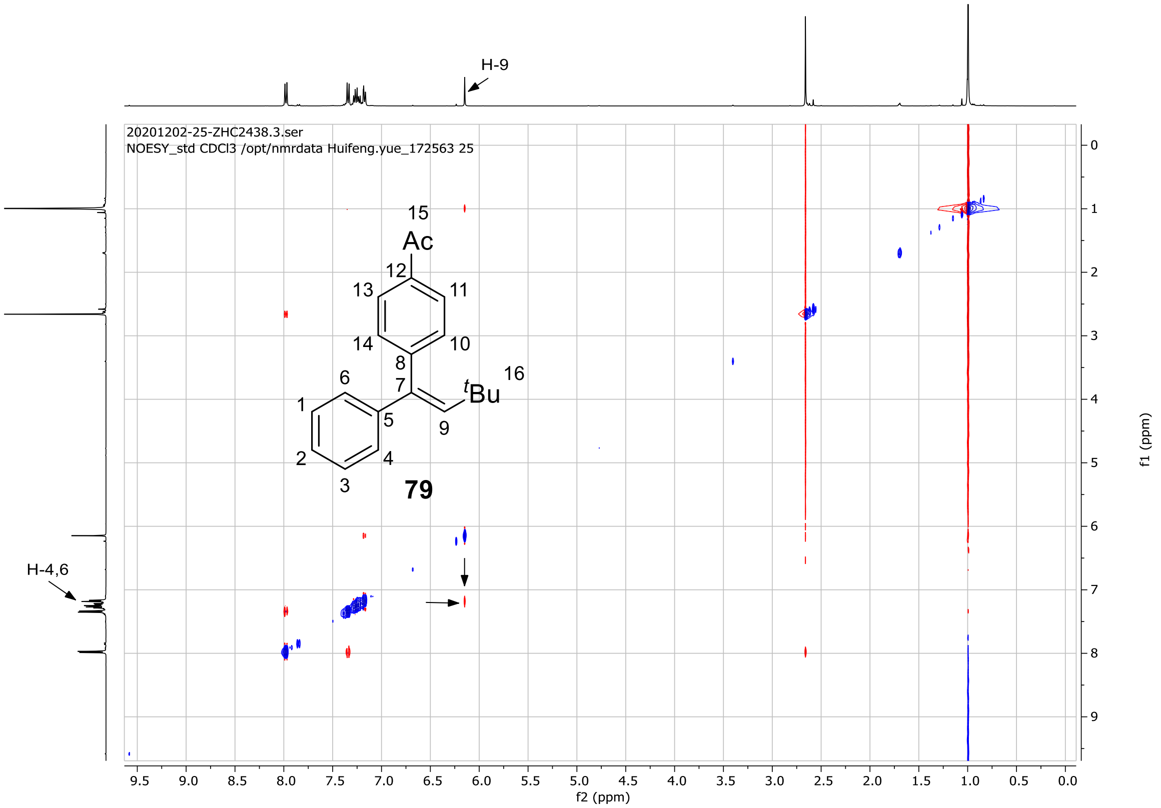

Supplementary Figure 195: NOE spectrum of compound **79**

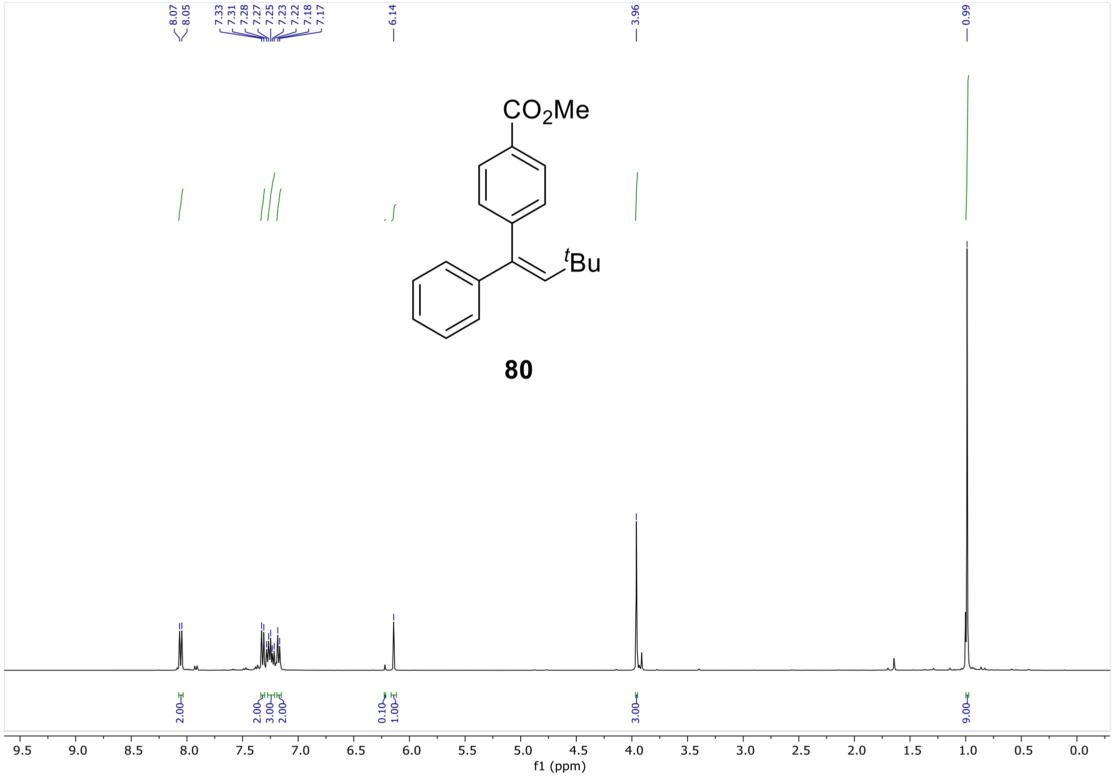

Supplementary Figure 196: ^{1}H NMR spectrum of compound **80**

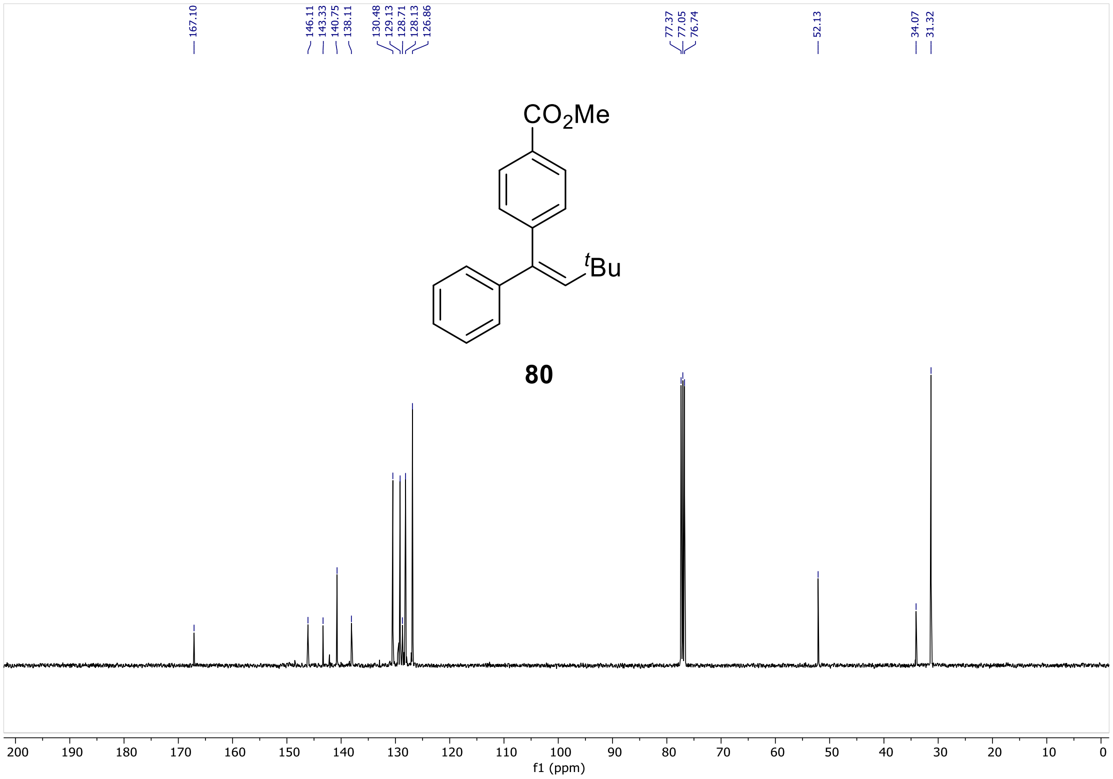

Supplementary Figure 197: ^{13}C NMR spectrum of compound **80**

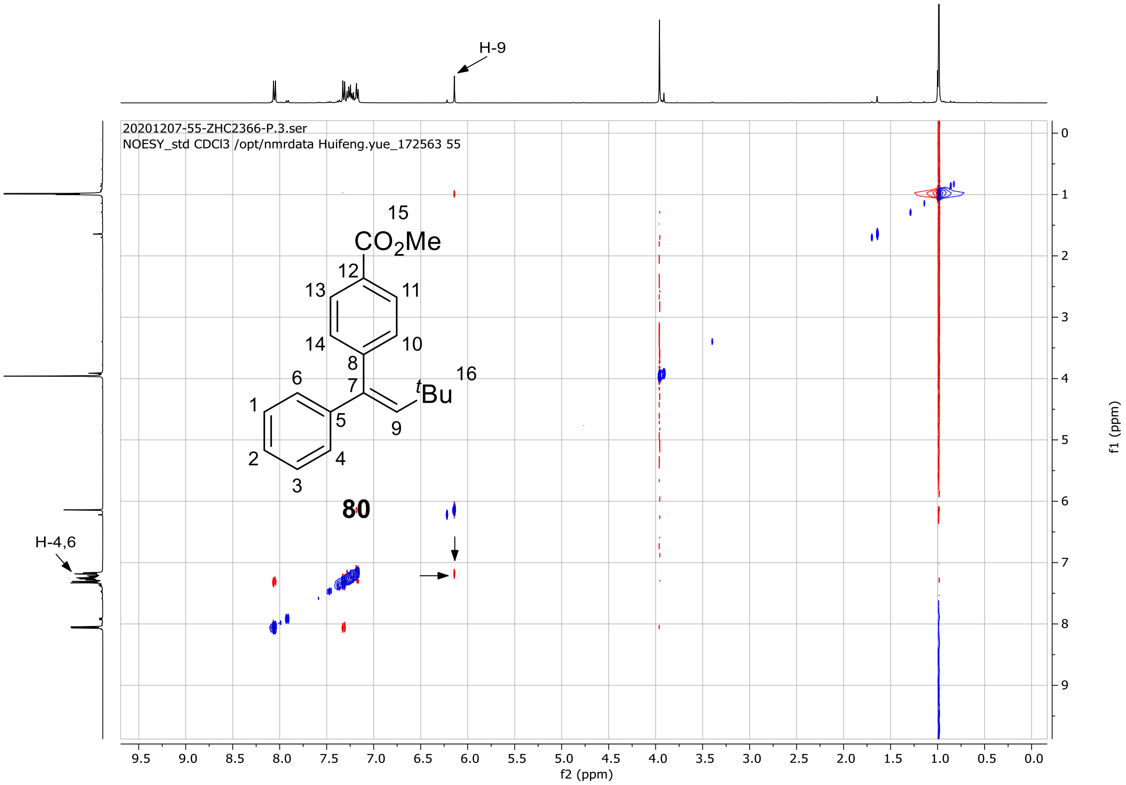

Supplementary Figure 198: NOE spectrum of compound **80**

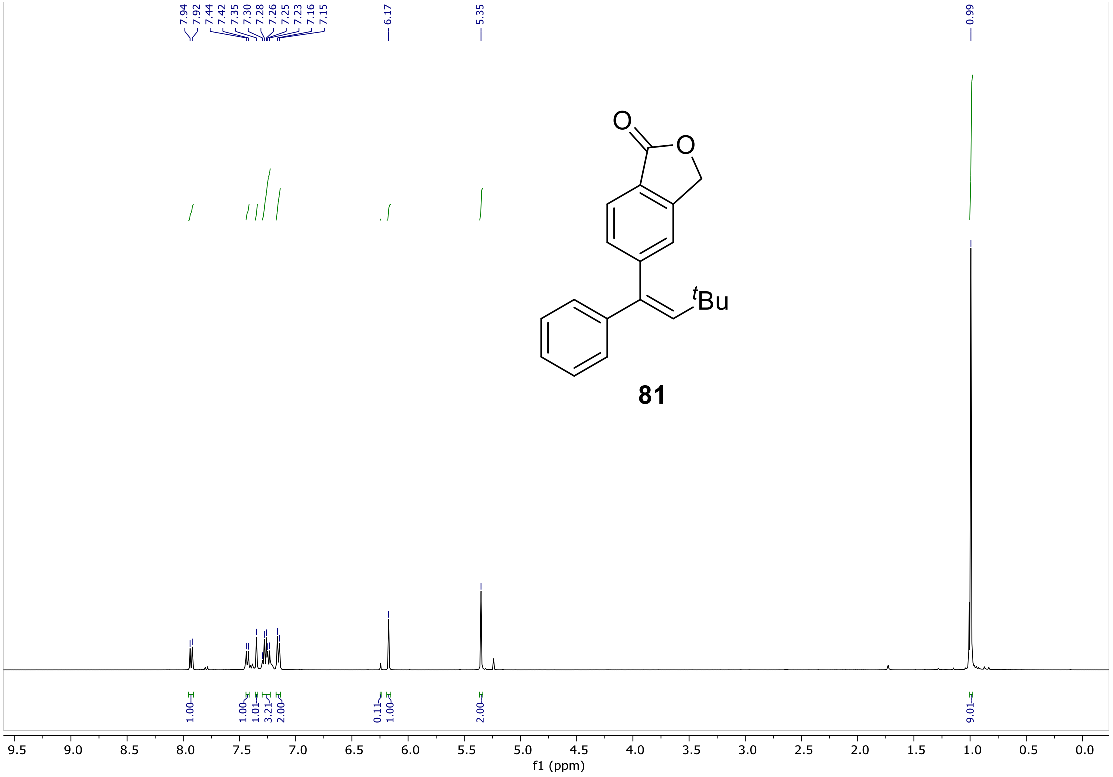

Supplementary Figure 199: ^{1}H NMR spectrum of compound **81**

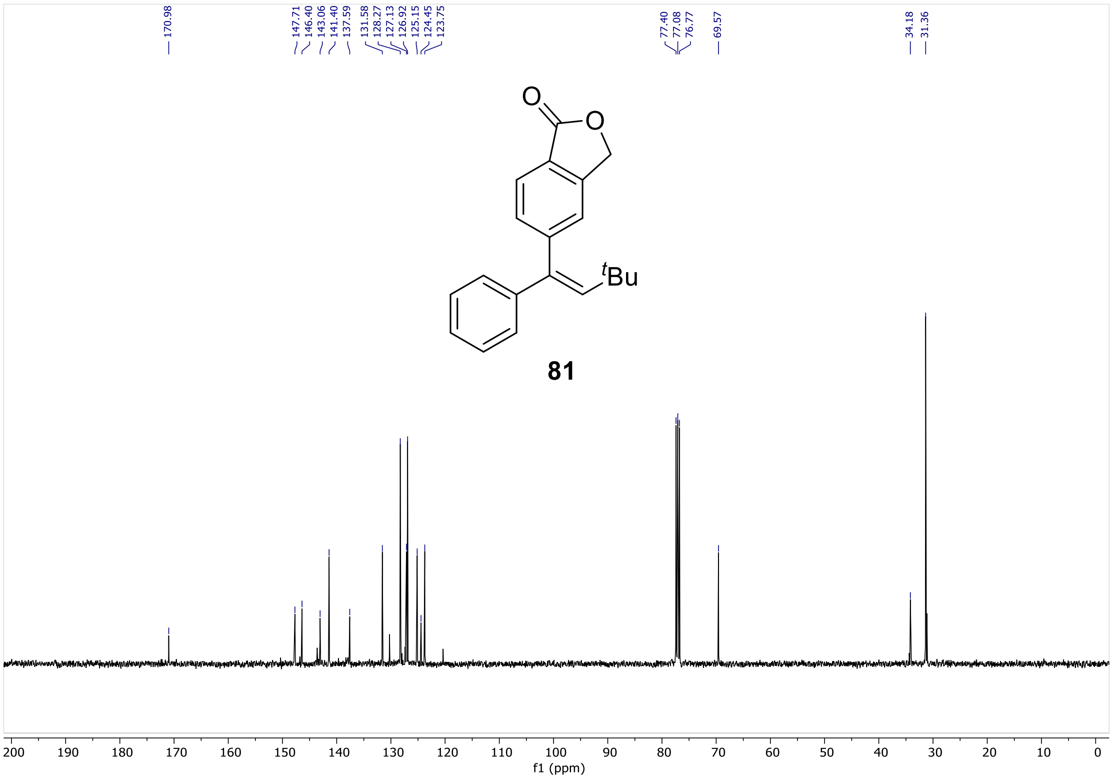

Supplementary Figure 200: ^{13}C NMR spectrum of compound **81**

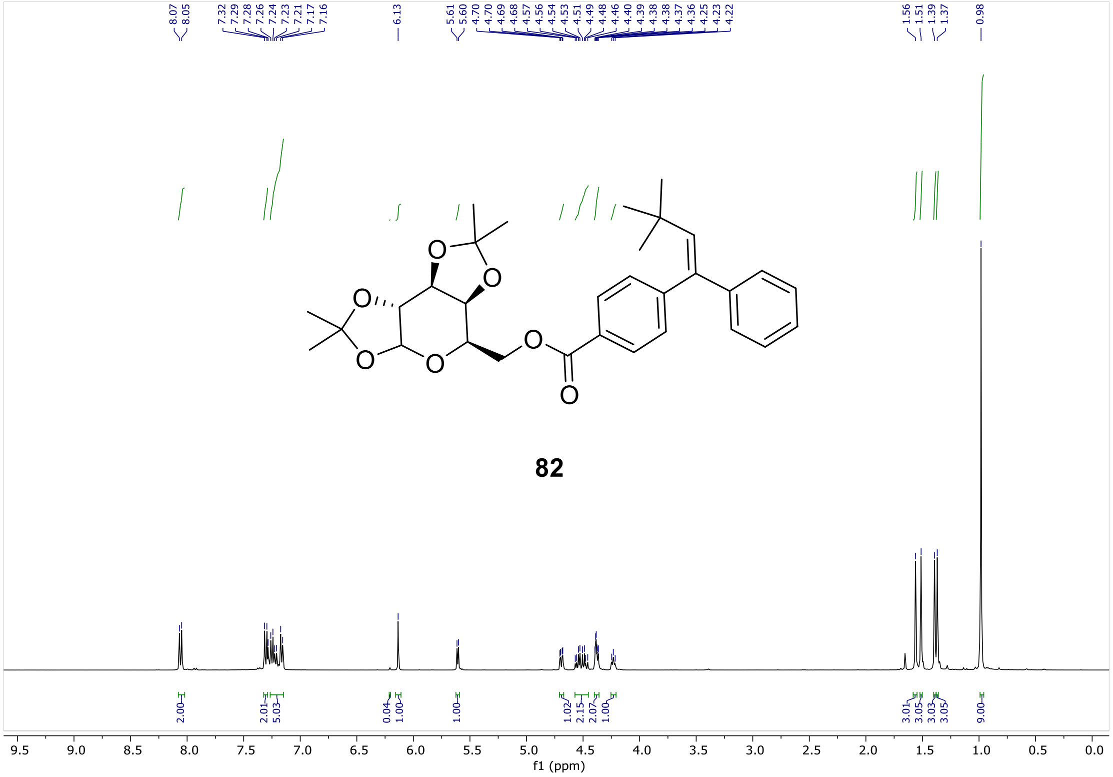

Supplementary Figure 201: ^{1}H NMR spectrum of compound **82**

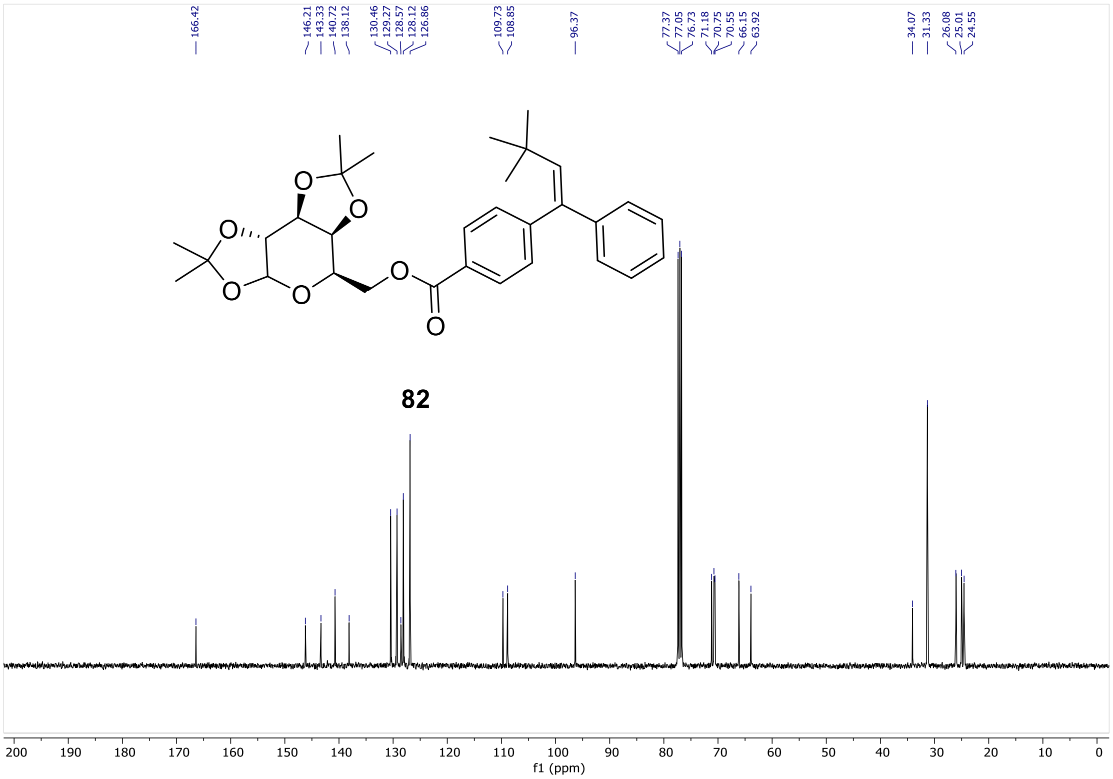

Supplementary Figure 202: ^{13}C NMR spectrum of compound **82**

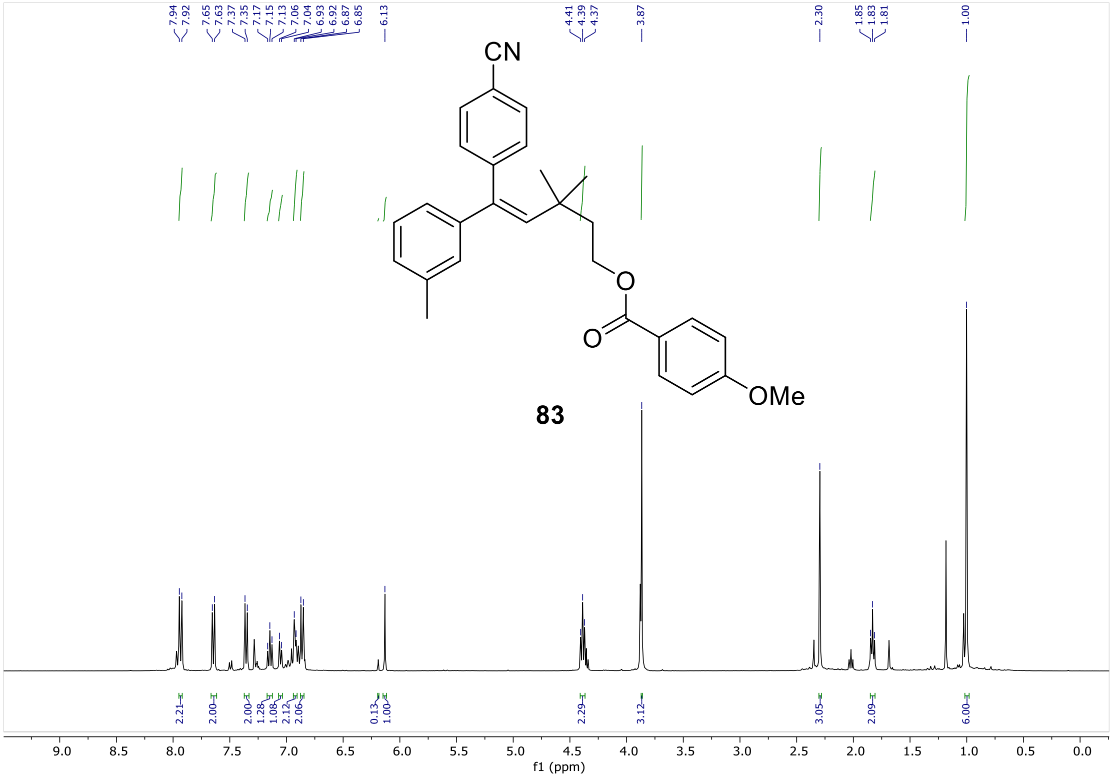

Supplementary Figure 203: ^{1}H NMR spectrum of compound **83**

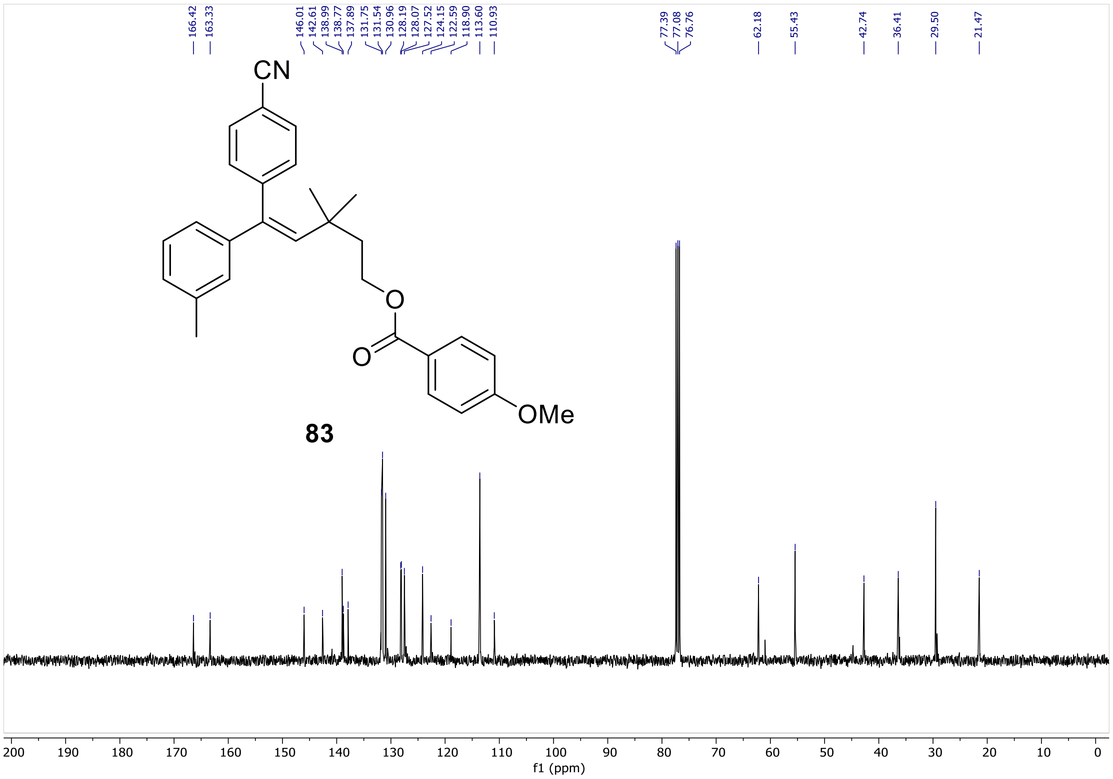

Supplementary Figure 204: ^{13}C NMR spectrum of compound **83**

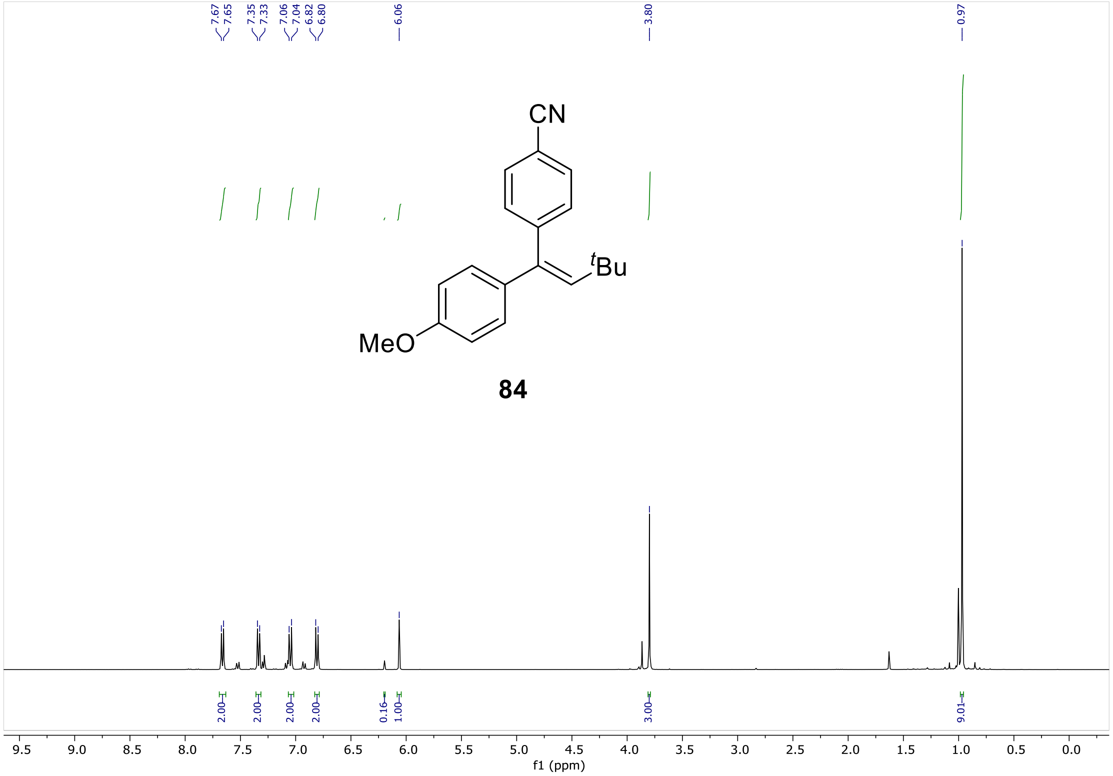

Supplementary Figure 205: ^{1}H NMR spectrum of compound **84**

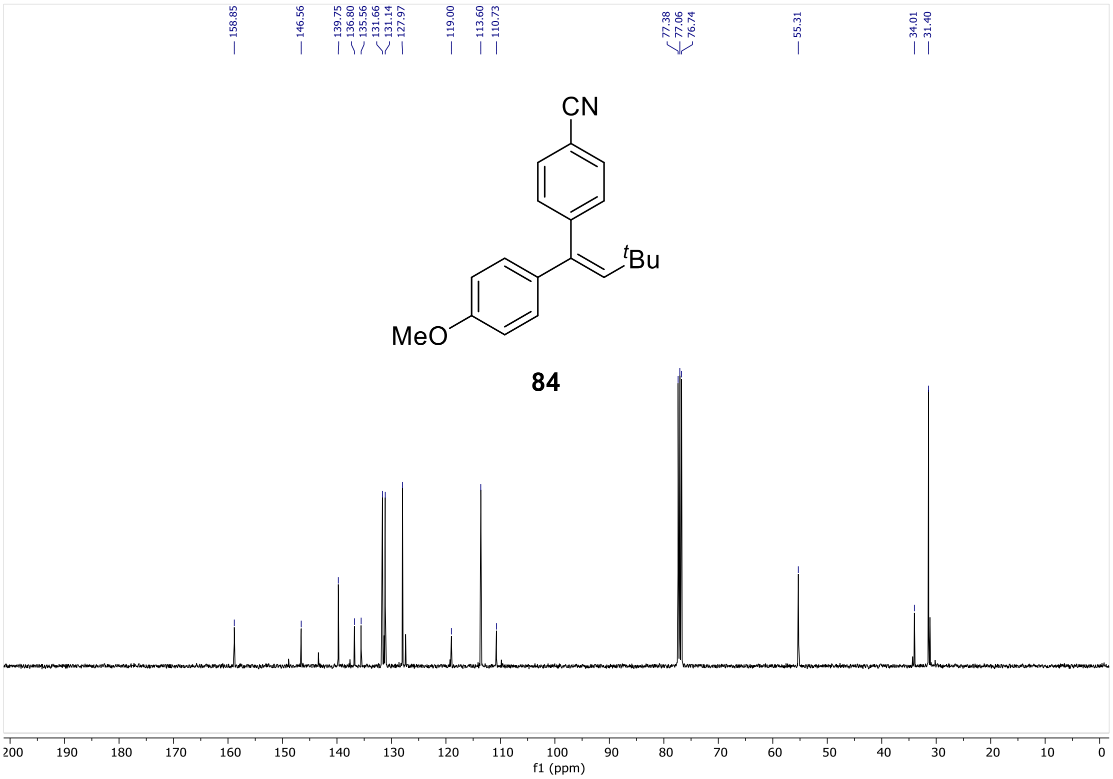

Supplementary Figure 206: ^{13}C NMR spectrum of compound **84**

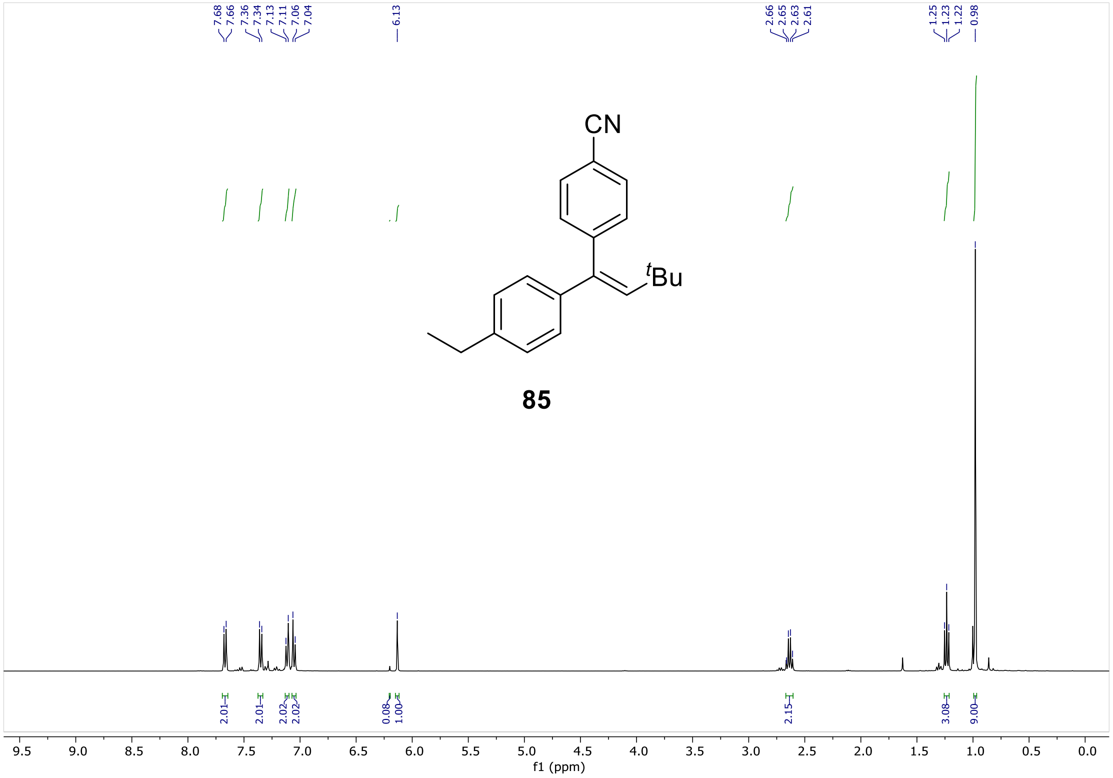

Supplementary Figure 207: ^{1}H NMR spectrum of compound **85**

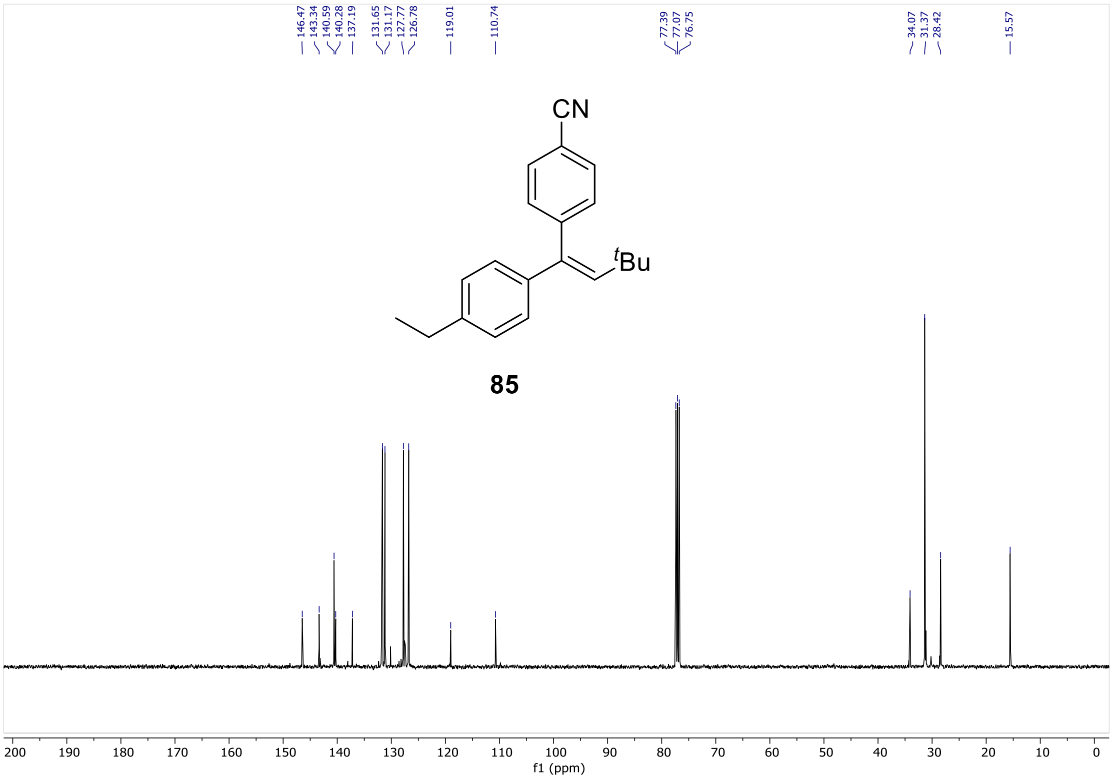

Supplementary Figure 208: ^{13}C NMR spectrum of compound **85**

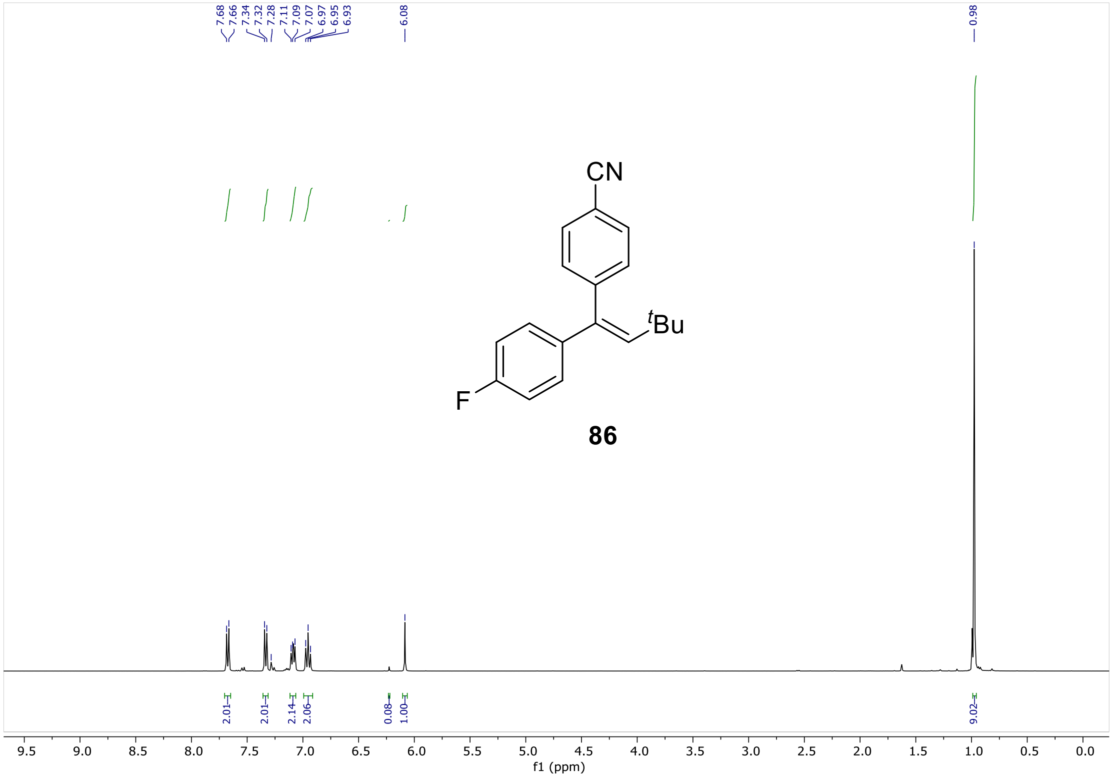

Supplementary Figure 209: ^{1}H NMR spectrum of compound **86**

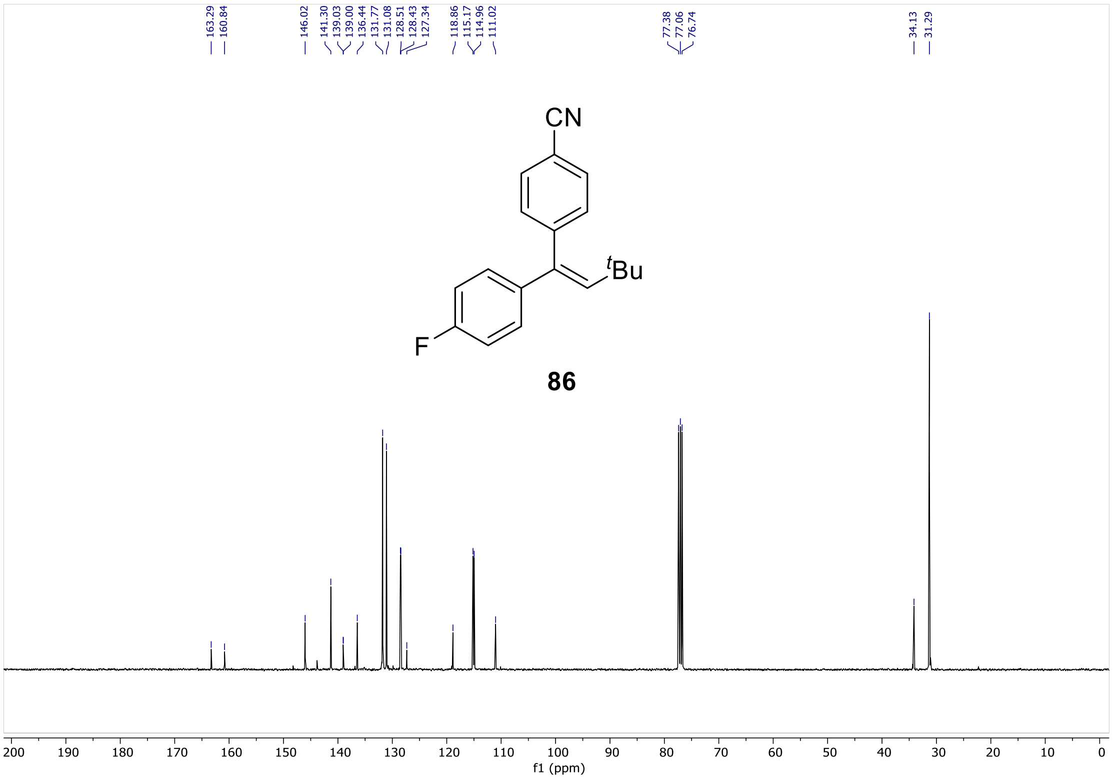

Supplementary Figure 210: ^{13}C NMR spectrum of compound **86**

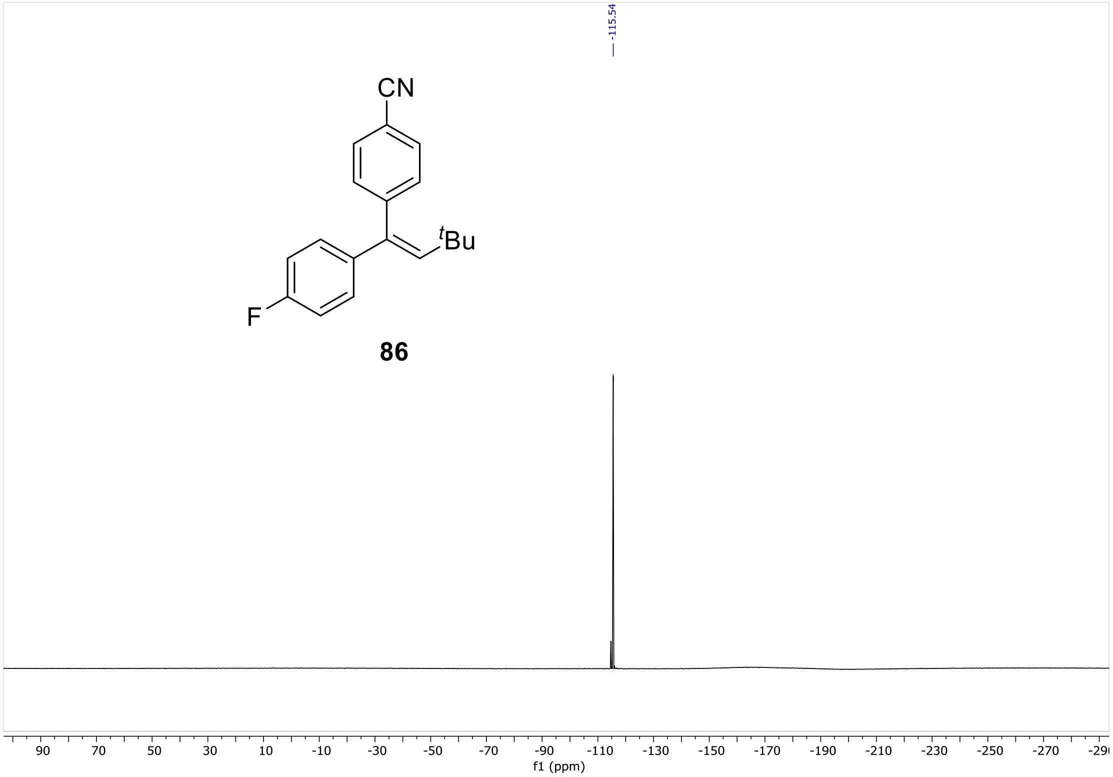

Supplementary Figure 211: ^{19}F NMR spectrum of compound **86**

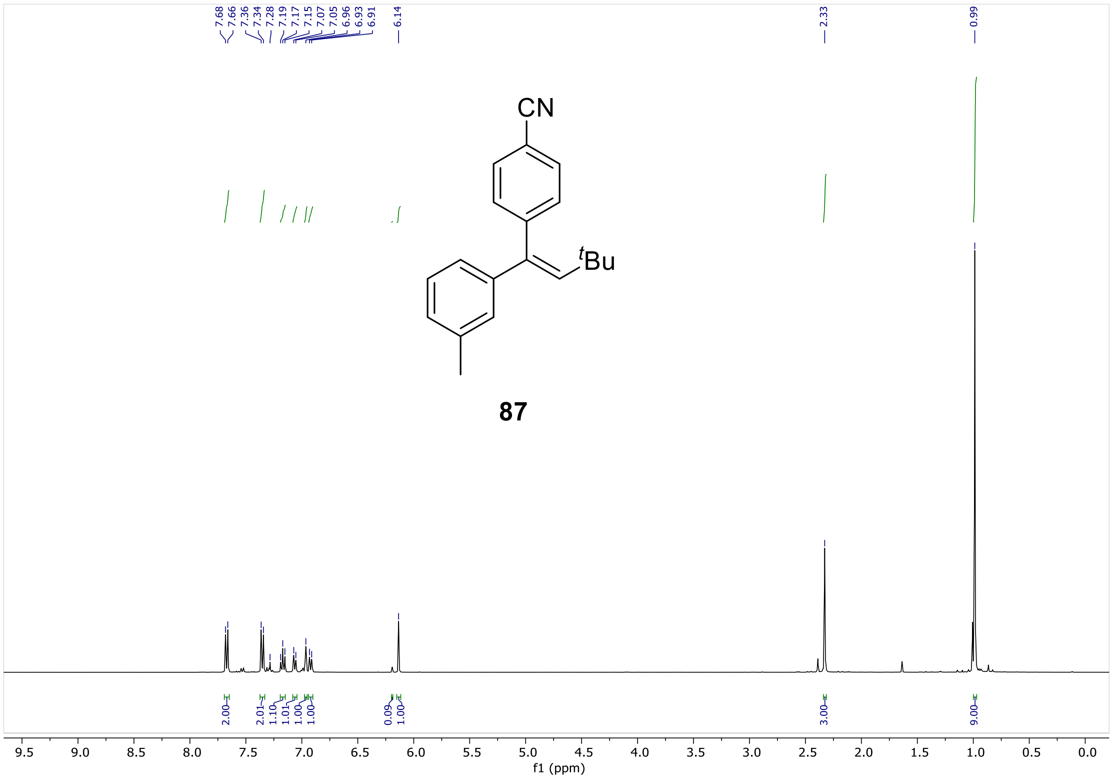

Supplementary Figure 212: ^{1}H NMR spectrum of compound **87**

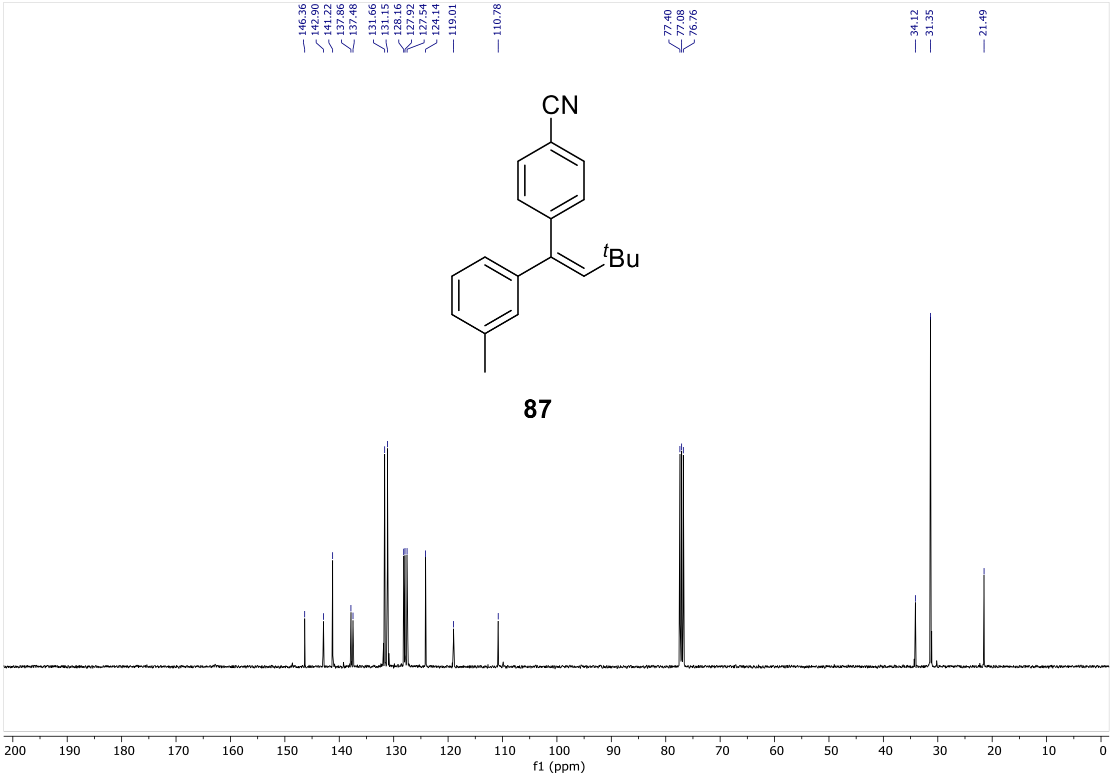

Supplementary Figure 213: ^{13}C NMR spectrum of compound **87**

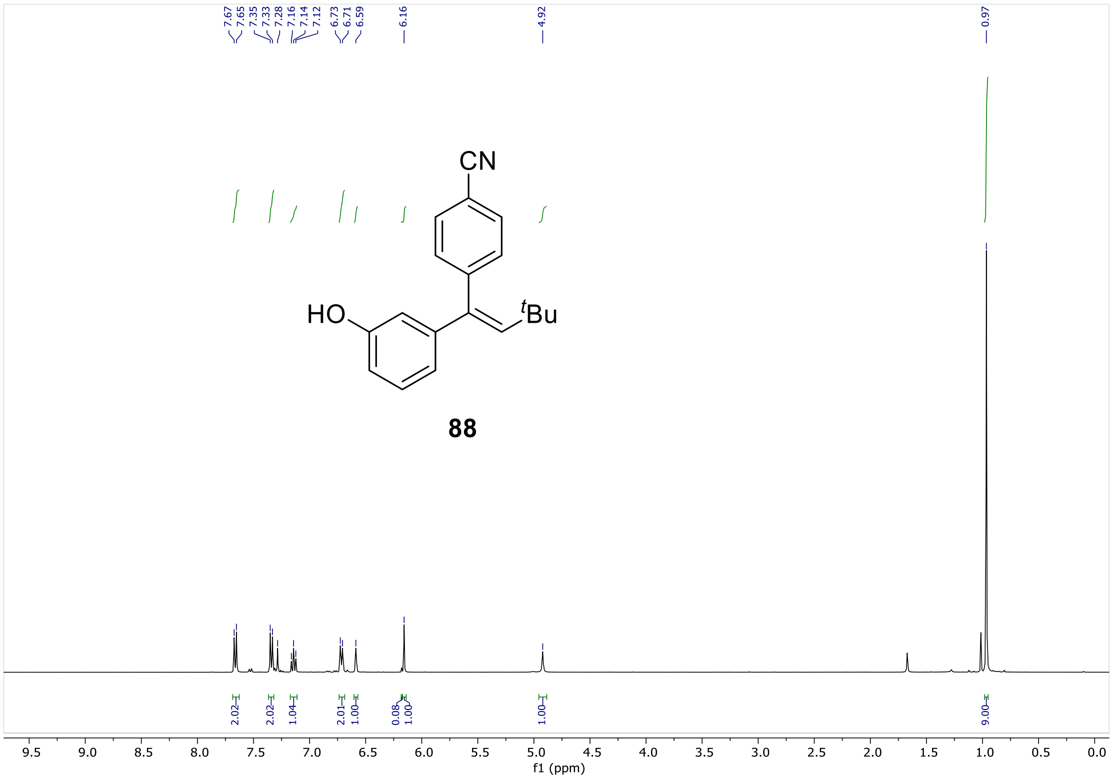

Supplementary Figure 214: ^{1}H NMR spectrum of compound **88**

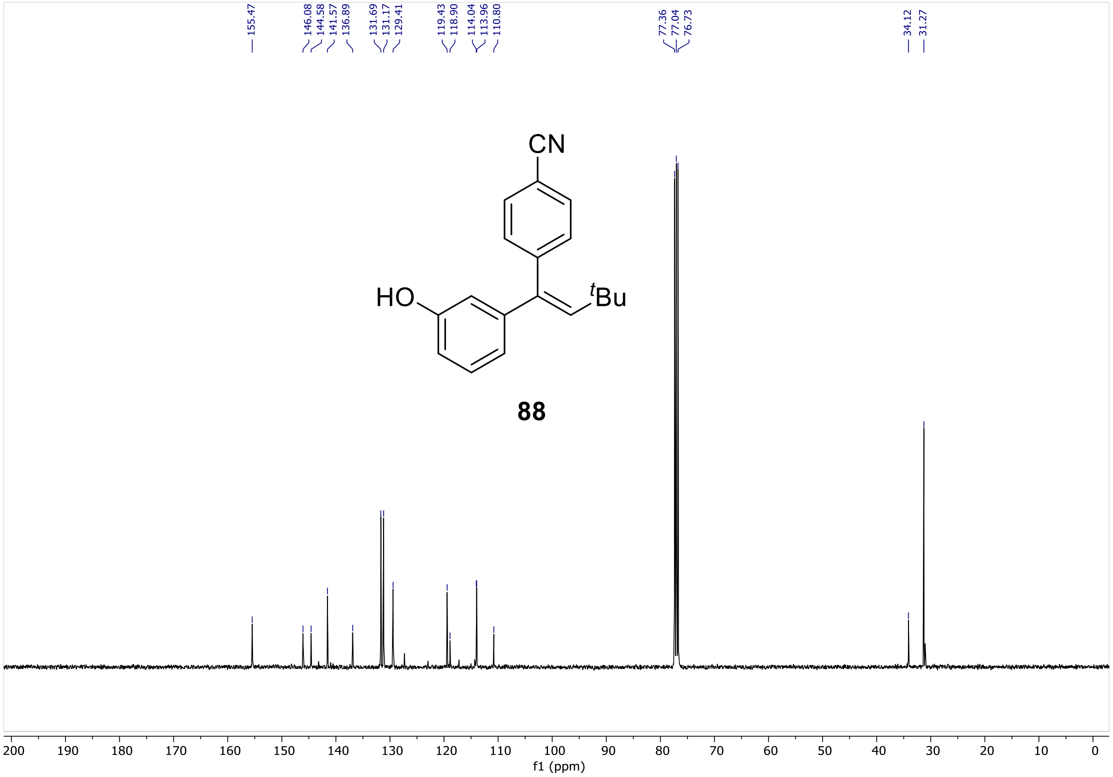

Supplementary Figure 215: ^{13}C NMR spectrum of compound **88**

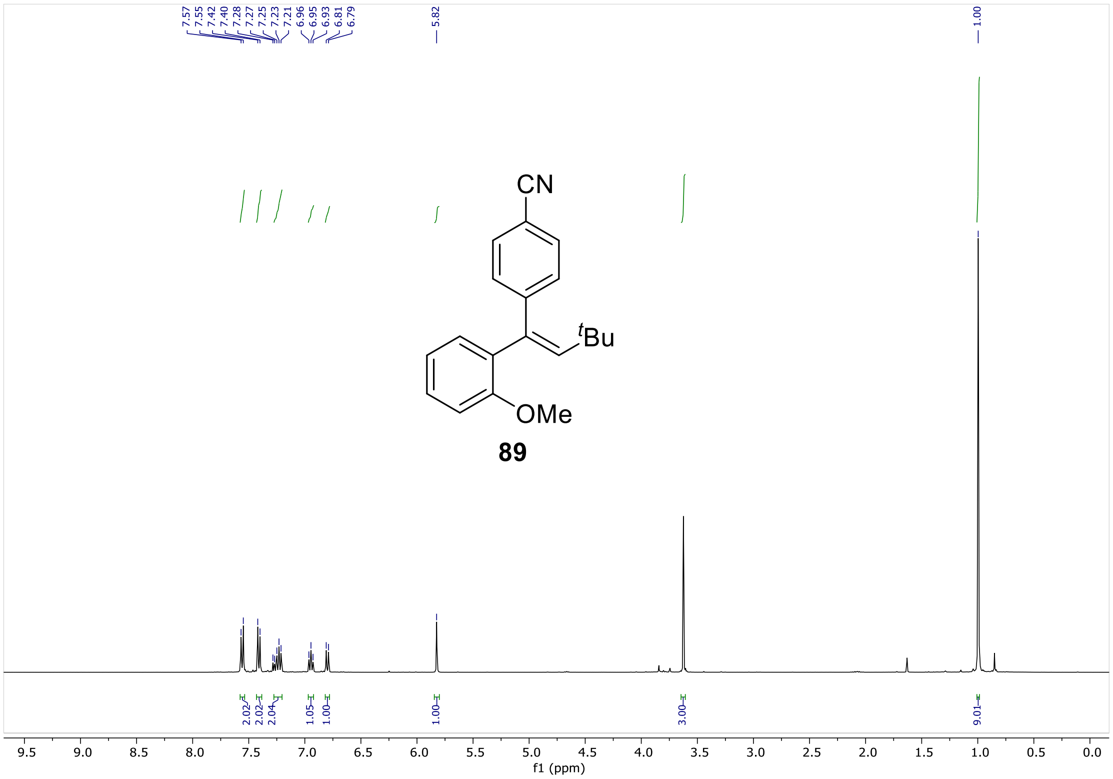

Supplementary Figure 216: ^{1}H NMR spectrum of compound **89**

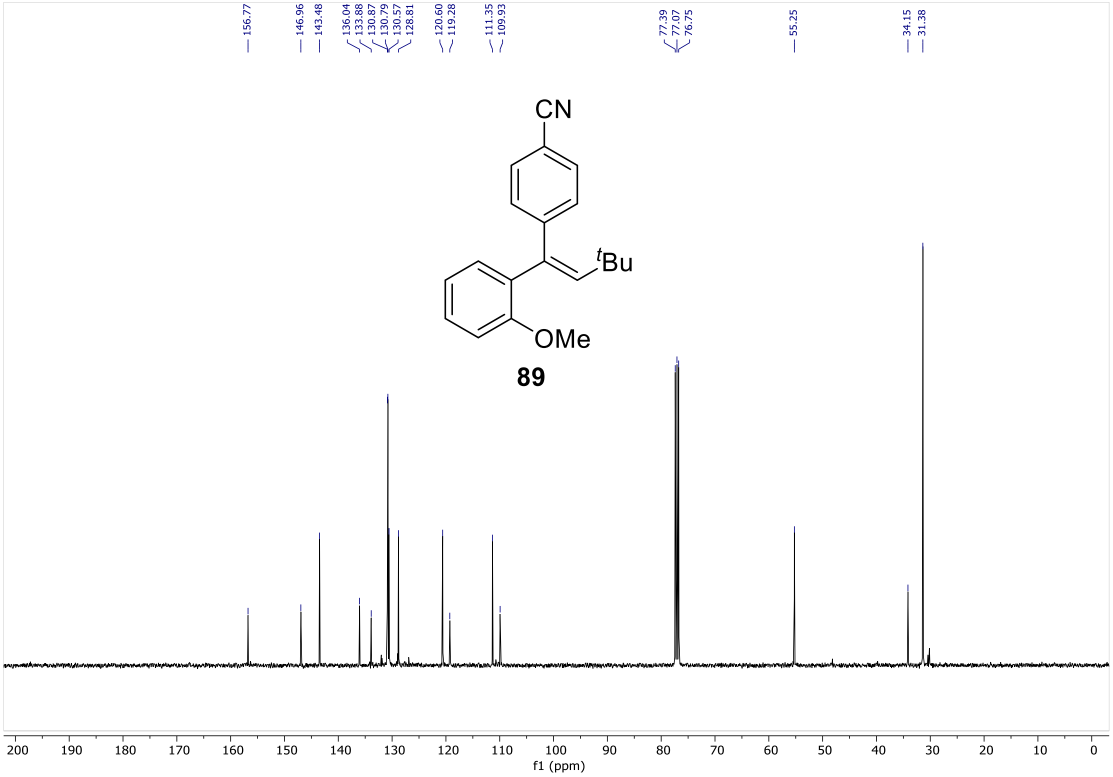

Supplementary Figure 217: ^{13}C NMR spectrum of compound **89**

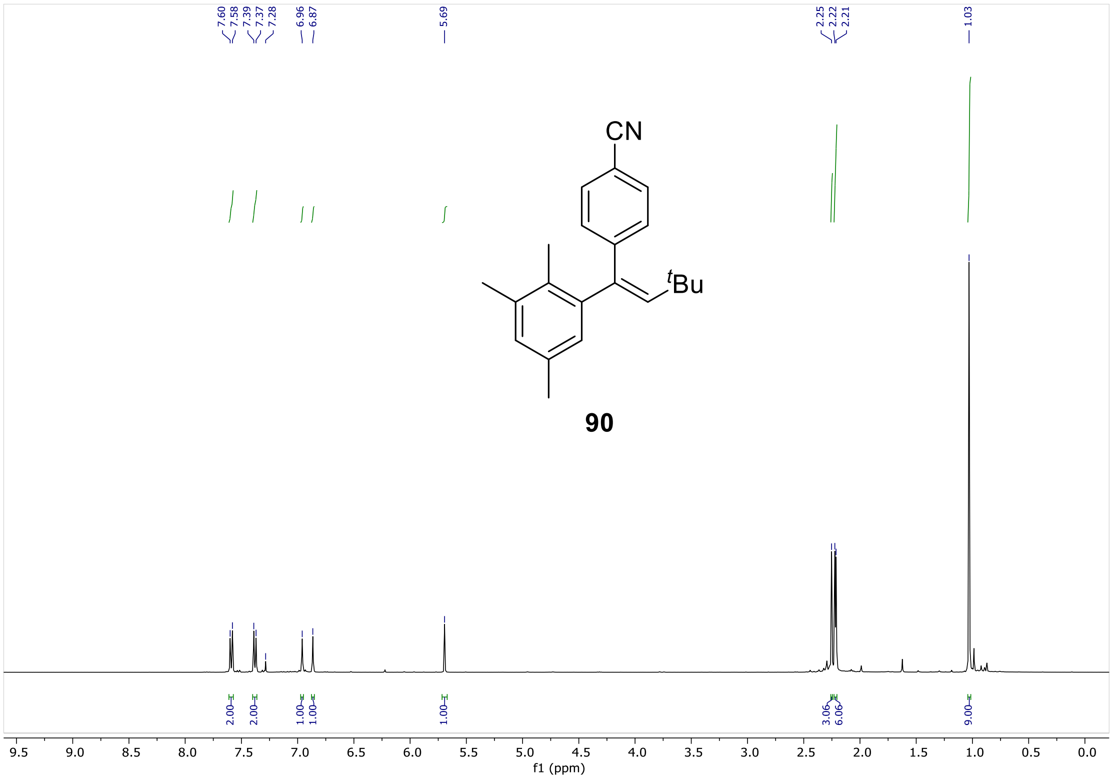

Supplementary Figure 218: ^{1}H NMR spectrum of compound **90**

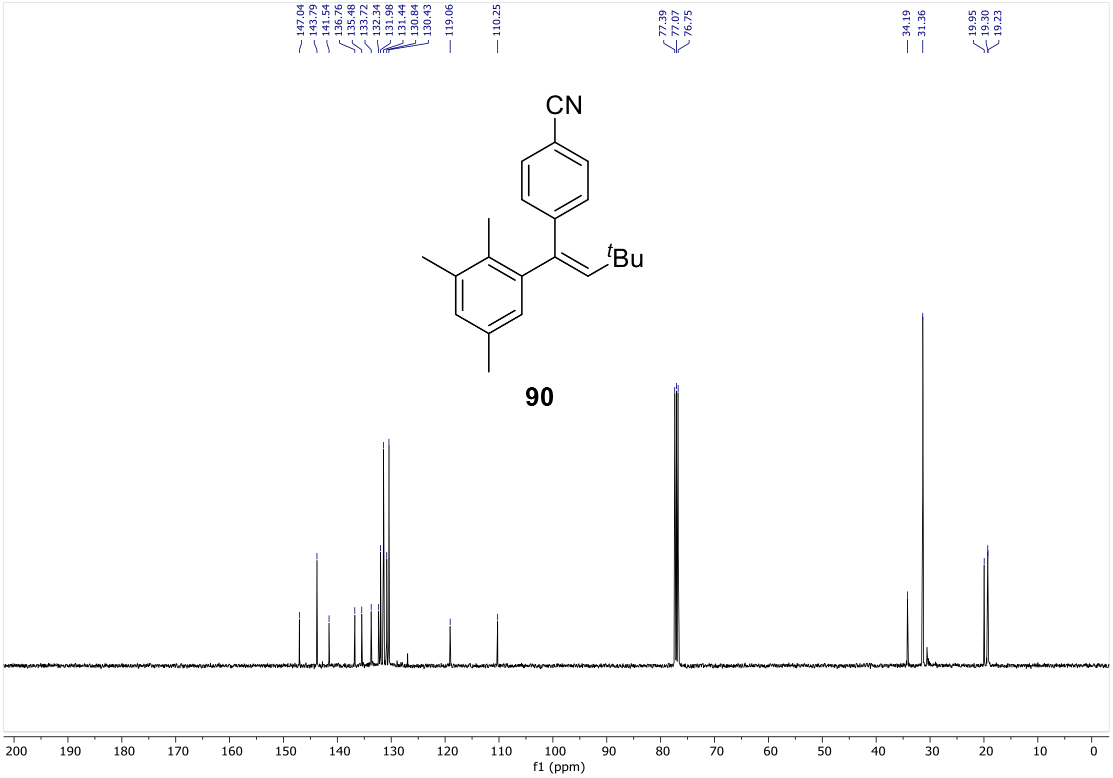

Supplementary Figure 219: ^{13}C NMR spectrum of compound **90**

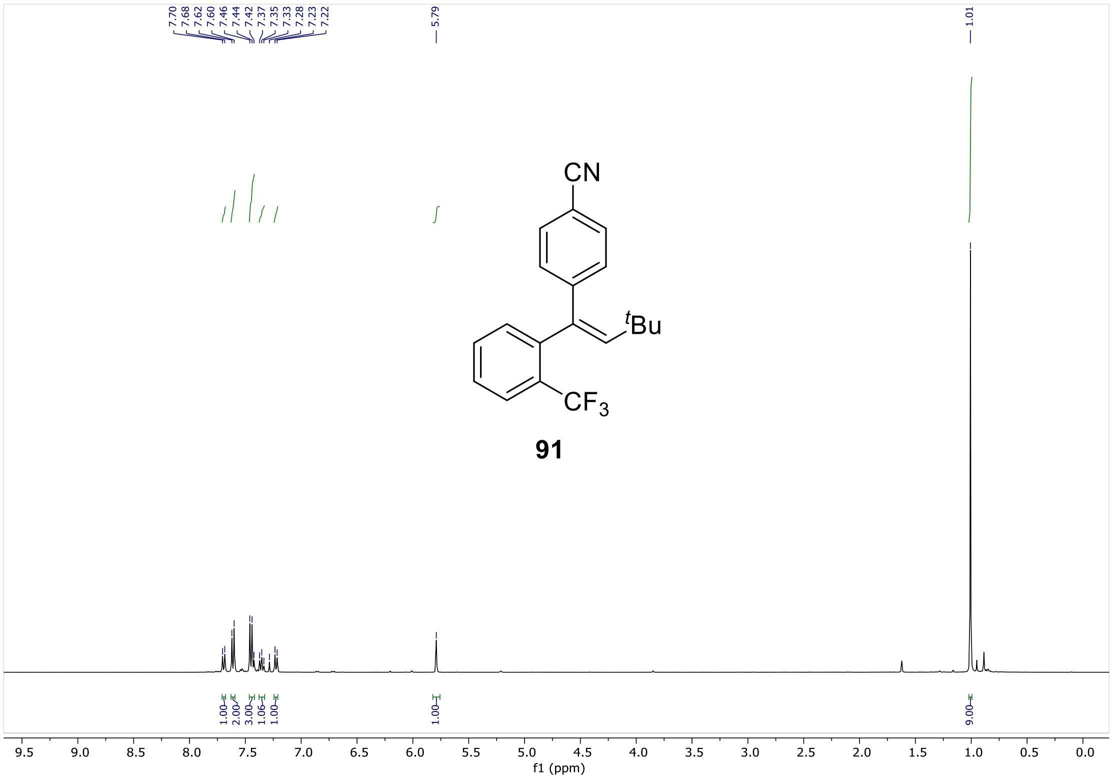

Supplementary Figure 220: ^{1}H NMR spectrum of compound **91**

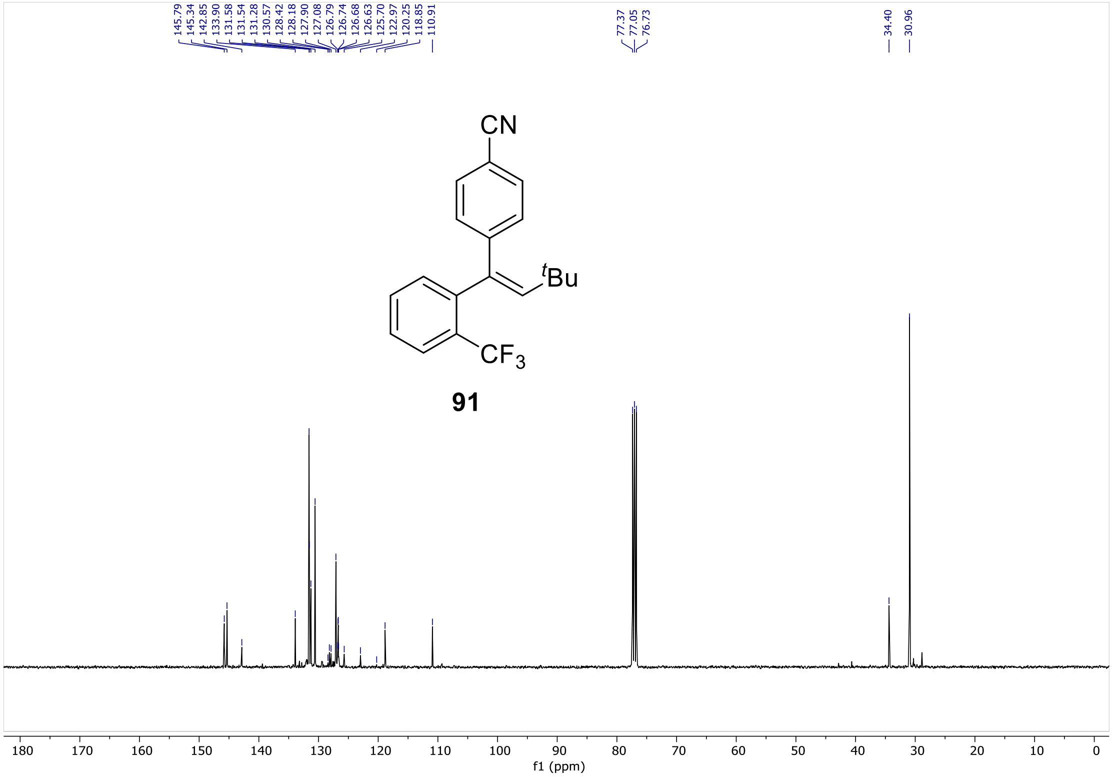

Supplementary Figure 221: ^{13}C NMR spectrum of compound **91**

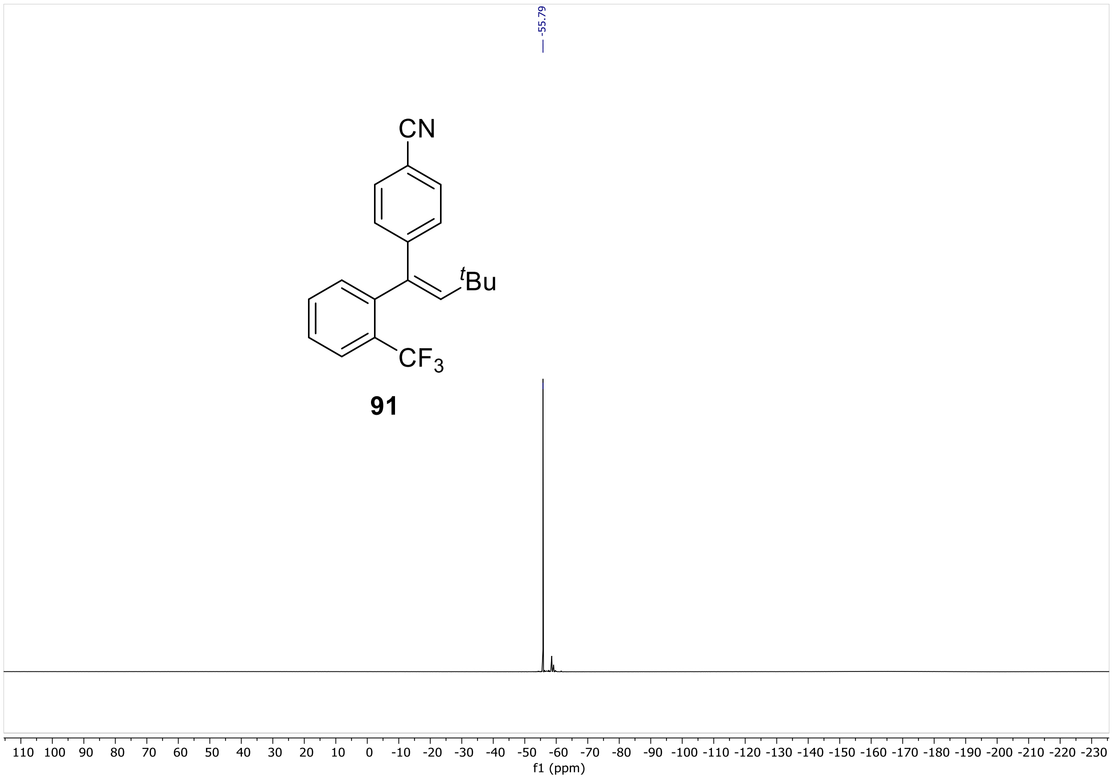

Supplementary Figure 222: ^{19}F NMR spectrum of compound **91**

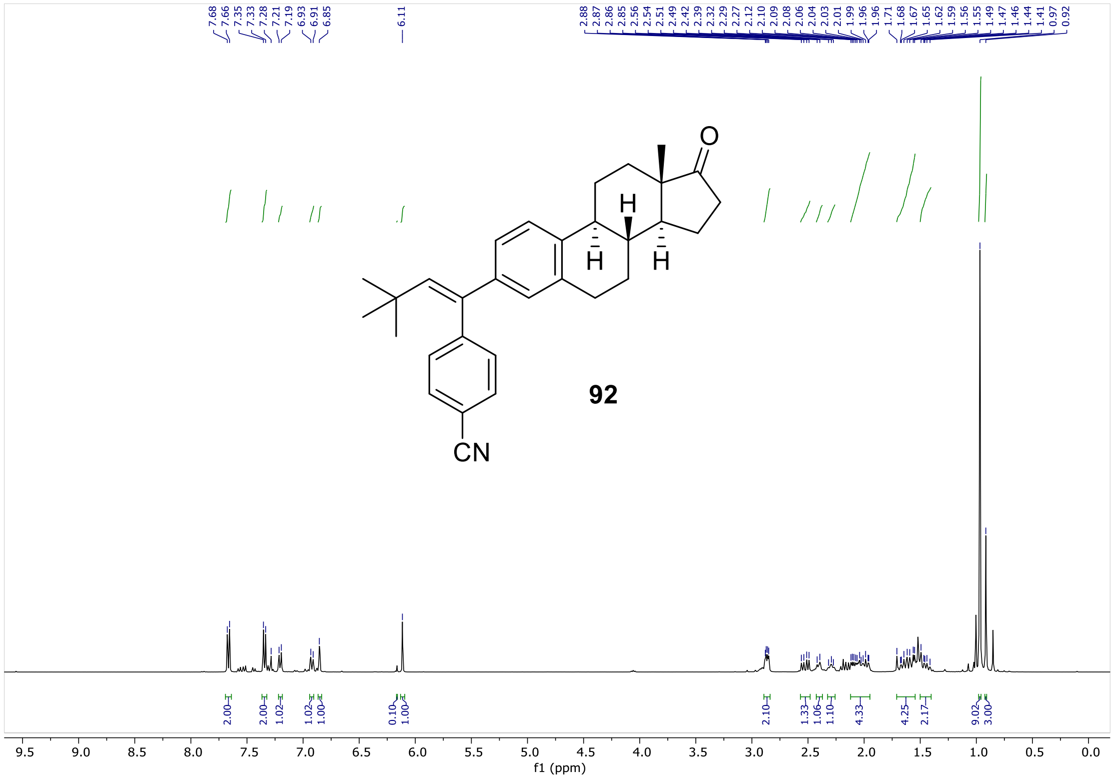

Supplementary Figure 223: ^{1}H NMR spectrum of compound **92**

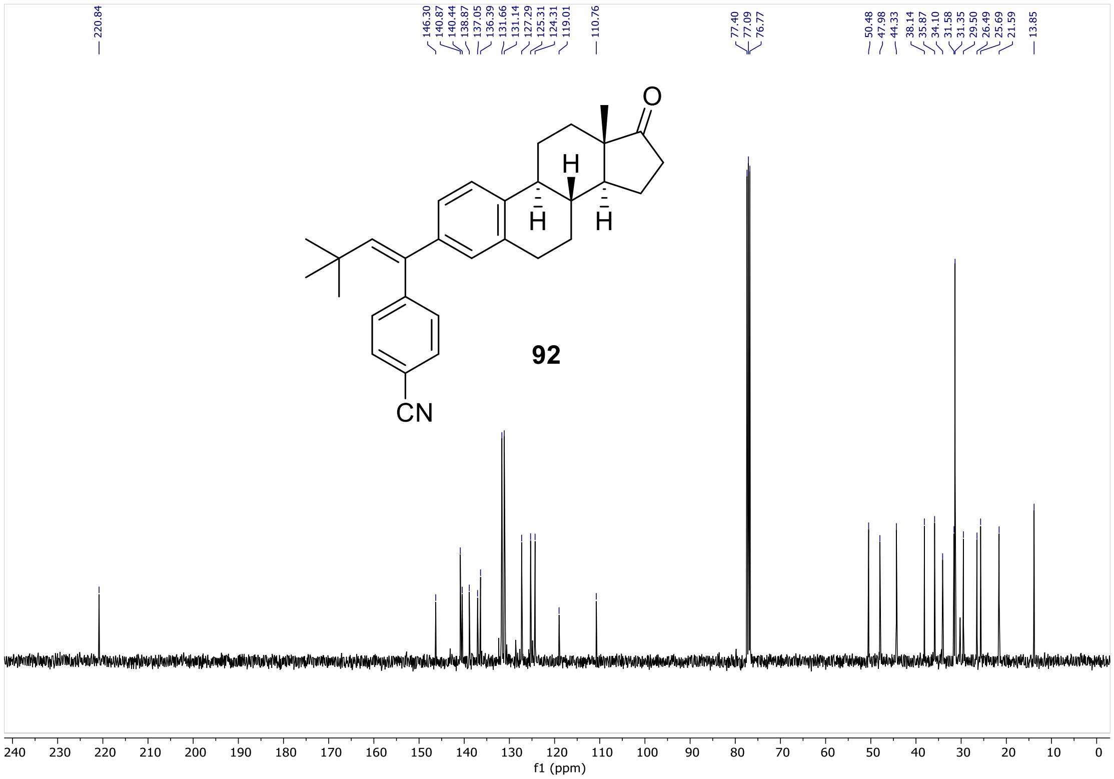

Supplementary Figure 224: ^{13}C NMR spectrum of compound **92**

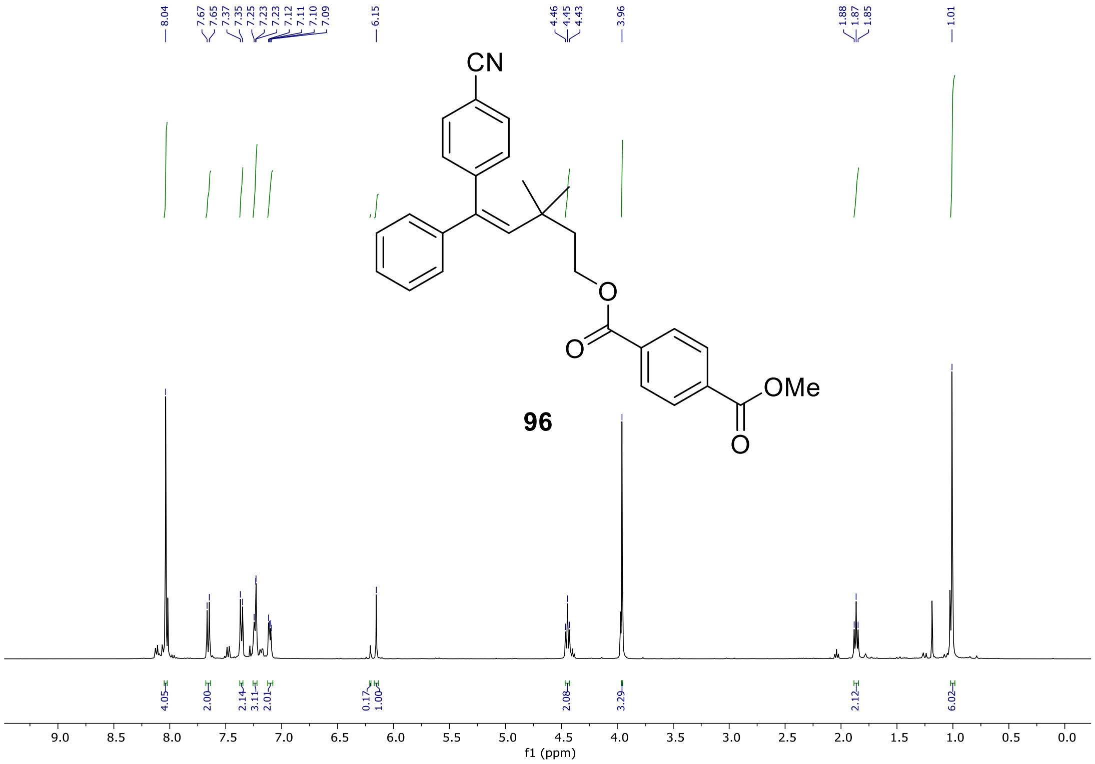

Supplementary Figure 225: ^{1}H NMR spectrum of compound **96**

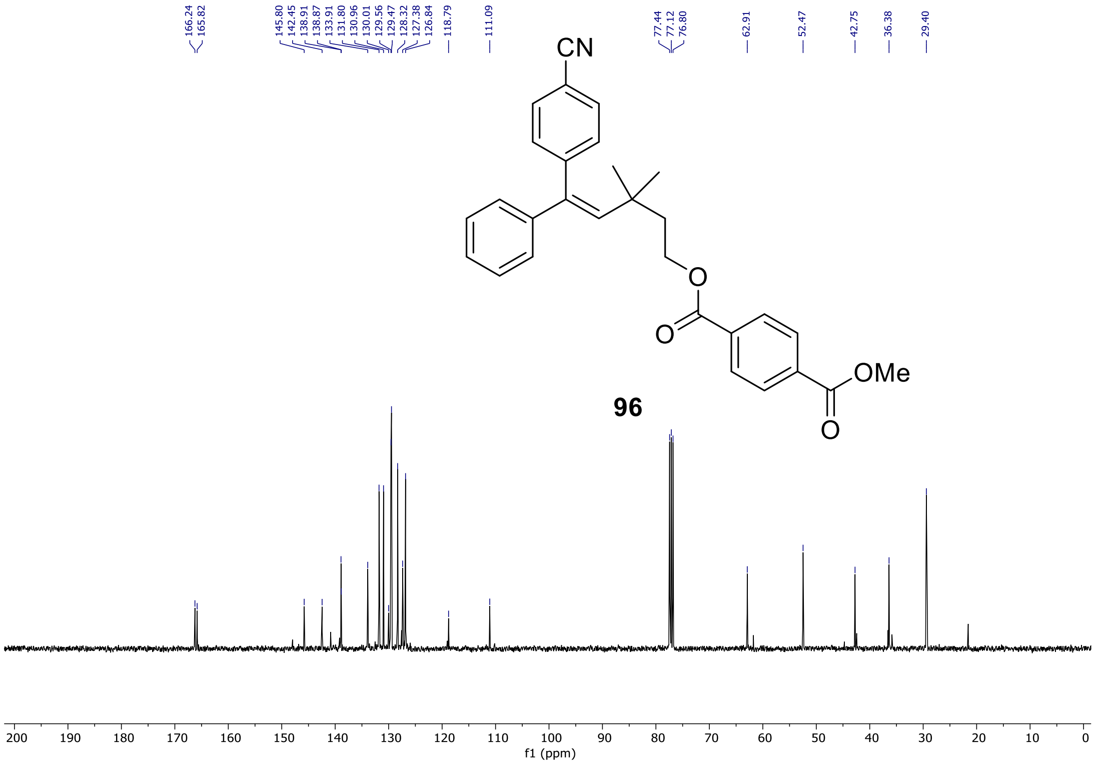

Supplementary Figure 226: ^{13}C NMR spectrum of compound **96**

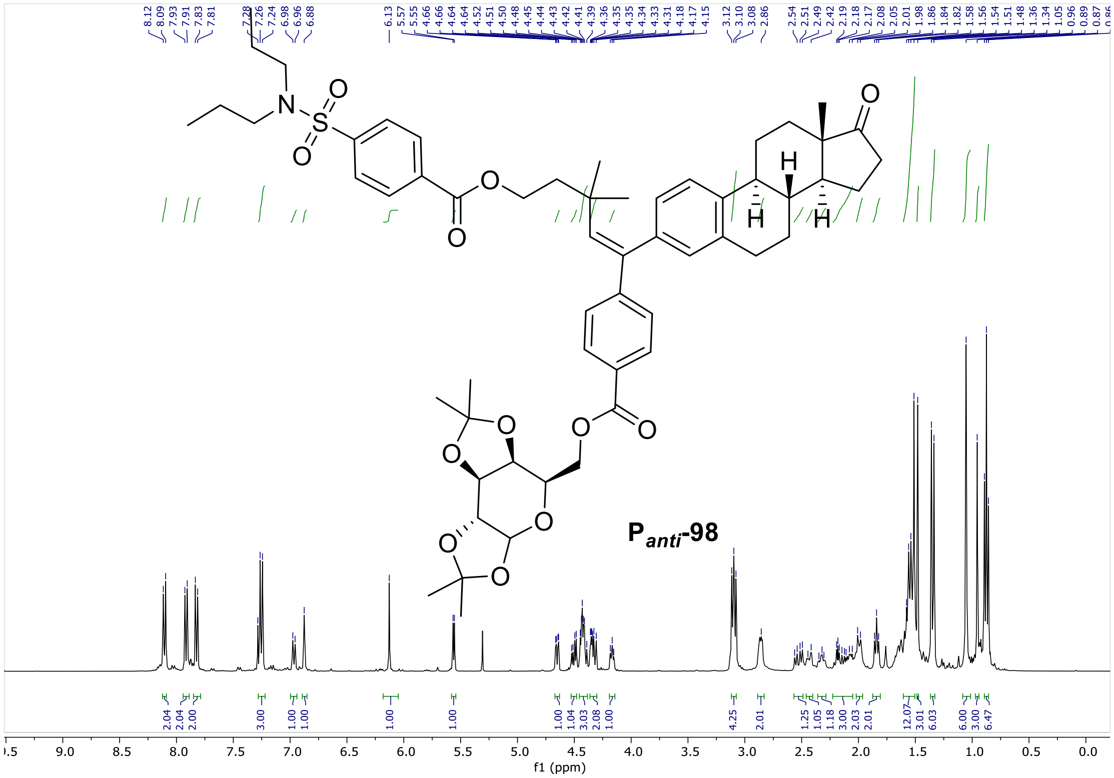

Supplementary Figure 227: ^{1}H NMR spectrum of compound **98**

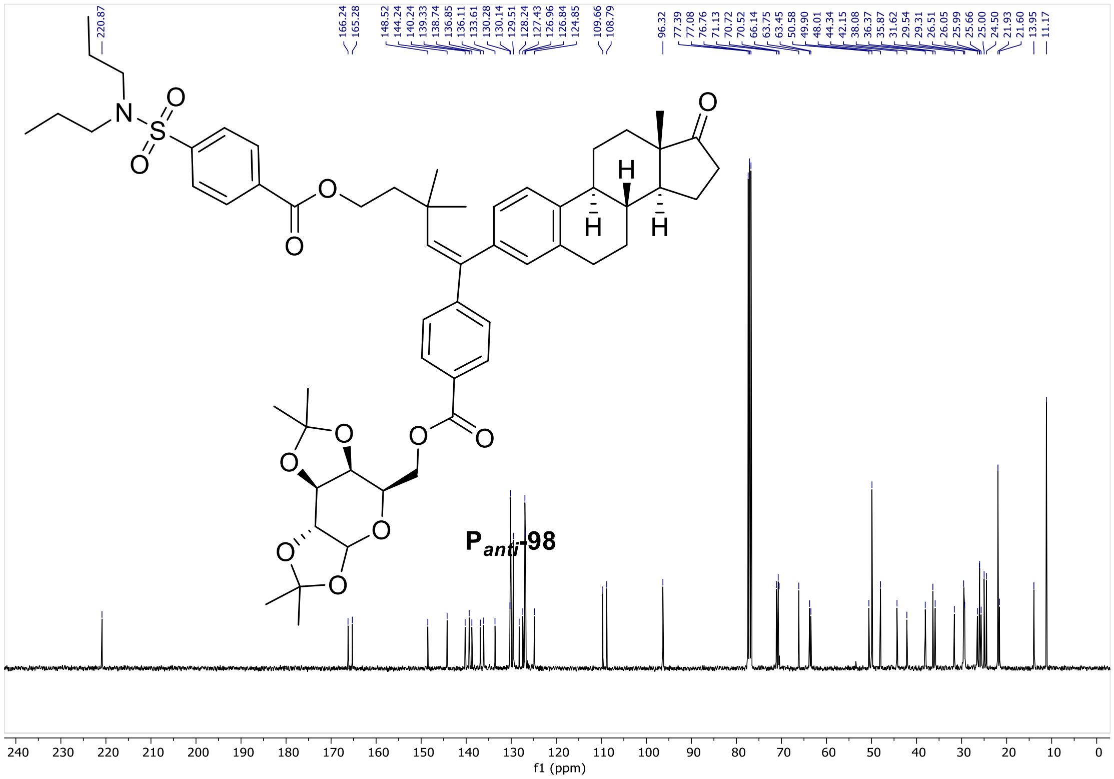

Supplementary Figure 228: ^{13}C NMR spectrum of compound **98**

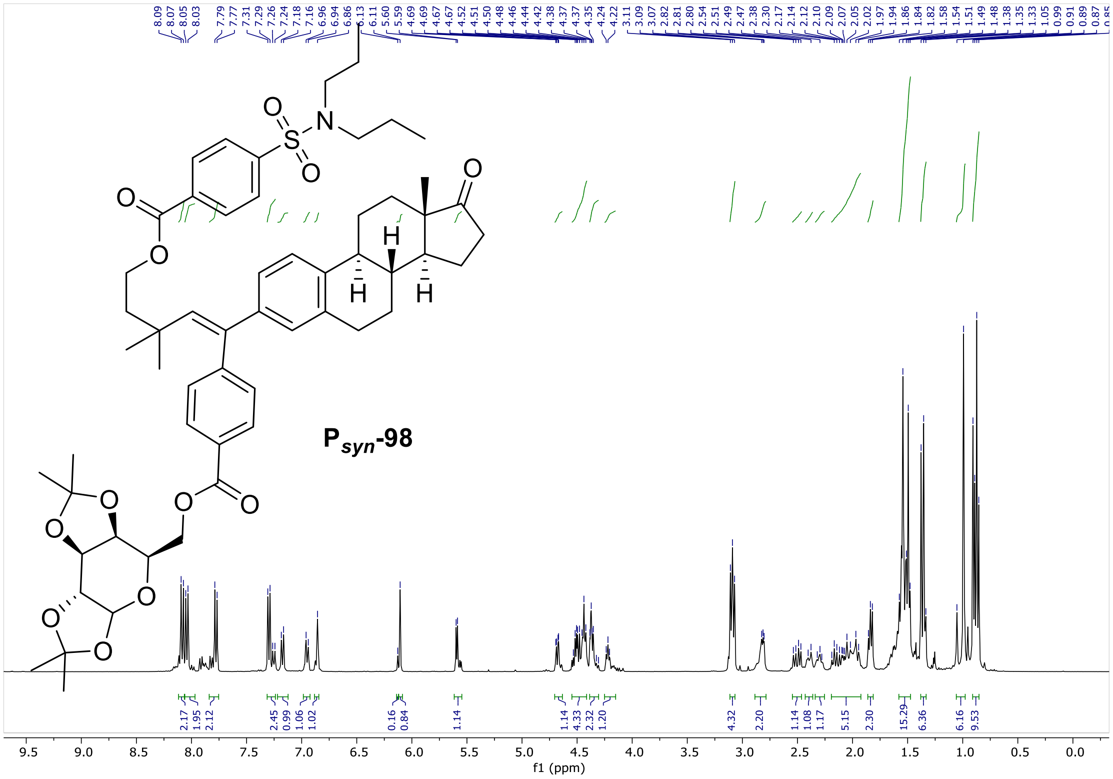

Supplementary Figure 229: ^{1}H NMR spectrum of compound **98**

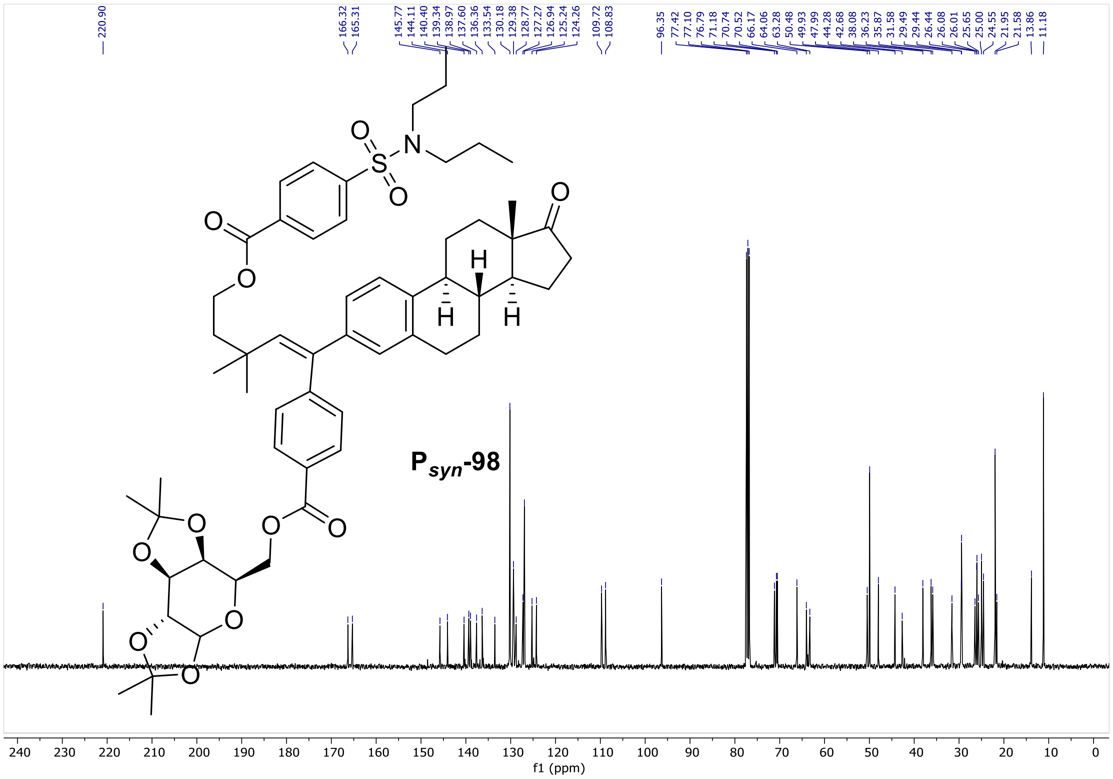

Supplementary Figure 230: ^{13}C NMR spectrum of compound **98**

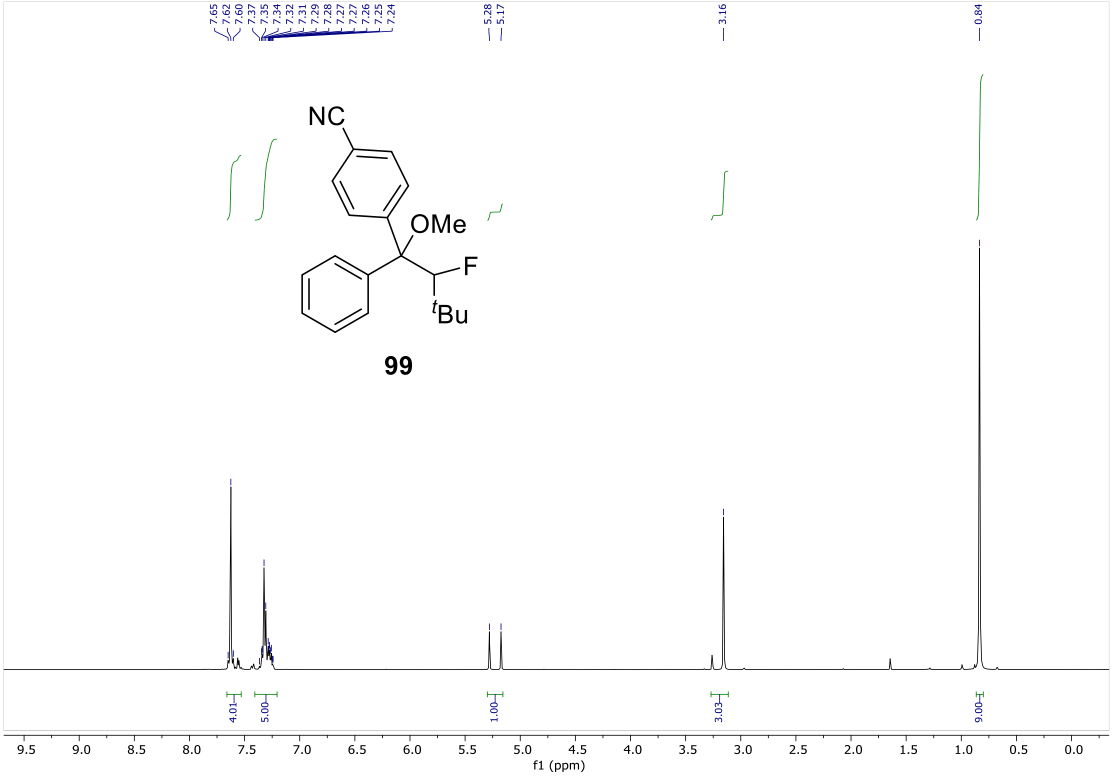

Supplementary Figure 231: ^{1}H NMR spectrum of compound **99**

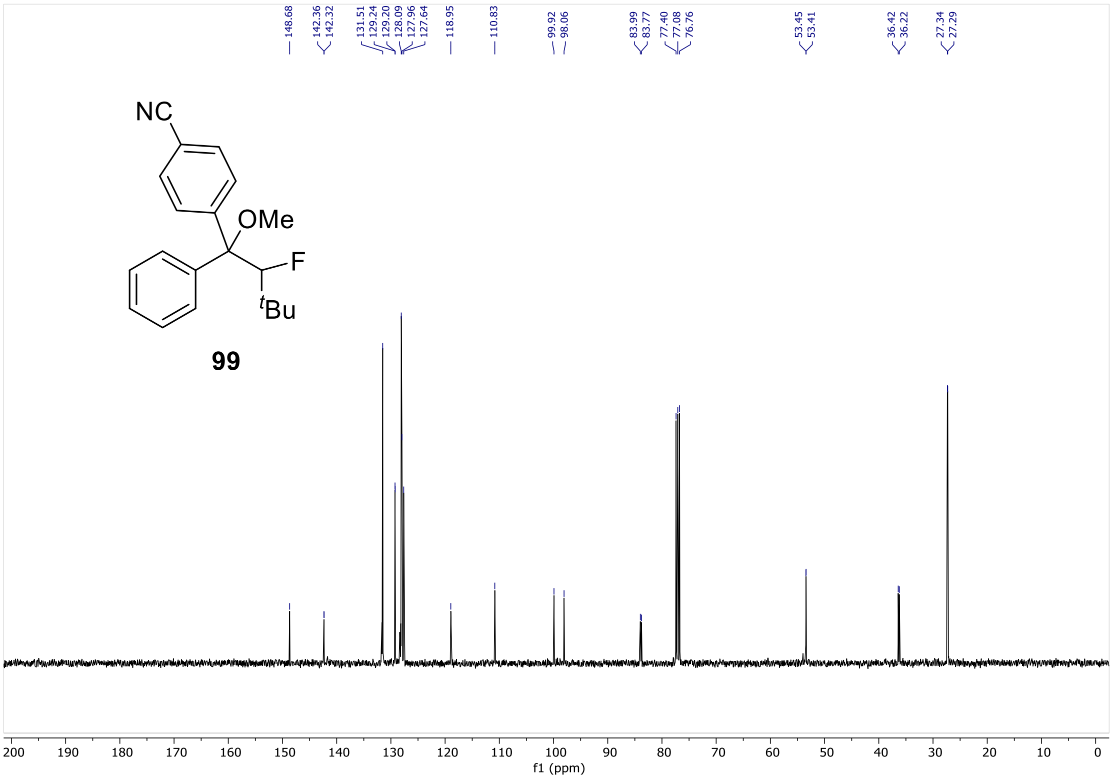

Supplementary Figure 232: ^{13}C NMR spectrum of compound **99**

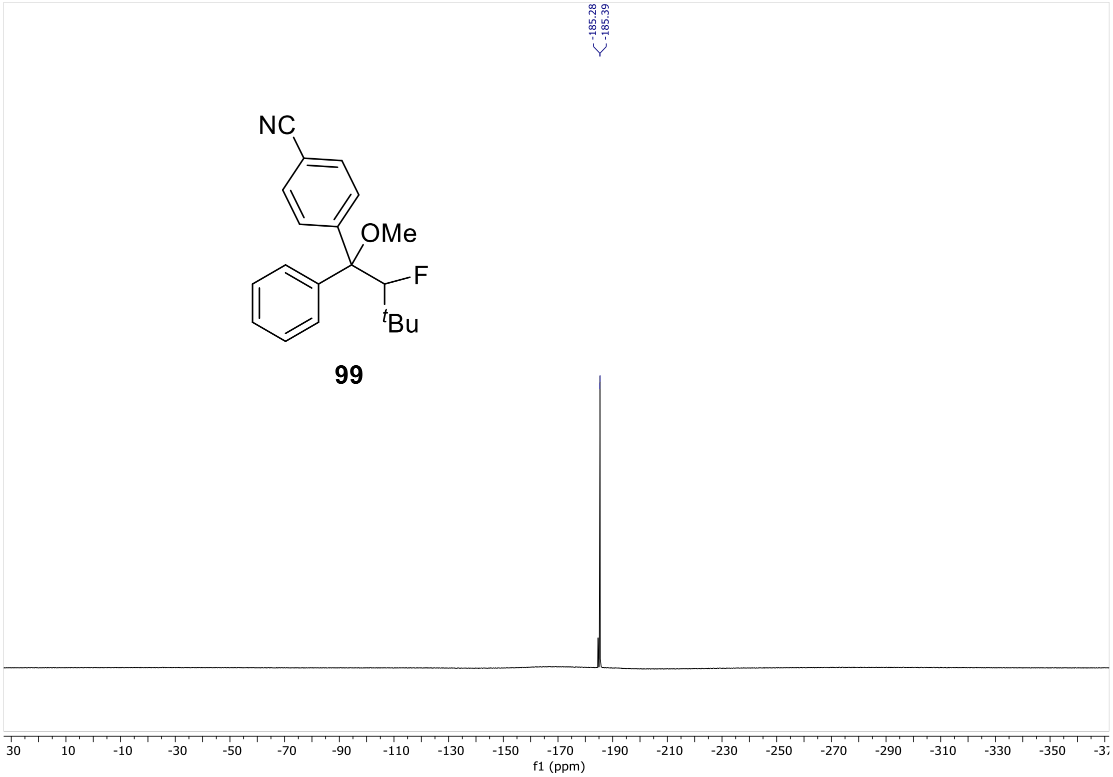

Supplementary Figure 233: ^{19}F NMR spectrum of compound **99**

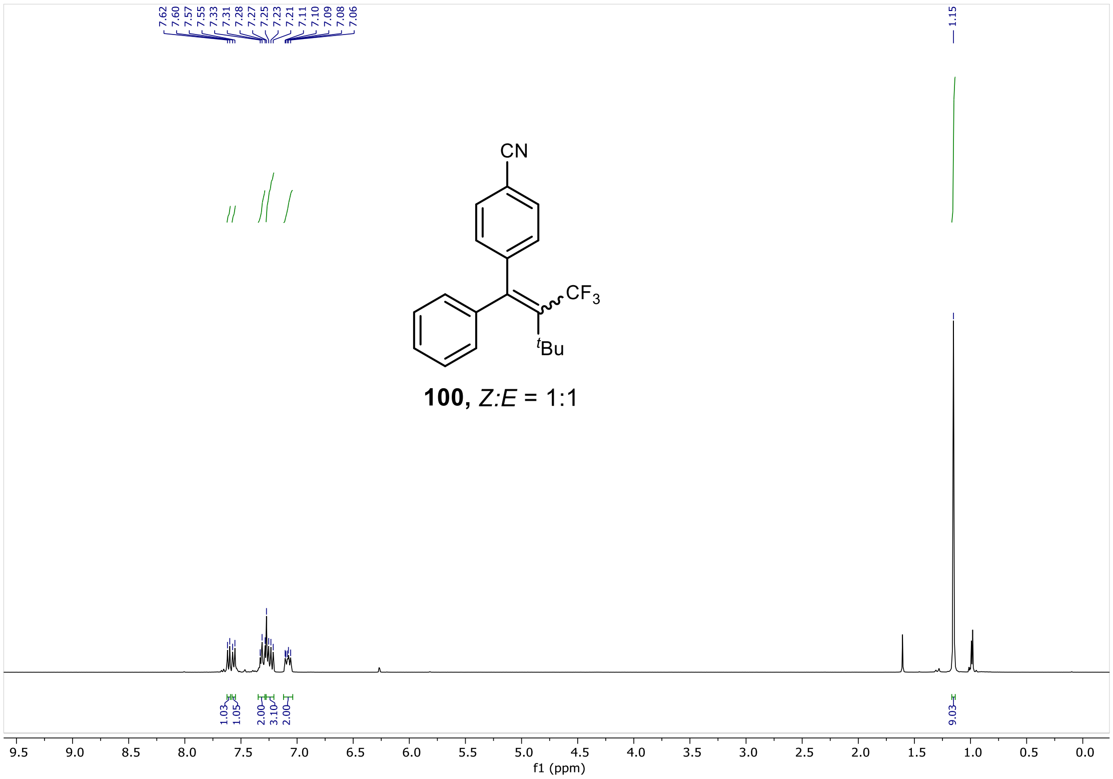

Supplementary Figure 234: ^{1}H NMR spectrum of compound **100**

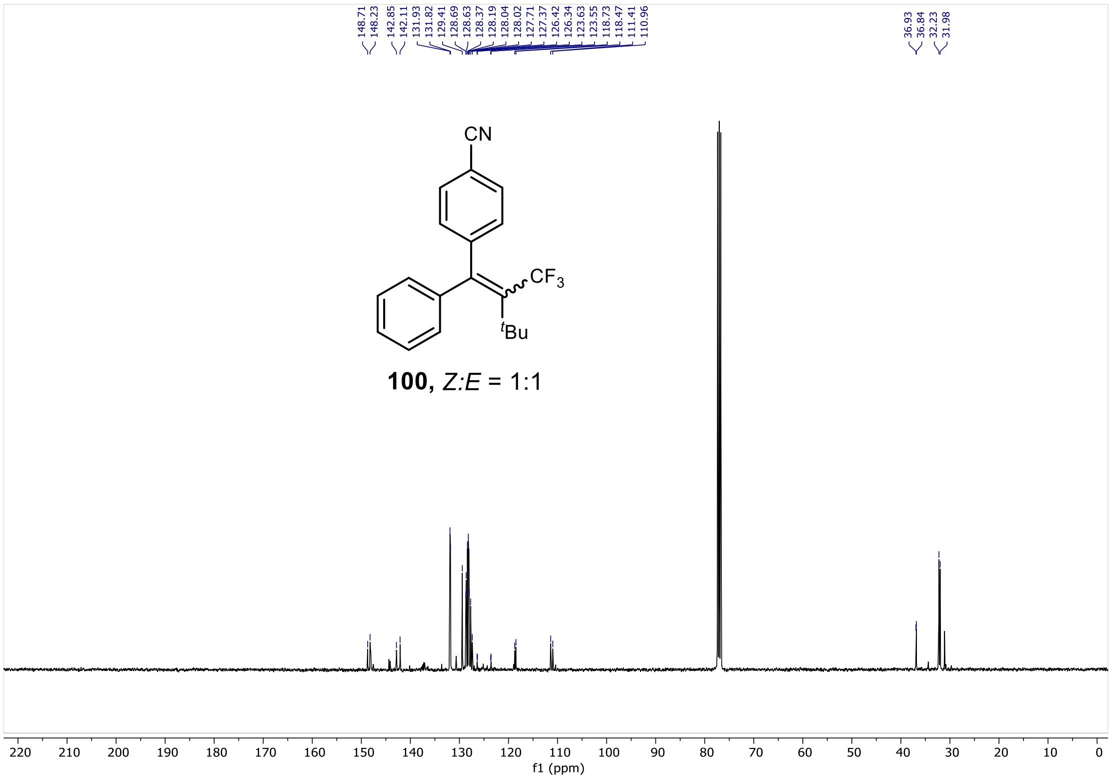

Supplementary Figure 235: ^{13}C NMR spectrum of compound **100**

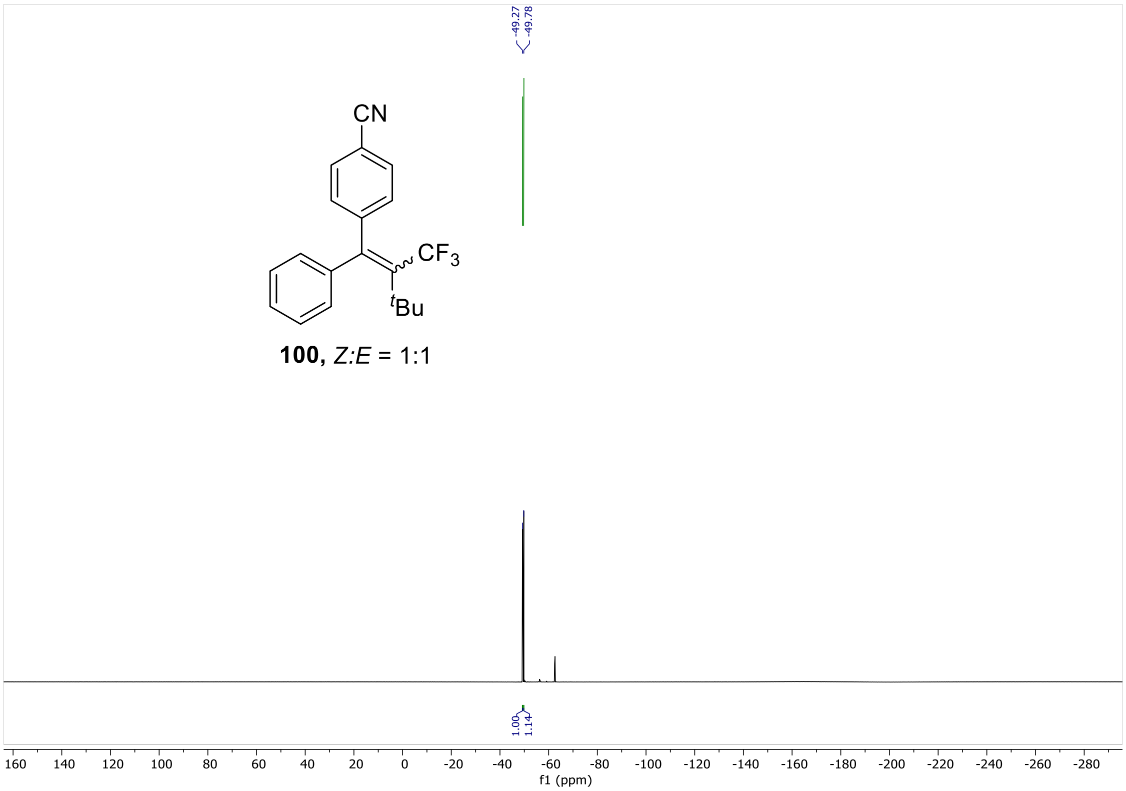

Supplementary Figure 236: ^{19}F NMR spectrum of compound **100**

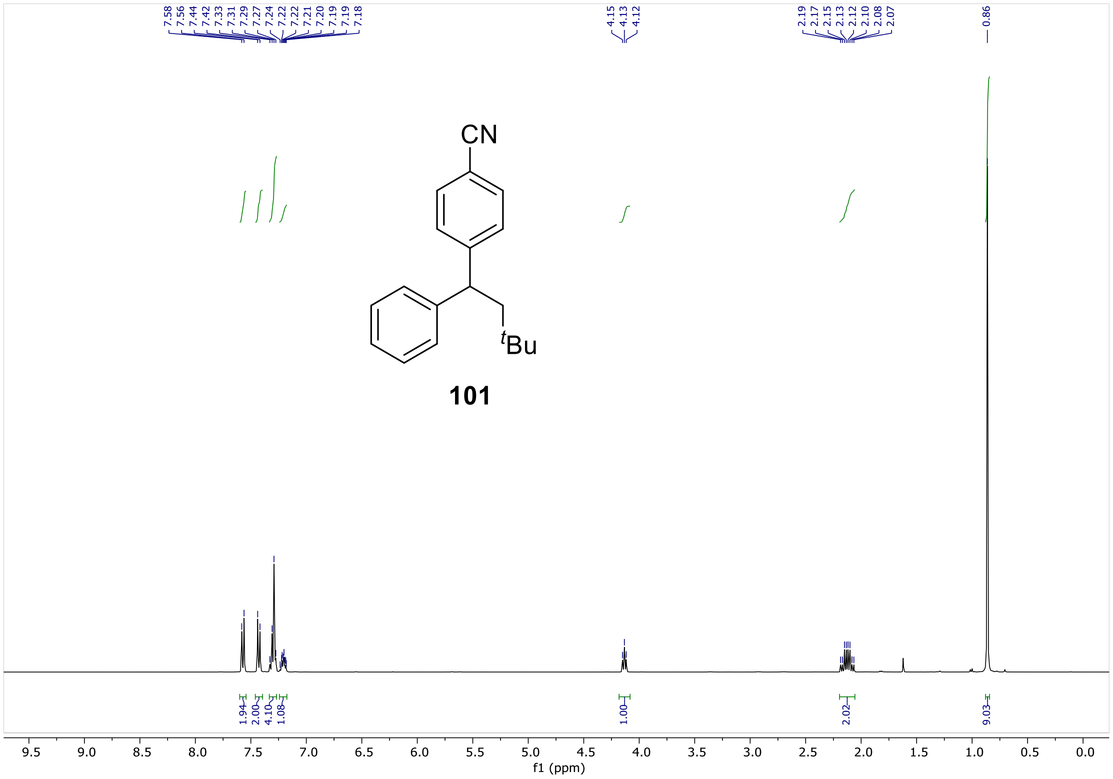

Supplementary Figure 237: ^{1}H NMR spectrum of compound **101**

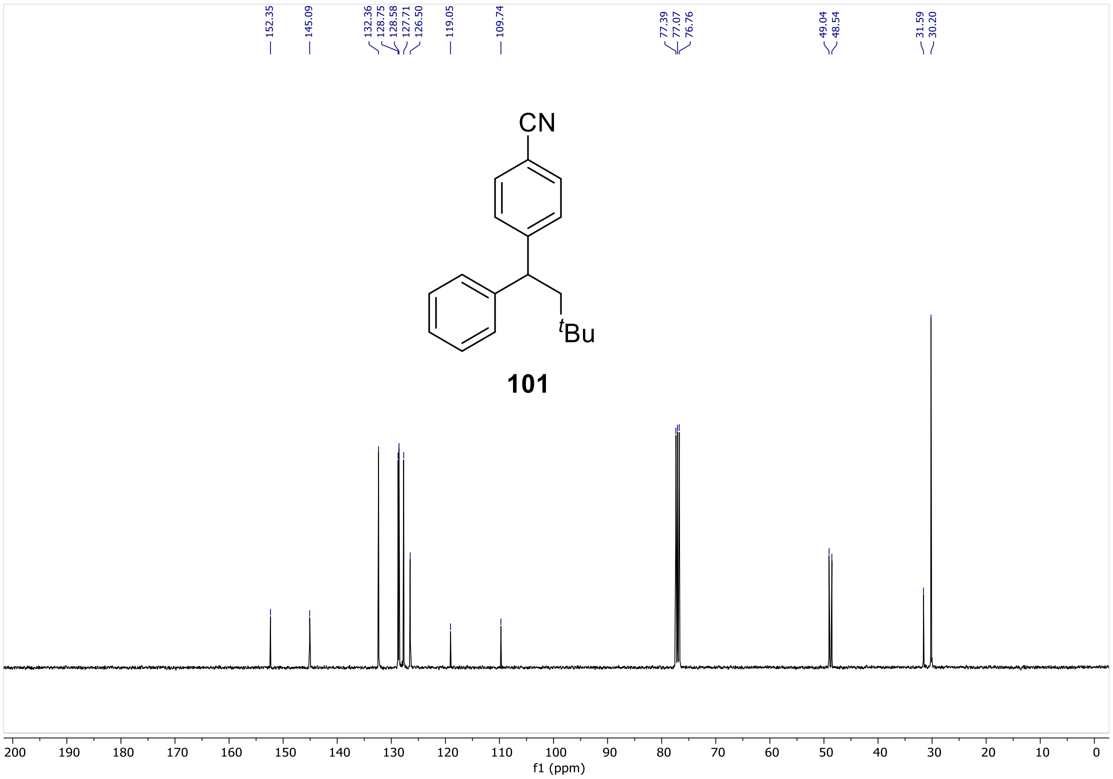

Supplementary Figure 238: ^{13}C NMR spectrum of compound **101**

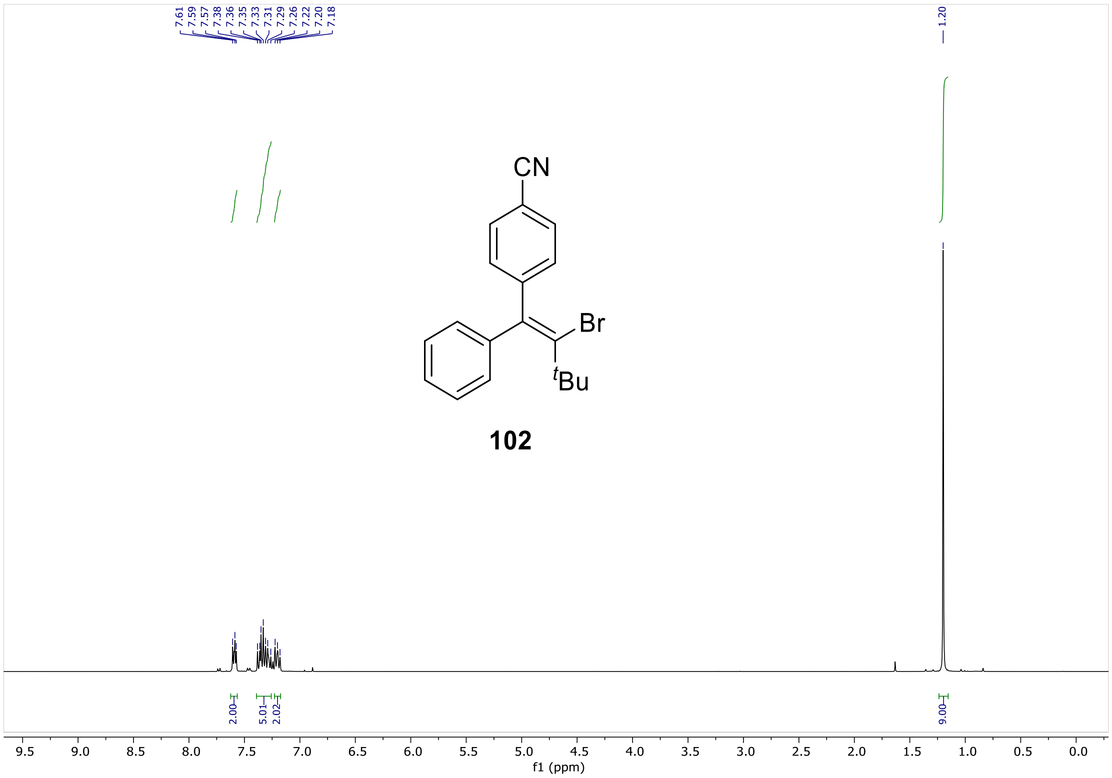

Supplementary Figure 239: ^{1}H NMR spectrum of compound **102**

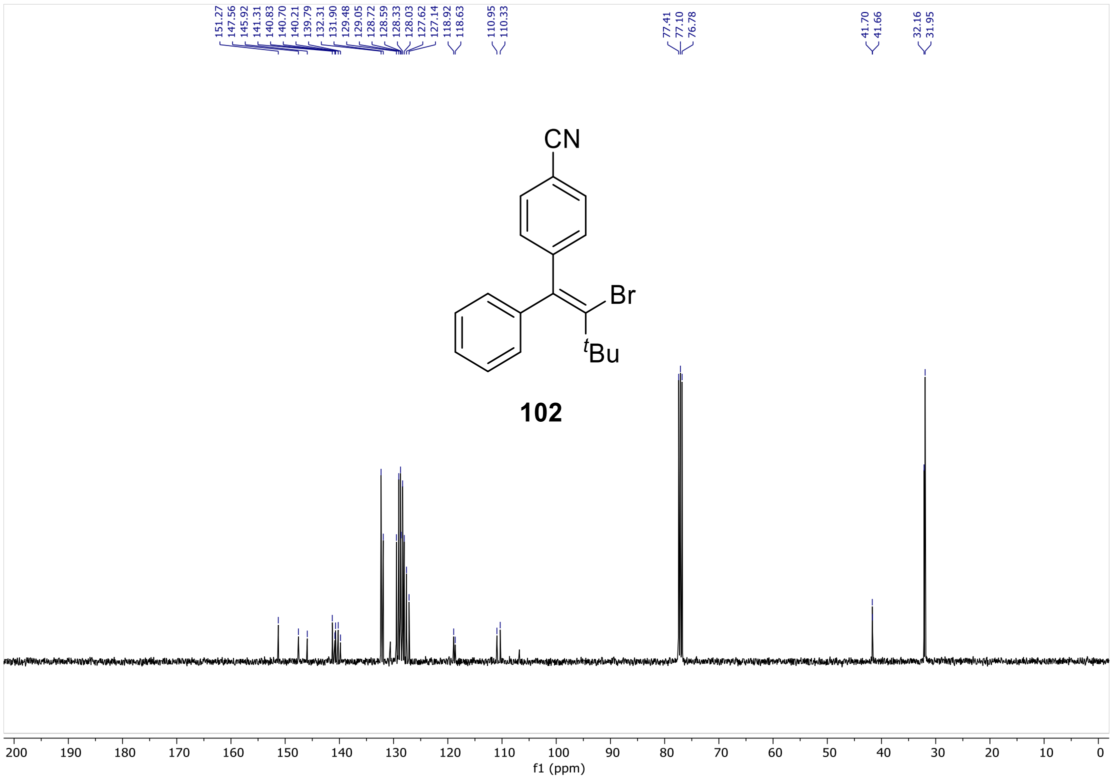

Supplementary Figure 240: ^{13}C NMR spectrum of compound **102**

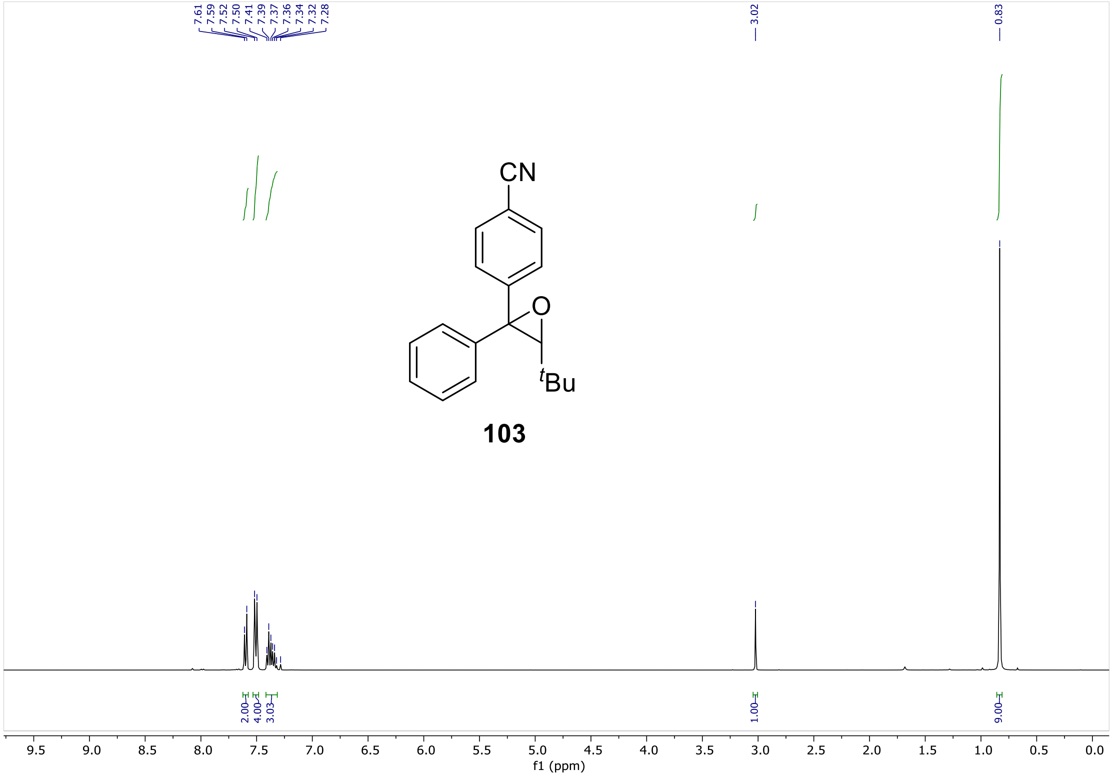

Supplementary Figure 241: ^{1}H NMR spectrum of compound **103**

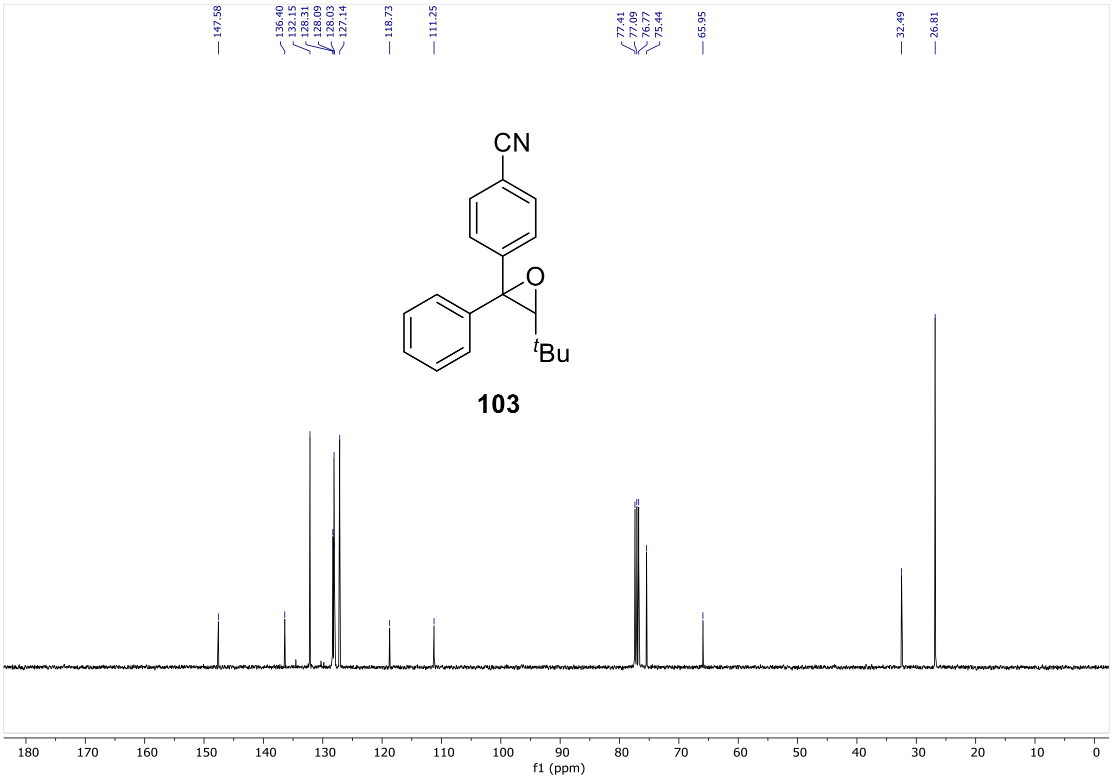

Supplementary Figure 242: ^{13}C NMR spectrum of compound **103**

Supplementary References

[1] N. G. Connelly, W. E. Geiger, *Chem. Rev.* **1996**, *96*, 877-910.

[2] J. L. Brennan, T. E. Keyes, R. J. Forster, *Langmuir* **2006**, *22*, 10754-10761.

[3] a) C. Ye, M. Li, J. Luo, L. Chen, Z. Tang, J. Pei, L. Jiang, Y. Song, D. Zhu, *J. Mater. Chem.* **2012**, *22*, 4299-4305; b) A. Singh, K. Teegardin, M. Kelly, K. S. Prasad, S. Krishnan, J. D. Weaver, *J. Organomet. Chem.* **2015**, *776*, 51-59.

[4] H. Hou, H. Li, Y. Xu, D. Tang, Y. Han, C. Yan, X. Chen, S. Zhu, *Tetrahedron* **2018**, *74*, 6577-6583.

[5] R. Tomita, Y. Yasu, T. Koike, M. Akita, *Beilstein J. Org. Chem.* **2014**, *10*, 1099-1106.

[6] J. Li, L. He, X. Liu, X. Cheng, G. Li, *Angew. Chem. Int. Ed.* **2019**, *58*, 1759-1763.
